# The prevalence of hypoxaemia in paediatric and adult patients in health-care facilities in low-income and middle-income countries: a systematic review and meta-analysis

**DOI:** 10.1016/S2214-109X(24)00469-8

**Published:** 2025-01-29

**Authors:** Hamish R Graham, Esrat Jahan, Rami Subhi, Farhia Azrin, Jaclyn R Maher, Jasmine L Miller, Ahmed Ehsanur Rahman, Felix Lam

**Affiliations:** a Melbourne Children's Global Health, MCRI, University of Melbourne, Royal Children's Hospital, Parkville, VIC, Australia; b International Centre for Diarrhoeal Disease Research, Bangladesh, Dhaka, Bangladesh; c Clinton Health Access Initiative, Boston, MA, USA

## Abstract

**Background:**

Hypoxaemia (low oxygen saturation in blood) is a key predictor of in-hospital mortality, affecting people of all ages with many different conditions. Early detection and treatment of hypoxaemia are critical, but there are few data to quantify hypoxaemia burden outside the child pneumonia population. We aimed to estimate hypoxaemia prevalence for adults and children with acute illness attending health facilities in low-income and middle-income countries (LMICs).

**Methods:**

We conducted a systematic review and meta-analysis, searching MEDLINE, PubMed, Embase, Cumulated Index in Nursing and Allied Health Literature, Index Medicus, and Google Scholar for studies reporting hypoxaemia prevalence among patients attending health facilities. We included articles with original data on peripheral blood oxygen saturation (SpO*_2_*), from an LMIC, published between Jan 1, 1998, and Jan 10, 2023. We included studies in acutely unwell people of any age and with any condition, but excluded those admitted to intensive care units, receiving perioperative care, or attending hospital for preventive or chronic care. We assessed study quality using Joanna Briggs Institute's Checklist for Prevalence Studies. Two reviewers independently conducted title and abstract screening, full-text review, data extraction, and quality assessment, requesting summary data from authors. We reported pooled prevalence of hypoxaemia (typically defined as SpO*_2_* <90%) overall and by condition, using a random-effects meta-analysis model. This study is registered with PROSPERO, CRD42019136622.

**Findings:**

We identified 9173 unique records from searches and included 213 in meta-analyses involving 601 757 participants. The majority of studies were from the World Bank regions of sub-Saharan Africa (108 [51%] of 213) or south Asia (58 [27%]). The pooled prevalence of hypoxaemia among admitted patients was 24·5% (95% CI 19·9–29·4) for neonates (aged 0–28 days), 12·1% (10·0–14·4) for children (aged 1 month–17 years), and 10·8% (4·9–18·7) for adults (aged ≥18 years). Hypoxaemia prevalence was highest in neonatal and primary respiratory conditions but still common in many other conditions. Hypoxaemia was associated with 4·84 (95% CI 4·11–5·69) times higher odds of death than no hypoxaemia.

**Interpretation:**

Hypoxaemia is common across all age groups and a range of primary respiratory and other critical illnesses and is strongly associated with death. These estimates will inform oxygen-related strategies and programmes, and integration of pulse oximetry and oxygen into clinical guidelines, service structures, and strategies for maternal, neonatal, child, adolescent, and adult health.

**Funding:**

Bill & Melinda Gates Foundation, the ELMA Foundation, and Unitaid.

## Introduction

Hypoxaemia (low oxygen saturation in blood) is a physiological sign of respiratory failure that affects patients with a range of acute and chronic illnesses across all ages. It can occur in neonates with sepsis or born preterm, infants with bronchiolitis, and children and adults with pneumonia, bronchiectasis, septicaemia, and other severe systemic illnesses and trauma. Hypoxaemia is strongly associated with increased risk of mortality, irrespective of age or type of illness.^1^, ^2^, ^3^, ^4^, ^5^, ^6^, ^7^

The COVID-19 pandemic highlighted the importance of early identification and treatment of hypoxaemia and stretched existing medical oxygen services to a breaking point in many low-income and middle-income countries (LMICs).^8^ Previous data had shown that medical oxygen services for children were weak and that improving facility oxygen services was a highly effective strategy to reduce child mortality from pneumonia.^9^, ^10^, ^11^, ^12^ A systematic review and meta-analysis published in 2021 quantified this, reporting a pooled odds ratio of 0·52 (95% CI 0·39–0·70) in favour of oxygen systems reducing in-hospital mortality due to childhood pneumonia, at a median cost of US$62 per disability-adjusted life-year averted (ie, highly cost-effective).^13^ However, as health workers, hospitals, local authorities, and global health agencies responded to the COVID-19 pandemic and the growing need for medical oxygen, quantification exercises revealed major gaps in understanding baseline oxygen needs or capacity to respond.^14^

Given that hypoxaemia is the key reason to administer oxygen therapy in acute illness, reliable data on hypoxaemia prevalence are crucial to inform estimates of oxygen needs. Previous systematic reviews have reported hypoxaemia prevalence in children with pneumonia.^15^, ^16^, ^17^ The largest and most recent of these reviews, published in 2022, reported pooled hypoxaemia prevalence of 31% (95% CI 26–36; 57 studies; 101 775 children) for children with WHO-classified pneumonia or severe pneumonia.^17^ However, although we know that many patients without pneumonia also require oxygen therapy, few studies have assessed hypoxaemia prevalence for populations with non-pneumonia conditions or outside the early childhood period. Subhi and colleagues’ 2009 systematic review identified only six studies in populations with non-pneumonia conditions, reporting median hypoxaemia prevalence of 19·2% (IQR 18·2–21·4; four studies, 2464 participants) among sick neonates, 9·5% (2·7–14·6; three studies, 136 participants) among children with meningitis, 4·1% (2·9–3·1; four studies, 6799 participants) among children with malaria, and 2·3% (1·1–3·6; four studies, 2802 participants) among children with diarrhoeal disease.^16^ There has been no synthesis of data on hypoxaemia prevalence for adolescent or adult populations.

**Research in context**

**Evidence before this study**

Three previous systematic reviews have assessed hypoxaemia prevalence among children, primarily focusing on children younger than 5 years with pneumonia. In 2001, Lozano reported that the prevalence of hypoxaemia among young children with pneumonia was 6% in outpatient settings, 31% in emergency departments, and 47% in hospital ward settings. In 2009, Subhi and colleagues reported that the prevalence of hypoxaemia among children hospitalised with WHO-classified pneumonia was 13% (IQR 9·3–37·5; 21 studies or datasets), among neonates was 19% (IQR 18–21; four studies), and was generally lower (but variable) among children with non-pneumonia conditions. In 2022, Rahman and colleagues reported pooled hypoxaemia prevalence of 31% (95% CI 26–36; 57 studies; 101 775 children) among children with WHO-classified pneumonia or severe pneumonia. Lazzerini and colleagues reported increased odds of death among young children with hypoxaemic pneumonia compared with those without hypoxaemia (odds ratio 5·47, 95% CI 3·93–7·63). We searched MEDLINE (PubMed), Embase, and Global Health via Ovid for systematic reviews on “hypoxaemia” and “prevalence” published between Jan 1, 2000, and Oct 31, 2024, without language restriction.

**Added value of this study**

This is the first synthesis of hypoxaemia data across all ages and all acute medical conditions, providing estimates of hypoxaemia prevalence for a range of respiratory and non-respiratory conditions. We reviewed more than 9000 articles to identify 213 studies for inclusion in a quantitative analysis involving 601 757 participants. We found that hypoxaemia affected a quarter of neonates (24·5%, 95% CI 19·9–29·4), one in eight children (12·1%, 10·0–14·4), and one in ten adults (10·8%, 4·9–18·7) admitted to hospital. We disaggregated the estimates by age, condition, facility level, geography, and altitude, providing the most comprehensive hypoxaemia estimates to date.

**Implications of all the available evidence**

Hypoxaemia is common among patients of all ages, multiple conditions, and at every level of care, and is strongly associated with death. Early detection of hypoxaemia using pulse oximetry must be prioritised by governments and health agencies globally, alongside improving access to oxygen therapy at, and referral pathways to, admitting health facilities. These hypoxaemia prevalence estimates provide a foundation for quantifying the global need for oxygen and inform national and global strategies and programmes seeking to improve coverage of oxygen services.

We aimed to estimate and describe the prevalence of hypoxaemia among patients of all ages attending health-care facilities in LMICs. This analysis also supports oxygen need estimation for the *Lancet Global Health* Commission on medical oxygen security.^18^

## Methods

### Search strategy and selection criteria

For this systematic review and meta-analysis, we followed the PRISMA guidelines for reporting.^19^ Our study protocol is published^20^ and registered at PROSPERO, CRD42019136622.

We searched MEDLINE, PubMed, Embase, Cumulated Index in Nursing and Allied Health Literature, Index Medicus, and Google Scholar (first 500 results) for studies reporting hypoxaemia prevalence among patients attending health facilities using search terms including “hypoxaemia” AND “oximetry/measurement” AND “low- and middle-income countries” (appendix pp 7–9). We included studies that were published between Jan 1, 1998, and Jan 10, 2023, and that contained original data on hypoxaemia prevalence among patients attending a health facility in an LMIC (defined using the World Bank classification).^21^ We assessed hypoxaemia according to peripheral blood oxygen saturation (SpO_2_) measured by any health-care worker, recognising that pulse oximetry is the primary method for detecting hypoxaemia in routine care (especially in resource-constrained, non-intensive care settings).^22^ We included studies in acutely unwell people of any age and with any condition but excluded patients receiving treatment in intensive care units, receiving perioperative care, or attending hospital for preventive or chronic care (eg, immunisation and prevention of mother-to-child transmission). We accepted any study design but excluded unpublished data. We included articles in any language and used online document translation tools to translate into English for analysis.

Our study team included topic experts in hypoxaemia (HRG, RS, AER, and FL) and a statistician (FA), with assistance from a senior research librarian (Poh Chua, Royal Children's Hospital, University of Melbourne, Melbourne, VIC, Australia). We used the Covidence (Melbourne, VIC, Australia) online systematic review management platform to manage the review. Duplicate articles were identified automatically using Covidence, with manual secondary checking. Each title and abstract was independently screened by two reviewers from our author pool, who selected all potentially eligible articles for full-text review. Two reviewers independently reviewed full texts for eligibility, with disagreement resolved through discussion with a third reviewer (HRG or RS). To maximise capture of hypoxaemia prevalence among under-reported non-pneumonia populations, we contacted authors of articles in which SpO_2_ data were present but inadequately reported (eg, reported mean SpO_2_ without numerator and denominator to calculate prevalence) if they were recent (2017 onwards) and not small (≥100 participants). We contacted authors by email at least twice.

### Data analysis

Two members of the team independently extracted data into a customised form in Covidence, with consensus by a third reviewer (HRG or RS). Where multiple studies reported the same or overlapping populations, we merged studies and used the most comprehensively reported data (including data for subpopulations). Two independent reviewers assessed risk of bias of included studies using the Joanna Briggs Institute's Checklist for Prevalence Studies, a widely used tool that was specifically designed for prevalence studies.^23^, ^24^ We reported risk of bias of each individual study, but did not exclude from or adjust analysis based on study quality.

Our primary outcome of interest was hypoxaemia prevalence (extracting numerator and denominator), and we included subgroup data on all populations for which they were available. Our secondary outcome of interest was relative odds of death in people with versus without hypoxaemia (extracting number of deaths and total cases for those with and without hypoxaemia). Additional variables are detailed in the appendix (pp 15–16).

As hypoxaemia prevalence is highly variable across population groups, we chose a random-effects model to calculate pooled prevalence of hypoxaemia with 95% CIs,^25^ using the Stata (version 18) metaprop command.^26^ We reported heterogeneity using τ^2^ and *I*^2^. For subgroups with fewer than three included studies, we reported study-specific data without meta-estimates.

We stratified the analyses by population, reflecting patients requiring admission (typically hospitalised inpatients) versus the broader population presenting with acute illness (typically attending outpatient or emergency departments), and by age (neonates 0–28 days, children 1 month–17 years, and adults ≥18 years). We categorised by condition, geographical region (World Bank geographical classifications), and altitude (high: ≥1500 m above sea level, low: <1500 m above sea level, and mixed). We were unable to further stratify by adolescent or adult age subgroups or by sex due to lack of disaggregation in the primary data. The lead analysts (EJ and FA) conducted the initial data checking and subgroup classification, and this was checked by the senior clinical supervisors (HRG and AER) to determine the final categories for analysis. We calculated relative odds of death with and without hypoxaemia using data from studies that reported in-hospital mortality data disaggregated by hypoxaemia. We generated forest plots for each group and then constructed tables and horizontal bar charts to display meta-estimates across multiple groups.

We conducted post-hoc sensitivity analyses to assess robustness of the synthesised results to differences in the hypoxaemia definition used by included studies (restricting to SpO_2_ <90%) and altitude (restricting to <1500 m above sea level). We chose SpO_2_ of less than 90% because this is the most commonly used hypoxaemia definition, recognising that this reflects severe hypoxaemia that typically warrants oxygen therapy.^22^ We did not plan specific analyses to assess risk of bias due to missing results because we considered publication bias unlikely for a non-interventional effect outcome such as hypoxaemia prevalence.^23^ In the absence of formal guidance on assessing certainty in prevalence meta-analysis,^23^ we conducted post-hoc assessment of certainty of estimates using an adapted GRADE approach (detailed in the appendix p 68). We used Stata (version 18) for data cleaning and statistical analysis.

### Role of the funding source

The funders of the study had no role in study design, data collection, data analysis, data interpretation, or writing of the report.

## Results

**Figure 1:**
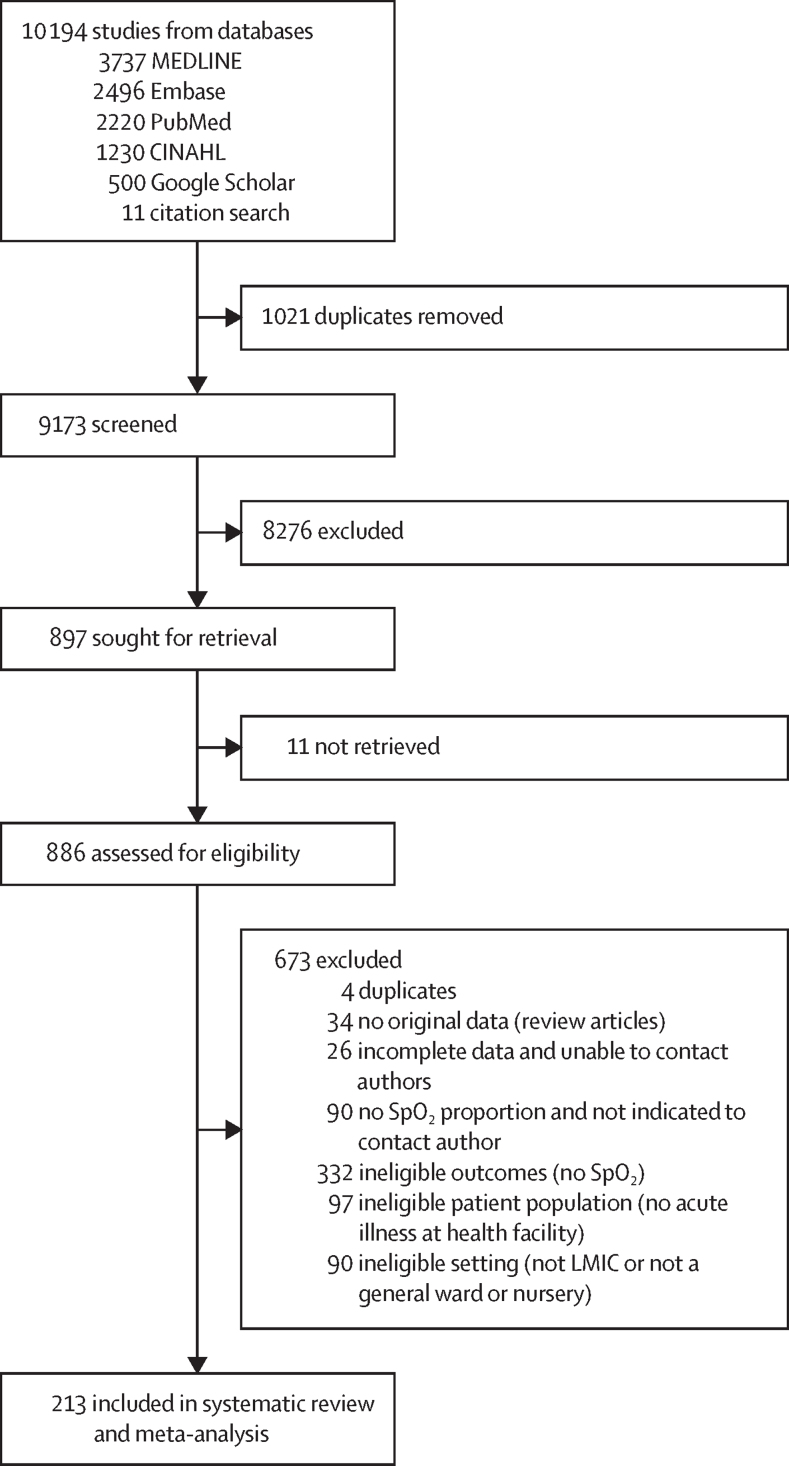
Study selection CINAHL=Cumulated Index in Nursing and Allied Health Literature. LMIC=low-income or middle-income country. SpO_2_=peripheral blood oxygen saturation.

**Figure 2:**
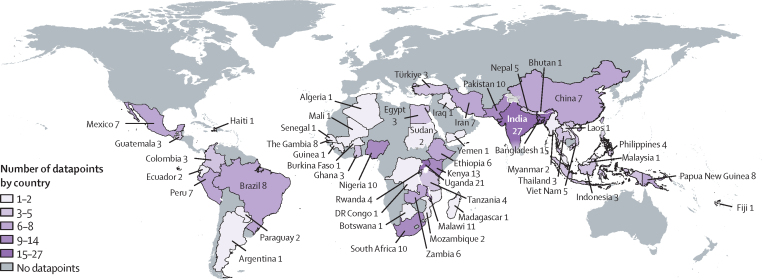
Distribution of the studies included in the meta-analysis by country

We screened 9173 unique articles, identifying 897 for full-text review and 213 for inclusion in quantitative synthesis (figure 1; appendix pp 69–97). The included studies had 601 757 participants, including 16 354 neonates (62 [29%] of 213 studies), 268 375 children (145 [67%] studies), and 212 955 adults (75 [35%] studies). The remaining individuals could not be accurately disaggregated. 108 (51%) studies were from sub-Saharan Africa (particularly east, west, and southern Africa) and 58 (27%) were from south Asia (figure 2), and the majority of studies represented low-altitude populations attending tertiary care facilities (table). Although 140 (66%) studies reported hypoxaemia as SpO_2_ of less than 90%, four (2%) used much lower cutoff points (eg, <85%) and 39 (18%) used much higher cutoff points (eg, <95%), with the latter including some large studies on COVID-19 (table). Few studies set out to estimate hypoxaemia prevalence, but overall study quality was generally moderate to high (appendix pp 42–66).

**Table:** Distribution of the studies included in the meta-analysis by background characteristics

	**Number of studies**	**Number of participants**
**Region (World Bank)**		
East Asia and Pacific	35	45 737
Europe and central Asia	3	1682
Latin America and Caribbean	35	216 181
Middle East and north Africa	13	13 181
South Asia	58	56 859
Sub-Saharan Africa	108	351 892
**Altitude**		
High (≥1500 m above sea level)	25	22 985
Low (<1500 m above sea level)	182	559 313
Mixed	6	19 459
**Facility type**		
Level 1 (primary care clinic)	17	37 327
Level 2 (secondary facility; eg, general hospital)	33	249 272
Level 3 (tertiary facility; eg, referral hospital)	135	93 559
Multiple or other	28	221 599
**Hypoxaemia reporting definition**		
SpO_2_ <93%, <94%, <95%, or <96%	39	202 824
SpO_2_ <91% or <92%	19	19 656
SpO_2_ <90%	140	367 433
SpO_2_ <88% or <89%	3	5002
SpO_2_ <85%, <86%, or <87%	4	5496
Missing	8	1346

SpO_2_=peripheral blood oxygen saturation.

**Figure 3:**
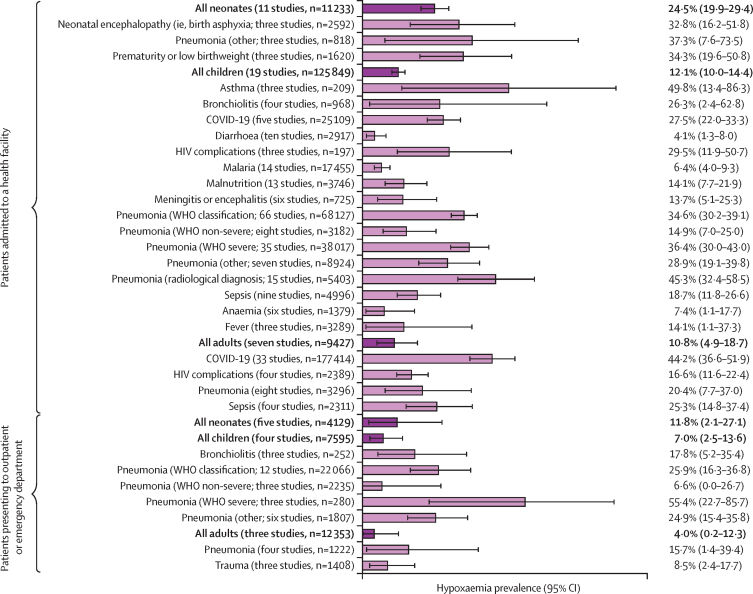
Hypoxaemia prevalence among hospitalised neonates, children, and adults, reporting random-effects pooled estimates across different subgroups Hypoxaemia was typically defined as peripheral blood oxygen saturation of less than 90%. Neonates were aged 0–28 days, children 1 month–17 years, and adults 18 years or older. Error bars show 95% CIs.

Hypoxaemia was common among acutely unwell neonates, children, and adults presenting with a wide range of conditions in LMICs (figure 3; appendix pp 17, 135). Among neonates admitted to hospital, the pooled prevalence of hypoxaemia was 24·5% (95% CI 19·9–29·4), including neonates with neonatal encephalopathy (32·8%, 16·2–51·8), prematurity or low birthweight (34·3%, 19·6–50·8), pneumonia (37·3%, 7·6–73·5), and neonatal sepsis (range 21·0–44·1% from two studies). The prevalence of hypoxaemia among admitted neonates was similar at secondary and tertiary health facilities (21·2%, 95% CI 17·5–25·2, *vs* 26·7%, 16·7–38·0; appendix p 35). Among the broader population of neonates presenting to a health facility but not necessarily requiring admission, 11·8% (95% CI 2·1–27·1) had hypoxaemia.

Among children admitted to hospital, the pooled prevalence of hypoxaemia was 12·1% (95% CI 10·0–14·4). Hypoxaemia was most common among children admitted with a primary respiratory illness: WHO-classified pneumonia (34·6%, 95% CI 30·2–39·1), asthma (49·8%, 13·4–86·3), bronchiolitis (26·3%, 2·4–62·8), and COVID-19 (27·5%, 22·0–33·3). Hypoxaemia was also common for other acute infections and conditions: sepsis (18·7%, 95% CI 11·8–26·6), meningitis or encephalitis (13·7%, 5·1–25·3), malnutrition (14·1%, 7·7–21·9), and HIV complications (29·5%, 11·9–50·7). Nine studies provided data on both hypoxaemia prevalence for child pneumonia and children overall and suggested that about 50% (range 27–81) of the overall burden is related to non-pneumonia conditions (appendix p 35). The prevalence of hypoxaemia among admitted children was similar at secondary and tertiary health facilities (12·5%, 95% CI 10·0–15·2, *vs* 12·2%, 6·2–19·7; appendix p 35). Among the broader population of children presenting to a health facility, 7·0% (95% CI 2·5–13·6) had hypoxaemia, including 25·9% (16·3–36·8) of children diagnosed with pneumonia on presentation.

Among adults admitted to hospital, the pooled prevalence of hypoxaemia was 10·8% (95% CI 4·9–18·7). Hypoxaemia was most common among adults with pneumonia (20·4%, 95% CI 7·7–37·0), COVID-19 (44·2%, 36·6–51·9), sepsis (25·3%, 14·8–37·4), and HIV complications (16·6%, 11·6–22·4; figure 3). Although the prevalence of hypoxaemia was lower among the broader population of adults presenting to hospital (4·0%, 95% CI 0·2–12·3), it was observed in 15·7% (1·4–39·4) of those presenting with pneumonia and 8·5% (2·4–17·7) of those presenting with trauma.

**Figure 4:**
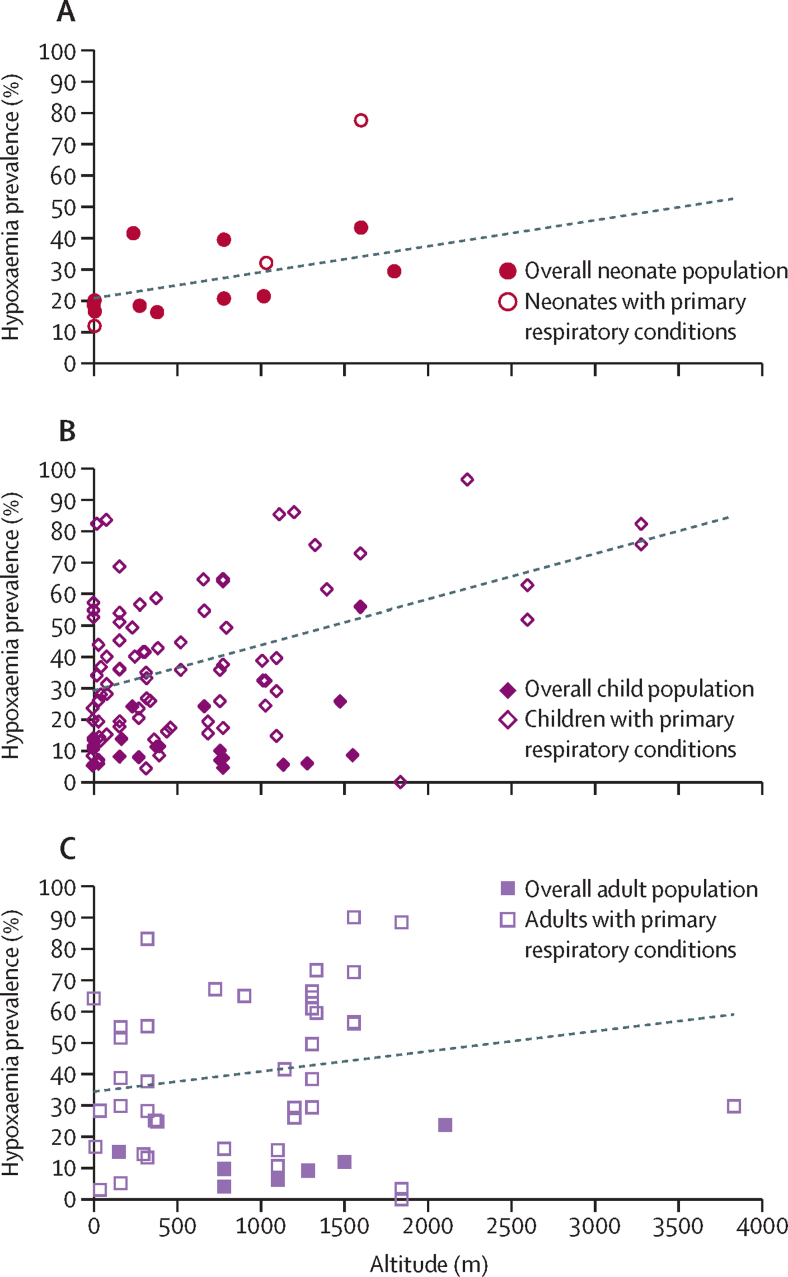
Scatterplot showing hypoxaemia prevalence among hospitalised neonates (A), children (B), and adults (C) at different altitude Neonates were aged 0–28 days, children 1 month–17 years, and adults 18 years or older. The linear line of best fit for neonates overall and children and adults with primary respiratory conditions is shown.

**Figure 5:**
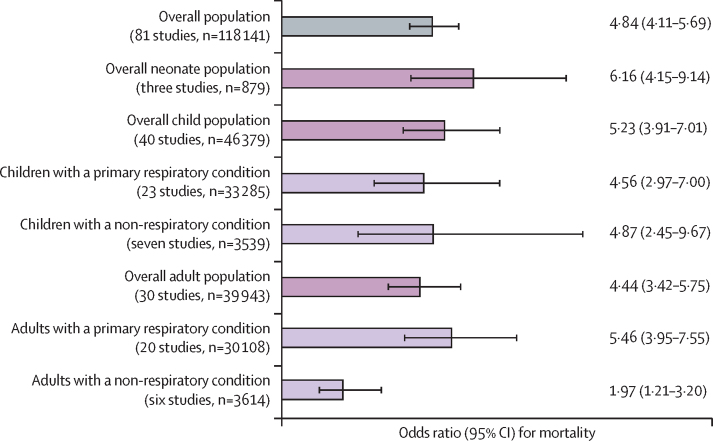
Relative odds of death comparing patients with hypoxaemia versus those without hypoxaemia Meta-estimates are presented for subgroups with three or more included studies. Hypoxaemia was typically defined as peripheral blood oxygen saturation of less than 90%, with odds of death compared with those with peripheral blood oxygen saturation of 90% or more. Neonates were aged 0–28 days, children 1 month–17 years, and adults 18 years or older. Error bars show 95% CIs.

Hypoxaemia prevalence generally increased with higher altitude (figure 4). In a subgroup analysis of children with pneumonia, the estimated pooled prevalence of hypoxaemia at high altitude (≥1500 m above sea level) was 66·8% (95% CI 51·1–80·9) versus 32·6% (28·2–37·2) at lower altitude (appendix p 229). However, this analysis included only three studies at high altitude, two of which used lower SpO_2_ cutoff values to define hypoxaemia (<88% or 89%). Absence of comparative data prevented additional subgroup analyses of altitude. Hypoxaemia prevalence in children admitted with pneumonia was similar in the sub-Saharan Africa (34·5%, 95% CI 26·9–42·5), south Asia (39·1%, 33·7–44·7), Latin America and Caribbean (45·1%, 19·3–72·4), and east Asia and Pacific (26·3%, 16·6–37·4) regions. Absence of comparative data prevented meaningful geographical disaggregation for additional subgroups. Neonates, children, and adults with hypoxaemia had four to six times higher odds of in-hospital death than those without hypoxaemia (pooled odds ratio 4·84, 95% CI 4·11–5·69; figure 5).

Neonatal, child, and adult hypoxaemia prevalence meta-estimates were robust to sensitivity analyses that were restricted to studies that reported hypoxaemia as SpO_2_ of less than 90% (appendix p 29). For adults with COVID-19, the sensitivity analysis included nine of the 33 studies from the primary analysis (n=7326) but found similar high prevalence of hypoxaemia (49·3%, 95% CI 24·7–74·0).

Overall, our certainty in the hypoxaemia prevalence evidence (assessed using adapted GRADE criteria) was affected by low precision of many of the estimates outside the largest subgroups, and moderate to high heterogeneity, with individual prevalence estimates widely dispersed in both directions from the pooled estimates (assessment of certainty detail is in the appendix p 68).

## Discussion

This study reports updated estimates of hypoxaemia prevalence for children and new estimates of hypoxaemia prevalence for adult populations in LMICs. We found that hypoxaemia was common among acutely unwell patients of all ages attending health facilities in LMICs, affecting 24·5% of admitted neonates, 12·1% of admitted children, and 10·8% of admitted adults. As expected, hypoxaemia was very common in patients with acute primary respiratory conditions, affecting 20–50% of patients admitted with pneumonia, asthma, bronchiolitis, or COVID-19. However, hypoxaemia was also common in other severe systemic illnesses, affecting 10–25% of patients admitted with sepsis, HIV-related complications, and meningoencephalitis, and 7–15% of patients admitted with malaria, malnutrition, and trauma. Hypoxaemia prevalence in patients admitted to secondary health facilities (general hospitals) was slightly lower than those admitted to tertiary facilities (referral hospitals). Hypoxaemia was more common among populations living at high altitude, in keeping with known lower blood oxygen ranges in healthy people living at high altitude.^27^ However, scarcity of data from high-altitude settings means our hypoxaemia prevalence estimates primarily represent low-altitude settings.

Among admitted neonates, our overall meta-estimate for hypoxaemia prevalence was slightly higher than reported in the 2009 review (24·5%, 95% CI 19·9–29·4, *vs* median 19·2%, IQR 18·2–21·4).^16^ With an estimated 35·3 million neonates born preterm or with low birthweight annually,^28^ our estimates suggest that more than 8·6 million of these neonates have hypoxaemia requiring oxygen or other respiratory support. We found that hypoxaemia was highly prevalent for the three commonest causes of neonatal illness and death, affecting a third of hospitalised neonates with prematurity, neonatal encephalopathy (often called birth asphyxia), and sepsis. With 2·3 million neonatal deaths annually, improving access to safe oxygen and respiratory care is essential for LMICs to achieve the Sustainable Development Goals target for neonatal mortality.^29^

Among admitted children, our meta-estimate for hypoxaemia prevalence for children with pneumonia (34·6%, 95% CI 30·2–39·1) was higher than the 2009 review (median 13·3%, IQR 9·3–37·5),^16^ but similar to the more recent 2022 review (31%, 95% CI 26–36).^17^ This supports the 2022 review estimates that about 7·2 million children younger than 5 years with pneumonia admitted to facilities in LMICs annually had hypoxaemia requiring oxygen or respiratory support in 2021.^17^, ^30^ Importantly, this estimate did not include children aged 5 years or older and adolescents with pneumonia for whom hypoxaemia is similarly common, or hypoxaemic children who do not reach a facility.

Data from our review suggested that about half of the hypoxaemia burden among children was for non-pneumonia conditions. However, these studies might over-represent data from settings with a high pneumonia burden, thereby overestimating the contribution of pneumonia to overall hypoxaemia prevalence. Although the broad WHO pneumonia definition used in many studies captures other respiratory conditions, such as bronchiolitis, pulmonary tuberculosis, HIV-related opportunistic chest infections, COVID-19, and possibly asthma,^31^ our hypoxaemia prevalence estimates for these were remarkably similar to estimates for children with pneumonia. Compared with the 2009 review, we found slightly higher hypoxaemia prevalence for malaria (6·4%, 95% CI 4·0–9·3, *vs* median 4·1%, range 3·3−17·1), and meningitis or encephalitis (13·7%, 5·1–25·3, *vs* median 9·5%, range 2·7−14·6), and diarrhoeal disease (4·7%, 1·7–9·0, *vs* median 2·3%, range 0·0−4·7),^16^ and higher prevalence for children with malnutrition (15·3%, 8·6–23·4, *vs* median 4·6%, range 1·8−8·3) who often have comorbidity and very high risk of death. Given the high burden of malaria and malnutrition in low-income and lower-middle-income countries in sub-Saharan Africa and Asia, our data suggest an under-recognised need for oxygen in these populations.

Among admitted adults, we found that overall hypoxaemia prevalence was similar to admitted children (10·8% *vs* 12·1%). In contrast to child populations, hypoxaemia was more common among admitted adults with COVID-19 than those with pneumonia more broadly (44·2% *vs* 20·4%), although this finding might be related to pandemic-related changes in admission and care criteria.^32^ Acute respiratory conditions and sepsis are leading causes of adult hypoxaemia, morbidity, and mortality,^33^ and we would expect large numbers of adults to need oxygen and critical care services globally.^34^ Our study was unable to provide meta-estimates of hypoxaemia prevalence for adults with other conditions needing oxygen and critical care services, such as trauma, maternity care, or acute on chronic cardiorespiratory compromise (eg, chronic obstructive pulmonary disease exacerbation and heart failure), due to scarcity of data.

Global guidelines recognise hypoxaemia detection and treatment as an essential component of emergency care for acutely unwell children and adults.^35^, ^36^ However, basic capacity for hypoxaemia detection with pulse oximetry and provision of simple oxygen therapy remain insufficient.^37^ The COVID-19 pandemic provided added urgency, revealing a particular need for hypoxaemia detection and essential treatments, and exposing gross weaknesses in current oxygen and critical care services.^38^, ^39^

Our findings support the need for an integrated approach to the care of critically unwell patients, with hypoxaemic respiratory failure being a common and life-threatening complication affecting patients with a wide range of conditions across all ages. From an advocacy and strategy perspective, hypoxaemia detection (with pulse oximetry) and management (oxygen-related services) must permeate agendas beyond child pneumonia and COVID-19 to be embraced by those working in universal health coverage, quality of care, maternal care, neonatal care, HIV, malaria, tuberculosis, surgical and anaesthetic care, and pandemic and emergency response. From a programme and implementation perspective, we must seek to improve and integrate essential emergency and critical care services across the health system,^38^ with hypoxaemia detection and early treatment being a sentinel indicator of broader hospital quality of care.

From a clinical perspective, our study shows a high burden of hypoxaemia across ages, geographies, and conditions. Much of this burden will remain unrecognised and contribute to excess mortality until pulse oximetry becomes a routine part of triage, assessment, and care for all acutely unwell patients globally.^40^ Although pulse oximeters are increasingly affordable and easy to use, challenges remain in adoption and effective implementation,^41^ particularly in poor and rural settings. Oximeter quality is highly variable, and independent testing has shown that many low-cost oximeters fail basic performance standards, even if they have regulatory approvals.^42^, ^43^ Recent device development and implementation efforts have produced more fit-for-purpose affordable oximeters that cost about US$250,^44^, ^45^, ^46^ but these compete with many cheaper fingertip oximeters with unclear clinical utility or accuracy and potentially higher lifetime cost.^43^ Concerningly, numerous studies have reported that oximeters systematically overestimate oxygen saturation in people with deeply pigmented skin (so-called skin tone bias), thereby missing individuals with clinically relevant hypoxaemia.^47^ This should not detract from efforts to increase pulse oximetry adoption but is a clear priority area for improved regulation and device performance.^47^ Moreover, oximetry is still absent from many global clinical guidelines, education curricula, and health facility standards. A positive outlier here is pulse oximetry in surgical, anaesthetic, and critical care, which has been a core focus for the Safe Surgery movement since 2008.^44^ Improving pulse oximetry use in facilities requires clear normative guidance, well supported champions, adequate and reliable devices, and clinical supervision to help health-care workers appreciate the added value of pulse oximetry in their practice.^48^

The strong association between hypoxaemia and mortality underlies the value of pulse oximetry in risk assessment and stratification,^49^, ^50^ in addition to its essential role in guiding oxygen treatment decisions. This is highly relevant for emergency and critical care triage and initial assessment,^35^, ^36^ and for identifying patients with hypoxaemia at lower-level facilities.^51^ Current WHO/UNICEF primary care guidelines provide limited guidance on when or how health-care workers should use pulse oximetry, with most patients with hypoxaemia presenting to primary care currently going unidentified.^52^ In these settings, pulse oximetry might be a useful tool to help identify patients requiring careful reassessment, consideration for referral, and closer follow-up.^7^

This study is a comprehensive synthesis of hypoxaemia data across all ages and various clinical conditions, representing populations in LMICs from every region of the world. Our broad inclusion criteria enabled us to capture data on age groups and conditions that had not been previously reported. We reviewed more than 9000 articles to identify 213 studies for inclusion in quantitative analysis involving 601 757 participants (compared with 57 studies and 101 775 child participants in the previous largest review).^17^ However, we were limited by the quality of source data. Few studies collected hypoxaemia prevalence as a core outcome, so the methodology and reporting quality of included studies varied. Included studies typically reported hypoxaemia based on assessment around the time of admission, and this might have led to underestimation of hypoxaemia prevalence over the entire admission period or before intervention. We focused on blood oxygen saturations obtained by pulse oximetry, as the gold-standard blood gas assessment using co-oximetry is typically only available in relatively well resourced intensive care settings and would not reflect general acutely unwell populations. However, pulse oximetry use is not routine in many smaller health facilities and poor communities, and our review had over-representation of research cohorts from larger facilities.^53^ Pulse oximeter accuracy varies between devices and is less accurate at lower oxygen saturations (<80%) and in low perfusion states,^42^ with additional measurement uncertainty stemming from patients and device users.^54^ Although these measurement uncertainties should not systematically bias our estimates, there is evidence that some oximeters overestimate blood oxygen saturation in patients with deeply pigmented skin, potentially underestimating hypoxaemia prevalence for these populations.^55^ Included studies used a range of SpO_2_ cutoff values to report hypoxaemia, and this prompted our sensitivity analysis focusing on studies with a cutoff value of less than 90%, which yielded similar findings. We believe this gives a reasonable conservative estimate of the proportion of people who would likely need oxygen, acknowledging that clinical decisions about oxygen should not be solely determined by a binary SpO_2_ cutoff value. We relied on recorded diagnostic classifications for categorising by condition, but these were often not standardised or inclusive of comorbidity.

Scarcity of high-quality data limited the certainty of estimates for many conditions, with particularly few data from adult populations. Included studies over-represented child populations and particular countries in Africa. To address these potential biases, we presented prespecified subgroup analysis by level of health facility, geographical region, and age.

In summary, hypoxaemia is a physiological marker of respiratory failure that is common among acutely unwell patients of all ages presenting to health facilities, small and large, and strongly associated with death. Recognising this sizable population in need of medical oxygen services, we urge continued effort to strengthen medical oxygen services and the broader assessment and referral pathways on which patients depend. Hypoxaemia detection with pulse oximetry and oxygen-related treatment are essential components of care for patients and should be integrated into clinical guidelines, service structures, and strategies for maternal, neonatal, child, adolescent, and adult health sectors.

### Contributors

### Data sharing

All relevant data are reported in the manuscript and appendix.

## Declaration of interests

HRG received general salary and grant support for child health research from the Royal Children's Hospital Foundation (Melbourne, VIC, Australia), the Australian National Health and Medical Research Council (ID 2009026), the Bill & Melinda Gates Foundation (INV 043011), and the Swedish Research Council, and has served as a trustee and adviser on oxygen-related projects with the Oxygen for Life Foundation, Lifebox Foundation, Unitaid, and Asian Development Bank. RS received general salary and grant support for child health research from the Murdoch Children's Research Institute and the Royal Australasian College of Physicians scholarship. AER received general salary and grant support for child health research from icddr,b, Global Affairs Canada, the US Agency for International Development (USAID), the UK Foreign, Commonwealth and Development Office, the Government of Bangladesh, WHO, UN Population Fund, the University of Edinburgh (Edinburgh, UK), the University of North Carolina (Chapel Hill, NC, USA), the University of Sheffield (Sheffield, UK), and Unitaid. FL and JLM worked for the Clinton Health Access Initiative, which has received grants related to oxygen service delivery from the Gates Foundation (INV 043011), ELMA Foundation (22-F0021), Unitaid, USAID, FHI 360, and GiveWell. All other authors declare no competing interests.
