# Supplementary material for: The prevalence of hypoxaemia in paediatric and adult patients in health-care facilities in low-income and middle-income countries: a systematic review and meta-analysis
Source: Lancet Glob Health. 2025 Jan 29;13(2):e222–31. doi: 10.1016/S2214-109X(24)00469-8 (PMC11783038; doi:10.1016/S2214-109X(24)00469-8)
Supplement: Supplementary appendix [file mmc1.pdf]

THE LANCET
Global Health

Supplementary appendix

This appendix formed part of the original submission and has been peer reviewed. We post it as supplied by the authors.

Supplement to: Graham HR, Jahan E, Subhi R, et al. The prevalence of hypoxaemia in paediatric and adult patients in health-care facilities in low-income and middle-income countries: a systematic review and meta-analysis. *Lancet Glob Health* 2025; **13:** e222–31.

Supplemental material

PAPER: The prevalence of hypoxaemia among paediatric and adult patients in healthcare facilities in low- and middle-income countries: systematic review and meta-analysis

AUTHORS: Hamish R Graham, Rami Subhi, Esrat Jahan, Farhia Azrin, Jaclyn Maher, Jasmine Miller, Ahmed Ehsanur Rahman, Felix Lam.

Contents

- Text S1: Search strategy including full search terms 7
  - Ovid MEDLINE 7
  - Embase 9
  - Pubmed 11
  - CINAHL 13
- Text S2: Full list of data items for extraction 15
- Table S1: Full results table mapping 17
- Table S2: Full results table mapping – sensitivity analysis 28
- Table S3: Hypoxaemia burden in children: pneumonia versus overall 34
- Figure S1: Secondary versus Tertiary facilities 35
- Figure S2: Secondary versus Tertiary facilities – sensitivity analysis 36
- Figure S3: Hypoxaemia prevalence – sensitivity analysis 37
- Figure S3: Altitude scatterplot – sensitivity analysis 38
- Figure S3: Relative odds of death – sensitivity analysis 40
- Table S4: Risk of bias assessments 41
- Text S3: Assessment of Certainty 67
- Table S5: Full list of included studies 69
- Table S6: Full list of excluded studies with reason for exclusion 98

Primary Results tables and Forest plots (Admitted) 134

- Admitted Neonates - all 134
- Admitted Neonates – neonatal encephalopathy / birth asphyxia 136
- Admitted Neonates - pneumonia 137
- Admitted Neonates - prematurity 138
- Admitted Neonates - sepsis 139
- Admitted Children - all 140
- Admitted Children - fever 142
- Admitted Children – pneumonia all WHO-classification 143

Admitted Children – pneumonia severe (WHO-classification, severe or very severe) 147
Admitted Children – pneumonia non-severe (WHO-classification) 150
Admitted Children – pneumonia radiological 151
Admitted Children - bronchiolitis 153
Admitted Children – pneumonia unspecified 154
Admitted Children - anaemia 155
Admitted Children - asthma 156
Admitted Children – HIV complication 157
Admitted Children - malaria 158
Admitted Children – meningitis / encephalitis 160
Admitted Children - seizures 161
Admitted Children – sepsis 162
Admitted Children – trauma / injury 163
Admitted Children - Tuberculosis 164
Admitted Children – COVID-19 165
Admitted Children - diarrhoea 167
Admitted Children - malnutrition 168
Admitted Adults – all 171
Admitted Adults - fever 173
Admitted Adults - pneumonia 174
Admitted Adults - asthma 176
Admitted Adults – HIV complications 177
Admitted Adults - seizures 178
Admitted Adults - sepsis 179
Admitted Adults – trauma / injury 180
Admitted Adults - tuberculosis 181
Admitted Adults – COVID-19 182
Primary Results tables and Forest plots (OPD/ED) 185
OPD/ED Neonates - all 185
OPD/ED Neonates - pneumonia 186
OPD/ED Children - all 187
OPD/ED Children - fever 188
OPD/ED Children – pneumonia all WHO-classified 189
OPD/ED Children – pneumonia severe (WHO-classified severe or very severe) 191
OPD/ED Children – pneumonia non-severe (WHO-classified) 192
OPD/ED Children – pneumonia radiological 193

OPD/ED Children - bronchiolitis 194
OPD/ED Children – pneumonia unspecified 195
OPD/ED Children - anaemia 196
OPD/ED Children - asthma 197
OPD/ED Children - malaria 198
OPD/ED Children - sepsis 199
OPD/ED Children - diarrhoea 200
OPD/ED Adults - all 201
OPD/ED Adults - pneumonia 202
OPD/ED Adults – pneumonia radiological 203
OPD/ED Adults – HIV complications 204
OPD/ED Adults – malaria 205
OPD/ED Adults – sepsis 206
OPD/ED Adults – trauma / injury 207
Subgroup Results tables and Forest plots (Tertiary vs Secondary level facilities) 208
Tertiary Admitted Neonates 208
Tertiary Admitted Children 210
Tertiary Admitted Adults 211
Tertiary Admitted Children – pneumonia all WHO-classified 212
Tertiary Admitted Children – malaria 214
Tertiary Admitted Children – sepsis 215
Tertiary Admitted Children – malnutrition 216
Secondary Admitted Neonates 218
Secondary Admitted Children 219
Secondary Admitted Adults 221
Secondary Admitted Children – pneumonia all WHO-classification 222
Secondary Admitted Children – malaria 224
Secondary Admitted Children – sepsis 226
Secondary Admitted Children – malnutrition 227
Subgroup Results tables and Forest plots (Altitude and Region) 228
High versus Low-altitude Admitted Children with pneumonia (WHO-classified pneumonia, severe, very severe) 228
Regional breakdown Admitted Children with pneumonia (WHO-classified pneumonia, severe, very severe) 232
Secondary Results tables and Forest plots (Relative Odds of Death) 235
Relative Odds of Death – neonates 235

Relative Odds of Death – neonates with non-primary respiratory disease 236
Relative Odds of Death - children 237
Relative Odds of Death – children with primary respiratory disease 240
Relative Odds of Death – children with non-primary respiratory disease 242
Relative Odds of Death – adults 243
Relative Odds of Death – adults with primary respiratory disease 246
Relative Odds of Death – adults with non-primary respiratory disease 248
Relative Odds of Death – overall (neonates, children, and adults) 249
Sensitivity Analysis (SpO2<90%) Results tables and Forest plots 253
Admitted Neonates - all 253
Admitted Neonates – neonatal encephalopathy / birth asphyxia 254
Admitted Neonates – pneumonia 256
Admitted Neonates - prematurity 257
Admitted Neonates - sepsis 258
Admitted Children – all 259
Admitted Children - fever 261
Admitted Children – pneumonia all WHO-classified 262
Admitted Children – pneumonia severe (WHO classification severe or very severe) 265
Admitted Children – pneumonia non-severe (WHO classification) 267
Admitted Children – pneumonia radiological 268
Admitted Children - bronchiolitis 270
Admitted Children – pneumonia unspecified 271
Admitted Children - anaemia 272
Admitted Children – asthma 273
Admitted Children – HIV complications 274
Admitted Children – malaria 275
Admitted Children – meningitis 277
Admitted Children – seizures 279
Admitted Children – sepsis 280
Admitted Children – trauma / injury 282
Admitted Children – tuberculosis 283
Admitted Children – COVID-19 284
Admitted Children – diarrhoea 285
Admitted Children – malnutrition 287
Admitted Adults – all 288
Admitted Adults - fever 290

Admitted Adults – pneumonia 291
Admitted Adults – asthma 292
Admitted Adults – HIV complications 293
Admitted Adults – seizures 294
Admitted Adults – sepsis 295
Admitted Adults – trauma / injury 297
Admitted Adults – COVID-19 298
OPD/ED Neonates – all 299
OPD/ED Neonates – pneumonia 300
OPD/ED Children – all 301
OPD/ED Children - fever 302
OPD/ED Children – pneumonia all WHO-classified 303
OPD/ED Children – pneumonia severe (WHO classification) 305
OPD/ED Children – pneumonia non-severe (WHO classification) 306
OPD/ED Children – pneumonia radiological 307
OPD/ED Children – bronchiolitis 308
OPD/ED Children – pneumonia unspecified 309
OPD/ED Children – anaemia 310
OPD/ED Children – asthma 311
OPD/ED Children – malaria 312
OPD/ED Children – sepsis 313
OPD/ED Children – diarrhoea 314
OPD/ED Adults – all 315
OPD/ED Adults – pneumonia 316
OPD/ED Adults – pneumonia radiological 317
OPD/ED Adults – HIV complications 318
OPD/ED Adults – malaria 319
OPD/ED Adults – sepsis 320
OPD/ED Adults – trauma / injury 321
Tertiary Admitted Neonates 322
Tertiary Admitted Children 324
Tertiary Admitted Adults 325
Tertiary Admitted Children – pneumonia all WHO-classified 326
Tertiary Admitted Children – malaria 328
Tertiary Admitted Children – sepsis 329
Tertiary Admitted Children – malnutrition 330

Secondary Admitted Neonates 331
Secondary Admitted Children 332
Secondary Admitted Adults 333
Secondary Admitted Children – pneumonia all WHO-classified.. 334
Secondary Admitted Children – malaria 336
Secondary Admitted Children – sepsis 337
Secondary Admitted Children – malnutrition 338
Relative Odds of Death - neonates.. 339
Relative Odds of Death – children.. 340
Relative Odds of Death – children with primary respiratory disease 343
Relative Odds of Death – children with non-primary respiratory disease 345
Relative Odds of Death - adults 346
Relative Odds of Death – adults with primary respiratory disease 348
Relative Odds of Death – adults with non-primary respiratory disease 349
Relative Odds of Death – overall (neonates, children, and adults) 350
High versus Low-altitude Admitted Children with pneumonia (WHO-classified pneumonia, severe, very severe) 353
Regional breakdown Admitted Children with pneumonia (WHO-classified pneumonia, severe, very severe) 357
References 360

Text S1: Search strategy including full search terms

The search strategy was developed with support from a research librarian (Royal Children's Hospital, University of Melbourne Department of Paediatrics). We provided a sample set of publications that included populations of different age groups, geographies, and regions. These were obtained from previous systematic reviews and an additional directed literature search. We used a bespoke filter created by the research librarian to capture papers from low- and middle-income countries. To maximise sensitivity we searched for major acute conditions that contribute to global mortality and specific conditions that are indicative of severe disease (e.g. meningitis/encephalitis). The full search terms for the respective databases are listed below.

Ovid MEDLINE

1. hypoxia/

2. hypoxia/di

3. (anox?emia or hypox?emia).tw,kf.

4. exp Respiratory Tract Infections/cl, co, mo

5. pneumonia/cl, co, mo or bronchopneumonia/cl, co, mo or pleuropneumonia/cl, co, mo or exp pneumonia, bacterial/cl, co, mo or pneumocystis/cl or pneumonia, viral/cl, co, mo

6. exp Malaria/cl, co, mo [Classification, Complications, Mortality]

7. exp Tuberculosis/cl, co, mo [Classification, Complications, Mortality]

8. exp Pulmonary Disease, Chronic Obstructive/cl, co, mo [Classification, Complications, Mortality]

9. exp Asthma/cl, co, mo [Classification, Complications, Mortality]

10. exp Sepsis/cl, co, mo [Classification, Complications, Mortality]

11. exp Meningitis/cl, co, mo [Classification, Complications, Mortality]

12. exp Brain Diseases/cl, co, mo

13. exp Multiple Trauma/cl, co, mo [Classification, Complications, Mortality]

14. exp Pregnancy Complications/cl, co, mo

15. exp neoplasms/cl, co, mo

16. exp Oximetry/

17. Oxygen/ad, bl, tu, th [Administration & Dosage, Blood, Therapeutic Use, Therapy]

18. (oximetry or oxygen-saturation).tw,kf.

19. nutrition disorders/cl, co, mo or malnutrition/cl, co, mo or exp severe acute malnutrition/co, mo or starvation/cl, co, mo

20. risk factors/

21. "reproducibility of results"/ or "sensitivity and specificity"/ or "predictive value of tests"/ or roc curve/ or Mobile Applications/ or exp Point-of-Care Systems/

22. incidence/ or prevalence/

23. developing countries/

24. (austere or (limited adj2 resource*) or (low adj2 resource*) or (transitioning adj econom*) or (third adj world) or LMIC or LMICs or (lami adj countr*) or (transitional adj countr*) or (low adj gdp) or (low adj gnp) or (low adj gross adj domestic) or (low adj gross adj national) or ((emerging or developing or (low adj income) or (middle adj income) or (low adj3 middle) or underdeveloped or under-developed or (less* adj developed) or underserved or under-served or deprived or poor*) and (countr* or nation*1 or econom* or population or world))).tw,kf.

25. exp africa/

26. americas/ or exp caribbean region/ or exp central america/ or latin america/ or mexico/ or exp south america/

27. europe/ or exp europe, eastern/ or exp transcaucasia/

28. antarctic regions/ or exp atlantic islands/ or exp indian ocean islands/ or exp pacific islands/

29. New Guinea/

30. asia/ or exp asia, central/ or asia, southeastern/ or borneo/ or cambodia/ or east timor/ or indonesia/ or laos/ or malaysia/ or mekong valley/ or myanmar/ or philippines/ or thailand/ or vietnam/ or asia, western/ or bangladesh/ or bhutan/ or india/ or middle east/ or afghanistan/ or iran/ or iraq/ or jordan/ or lebanon/ or oman/ or saudi arabia/ or syria/ or turkey/ or yemen/ or nepal/ or pakistan/ or sri lanka/ or far east/ or china/ or tibet/ or exp korea/ or mongolia/

31. (Afghanistan or Albania or Algeria or Angola or Antigua or Argentina or Armenia* or Aruba or Azerbaijan or Bahrain or Bangladesh or Barbados or Barbuda or Belarus or Byelarus* or Byelorussian or Belorussian or Belorus* or Belize or Benin or Bhutan or Bolivia or Bosnia or Botswana or Brasil or Brazil or Bulgaria or (Burkina adj Fas*) or (Upper adj Volta) or Burma or Burundi or Cambodia or Khmer or Kampuchea or Cameron* or Cameroon* or (Cape adj Verde) or (Cabo adj Verde) or (Central adj African adj Republic) or Chad or Chile or China or Colombia or Comoros or (Comoro adj Island*) or Comores or Mayotte or Congo or Kongo or (Cook adj Island*) or (Costa adj Rica) or (Cote adj D'ivoire) or Croatia or Cuba or Cyprus or (Czech adj Republic) or Czechoslovakia or Djibouti or Dominica or Dominican or (East adj Timor) or (East adj Timur) or Ecuador or Egypt or El-Salvador or (Equatorial adj Guinea) or Eritrea or Estonia or Ethiopia or Fiji or (French adj Somaliland) or Futuna or Gabon or (Gabonese adj Republic) or Gambia or Gaza or (Georgia* adj Republic) or Ghana or Grenada or Guam or Guatemala or Guinea or Guiana or Guyana or Haiti or Herzeg* or Hercegovina or Honduras or Hungary or India or Indonesia or Iran or Iraq or (Ivory adj Coast) or Jamaica or Jordan or Kazakh* or Kenya or Kiribati or Korea or Kosovo or (Kyrgyz adj Republic) or Kyrgyzstan or Kirghizia or Kirghiz or Kirgizstan or Laos or (Lao* adj2 Democratic adj Republic) or (Lao* adj PDR) or Latvia or Lebanon or Lesotho or Basutoland or Liberia or Libya or Lithuania or Macedonia or Madagascar or (Magalasy adj Republic) or Malawi or Malay* or Sabah or Sarawak or Maldives or Mali or (Marshall adj Island*) or Mauritania or Mauritius or (Agalega adj Island*) or Mexico or Micronesia or Moldov* or Mongolia or Montserrat or Montenegro or Morocco or Ifni or Mozambique or Myanma* or Namibia or Nauru or Nepal or (Netherlands adj Antilles) or (Dutch adj Antilles) or (New adj Guinea) or (New adj Caledonia) or Nicaragua or Niue or Niger or Nigeria or (Northern adj Mariana adj Island*) or Nyasaland or Oman or Pakistan or Palau or Panama or (Papua adj New adj Guinea) or PNG or Palestine or Paraguay or Peru or Philipines or Philippines or Phillipines or Phillippines or Poland or (Puerto adj Rico) or Yemen or Romania or Roumania or Rumania or Russia* or Rwanda or Ruanda or (Saint adj Kitts) or (St adj Kitts) or Nevis or (Saint adj Vincent) or (St adj Vincent) or Grenadines or Samoa* or (Navigator adj Island*) or (Saint adj Lucia) or (St adj Lucia) or (Saint adj Helena) or (St adj Helena) or (Sao adj Tome) or (Saudi adj Arabia) or Senegal or Serbia or Seychelles or (Sierra adj Leone) or Slovenia or Slovak* or (South adj Africa) or (Solomon adj Island*) or Somalia or (Sri adj Lanka) or Ceylon or Sudan or Surinam* or Swaziland or Syria or Tajikistan or Tadzhikistan or Tadjikistan or Tadzhik or Tanzania or Thailand or Tibet or Timor-Leste or Togo or (Togolese adj Republic) or Tokelau or Tonga or Trinidad or Tobago or Tunisia or Turkey or Turkmenistan or Turkmen or Tuvalu or Uganda or Ukraine or Uruguay or Urundi or USSR or (Soviet adj Union) or "Union of Soviet Socialist Republics" or Uzbekistan or Vanuatu or (New adj Hebrides) or

Venezuela or Vietnam or (Viet adj Nam) or (Wallis adj Futuna) or (United adj Arab adj Republic) or (West adj Bank) or (West adj Indies) or Yemen or Yugoslavia or Zaire or Zambia or Zimbabwe or Rhodesia).tw,kf.

32. (africa or americas or caribbean or (central adj America) or (latin adj America) or (south adj America) or (eastern adj Europe) or Transcaucasia or antarctic or (atlantic adj island*) or (indian adj ocean adj island*) or (pacific adj island*) or polynesia or (central adj asia) or (southeast* adj asia) or (south-east* adj asia) or borneo or mekong or (western adj asia) or (middle adj east) or (far adj east)).tw,kf.

33. 23 or 24 or 25 or 26 or 27 or 28 or 29 or 30 or 31 or 32

34. (1 or 3 or 16 or 17 or 18) and (4 or 5 or 6 or 7 or 8 or 9 or 10 or 11 or 12 or 13 or 14 or 15 or 19) and 33

35. (1 or 3 or 16 or 17 or 18) and 20 and 33

36. (2 or exp *Respiratory Tract Infections/cl, co, mo or (*pneumonia/cl, co, mo or *bronchopneumonia/cl, co, mo or *pleuropneumonia/cl, co, mo or exp *pneumonia, bacterial/cl, co, mo or *pneumocystis/cl or *pneumonia, viral/cl, co, mo) or exp *Malaria/cl, co, mo or exp *Tuberculosis/cl, co, mo or exp *Pulmonary Disease, Chronic Obstructive/cl, co, mo or exp *Asthma/cl, co, mo or exp *Sepsis/cl, co, mo or exp *Meningitis/cl, co, mo or exp *Brain Diseases/cl, co, mo or exp *Multiple Trauma/cl, co, mo or exp *Pregnancy Complications/cl, co, mo or exp *neoplasms/cl, co, mo or (*nutrition disorders/cl, co, mo or *malnutrition/cl, co, mo or exp *severe acute malnutrition/co, mo or *starvation/cl, co, mo)) and 21 and 33

37. *hypoxia/ and 22

38. (1 or 3) and (16 or 17 or 18 or 21) and 33

39. 34 or 35 or 36 or 37 or 38

40. exp animals/ not human*.sh.

41. 39 not 40

42. limit 41 to yr="1998 -Current"

Embase

1. *hypoxia/

2. (anox?emia or hypoxemia).tw,kw,dq,hw.

3. exp respiratory tract infection/co, di, et

4. exp bacterial pneumonia/di, ep, et or exp infectious pneumonia/di, ep, et

5. exp malaria/co, di, dm, th [Complication, Diagnosis, Disease Management, Therapy]

6. tuberculosis/co, di, dm, th [Complication, Diagnosis, Disease Management, Therapy]

7. chronic obstructive lung disease/co, di, dm, th [Complication, Diagnosis, Disease Management, Therapy]

8. exp asthma/co, di, dm, th [Complication, Diagnosis, Disease Management, Therapy]

9. exp sepsis/co, di, dm, th [Complication, Diagnosis, Disease Management, Therapy]

10. exp meningitis/co, di, dm, th [Complication, Diagnosis, Disease Management, Therapy]

11. brain disease/co, di, dm, th [Complication, Diagnosis, Disease Management, Therapy]

12. exp multiple trauma/co, di, dm, th [Complication, Diagnosis, Disease Management, Therapy]

13. pregnancy complication/co, di, dm, th [Complication, Diagnosis, Disease Management, Therapy]

14. exp neoplasm/co, di, dm, th [Complication, Diagnosis, Disease Management, Therapy]

15. exp pulse oximetry/ or exp oximetry/

16. exp oxygen/ad, dt [Drug Administration, Drug Therapy]

17. exp oxygen blood level/

18. exp nutritional disorder/co, di, dm, th [Complication, Diagnosis, Disease Management, Therapy]

19. exp risk factor/

20. exp reproducibility/ or exp "sensitivity and specificity"/ or predictive value/ or receiver operating characteristic/ or exp mobile application/ or "point of care system"/

21. exp incidence/ or exp prevalence/

22. developing country/

23. (austere or (limited adj2 resource*) or (low adj2 resource*) or (transitioning adj econom*) or (third adj world) or LMIC or LMICs or (lami adj countr*) or (transitional adj countr*) or (low adj gdp) or (low adj gnp) or (low adj gross adj domestic) or (low adj gross adj national) or ((emerging or developing or (low adj income) or (middle adj income) or (low adj3 middle) or underdeveloped or under-developed or (less* adj developed) or underserved or under-served or deprived or poor*) and (countr* or nation*1 or econom* or population or world))).tw,kw.

24. exp Africa/

25. exp "south and central america"/ or mexico/

26. asia/ or far east/ or china/ or exp korea/ or mongolia/ or philippines/ or kazakhstan/ or kyrgyzstan/ or tajikistan/ or turkmenistan/ or uzbekistan/ or middle east/ or iran/ or iraq/ or jordan/ or lebanon/ or oman/ or palestine/ or saudi arabia/ or syrian arab republic/ or "turkey (republic)"/ or yemen/ or southeast asia/ or borneo/ or cambodia/ or indonesia/ or laos/ or malaysia/ or myanmar/ or papua new guinea/ or thailand/ or timor-leste/ or viet nam/ or exp south asia/

27. exp "arctic and antarctic"/

28. atlantic islands/ or bermuda/ or bouvet island/ or exp caribbean islands/ or "falkland islands (malvinas)"/ or saint helena/ or "saint pierre and miquelon"/ or "sao tome and principe"/ or "south georgia and the south sandwich islands"/ or exp indian ocean/ or exp pacific ocean/

29. (Afghanistan or Albania or Algeria or Angola or Antigua or Argentina or Armenia* or Aruba or Azerbaijan or Bahrain or Bangladesh or Barbados or Barbuda or Belarus or Byelarus* or Byelorussian or Belorussian or Belorus* or Belize or Benin or Bhutan or Bolivia or Bosnia or Botswana or Brasil or Brazil or Bulgaria or (Burkina adj Fas*) or (Upper adj Volta) or Burma or Burundi or Cambodia or Khmer or Kampuchea or Cameron* or Cameroon* or (Cape adj Verde) or (Cabo adj Verde) or (Central adj African adj Republic) or Chad or Chile or China or Colombia or Comoros or (Comoro adj Island*) or Comores or Mayotte or Congo or Kongo or (Cook adj Island*) or (Costa adj Rica) or (Cote adj D'ivoire) or Croatia or Cuba or Cyprus or (Czech adj Republic) or Czechoslovakia or Djibouti or Dominica or Dominican or (East adj Timor) or (East adj Timur) or Ecuador or Egypt or El-Salvador or (Equatorial adj Guinea) or Eritrea or Estonia or Ethiopia or Fiji or (French adj Somaliland) or Futuna or Gabon or (Gabonese adj Republic) or Gambia or Gaza or (Georgia* adj Republic) or Ghana or Grenada or Guam or Guatemala or Guinea or Guiana or Guyana or Haiti or Herzeg* or Hercegovina or Honduras or Hungary or India or Indonesia or Iran or Iraq or (Ivory adj

Coast) or Jamaica or Jordan or Kazakh* or Kenya or Kiribati or Korea or Kosovo or (Kyrgyz adj Republic) or Kyrgyzstan or Kirghizia or Kirghiz or Kirgizstan or Laos or (Lao* adj2 Democratic adj Republic) or (Lao* adj PDR) or Latvia or Lebanon or Lesotho or Basutoland or Liberia or Libya or Lithuania or Macedonia or Madagascar or (Magalasy adj Republic) or Malawi or Malay* or Sabah or Sarawak or Maldives or Mali or (Marshall adj Island*) or Mauritania or Mauritius or (Agalega adj Island*) or Mexico or Micronesia or Moldov* or Mongolia or Montserrat or Montenegro or Morocco or Ifni or Mozambique or Myanma* or Namibia or Nauru or Nepal or (Netherlands adj Antilles) or (Dutch adj Antilles) or (New adj Guinea) or (New adj Caledonia) or Nicaragua or Niue or Niger or Nigeria or (Northern adj Mariana adj Island*) or Nyasaland or Oman or Pakistan or Palau or Panama or (Papua adj New adj Guinea) or PNG or Palestine or Paraguay or Peru or Philipines or Philippines or Phillipines or Phillippines or Poland or (Puerto adj Rico) or Yemen or Romania or Roumania or Rumania or Russia* or Rwanda or Ruanda or (Saint adj Kitts) or (St adj Kitts) or Nevis or (Saint adj Vincent) or (St adj Vincent) or Grenadines or Samoa* or (Navigator adj Island*) or (Saint adj Lucia) or (St adj Lucia) or (Saint adj Helena) or (St adj Helena) or (Sao adj Tome) or (Saudi adj Arabia) or Senegal or Serbia or Seychelles or (Sierra adj Leone) or Slovenia or Slovak* or (South adj Africa) or (Solomon adj Island*) or Somalia or (Sri adj Lanka) or Ceylon or Sudan or Surinam* or Swaziland or Syria or Tajikistan or Tadzhikistan or Tadjikistan or Tadzhik or Tanzania or Thailand or Tibet or Timor-Leste or Togo or (Togolese adj Republic) or Tokelau or Tonga or Trinidad or Tobago or Tunisia or Turkey or Turkmenistan or Turkmen or Tuvalu or Uganda or Ukraine or Uruguay or Urundi or USSR or (Soviet adj Union) or "Union of Soviet Socialist Republics" or Uzbekistan or Vanuatu or (New adj Hebrides) or Venezuela or Vietnam or (Viet adj Nam) or (Wallis adj Futuna) or (United adj Arab adj Republic) or (West adj Bank) or (West adj Indies) or Yemen or Yugoslavia or Zaire or Zambia or Zimbabwe or Rhodesia).tw,kw.

30. (africa or americas or caribbean or (central adj America) or (latin adj America) or (south adj America) or (eastern adj Europe) or Transcaucasia or antarctic or (atlantic adj island*) or (indian adj ocean adj island*) or (pacific adj island*) or polynesia or (central adj asia) or (southeast* adj asia) or (south-east* adj asia) or borneo or mekong or (western adj asia) or (middle adj east) or (far adj east)).tw,kw.

31. europe/ or exp eastern europe/

32. 22 or 23 or 24 or 25 or 26 or 27 or 28 or 29 or 30 or 31

33. (1 or 2 or 15 or 16 or 17) and (3 or 4 or 5 or 6 or 7 or 8 or 9 or 10 or 11 or 12 or 13 or 14 or 18) and 32

34. (1 or 2 or 15 or 16 or 17) and 19 and 32

35. 1 and 21

36. (1 or 2) and (15 or 16 or 17 or 20) and 32

37. 33 or 34 or 35 or 36

38. exp animal/ not human*.sh.

39. 37 not 38

40. limit 39 to yr="1998 -Current"

Pubmed

((((Hypoxia[Title/Abstract] OR hypoxic[Title/Abstract] OR hypoxaemia[Title/Abstract] OR hypoxemic[Title/Abstract] OR anoxic[Title/Abstract] OR anoxaemia[Title/Abstract])) AND (Respiratory-tract-infection*[Title/Abstract] OR pneumonia[Title/Abstract] OR malaria[Title/Abstract] OR tuberculosis[Title/Abstract] OR COPD[Title/Abstract] OR chronic-obstructive-pulmonary-disease[Title/Abstract] OR asthma[Title/Abstract] OR sepsis[Title/Abstract] OR meningitis[Title/Abstract] OR brain-disease*[Title/Abstract] OR trauma[Title/Abstract] OR pregnancy[Title/Abstract] OR

neoplasm[Title/Abstract] OR neoplasms[Title/Abstract] OR risk-factor*[Title/Abstract])) AND ((austere OR limited resource* OR "resource limited" OR low resource* OR transitioning econom* OR lami countr* OR transitional countr* OR "low gdp" OR "low gnp" OR "low gross domestic" OR "low gross national" OR ((emerging OR developing OR "low income" OR "middle income" OR (low AND middle) OR underdeveloped OR "under developed" OR under-developed OR underserved OR "under served" OR under-served OR (less* AND developed) OR derived OR poor) AND (countr* OR nation* OR econom* OR population OR world)) OR "third world" OR lmic OR lmics) OR afghanistan OR albania OR algeria OR angola OR antigua OR argentina OR Armenia* OR aruba OR azerbaijan OR bahrain OR bangladesh OR barbados OR barbuda OR belarus OR Byelarus* OR byelorussian OR Belorus* OR belize OR benin OR bhutan OR bolivia OR bosnia OR botswana OR brasil OR brazil OR bulgaria OR burkina Fas* OR "Upper Volta" OR burma OR burundi OR cambodia OR khmer OR kampuchea OR Cameron* OR Cameroon* OR "Cape Verde" OR "Cabo Verde" OR "Central African Republic" OR chad OR chile OR china OR colombia OR comoros OR comoro Island* OR comores OR mayotte OR congo OR kongo OR cook Island* OR "Costa Rica" OR "Cote D'ivoire" OR croatia OR cuba OR cyprus OR "Czech Republic" OR czechoslovakia OR djibouti OR dominica OR dominican OR "East Timor" OR "East Timur" OR ecuador OR egypt OR el-salvador OR "Equatorial Guinea" OR eritrea OR estonia OR ethiopia OR fiji OR "French Somaliland" OR futuna OR gabon OR "Gabonese Republic" OR gambia OR gaza OR (Georgia* AND republic) OR ghana OR grenada OR guam OR guatemala OR guinea OR guiana OR guyana OR haiti OR Herzeg* OR hercegovina OR honduras OR hungary OR india OR indonesia OR iran OR iraq OR "Ivory Coast" OR jamaica OR jordan OR Kazakh* OR kenya OR kiribati OR korea OR kosovo OR "Kyrgyz Republic" OR kyrgyzstan OR kirghizia OR kirghiz OR kyrgyzstan OR Lao OR laos OR latvia OR lebanon OR lesotho OR basutoland OR liberia OR libya OR lithuania OR macedonia OR madagascar OR "Magalasy Republic" OR malawi OR Malay* OR sabah OR sarawak OR maldives OR mali OR marshall Island* OR mauritania OR mauritius OR galega Island* OR mexico OR micronesia OR Moldov* OR mongolia OR montserrat OR montenegro OR morocco OR ifni OR mozambique OR Myanma* OR namibia OR nauru OR nepal OR "Netherlands Antilles" OR "Dutch Antilles" OR "New Guinea" OR "New Caledonia" OR nicaragua OR niue OR niger OR nigeria OR ("Northern Mariana" AND Island*) OR nyasaland OR oman OR pakistan OR palau OR panama OR "Papua New Guinea" OR PNG OR palestine OR paraguay OR peru OR philippines OR philippines OR philippines OR philippines OR poland OR "Puerto Rico" OR yemen OR romania OR roumania OR rumania OR Russia* OR rwanda OR ruanda OR "Saint Kitts" OR "St Kitts" OR nevis OR "Saint Vincent" OR "St Vincent" OR grenadines OR Samoa* OR navigator Island* OR "Saint Lucia" OR "St Lucia" OR "Saint Helena" OR "St Helena" OR "Sao Tome" OR "Saudi Arabia" OR senegal OR serbia OR seychelles OR "Sierra Leone" OR slovenia OR Slovak* OR "South Africa" OR solomon Island* OR somalia OR "Sri Lanka" OR ceylon OR sudan OR Surinam* OR swaziland OR syria OR tajikistan OR tadzhikistan OR tadjikistan OR tadzhik OR tanzania OR thailand OR tibet OR timor-leste OR togo OR "Togolese Republic" OR tokelau OR tonga OR trinidad OR tobago OR tunisia OR turkey OR turkmenistan OR turkmen OR tuvalu OR uganda OR ukraine OR uruguay OR urundi OR ussr OR "Soviet Union" OR "Union of Soviet Socialist Republics" OR uzbekistan OR vanuatu OR "New Hebrides" OR venezuela OR vietnam OR "Viet Nam" OR "Wallis Futuna" OR "United Arab Republic" OR "West Bank" OR "West Indies" OR yemen OR yugoslavia OR zaire OR zambia OR zimbabwe OR rhodesia OR africa OR americas OR caribbean OR "central America" OR "latin America" OR "south America" OR "eastern Europe" OR transcaucasia OR antarctic OR atlantic island* OR "indian ocean" OR pacific island* OR polynesia OR "central asia" OR (southeast* AND asia) OR (south-east* AND asia) OR "south east asia" OR "south eastern asia" OR borneo OR mekong OR "western asia" OR "middle east" OR "far east") AND (NOTNLM OR publisher[sb] OR inprocess[sb] OR pubmednotmedline[sb] OR indatareview[sb] OR pubstatusaheadofprint)) OR ((((Hypoxia[Title/Abstract] OR hypoxic[Title/Abstract] OR hypoxaemia[Title/Abstract] OR hypoxemic[Title/Abstract] OR anoxic[Title/Abstract] OR anoxaemia[Title/Abstract])) AND (oximetry[Title/Abstract] OR oxygen-saturation[Title/Abstract] OR oxygen-blood-level[Title/Abstract] OR reproducibility[Title/Abstract] OR sensitivity[Title/Abstract] OR specificity[Title/Abstract] OR predictive-value[Title/Abstract] OR mobile-app*[Title/Abstract] OR point-of-care[Title/Abstract])) AND ((austere OR limited resource* OR "resource limited" OR low resource* OR transitioning econom* OR lami countr* OR transitional countr* OR "low gdp" OR "low gnp" OR "low gross domestic" OR "low gross national" OR ((emerging OR developing OR "low income" OR "middle income" OR (low AND middle) OR underdeveloped OR "under developed" OR under-developed OR underserved

OR "under served" OR under-served OR (less* AND developed) OR derived OR poor) AND (countr* OR nation* OR econom* OR population OR world)) OR "third world" OR lmic OR lmics) OR afghanistan OR albania OR algeria OR angola OR antigua OR argentina OR Armenia* OR aruba OR azerbaijan OR bahrain OR bangladesh OR barbados OR barbuda OR belarus OR Byelarus* OR byelorussian OR Belorus* OR belize OR benin OR bhutan OR bolivia OR bosnia OR botswana OR brasil OR brazil OR bulgaria OR burkina Fas* OR "Upper Volta" OR burma OR burundi OR cambodia OR khmer OR kampuchea OR Cameron* OR Cameroon* OR "Cape Verde" OR "Cabo Verde" OR "Central African Republic" OR chad OR chile OR china OR colombia OR comoros OR comoro Island* OR comores OR mayotte OR congo OR kongo OR cook Island* OR "Costa Rica" OR "Cote D'ivoire" OR croatia OR cuba OR cyprus OR "Czech Republic" OR czechoslovakia OR djibouti OR dominica OR dominican OR "East Timor" OR "East Timur" OR ecuador OR egypt OR el-salvador OR "Equatorial Guinea" OR eritrea OR estonia OR ethiopia OR fiji OR "French Somaliland" OR futuna OR gabon OR "Gabonese Republic" OR gambia OR gaza OR (Georgia* AND republic) OR ghana OR grenada OR guam OR guatemala OR guinea OR guiana OR guyana OR haiti OR Herzeg* OR hercegovina OR honduras OR hungary OR india OR indonesia OR iran OR iraq OR "Ivory Coast" OR jamaica OR jordan OR Kazakh* OR kenya OR kiribati OR korea OR kosovo OR "Kyrgyz Republic" OR kyrgyzstan OR kirghizia OR kirghiz OR kyrgyzstan OR Lao OR laos OR latvia OR lebanon OR lesotho OR basutoland OR liberia OR libya OR lithuania OR macedonia OR madagascar OR "Magalasy Republic" OR malawi OR Malay* OR sabah OR sarawak OR maldives OR mali OR marshall Island* OR mauritania OR mauritius OR galega Island* OR mexico OR micronesia OR Moldov* OR mongolia OR montserrat OR montenegro OR morocco OR ifni OR mozambique OR Myanma* OR namibia OR nauru OR nepal OR "Netherlands Antilles" OR "Dutch Antilles" OR "New Guinea" OR "New Caledonia" OR nicaragua OR niue OR niger OR nigeria OR ("Northern Mariana" AND Island*) OR nyasaland OR oman OR pakistan OR palau OR panama OR "Papua New Guinea" OR PNG OR palestine OR paraguay OR peru OR philippines OR philippines OR philippines OR philippines OR poland OR "Puerto Rico" OR yemen OR romania OR roumania OR rumania OR Russia* OR rwanda OR ruanda OR "Saint Kitts" OR "St Kitts" OR nevis OR "Saint Vincent" OR "St Vincent" OR grenadines OR Samoa* OR navigator Island* OR "Saint Lucia" OR "St Lucia" OR "Saint Helena" OR "St Helena" OR "Sao Tome" OR "Saudi Arabia" OR senegal OR serbia OR seychelles OR "Sierra Leone" OR slovenia OR Slovak* OR "South Africa" OR solomon Island* OR somalia OR "Sri Lanka" OR ceylon OR sudan OR Surinam* OR swaziland OR syria OR tajikistan OR tadzhikistan OR tadjikistan OR tadzhik OR tanzania OR thailand OR tibet OR timor-leste OR togo OR "Togolese Republic" OR tokelau OR tonga OR trinidad OR tobago OR tunisia OR turkey OR turkmenistan OR turkmen OR tuvalu OR uganda OR ukraine OR uruguay OR urundi OR ussr OR "Soviet Union" OR "Union of Soviet Socialist Republics" OR uzbekistan OR vanuatu OR "New Hebrides" OR venezuela OR vietnam OR "Viet Nam" OR "Wallis Futuna" OR "United Arab Republic" OR "West Bank" OR "West Indies" OR yemen OR yugoslavia OR zaire OR zambia OR zimbabwe OR rhodesia OR africa OR americas OR caribbean OR "central America" OR "latin America" OR "south America" OR "eastern Europe" OR transcaucasia OR antarctic OR atlantic island* OR "indian ocean" OR pacific island* OR polynesia OR "central asia" OR (southeast* AND asia) OR (south-east* AND asia) OR "south east asia" OR "south eastern asia" OR borneo OR mekong OR "western asia" OR "middle east" OR "far east") AND (NOTNLM OR publisher[sb] OR inprocess[sb] OR pubmednotmedline[sb] OR indatareview[sb] OR pubstatusaheadofprint))

CINAHL

Limited 1998-current

S1 (Hypoxia OR hypoxic OR hypoxaemia OR hypoxemic OR anoxic OR anoxaemia)

S2 (Respiratory-tract-infection* OR pneumonia OR malaria OR tuberculosis OR COPD OR chronic-obstructive-pulmonary-disease OR asthma OR sepsis OR meningitis OR brain-disease* OR trauma OR pregnancy OR neoplasm OR neoplasms OR risk-factor*)

S3 reproducibility OR sensitivity OR specificity OR predictive-value OR mobile-app* OR point-of-care OR incidence OR prevalence

S4	(developing countr*) OR (low and middle income) OR (LMIC)	Expanders - Apply equivalent subjects
S5	S1 AND S2 AND S4	
S6	S1 AND S3 AND S4	
S7	S1 AND S4	
S8	S2 AND S3 AND S4	
S9	S5 OR S6 OR S7 OR S8	

Text S2: Full list of data items for extraction

- Title
- Country in which the study conducted
- Geographic setting (urban/rural)
- Altitude (in metres above sea level)
- Health care setting (community, primary, secondary, tertiary)
- Ward/unit
- Aim of study
- Study design
- Start date (Month Year)
- End date (Month Year)
- Study funding and COI
- Population description
- Inclusion criteria
- Exclusion criteria
- Number recruited (i.e. in denominator population)
- Age range (specify days, weeks, months, years)
- Median age (specify days, weeks, months, years)
- Hypoxaemia definition
- Information on pulse oximeter
- Hypoxaemia data
    - Total number of eligible participants (for SpO2 measurement)
    - Number of participants with SpO2 recorded
    - Number of missing participants (elibible - recorded)
    - Number with hypoxaemia
    - Proportion with hypoxaemia (hypoxaemic/number SpO2 recorded)
    - Proportion of missing data (number missing SpO2/number eligible)
    - Notes on hypoxaemia analysis
- Age groups: neonate, child, older child, adolescent/adult
    - Total number

- Missing SpO2
- Number with hypoxaemia

- Diagnostic groups Neonate: Neonatal encephalopathy / birth asphyxia, Meconium aspiration syndrome, Pneumonia, Prematurity, Respiratory distress syndrome (RDS) / Hyline Membrane Disease (HMD), Sepsis, Pneumonia, Other conditions (specified)
    - Total number
    - Missing SpO2
    - Number with hypoxaemia
- Diagnostic groups Child: Fever, Pneumonia (and subgroups), Bronchiolitis / viral pneumonia, Anaemia, Asthma, HIV complications, Malaria, Meningitis / encephalitis, Seizures, Sepsis, Trauma, Tuberculosis, Other conditions (specified)
    - Total number
    - Missing SpO2
    - Number with hypoxaemia
- Diagnostic groups Adolescent/Adult: Fever, Pneumonia (and subgroups), Acute exacerbation COPD, Asthma, Heart failure / acute myocardial infarction, HIV complications, Malaria, Meningitis / encephalitis, Seizures, Sepsis, Trauma, Tuberculosis, Other conditions (specified)
    - Total number
    - Missing SpO2
    - Number with hypoxaemia
- Was relative odds of death reported for hypoxaemic vs normoxaemic patients (or data adequate for us to calculate)?
    - Odds of death, hypoxaemic patients
    - Odds of death, normoxaemic patients
    - Crude OR of death: hypoxaemic versus normoxaemic
    - Adjusted OR of death: hypoxaemic versus normoxaemic

Table S1: Full results table mapping

Full results table mapping included data across all conditions, with meta-estimates (if ≥3 included data points) or individual study proportions (if <3 data points)

Conditions	Admitted/required admission			Presenting in OPD/ED		
	Neonate	Child	Adult	Neonate	Child	Adult
	# articles; # participants; Proportion % (95% CI); Variance	# articles; # participants; Proportion % (95% CI); Variance	# articles; # participants; Proportion % (95% CI); Variance	# articles; # participants; Proportion % (95% CI); Variance	# articles; # participants; Proportion % (95% CI); Variance	# articles; # participants; Proportion % (95% CI); Variance
All	11; 11233; 24.5 (19.9, 29.4) I^2=94.49%, T^2=0.03, $p<0.05$	19; 125849; 12.1 (10.0, 14.4) I^2=99.03%, T^2=0.02, $p<0.05$	7; 9427; 10.8 (4.9, 18.7) I^2=98.98%, T^2=0.09, $p<0.05$	5; 4129; 11.8 (2.1, 27.1) I^2=98.43%, T^2=0.18, $p<0.05$	4; 7595; 7.0 (2.5, 13.6) I^2=98.68%, T^2=0.05, $p<0.05$	3; 12353; 4.0 (0.2, 12.3) I^2=99.63%, T^2=0.08, $p<0.05$
**Condition/Disease Specific**						
Asthma	No data	3; 209; 49.8 (13.4, 86.3) I^2=97.03%, T^2=0.49, $p<0.05$	1; 95; 16.8 (9.9, 25.9)	No data	2; 271; 3.2 (1.2, 6.8), 9.8 (4.3, 18.3)	No data
Bronchiolitis	No data	4; 968; 26.3 (2.4, 62.8) I^2=99.16%, T^2=0.56, $p<0.05$	No data	No data	3; 252; 17.8 (5.2, 35.4) I^2=89.77%, T^2=0.11, $p<0.05$	No data
COVID	No data	5; 25109; 27.5	33; 177414; 44.2	No data	No data	No data

Conditions	Admitted/required admission			Presenting in OPD/ED		
	Neonate	Child	Adult	Neonate	Child	Adult
	# articles;<br># participants;<br>Proportion %<br>(95% CI);<br>Variance	# articles;<br># participants;<br>Proportion %<br>(95% CI);<br>Variance	# articles;<br># participants;<br>Proportion %<br>(95% CI);<br>Variance	# articles;<br># participants;<br>Proportion %<br>(95% CI);<br>Variance	# articles;<br># participants;<br>Proportion %<br>(95% CI);<br>Variance	# articles;<br># participants;<br>Proportion %<br>(95% CI);<br>Variance
		(22.0, 33.3)<br>I^2=96.17%,<br>T^2=0.02, $p<0.05$	(36.6, 51.9)<br>I^2=99.84%,<br>T^2=0.20,<br>$p<0.05$			
**Diarrhoea**	No data	10;<br>2917;<br>4.1<br>(1.3, 8.0)<br>I^2=91.25%,<br>T^2=0.05, $p<0.05$	No data	No data	1;<br>300;<br>1.0<br>(0.2, 2.9)	No data
**HIV complications**	No data	3;<br>197;<br>29.5<br>(11.9, 50.7)<br>I^2=78.30%,<br>T^2=0.11, $p<0.05$	4;<br>2389;<br>16.6<br>(11.6, 22.4)<br>I^2=90.08%,<br>T^2=0.02,<br>$p<0.05$	No data	No data	1;<br>1224;<br>16.0<br>(14.0, 18.2)
**Malaria**	No data	14;<br>17455;<br>6.4<br>(4.0, 9.3)<br>I^2=97.37%,<br>T^2=0.03, $p<0.05$	No data	No data	2;<br>2611;<br>9.7<br>(8.1, 11.5),<br>0.8<br>(0.4, 1.4)	1;<br>965;<br>0.1<br>(0.0, 0.6)
**Malnutrition**	No data	13;<br>3746;<br>14.1<br>(7.7, 21.9)<br>I^2=96.76%,<br>T^2=0.12, $p<0.05$	No data	No data	No data	No data

Conditions	Admitted/required admission			Presenting in OPD/ED		
	Neonate	Child	Adult	Neonate	Child	Adult
	# articles; # participants; Proportion % (95% CI); Variance	# articles; # participants; Proportion % (95% CI); Variance	# articles; # participants; Proportion % (95% CI); Variance	# articles; # participants; Proportion % (95% CI); Variance	# articles; # participants; Proportion % (95% CI); Variance	# articles; # participants; Proportion % (95% CI); Variance
**Meningitis/ Encephalitis**	No data	6; 725; 13.7 (5.1, 25.3) I^2=91.02%, T^2=0.11, p<0.05	No data	No data	No data	No data
**Birth Asphyxia/ Neonatal Encephalopathy**	3; 2592; 32.8 (16.2, 51.8) I^2=92.78%, T^2=0.10, p<0.05	No data	No data	No data	No data	No data
**Pneumonia (Non-WHO specified)**	3; 818; 37.3 (7.6, 73.5) I^2=96.25%, T^2=0.39, p<0.05	7; 8924; 28.9 (19.1, 39.8) I^2=98.78%, T^2=0.09, p<0.05	8; 3296; 20.4 (7.7, 37.0) I^2=98.82%, T^2=0.28, p<0.05	No data	6; 1807; 24.9 (15.4, 35.8) I^2=93.08%, T^2=0.08, p<0.05	4; 1222; 15.7 (1.4, 39.4) I^2=98.36%, T^2=0.27, p<0.05
**Pneumonia (Radiology)**	No data	15; 5403; 45.3 (32.4, 58.5) I^2=98.81%, T^2=0.26, p<0.05	No data	No data	1; 97; 67.0 (56.7, 76.2)	1; 404; 4.7 (2.9, 7.3)
**Pneumonia (WHO Non-severe)**	No data	8; 3182; 14.9	No data	No data	3; 2235; 6.6	No data

Conditions	Admitted/required admission			Presenting in OPD/ED		
	Neonate	Child	Adult	Neonate	Child	Adult
	# articles; # participants; Proportion % (95% CI); Variance	# articles; # participants; Proportion % (95% CI); Variance	# articles; # participants; Proportion % (95% CI); Variance	# articles; # participants; Proportion % (95% CI); Variance	# articles; # participants; Proportion % (95% CI); Variance	# articles; # participants; Proportion % (95% CI); Variance
		(7.0, 25.0) I^2=97.75%, T^2=0.12, p<0.05			(0.0, 26.7) I^2=96.96%, T^2=0.23, p=0.12	
Pneumonia (WHO Severe)	No data	35; 38017; 36.4 (30.0, 43.0) I^2=99.41%, T^2=0.16, p<0.05	No data	No data	3; 280; 55.4 (22.7, 85.7) I^2=96.77%, T^2=0.34, p<0.05	No data
Pneumonia (broadly fitting WHO severe or non-severe classification)	No data	66; 68127; 34.6 (30.2, 39.1) I^2=99.31%, T^2=0.14, p<0.05	No data	No data	12; 22066; 25.9 (16.3, 36.8) I^2=99.52%, T^2=0.17, p<0.05	No data
Prematurity/ Low Birth Weight	3; 1620; 34.3 (19.5, 50.8) I^2=89.23%, T^2=0.07, p<0.05	No data	No data	No data	No data	No data
Sepsis	2; 3229; 44.1 (27.2, 62.1),	9; 4996; 18.7 (11.8, 26.6)	4; 2311; 25.3 (14.8, 37.4)	No data	1; 55; 0.0 (0.0, 6.5)	1; 56; 0.0 (0.0, 6.4)

Conditions	Admitted/required admission			Presenting in OPD/ED		
	Neonate	Child	Adult	Neonate	Child	Adult
	# articles; # participants; Proportion % (95% CI); Variance	# articles; # participants; Proportion % (95% CI); Variance	# articles; # participants; Proportion % (95% CI); Variance	# articles; # participants; Proportion % (95% CI); Variance	# articles; # participants; Proportion % (95% CI); Variance	# articles; # participants; Proportion % (95% CI); Variance
	21.0 (19.6, 22.5)	I^2=92.40%, T^2=0.06, p<0.05	I^2=95.41%, T^2=0.06, p<0.05			
Trauma	No data	2; 482; 2.0 (0.6, 5.0), 7.1 (4.4, 10.7)	1; 66; 37.9 (26.2, 50.7)	No data	No data	3; 1408; 8.5 (2.4, 17.7) I^2=96.08%, T^2=0.06, p<0.05
Tuberculosis	No data	1; 20; 20.0 (5.7, 43.7)	2; 703; 26.4 (16.7, 38.1), 34.2 (30.5, 38.10)	No data	No data	No data
Signs/Symptoms specific						
Anaemia	No data	6; 1379; 7.4 (1.1, 17.7) I^2=96.14%, T^2=0.14, p<0.05	No data	No data	1; 88; 18.2 (10.8, 27.8)	No data
Fever	No data	3; 3289; 14.1 (1.1, 37.3) I^2=99.30%, T^2=0.22, p<0.05	2; 538; 21.0 (14.1, 29.4), 9.8 (7.1, 13.0)	No data	2; 1985; 0.7 (0.0, 3.7), 1.0 (0.6, 1.6)	No data

Conditions	Admitted/required admission			Presenting in OPD/ED		
	Neonate	Child	Adult	Neonate	Child	Adult
	# articles; # participants; Proportion % (95% CI); Variance	# articles; # participants; Proportion % (95% CI); Variance	# articles; # participants; Proportion % (95% CI); Variance	# articles; # participants; Proportion % (95% CI); Variance	# articles; # participants; Proportion % (95% CI); Variance	# articles; # participants; Proportion % (95% CI); Variance
**Seizures**	**No data**	**2; 3398; 18.3 (16.2, 20.5), 4.1 (3.3, 5)**	**1; 119; 25.2 (17.7, 34.0)**	**No data**	**No data**	**No data**

Conditions	Admitted/required admission			Presenting in OPD/ED		
	Neonate	Child	Adult	Neonate	Child	Adult
	# articles; # of participants; Proportion % (95% CI); Variance	# of articles; # of people; Proportion % (95% CI); Variance	# of articles; # of people; Proportion % (95% CI); Variance	# of articles; # of people; Proportion % (95% CI); Variance	# of articles; # of people; Proportion % (95% CI); Variance	# of articles; # of people; Proportion % (95% CI); Variance
**All**	11; 11233; **24.5** (19.9, 29.4) I^2=94.49%, T^2=0.03, p<0.05	19; 125849; **12.1** (10.0, 14.4) I^2=99.03%, T^2=0.02, p<0.05	8; 10473; **11.0** (5.7, 17.7) I^2=98.82%, T^2=0.07, p<0.05	5; 4129; **11.8** (2.1, 27.1) I^2=98.43%, T^2=0.18, p<0.05	4; 7595; **7.0** (2.5, 13.6) I^2=98.68%, T^2=0.05, p<0.05	3; 12353; **4.0** (0.2, 12.3) I^2=99.63%, T^2=0.08, p<0.05
**Condition/Disease Specific**						
**Asthma**	No data	3;	1;	No data	2;	No data

Conditions	Admitted/required admission			Presenting in OPD/ED		
	Neonate	Child	Adult	Neonate	Child	Adult
	# articles;<br># of participants;<br>Proportion % (95% CI);<br>Variance	# of articles;<br># of people;<br>Proportion % (95% CI);<br>Variance	# of articles;<br># of people;<br>Proportion % (95% CI);<br>Variance	# of articles;<br># of people;<br>Proportion % (95% CI);<br>Variance	# of articles;<br># of people;<br>Proportion % (95% CI);<br>Variance	# of articles;<br># of people;<br>Proportion % (95% CI);<br>Variance
		209;<br>**49.8**<br>(13.4, 86.3)<br>I^2=97.03%,<br>T^2=0.49, $p<0.05$	95;<br>**16.8**<br>(9.9, 25.9)		271;<br>**3.2**<br>(1.2, 6.8),<br>**9.8**<br>(4.3, 18.3)	
**Bronchiolitis**	No data	4;<br>968;<br>**26.3**<br>(2.4, 62.8)<br>I^2=99.16%,<br>T^2=0.56, $p<0.05$	No data	No data	3;<br>252;<br>**17.8**<br>(5.2, 35.4)<br>I^2=89.77%,<br>T^2=0.11,<br>$p<0.05$	No data
**COVID**	No data	5;<br>25109;<br>**27.5**<br>(22.0, 33.3)<br>I^2=96.17%,<br>T^2=0.02, $p<0.05$	33;<br>177414;<br>**44.2**<br>(36.6, 51.9)<br>I^2=99.84%,<br>T^2=0.20,<br>$p<0.05$	No data	No data	No data
**Diarrhea**	No data	10;<br>2917;<br>**4.1**<br>(1.3, 8.0)<br>I^2=91.25%,<br>T^2=0.05, $p<0.05$	No data	No data	1;<br>300;<br>**1.0**<br>(0.2, 2.9)	No data
**HIV complications**	No data	3;<br>197;<br>**29.5**	4;<br>2389;<br>**16.6**	No data	No data	1;<br>1224;<br>**16.0**

Conditions	Admitted/required admission			Presenting in OPD/ED		
	Neonate	Child	Adult	Neonate	Child	Adult
	# articles; # of participants; Proportion % (95% CI); Variance	# of articles; # of people; Proportion % (95% CI); Variance	# of articles; # of people; Proportion % (95% CI); Variance	# of articles; # of people; Proportion % (95% CI); Variance	# of articles; # of people; Proportion % (95% CI); Variance	# of articles; # of people; Proportion % (95% CI); Variance
		(11.9, 50.7) I^2=78.30%, T^2=0.11, p<0.05	(11.6, 22.4) I^2=90.08%, T^2=0.02, p<0.05			(14.0, 18.2)
**Malaria**	No data	14; 17455; **6.4** (4.0, 9.3) I^2=97.37%, T^2=0.03, p<0.05	No data	No data	2; 2611; **9.7** (8.1, 11.5), **0.8** (0.4, 1.4)	1; 965; **0.1** (0.0, 0.6)
**Malnutrition**	No data	13; 3746; **14.1** (7.7, 21.9) I^2=96.76%, T^2=0.12, p<0.05	No data	No data	No data	No data
**Meningitis/ Encephalitis**	No data	6; 725; **13.7** (5.1, 25.3) I^2=91.02%, T^2=0.11, p<0.05	No data	No data	No data	No data
**Birth Asphyxia/ Neonatal Encephalopathy**	3; 2592; **32.8** (16.2, 51.8) I^2=92.78%, T^2=0.10, p<0.05	No data	No data	No data	No data	No data

Conditions	Admitted/required admission			Presenting in OPD/ED		
	Neonate	Child	Adult	Neonate	Child	Adult
	# articles; # of participants; Proportion % (95% CI); Variance	# of articles; # of people; Proportion % (95% CI); Variance	# of articles; # of people; Proportion % (95% CI); Variance	# of articles; # of people; Proportion % (95% CI); Variance	# of articles; # of people; Proportion % (95% CI); Variance	# of articles; # of people; Proportion % (95% CI); Variance
**Pneumonia (Non-WHO specified)**	3; 818; **37.3** (7.6, 73.5) I^2=96.25%, T^2=0.39, p<0.05	7; 8924; **28.9** (19.1, 39.8) I^2=98.78%, T^2=0.09, p<0.05	8; 3296; **20.4** (7.7, 37.0) I^2=98.82%, T^2=0.28, p<0.05	1; 150; **38.7** (30.8, 47.0)	6; 1807; **24.9** (15.4, 35.8) I^2=93.08%, T^2=0.08, p<0.05	4; 1222; **15.7** (1.4, 39.4) I^2=98.36%, T^2=0.27, p<0.05
**Pneumonia (Radiology)**	No data	15; 5403; **45.3** (32.4, 58.5) I^2=98.81%, T^2=0.26, p<0.05	No data	No data	1; 97; **67.0** (56.7, 76.2)	1; 404; **4.7** (2.9, 7.3)
**Pneumonia (WHO Non-severe)**	No data	8; 3182; **14.9** (7.0, 25.0) I^2=97.75%, T^2=0.12, p<0.05	No data	No data	3; 2235; **6.6** (0.0, 26.7) I^2=96.96%, T^2=0.23, p=0.12	No data
**Pneumonia (WHO Severe)**	No data	34; 37567; **34.8** (28.8, 41.1) I^2=99.34%, T^2=0.14, p<0.05	No data	No data	4; 730; **63.9** (30.5, 91.2) I^2=98.46%, T^2=0.46, p<0.05	No data

Conditions	Admitted/required admission			Presenting in OPD/ED		
	Neonate	Child	Adult	Neonate	Child	Adult
	# articles; # of participants; Proportion % (95% CI); Variance	# of articles; # of people; Proportion % (95% CI); Variance	# of articles; # of people; Proportion % (95% CI); Variance	# of articles; # of people; Proportion % (95% CI); Variance	# of articles; # of people; Proportion % (95% CI); Variance	# of articles; # of people; Proportion % (95% CI); Variance
**Pneumonia (broadly fitting WHO severe or non-severe classification)**	No data	65; 67677; **33.8** (29.5, 38.1) I^2=99.27%, T^2=0.13, $p<0.05$	No data	No data	13; 22516; **30.4** (18.4, 44.0) I^2=99.67%, T^2=0.26, $p<0.05$	No data
**Prematurity/ Low Birth Weight**	3; 1620; **34.3** (19.5, 50.8) I^2=89.23%, T^2=0.07, $p<0.05$	No data	No data	No data	No data	No data
**Sepsis**	2; 3229; **44.1** (27.2, 62.1), **21.0** (19.6, 22.5)	9; 4996; **18.7** (11.8, 26.6) I^2=92.40%, T^2=0.06, $p<0.05$	4; 2311; **25.3** (14.8, 37.4) I^2=95.41%, T^2=0.06, $p<0.05$	No data	1; 55; **0.0** (0.0, 6.5)	1; 56; **0.0** (0.0, 6.4)
**Trauma**	No data	2; 482; **2.0** (0.6, 5.0), **7.1** (4.4, 10.7)	1; 66; **37.9** (26.2, 50.7)	No data	No data	3; 1408; **8.5** (2.4, 17.7) I^2=96.08%, T^2=0.06, $p<0.05$
**Tuberculosis**	No data	1;	2;	No data	No data	No data

Conditions	Admitted/required admission			Presenting in OPD/ED		
	Neonate	Child	Adult	Neonate	Child	Adult
	# articles; # of participants; Proportion % (95% CI); Variance	# of articles; # of people; Proportion % (95% CI); Variance	# of articles; # of people; Proportion % (95% CI); Variance	# of articles; # of people; Proportion % (95% CI); Variance	# of articles; # of people; Proportion % (95% CI); Variance	# of articles; # of people; Proportion % (95% CI); Variance
		20; **20.0** (5.7, 43.7)	703; **26.4** (16.7, 38.1), **34.2** (30.5, 38.10)			
**Signs/Symptoms specific**						
**Anemia**	No data	6; 1379; **7.4** (1.1, 17.7) I^2=96.14%, T^2=0.14, $p<0.05$	No data	No data	1; 88; **18.2** (10.8, 27.8)	No data
**Fever**	No data	3; 3289; **14.1** (1.1, 37.3) I^2=99.30%, T^2=0.22, $p<0.05$	2; 538; **21.0** (14.1, 29.4), **9.8** (7.1, 13.0)	No data	2; 1985; **0.7** (0.0, 3.7), **1.0** (0.6, 1.6)	No data
**Seizures**	No data	2; 3398; **18.3** (16.2, 20.5), **4.1** (3.3, 5)	1; 119; **25.2** (17.7, 34.0)	No data	No data	No data

p<0.05 indicates there is heterogeneity among studies which is statistically significant; green denotes meta-estimate possible, yellow means meta-estimate not possible due to less data points, grey mean no data available.

Table S2: Full results table mapping – sensitivity analysis

Full results table mapping included data across all conditions, with meta-estimates (if ≥3 included data points) or individual study proportions (if <3 data points) for results SpO2<90%.

Conditions	Admitted/required admission			Presenting in OPD/ED		
	Neonate	Child	Adult	Neonate	Child	Adult
	# articles;<br># participants;<br>Proportion %<br>(95% CI);<br>Variance	# articles;<br># participants;<br>Proportion %<br>(95% CI);<br>Variance	# articles;<br># participants;<br>Proportion %<br>(95% CI);<br>Variance	# articles;<br># participants;<br>Proportion %<br>(95% CI);<br>Variance	# articles;<br># participants;<br>Proportion %<br>(95% CI);<br>Variance	# of articles;<br># of people;<br>Proportion %<br>(95% CI);<br>Variance
All	9;<br>10694;<br>22.0<br>(17.7, 26.7)<br>I^2=93.60%,<br>T^2=0.02, $p<0.05$	17;<br>121184;<br>10.5<br>(8.6, 12.5)<br>I^2=98.82%,<br>T^2=0.02, $p<0.05$	6;<br>5323;<br>8.9<br>(5.7, 12.7)<br>I^2=93.45%,<br>T^2=0.02, $p<0.05$	4;<br>3929;<br>6.8<br>(0.3, 18.7)<br>I^2=97.65%,<br>T^2=0.12, $p<0.05$	4;<br>7595;<br>7.0<br>(2.5, 13.6)<br>I^2=98.68%,<br>T^2=0.05, $p<0.05$	2;<br>5053;<br>0.1<br>(0.0, 0.3)<br>12.1<br>(10.6, 13.7)
Condition/Disease Specific						
Asthma	No data	2;<br>158;<br>20.4<br>(12.9, 29.7)<br>83.3<br>(71.5, 91.7)	1;<br>95;<br>16.8<br>(9.9, 25.9)	No data	1;<br>82;<br>9.8<br>(4.3, 18.3)	No data
Bronchiolitis	No data	2;<br>686;<br>25.7<br>(21.9, 30.0)<br>14.4<br>(10.0, 19.8)	No data	No data	1;<br>90;<br>8.9<br>(3.9, 16.8)	No data
COVID	No data	1;<br>112;<br>49.1<br>(39.5, 58.7)	9;<br>7326;<br>49.3<br>(24.7, 74.0)<br>I^2=99.78%,<br>T^2=0.61, $p<0.05$	No data	No data	No data

Conditions	Admitted/required admission			Presenting in OPD/ED		
	Neonate	Child	Adult	Neonate	Child	Adult
	# articles;<br># participants;<br>Proportion %<br>(95% CI);<br>Variance	# articles;<br># participants;<br>Proportion %<br>(95% CI);<br>Variance	# articles;<br># participants;<br>Proportion %<br>(95% CI);<br>Variance	# articles;<br># participants;<br>Proportion %<br>(95% CI);<br>Variance	# articles;<br># participants;<br>Proportion %<br>(95% CI);<br>Variance	# of articles;<br># of people;<br>Proportion %<br>(95% CI);<br>Variance
**Diarrhoea**	No data	8;<br>1956;<br>4.6<br>(1.9, 8.3)<br>I^2=77.87%,<br>T^2=0.03, $p<0.05$	No data	No data	1;<br>300;<br>1.0<br>(0.2, 2.9)	No data
**HIV complications**	No data	2;<br>39;<br>55.0<br>(31.5, 76.9)<br>10.5<br>(1.3, 33.1)	3;<br>2187;<br>14.0<br>(10.4, 18.1)<br>I^2=81.36%,<br>T^2=0.01, $p<0.05$	No data	No data	1;<br>1224;<br>16.0<br>(14.0, 18.2)
**Malaria**	No data	12;<br>16788;<br>7.0<br>(4.4, 10.1)<br>I^2=97.76%,<br>T^2=0.04, $p<0.05$	No data	No data	1;<br>1363;<br>0.8<br>(0.4, 1.4)	1;<br>965;<br>0.1<br>(0.0, 0.6)
**Malnutrition**	No data	9;<br>2874;<br>7.6<br>(3.9, 12.2)<br>I^2=91.16%,<br>T^2=0.04, $p<0.05$	No data	No data	No data	No data
**Meningitis/ Encephalitis**	No data	5;<br>685;<br>8.8<br>(3.2, 16.6)<br>I^2=84.35%,	No data	No data	No data	No data

Conditions	Admitted/required admission			Presenting in OPD/ED		
	Neonate	Child	Adult	Neonate	Child	Adult
	# articles;<br># participants;<br>Proportion %<br>(95% CI);<br>Variance	# articles;<br># participants;<br>Proportion %<br>(95% CI);<br>Variance	# articles;<br># participants;<br>Proportion %<br>(95% CI);<br>Variance	# articles;<br># participants;<br>Proportion %<br>(95% CI);<br>Variance	# articles;<br># participants;<br>Proportion %<br>(95% CI);<br>Variance	# of articles;<br># of people;<br>Proportion %<br>(95% CI);<br>Variance
		$T^2=0.06$, $p<0.05$				
Birth Asphyxia/ Neonatal Encephalopathy	1;<br>2458;<br>33.4<br>(31.5, 35.3)	No data	No data	No data	No data	No data
Pneumonia (Non-WHO specified)	1;<br>749;<br>11.9<br>(9.7, 14.4)	5;<br>5407;<br>30.1<br>(13.6, 49.8)<br>$I^2=98.54\%$,<br>$T^2=0.21$, $p<0.05$	4;<br>2736;<br>31.9<br>(8.7, 61.5)<br>$I^2=99.40\%$,<br>$T^2=0.37$, $p<0.05$	No data	5;<br>1759;<br>22.5<br>(12.6, 34.3)<br>$I^2=94.15\%$,<br>$T^2=0.08$, $p<0.05$	4;<br>1222;<br>15.7<br>(1.4, 39.4)<br>$I^2=98.36\%$,<br>$T^2=0.27$, $p<0.05$
Pneumonia (Radiology)	No data	10;<br>2984;<br>38.4<br>(23.6, 54.3)<br>$I^2=98.61\%$,<br>$T^2=0.26$, $p<0.05$	No data	No data	1;<br>97;<br>67.0<br>(56.7, 76.2)	1;<br>404;<br>4.7<br>(2.9, 7.3)
Pneumonia (WHO Non-severe)	No data	7;<br>3081;<br>14.3<br>(5.9, 25.2)<br>$I^2=98.05\%$,<br>$T^2=0.12$, $p<0.05$	No data	No data	3;<br>2235;<br>6.6<br>(0.0, 26.7)<br>$I^2=96.96\%$,<br>$T^2=0.23$, $p<0.05$	No data
Pneumonia (WHO Severe)	No data	27;<br>27137;<br>34.7<br>(27.2, 42.6)<br>$I^2=99.42\%$,<br>$T^2=0.18$, $p<0.05$	No data	No data	3;<br>280;<br>55.4<br>(22.7, 85.7)<br>$I^2=96.77\%$,<br>$T^2=0.34$, $p<0.05$	No data

Conditions	Admitted/required admission			Presenting in OPD/ED		
	Neonate	Child	Adult	Neonate	Child	Adult
	# articles; # participants; Proportion % (95% CI); Variance	# articles; # participants; Proportion % (95% CI); Variance	# articles; # participants; Proportion % (95% CI); Variance	# articles; # participants; Proportion % (95% CI); Variance	# articles; # participants; Proportion % (95% CI); Variance	# of articles; # of people; Proportion % (95% CI); Variance
Pneumonia (broadly fitting WHO severe or non-severe classification)	No data	51; 55109; 34.1 (29.2, 39.2) I^2=99.35%, T^2=0.15, p<0.05	No data	No data	10; 20232; 25.1 (15.6, 36.1) I^2=99.42%, T^2=0.14, p<0.05	No data
Prematurity/ Low Birth Weight	1; 1500; 25.8 (23.6, 28.1)	No data	No data	No data	No data	No data
Sepsis	1; 3195; 21.0 (19.6, 22.5)	6; 4808; 13.9 (8.2, 20.7) I^2=90.41%, T^2=0.04, p<0.05	3; 2291; 20.7 (11.0, 32.5) I^2=96.46%, T^2=0.05, p<0.05	No data	1; 55; 0.0 (0.0, 6.5)	1; 56; 0.0 (0.0, 6.4)
Trauma	No data	2; 482; 7.1 (4.4, 10.7) 2.0 (0.6, 5.0)	1; 66; 37.9 (26.2, 50.7)	No data	No data	2; 874; 3.4 (2.2, 5.1) 20.6 (15.3, 26.8)
Tuberculosis	No data	1; 20; 20.0 (5.7, 43.7)	No data	No data	No data	No data
Signs/Symptoms specific						
Anemia	No data	6; 1379;	No data	No data	1; 88;	No data

Conditions	Admitted/required admission			Presenting in OPD/ED		
	Neonate	Child	Adult	Neonate	Child	Adult
	# articles; # participants; Proportion % (95% CI); Variance	# articles; # participants; Proportion % (95% CI); Variance	# articles; # participants; Proportion % (95% CI); Variance	# articles; # participants; Proportion % (95% CI); Variance	# articles; # participants; Proportion % (95% CI); Variance	# of articles; # of people; Proportion % (95% CI); Variance
		7.4 (1.1, 17.7) I^2=96.14%, T^2=0.14, $p<0.05$			18.2 (10.8, 27.8)	
Fever	No data	3; 3289; 14.1 (1.1, 37.3) I^2=99.30%, T^2=0.22, $p<0.05$	2; 538; 9.8 (7.1, 13.0) 21.0 (14.1, 29.4)	No data	2; 1985; 1.0 (0.6, 1.5) 0.7 (0.0, 3.7)	No data
Seizures	No data	2; 3398; 4.1 (3.3, 5.0) 18.3 (16.2, 20.5)	1; 119; 25.2 (17.7, 34.0)	No data	No data	No data

Table S3: Hypoxaemia burden in children: pneumonia versus overall

	**Overall child population**			**Child pneumonia population**				**Hypoxaemic pneumonia as propoprtion of all hypoxaemia**
**Study**	**Number overall**	**Hypoxaemia**	**Hypoxaemia prevalence**	**Number with pneumonia**	**Pneumonia as proportion of population**	**Hypoxaemia**	**Hypoxaemia prevalence**	
Duke 2002[1]	359	200	56%	223	62%	162	73%	81%
Graham 2022[2]	2480	24	1%	142	6%	8	6%	33%
Graham 2019[3]	16453	1304	8%	2073	13%	486	23%	37%
Junge 2006[4]	3269	188	6%	436	13%	51	12%	27%
King 2022[5]	788	161	20%	111	14%	63	57%	39%
McCollum 2013[6]	727	33	5%	151	21%	26	17%	79%
Mwaniki 2009[7]	13183	693	5%	5489	42%	461	8%	67%
Orimadegun 2013[8]	1272	306	24%	313	25%	154	49%	50%
Wandi 2006[9]	1896	458	24%	578	30%	315	54%	69%
**TOTAL (Mean)**	**40427**	**3367**	**8%** (median)	**9516**	**21%** (median)	**1726**	**23%** (median)	**50% (median)**

Figure S1: Secondary versus Tertiary facilities

Meta-estimates of hypoxaemia prevalence among neonates, children, and adults admitted to Level 2 (secondary/general hospitals) and Level 3 (tertiary/referral hospitals) facilities.

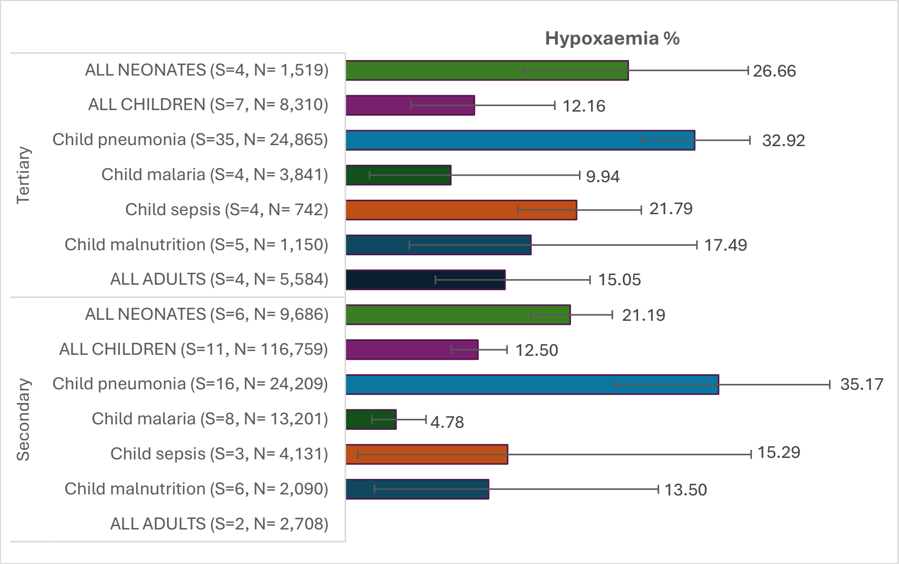

Figure S2: Secondary versus Tertiary facilities – sensitivity analysis

Meta-estimates of hypoxaemia prevalence among neonates, children, and adults admitted to Level 2 (secondary/general hospitals) and Level 3 (tertiary/referral hospitals) facilities (for papers with SpO_2 cut-off <90%)

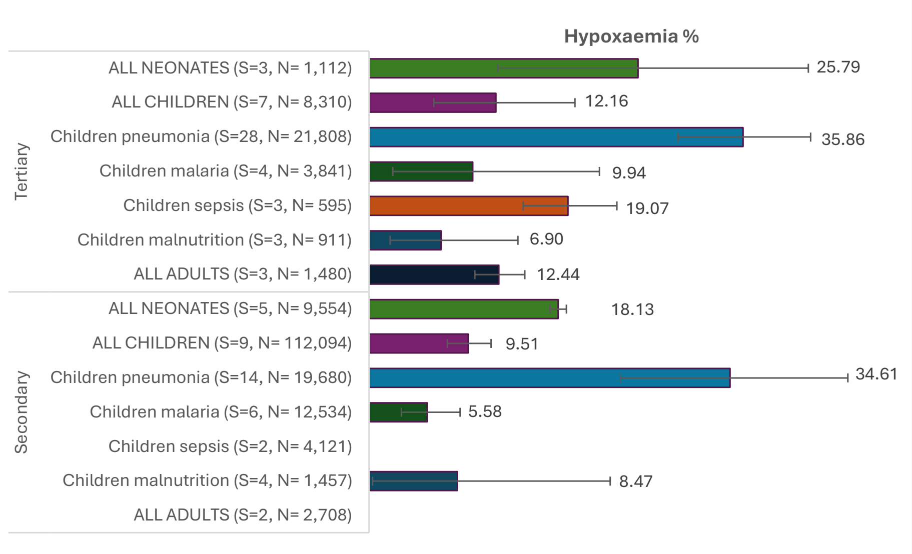

Figure S3: Hypoxaemia prevalence – sensitivity analysis

Hypoxaemia prevalence among hospitalised neonates, children, and adolescents and adults, reporting random-effects pooled estimates across different subgroups (for papers with SpO_2 cut-off <90%)

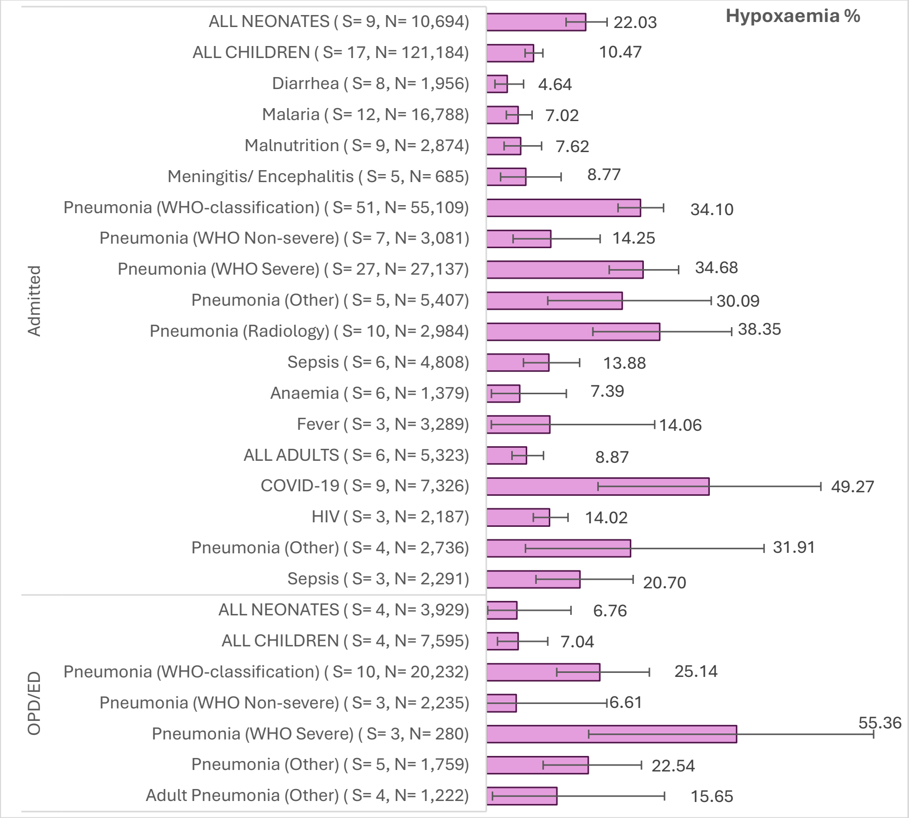

Figure S3: Altitude scatterplot – sensitivity analysis

Scatterplot showing hypoxaemia prevalence among hospitalised neonates (A), children (B), and adolescents and adults (C) by altitude (papers reporting SpO_2<90%)

(A)

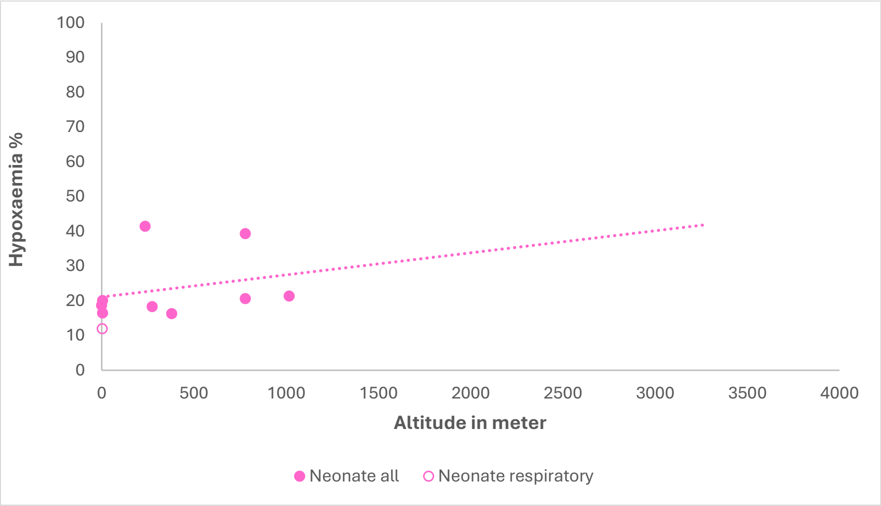

(B)

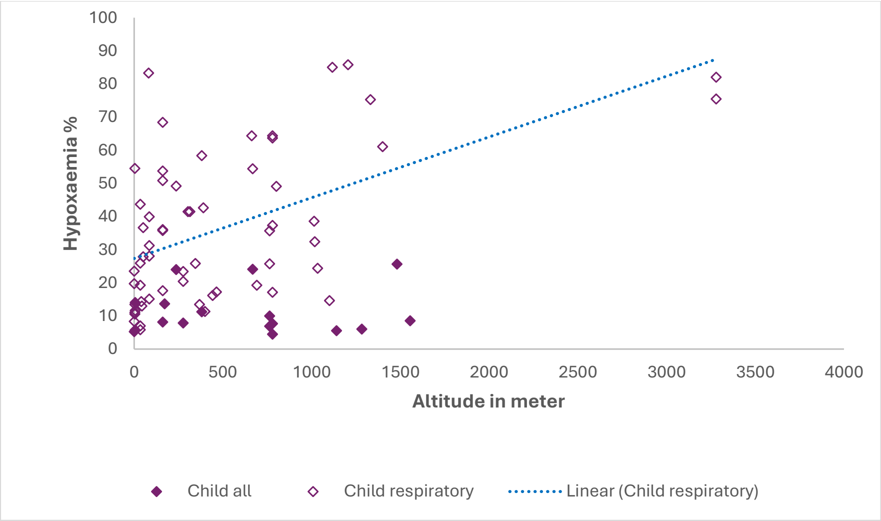

(C)

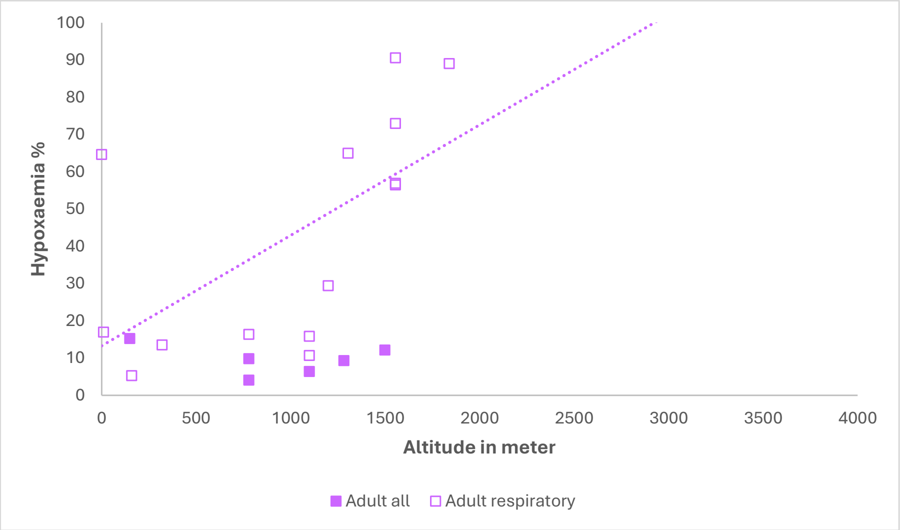
100
90
80
70
60
50
40
30
20
10
0
Hypoxaemia %
0
500
1000
1500
2000
2500
3000
3500
4000
Altitude in meter
Adult all
Adult respiratory

Figure S3: Relative odds of death – sensitivity analysis

Relative odds of death comparing those with hypoxaemia to those without hypoxaemia (papers reporting SpO_2 <90%)

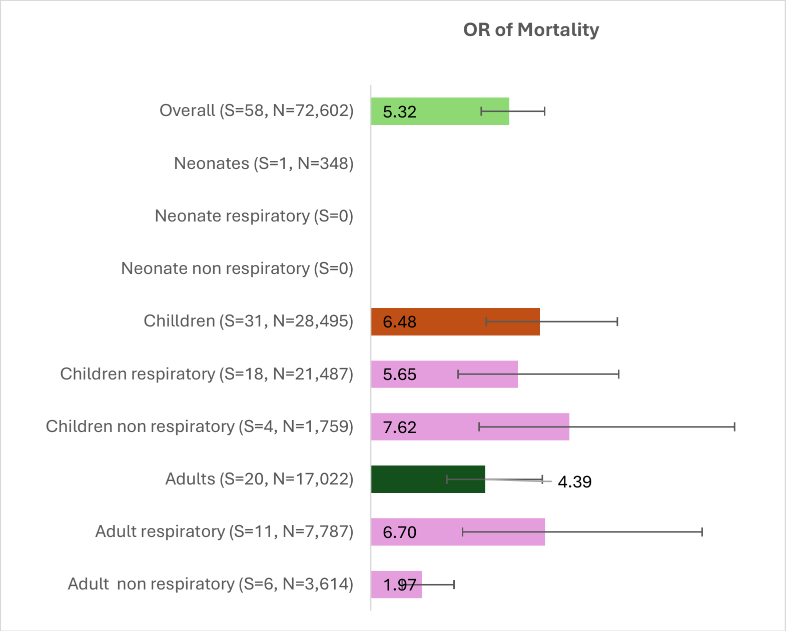

Table S4: Risk of bias assessments

Risk of bias assessments were conducted using the Joanna Briggs Institute's Checklist for Prevalence Studies, a widely used quality assessment tool that was specifically designed for prevalence studies.[10,11]

Grading summary

SAMPLING FRAME: Was the sample frame appropriate to address the target population?

- YES (sample frame appropriate for the target population)
- NO (sample frame not appropriate for the target population)

RECRUITMENT: Were study participants recruited in an appropriate way?

- YES (complete or random selection from the target population over a representative time period)
- NO (convenience sample or other incomplete/non-random selection, or recruited from a biased sub-population, or biased time period)

SAMPLE SIZE: Was the sample size adequate?

- YES (>200 participants for largest population reported)
- NO (<200 participants for largest population reported)

SUBJECTS & SETTING: Were the study subjects and setting described in detail?

- YES (clear description of study subjects and setting)
- NO (inadequate description of study subjects or setting)

MISSING DATA: Was outcome data adequately reported for the population?

- YES (SpO2 documented for >80% of study population)
- NO (SpO2 documented for <80% of study population)

COVERAGE BIAS: Was data analysis conducted with sufficient coverage of the identified sample?

- YES (SpO2 coverage similar across all subgroups)
- No (SpO2 coverage low for certain subgroups)
- Not applicable (no sub-populations)

VALID DIAGNOSTIC METHODS: Were valid methods used for identification of hypoxaemia and diagnostic categories?

- YES (Used clearly-described standard case definitions, e.g. WHO or national guidelines)
- NO (Used arbitrary case definitions or 'clinician diagnosis' only)
- Not applicable (No diagnostic sub-categories)

MEASUREMENT: Was SpO2 measured in a standard, reliable way for all participants?

- YES (SpO2 conducted by trained staff according to a guideline/procedure)
- NO (SpO2 conducted by untrained staff or without a guideline/procedure)

STATISTICAL ANALYSIS: Was there appropriate statistical analysis? Specifically, was numerator and denominator reported?

- YES (Numerator and Denominator reported, with description of any additional analysis)
- NO (Numerator or Denominator not reported)

Author & Year	Title	Sampling frame	Recruitment	Samp size	Subjects & setting	Missing data	Coverage bias	Diagnostic methods	Measureme nt	Stat analysis
AbdelGhaffar, 2022	Prediction of mortality in hospitalized Egyptian patients with Coronavirus disease-2019: A multicenter retrospective study	YES	YES	YES	YES	YES	YES	NO	UNCLEAR	YES
Abdulkadir 2015	Hypoxaemia as a measure of disease severity in young hospitalised Nigerian children with pneumonia: A cross-sectional study	YES	YES	YES	YES	YES	YES	YES	YES	YES
Acar 2021	An easy-to-use nomogram for predicting in-hospital mortality risk in COVID-19: a retrospective cohort study in a university hospital.	YES	YES	YES	NO	YES	YES	NO	UNCLEAR	YES
Addo-Yobo, 2004	Oral amoxicillian versus inectable penicillin for severe pneumonia in children aged 3 to 59 months: a randomized multicentre equivalency study	YES	YES	YES	YES	YES	YES	YES	YES	YES
Adebola, 2014	Hypoxemia predicts death from severe falciparum malaria among children under 5 years of age in Nigeria: the need for pulse oximetry in case management	YES	YES	YES	YES	YES	YES	YES	YES	YES
Agrawal 2011	Relationship of respiratory symptoms and signs with hypoxemia in infants under 2 months of age	YES	YES	NO	YES	YES	YES	YES	YES	YES
Ahmed 2022	Soluble T cell immunoglobulin and mucin-domain containing protein 3 in children hospitalized with pneumonia in resource-limited settings	YES	UNCLEAR	NO	NO	YES	YES	YES	UNCLEAR	YES
Al Janabi 2009		YES	YES	YES	YES	YES	YES	YES	YES	YES
Alizadehsani 2022	Factors associated with mortality in hospitalized cardiovascular	YES	YES	YES	NO	YES	NOT APPLICABLE	NO	YES	NO

Author & Year	Title	Sampling frame	Recruitment	Samp size	Subjects & setting	Missing data	Coverage bias	Diagnostic methods	Measurement	Stat analysis
	disease patients infected with COVID-19.									
Alva 2022	Risk factors for intensive care unit admission or mortality in adults hospitalized for COVID-19 at high altitude.	YES	YES	YES	NO	YES	NOT APPLICABLE	NO	UNCLEAR	YES
Alwadhi 2017	Tachypnea and other danger signs vs pulse oximetry for prediction of hypoxia in severe pneumonia/very severe disease	YES	YES	NO	NO	YES	YES	YES	UNCLEAR	YES
Amare 2008	Status epilepticus: Clinical presentation, cause, outcome, and predictors of death in 119 Ethiopian patients	YES	YES	NO	YES	YES	YES	YES	UNCLEAR	YES
Anyaypoma-Ocon 2021	Factors associated with COVID-19 lethality in a hospital in the Cajamarca region in Peru.	YES	YES	YES	UNCLEAR	YES	YES	YES	UNCLEAR	YES
Araban 2022	Epidemiological and clinical characteristics of patients with COVID-19 in Islamic Republic of Iran.	YES	YES	YES	NO	YES	NOT APPLICABLE	NO	UNCLEAR	YES
Arana-Calderon 2022	Risk factors associated with mortality in patients with SARS-CoV-2 pneumonia in a Level I Social Security Hospital, Peru-La Libertad	YES	YES	NO	YES	YES	NOT APPLICABLE	YES	UNCLEAR	YES
Asghar 2008	Chloramphenicol versus ampicillin plus gentamicin for community acquired very severe pneumonia among children aged 2-59 months in low resource settings: Multicentre randomised controlled trial (SPEAR study)	YES	YES	YES	YES	YES	YES	YES	YES	YES
Ashraf 2008	Day-care management of severe and very severe pneumonia, without associated co-morbidities such as severe malnutrition, in an urban health clinic in Dhaka, Bangladesh	YES	YES	YES	YES	YES	YES	YES	UNCLEAR	YES

Author & Year	Title	Sampling frame	Recruitment	Samp size	Subjects & setting	Missing data	Coverage bias	Diagnostic methods	Measurement	Stat analysis
Ashraf 2010	Randomized controlled trial of day care versus hospital care of severe pneumonia in Bangladesh.	YES	YES	YES	YES	YES	NOT APPLICABLE	YES	YES	YES
Ashraf 2019	Day clinic vs. hospital care of pneumonia and severe malnutrition in children under five: a randomised trial	YES	YES	YES	YES	YES	YES	YES	UNCLEAR	YES
Aslam 2021	Frequency of undiagnosed hypoxia in patients	YES	UNCLEAR	YES	YES	YES	YES	YES	UNCLEAR	YES
Aston 2019	Etiology and Risk Factors for Mortality in an Adult Community-acquired Pneumonia Cohort in Malawi	UNCLEAR	UNCLEAR	YES	YES	YES	YES	YES	UNCLEAR	YES
Awasthi 2022	Epidemiology of Hypoxic Community-Acquired Pneumonia in Children Under 5 Years of Age: An Observational Study in Northern India	YES	YES	YES	YES	YES	YES	YES	YES	YES
Barennes 2016	High mortality risk in hypoglycemic and dysglycemic children admitted at a referral hospital in a non malaria tropical setting of a low income country	YES	NO	YES	YES	YES	NOT APPLICABLE	YES	UNCLEAR	YES
Basnet 2006	Hypoxemia in children with pneumonia and its clinical predictors	UNCLEAR	UNCLEAR	YES	NO	YES	YES	YES	UNCLEAR	YES
Basnet 2015	Predictors of duration and treatment failure of severe pneumonia in hospitalized young Nepalese children	YES	YES	YES	YES	YES	YES	YES	YES	UNCLEAR
Bassat 2016	Hypoxaemia in Mozambican children <5 years of age admitted to hospital with clinical severe pneumonia: clinical features and performance of predictor models	YES	YES	YES	YES	YES	YES	YES	YES	YES
Benet 2017	Severity of pneumonia in under 5-year-old children from developing countries: A	YES	YES	YES	YES	YES	YES	YES	UNCLEAR	YES

Author & Year	Title	Sampling frame	Recruitment	Samp size	Subjects & setting	Missing data	Coverage bias	Diagnostic methods	Measureme nt	Stat analysis
	multicenter, prospective, observational study									
Bepouka 2020	Predictors of mortality in COVID-19 patients at Kinshasa University Hospital, Democratic Republic of the Congo, from March to June 2020.	YES	YES	NO	YES	YES	NOT APPLICABLE	NO	UNCLEAR	YES
Bhargava 2016	Scrub typhus in uttarakhand & adjoining Uttar Pradesh: Seasonality, clinical presentations & predictors of mortality	YES	YES	YES	YES	YES	NOT APPLICABLE	YES	UNCLEAR	YES
Bills 2020	Acute respiratory illness among a prospective cohort of pediatric patients using emergency medical services in India: Demographic and prehospital clinical predictors of mortality	YES	NO	YES	YES	YES	NOT APPLICABLE	YES	UNCLEAR	YES
Blanc 2019	Retrospective study on the usefulness of pulse oximetry for the identification of young children with severe illnesses and severe pneumonia in a rural outpatient clinic of Papua New Guinea.	YES	YES	YES	YES	YES	NOT APPLICABLE	YES	YES	YES
Boonmee 2020	Predictors of Mortality in Elderly and Very Elderly Emergency Patients with Sepsis: A Retrospective Study.	YES	YES	YES	YES	YES	UNCLEAR	YES	UNCLEAR	YES
Boyce 2017	Use of a Dual-Antigen Rapid Diagnostic Test to Screen Children for Severe Plasmodium falciparum Malaria in a High-Transmission, Resource-Limited Setting.	YES	YES	YES	YES	UNCLEAR	NOT APPLICABLE	YES	UNCLEAR	NO
Breiman 2015	Severe acute respiratory infection in children in a densely populated urban slum in Kenya, 2007-2011	YES	YES	YES	YES	YES	YES	YES	UNCLEAR	UNCLEAR

Author & Year	Title	Sampling frame	Recruitment	Samp size	Subjects & setting	Missing data	Coverage bias	Diagnostic methods	Measurement	Stat analysis
Bruce 2007	Pneumonia case-finding in the RESPIRE Guatemala indoor air pollution trial: standardizing methods for resource-poor settings.	YES	YES	YES	YES	YES	NOT APPLICABLE	YES	YES	YES
Bui-Binh-Bao 2021	Is Serum Lactate a Good Predictor of Mortality in Children Aged 2 Months to 5 Years With Pneumonia in Central Vietnam	YES	YES	YES	NO	YES	NOT APPLICABLE	YES	UNCLEAR	YES
Caio-SimoesSOUZA 2014	Severe hypoxaemia can predict unfavourable clinical outcomes in individuals with pulmonary embolism aged over 40 years	YES	YES	NO	YES	YES	NOT APPLICABLE	YES	UNCLEAR	YES
Carugati 2018	Predicting Mortality for Adolescent and Adult Patients with Fever in Resource-Limited Settings.	YES	YES	YES	YES	YES	NOT APPLICABLE	YES	UNCLEAR	YES
Chan 2002	RISK FACTORS FOR HYPOXEMIA AND RESPIRATORY FAILURE IN RESPIRATORY SYNCYTIAL VIRUS BRONCHIOLITIS	YES	YES	YES	YES	YES	NOT APPLICABLE	YES	YES	YES
Chaudhary 2020	Clinical predictors of radiological pneumonia: A cross-sectional study from a tertiary hospital in Nepal	YES	YES	NO	YES	UNCLEAR	NOT APPLICABLE	YES	UNCLEAR	NO
Chinawa 2013	Prevalence of hypoxemia among children with sickle cell anemia during steady state and crises: a cross-sectional study.	UNCLEAR	YES	NO	YES	YES	NOT APPLICABLE	YES	YES	YES
Chisti 2013	Predictors and outcome of hypoxemia in severely malnourished children under five with pneumonia: a case control design.	YES	YES	YES	YES	YES	NOT APPLICABLE	YES	UNCLEAR	YES
Chisti 2021	Antibiotic-Resistant Bacteremia in Young Children Hospitalized with Pneumonia in Bangladesh Is Associated with a High Mortality Rate	YES	YES	YES	YES	YES	NOT APPLICABLE	YES	UNCLEAR	YES

Author & Year	Title	Sampling frame	Recruitment	Samp size	Subjects & setting	Missing data	Coverage bias	Diagnostic methods	Measurement	Stat analysis
Chisti 2022	Prevalence and outcome of anemia among children hospitalized for pneumonia and their risk of mortality in a developing country	YES	YES	YES	YES	YES	NOT APPLICABLE	YES	UNCLEAR	YES
CHISTI, 2011	Co-morbidity: exploring the clinical overlap between pneumonia and diarrhoea in a hospital in Dhaka, Bangladesh	YES	YES	YES	YES	YES	YES	YES	YES	YES
Colbourn 2020	Predictive value of pulse oximetry for mortality in infants and children presenting to primary care with clinical pneumonia in rural Malawi: A data linkage study	YES	UNCLEAR	YES	YES	YES	YES	YES	YES	YES
Cserti-Gazdewich 2013	Inter-relationships of cardinal features and outcomes of symptomatic pediatric Plasmodium falciparum MALARIA in 1,933 children in Kampala, Uganda.	YES	YES	YES	YES	YES	YES	YES	UNCLEAR	YES
Davis, 2010	Clinical and Radiographic Factors Do NOT Accurately Diagnose Smear-Negative Tuberculosis in HIV-infected Inpatients in Uganda: A cross-Sectional Study	YES	YES	YES	YES	YES	YES	UNCLEAR	UNCLEAR	YES
deJesus 2022	Profile of Brazilian inpatients with COVID-19 vaccine breakthrough infection and risk factors for unfavorable outcome	YES	YES	YES	YES	YES	NOT APPLICABLE	NO	UNCLEAR	YES
Dembele 2019	Aetiology and risks factors associated with the fatal outcomes of childhood pneumonia among hospitalised children in the Philippines from 2008 to 2016: a case series study.	YES	YES	YES	YES	YES	NOT APPLICABLE	YES	UNCLEAR	YES

Author & Year	Title	Sampling frame	Recruitment	Samp size	Subjects & setting	Missing data	Coverage bias	Diagnostic methods	Measurement	Stat analysis
Diaz-Velez 2021	Risk factors for mortality in hospitalized patients with COVID-19 from three hospitals in Peru: a retrospective cohort study.	YES	YES	YES	YES	YES	NOT APPLICABLE	YES	UNCLEAR	YES
Diendere 2021	Predictors of severe hypoxemia among COVID-19 patients in Burkina Faso (West Africa): Findings from hospital based cross-sectional study	YES	YES	YES	YES	YES	NOT APPLICABLE	YES	YES	YES
Djelantik 2003	Case fatality proportions and predictive factors for mortality among children hospitalized with severe pneumonia in a rural developing country setting.	YES	YES	YES	YES	YES	NOT APPLICABLE	YES	YES	YES
Duke 2002	Hypoxaemia in acute respiratory and non-respiratory illnesses in neonates and children in a developing country	YES	YES	YES	YES	YES	YES	YES	YES	YES
Duke 2008		YES	YES	YES	YES	YES	YES	YES	YES	YES
Emdin 2015	Utility and feasibility of integrating pulse oximetry into the routine assessment of young infants at primary care clinics in Karachi, Pakistan: A cross-sectional study	YES	YES	YES	YES	YES	NOT APPLICABLE	YES	YES	YES
English 2003	Causes and outcome of young infant admissions to a Kenyan district hospital	YES	YES	YES	YES	YES	YES	YES	YES	YES
Enoch 2019	the Clinical Information Network, McGivern G, Shepperd S (2019) Variability in the use of pulse oximeters with children in Kenyan hospitals: A mixed-methods analysis	YES	NO	YES	NO	NO	NOT APPLICABLE	UNCLEAR	UNCLEAR	NO
Evans 2012	Oxygen saturations of medical inpatients in a Malawian hospital: cross-sectional study of oxygen supply and demand	UNCLEAR	YES	NO	NO	YES	YES	YES	UNCLEAR	YES

Author & Year	Title	Sampling frame	Recruitment	Samp size	Subjects & setting	Missing data	Coverage bias	Diagnostic methods	Measurement	Stat analysis
Fagbohun 2020	Application of a prognostic scale to estimate the outcome of hospitalized children with pneumonia	YES	YES	YES	YES	YES	NOT APPLICABLE	YES	UNCLEAR	YES
Faruk 2022	Death in severely malnourished hospitalized children presenting with diarrhea and vomiting	YES	YES	YES	YES	YES	NOT APPLICABLE	YES	UNCLEAR	YES
Fashanu 2020	Improved oxygen systems at hospitals in three Nigerian states: An implementation research study	YES	YES	YES	YES	YES	NOT APPLICABLE	NO	UNCLEAR	YES
Foran 2010	Prevalence of undiagnosed hypoxemia in adults and children in an under-resourced district hospital in Zambia	YES	NO	NO	YES	YES	YES	YES	YES	YES
Friedman, 2020	Excess Out-of-Hospital Mortality and Declining Oxygen Saturation: The Sentinel Role of Emergency Medical Services Data in the COVID-19 Crisis in Tijuana, Mexico	YES	YES	YES	YES	YES	YES	UNCLEAR	UNCLEAR	YES
Graham 2019	Hypoxaemia in hospitalised children and neonates: A prospective cohort study in Nigerian secondary-level hospitals	YES	YES	YES	NO	YES	NO	YES	YES	YES
Graham 2021	Hypoxaemia prevalence and management among children and adults presenting to primary care facilities in Uganda: a prospective cohort study	YES	YES	YES	YES	YES	YES	YES	YES	YES
Graham 2022	Cost-effectiveness and sustainability of improved hospital oxygen systems in Nigeria	UNCLEAR	UNCLEAR	YES	YES	NO	NO	YES	YES	YES
Hau 2018	Post-hospital mortality in children aged 2-12 years in Tanzania: A prospective cohort study.	YES	YES	YES	YES	YES	YES	NOT APPLICABLE	UNCLEAR	YES

Author & Year	Title	Sampling frame	Recruitment	Samp size	Subjects & setting	Missing data	Coverage bias	Diagnostic methods	Measurement	Stat analysis
HILDENWALL, 2008	Bacterial aetiology and outcome in children with severe pneumonia in Uganda	YES	YES	NO	YES	YES	YES	YES	YES	YES
Homayounieh 2020	Clinical and imaging features predict mortality in COVID-19 infection in Iran.	YES	YES	NO	YES	YES	NO	YES	UNCLEAR	YES
HONARVAR 2021	Epidemiological and clinical characteristics of the COVID-19 epidemic and associated factors for mortality in Golestan province, Iran: a retrospective cohort study	YES	YES	YES	YES	YES	YES	YES	UNCLEAR	YES
Hong, 2010	Hospitalized patients with novel influenza A (H1N1) virus infection: Shanghai, June–July 2009	YES	YES	NO	YES	YES	YES	UNCLEAR	YES	YES
Hooli 2020	The Epidemiology of Hypoxemic Pneumonia among Young Infants in Malawi.	YES	YES	YES	YES	YES	NOT APPLICABLE	YES	YES	YES
Hu 2020	Initial diagnosis and management of adult community-acquired pneumonia: A 5-day prospective study in Shanghai	YES	NO	YES	YES	YES	NOT APPLICABLE	YES	UNCLEAR	NO
Husain 2009	Clinical characteristics, management and outcome of major pulmonary embolism: an experience from a tertiary care center in Pakistan.	YES	YES	NO	YES	YES	NOT APPLICABLE	YES	UNCLEAR	YES
Hussain 2005	Trends in hospital-based management of acute asthma from a teaching hospital in South Asia	YES	YES	NO	YES	YES	NOT APPLICABLE	UNCLEAR	UNCLEAR	YES
Ibraheem 2014	Relationship between some risk factors of pneumonia and hypoxaemia in hospitalised Nigerian children	YES	YES	YES	YES	YES	NOT APPLICABLE	YES	YES	YES

Author & Year	Title	Sampling frame	Recruitment	Samp size	Subjects & setting	Missing data	Coverage bias	Diagnostic methods	Measureme nt	Stat analysis
Ibrahima Bah, 2015	Clinical Presentation of Patients with Ebola Virus Disease in Conakry, Guinea	YES	YES	NO	YES	YES	YES	YES	YES	YES
Izadnegahdar 2012	Frequency and trajectory of abnormalities in respiratory rate, temperature and oxygen saturation in severe pneumonia in children	YES	YES	YES	YES	NO	YES	YES	UNCLEAR	YES
Jain 2013	Predictors of treatment failure in hospitalized children [3-59 months] with severe and very severe pneumonia	YES	UNCLEAR	NO	NO	YES	NOT APPLICABLE	YES	UNCLEAR	NO
Jain 2018	Evaluation of cardiac biomarkers in children with acute severe bronchial Asthma-A prospective study from tertiary care center in northern India	YES	UNCLEAR	NO	YES	YES	NOT APPLICABLE	UNCLEAR	UNCLEAR	YES
Jeena 2003	Risk factors for admission and the role of respiratory syncytial virus-specific cytotoxic T-lymphocyte responses in children with acute bronchiolitis	YES	YES	NO	YES	YES	YES	UNCLEAR	UNCLEAR	NO
Jullien 2022	Association of Clinical Signs, Host Biomarkers and Etiology With Radiological Pneumonia in Bhutanese Children	YES	YES	NO	YES	NO	YES	YES	UNCLEAR	YES
Junge 2006	The spectrum of hypoxaemia in children admitted to hospital in The Gambia, West Africa.	YES	YES	YES	YES	YES	UNCLEAR	UNCLEAR	YES	YES
Kapoor 2022	Predicting Mortality and Use of RISC Scoring System in Hospitalized Under-Five Children Due to WHO Defined Severe Community Acquired Pneumonia	YES	YES	NO	YES	YES	NOT APPLICABLE	YES	UNCLEAR	YES
Karim 2021	Epidemiology of Patients with Head Injury at a Tertiary Hospital in Rwanda.	YES	YES	YES	YES	UNCLEAR	YES	YES	UNCLEAR	UNCLEAR
Katagira 2016	Empiric TB Treatment of Severely Ill Patients With HIV and	YES	YES	YES	YES	YES	YES	YES	UNCLEAR	YES

Author & Year	Title	Sampling frame	Recruitment	Samp size	Subjects & setting	Missing data	Coverage bias	Diagnostic methods	Measurement	Stat analysis
	Presumed Pulmonary TB Improves Survival.									
Kaya 2016	Predictors of poor outcome in gastrointestinal bleeding in emergency department.	YES	YES	YES	NO	YES	YES	NO	UNCLEAR	YES
Kayambankadzanja 2021	Unmet need of essential treatments for critical illness in Malawi	YES	YES	YES	YES	YES	YES	YES	YES	YES
KazemiAski 2022	Clinical features and risk factors associated with acute respiratory distress syndrome in pregnant women diagnosed with COVID-19: a multi-center case-control study.	YES	NO	NO	NO	YES	YES	NO	UNCLEAR	YES
Kelly 2015	Treatment Failures and Excess Mortality Among HIV-Exposed, Uninfected Children With Pneumonia	YES	YES	YES	NO	YES	YES	YES	UNCLEAR	YES
King 2022	Prospective cohort study of referred Malawian children and their survival by hypoxaemia and hypoglycaemia status	YES	YES	YES	YES	YES	NOT APPLICABLE	UNCLEAR	YES	YES
Kintwa 2021	Clinical and laboratory features associated with mortality in children with severe malnutrition in Papua New Guinea.	YES	YES	NO	NO	YES	YES	YES	UNCLEAR	YES
Kiputa 2022	Referral challenges and outcomes of neonates received at Muhimbili National Hospital, Dar es Salaam, Tanzania	YES	YES	YES	YES	YES	YES	YES	YES	YES
Koss, 2015	A Clinical Predictor Score for 30-Day Mortality among HIV-Infected Adults Hospitalized with Pneumonia in Uganda	YES	YES	YES	YES	YES	NOT APPLICABLE	UNCLEAR	UNCLEAR	YES
Krebs 2017	Mortality-Associated Characteristics of Patients with Traumatic Brain Injury at the University Teaching Hospital of Kigali, Rwanda	YES	YES	YES	YES	YES	YES	YES	YES	YES

Author & Year	Title	Sampling frame	Recruitment	Samp size	Subjects & setting	Missing data	Coverage bias	Diagnostic methods	Measurement	Stat analysis
Krithika 2022	Effectiveness of admission TOPRS score in predicting the outcome in pediatric inpatients - A prospective observational multicentric study	YES	YES	YES	YES	YES	YES	YES	UNCLEAR	YES
Kuti 2013	Determinants of oxygen therapy in childhood pneumonia in a resource-constrained region	YES	YES	YES	YES	YES	NOT APPLICABLE	YES	YES	YES
Kuti 2013	Risk factors for mortality in childhood pneumonia in a rural West African region	YES	YES	YES	YES	YES	YES	YES	YES	YES
Kwizera 2020	Acute hypoxaemic respiratory failure in a low-income country: A prospective observational study of hospital prevalence and mortality	YES	YES	YES	YES	YES	NOT APPLICABLE	NO	UNCLEAR	YES
Laghari 2019	Therapeutic Role of Zinc Supplementation in Children Hospitalized with Pneumonia	YES	UNCLEAR	NO	YES	YES	NOT APPLICABLE	UNCLEAR	UNCLEAR	YES
Laher 2022	Development and internal validation of the HIV In-hospital Mortality Prediction (HIV-IMP) risk score.	YES	YES	YES	YES	YES	YES	YES	UNCLEAR	YES
Laman 2005	Can clinical signs predict hypoxaemia in Papua New Guinean children with moderate and severe pneumonia?	YES	YES	NO	YES	YES	YES	YES	YES	YES
Landes 2017	Epidemiology, clinical characteristics and outcomes of head injured patients in an Ethiopian emergency centre	YES	YES	YES	YES	YES	NOT APPLICABLE	UNCLEAR	UNCLEAR	YES
Leligdowicz 2021	Risk-stratification of febrile African children at risk of sepsis using sTREM-1 as basis for a rapid triage test	YES	YES	YES	YES	YES	NOT APPLICABLE	UNCLEAR	UNCLEAR	YES
LeRoux 2021	Factors associated with serious outcomes of pneumonia among children in a birth cohort in South Africa.	YES	YES	NO	YES	YES	YES	YES	UNCLEAR	NO

Author & Year	Title	Sampling frame	Recruitment	Samp size	Subjects & setting	Missing data	Coverage bias	Diagnostic methods	Measurement	Stat analysis
Li 2020	Clinical Characteristics of Diabetic Patients with COVID-19.	YES	YES	NO	NO	YES	YES	UNCLEAR	UNCLEAR	UNCLEAR
Libster 2010	Pediatric hospitalizations associated with 2009 pandemic influenza A (H1N1) in Argentina	YES	YES	YES	YES	YES	NOT APPLICABLE	YES	UNCLEAR	YES
Linda 2006	Brief Hospitalization and Pulse Oximetry for Predicting Amoxicillin Treatment Failure in Children with Severe Pneumonia	YES	YES	YES	YES	YES	YES	YES	YES	YES
Ling 2020	Identify clinical factors related to Mycoplasma pneumoniae pneumonia with hypoxia in children	YES	NO	YES	YES	YES	YES	YES	UNCLEAR	YES
Liu 2020	Clinical course and characteristics of patients with coronavirus disease 2019 in Wuhan, China: a single-centered, retrospective, observational study	YES	YES	NO	NO	YES	NOT APPLICABLE	YES	UNCLEAR	YES
Lodha 2004	Can clinical symptoms or signs accurately predict hypoxemia in children with acute lower respiratory tract infections?	YES	YES	NO	YES	YES	NOT APPLICABLE	YES	YES	YES
Lowlaavar 2016	Pediatric in-Hospital Death from Infectious Disease in Uganda: Derivation of Clinical Prediction Models.	YES	YES	YES	YES	YES	NOT APPLICABLE	YES	YES	YES
Ma 2019	Handheld point-of-care lactate measurement at admission predicts mortality in Ugandan children hospitalized with pneumonia: A prospective cohort study	YES	YES	NO	YES	YES	NOT APPLICABLE	YES	UNCLEAR	YES
Magree, 2004	Chest X-ray-confirmed pneumonia in children in Fiji	YES	YES	YES	YES	YES	YES	YES	YES	YES
Maitland 2003	Severe P. falciparum malaria in Kenyan children: evidence for hypovolaemia.	YES	YES	YES	YES	YES	NOT APPLICABLE	UNCLEAR	UNCLEAR	YES

Author & Year	Title	Sampling frame	Recruitment	Samp size	Subjects & setting	Missing data	Coverage bias	Diagnostic methods	Measureme nt	Stat analysis
Maitland 2006	Children with severe malnutrition: can those at highest risk of death be identified with the WHO protocol?.	YES	YES	YES	YES	NO	YES	YES	YES	YES
Marcolino 2021	Clinical characteristics and outcomes of patients hospitalized with COVID-19 in Brazil: Results from the Brazilian COVID-19 registry.	YES	YES	YES	YES	YES	NOT APPLICABLE	YES	UNCLEAR	YES
MarMinn 2021	The comparative ability of commonly used disease severity scores to predict death or a requirement for ICU care in patients hospitalised with possible sepsis in Yangon, Myanmar	YES	YES	YES	YES	YES	NOT APPLICABLE	YES	UNCLEAR	YES
Martinez-Medina 2010	Diagnostic assessment of head nodding in hypoxemia in children with pneumonia	YES	UNCLEAR	NO	YES	YES	NOT APPLICABLE	YES	YES	NO
Mathisen	Clinical presentation and severity of viral community-acquired pneumoniain young Nepalese children	YES	YES	YES	YES	YES	YES	NO	YES	YES
McCollum 2013	Multicenter study of hypoxemia prevalence and quality of oxygen treatment for hospitalized Malawian children	YES	YES	YES	YES	YES	YES	YES	YES	YES
McCollum 2019	Bubble continuous positive airway pressure for children with high-risk conditions and severe pneumonia in Malawi: an open label, randomised, controlled trial.	YES	YES	YES	YES	YES	YES	YES	YES	YES
McCracken 2013	Respiratory syncytial virus infection in Guatemala, 2007-2012	YES	YES	YES	YES	NO	YES	YES	UNCLEAR	YES
Mejia 2020	Oxygen saturation as a predictor of mortality in hospitalized adult	YES	YES	YES	YES	YES	NOT APPLICABLE	YES	UNCLEAR	UNCLEAR

Author & Year	Title	Sampling frame	Recruitment	Samp size	Subjects & setting	Missing data	Coverage bias	Diagnostic methods	Measurement	Stat analysis
	patients with COVID-19 in a public hospital in Lima, Peru.									
Merida-Vieyra 2019	Detection of Mycoplasma pneumoniae in Mexican children with community-acquired pneumonia: Experience in a tertiary care hospital	YES	YES	NO	YES	YES	NOT APPLICABLE	YES	UNCLEAR	YES
Mor 2010	Respiratory cryptosporidiosis in HIV-seronegative children in Uganda: Potential for respiratory transmission	YES	YES	YES	NO	YES	NOT APPLICABLE	YES	UNCLEAR	YES
Morgan 2018	Pulse oximetry values of neonates admitted for care and receiving routine oxygen therapy at a resource-limited hospital in Kenya	YES	UNCLEAR	YES	NO	YES	NOT APPLICABLE	UNCLEAR	YES	YES
Muller 2012	Blood oxygen saturation levels associated with respiratory viruses in children hospitalized with pneumonia in Guatemala, 2008-2011	UNCLEAR	UNCLEAR	YES	UNCLEAR	YES	NOT APPLICABLE	YES	UNCLEAR	YES
Muro 2020		YES	YES	YES	YES	YES	YES	YES	YES	YES
Mvalo 2022	Antibiotic treatment failure in children aged 1 to 59 months with World Health Organization-defined severe pneumonia in Malawi: A CPAP IMPACT trial secondary analysis	YES	YES	YES	YES	YES	YES	YES	UNCLEAR	YES
Mwaniki 2009	Emergency triage assessment for hypoxaemia in neonates and young children in a Kenyan hospital: An observational study	YES	YES	YES	YES	YES	NOT APPLICABLE	YES	YES	YES
Nabukeera-Barungi, 2018	Predictors of mortality among hospitalized childre-n with severe acute malnutrition: a prospective study from Uganda	YES	YES	YES	YES	YES	NOT APPLICABLE	UNCLEAR	UNCLEAR	YES
Navuluri 2023	Prevalence and pheNOTypic trajectories of hypoxaemia among hospitalised adults in	YES	YES	YES	YES	YES	YES	NO	YES	YES

Author & Year	Title	Sampling frame	Recruitment	Samp size	Subjects & setting	Missing data	Coverage bias	Diagnostic methods	Measurement	Stat analysis
	Kenya: a single-centre, prospective cohort study									
Nemani 2016	Malnutrition and anaemia associated with hypoxia among hospitalized children with community-acquired pneumonia in North India	YES	YES	NO	YES	YES	YES	NO	YES	YES
Nemati 2021	Association Between rRT-PCR Test Results Upon Admission and Outcome in Hospitalized Chest CT-Positive COVID-19 Patients: A Provincial Retrospective Cohort with Active Follow-up	YES	YES	YES	YES	YES	NOT APPLICABLE	YES	UNCLEAR	YES
Nielsen 2018	Emergency department risk factors for serious clinical deterioration in a paediatric hospital in Peru	YES	YES	YES	YES	YES	YES	YES	UNCLEAR	YES
Njuguna 2018	Urgent versus post-stabilisation antiretroviral treatment in hospitalised HIV-infected children in Kenya (PUSH): a randomised controlled trial	YES	YES	NO	YES	YES	YES	YES	UNCLEAR	NO
Nyawanda 2016	Evaluation of case definitions to detect respiratory syncytial virus infection in hospitalized children below 5 years in Rural Western Kenya, 2009-2013	YES	YES	YES	YES	YES	YES	YES	UNCLEAR	YES
Ojuawo 2020	Clinical and microbiological profile of adult inpatients with community acquired pneumonia in ilorin, north central, nigeria	YES	YES	NO	YES	NO	NOT APPLICABLE	UNCLEAR	UNCLEAR	YES
Oktaria 2021	Vitamin D deficiency and severity of pneumonia in Indonesian children	YES	YES	NO	YES	YES	NOT APPLICABLE	YES	UNCLEAR	YES
Oliveira 2022	Comparison of the First and Second Waves of the Coronavirus Disease 2019 Pandemic in Children and Adolescents in a Middle-Income	YES	YES	YES	YES	NO	NOT APPLICABLE	NO	UNCLEAR	YES

Author & Year	Title	Sampling frame	Recruitment	Samp size	Subjects & setting	Missing data	Coverage bias	Diagnostic methods	Measurement	Stat analysis
	Country: Clinical Impact Associated with Severe Acute Respiratory Syndrome Coronavirus 2 Gamma Lineage									
Olupot-Olupot 2020	The clinical spectrum of severe childhood malaria in Eastern Uganda	YES	YES	YES	YES	YES	YES	UNCLEAR	UNCLEAR	YES
Ondoa-Onama 2003	Immediate outcome of babies with low Apgar score in Mulago Hospital, Uganda	YES	UNCLEAR	NO	YES	YES	YES	NO	YES	YES
Onubogu 2022	Pulse oximetry and peak expiratory flow rate correlations in acute asthma exacerbation in children	YES	YES	NO	YES	YES	YES	UNCLEAR	YES	YES
Orimadegun 2013	Prevalence and predictors of hypoxaemia in respiratory and non-respiratory primary diagnoses among emergently ill children at a tertiary hospital in south western Nigeria	YES	YES	YES	YES	YES	YES	YES	YES	YES
Orimadegun 2014	Hypoxemia predicts death from severe falciparum malaria among children under 5 years of age in Nigeria: the need for pulse oximetry in case management	YES	YES	YES	YES	YES	YES	YES	YES	YES
Padmaprakash 2021	Clinical characteristics and clinical predictors of mortality in hospitalised patients of COVID 19: An Indian study	YES	YES	YES	YES	YES	YES	YES	UNCLEAR	YES
Papali 2017	Treatment outcomes after implementation of an adapted WHO protocol for severe sepsis and septic shock in Haiti	YES	YES	NO	YES	YES	NOT APPLICABLE	YES	UNCLEAR	YES
Patel 2021	Multicenter epidemiologic study of coronavirus disease-associated mucormycosis, India	YES	YES	YES	YES	YES	UNCLEAR	UNCLEAR	UNCLEAR	YES
Pati, 2013	Clinical Presentation of Patients with Seasonal Influenza and	YES	YES	NO	YES	UNCLEAR	YES	UNCLEAR	UNCLEAR	YES

Author & Year	Title	Sampling frame	Recruitment	Samp size	Subjects & setting	Missing data	Coverage bias	Diagnostic methods	Measurement	Stat analysis
	Pandemic Influenza A (H1N1-2009) Requiring Hospitalisation									
PneumoniaEtiologyResearchforChildHealth(PERCH)StudyGroup 2019	Causes of severe pneumonia requiring hospital admission in childrenwithout HIV infection from Africa and Asia: the PERCH multi-countrycase-control study	YES	YES	YES	YES	YES	NOT APPLICABLE	YES	UNCLEAR	YES
Pukai 2020	Nebulised normal saline in moderate acute bronchiolitis and pneumonia in a low- to middle-income country: a randomised trial in Papua New Guinea	YES	YES	NO	YES	YES	YES	YES	UNCLEAR	YES
Puumalainen 2008	Clinical case review: a method to improve identification of true clinical and radiographic pneumonia in children meeting the World Health Organization definition for pneumonia	YES	YES	YES	YES	YES	YES	YES	UNCLEAR	YES
Rabha 2020	CLINICAL MANIFESTATIONS OF CHILDREN AND ADOLESCENTS WITH COVID-19: REPORT OF THE FIRST 115 CASES FROM SABARA HOSPITAL INFANTIL.	YES	YES	NO	YES	YES	YES	NO	UNCLEAR	NO
Rahman 2021	Hypoxaemia prevalence and its adverse clinical outcomes among children hospitalised with WHO-defined severe pneumonia in Bangladesh.	YES	YES	YES	YES	YES	YES	YES	YES	YES
Rahnama&#039 2006	Which clinical signs and symptoms predict hypoxemia in acute childhood asthma?	YES	NO	NO	YES	YES	NOT APPLICABLE	YES	YES	YES
Raihana 2015	Development and Internal Validation of a Predictive Model Including Pulse Oximetry for Hospitalization of Under-Five Children in Bangladesh.	YES	YES	YES	YES	YES	NOT APPLICABLE	YES	YES	YES
Rajesh 2000	Tachypnoea is a good predictor of hypoxia in acutely ill infants under 2 months	YES	YES	NOT APPLICABLE	YES	YES	NOT APPLICABLE	YES	YES	YES

Author & Year	Title	Sampling frame	Recruitment	Samp size	Subjects & setting	Missing data	Coverage bias	Diagnostic methods	Measurement	Stat analysis
Ramakrishna 2012	Lactate as a predictor of mortality in Malawian children with WHO-defined pneumonia.	YES	YES	YES	YES	YES	YES	YES	UNCLEAR	YES
Ramatillah 2022	Impact of cytokine storm on severity of COVID-19 disease in a private hospital in West Jakarta prior to vaccination.	UNCLEAR	NO	NO	NO	YES	YES	YES	UNCLEAR	YES
Rao 2012	Clinical predictors of hypoxemia in Indian children with acute respiratory tract infection presenting to pediatric emergency department	YES	YES	YES	YES	YES	YES	YES	UNCLEAR	YES
Rao 2021	Outcome of Children Admitted With SARS-CoV-2 Infection: Experiences From a Pediatric Public Hospital.	YES	YES	NO	YES	YES	YES	YES	UNCLEAR	YES
Riccetto 2006	Respiratory Syncytial Virus (RSV) in infants hospitalized for acute lower respiratory tract disease: Incidence and associated risks	YES	YES	NO	YES	YES	YES	YES	UNCLEAR	YES
Riviello 2016	Hospital Incidence and Outcomes of the Acute Respiratory Distress Syndrome Using the Kigali Modification of the Berlin Definition	YES	YES	YES	YES	YES	YES	YES	YES	YES
Rudd 2014	Sepsis presentation, management, and outcomes in adults and children admitted to a rural ugandan hospital: A prospective observational cohort study	YES	NO	NO	YES	YES	YES	YES	UNCLEAR	YES
Salah 2014	The use of vital signs as predictors for serious bacterial infections in children with acute febrile illness in a pediatric emergency setting in Sudan.	YES	YES	NO	YES	YES	YES	YES	UNCLEAR	YES
Salah 2015	Prevalence of hypoxemia in under-five children with	YES	YES	NO	YES	YES	UNCLEAR	YES	YES	YES

Author & Year	Title	Sampling frame	Recruitment	Samp size	Subjects & setting	Missing data	Coverage bias	Diagnostic methods	Measureme nt	Stat analysis
	pneumonia in an emergency pediatrics hospital in Sudan									
Saleh 2022	Surfactant protein D: a predictor for severity of community-acquired pneumonia in children	YES	YES	NO	YES	YES	YES	YES	UNCLEAR	YES
Sampathkumar 2021	Incidence, Risk Factors and Outcome of COVID-19 Associated AKI- A Study from South India	YES	YES	NO	YES	YES	YES	UNCLEAR	UNCLEAR	YES
Santhanam, 2008	A Prospective Randomized Controlled Study of Two Fluid Regimens in the Initial Management of Septic Shock in the Emergency Department	YES	NO	NO	YES	UNCLEAR	UNCLEAR	UNCLEAR	UNCLEAR	YES
Sarfaraz 2021	Determinants of in-hospital mortality in COVID-19; a prospective cohort study from Pakistan.	YES	YES	NO	YES	YES	YES	YES	UNCLEAR	YES
Sempertegui 2014		YES	YES	YES	YES	YES	YES	YES	YES	YES
Shahid 2016	Factors Associated with Streptococcal Bacteremia in Diarrheal Children under Five Years of Age and Their Outcome in an Urban Hospital in Bangladesh.	YES	YES	NO	YES	YES	YES	YES	UNCLEAR	YES
Shahrin 2020	Clinical and laboratory predictors of 30-day mortality in severe acute malnourished children with severe pneumonia	YES	YES	NO	YES	YES	NOT APPLICABLE	YES	UNCLEAR	YES
Shahunja 2020	Clinical and laboratory characteristics of children under five hospitalized with diarrhea and bacteremia	YES	YES	YES	YES	YES	YES	YES	UNCLEAR	YES
Sharawat 2021	High resolution computed tomography of thorax- is it an early predictor of hypoxaemia in covid-19 patients?	YES	YES	YES	NO	YES	YES	NO	UNCLEAR	YES
Sigauque 2009		YES	YES	YES	YES	YES	YES	YES	YES	YES

Author & Year	Title	Sampling frame	Recruitment	Samp size	Subjects & setting	Missing data	Coverage bias	Diagnostic methods	Measurement	Stat analysis
Siqueira 2022	Clinical characteristics and risk factors for maternal deaths due to COVID-19 in Brazil: a nationwide population-based cohort study	YES	YES	YES	YES	YES	NOT APPLICABLE	YES	UNCLEAR	YES
Sirohiya 2022	Silent Hypoxia in Coronavirus disease-2019: Is it more dangerous? -Aretrospective cohort study	YES	YES	YES	YES	YES	UNCLEAR	NO	UNCLEAR	YES
Smith 2011		YES	YES	YES	YES	YES	YES	YES	YES	YES
Snouber 2022	Clinical and computed tomography features of patients suspected of COVID-19 in the university hospital of Oran, Algeria	UNCLEAR	YES	NO	NO	YES	NOT APPLICABLE	YES	UNCLEAR	NO
Soto 2022	Mortality and associated risk factors in patients hospitalized due to COVID-19 in a Peruvian reference hospital.	YES	YES	YES	YES	YES	NOT APPLICABLE	NO	UNCLEAR	YES
Srinivasan 2012	Zinc adjunct therapy reduces case fatality in severe childhood pneumonia: a randomized double blind placebo-controlled trial.	YES	YES	YES	YES	YES	YES	NO	YES	YES
Stassen 2014	The prevalence of hypotension and hypoxaemia in blunt traumatic brain injury in the prehospital setting of Johannesburg, South Africa: A retrospective chart review.	YES	YES	NO	NO	YES	NOT APPLICABLE	YES	UNCLEAR	YES
Sultan 2021	Clinical Characteristics of COVID-19 Related Deaths in Ethiopia.	UNCLEAR	NO	NO	NO	YES	YES	UNCLEAR	UNCLEAR	NO
Sutherland 2016	Oxygen as an Essential Medicine: Under- and Over-Treatment of Hypoxemia in Low- and High-Income Nations	YES	YES	YES	NO	YES	YES	YES	YES	YES

Author & Year	Title	Sampling frame	Recruitment	Samp size	Subjects & setting	Missing data	Coverage bias	Diagnostic methods	Measurement	Stat analysis
Sutherland 2019	The "Just Right" amount of oxygen improving oxygen use in a Rwandan emergency department	YES	YES	YES	YES	YES	NOT APPLICABLE	YES	YES	YES
Sylvies, 2020	The impact of pulse oximetry and Integrated Management of Childhood Illness (IMCI) training on antibiotic prescribing practices in rural Malawi: A mixed-methods study	YES	YES	YES	YES	YES	YES	YES	YES	YES
Tesfaye 2020	Pulse oximeter with integrated management of childhood illness for diagnosis of severe childhood pneumonia at rural health institutions in Southern Ethiopia: Results from a cluster-randomised controlled trial	YES	YES	YES	YES	YES	YES	YES	YES	YES
Thomas 2015	Disease mapping for informing targeted health interventions: childhood pneumonia in Bohol, Philippines	YES	YES	YES	YES	UNCLEAR	YES	YES	YES	YES
Tiewsoh 2009	Factors determining the outcome of children hospitalized with severe pneumonia	YES	YES	YES	YES	YES	YES	YES	UNCLEAR	YES
Tokman 2014	Procalcitonin predicts mortality in HIV-infected Ugandan adults with lower respiratory tract infections	YES	UNCLEAR	YES	YES	YES	NOT APPLICABLE	UNCLEAR	UNCLEAR	YES
Tolossa 2022	Acute respiratory distress syndrome among patients with severe COVID-19 admitted to treatment center of Wollega University Referral Hospital, Western Ethiopia	YES	YES	YES	YES	YES	NOT APPLICABLE	NO	UNCLEAR	YES
Tran, 2016	Clinical and epidemiological characteristics of acute respiratory virus infections in Vietnamese children	YES	YES	YES	YES	YES	YES	UNCLEAR	UNCLEAR	YES
Tuti 2021	Pulse oximetry adoption and oxygen orders at paediatric	YES	YES	YES	YES	NO	UNCLEAR	YES	YES	NO

Author & Year	Title	Sampling frame	Recruitment	Samp size	Subjects & setting	Missing data	Coverage bias	Diagnostic methods	Measurement	Stat analysis
	admission over 7 years in Kenya: A multihospital retrospective cohort study									
Usen 1999	Clinical predictors of hypoxaemia in Gambian children with acute lower respiratory tract infection: Prospective cohort study	YES	YES	YES	YES	YES	YES	YES	YES	YES
VafadarMoradi 2021	Increased age, neutrophil-to-lymphocyte ratio (NLR) and white blood cells count are associated with higher COVID-19 mortality.	YES	YES	YES	NO	YES	NOT APPLICABLE	YES	UNCLEAR	YES
vonderWeid 2018	Clinical signs of hypoxaemia in children aged 2 months to 5 years with acute respiratory distress in Switzerland and Senegal	YES	YES	NO	YES	YES	YES	YES	UNCLEAR	YES
Vongchaiudomchoke 2016	Positive pulmonary computed tomography angiography in patients with suspected acute pulmonary embolism: Clinical prediction rules, thromboembolic risk factors, and implications for appropriate use	YES	YES	YES	YES	YES	NOT APPLICABLE	YES	UNCLEAR	UNCLEAR
Wandi 2006	Hypoxaemia among children in rural hospitals in Papua New Guinea: Epidemiology and resource availability - A study to support a national oxygen programme	YES	YES	YES	YES	YES	YES	YES	YES	YES
Wasingya-Kasereka 2020	Oxygen use in low-resource settings: An intervention still triggered by intuition.	YES	YES	YES	YES	YES	NOT APPLICABLE	YES	UNCLEAR	YES
Webb 2012		YES	YES	YES	YES	YES	YES	YES	YES	YES
Weber 2002	Clinical predictors of bacterial meningitis in infants and young children in The Gambia.	YES	YES	YES	YES	YES	YES	YES	UNCLEAR	YES

Author & Year	Title	Sampling frame	Recruitment	Samp size	Subjects & setting	Missing data	Coverage bias	Diagnostic methods	Measurement	Stat analysis
Weber 2003	Predictors of neonatal sepsis in developing countries	YES	YES	YES	YES	YES	NOT APPLICABLE	YES	UNCLEAR	NO
West 1999	Long-term morbidity and mortality following hypoxaemic lower respiratory tract infection in Gambian children.	YES	YES	NO	YES	YES	YES	YES	UNCLEAR	YES
Wollenstein-Betech, 2020	Physiological and socioeconomic characteristics predict COVID-19 mortality and resource utilization in Brazil	YES	YES	YES	YES	YES	NOT APPLICABLE	UNCLEAR	UNCLEAR	YES
Worodria 2018	Predictors of Mortality Among Hospitalized Patients With Lower Respiratory Tract Infections in a High HIV Burden Setting.	YES	YES	YES	YES	YES	YES	YES	UNCLEAR	YES
Xiong 2021	Association of consciousness impairment and mortality in people with COVID-19.	YES	YES	YES	NO	YES	YES	YES	UNCLEAR	YES
Yahia 2012	Human metapneumovirus (hMPV) in acute respiratory infection: A clinic-based study in Egypt	UNCLEAR	NO	YES	YES	UNCLEAR	UNCLEAR	UNCLEAR	UNCLEAR	YES
YalÃ§Ä±n 2018	Agreement Between Integrated Management of Childhood Illness and Final Diagnosis in Acute Respiratory Tract Infections	YES	YES	YES	YES	YES	YES	NO	UNCLEAR	YES
YeLynn 2019	The clinical characteristics of patients with sepsis in a tertiary referral hospital in Yangon, Myanmar	YES	YES	NO	YES	YES	NOT APPLICABLE	YES	UNCLEAR	YES
Zampoli 2017	Adenovirus-associated pneumonia in South African children: Presentation, clinical course and outcome	YES	YES	YES	YES	YES	YES	YES	UNCLEAR	YES
Zar 2022	Klebsiella pneumoniae Lower Respiratory Tract Infection in a South African Birth Cohort: a Longitudinal Study	YES	YES	YES	YES	YES	YES	UNCLEAR	UNCLEAR	NO

Author & Year	Title	Sampling frame	Recruitment	Samp size	Subjects & setting	Missing data	Coverage bias	Diagnostic methods	Measureme nt	Stat analysis
Zhou, 2020	Predictive factors of severe coronavirus disease 2019 in previously healthy young adults: a single-center, retrospective study	YES	YES	NO	YES	YES	YES	YES	YES	YES

Text S3: Assessment of Certainty

Assessment of certainty of meta-estimates is uncommonly done in systematic reviews and meta-analysis of prevalence. Usual tools, such as the GRADE approach, are primarily intended for assessing the certainty of effect sizes from synthesis of interventional studies. While GRADE does have adaptations for non-interventional effect topics, such as prognosis or diagnosis, there are no guidance on assessment of certainty for questions of prevalence. A recent review of systematic reviews of prevalence, authors reported that only 9/235 (3.8%) studies included a formal quality assessment or process to establish certainty of the entire body of evidence (*Migliavaca et al., BMC Medical Research Methodology 2020*). In the absence of clear guidance, we used the five items from GRADE, informed by recommendations from studies applying this to prognosis and diagnosis (*Iorio et al. BMJ 2015; GRADE Handbook 2013*).

- the risk of bias
- the precision of the estimates
- the consistency of the individual study results
- how directly the evidence answers the question of interest
- the risk of publication or reporting biases.

We applied these to each meta-estimate according to the explanations in the below table. We downgraded the overall certainty score for each item that scored low. The certainty ratings were given an overall rating of High, Moderate, Low, or Very Low accordingly.

**Factors that determine and can decrease the quality of evidence**	**Explanations of how we applied it to our prevalence study**
Risk of bias (limitations in study design and execution)	Different criteria for prevalence studies. Cross-sectional or cohort studies in representative patient populations with appropriate assessment are considered high quality and can move to moderate, low or very low depending on other factors.<br><br>We assessed risk of bias using JBI tool for prevalence studies (see Risk of bias assessment section for detail), then determined:<br>• Representativeness of the population that was intended to be sampled (sampling frame, recruitment, missing, coverage)<br>• Validity of diagnostic assessment (method for measurement)<br>• Adequacy of the data (statistical analysis)
Precision of the estimates	Wide confidence intervals for estimates of prevalence can lower the quality of evidence.
Consistency in study results	Unexplained inconsistency in prevalence estimates can lower the quality of evidence.<br><br>For our hypoxaemia prevalence study, relevant explanations include things such as altitude, population characteristics (e.g. age, condition), and participant recruitment or selection process. NB: In prevalence studies, I^2 for the pooled estimate can be misleading (e.g. extremely high I^2 with modest inconsistency between studies) and the extent of variation in point estimates is far more important.[12]

Directness of evidence to the question	The quality of evidence can be lowered if there are important differences between the populations studied and those for whom the recommendation is intended or if the method for assessing prevalence is different to that which is intended.<br><br>For our hypoxaemia prevalence study, we only included studies that used pulse oximetry so the main directness consideration was related to whether the included population fitted with one of our designated populations of interest.
Risk of Publication or Reporting bias	A high risk of publication bias (e.g., evidence only from small studies supporting a new hypoxaemia intervention, or asymmetry in a funnel plot) can lower the quality of evidence.<br><br>For our hypoxaemia prevalence study, the risk of publication or reporting bias was considered low.
Upgrading for dose effect, large effects	Methods have not been properly developed for upgrading for large effects in prevalence (or prognostic) studies. We did not apply upgrading to our assessment of certainty.[12]

Table S5: Full list of included studies

Study	Country	WB Cat	Context	Altitude (meters)	Level	Setting	Design	Condition	Age range	Hypox definition	N	Hypoxaemia (n/N)
AbdelGhaffar 2022	Egypt	LMIC	Urban	321	Tertiary	Inpatient	Cross sectional study	COVID 19	0 - 90 years	SpO2<95%	3752	Child Pneum (other): 1051/3172 ; Child COVID-19: 1051/3172 ; Adult COVID-19: 1051/3712; Hypoxemia Death: 561; Hypoxemia Survive: 490; Normoxemia Death: 339; Normoxemia Survival: 2322; Death OR: 7.84
Abdulkadir 2015	Nigeria	LMIC	Urban	313	Tertiary	Emergenc y	Cross sectional study	Pneumonia	2-59 months	SpO2<90%	200	Child All Pneum (WHO): 83/200; Hypoxemia_Death: 17; Hypoxemia_Survive: 66; Normoxemia_Death:0; Normoxemia_Survival: 117
Acar 2021	Turkey	UMIC	Unclear	1141	Tertiary	Inpatient	Cohort Study	COVID 19	19 - 75 years	SpO2 <95%	709	Missing adult: 38; Adult COVID_19: 280/671; Hypoxemia_Death:57; Hypoxemia_Survive: 223; Normoxemia_Death:16; Normoxemia_Survival: 375;
Addo-Yobo 2004	Other: Colombia, Ghana, India, Mexico, Pakistan, South Africa, Vietnam, Zambia	Mixed	Unclear	8 (Durban) - 2640 (Bogota)	Tertiary	Inpatient	Randomised controlled trial	Pneumonia	3 - 59 months	Spo2< 90%	1702	Missing child: 8; Child All Pneum (WHO) : 325/1702; Child Pneum (WHO-severe): 325/1702
Agrawal 2011	Other: Nepal	LMIC	Urban	1400	Tertiary	Mixed	Cross sectional study	All patients	0- 2 months	SpO2<90%	160	All neon: 82; All neonate hypoxaemia: 27; All child: 34/78
Ahmed 2022	Uganda	LIC	Unclear	1331	Secondary	Inpatient	Cohort Study	Pneumonia	<13 years	SpO2<90%	77	All Child Pneum(WHO): 54/77
Alizadehsani 2022	Iran	LMIC	Unclear	1305	Tertiary	Inpatient	Cohort Study	COVID 19	17- 100 years	SpO2<93%	660	Adult COVID_19: 400/600; Hypoxemia_Death:85; Hypoxemia_Survive: 315, Normoxemia_Death: 8; Normoxemia_Survival: 192; Death OR :6.429

Study	Country	WB Cat	Context	Altitude (meters)	Level	Setting	Design	Condition	Age range	Hypox definition	N	Hypoxaemia (n/N)
Al Janabi 2009	Iraq	LMIC	Unclear	312	Tertiary	Emergency	Case Control	ARI	2-59 months	SpO2<90%	104	Child:<br>- Pneum (WHO classified): 7/104
Alva 2022	Peru	UMIC	Unclear	3827	Secondary	Inpatient	Cohort Study	COVID 19	40-60 years	SpO2 <85%	348	Missing Adult : 2;<br>Adult COVID_19: 104/348
Alwadhi 2017	India	LMIC	Urban	621	Tertiary	Inpatient	Cross sectional study	Pneumonia	2 months to 5 years	SpO2<90%	112	All Child Pneum(WHO): 57/112; Child Pneum (WHO- severe): 57/112; Child Pneum (radiological): 50/93;<br>Hypoxemia_Death:4 :<br>Hypoxemia_Survive: 53;<br>Normoxemia_Death: 0;<br>Normoxemia_Survival: 55
Amare 2008	Ethiopia	LIC	Urban	2355	Tertiary	Inpatient	Cross sectional study	Status epilepticus	14-75 years	SpO2<90%	119	Adult Seizures: 30/119;<br>Hypoxemia_Death:7;<br>Hypoxemia_Survive:23;<br>Normoxemia_Death:17,<br>Normoxemia_Survival: 72;<br>Death OR: 1.29
Anyaypoma-Ocon 2021	Peru	UMIC	Unclear	1555	Tertiary	Inpatient	Cohort Study	COVID 19	>= 14 years	SpO2<90%	225	Adult COVID_19: 164/225;<br>Hypoxemia_Death: 79;<br>Hypoxemia_Survive: 85;<br>Normoxemia_Death:73;<br>Normoxemia_Survival: 87;<br>Death OR:1.2
Araban 2022	Iran	LMIC	Unclear	1305	Mixed	Mixed	Cross sectional study	COVID19	0 - 80 years	SpO2< 93%	3181	Adult COVID_19: 1228/3181;<br>Hypoxemia_Death: 228;<br>Hypoxemia_Survive: 1000;<br>Normoxemia_Death: 82,<br>Normoxemia_Survival: 1871:<br>Death OR: 5.18
Arana-Calderon 2022	Peru	UMIC	Unclear	1555	Secondary	Mixed	Cohort Study	Pneumonia	Not reported	SpO2<90%	158	Adult Pneum: 143/158;<br>Hypoxemia_Death: 94;<br>Hypoxemia_Survive: 49,<br>Normoxemia_Death:1 ;<br>Normoxemia_Survival: 14;<br>Death OR: 27.3
Asghar 2008	Other: Bangladesh, Ecuador, India, Mexico, Pakistan,	Mixed	Urban	Bangladesh (Dhaka) - 4, Ecuador (Guayaquil) - 4, India (Chandigarh) - 321, Mexico	Tertiary	Inpatient	Randomised controlled trial	Pneumonia	2-59 months	SpO2 <90%	958	All Child Pneum(WHO): 619/ 958;<br>Child Pneum (WHO- severe): 619/ 958;<br>Hypoxemia_Death: 53,<br>Hypoxemia_Survive: 566;<br>Normoxemia_Death: 12;<br>Normoxemia_Survival: 327;<br>Death OR: 2.55

Study	Country	WB Cat	Context	Altitude (meters)	Level	Setting	Design	Condition	Age range	Hypox definition	N	Hypoxaemia (n/N)
	Yemen, Zambia			(Mexico City 2240), Pakistan (Multan) - 122 & (Rawalpindi) - 508, Yemen (Sana'a) - 2250, and Zambia (Lusaka) - 1,279								
Ashraf 2010	Bangladesh	LMIC	Urban	4	Mixed	Mixed	Randomised controlled trial	Pneumonia	2-59 months	SpO2<95%	360	All Child Pneum(WHO): 189/360; Child Pneum (WHO- severe): 189/360; Hypoxemia_Death: 0; Hypoxemia_Survive: 189: Normoxemia_Death: 2, Normoxemia_Survival: 169;
Ashraf 2008	Bangladesh	LMIC	Urban	4	Primary	Outpatient	Cohort Study	Pneumonia	2-59 months	SpO2 <95%	251	All Child Pneum(WHO): 143/251; Child Pneum (WHO- severe): 143/251
Ashraf 2019	Bangladesh	LMIC	Urban	4	Mixed	Mixed	Randomised controlled trial	Pneumonia and malnutrition	2-59 months	SpO2<90%	470	All Child Pneum(WHO): 50/470; Child Pneum (WHO- severe): 50/470; Child Maln 50/470
Aslam 2021	Pakistan	LMIC	Urban	150	Tertiary	Inpatient	Cross sectional study	Multiple lung diseases	20-70 years	SpO2<90%	290	Adult hypoxaemia: 44/290
Aston 2019	Malawi	LIC	Unclear	779	Tertiary	Inpatient	Cross sectional study	Pneumonia	>= 18 years	SpO2<90%	459	Missing Adult: 9; Adult Pneum: 73/450; Hypoxemia_Death: 27; Hypoxemia_Survive: 45; Normoxemia_Death: 36; Normoxemia_Survival: 323; Death OR:.516
Awasthi 2022	India	LMIC	Unclear	621	Secondary	Inpatient	Cohort Study	Pneumonia	2-59 months	SpO2<90%	7196	All Child Pneum(WHO): 2580/7196; Hypoxemia_Death: 45; Hypoxemia_Survive: 2535; Normoxemia_Death: 34; Normoxemia_Survival: 4592; Death OR: 0.03
Bah 2015	Other: Guinea	LIC	Urban	30	Mixed	Inpatient	Observational study	Ebola virus	28 - 46 years	SpO2<90%	37	Overall: 1/37

Study	Country	WB Cat	Context	Altitude (meters)	Level	Setting	Design	Condition	Age range	Hypox definition	N	Hypoxaemia (n/N)
Barennes 2016	Other: Laos PDR	LMIC	Urban	172	Tertiary	Inpatient	Cohort Study	All patients	1 month to 15 years	SpO2<90%	350	All child: 48/350; Child hypoxaemia: 48; Hypoxemia_Death: 8; Hypoxemia_Survive: 40; Normoxemia_Death: 13; Normoxemia_Survival: 289; Death OR: 4.446
Basnet 2015	Other: Nepal	LMIC	Unclear	1400	Tertiary	Inpatient	Clinical Trial	Pneumonia	2- 35 months	SpO2<90%	598	All Child Pneum(WHO): 373/610; Child Pneum (WHO- severe): 373/610
Basnet 2006	Other: Nepal	LMIC	Urban	1336	Tertiary	Mixed	Cross sectional study	ARI	2 - 59 months	SpO2<90%	250	All Child Pneum(WHO): 58/150; Child Pneum (WHO- severe): 40/45; Child Pneum (WHO- nonsevere): 18/105
Bassat 2016	Other: Mozambique	UMIC	Rural	50	Secondary	Inpatient	Cross sectional study	Pneumonia	1-59 months	SpO2<90%	825	Child Fever: 207/746; All Child Pneum(WHO): 230/825; Child Pneum (WHO- severe): 230/825; Child Pneum (WHO- non-severe): 230/825; Child Pneum (radiological): 117/319; Hypoxemia_Death: 40, Hypoxemia_Survive: 190; Normoxemia_Death: 39; Normoxemia_Survival: 556; Death OR: 3.001
Benet 2017	Other: India, Madagascar, Mali, Paraguay	Mixed	Mixed	464	Tertiary	Inpatient	Cohort Study	Pneumonia	2 - 60 months	SpO2<90%	464	Missing Child : 59; Child Pneum (radiological): 70/405; Hypoxemia_Death: 6; Hypoxemia_Survive: 64; Normoxemia_Death: 8; Normoxemia_Survival: 327; Death OR: 3.83
Bepouka 2020	DR Congo	LIC	Urban	726	Tertiary	Mixed	Cohort Study	COVID 19	40-60 years	SpO2 <96%	141	Adult COVID_19: 95/141 ; Hypoxemia_Death: 39, Hypoxemia_Survive: 56; Normoxemia_Death: 2; Normoxemia_Survival: 44; Death OR : 15.312
Bhargava 2016	India	LMIC	Mixed	566	Tertiary	Emergency	Cohort Study	Scrub typhus	>= 18 years	SpO2<90%	284	Overall: 52/284
Bills 2020	India	LMIC	Mixed	160	Primary	Emergency	Cohort Study	ARI	0-17 years	SpO2 <95%	1433	All Child Pneum(WHO): 380/ 1173; Death OR: 6.03
Blanc 2019	Other: Papua New Guinea	LMIC	Rural	42	Primary	Outpatient	Cohort Study	All patients	3-27 months	SpO2<94%	1663 unique illness	All child: 1663, child hypoxaemia: 23

Study	Country	WB Cat	Context	Altitude (meters)	Level	Setting	Design	Condition	Age range	Hypox definition	N	Hypoxaemia (n/N)
											episodes (643 patients)	
Boonmee 2020	Thailand	UMIC	Urban	287	Tertiary	Emergency	Cross sectional study	Sepsis	>18 years	unclear	1616	Adult Seps: 439/1616; Hypoxemia_Death: 177; Hypoxemia_Survive: 262; Normoxemia_Death: 279; Normoxemia_Survival: 898; Death OR: 2.16
Boyce 2017	Uganda	LIC	Rural	1232	Primary	Outpatient	Cohort Study	Malaria	<12 years	SpO2 <92%	1248	Child Mal: 121/1248
Breiman 2015	Kenya	LMIC	Urban	762	Primary	Inpatient	Cohort Study	Pneumonia	< 5 years	SpO2<90%	Total: 2592 Nasal swab collected: 815 Not collected: 1777	All Child Pneum(WHO): 916/2563; Child Pneum (WHO- severe): 916/2563;
Bruce 2007	Other: Guatemala	UMIC	Rural	2600	Primary	Other - Community (household)	Randomised controlled trial	Pneumonia	<18 months	SpO2 < 87%	263	All Child Pneum(WHO): 136/263; Child Pneum(radiological): 55/88
Bui-Binh-Bao 2021	Vietnam	LMIC	Unclear	398	Tertiary	Inpatient	Cohort Study	Pneumonia	2-59 months	SpO2<90%	281	All Child Pneum(WHO): 32/281; Hypoxemia_Death: 7; Hypoxemia_Survive: 25; Normoxemia_Death: 1; Normoxemia_Survival: 248; Death OR: 70
Caio-SimoesSOUZA 2014	Brazil	UMIC	Urban	1172	Tertiary	Inpatient	Cohort Study	Pulmonary embolism	17-92 years	SpO2<90%	102	Overall: 17/102
Carugati 2018	Tanzania	LMIC	Urban	880	Tertiary	Emergency	Cohort Study	Febrile illness	>9 years	SpO2<90%	419	Adult fever: 41/419 ; Adult HIV: 24/160; Hypoxemia_Death: 11; Hypoxemia_Survive: 30; Normoxemia_Death: 33;

Study	Country	WB Cat	Context	Altitude (meters)	Level	Setting	Design	Condition	Age range	Hypox definition	N	Hypoxaemia (n/N)
												Normoxemia_Survival: 354;<br>Death OR: 3.8
Chan 2002	Other: Malaysia	UMIC	Urban	40	Tertiary	Inpatient	Cohort Study	Bronchiolitis	0-2 years	SpO2<90%	216	Child Bronch: 31/216
Chaudhary 2020	Other: Nepal	LMIC	Urban	1400	Tertiary	Mixed	Cross sectional study	Pneumonia	3-60 months	SpO2<90%	160	All Child Pneum(WHO): 93/160 ;<br>Child Pneum(radiological):: 65/ 97
Chinawa 2013	Nigeria	LMIC	Mixed	253	Tertiary	Mixed	Cross sectional study	Anaemia	6 months to 18 years	SpO2<90%	42	Child Anaem: 16/ 88
Chisti 2021	Banglades h	LMIC	Urban	85	Tertiary	Inpatient	Cohort Study	Pneumonia	<5 years	SpO2<90%	4007	All Child Pneum(WHO): 1127/4007;<br>Hypoxemia_Death: 153;<br>Hypoxemia_Survive: 974;<br>Normoxemia_Death: 33;<br>Normoxemia_Survival: 2847:<br>Death OR: 13.55
Chisti 2022	Banglades h	LMIC	Urban	85	Tertiary	Inpatient	Cross sectional study	Pneumonia	0-59 months	SpO2<90%	3468<br><br>anae mic: 1712<br>non anae mic: 1756	All Child Pneum(WHO): 1083/3468;<br>Hypoxemia_Death: 165;<br>Hypoxemia_Survive: 918;<br>Normoxemia_Death: 29,<br>Normoxemia_Survival: 2356:<br>Death OR: 18
Chisti 2013	Banglades h	LMIC	Urban	4	Tertiary	Inpatient	Case control study	Pneumonia	0-59 months	SpO2<90%	334	All Child Pneum(radilogical): 37/334;<br>Child Maln: 37/334;<br>Hypoxemia_Death: 14;<br>Hypoxemia_Survive: 3;<br>Normoxemia_Death: 21;<br>Normoxemia_Survival: 102;<br>Death OR: 22.66667
Chisti 2011	Banglades h	LMIC	Urban	4	Tertiary	Inpatient	Cohort Study	Diarrhoea	0 - 59 months	SpO2<90%	258	All Child Pneum(WHO): 108/198;<br>Child Diarrhoea: 11/60;<br>Hypoxemia_Death: 25;<br>Hypoxemia_Survive: 94;<br>Normoxemia_Death :4 ;<br>Normoxemia_Survival: 135;<br>Death OR: 8.98
Cserti-Gazdewich 2013	Uganda	LIC	Urban	1200	Tertiary	Inpatient	Cohort Study	Malaria	6 months to 12 years	SpO2<90%	1933	Child Mal: 43/1901;<br>Hypoxemia_Death: 10;<br>Hypoxemia_Survive: 33;<br>Normoxemia_Death: 48;

Study	Country	WB Cat	Context	Altitude (meters)	Level	Setting	Design	Condition	Age range	Hypox definition	N	Hypoxaemia (n/N)
												Normoxemia_Survival: 758;<br>Death OR: 4.785353
Davis 2010	Uganda	LIC	Urban	1200	Tertiary	Inpatient	Cohort Study	HIV	Not reported	SpO2 <93%	202	Adult Pneum: 53/202;<br>Adult HIV 53/202;<br>Adult Tuber: 19/72
deJesus 2022	Brazil	UMIC	Mixed	320	Mixed	Inpatient	Cross sectional study	COVID 19	>= 18 years	SpO2 < 95%	29369	Adult missing: 3077;<br>Adult COVID_19: 21987/ 26292
Dembele 2019	Philippines	LMIC	Mixed	4	Mixed	Inpatient	Cohort Study	Pneumonia	8 days to 59 months	SpO2<90%	5054	All neon: 654/5023<br>Missing neon: 5<br>Missing child: 26<br>-Pneum 89/749<br>-WHO Pneum 565/4179<br>-WHO severe or very severe 565/4179<br>Hypoxemia death 85<br>Hypoxemia survive 569<br>Normxemia death 152<br>Normoxemoa survival 4217<br>Death OR 4.144
Diaz-Velez 2021	Peru	UMIC	Urban	1555	Tertiary	Inpatient	Cohort Study	COVID 19	>= 18 years	SpO2<90%	493	Missing adult 23<br>-COVID 278/493<br>Hopoxemia death 210<br>Hypoxemia survive 68<br>Normoxemia death 87<br>Normoxemia survival 128<br>Death OR 4.54
Diendere 2021	Other: Burkina Faso	LIC	Unclear	297	Mixed	Inpatient	Cross sectional study	COVID 19	9 - 65 years	SpO2<95%	442	COVID 64/442
Djelantik 2003	Indonesia	LMIC	Rural	4	Secondary	Inpatient	Cohort Study	All patients	<24 months	SpO2<86%	4351	All child: 482/4351<br>Missing 45<br>-WHO Pneum 482/4306<br>-WHO severe or very severe 482/4306<br>Hypoxemia death 135<br>Hypoxemia survive 347<br>Normoxemia death 250<br>Normoxemia survival 3574<br>Death OR 5.5618
Duke 2002	Other: Papua New Guinea	LMIC	Mixed	1600	Secondary	Inpatient	Cohort Study	ARI and non ARI	0-59 months	SpO2<88%	491	All neon: 57/132<br>-NE 15/25<br>-Pneum 17/22<br>-Preterm 10/33<br>-Sepsis 15/34<br>All child: 200/359

Study	Country	WB Cat	Context	Altitude (meters)	Level	Setting	Design	Condition	Age range	Hypox definition	N	Hypoxaemia (n/N)
												-WHO pneum 162/233<br>-Malaria 0/5<br>-Mening 21/40<br>-Sepsis 6/10<br>-Diar 3/35<br>-Malnut 5/21<br>Hypoxemia survive 22<br>Hypoxemia death 236<br>Normoxemia death 7<br>Normoxemia survival 226<br>Death OR 3.01
Duke 2008	Papua New Guinea	LMIC	Unclear	Three hospitals were based in highlands provinces (Mount Hagen, Mendi, and Kundiawa) at 1600–1800 m above sea level, one in a coastal province (Wewak), and one inland at 400 m above sea level (Maprik)	Secondary	Inpatient	Non Randomized Exp Trial	Pneumonia	0-59 months	SpO2<90%	4130	Child:<br>- Pneum (WHO classified): 330/578
Emdin 2015	Pakistan	LMIC	Urban	10	Primary	Outpatient	Cross sectional study	All patients	0-59 days	SpO2<90%	529	All neon: 2/529<br>Missing 1
English 2003	Kenya	LMIC	Mixed	5	Secondary	Inpatient	Cohort Study	All patients	0-90 days	SpO2<90%	1080	All neon: 123/692<br>Missing 76<br>All child: 41/340<br>Missing 48

Study	Country	WB Cat	Context	Altitude (meters)	Level	Setting	Design	Condition	Age range	Hypox definition	N	Hypoxaemia (n/N)
Enoch 2019	Kenya	LMIC	Mixed	762	Secondary	Inpatient	Cross sectional study	All patients	1 month to 11 years.	SpO2<90%	27906	All child: 1423/14232<br>Missing 0
Evans 2012	Other: Malawi	LIC	Unclear	779	Tertiary	Inpatient	Cross sectional study	All patients	Not reported	SpO2<90%	144	All adult: 14/144<br>Missing 0
Fagbohun 2020	Other: Paraguay	UMIC	Urban	43	Tertiary	Inpatient	Cohort Study	Pneumonia	35 days to 15 years	SpO2<90%	860	-Child WHO Pneum 112/860<br>-Pneum radiological 112/860<br>Hypoxemia death 31<br>Hypoxemia survive 81<br>Normoxemia death 25<br>Normoxemia survival 723<br>Death OR 11.068
Faruk 2022	Bangladesh	LMIC	Urban	85	Tertiary	Inpatient	retrospective chart review study	diarrhea, (iii) vomiting, and (iv) severe malnutrition	0 - 59 months	SpO2<90%	306	-Malaria 30/306
Fashanu 2020	Nigeria	LMIC	Mixed	380	Mixed	Mixed	Non-randomised experimental study (including pre-post intervention comparisons)	Pneumonia	< 5 years	SpO2<90%	1817	Missing child 320<br>-WHO Pneum 874/1497
Foran 2010	Other: Zambia	LIC	Mixed	1283	Secondary	Inpatient	Cross sectional study	All patients	1 month to 75 years	SpO2<90%	192	All child: 5/83<br>Missing 0<br>All adult: 10/109<br>Missing 0
Friedman 2020	Mexico	UMIC	Unclear	1111	Ambulance	Emergency	Observational study	Respiratory Patients	1 - 104 years	SpO2<90%	All during 2019: 1569<br><br>During peak of pandemic: 329	- Adult Pneum 245/720

Study	Country	WB Cat	Context	Altitude (meters)	Level	Setting	Design	Condition	Age range	Hypox definition	N	Hypoxaemia (n/N)
Fu 2006	Colombia, Ghana, India, Mexico, Pakistan, South Africa, Vietnam, and Zambia.	UMIC	Mixed	690	Tertiary	Inpatient	Randomised controlled trial	Pneumonia	3-59 months	SpO2<90%	1704	Child missing 10<br>-WHO Pneum 327/1694<br>-WHO severe or very severe 327/1694
Graham 2019	Nigeria	LMIC	Mixed	275	Secondary	Inpatient	Cohort Study	All patients	0-59 months	SpO2<90%	overall : 23926<br><br>Neonate: 7473<br><br>Children (1-59 months): 4619+ 8060 = 12679<br><br>Children 5-14 years = 3671	All Neon: 1363/7473<br>-NE 821/2458<br>-Prem 387/1500<br>-Sepsis 671/3195<br>All Child: 1304/16453<br>-Pneum WHO 486/2073 (sev 367/1017; non 119/717)<br>-Asthma 20/98<br>-HIV 2/19<br>-Malaria 428/5035<br>-Mening 75/431<br>-Seizures 238/1301<br>-Sepsis 356/4092<br>-Trauma 20/282<br>-Diar 86/1410<br>-Malnut 45/1286<br>Death OR 8.4
Graham 2021	Uganda	LIC	Unclear	1100	Primary	Outpatient	Cohort Study	All patients	Neonate - 50+ years	SpO2<90%	5813	All Neon: 0/16<br>All Child: 24/2480<br>-Fever 18/1835<br>-Pneum 8/142<br>-Malaria 10/882<br>-Sepsis 0/55<br>-Diar 3/300<br>-ARI 118/2206<br>All Adult: 3/3284<br>-Pneum 0/14<br>-Malaria 1/965<br>-Sepsis 0/56

Study	Country	WB Cat	Context	Altitude (meters)	Level	Setting	Design	Condition	Age range	Hypox definition	N	Hypoxaemia (n/N)
Graham 2022	Nigeria	LMIC	Unclear/ unspecif ied	380	Secondary	Inpatient	Prospective evaluation study	All neonates and children	o days to 15 years	SpO2<90%	10267	All Neon: 53/326<br>All Child: 57/506
Hau 2018	Tanzania	LMIC	Urban	1140	Tertiary	Inpatient	Cohort Study	All patients	2-12 years	SpO2<90%	537	All child: 30/537<br>Hypoxemia death13<br>Hypoxemia survive 73<br>Normoxemia death 17<br>Normoxemia survival 434 Death OR 4.546334
Homayounie h 2020	Iran	LMIC	Urban	1200	Tertiary	Inpatient	Cohort Study	COVID 19	21-100 years	SpO2<90%	90	Missing adult 15<br>Hypoxemia death 7<br>Hypoxemia survive 8<br>Normoxemia death 15<br>Normoxemia survival 45<br>Death OR 2.625
Honarvar 2021	Iran	LMIC	Urban	1305	Tertiary	Inpatient	Cross sectional study	ARI	0 - 99 years	SpO2<93%	2835	-Adult COVID 408/819<br>Hypoxemia death 97<br>Hypoxemia survive 207<br>Normoxemia death 191<br>Nomoxemia survival 1480<br>Death OR 3.64
HongXIAO 2010	China	UMIC	Urban	1840	Tertiary	Inpatient	Cross sectional study	H1N1 virus infection	2 - 52 years	SaO2<95%	156	-Child Bronch 0/31<br>-Adult Pneum 0/125
Hooli 2020	Other: Malawi	LIC	Mixed	1100	Mixed	Inpatient	Cohort Study	Pneumonia	0 - 2 months	SpO2<90%	In hospit al: 1618<br><br>Outpa tient health center : 235	-Child Pneum 219/1491<br>Hypoxemia death 15<br>Hypoxemia survive 204<br>Normoxemia death 20<br>Normoxemia survival 1252<br>Death OR 7.1
Hu 2020	China	UMIC	Mixed	4	Mixed	Mixed	Cohort Study	ARI	18-97 years	SpO2<90%	435	Adult missing 31<br>-Adult Pneum 19/404 (pneum radio 19/404)
Husain 2009	Pakistan	LMIC	Urban	10	Tertiary	Inpatient	Cohort Study	Pulmonary embolism	23-75 years	unclear	30	Hypoxemia death 4<br>Hypoxemia survive 16<br>Normoxemia death 0<br>Normoxemia survive 10
Hussain 2005	Pakistan	LMIC	Urban	10	Tertiary	Inpatient	Cohort Study	Asthma	17-80 years	SpO2<90%	102	Adult missing 7<br>-Adult Asth 16/95

Study	Country	WB Cat	Context	Altitude (meters)	Level	Setting	Design	Condition	Age range	Hypox definition	N	Hypoxaemia (n/N)
Ibraheem 2014	Nigeria	LMIC	Urban	303	Tertiary	Mixed	Cross sectional study	Pneumonia	2- 59 months	SpO2<90%	200	-Child WHO Pneum 83/200
Izadnegahdar 2012	Other: Colombia, Ghana, India, Mexico, Pakistan, South Africa, Vietnam and Zambia	Mixed	Unclear	690	Tertiary	Inpatient	Cohort Study	Pneumonia	2-59 months	SpO2 <92%	2714	Child missing 1275<br>-Child Pneum 223/1439 (severe 223/1439)
Jain 2013	India	LMIC	Unclear	160	Tertiary	Inpatient	Case control study	ARI	3-59 months	SpO2<90%	181	-Child Pneum 32/181
Jain 2018	India	LMIC	Urban	81	Tertiary	Inpatient	Case control study	Asthma	5-15 years	SpO2<90%	60	-Child Asth 50/60
Jeena 2003	South Africa	UMIC	Urban	6	Tertiary	Outpatient	Cohort Study	Bronchiolitis	3-24 months	SpO2 <92%	114	-Child Bronch 13/114
Jullien 2022	Other: Bhutan	LMIC	Urban	3280	Tertiary	Inpatient	Cross sectional study	Pneumonia	2 - 59 months	SpO2<90%	189	Child missing 42<br>-Child WHO Pneum 111/147 (Radiological 32/39)
Junge 2006	Gambia, The	LIC	Urban	6	Tertiary	Inpatient	Cohort Study	Multiple diagnoses	0-15 years	SpO2<90%	4047	All Neon: 51/310<br>All Child: 188/3269<br>-WHO Pneum 51/436<br>-Anaem 4/225<br>-Mal 30/1044<br>-Mening 2/74<br>-Diar 0/114<br>-Maln 5/271<br>Hypoxemia death 90<br>Hypoxemia survive 98<br>Normoxemia death 338<br>Normoxemia survival 2743<br>Death OR 7.452
Kapoor 2022	India	LMIC	Urban	160	Tertiary	Inpatient	Case control study	Pneumonia	1 - 59 months	SpO2<90%	180	-Child WHO Pneum 65/180 (severe 65/180)<br>Hypoxemia death 15<br>Hypoxemia survive 50<br>Normoxemia death 2<br>Normoxemia survival 113<br>Death OR 15

Study	Country	WB Cat	Context	Altitude (meters)	Level	Setting	Design	Condition	Age range	Hypox definition	N	Hypoxaemia (n/N)
Karim 2021	Other: Rwanda	LIC	Urban	1598	Tertiary	Emergency	Cohort Study	Head injury	Not reported	unclear	534	-Adult Trauma 30/534
Katagira 2016	Uganda	LIC	Urban	1200	Tertiary	Inpatient	Cohort Study	HIV	Not reported	SpO2< 94%	631	-Adult Tuberculosis 216/631
Kaya 2016	Turkey	UMIC	Urban	1141	Tertiary	Emergency	Cohort Study	GI bleeding	>=18 years	SpO2<92%	600	Overall: 14/600
Kayambankadzanja 2021	Malawi	LIC	Unclear	779	Mixed	Inpatient	Cross sectional study	All patients	>=18 years	SpO2 < 90%	1135	All Adult 45/1135<br>Hypoxemia death 10<br>Hypoxemia survive 35<br>Normoxemia death 74<br>Normoxemia survival 1016<br>Death OR 4
KazemiAski 2022	Iran	LMIC	Unclear	1305	Tertiary	Inpatient	Case control study	COVID 19	Not reported	SpO2<93%	44	-Adult COVID 13/44
Kelly 2015	Botswana	UMIC	Unclear	1013	Tertiary	Inpatient	Cohort Study	Pneumonia	1-23 months	SpO2<90%	8852	Child missing 1<br>-Child WHO Pneum 92/238<br>-Child HIV 11/20
King 2022	Other: Malawi	LIC	Unclear	779	Primary	Inpatient	Cohort Study	All patients	0 - 12 years	SpO2 < 90%	826	All Neon 11/38<br>Missing 10<br>All Child 60/788<br>Missing 8<br>-Pneum 32/108 (unspecified 7/55)<br>-Mal 28/310<br>-Seps 12/92<br>-Trauma 4/200<br>-Diar 1/18<br>-Maln 2/36<br>Hypoxemia death 11<br>Hypoxemia survive 80<br>Normoxemia death 15<br>Normoxemia survival 696<br>Death OR 6.67
Kintwa 2021	Other: Papua New Guinea	LMIC	Unclear	667	Secondary	Inpatient	Cohort Study	Children with malnutrition or gastro-enteritis, dehydration and pneumonia with associated severe malnutrition	2 - 112 months	SpO2<90%	150	-Child Maln 29/140<br>Hypoxemia death 12<br>Hypoxemia survive 17<br>Normoxemia death 7<br>Normoxemia survival 104<br>Death OR 10.499

Study	Country	WB Cat	Context	Altitude (meters)	Level	Setting	Design	Condition	Age range	Hypox definition	N	Hypoxaemia (n/N)
Kiputa 2022	Tanzania	LMIC	Urban	1018	Tertiary	Inpatient	Cross sectional study	All patients	0 - 28 days	SpO2 < 90%	348	All Neon 74/348<br>Hypoxemia death 38<br>Hypoxemia survive 36<br>Normoxemia death 40<br>Normoxemia survival 234<br>Death OR 6.63
Koss 2015	Uganda	LIC	Unclear	1100	Tertiary	Inpatient	Cohort Study	HIV and Pneumonia (half had TB)	>18 years	SpO2<90%	835	-Adult HIV 137/835<br>Hypoxemia death 43<br>Hypoxemia survive 94<br>Normoxemia death 109<br>Normoxemia survival 589<br>Death OR 2.469
Krebs 2017	Other: Rwanda	LIC	Urban	1567	Tertiary	Emergenc y	Cohort Study	Head injury	10 - 50 years	SpO2< 90%	670	-Adult Trauma 23/670<br>Hypoxemia death 13<br>Hypoxemia survive 20<br>Normoxemia death 19<br>Normoxemia survival 509<br>Death OR 16.88
Krithika 2022	India	LMIC	Urban	160	Tertiary	Inpatient	Cohort Study	All patients	1 month to 12 years	SpO2<90%	600	All Child: 49/600<br>Hypoxemia death 39<br>Hypoxemia survive 10<br>Normoxemia death 11<br>Normoxemia survival 540<br>Death OR 192.12
Kuti 2013	Gambia, The	LIC	Rural	0	Secondary	Inpatient	Cohort Study	ARI	2- 59 months	SpO2<90%	420	-Child WHO Pneum 81/410 (severe 81/410)
Kuti 2013	Gambia, The	LIC	Rural	34	Secondary	Inpatient	Cohort Study	ARI	2 - 59 months	SpO2<90%	420	-Child WHO Pneum 81/420 (severe 81/420)<br>Hypoxemia death 6<br>Hypoxemia survive 67<br>Normoxemia death 9<br>Normoxemia survival 307<br>Death OR 3.05
Kwizera 2020	Uganda	LIC	Urban	1100	Tertiary	Emergenc y	Cohort Study	AHRF (acute hypoxic respiratory failure)	>= 18 years	SpO2<91%	7300	All Adult: 327/7300<br>Hypoxemia death 177<br>Hypoxemia survive 30<br>Normoxemia death 77<br>Normoxemia survival 43<br>Death OR 3.3
Laghari 2019	Pakistan	LMIC	Urban	24	Tertiary	Inpatient	Randomised controlled trial	Pneumonia	28 days to 5 years	SpO2<92%	100	Child:<br>-All WHO Pneumonia: 34/100

Study	Country	WB Cat	Context	Altitude (meters)	Level	Setting	Design	Condition	Age range	Hypox definition	N	Hypoxaemia (n/N)
Laher 2022	South Africa	UMIC	Urban	1034	Tertiary	Emergency	Cohort Study	HIV	>= 18 years	SpO2<90%	1224	Adult:<br>-HIV/AIDS: 196/1224<br>-Death OR: 1.74
Laman 2005	Other: Papua New Guinea	LMIC	Urban	35	Tertiary	Inpatient	Cohort Study	Pneumonia	1 - 60 months	SpO2<90%	77	Child:<br>-All WHO Pneumonia: 20/77
Landes 2017	Ethiopia	LIC	Urban	1330	Tertiary	Emergency	Cohort Study	Head injury	All ages	SpO2<90%	204	Adult:<br>-Trauma: 42/204<br>-Hypoxemia_Death: 13<br>-Hypoxemia_Survive: 20<br>-Normoxemia_Death: 8<br>-Normoxemia_Survival: 129<br>-Death OR: 10.83
Leligdowicz 2021	Uganda	LIC	Unclear	1204	Secondary	Emergency	Cohort Study	Febrile illness	2 - 59 months	SpO2<90%	2502	Child:<br>-Fever: 102/2469<br>-Mal: 33/1317
LeRoux 2021	South Africa	UMIC	Urban	1034	Mixed	Outpatient	Cohort Study	Pneumonia	<2months - 24 months	SpO2 <92%	174	Neon:<br>-Pneum: 15/47<br>Child:<br>-All WHO Pneum: 41/127
Li 2020	China	UMIC	Urban	37	Tertiary	Inpatient	Cohort Study	COVID 19	<2 months to >24 months	SpO2 < 93%	199	Adult:<br>COVID-19: 6/199
Libster 2010	Argentina	UMIC	Urban	25	Tertiary	Inpatient	Cohort Study	ARI or febrile illness	<1 month-226 months	SpO2<93%	251	Child:<br>-Bronch: 206/251<br>-Hypoxemia_Death: 8<br>-Hypoxemia_Survive: 198<br>-Normoxemia_Death: 5<br>-Normoxemia_Survival: 40<br>-Death OR: 0.32
Ling 2020	China	UMIC	Urban	1840	Tertiary	Inpatient	Case control study	Pneumonia	0-15 years	SpO2< 92%	345	Child:<br>-Pneum unspecified: 69/345
Liu 2020	China	UMIC	Urban	37	Tertiary	Inpatient	Cohort Study	COVID 19	29-97 years	SpO2<94%	109	Adult:<br>COVID-19: 31/109
Lodha 2004	India	LMIC	Urban	239	Tertiary	Emergency	Cross sectional study	ARI	0-59 months	SpO2<90%	109	Child:<br>All WHO Pneum: 28/109
Lowlaavar 2016	Uganda	LIC	Unclear	1480	Tertiary	Inpatient	Cohort Study	All patients	6-60 months	SpO2<90%	1307	All child: 332/1307<br>-Missing: 16<br>-Hypoxemia_Death: 30<br>-Hypoxemia_Survive: 302<br>-Normoxemia_Death: 32

Study	Country	WB Cat	Context	Altitude (meters)	Level	Setting	Design	Condition	Age range	Hypox definition	N	Hypoxaemia (n/N)
												-Normoxemia_Survival: 927<br>-Death OR: 2.8777
Ma 2019	Uganda	LIC	Mixed	1204	Mixed	Inpatient	Cohort Study	Pneumonia	0-59 months	SpO2<90%	155	Child:<br>-All WHO Pneum: 133/155<br>-Hypoxemia_Death: 21<br>-Hypoxemia_Survive: 112<br>-Normoxemia_Death: 1<br>-Normoxemia_Survival: 21<br>-Death OR: 3.9375
Magree 2005	Fiji	UMIC	Urban	662	Tertiary	Inpatient	Cohort Study	ARI	1 month to 5 years	SpO2<90%	248	Child:<br>-Pneum radiological: 38/59<br>-Pneum unspecified: 41/248
Maitland 2006	Kenya	LMIC	Rural	762	Secondary	Inpatient	Cohort Study	Malnutrition	>3 months	SpO2<95%	920	Child:<br>-Maln: 161/612<br>-Hypoxemia_Death: 45<br>-Hypoxemia_Survive: 116<br>-Normoxemia_Death:m 113<br>-Normoxemia_Survival: 444<br>-Death OR: 1.56
Maitland 2003	Kenya	LMIC	Rural	762	Secondary	Inpatient	Cohort Study	Malaria	<3 months to 60+ months	SpO2< 90%	515	Child:<br>-Mal: 86/501<br>-Hypoxemia_Death: 25<br>-Hypoxemia_Survive: 61<br>-Death OR: 4.3
Marcolino 2021	Brazil	UMIC	Mixed	320	Mixed	Mixed	Cohort Study	COVID 19	Not reported	SpO2<90%	2054	Adult:<br>-Missing: 90<br>-COVID-90: 263/1964<br>-Hypoxemia_Death: 105<br>-Hypoxemia_Survive: 151<br>-Normoxemia_Death: 300<br>-Normoxemia_Survival: 1351<br>-Death OR: 3.1
MarMinn 2021	Other: Myanmar	LMIC	Urban	702	Tertiary	Inpatient	Cohort Study	Sepsis	29-64 years	SpO2<90%	509	Adult:<br>-Seps: 62/509<br>-Hypoxemia_Death: 15<br>-Hypoxemia_Survive: 47<br>-Normoxemia_Death: 34<br>-Normomxemia_Survival: 411<br>-Death OR: 3
Martinez-Medina 2010	Mexico	UMIC	Urban	282	Tertiary	Inpatient	Cross sectional study	Pneumonia or bronchiolitis	1 month to 4 years	SpO2<94%	62	Child:<br>-All WHO Pcneum: 35/62<br>-Pneum radiological: 35/62
Mathisen 2010	Other: Nepal	LMIC	Urban	3265	Primary	Outpatient	Cohort Study	ARI	2 - 34 months	SpO2<90%	2219	Child:<br>-All WHO Pneum: 42/2219

Study	Country	WB Cat	Context	Altitude (meters)	Level	Setting	Design	Condition	Age range	Hypox definition	N	Hypoxaemia (n/N)
												-WHO severe or very severe: 35/131<br>-WHO non severe pneum: 7/2088
McCollum 2019	Other: Malawi	LIC	Unclear	779	Secondary	Inpatient	Randomised controlled trial	Pneumonia or HIV or Malnutrition	1 to 59 months	SpO2<90%	644	Child:<br>-All WHO Pneum: 415/644<br>-WHO severe or very severe: 415/644<br>-Hypoxemia_Death: 57<br>-Hypoxemia_Survive: 358<br>-Normoxemia_Death: 31<br>-Normomxemia_Survival: 198<br>-Death OR: 1
McCollum 2016	Malawi	LIC	Rural	779	Primary	Outpatient	Implementation study	Pneumonia	2 months to 5 years	SpO2<90%	14092	Child:<br>-Missing: 826<br>-All WHO Pneum: 652/13266
McCollum 2013	Other: Malawi	LIC	Mixed	779	Secondary	Inpatient	Cohort Study	All patients	0 months - 15 years	SpO2<90%	761	All neon: 7/34<br>All child: 33/727<br>-All WHO Pneum: 26/151<br>-WHO severe or very severe: 24/138<br>-WHO non severe pneum: 2/13<br>-Anaemia unspecified: 1/31<br>-Mal: 8/427<br>-Seps: 1/29<br>-Diar: 0/29<br>-Maln: 1/19<br><br>Hypoxemia_Death: 8<br>Hypoxemia_Survive: 32<br>Normoxemia_Death: 33<br>Normomxemia_Survival: 688<br>Death OR: 5
McCracken 2013	Other: Guatemala	UMIC	Mixed	Santa Rosa: 900<br><br>Guatemala City: 1500<br><br>Quetzaltenango: 2300	Mixed	Mixed	Cross sectional study	Pneumonia	All ages	SpO2< 90%	8852	Child:<br>-Pneum unspecifc: 365/1249<br>Adult:<br>-Pneum any: 30/84
Mejia 2020	Peru	UMIC	Urban	0	Tertiary	Inpatient	Cohort Study	COVID 19	Not reported	SpO2<90%	369	Adult:<br>-COVID-19: 238/369<br>-Hypoxemia_Death: 183<br>-Hypoxemia_Survive: 186

Study	Country	WB Cat	Context	Altitude (meters)	Level	Setting	Design	Condition	Age range	Hypox definition	N	Hypoxaemia (n/N)
												-Normoxemia_Death: 19<br>-Normomxemia_Survival: 112<br>-Death OR: 5.76
Merida-Vieyra 2019	Mexico	UMIC	Urban	2240	Tertiary	Inpatient	Cohort Study	Pneumonia	0 - 18 years	SpO2<92%	154	Child:<br>-Pneum radiological: 148/154
Mor 2010	Uganda	LIC	Unclear	1100	Tertiary	Inpatient	Cohort Study	Diarrhoea	9-36 months	SpO2 <92%	926	Child:<br>-Diar: 5/926
Morgan 2018	Kenya	LMIC	Unclear	1800	Tertiary	Inpatient	Cohort Study	All patients	Not reported	SpO2 < 89%	407	All Neon: 119/407<br>-Prem: 42/87<br>-Hypoxemia_Death: 36<br>-Hypoxemia_Survive: 83<br>-Normoxemia_Death: 18<br>-Normomxemia_Survival: 270<br>-Death OR: 6.14
Muller 2012	Other: Guetemal a	UMIC	Unclear	Cuilapa (893), Guatemal a City (1499) and Quetzalte nango (2333).	Mixed	Inpatient	Cross sectional study	Pneumonia	0-59 months	SpO2<90%	2384	Child:<br>-All WHO Pneum: 448/1993
Muro 2020	Tanzania	LMIC	Unclear	1018	Tertiary	Inpatient	Cohort study	Severe community acquired pneumonia	2-59 months	SpO2<90%	250	Child:<br>- Pneum (WHO classified): 81/250<br>- Pneum (WHO severe): 81/250
Mvalo 2022	Other: Malawi	LIC	Unclear	779	Secondary	Inpatient	Cross sectional study	Pneumonia or HIV or Malnutrition	1 - 59 months	SpO2<90%	538	Child:<br>-All WHO Pneum: 343/538<br>-WHO severe or very severe: 343/538
Mwaniki 2009	Kenya	LMIC	Unclear	0	Secondary	Inpatient	Cohort Study	All patients	0 to > 60 days	SpO2<90%	15289	All Neon: 206/1105<br>All Child: 693/13183<br>-All WHO Pneum: 461/5489<br>-WHO severe or very severe: 447/4792<br>-WHO non severe pneum: 14/697<br>-Anaemia unspecified: 30/717<br>-Mal: 244/4982<br><br>Hypoxemia_Death: 290<br>Hypoxemia_Survive: 687<br>Normoxemia_Death: 881<br>Normomxemia_Survival: 13298<br>Death OR: Admissions aged >= 60 days: 0.28/0.05= 5.6

Study	Country	WB Cat	Context	Altitude (meters)	Level	Setting	Design	Condition	Age range	Hypox definition	N	Hypoxaemia (n/N)
												Admissions aged 7- 59 days: 0.39/0.07= 5.6<br><br>Admissions in 1st week of life: 1.34/0.32= 4.2
Nabukeera-Barungi 2018	Uganda	LIC	Urban	1100	Tertiary	Inpatient	Cohort Study	Malnutrition	6 to 59 months	SpO2 < 94%	400	Child:<br>-Missing: 213<br>-Maln: 25/187<br>-Hypoxemia_Death: 6<br>-Hypoxemia_Survive: 19<br>-Normoxemia_Death: 33<br>-Normomxemia_Survival: 342<br>-Death OR: 3
Nantanda 2008	Uganda	LIC	Mixed	1100	Tertiary	Inpatient	Cross sectional study	Pneumonia	2 to 59 months	SaO2 <92%	157	Child:<br>-Missing: 68<br>-All WHO Pneum: 62/157<br>-WHO severe or very severe: 62/157<br>-Hypoxemia_Death: 18<br>-Hypoxemia_Survive: 44<br>-Normoxemia_Death: 6<br>-Normomxemia_Survival: 89<br>-Death OR: 6.07
Navuluri 2023	Kenya	LMIC	Unclear	2100	Tertiary	Inpatient	Cohort Study	All patients	18-103 years	SpO2< 89%	4104	All Adult: 975/4104
Nemani 2016	India	LMIC	Unclear	252	Tertiary	Inpatient	Cross sectional study	Pneumonia	1 - 59 months	SpO2 <92%	135	Child:<br>-All WHO Pneum: 54/135<br>-WHO severe or very severe: 34/34<br>-WHO non severe pneum: 20/101<br>-Maln: 39/52
Nemati 2021	Iran	LMIC	Unclear	1305	Tertiary	Inpatient	Cohort Study	COVID 19	<50years - >=70years	SpO2< 94%	998	Adult:<br>-COVID-19: 612/998<br>-Hypoxemia_Death: 61<br>-Hypoxemia_Survive: 513<br>-Normoxemia_Death: 31<br>-Normomxemia_Survival: 341<br>-Death OR: 1
Nielsen 2018	Peru	UMIC	Urban	1555	Tertiary	Emergency	Case control study	All patients (with serious clinical deterioration)	0-18 years	SpO2<90%	974	All Child: 85/991
Njuguna 2018	Kenya	LMIC	Unclear	762	Secondary	Inpatient	Randomised controlled trial	HIV	0-12 years	SpO2 <92%	181	Child:<br>-HIV/AIDS: 44/158

Study	Country	WB Cat	Context	Altitude (meters)	Level	Setting	Design	Condition	Age range	Hypox definition	N	Hypoxaemia (n/N)
Nyawanda 2016	Kenya	LMIC	Rural	762	Secondary	Inpatient	Cross sectional study	ARI	<5 years	SpO2<90%	4714	Child:<br>-Bronch: 121/470<br>-Pneum unspecified: 787/4696
Ojuawo 2020	Nigeria	LMIC	Urban	380	Tertiary	Inpatient	Cross sectional study	ARI	20 - 65 years	unclear	102	Adult:<br>-Missing: 35<br>-Pneum any: 34/136<br>-Hypoxemia_Death: 13<br>-Hypoxemia_Survive: 21<br>-Normoxemia_Death: 4<br>-Normomxemia_Survival: 64<br>-Death OR: 61
Oktaria 2021	Indonesia	LMIC	Mixed	367	Secondary	Inpatient	Cross sectional study	Pneumonia	2 - 59 months	SpO2<90%	133	Child:<br>-Missing: 8<br>-All WHO Pneum: 18/133
Oliveira 2022	Brazil	UMIC	Mixed	320	Mixed	Inpatient	Cohort Study	COVID 19	< 20 years	SpO2<95%	21591	Child:<br>-COVID-19: 7523/21591<br>-Hypoxemia_Death: 505<br>-Hypoxemia_Survive: 3174<br>-Normoxemia_Death: 391<br>-Normomxemia_Survival: 6841<br>-Death OR: 2.67
Olupot-Olupot 2020	Uganda	LIC	Unclear	1100	Secondary	Inpatient	Case control study	Malaria	2 months - 12 years	SpO2< 92%	662	Child:<br>-Mal: 49/662<br>-Hypoxemia_Death: 14<br>-Hypoxemia_Survive: 35<br>-Normoxemia_Death: 49<br>-Normomxemia_Survival: 564<br>-Death OR: 4.61
Ondoa-Onama 2003	Uganda	LIC	Urban	1100	Tertiary	Inpatient	Case control study	Sick neonates (low APGAR)	0-2 days	SpO2<92%	124	Neon:<br>-NE: 16/109<br>-Hypoxemia_Death: 11<br>-Hypoxemia_Survive: 39<br>-Normoxemia_Death: 4<br>-Normomxemia_Survival: 70<br>-Death OR: 4.94
Onubogu 2022	Nigeria	LMIC	Urban	380	Tertiary	Outpatient	Cross sectional study	Asthma	6 - 16 years	SpO2< 92%	189	Child:<br>-Asth: 6/189
Orimadegun 2013	Nigeria	LMIC	Urban	237	Tertiary	Emergency	Cross sectional study	All patients	0-60+ months	SpO2<90%	1726	All Neon: 188/454<br>All child: 306/1272<br>-All WHO Pneum: 154/313<br>-WHO severe or very severe: 154/313<br>-Mal: 76/527<br>-Mening: 4/50

Study	Country	WB Cat	Context	Altitude (meters)	Level	Setting	Design	Condition	Age range	Hypox definition	N	Hypoxaemia (n/N)
												-Seps: 38/168<br>-Diar: 6/94<br>-Hypoxemia_Death: 56<br>-Hypoxemia_Survive: 438<br>-Normoxemia_Death: 85<br>-Normomxemia_Survival: 1147<br>-Death OR: 1.86
Orimadegun 2014	Nigeria	LMIC	Urban	230	Tertiary	Inpatient	Cross sectional study	Malaria	6- 59 months	SpO2<90%	369	Child:<br>-Anaemia unspecified: 82/288<br>-Mal: 110/369<br>-Hypoxemia_Death: 19<br>-Hypoxemia_Survive: 91<br>-Normoxemia_Death: 11<br>-Normomxemia_Survival: 248<br>-Death OR: 4.71
Padmaprakash 2021	India	LMIC	Urban	160	Tertiary	Inpatient	Cohort Study	COVID 19	15-94 years	SpO2< 90%	1536	Adult:<br>-COVID-19: 79/1536<br>-Hypoxemia_Death: 49<br>-Hypoxemia_Survive: 30<br>-Normoxemia_Death: 27<br>-Normomxemia_Survival: 1430<br>-Death OR: 61.154
Papali 2017	Other: Haiti	LMIC	Urban	98	Secondary	Emergency	Non-randomised experimental study (including pre-post intervention comparisons)	Sepsis	>= 17 years	SpO2<90%	166	All adult: 40/166<br>- Seps: 40/166
Patel 2021	India	LMIC	Urban	160	Mixed	Inpatient	Cross sectional study	Mucormycosis	Not reported	Not reported	287	All adult: 86/287<br>- covid 19: 86/287
Pati 2013	India	LMIC	Urban	160	Tertiary	Mixed	Cross sectional study	Influenza	22-80 years	Not reported	106	All adult: 5/97<br>- pneum: 5/97
PERCHStudy Group 2019	Other: Bangladesh, The Gambia, Kenya, Mali, South	Mixed	Mixed	526	Mixed	Inpatient	Case control study	Pneumonia	1 - 59 months	SpO2 < 92%	3981<br><br>Thea paper-1935	Child:<br>- Pneum (WHO classified): 1423/3981<br>- Pneum (WHO severe): 1423/3981<br>- Pneum (radiological): 862/1935

Study	Country	WB Cat	Context	Altitude (meters)	Level	Setting	Design	Condition	Age range	Hypox definition	N	Hypoxaemia (n/N)
	Africa, Thailand and Zambia											
Pukai 2020	Other: Papua New Guinea	LMIC	Unclear	667	Tertiary	Emergency	Randomised controlled trial	Bronchiolitis or pneumonia	<2 yrs	SpO2< 90%	199	Child:<br>- Pneum (WHO classified): 182/199
Puumalainen 2008	Philippines	LMIC	Unclear	442	Tertiary	Inpatient	Randomised controlled trial	Pneumonia	2-23 months	SpO2<90%	821 (episodes: 1195)	Child:<br>- Pneum (WHO classified): 186/1151<br>- Pneum (WHO severe): 169/865<br>- Pneum (WHO non-severe): 17/286
Rabha 2020	Brazil	UMIC	Urban	320	Tertiary	Inpatient	Cross sectional study	COVID 19	0 - <18 years	SpO2< 94%	115	Child<br>- COVID-19: 5/115
Rahman 2021	Bangladesh	LMIC	Urban	85	Tertiary	Inpatient	Cross sectional study	Pneumonia	2 - 59 months	SpO2<90%	2646	Child<br>- Pneum (WHO classified): 1058/2646<br>- Pneum (WHO severe): 1058/2646
Rahnama&#039 2006	India	LMIC	Urban	160	Tertiary	Inpatient	Cohort Study	Asthma	29 - 186 months	SpO2<=92%	51	Child<br>- Asth: 23/51
Raihana 2015	Bangladesh	LMIC	Mixed	10	Tertiary	Mixed	Cohort Study	All patients	1-59 months	SpO2<90%	3374	All Child: 190/3374
Rajesh 2000	India	LMIC	Urban	160	Tertiary	Emergency	Cohort Study	All patients	0-2 months	SpO2<91%	200	All neon: 77/200<br><br>Mortality data:<br>- Hypoxemia_Death: 17<br>- Hypoxemia_Survive: 60<br>- Normoxemia_Death: 15<br>- Normoxemia_Survival: 108<br>- Crude OR of death: 2
Ramakrishna 2012	Other: Malawi	LIC	Unclear	779	Tertiary	Inpatient	Cohort Study	Pneumonia	2 months to 14 years	SpO2<90%	233	Child:<br>- Pneum (WHO classified): 87/233<br>- Pneum (WHO severe): 87/233<br><br>Mortality data:<br>- Hypoxemia_Death: 19<br>- Hypoxemia_Survive: 68<br>-Normoxemia_Death: 6<br>- Normoxemia_Survival: 140<br>- Crude OR of death: hypoxaemic versus normoxaemic: 7
Ramatillah 2022	Indonesia	LMIC	Urban	367	Tertiary	Inpatient	Cohort Study	COVID 19	> 18 years	SpO2 < 93%	91	Adult:<br>- COVID-19: 23/91

Study	Country	WB Cat	Context	Altitude (meters)	Level	Setting	Design	Condition	Age range	Hypox definition	N	Hypoxaemia (n/N)
Rao 2021	India	LMIC	Urban	160	Tertiary	Inpatient	Cohort Study	COVID 19	Not reported	SpO2 <94%	123	Child:<br>- COVID-19: 23/119<br><br>mortality data:<br>- Hypoxemia_Death: 8<br>- Hypoxemia_Survive: 15<br>- Normoxemia_Death: 6<br>- Normoxemia_Survival : 90<br>- Crude OR of death: hypoxaemic versus normoxaemic: 7.995
Rao 2012	India	LMIC	Urban	126	Tertiary	Emergency	Cross sectional study	ARI	2 to 59 months	SpO2<90%	261	Child:<br>- Pneum (WHO classified): 54/146<br>- Pneum (WHO severe): 50/104<br>- Pneum (WHO non-severe): 4/42<br><br>mortality data:<br>- Crude OR of death: hypoxaemic versus normoxaemic: 7
Riccetto 2006	Brazil	UMIC	Urban	320	Tertiary	Inpatient	Cohort Study	ARI	0-12 months	SpO2<90%	152	Child:<br>- Pneum (other): 33/149
Riviello 2016	Other: Rwanda	LIC	Urban	1500	Tertiary	Inpatient	Cohort Study	All patients	>15 years	SpO2<90%	1046	All adult: 126/1046
Rudd 2014	Uganda	LIC	Rural	1100	Primary	Inpatient	Cohort Study	Sepsis	28 days to >= 18 years	SpO2<94%	51	Child:<br>- Seps: 9/31<br><br>Adult:<br>- Seps: 11/20
Salah 2014	Other: Sudan	LIC	Urban	568	Tertiary	Emergency	Cross sectional study	Febrile illness	1 month to < 16 years	SpO2< 95%	150	Child:<br>- Fever: 1/150
Salah 2015	Other: Sudan	LIC	Urban	390	Tertiary	Emergency	Cross sectional study	ARI	2 - 60 months	SpO2<90%	150	Child:<br>- Pneum (WHO classified): 64/150<br>- Pneum (WHO severe): 7/77<br>- Pneum (WHO non-severe): 27/73
Saleh 2022	Egypt	LMIC	Unclear	321	Tertiary	Inpatient	Cohort Study	Pneumonia	2 - 59 months	SPO2 <94%	180	Child:<br>- Pneum (WHO classified): 48/180<br>- Pneum (radiological): 48/180
Sampathkumar 2021	India	LMIC	Unclear	160	Tertiary	Inpatient	Cohort Study	COVID 19	Not reported	SpO2 < 94%	52	Adult:<br>- COVID-19: 27/52
Santhanam 2008	India	LMIC	Urban	160	Tertiary	Emergency	Randomised controlled trial	Sepsis	1 month - 12 years	Not reported	147	Child:<br>- Seps: 41/147
Sarfaraz 2021	Pakistan	LMIC	Urban	900	Tertiary	Inpatient	Cohort Study	COVID 19	>=18 years	SpO2< 93%	170	Adult:<br>- COVID-19: 111/170

Study	Country	WB Cat	Context	Altitude (meters)	Level	Setting	Design	Condition	Age range	Hypox definition	N	Hypoxaemia (n/N)
												mortality data:<br>- Hypoxemia_Death: 54<br>- Hypoxemia_Survive: 57<br>- Normoxemia_Death: 13<br>- Normoxemia_Survival : 46<br>- Crude OR of death: hypoxaemic versus normoxaemic: 3.351
Sempertegui 2014	Ecuador	LMIC	Unclear	1117	Tertiary	Inpatient	RCT	Severe pneumonia	2-59 months	SpO2<90%	450	Child:<br>-All WHO Pneum: 383/450<br>-WHO severe or very severe: 383/450
Shahid 2016	Banglades h	LMIC	Urban	0	Tertiary	Inpatient	Case control study	Diarrhoea and sepsis	<5 years	SpO2<90%	104	Child:<br>- Seps: 6/26<br>- Diar: 7/104
Shahrin 2020	Banglades h	LMIC	Urban	85	Tertiary	Inpatient	Cohort Study	Pneumonia or malnutrition	0-59 months	SpO2<90%	191	Child:<br>- Missing: 15<br>- Pneum (WHO classified): 29/191<br>- Pneum (WHO severe): 29/191<br><br>mortality data:<br>- Hypoxemia_Death: 6<br>- Hypoxemia_Survive: 23<br>- Normoxemia_Death: 8<br>- Normoxemia_Survival : 139<br>- Crude OR of death: hypoxaemic versus normoxaemic: 4.53
Shahunja 2020	Banglades h	LMIC	Urban	85	Tertiary	Inpatient	Cross sectional study	Diarrhoea	<5 years	SpO2<90%	401	Child:<br>- Seps: 68/401<br><br>mortality data:<br>- Hypoxemia_Death: 29<br>- Hypoxemia_Survive: 39<br>- Normoxemia_Death: 16<br>- Normoxemia_Survival : 317<br>- Crude OR of death: hypoxaemic versus normoxaemic: 14.8
Sharawat 2021	India	LMIC	Unclear	160	Tertiary	Inpatient	Cohort Study	COVID 19	8-97 years	SpO2 <95%	1000	Adult:<br>- COVID-19: 552/1000
Sigauque 2009	Mozambic an	LMIC	Unclear	345	Tertiary	Inpatient	Cohort	Severe pneumonia	0-23 months	SpO2<90%	757	Child:<br>-All WHO Pneum: 177/685<br>-WHO severe or very severe: 177/685<br>-Hypoxemia_Death: 18<br>-Hypoxemia_Survive: 159<br>-Normoxemia_Death: 20

Study	Country	WB Cat	Context	Altitude (meters)	Level	Setting	Design	Condition	Age range	Hypox definition	N	Hypoxaemia (n/N)
												-Normomxemia_Survival: 387<br>-Death OR:
Siqueira 2022	Brazil	UMIC	Mixed	320	Mixed	Mixed	Cohort Study	COVID 19	13- 48 years	SpO2<95%	15105	Adult:<br>- COVID-19: 5715/15105<br><br>mortality data:<br>- Hypoxemia_Death: 1285<br>- Hypoxemia_Survive: 4430<br>- Normoxemia_Death: 573<br>- Normoxemia_Survival : 8817<br>- Crude OR of death: hypoxaemic versus normoxaemic: 4.8
Sirohiya 2022	India	LMIC	Urban	160	Tertiary	Inpatient	Cohort Study	COVID 19	All ages	SpO2<94%	2080	Adult:<br>- COVID-19: 811/2080
Smith 2011	Guatemal a	LMIC	Unclear	2600	Primary	Outpatient	Parallel RCT	Pneumonia	0-18 months	SpO2<87%	534	Child:<br>- Pneum (WHO classified): 173/661
Snouber 2022	Other: Algeria	LMIC	Unclear	800	Tertiary	Inpatient	Cross sectional study	COVID 19	18-88 years	SpO2<90%	112	Child:<br>- COVID-19: 55/112
Soto 2022	Peru	UMIC	Urban	1555	Tertiary	Inpatient	Cohort Study	COVID 19	Not reported	Spo2< 90%	1418	Adult:<br>- COVID-19: 806/1418<br><br>mortality data:<br>- Hypoxemia_Death: 479<br>- Hypoxemia_Survive: 327<br>- Normoxemia_Death: 115<br>- Normoxemia_Survival : 402<br>- Crude OR of death: hypoxaemic versus normoxaemic: 5
Srinivasan 2012	Uganda	LIC	Urban	1100	Tertiary	Inpatient	Randomised controlled trial	Pneumonia	6 - 59 months	SpO2<92%	352	Child:<br>- Pneum (WHO classified): 102/352<br>- Pneum (WHO severe): 102/352<br><br>Mortality data:<br>- Crude OR of death: hypoxaemic versus normoxaemic: 5
Stassen 2014	South Africa	UMIC	Mixed	1034	Primary	Emergenc y	Cross sectional study	Head injury	>= 18 years	SpO2<90%	66	Adult Trauma 25/66
Sultan 2021	Ethiopia	LIC	Mixed	1330	Mixed	Inpatient	Cross sectional study	COVID 19	17-92 years	unclear	92	Adult:<br>- COVID-19: 55/92

Study	Country	WB Cat	Context	Altitude (meters)	Level	Setting	Design	Condition	Age range	Hypox definition	N	Hypoxaemia (n/N)
Sutherland 2019	Other: Rwanda	LIC	Urban	1598	Tertiary	Emergency	Non-randomised experimental study (including pre-post intervention comparisons)	All patients	>15 years	SpO2<90%	1765	All adult: 214/1769
Sylvies 2020	Malawi	LIC	Unclear	779	Primary	Outpatient	Mixed Methods study and logbook review	Febrile illness	<5 years	SpO2 < 90%	3504	Child:<br>- Pneum (other): 29/63
Tesfaye 2020	Ethiopia	LIC	Unclear	2993	Primary	Outpatient	Randomised controlled trial	ARI	2 - 59 months	SpO2<90%	1804	Child:<br>- Pneum (WHO classified): 135/928
Thomas 2015	Philippines	LMIC	Urban	442	Secondary	Outpatient	Randomised controlled trial	Pneumonia	3 - 23 months	SpO2<90%	2951	Child:<br>- Pneum (WHO classified): 219/2951
Tiewsoh 2009	India	LMIC	Urban	160	Tertiary	Inpatient	Cross sectional study	Pneumonia	2- 60 months	SpO2<90%	200	Child:<br>- Pneum (radiological): 87/127
Tokman 2014	Uganda	LIC	Unclear	1100	Tertiary	Inpatient	Case control study	HIV	>=18 years	SpO2<90%	241	Adult:<br>- Pneum: 38/241
Tolossa 2022	Ethiopia	LIC	Urban	1330	Tertiary	Mixed	Cross sectional study	COVID 19	All ages	SpO2<94%	318	Adult:<br>- COVID-19: 234/318
Tran 2016	Vietnam	LMIC	Urban	398	Tertiary	Inpatient	Cross sectional study	ARI	0 -161 months	SpO2<93%	1082	Child:<br>- Pneum (WHO classified): 92/1082
Tuti 2021	Kenya	LMIC	Unclear	762	Secondary	Inpatient	Cohort Study	All patients	0-13 years	SpO2<90%	132737	Neon:<br>- missing: 1221<br><br>All child: 4510/64722
Usen 1999	Gambia, The	LIC	Mixed	34	Mixed	Inpatient	Cohort Study	ARI	2 - 33 months	SpO2<90%	1072	Child:<br>- Fever: 11/74<br>- Pneum (WHO classified): 63/1072<br>- Pneum (radiological): 12/170<br>- Mal: 4/103<br><br>mortality data:<br>- Hypoxemia_Death: 8<br>- Hypoxemia_Survive: 55

Study	Country	WB Cat	Context	Altitude (meters)	Level	Setting	Design	Condition	Age range	Hypox definition	N	Hypoxaemia (n/N)
												- Normoxemia_Death: 28<br>- Normoxemia_Survival : 981<br>- Crude OR of death: hypoxaemic versus normoxaemic: 5
VafadarMoradi 2021	Iran	LMIC	Unclear	1305	Tertiary	Inpatient	Cohort Study	COVID 19	Not reported	SpO2<90%	219	Adult:<br>- COVID-19: 142/219
vonderWeid 2018	Other: Senegal	LMIC	Unclear	450	Mixed	Emergency	Cross sectional study	ARI	2 - 59 months	SpO2<90%	44	Child:<br>- Pneum (other): 7/44<br>- Asth: 8/82
Vongchaiudomchoke 2016	Thailand	UMIC	Urban	287	Tertiary	Inpatient	Cohort Study	Pulmonary embolism	Not reported	SpO2<90%	300	Overall: 256/300
Wandi 2006	Other: Papua New Guinea	LMIC	Rural	667	Secondary	Inpatient	Cohort Study	All patients	1 month to 12 years	SpO2<90%	1313	All child: 458/1896<br>-All WHO pneum: 315/578<br>-Pneum radiological: 315/578<br>-Anaemia unspecified: 2/63<br>-Mal: 9/272<br>-Mening: 6/41<br>-Tuberculosis: 4/20<br>-Diar: 6/127<br>-Maln: 1/12<br>- Death OR: 0.12
Wasingya-Kasereka 2020	Uganda	LIC	Unclear	1100	Secondary	Emergency	Cross sectional study	All patients	Not reported	SpO2<90%	2599	All adult: 164/2599
Webb 2012	Kenya	LMIC	Unclear	0	Secondary	Inpatient	Cohort	Severe pneumonia, HIV, severe malnutrition, malaria	2-59 months	SpO2<90%	710	Child:<br>-All WHO Pneum: 134/568<br>-WHO severe or very severe: 134/568
Weber 2002	Gambia, The	LIC	Unclear	34	Mixed	Inpatient	Cohort Study	Sepsis - and meningitis subgroup	2 - 33 months	SpO2<90%	2097	Child:<br>-Mening: 4/89<br>-Seizures: 85/2097
Weber 2003	Other: Ethiopia, The Gambia, Papua New Guinea, Philippines	Mixed	Mixed	2200	Mixed	Outpatient	Cohort Study	All patients	0-2 months	SpO2 <90%	3303	All neon: 259/3303<br>-Death OR: 4.5
West 1999	Gambia, The	LIC	Urban	34	Tertiary	Inpatient	Case control study	ARI	1 week to 5 years	SpO2<90%	190	Child:<br>-All WHO pneum: 83/190<br>Hypoxemia death 8

Study	Country	WB Cat	Context	Altitude (meters)	Level	Setting	Design	Condition	Age range	Hypox definition	N	Hypoxaemia (n/N)
												Hypoxemia survive 45<br>Normoxemia death 4<br>Normoxemia survival 61<br>Death OR 2.57
Wollenstein-Betech 2020	Brazil	UMIC	Mixed	320	Mixed	Inpatient	Cross sectional study	COVID 19	0- 100 years	SpO2<95%	113214	Adult:<br>-COVID-19: 62908/113214
Worodria 2018	Uganda	LIC	Urban	1100	Tertiary	Inpatient	Cohort Study	ARI	>=18 years	SpO2<90%	1887	-Adult Pneum 200/1887<br>-Adult HIV 136/1192<br>Hypoxemia death 86<br>Hypoxemia survive 114<br>Normoxemia death 286<br>Normoxemia survival 1401<br>Death OR 3.75
Xiong 2021	China	UMIC	Mixed	1840	Mixed	Inpatient	Cross sectional study	COVID 19	<65 - >=65 years	SpO2<90%	1143	-Adult COVID 913/1027<br>Hypoxemia death 39<br>Hypoxemia survive 45<br>Normoxemia death 15<br>Normoxemia survival 700<br>Death OR 43.5
Yahia 2012	Egypt	LMIC	Urban	321	Tertiary	Outpatient	Cross sectional study	ARI	2-108 months	unclear	48	-Child Broch 19/48<br>-Child Pneum 19/48
Yalcin 2018	Turkey	UMIC	Mixed	1141	Tertiary	Inpatient	Cross sectional study	ARI	2 - 60 months	SpO2<93%	373	Overall: 20/373
YeLynn 2019	Other: Myanmar	LMIC	Urban	702	Tertiary	Inpatient	Cohort Study	Febrile illness	>= 13 years	SpO2<90%	120	-Adult Fever 25/119<br>Hypoxemia death 14<br>Hypoxemia survive 11<br>Normoxemia death 74<br>Normoxemia survival 19<br>Death OR 0.3
Zampoli 2017	South Africa	UMIC	Urban	1034	Tertiary	Inpatient	Cross sectional study	Pneumonia	<13 years	SpO2<90%	206	-Child Pneum 146/206<br>Hypoxemia death 17<br>Hypoxemia survive 129<br>Normoxemia death 1<br>Normoxemia survival 59<br>Death OR 6.5
Zar 2022	South Africa	UMIC	Urban	1034	Primary	Inpatient	Case control study	ARI	0-1 year	SpO2<90%	439	Child:<br>-All WHO Pneum: 49/201
Zhou 2020	China	UMIC	Unclear	1840	Tertiary	Inpatient	Cross sectional study	COVID 19	18-50 years diagnos	SpO2 <94%	123	Adult:<br>-COVID-19: 4/123

Altitude reported as 'sea level' depicted as 0; N, number of participants; WB Cat, World Bank income category.
Age categorised as: Neon, neonates 0-28 days; Child, children 1 month-17 years; Adult, adults 18 years and older.
Conditions categorised as: NE, neonatal encephalopathy / birth asphyxia; Pneum, pneumonia (WHO classified, radiological, WHO non-severe, WHO severe, other); Prem, prematurity; Asth, asthma; Bronch, bronchiolitis; COVID-19, coronavirus disease caused by the SARS-CoV-2 virus; Diar, diarrhoeal disease; HIV/AIDS, human immunodeficiency-related condition; Mal, malaria; Maln, malnutrition; Mening, meningitis / encephalitis; Seps, sepsis; Anaem, anaemia; Fever, fever.

Table S6: Full list of excluded studies with reason for exclusion

Exclusion reason: Duplicate paper (not picked up during screening)

Commented [HG1]: Should be able to drop these

1.	Paul DMLA, Vega-Briceno LE, Potin SM, Ferres GM, Pulgar BD, Garcia BC, et al. Clinical characterizes of respiratory infection due to Mycoplasma pneumoniae in hospitalized children. Revista Chilena de Infectologia [Internet]. 2009;26(4):343–9. Available from: http://www.scielo.cl/pdf/rci/v26n4/art07.pdf
2.	Riviello E, Talmor D, Novack V, Kiviri W, Twagirumugabe T, Banner-Goodspeed V, et al. Incidence and outcomes of ards in rwanda using modified berlin criteria for resource-poor settings. Critical Care Medicine. 2014;42(12 SUPPL. 1):A1447–8.
3.	Laman M, Ripa P, Vince J, Tefuarani N. Head nodding predicts mortality in young hypoxaemic Papua New Guinean children with acute lower respiratory tract infection. Journal of tropical pediatrics [Internet]. 2013;59(1):75–6. Available from: https://watermark.silverchair.com/fms048.pdf?token=AQECAHi208BE49Ooan9kkhW_Ercy7Dm3ZL_9Cf3qfKAc485ysgAAAqEwggKdBgkqhkiG9w0BBwagggKOMIICigIBADCCAoMGCSqGSIb3DQEHATAeBglghkgBZQMEAS4wEQQM4rcze2jZkX_zWmFlAgEQgIICVMVsuWbE3KZQFaIfkiAPkW2FyZG_ejqJPXwDXaC7zSRKBkOz
4.	Adebola O, Babatunde O, Bose O. Hypoxemia predicts death from severe falciparum malaria among children under 5 years of age in Nigeria: The need for pulse oximetry in case management. African Health Sciences. 2014 Jun 11;14(2):397.

Exclusion reason: Full text not available (after inter-library loan requests and contacting authors)

1.	Azimi A. Hypoxemia in Children. Journal of Comprehensive Pediatrics [Internet]. 2021;13(Supplement):3. Available from: https://brieflands.com/articles/jcp-124167.pdf
2.	Maira D, Duca L, E DP, Consonni D, Grasselli G, Proietti M, et al. Anemia and iron metabolism in patients with sars-cov-2 infection: The role of inflammation and hypoxia. HemaSphere. 2021;5(SUPPL 2):389–90.
3.	Mehic B, Dizdarevic Z, Zutic H. [An addition to the clinical algorithm for treatment of patients with community-acquired pneumonia in Bosnia-Herzegovina: a multicenter prospective study in Sarajevo]. Medicinski arhiv. 1999;53(3 Suppl 3):79–81.
4.	Cespedes Londono J, Fernandez Sarmiento J, Baron Puentes O, Villa Pertuz F, Pieschacon P. Acquired pneumonia's behavior in a community in the pediatrics service at the Fundacion Cardio Infantil, Bogota, Colombia. Saludarte. 2004;3(10):41–55.
5.	Roine I, Vasquez A, Munoz MP, Herrera AM, Banfi A, Perez D, et al. Yearly changes, overuse of drugs and prolonged hospital stay characterize respiratory syncytial virus infections in Santiago, Chile. Saludarte. 2004;3(11):7–16.
6.	Li GQ, Zhang YH. [Clinical features of 77 patients with severe acute respiratory syndrome]. Zhongguo wei zhong bing ji jiu yi xue = Chinese critical care medicine = Zhongguo weizhongbing jijiuyixue. 2003;15(7):404–7.
7.	Hussain SF, Irfan M, Naqi YS, Islam M, Akhtar W. Acute respiratory failure in Pakistani patients: Risk factors associated with mortality. Journal of the College of Physicians and Surgeons Pakistan. 2006;16(4):287–90.
8.	Ben Cheikh A, Ghali H, Bhiri S, Bannour R, Dhouib W, Khefacha S, et al. Clinical characteristics, outcomes, and risk factors for mortality in hospitalized patients with COVID-19 from the start of the pandemic in Sahloul university hospital. Antimicrobial Resistance and Infection Control. 2021;10(SUPPL 1).
9.	Ayinbuomwan SA, Mokogwu N, Akoria OA, Okwara BU, Omuemu CE, Obaseki DE. Arterial Oxygen Saturation and other Clinical Predictors of Survival in Patients with Covid-19: A Review of Cases in a Tertiary Care Hospital in Nigeria. West African journal of medicine. 2021;38(2):109–13.
10.	Liu D, Zhao F, Huang QM, Lyu YB, Zhong WF, Zhou JH, et al. [Effects of oxygen saturation on all-cause mortality among the elderly over 65 years old in 9 longevity areas of China]. Zhonghua yu fang yi xue za zhi [Chinese journal of preventive medicine]. 2021;55(1):45–52.
11.	Khowaja S, Alam J, Mulla M, Alshammary F, Siddiqui AA, Rathore HA, et al. Maxillofacial Fractures Treated in a Dental Department of a Tertiary Care Hospital Karachi, Pakistan: a 5-Year Retrospective Analysis. International Medical Journal [Internet]. 2020;27(6):793–5. Available from: https://search.ebscohost.com/login.aspx?direct=true&db=rzh&AN=147207313&site=ehost-live

Exclusion reason: Incomplete data and unable to obtain data from authors

1.	Keitel K, Kagoro F, Masimba J, Samaka J, Said Z, Temba H, et al. The usefulness of oximetry in triaging febrile children at outpatient level: Experience from a clinical trial in dar es salaam, Tanzania. American Journal of Tropical Medicine and Hygiene [Internet]. 2016;95(5 Supplement 1):554. Available from: http://www.ajtmh.org/deliver/fulltext/14761645/95/5_Suppl/ASTMH16AbstractBook.pdf?itemId=/content/journals/10.4269/ajtmh.abstract2016&mimeType=pdf&containerItemId=content/journals/14761645
2.	Tao H-Q, Zhang J-X, Zou S-C. Clinical characteristics and management of patients with early acute severe pancreatitis: Experience from a medical center in China. World Journal of Gastroenterology [Internet]. 2004;10(6):919–21. Available from: https://www.ncbi.nlm.nih.gov/pmc/articles/PMC4727019/pdf/WJG-10-919.pdf

3. Wandeler G, Pauchard JY, Zangger E, Diawara H, Gehri M. Which clinical signs predict hypoxaemia in young senegalese children with acute lower respiratory tract disease? Paediatrics and International Child Health [Internet]. 2015;35(1):65–8. Available from: http://www.maneyonline.com/doi/pdfplus/10.1179/2046905513Y.0000000111

4. Muhanuzi B, Sawe HR, Kilindimo SS, Mfinanga JA, Weber EJ. Respiratory compromise in children presenting to an urban emergency department of a tertiary hospital in Tanzania: a descriptive cohort study. BMC Emerg Med [Internet]. 2019;19(1):21. Available from: https://www.ncbi.nlm.nih.gov/pmc/articles/PMC6393970/pdf/12873_2019_Article_235.pdf

5. Sawe HR, Reynolds TA, Mfinanga JA, Runyon MS, Murray BL, Wallis LA, et al. The clinical presentation, utilization, and outcome of individuals with sickle cell anaemia presenting to urban emergency department of a tertiary hospital in Tanzania. BMC Hematol [Internet]. 2018;18:25. Available from: https://www.ncbi.nlm.nih.gov/pmc/articles/PMC6142707/pdf/12878_2018_Article_122.pdf

6. Schmedding M, Adegbite BR, Gould S, Beyeme JO, Adegnika AA, Grobusch MP, et al. A Prospective Comparison of Quick Sequential Organ Failure Assessment, Systemic Inflammatory Response Syndrome Criteria, Universal Vital Assessment, and Modified Early Warning Score to Predict Mortality in Patients with Suspected Infection in Gabon. The American journal of tropical medicine and hygiene [Internet]. 2019;100(1):202–8. Available from: https://www.ajtmh.org/downloadpdf/journals/tpmd/100/1/article-p202.pdf

7. Shete S, Nagori G, Nagori P, Hamid M. Relation between pulse oximetry and clinical score in infants with acute bronchiolitis. National Journal of Physiology, Pharmacy and Pharmacology [Internet]. 2014;4(2):124–7. Available from: http://www.scopemed.org/fulltextpdf.php?mno=43744

8. Waitt PI, Mukaka M, Goodson P, SimuKonda FD, Waitt CJ, Feasey N, et al. Sepsis carries a high mortality among hospitalised adults in Malawi in the era of antiretroviral therapy scale-up: a longitudinal cohort study. Journal of Infection. 2015;70(1):11–9.

9. Yang JK, Feng Y, Yuan MY, Yuan SY, Fu HJ, Wu BY, et al. Plasma glucose levels and diabetes are independent predictors for mortality and morbidity in patients with SARS. Diabetic Medicine [Internet]. 2006;23(6):623–8. Available from: https://onlinelibrary.wiley.com/doi/pdfdirect/10.1111/j.1464-5491.2006.01861.x?download=true

10. Sala H, Roca JS, Zerbo C, Garcia R, Cabral G, Fernandez A, et al. Initial clinical management of symptomatic adult patients during influenza A (H1N1) epidemics. Journal of Emergency Medicine. 2011;41(4):435–40.

11. Leung DT, Bogetz J, Itoh M, Ganapathi L, Pietroni MAC, Ryan ET, et al. Factors associated with encephalopathy in patients with Salmonella enterica serotype Typhi bacteremia presenting to a diarrheal hospital in Dhaka, Bangladesh. The American journal of tropical medicine and hygiene [Internet]. 2012;86(4):698–702. Available from: https://www.ajtmh.org/downloadpdf/journals/tpmd/86/4/article-p698.pdf

12. Planting NS, Visser GL, Nicol MP, Workman L, Isaacs W, Zar HJ. Safety and efficacy of induced sputum in young children hospitalised with suspected pulmonary tuberculosis. The international journal of tuberculosis and lung disease : the official journal of the International Union against Tuberculosis and Lung Disease [Internet]. 2014;18(1):8–12. Available from: http://docserver.ingentaconnect.com/deliver/connect/iuatld/10273719/v18n1/s4.pdf?expires=1618470151&id=0000&titleid=3764&checksum=AF214A16177893DBE4D3CCA41B9FAFC8

13. Sigauque B, Roca A, Bassat Q, Morais L, Quinto L, Berenguera A, et al. Severe pneumonia in Mozambican young children: Clinical and radiological characteristics and risk factors. Journal of Tropical Pediatrics [Internet]. 2009;55(6):379–87. Available from: https://watermark.silverchair.com/fmp030.pdf?token=AQECAHi208BE49Ooan9kkhW_Ercy7Dm3ZL_9Cf3qfKAc485ysgAAArowggK2BgkqhkiG9w0BBwagggKnMIICowIBADCCApwGCSqGSIb3DQEHATAeBglghkgBZQMEAS4wEQQMG9TIlbCbzCQOZfZnAgEQgIICbZD2dF6a_JZM17a3ys6er_8dcTV1kJRi22xBSx6f7A939K_y

14. Garcia Hernandez D, Calls Samora M, Llimona Gonzalez A, Dinamarca F, Casanovas F, Perez Oms A, et al. Acute Confusional Syndrome and Covid-19 disease. Clinical and Sociodemographic differences with other comorbid diseases. European Psychiatry. 2022;65(Supplement 1):S483-.

15. Şık N, Şenol HB, Öztürk A, Yılmaz D, Duman M. A reappraisal of childhood drowning in a pediatric emergency department. Am J Emerg Med. 2021;41:90–5.

16. Chinawa AT, Chukwu BF, Chinawa JM, Nduagubam OC, Aronu AE. Correlation between pulse oximetry and the clinical profile of children with acute lower respiratory tract infection. SAJCH South African Journal of Child Health [Internet]. 2022;15(4):198–200. Available from: http://www.sajch.org.za/index.php/SAJCH/article/download/1645/1116

17. Nadjm B, Mtove G, Amos B, Hildenwall H, Najjuka A, Mtei F, et al. Blood glucose as a predictor of mortality in children admitted to the hospital with febrile illness in Tanzania. The American journal of tropical medicine and hygiene [Internet]. 2013;89(2):232–7. Available from: https://www.ajtmh.org/downloadpdf/journals/tpmd/89/2/article-p232.pdf

18. Millman AJ, Greenbaum A, Walaza S, Cohen AL, Groome MJ, Reed C, et al. Development of a respiratory severity score for hospitalized adults in a high HIV-prevalence setting-South Africa, 2010-2011. BMC pulmonary medicine [Internet]. 2017;17(1):28. Available from: https://www.ncbi.nlm.nih.gov/pmc/articles/PMC5288997/pdf/12890_2017_Article_368.pdf

19. Marateb HR, von Cube M, Sami R, Haghjooy Javanmard S, Mansourian M, Amra B, et al. Absolute mortality risk assessment of COVID-19 patients: the Khorshid COVID Cohort (KCC) study. BMC medical research methodology. 2021;21(1):146.

20. Suttapanit K, Wisan M, Sanguanwit P, Prachanukool T. Prognostic Accuracy of VqSOFA for Predicting 28-day Mortality in Patients with Suspected Sepsis in the Emergency Department. Shock (Augusta, Ga). 2021;56(3):368–73.

21. Najafi Z, Zakeri H, Mirhaghi A. The accuracy of acuity scoring tools to predict 24-h mortality in traumatic brain injury patients: A guide to triage criteria. International emergency nursing. 2018;36(101472191):27–33.

22. Kaung M, Kyi TT, Aung NM, Kyaw MP, Min M, Htet ZW, et al. The prognostic utility of bedside assessment of adults hospitalized with malaria in Myanmar: a retrospective analysis. Malaria journal [Internet]. 2015;14(101139802):63. Available from: https://malariajournal.biomedcentral.com/track/pdf/10.1186/s12936-015-0549-y.pdf

23. Yang W, Cao Q, Qin L, Wang X, Cheng Z, Pan A, et al. Clinical characteristics and imaging manifestations of the 2019 novel coronavirus disease (COVID-19):A multi-center study in Wenzhou city, Zhejiang, China. The Journal of infection [Internet]. 2020;80(4):388–93. Available from: https://www.ncbi.nlm.nih.gov/pmc/articles/PMC7102539/pdf/main.pdf

24. Webb C, Ngama M, Ngatia A, Shebbe M, Morpeth S, Mwarumba S, et al. Treatment failure among Kenyan children with severe pneumonia--a cohort study. The Pediatric infectious disease journal [Internet]. 2012;31(9):e152-7. Available from: https://www.ncbi.nlm.nih.gov/pmc/articles/PMC3691501/pdf/emss-52807.pdf

25. Singh A, Khera K, Agarwal J, Awasthi S, Francis JM, Thunga G, et al. Descriptive Analysis of Mortality Predictors in H1n1 Influenza in South Indian Patients. Infectious disorders drug targets. 2017;17(2):106–15.

26. Golcuk Y, Golcuk B, Bilge A, Korkmaz A, Irik M, Hayran M, et al. Prognostic value of serum pregnancy-associated plasma protein A level at the initial ED presentation in elderly patients with CAP. The American journal of emergency medicine. 2015;33(9):1232–6.

Exclusion reason: No original data (review article)

1. Khare C, Gupta A. Hypoxic respiratory failure in small neonates in developing countries – A call for improving service delivery. Tropical Doctor [Internet]. 2022;52(1):220–1. Available from: https://search.ebscohost.com/login.aspx?direct=true&db=rzh&AN=155515869&site=ehost-live

2. Lindtjørn B. Could devolving pneumonia treatment to local health centres reduce mortality in developing countries? Acta Paediatrica [Internet]. 2021;110(2):389–90. Available from: https://search.ebscohost.com/login.aspx?direct=true&db=rzh&AN=148229598&site=ehost-live

3. Mariani J, Tajer C, Antonietti L, Inserra F, Ferder L, Manucha W. High-dose vitamin D versus placebo to prevent complications in COVID-19 patients: A structured summary of a study protocol for a randomised controlled trial (CARED-TRIAL). Trials. 2021;22(1):111.

4. Suffredini DA, Allison MG. A Rationale for Use of High Flow Nasal Cannula for Select Patients With Suspected or Confirmed Severe Acute Respiratory Syndrome Coronavirus-2 Infection. J Intensive Care Med. 2021;36(1):9–17.

5. Mvalo T. Need for routine pulse oximetry and oxygen delivery systems in low-income and middle-income countries. The Lancet Global Health [Internet]. 2022;10(3):e301–2. Available from: http://www.elsevier.com/journals/the-lancet-global-health/2214-109x

6. Bepouka B, Odio O, Mayasi N, Longokolo M, Mangala D, Mandina M, et al. Prevalence and Outcomes of COVID -19 Patients with Happy Hypoxia: A Systematic Review. Infection and Drug Resistance [Internet]. 2022;15((Bepouka, Odio, Mayasi, Longokolo, Mangala, Mandina, Mbula, Kayembe, Situakibanza) Department of Internal Medicine, University of Kinshasa, Kinshasa, Democratic Republic Congo):5619–28. Available from: https://www.dovepress.com/getfile.php?fileID=84156

7. Li-Kim-Moy J, Macartney K. Time to prioritise influenza vaccination in pregnancy. Lancet Infectious Diseases [Internet]. 2021;21(1):8–10. Available from: https://search.ebscohost.com/login.aspx?direct=true&db=rzh&AN=147752272&site=ehost-live

8. McIntyre S, Nelson KB, Mulkey SB, Lechpammer M, Molloy E, Badawi N. Neonatal encephalopathy: Focus on epidemiology and underexplored aspects of etiology. Seminars in Fetal & Neonatal Medicine [Internet]. 2021;26(4):N.PAG-N.PAG. Available from: https://search.ebscohost.com/login.aspx?direct=true&db=rzh&AN=153493449&site=ehost-live

9. Wang X, Li Y, Deloria-Knoll M, Madhi SA, Cohen C, Ali A, et al. Global burden of acute lower respiratory infection associated with human metapneumovirus in children under 5 years in 2018: a systematic review and modelling study. The Lancet Global Health [Internet]. 2021;9(1):e33–43. Available from: http://www.elsevier.com/journals/the-lancet-global-health/2214-109x

10. Noronha SA. Cardiac causes of hypoxia in sickle cell disease. Progress in Pediatric Cardiology [Internet]. 2020;56((Noronha) Division of Pediatric Hematology/Oncology, University of Rochester, Rochester, NY, United States):101192. Available from: https://www.elsevier.com/locate/ppedcard

11. Wick KD, Matthay MA, Ware LB. Pulse oximetry for the diagnosis and management of acute respiratory distress syndrome. The Lancet Respiratory Medicine [Internet]. 2022;10(11):1086–98. Available from: http://www.elsevier.com/journals/the-lancet-respiratory-medicine/2213-2600

12. Rahman AE, Hossain AT, Nair H, Chisti MJ, Dockrell D, Arifeen SE, et al. Prevalence of hypoxaemia in children with pneumonia in low-income and middle-income countries: a systematic review and meta-analysis. The Lancet Global Health [Internet]. 2022;10(3):e348–59. Available from: http://www.elsevier.com/journals/the-lancet-global-health/2214-109x

13. Qazi S. Oxygen therapy for acute respiratory infections in young children. Indian Pediatr [Internet]. 2002; Available from: http://imsear.searo.who.int/handle/123456789/10895

14. Dutta S. Signs of severe illness in young infants. Indian Pediatr [Internet]. 2008; Available from: http://imsear.searo.who.int/handle/123456789/6696

15. Singh M. Monitoring of perinatal asphyxia in the hospital. Indian J Pediatr [Internet]. 1991; Available from: http://imsear.searo.who.int/handle/123456789/78965

16. Mandelzweig K, Leligdowicz A, Murthy S, Lalitha R, Fowler RA, Adhikari NKJ. Non-invasive ventilation in children and adults in low- and low-middle income countries: A systematic review and meta-analysis. J Crit Care. 2018;47:310–9.
17. Zhang L, Mendoza-Sassi R, Santos JCH, Lau J. Accuracy of symptoms and signs in predicting hypoxaemia among young children with acute respiratory infection: A meta-analysis. International Journal of Tuberculosis and Lung Disease [Internet]. 2011;15(3):317–25. Available from: http://docstore.ingenta.com/cgi-bin/ds_deliver/1/u/d/ISIS/61193397.1/iuatld/ijtld/2011/00000015/00000003/art00005/59A449ED38B492271297655209 4F3FDC56111C28EC.pdf?link=http://www.ingentaconnect.com/error/delivery&format=pdf
18. Lam F, Fashanu C, Mekonnen T, Schroder K, Wiwa O. Scaling-up access to oxygen in 3 states in Nigeria: A program evaluation. American Journal of Tropical Medicine and Hygiene [Internet]. 2018;99(4 Supplement):608. Available from: http://www.ajtmh.org/docserver/fulltext/14761645/99/4_Suppl/tropmedabstract2018.pdf?expires=1555047060&id=id&accname=12015&checksum=81160026DD083BF102C66BCBC433A8AE
19. Lozano JM. Epidemiology of hypoxaemia in children with acute lower respiratory infection. International Journal of Tuberculosis and Lung Disease. 2001;5(6):496–504.
20. Hamid MA, Chandna A, Siddiqui S, Fayyaz J. Pulse oximetry: A reliable and cost effective screening tool in children with pneumonia for developing countries. Journal of the Pakistan Medical Association [Internet]. 2016;66(8):1015–8. Available from: http://jpma.org.pk/PdfDownload/7869.pdf
21. Usen S, Weber M. Clinical signs of hypoxaemia in children with acute lower respiratory infection: Indicators of oxygen therapy. International Journal of Tuberculosis and Lung Disease. 2001;5(6):505–10.
22. Zubieta-Calleja G, Zubieta-DeUrioste N. Pneumolysis and “Silent Hypoxemia” in COVID-19. Indian J Clin Biochem. 2021;36(1):112–6.
23. Malik P, Patel U, Patel NH, Somi S, Singh J. Elevated cardiac troponin I as a predictor of outcomes in COVID-19 hospitalizations: a meta-analysis. Le infezioni in medicina. 2020;28(4):500–6.
24. Chew R, Zhang M, Chandna A, Lubell Y. The impact of pulse oximetry on diagnosis, management and outcomes of acute febrile illness in low-income and middle-income countries: a systematic review. BMJ global health. 2021;6(11).
25. Deng J, Peng ZY, Wen ZX, Dong GQ, Xie MX, Xu GG. High COVID-19 mortality in the UK: lessons to be learnt from Hubei province: are under-detected “silent hypoxia” and subsequently low admission rate to blame?. QJM : monthly journal of the Association of Physicians. 2020;113(12):854–5.
26. Rees CA, Basnet S, Gentile A, Gessner BD, Kartasasmita CB, Lucero M, et al. An analysis of clinical predictive values for radiographic pneumonia in children. BMJ Glob Health [Internet]. 2020;5(8). Available from: https://gh.bmj.com/content/bmjgh/5/8/e002708.full.pdf
27. Behl L, Grover N, Kaushik SL. Perinatal and neonatal mortality--a hospital based study. Indian Pediatr [Internet]. 1998; Available from: http://imsear.searo.who.int/handle/123456789/7100
28. di Fiore JM, Vento M. Intermittent hypoxemia and oxidative stress in preterm infants. Respiratory physiology & neurobiology. 2019;266:121–9.
29. Subhi R, Adamson M, Campbell H, Weber M, Smith K, Duke T. The prevalence of hypoxaemia among ill children in developing countries: a systematic review. The Lancet Infectious Diseases. 2009;9(4):219–27.
30. Gupta S, Gazendam N, Farina JM, Saldarriaga C, Mendoza I, López-Santi R, et al. Malaria and the Heart: JACC State-of-the-Art Review. Journal of the American College of Cardiology (JACC) [Internet]. 2021;77(8):1110–21. Available from: https://search.ebscohost.com/login.aspx?direct=true&db=rzh&AN=148925339&site=ehost-live
31. Howie SR, Ebruke BE, Gil M, Bradley B, Nyassi E, Edmonds T, et al. The development and implementation of an oxygen treatment solution for health facilities in low and middle-income countries. Journal of Global Health [Internet]. 2020;10(2):20425. Available from: https://search.ebscohost.com/login.aspx?direct=true&db=rzh&AN=153545047&site=ehost-live
32. Guo L, Jin Z, Gan TJ, Wang E. Silent Hypoxemia in Patients with COVID-19 Pneumonia: A Review. Medical science monitor : international medical journal of experimental and clinical research. 2021;27(dxw, 9609063):e930776-.
33. Srivastava S, Garg I, Bansal A, Kumar B. SARS-CoV-2 infection: physiological and environmental gift factors at high altitude. Virusdisease. 2020;31(4):450–2.
34. Graham HR, Olojede OE, Bakare AAA, McCollum ED, Iuliano A, Isah A, et al. Pulse oximetry and oxygen services for the care of children with pneumonia attending frontline health facilities in Lagos, Nigeria (INSPIRING-Lagos): Study protocol for a mixed-methods evaluation. BMJ Open [Internet]. 2022;12(5):e058901-. Available from: http://bmjopen.bmj.com/content/early/by/section

Exclusion reason: Incomplete data and not indicated to contact author

1. Aznab M, N ER, Moazen H. Clinical Characteristics and Risk Factors of COVID-19 in 60 Adult Cancer Patients. Clinical Medicine Insights: Oncology [Internet]. 2022;16((Aznab) Internal Medicine Department, Kermanshah University of Medical Sciences, Kermanshah, Iran, Islamic Republic of(Eskandari Roozbahani) Clinical Research Development Center, Imam Reza Hospital, Kermanshah University of Medical Sciences, Kermanshah, I). Available from: https://journals.sagepub.com/home/onc
2. Loh LC, Khoo SK, Quah SY, Visvalingam V, Radhakrishnan A, Vijayasingham P, et al. Adult community-acquired pneumonia in Malaysia: prediction of mortality from severity assessment on admission. Respirology (Carlton, Vic). 2004;9(3):379–86.

3. Obgandze TN, Nemsadze KP, Chkhaidze IG, Peradze DI. [Effectiveness of treatment of bronchoobstruction in children with acute respiratory infections using home-made spacer]. Georgian medical news. 2005;(118):46–9.
4. Graham SM, Mtitimila EI, Kamanga HS, Walsh AL, Hart CA, Molyneux ME. Clinical presentation and outcome of Pneumocystis carinii pneumonia in Malawian children. Lancet (London, England). 2000;355(9201):369–73.
5. Li X, Marmar T, Xu Q, Tu J, Yin Y, Tao Q, et al. Predictive indicators of severe COVID-19 independent of comorbidities and advanced age: a nested case-control study. Epidemiology and infection [Internet]. 2020;148(epi, 8703737):e255-. Available from: https://www.cambridge.org/core/services/aop-cambridge-core/content/view/A6D145A3AF3873B79F0C27A380EF4D48/S0950268820002502a.pdf/div-class-title-predictive-indicators-of-severe-covid-19-independent-of-comorbidities-and-advanced-age-a-nested-case-control-st
6. Gupta B, D'Souza N, Sawhney C, Farooque K, Kumar A, Agrawal P, et al. Analyzing fat embolism syndrome in trauma patients at AIIMS Apex Trauma Center, New Delhi, India. J Emerg Trauma Shock. 2011;4(3):337–41.
7. Waheed S, Baig MA, Khursheed M, Awan S. Impact of heat waves on patients presenting to the emergency department of a tertiary care hospital - A single center cross-sectional study. J Pak Med Assoc. 2019;69(5):741–4.
8. Bag Soytas R, Unal D, Arman P, Suzan V, Emiroglu Gedik T, Can G, et al. Factors affecting mortality in geriatric patients hospitalized with COVID-19. Turkish journal of medical sciences. 2021;51(2):454–63.
9. Bakamutumaho B, Cummings MJ, Owor N, Kayiwa J, Namulondo J, Byaruhanga T, et al. Severe COVID-19 in uganda across two epidemic phases: A prospective cohort study. American Journal of Tropical Medicine and Hygiene [Internet]. 2021;105(3):740–4. Available from: https://www.ajtmh.org/view/journals/tpmd/105/3/article-p740.xml
10. Becerra-Munoz VM, Nunez-Gil IJ, Eid CM, Garcia Aguado M, Romero R, Huang J, et al. Clinical profile and predictors of in-hospital mortality among older patients hospitalised for COVID-19. Age and ageing. 2021;50(2):326–34.
11. Cisse FA, Ligot N, Conde K, Barry DS, Toure LM, Konate M, et al. Predictors of stroke favorable functional outcome in Guinea, results from the Conakry stroke registry. Scientific reports. 2022;12(1):1125.
12. Cordova E, Mykietiuk A, Sued O, de Vedia L, Pacifico N, Garcia Hernandez MH, et al. Clinical characteristics and outcomes of hospitalized patients with SARS-CoV-2 infection in a Latin American country: Results from the ECCOVID multicenter prospective study. PloS one. 2021;16(10):e0258260-.
13. Custodio ACD, Ribas F v, Toledo L v, J de CC, Lima LM, C de FBA. Hospitalizations and mortality by severe acute respiratory syndrome: Comparison between the pre-pandemic and pandemic periods. Revista Brasileira de Epidemiologia [Internet]. 2021;24((Custodio, Ribas) Universidade Federal de Vicosa, Vicosa (MG), Brazil(Toledo, de Carvalho, Lima, Freitas) Department of Medicine and Nursing, Universidade Federal de Vicosa, Vicosa (MG), Brazil):E210052-. Available from: http://www.scielo.br/scielo.php/script_sci_serial/pid_1415-790X/lng_en/nrm_iso
14. da Silveira WC, Ramos LEF, Silva RT, de Paiva BBM, Pereira PD, Schwarzbold AV, et al. Predictors of venous thromboembolism in COVID-19 patients: results of the COVID-19 Brazilian Registry. Internal and emergency medicine. 2022;17(7):1863–78.
15. Darabi A, Dehghanfard M, Jozan S, Tahmasebi R, Movahed A, Zamani M, et al. Investigating the association between allergic diseases and COVID-19 in 400 Iranian patients. Allergologia et immunopathologia. 2021;49(5):9–15.
16. Dillon K, Hook C, Coupland Z, Avery P, Taylor H, Lockyer A. Pre-hospital lowest recorded oxygen saturation independently predicts death in patients with COVID-19. Br Paramed J. 2020;5(3):59–65.
17. Dominguez-Rojas JA, Vasquez-Hoyos P, Perez-Morales R, Monsalve-Quintero AM, Mora-Robles L, Diaz-Diaz A, et al. Association of Cancer Diagnosis and Therapeutic Stage With Mortality in Pediatric Patients With COVID-19, Prospective Multicenter Cohort Study From Latin America. Frontiers in Pediatrics [Internet]. 2022;10((Dominguez-Rojas) Pediatric Critical Care, Hospital Edgardo Rebagliati Martins, Red Colaborativa Pediatrica de Latinoamerica, Lima, Peru(Vasquez-Hoyos) Pediatric Critical Care, Hospital de San Jose, Red Colaborativa Pediatrica de Latinoamerica, Bogota, Co):885633. Available from: https://www.frontiersin.org/journals/pediatrics
18. Ershov VI, Belkin AA, Gorbachev VI, Gritsan AI, Zabolotskikh IB, Lebedinskii KM, et al. [Russian multicenter observational clinical study <>: a comparative analysis of the outcomes of stroke]. Zhurnal nevrologii i psikhiatrii imeni SS Korsakova. 2022;122(3. Vyp. 2):22–30.
19. Esteban I, Polack FP, Bergero G, Alves C, Bronstein M, Ziegler V, et al. Asymptomatic COVID-19 in the elderly: Dementia and viral clearance as risk factors for disease progression. Gates Open Research [Internet]. 2022;5((Esteban, Polack, Bergero, Alves, Bronstein, Ziegler, Caballero, Libster) INFANT Foundation, Buenos Aires, Argentina(Wood, Perez Marc) Hospital Militar Central, Buenos Aires, Argentina(Caballero) Consejo Nacional de Investigaciones Cientificas y Tecnicas):143. Available from: https://gatesopenresearch.org/
20. Gokhale Y, Mehta R, Kulkarni U, Karnik N, Gokhale S, Sundar U, et al. Tocilizumab improves survival in severe COVID-19 pneumonia with persistent hypoxia: a retrospective cohort study with follow-up from Mumbai, India. BMC infectious diseases. 2021;21(1):241.
21. Gupta N, John A, Kokkottil MS, Varma M, Umakanth S, Saravu K. Clinical profile and outcomes of asymptomatic vs. symptomatic travellers diagnosed with COVID-19: An observational study from a coastal town in South India. Drug Discoveries and Therapeutics [Internet]. 2021;15(1):1–8. Available from: https://www.ddtjournal.com/downloadpdf/2046
22. Jouffroy R, Lemoine S, Derkenne C, Kedzierewicz R, Scannavino M, Bertho K, et al. Prehospital management of acute respiratory distress in suspected COVID-19 patients. The American journal of emergency medicine. 2021;45(aa2, 8309942):410–4.

23. Khalaf JM, Hussein II, Al-Nimer MS. Aminophylline as anti-hypoxic add-on therapy in the management of COVID-19 in Baghdad: An experience from single center report case study. Journal of Research in Pharmacy [Internet]. 2021;25(6):852–6. Available from: http://jrespharm.com/uploads/pdf/pdf_MPJ_969.pdf

24. Khan MAS, Hasan MJ, Rashid MU, Sagar SK, Khan S, Zaman S, et al. Factors associated with in-hospital mortality of adult tetanus patients-a multicenter study from Bangladesh. PLoS Neglected Tropical Diseases [Internet]. 2022;16(3):e0010235-. Available from: https://journals.plos.org/plosntds/article/file?id=10.1371/journal.pntd.0010235&type=printable

25. Khan MSI, Debnath CR, Nath PN, Mahtab MA, Nabeka H, Matsuda S, et al. Ivermectin Treatment May Improve the Prognosis of Patients With COVID-19. Archivos de Bronconeumologia [Internet]. 2020;56(12):828–30. Available from: http://www.elsevier.com/wps/find/journaldescription.cws_home/710346/description#description

26. Lazar Neto F, Salzstein GA, Cortez AL, Bastos TL, Baptista FVD, Moreira JA, et al. Comparative assessment of mortality risk factors between admission and follow-up models among patients hospitalized with COVID-19. International journal of infectious diseases : IJID : official publication of the International Society for Infectious Diseases. 2021;105(c3r, 9610933):723–9.

27. Li G, Wu X, Zhou CL, Wang YM, Song B, Cheng XB, et al. Uric acid as a prognostic factor and critical marker of COVID-19. Scientific reports. 2021;11(1):17791.

28. Li J, Chen Y, Chen S, Wang S, Zhang D, Wang J, et al. Derivation and validation of a prognostic model for predicting in-hospital mortality in patients admitted with COVID-19 in Wuhan, China: the PLANS (platelet lymphocyte age neutrophil sex) model. BMC infectious diseases. 2020;20(1):959.

29. Liang C, Mao X, Niu H, Dong F, Chen Y, Huang K, et al. Characteristics, management and in-hospital clinical outcomes among inpatients with acute exacerbation of chronic obstructive pulmonary disease in china: Results from the phase i data of acure study. International Journal of COPD [Internet]. 2021;16((Liang, Mao, Huang, Zhang) Chinese Alliance for Respiratory Diseases in Primary Care, Beijing, China(Niu, Huang, Dong, Zhan, Yang, Wang) Department of Pulmonary and Critical Care Medicine, China-Japan Friendship Hospital, Beijing, China(Niu, Huang, Dong,):451–65. Available from: https://www.dovepress.com/getfile.php?fileID=67069

30. Long L, Wu L, Chen L, Zhou D, Wu H, Lu D, et al. Effect of early oxygen therapy and antiviral treatment on disease progression in patients with COVID-19: A retrospective study of medical charts in China. PLoS Neglected Tropical Diseases [Internet]. 2021;15(1):1–15. Available from: https://journals.plos.org/plosntds/article?id=10.1371/journal.pntd.0009051

31. Mahmud R, Rahman MM, Rassel MA, Monayem FB, S.K.J.B. S, Islam MS, et al. Post-COVID-19 syndrome among symptomatic COVID-19 patients: A prospective cohort study in a tertiary care center of Bangladesh. PLoS ONE [Internet]. 2021;16(4 April):e0249644-. Available from: https://journals.plos.org/plosone/article/file?id=10.1371/journal.pone.0249644&type=printable

32. Kaplan NM, Dove W, Abu-Zeid AF, Shamoon HE, Abd-Eldayem SA, Hart CA. Evidence of human metapneumovirus infection in Jordanian children. Saudi Medical Journal [Internet]. 2006;27(7):1081–3. Available from: http://www.smj.org.sa/PDFFiles/Jul06/Briefcomm.pdf

33. Mei J, Hu W, Chen Q, Li C, Chen Z, Fan Y, et al. Development and external validation of a COVID-19 mortality risk prediction algorithm: a multicentre retrospective cohort study. BMJ open. 2020;10(12):e044028-.

34. Muro RP, Masoza TS, Kasanga G, Kayange N, Kidenya BR. Predictors and outcome of first line treatment failure among under-five children with community acquired severe pneumonia at Bugando Medical Centre, Mwanza, Tanzania: A prospective cohort study. PloS one. 2020;15(12):e0243636-.

35. Nakkazi E. Oxygen supplies and COVID-19 mortality in Africa. The Lancet Respiratory medicine. 2021;9(4):e39-.

36. Pozzobon FM, Perazzo H, Bozza FA, Rodrigues RS, de Mello Perez R, Chindamo MC. Liver injury predicts overall mortality in severe COVID-19: a prospective multicenter study in Brazil. Hepatology international. 2021;15(2):493–501.

37. Raffee LA, Alawneh KZ, K ASM, Ibdah RK, Rawashdeh SI, A.-H.W. AM. An observational study of the occurrence of acute coronary syndrome (ACS) among jordanian patients: Identifying the influence of Ramadan Fasting. Annals of Medicine and Surgery [Internet]. 2020;59((Raffee, Al Suleiman) Department of Accident and Emergency Medicine, Faculty of Medicine, Jordan University of Science and Technology, Irbid, Jordan(Alawneh) Department of Diagnostic Radiology and Nuclear Medicine, Faculty of Medicine, Jordan University o):171–5. Available from: http://www.elsevier.com/journals/annals-of-medicine-and-surgery/2049-0801

38. Russell FM, Wang A, Ehrman RR, Jacobs J, Croft A, Larsen C. Risk factors associated with hospital admission in COVID-19 patients initially admitted to an observation unit. Am J Emerg Med. 2021;46:339–43.

39. Shafiekhani S, Rafiei S, Abdollahzade S, Souri S, Moomeni Z. Risk Factors Associated with In-Hospital Mortality in Iranian Patients with COVID-19: Application of Machine Learning. Polish Journal of Medical Physics and Engineering [Internet]. 2022;28(1):19–29. Available from: http://www.degruyter.com/view/j/pjmpe

40. Stefan G, Mehedinti AM, Andreiana I, Zugravu AD, Cinca S, Busuioc R, et al. Clinical features and outcome of maintenance hemodialysis patients with COVID-19 from a tertiary nephrology care center in Romania. Renal failure. 2021;43(1):49–57.

41. Vahedi E, Ghanei M, Ghazvini A, Azadi H, Izadi M, Panahi Y, et al. The clinical value of two combination regimens in the Management of Patients Suffering from Covid-19 pneumonia: a single centered, retrospective, observational study. Daru : journal of Faculty of Pharmacy, Tehran University of Medical Sciences. 2020;28(2):507–16.

42. Valentania V, Somasetia DH, Hilmanto D, Setiabudi D, Nataprawira HMN. Modified PIRO (predisposition, insult, response, organ dysfunction) severity score as a predictor for mortality of children with pneumonia in Hasan Sadikin Hospital, Bandung, Indonesia. Multidisciplinary Respiratory Medicine [Internet]. 2021;16((Valentania, Somasetia, Hilmanto, Setiabudi, Nataprawira) Department of

Child Health, Faculty of Medicine, Universitas Padjadjaran/Dr. Hasan Sadikin General Hospital, Bandung, Indonesia):735. Available from: https://mrmjournal.org/mrm/article/view/735

43. Wang Y, Shu H, Liu H, Li X, Zhou X, Zou X, et al. The peak levels of highly sensitive troponin I predicts in-hospital mortality in COVID-19 patients with cardiac injury: a retrospective study. European heart journal Acute cardiovascular care. 2021;10(1):6–15.

44. Yanamandra U, Shobhit S, Paul D, Aggarwal B, Kaur P, Duhan G, et al. Relationship of Computed Tomography Severity Score With Patient Characteristics and Survival in Hypoxemic COVID-19 Patients. Cureus. 2022;14(3):e22847-.

45. Yang Y, Zhu XF, Huang J, Chen C, Zheng Y, He W, et al. Nomogram for prediction of fatal outcome in patients with severe COVID-19: a multicenter study. Military Medical Research. 2021;8(1):21.

46. Yeshaneh A, Tadele B, Dessalew B, Alemayehu M, Wolde A, Adane A, et al. Incidence and predictors of mortality among neonates referred to comprehensive and specialized hospitals in Amhara regional state, North Ethiopia: a prospective follow-up study. Italian journal of pediatrics. 2021;47(1):186.

47. Zar HJ, Barnett W, Stadler A, Gardner-Lubbe S, Myer L, Nicol MP. Aetiology of childhood pneumonia in a well vaccinated South African birth cohort: a nested case-control study of the Drakenstein Child Health Study. The Lancet Respiratory Medicine [Internet]. 2016;4(6):463–72. Available from: https://www.sciencedirect.com/science/article/pii/S2213260016000965

48. Moschovis PP, Addo-Yobo EOD, Banajeh S, Chisaka N, Christiani DC, Hayden D, et al. Stunting is associated with poor outcomes in childhood pneumonia. Tropical medicine & international health : TM & IH [Internet]. 2015;20(10):1320–8. Available from: https://onlinelibrary.wiley.com/doi/pdfdirect/10.1111/tmi.12557?download=true

49. Rodrigues RM, Schvartsman BGS, Farhat SCL, Schvartsman C. Hypotonic solution decreases serum sodium in infants with moderate bronchiolitis. Acta paediatrica (Oslo, Norway : 1992) [Internet]. 2014;103(3):e111-5. Available from: https://onlinelibrary.wiley.com/doi/pdfdirect/10.1111/apa.12493?download=true

50. Sun H, Ning R, Tao Y, Yu C, Deng X, Zhao C, et al. Risk Factors for Mortality in 244 Older Adults With COVID-19 in Wuhan, China: A Retrospective Study. Journal of the American Geriatrics Society [Internet]. 2020;68(6):E19–23. Available from: https://www.ncbi.nlm.nih.gov/pmc/articles/PMC7267277/pdf/JGS-9999-na.pdf

51. Gharebaghi N, Farshid S, Boroofeh B, Nejadrahim R, Mousavi J, Dindarian S, et al. Evaluation of epidemiology, clinical features, prognosis, diagnosis and treatment outcomes of patients with COVID-19 in West Azerbaijan Province. International Journal of Clinical Practice [Internet]. 2021;75(6):e14108-. Available from: http://onlinelibrary.wiley.com/journal/10.1111/(ISSN)1742-1241

52. Simoes E Silva AC, Vasconcelos MA, Colosimo EA, Mendonca ACQ, Martelli-Junior H, Silva LR, et al. Outcomes and risk factors of death among hospitalized children and adolescents with obesity and COVID-19 in Brazil: An analysis of a nationwide database. Pediatric obesity. 2022;17(9):e12920-.

53. Park J, Ahn S, Lee S, Song J, Moon S, Kim J, et al. Association of ischemia modified albumin with mortality in qSOFA positive sepsis patients by sepsis-3 in the emergency department. The American journal of emergency medicine. 2021;44(aa2, 8309942):72–7.

54. Leeyaphan J, Charoenpong L, Prasithsirikul W, Prasithsirikul S. Clinical Characteristics and Treatment Outcomes of Mild COVID-19 Patients in Field Hospital: A Cross-Sectional Study. Journal of the Medical Association of Thailand [Internet]. 2022;105(9):799–805. Available from: http://www.jmatonline.com/index.php/jmat/article/viewfile/13589/10639

55. Duan J, Wang X, Chi J, Chen H, Bai L, Hu Q, et al. Correlation between the variables collected at admission and progression to severe cases during hospitalization among patients with COVID-19 in Chongqing. Journal of medical virology. 2020;92(11):2616–22.

56. Ghosh T, Suri TM, Jat KR, Gupta AK, Bhatnagar S, Tiwari P, et al. Clinical profile and in-hospital outcomes of COVID-19 among adolescents at a tertiary care hospital in India. Lung India [Internet]. 2022;39(4):343–7. Available from: http://www.lungindia.com

57. UlHaq Z, Shahzad M, Khattak MI, Fazid S, Ullah N, Shireen A, et al. Clinical Characteristics, Mortality and Associated risk factors in COVID-19 patients reported in ten major hospitals of Khyber Pakhtunkhwa, Pakistan. Journal of Ayub Medical College, Abbottabad : JAMC. 2020;32(Suppl 1)(4):S633–9.

58. Chisti MJ, Sarker SA, Shahunja KM, A.S.M.S.B. S, Sharifuzzaman, Hasan MI, et al. Seizure in Children under Five Presenting with Pneumonia in a Critical Care Ward in Bangladesh: Prevalence, Associated Factors, and Outcome. Pediatric Infectious Disease Journal [Internet]. 2021;((Chisti, Sarker, Shahunja, Shahid, Sharifuzzaman, Hasan, Nuzhat, Kabir, Afroze, Alam, Shahrin, Ahmed) International Centre for Diarrhoeal Disease Research, Bangladesh (Icddr,b), Dhaka, Bangladesh):389–93. Available from: http://journals.lww.com/pidj

59. Linli Z, Chen Y, Tian G, Guo S, Fei Y. Identifying and quantifying robust risk factors for mortality in critically ill patients with COVID-19 using quantile regression. The American journal of emergency medicine. 2021;45(aa2, 8309942):345–51.

60. Hueda-Zavaleta M, Copaja-Corzo C, Bardales-Silva F, Flores-Palacios R, Barreto-Rocchetti L, Benites-Zapata VA. Factors associated with mortality due to COVID-19 in patients from a public hospital in Tacna, Peru. Revista peruana de medicina experimental y salud publica. 2021;38(2):214–23.

61. Erturk Sengel B, Tukenmez Tigen E, Ilgin C, Basari T, Bedir M, Odabasi Z, et al. Application of CALL score for prediction of progression risk in patients with COVID-19 at university hospital in Turkey. International Journal of Clinical Practice [Internet]. 2021;75(10):e14642-. Available from: http://onlinelibrary.wiley.com/journal/10.1111/(ISSN)1742-1241

62. Shander A, Javidroozi M, Sentilhes L. Tranexamic acid and obstetric hemorrhage: give empirically or selectively? International Journal of Obstetric Anesthesia [Internet]. 2021;48:N.PAG-N.PAG. Available from: https://search.ebscohost.com/login.aspx?direct=true&db=rzh&AN=153160640&site=ehost-live

63. Turan O, B AY, Turan PA, Mirici A. Clinical characteristics and outcomes of hospitalized COVID-19 patients with COPD. Expert Review of Respiratory Medicine [Internet]. 2021;15(8):1069–76. Available from: http://www.tandfonline.com/loi/ierx20

64. de Souza FSH, Hojo-Souza NS, Batista BD de O, da Silva CM, Guidoni DL. On the analysis of mortality risk factors for hospitalized COVID-19 patients: A data-driven study using the major Brazilian database. PloS one. 2021;16(3):e0248580-.

65. Zhang Y, Chen Q, Yao L, Chen J, Yu X. Study on Laboratory Indicators Related to Lung Injury of Corona Virus Disease 2019. Clinical laboratory. 2021;67(1).

66. da Silva Ramos FJ, Rezende de Freitas FG, Machado FR, Freitas FGR de, de Freitas FGR. Sepsis in patients hospitalized with coronavirus disease 2019: how often and how severe? Current Opinion in Critical Care [Internet]. 2021;27(5):474–9. Available from: https://search.ebscohost.com/login.aspx?direct=true&db=rzh&AN=152934060&site=ehost-live

67. Torres Jimenez AR, Ruiz Vela N, Cespedes Cruz AI, Velazquez Cruz A, Bernardino Gonzalez AK. Shrinking lung syndrome in pediatric systemic lupus erythematosus. Lupus. 2021;30(7):1175–9.

68. El Ghoul J, Bendayekh A, Fki W, Yengui I, Ferjani S, Milouchi S, et al. [Risk factors for hospital mortality during pulmonary embolism]. Annales de cardiologie et d'angeiologie. 2020;69(1):7–11.

69. Pigoga JL, Omer YO, Wallis LA. Derivation of a Contextually-Appropriate COVID-19 Mortality Scale for Low-Resource Settings. Annals of global health. 2021;87(1):31.

70. Ackah M, Gazali Salifu M, Osei Yeboah C. Estimated incidence and case fatality rate of traumatic brain injury among children (0-18 years) in Sub-Saharan Africa. A systematic review and meta-analysis. PloS one. 2021;16(12):e0261831-.

71. Pradeepkumar V. Diabetes and Covid Associated Mucormucormycosis-A Cross Sectional Study in Kilpauk Medical College. The Journal of the Association of Physicians of India. 2022;70(4):11–2.

72. Nematswerani N, Collie S, Chen T, Cohen M, Champion J, Feldman C, et al. The impact of routine pulse oximetry use on outcomes in COVID-19-infected patients at increased risk of severe disease: A retrospective cohort analysis. South African medical journal = Suid-Afrikaanse tydskrif vir geneeskunde. 2021;111(10):950–6.

73. Tutuncu EE, Ozturk B, Gurbuz Y, Haykir A, Sencan I, Kuscu F, et al. Clinical characteristics of 74 pandemic H1N1 influenza patients from Turkey. Risk factors for fatality. Saudi medical journal. 2010;31(9):993–8.

74. Ak C, Sayar S, Polat ZP, Kilic ET, Ozdil K. Clinical and laboratory factors associated with severe disease course in Turkish patients with COVID-19 infection. Iranian Red Crescent Medical Journal [Internet]. 2021;23(2):e283-. Available from: http://ircmj.org/index.php/IRCMJ/issue/archive

75. Li Y, Li H, Song C, Lu R, Zhao Y, Lin F, et al. Early Prediction of Disease Progression in Patients with Severe COVID-19 Using C-Reactive Protein to Albumin Ratio. Disease markers. 2021;2021(dim, 8604127):6304189.

76. Gulec-Balbay E, Altundal MMB, Kaypak MK, Cangur S, Kaya S. Clinical Characteristics Predicting Mortality Risk in Hospitalized Geriatric Patients with COVID-19 Pneumonia: a Retrospective Study. Clinical laboratory. 2022;68(8).

77. Hasan MJ, Rabbani R, Anam AM, Huq SMR, Polash MMI, Nessa SST, et al. Impact of high dose of baricitinib in severe COVID-19 pneumonia: a prospective cohort study in Bangladesh. BMC infectious diseases. 2021;21(1):427.

78. Tang H, Tu C, Xiong F, Sun X, Tian JB, Dong JW, et al. Risk factors for the mortality of hemodialysis patients with COVID-19: A multicenter study from the overall hemodialysis population in Wuhan. Seminars in dialysis. 2022;35(1):71–80.

79. Yang C, Liu F, Liu W, Cao G, Liu J, Huang S, et al. Myocardial injury and risk factors for mortality in patients with COVID-19 pneumonia. International journal of cardiology. 2021;326(gqw, 8200291):230–6.

80. Jalili E, Keramat F, Bashirian S, Khazaei S, Talebi-Ghane E, Karami M, et al. Demographic and clinical features association with mortality in patients with covid-19: A cross-sectional study in the west of Iran. Acta Medica Iranica [Internet]. 2021;59(10):587–94. Available from: https://acta.tums.ac.ir/index.php/acta/article/download/9105/5583

81. Li T, Wang X, Zhuang X, Wang H, Li A, Huang L, et al. Baseline characteristics and changes of biomarkers in disease course predict prognosis of patients with COVID-19. Internal and emergency medicine. 2021;16(5):1165–72.

82. Lopes RD, Macedo AVS, de Barros E Silva PGM, Moll-Bernardes RJ, dos Santos TM, Mazza L, et al. Effect of Discontinuing vs Continuing Angiotensin-Converting Enzyme Inhibitors and Angiotensin II Receptor Blockers on Days Alive and Out of the Hospital in Patients Admitted With COVID-19: A Randomized Clinical Trial. JAMA. 2021;325(3):254–64.

83. Osaikhuwuomwan J, Ezeanochie M, Uwagboe C, Ndukwu K, Yusuf S, Ande A. Clinical characteristics and outcomes for pregnant women diagnosed with COVID-19 disease at the University of Benin Teaching Hospital, Benin City, Nigeria. The Pan African medical journal. 2021;39(101517926):134.

84. Jamora RDG, Prado MB, Anlacan VMM, Sy MCC, Espiritu AI, Prado MBJ. Incidence and risk factors for stroke in patients with COVID-19 in the Philippines: An analysis of 10,881 cases. Journal of Stroke & Cerebrovascular Diseases [Internet]. 2022;31(11):N.PAG-N.PAG. Available from: https://search.ebscohost.com/login.aspx?direct=true&db=rzh&AN=159821006&site=ehost-live

85.	Hormanstorfer M, Ragusa MA, Poggio L, Moreira-Facundo J, Orellana-Villa Z, Bobrowski FA, et al. Development of Simple and Sensitive Score to Assess the Risk of Pneumonia in COVID-19 Patients. Revista de investigacion clinica; organo del Hospital de Enfermedades de la Nutricion. 2020;73(1):52–8.
86.	Duenas-Castell C, Borre-Naranjo D, Rodelo D, Lora L, Almanza A, Coronell W, et al. Changes in Oxygenation and Clinical Outcomes with Awake Prone Positioning in Patients with Suspected COVID-19 In Low-Resource Settings: A Retrospective Cohort Study. Journal of Intensive Care Medicine [Internet]. 2021;36(11):1347–53. Available from: https://journals.sagepub.com/home/jic
87.	Li X, Wu Z, Xue M, Du W. Smoking status affects clinical characteristics and disease course of acute exacerbation of chronic obstructive pulmonary disease: A prospectively observational study. Chronic respiratory disease. 2020;17(101197408):1479973120916184-.
88.	Mecheril Balachandran D, Karuppusamy D, Kumar Maurya D, Sekhar Kar S, Keepanasseril A. Indicators for maternal near miss: an observational study, India. Bulletin of the World Health Organization [Internet]. 2022;100(7):436–46. Available from: https://search.ebscohost.com/login.aspx?direct=true&db=rzh&AN=157963613&site=ehost-live
89.	Jroundi I, Benmessaoud R, Mahraoui C, Moraleda C, Tligui H, Seffar M, et al. Antibiotic usage prior and during hospitalization for clinical severe pneumonia in children under five years of age in Rabat, Morocco. Antibiotics [Internet]. 2013;2(4):450–64. Available from: http://www.mdpi.com/2079-6382/2/4/450/pdf
90.	Duzenli Kar Y, Akln F, Sert A, Arslan S. Pulmonary Hypertension in Children with Lower Respiratory Tract Infections in the Konya Province of Turkey. Journal of Pediatric Infectious Diseases [Internet]. 2020;15(2):95–101. Available from: https://www.thieme-connect.com/products/ejournals/journal/10.1055/s-00029028

Exclusion reason: Wrong outcomes (no SpO2 at time of presentation/admission)

1.	ADITOMO E, D/enny D. POS-920 MORTALITY RATE OF COVID 19 IN END STAGE RENAL DISEASE WITH HEMODIALYSIS ROUTINE TREATMENT AT MARDI WALUYO HOSPITAL LAMPUNG INDONESIA, OCTOBER 2020-AUGUST 2021. Kidney International Reports. 2022;7(2 Supplement):S401–2.
2.	Castro EM, Limpin M. The Value of Inflammatory Markers in Predicting the Severity of Hypoxemia Among COVID 19 Patients at Philippine Heart Center. American Journal of Respiratory and Critical Care Medicine [Internet]. 2022;205(1). Available from: https://www.atsjournals.org/doi/abs/10.1164/ajrccm-conference.2022.205.1_MeetingAbstracts.A4582
3.	Kumar M, Murugan T, Lionel A, Thomas M, Mannam P, Yoganathan S. Management of children and adolescents with Wilson Disease and neurological worsening following D–Penicillamine therapy: A single centre experience. Annals of Indian Academy of Neurology [Internet]. 2022;25(4):698–702. Available from: https://search.ebscohost.com/login.aspx?direct=true&db=rzh&AN=159193259&site=ehost-live
4.	Ijaz M, Jaffar Khan M, Khan J, Usama U. Association of clinical characteristics of patients presenting with influenza like illness or severe acute respiratory illness with development of acute respiratory distress syndrome. Monaldi archives for chest disease = Archivio Monaldi per le malattie del torace. 2017;87(1):765.
5.	da Costa LG v, Carmona MJC, Malbouisson LM, Rizoli S, Rocha-Filho JA, Cardoso RG, et al. Independent early predictors of mortality in polytrauma patients: a prospective, observational, longitudinal study. Clinics (Sao Paulo, Brazil). 2017;72(8):461–8.
6.	Beane A, de Silva AP, de Silva N, Sujeewa JA, Rathnayake RMD, Sigera PC, et al. Evaluation of the feasibility and performance of early warning scores to identify patients at risk of adverse outcomes in a low-middle income country setting. BMJ open [Internet]. 2018;8(4):e019387-. Available from: https://www.ncbi.nlm.nih.gov/pmc/articles/PMC5922475/pdf/bmjopen-2017-019387.pdf
7.	Wang K, Kang S, Tian R, Zhang X, Wang Y. Imaging manifestations and diagnostic value of chest CT of coronavirus disease 2019 (COVID-19) in the Xiaogan area. Clinical radiology [Internet]. 2020;75(5):341–7. Available from: https://www.ncbi.nlm.nih.gov/pmc/articles/PMC7118637/pdf/main.pdf
8.	Zheng S, Zou Q, Wang X, Bao J, Yu F, Guo F, et al. Factors associated with fatality due to avian influenza A(H7N9) infection in China. Clinical Infectious Diseases [Internet]. 2020;71(1):125–32. Available from: http://cid.oxfordjournals.org/content/by/year
9.	Leon-Abarca JA, Accinelli RA. Hypoxia predicts lower respiratory tract diseases in adults living at high altitudes. American Journal of Respiratory and Critical Care Medicine [Internet]. 2019;199(9). Available from: https://www.atsjournals.org/doi/abs/10.1164/ajrccm-conference.2019.199.1_MeetingAbstracts.A4921
10.	Ajayi MT, Oladokun RA, Falade AG. Antibiotic treatment of community acquired pneumonia in well-nourished young Nigerian children. Journal of Tropical Pediatrics [Internet]. 2005;51(5):319–20. Available from: https://watermark.silverchair.com/fmi021.pdf?token=AQECAHi208BE49Ooan9kkhW_Ercy7Dm3ZL_9Cf3qfKAc485ysgAAAqIwggKeBgkqhkiG9w0BBwagggKPMIICiwIBADCCAoQGCSqGSIb3DQEHATAeBglghkgBZQMEAS4wEQQMvCuX_Q641ohEfs_TAgEQgIICVc5Q3kGAyePsAWI1lTaMp0Nbh1z7-yoR5cZjF32hSrnvfZve
11.	Akdur G, Das M, Bardakci O, Akman C, Siddikoglu D, Akdur O, et al. Prediction of mortality in COVID-19 through combing CT severity score with NEWS, qSOFA, or peripheral perfusion index. The American journal of emergency medicine. 2021;50(aa2, 8309942):546–52.
12.	Babenko D, Seidullayeva A, Bayesheva D, Turdalina B, Omarkulov B, Almabayeva A, et al. Ability of Procalcitonin and C-Reactive Protein for Discriminating between Bacterial and Enteroviral Meningitis in Children Using Decision Tree. BioMed Research International [Internet]. 2021;1–7. Available from: https://search.ebscohost.com/login.aspx?direct=true&db=rzh&AN=151718513&site=ehost-live

13. Bakare AA, Graham H, Ayede AI, Peel D, Olatinwo O, Oyewole OB, et al. Providing oxygen to children and newborns: a multi-faceted technical and clinical assessment of oxygen access and oxygen use in secondary-level hospitals in southwest Nigeria. Int Health [Internet]. 2020;12(1):60–8. Available from: https://www.ncbi.nlm.nih.gov/pmc/articles/PMC6964224/pdf/ihz009.pdf

14. Barreto-Filho JA, Seabra-Garcez JD, Garcez FB, Moreira TS, Drager LF. Nondyspnogenic acute hypoxemic respiratory failure in COVID-19 pneumonia. J Appl Physiol (1985). 2021;130(3):892–7.

15. Buss IM, Birkhamshaw E, Innes MA, Magadoro I, Waitt PI, Rylance J. Validating a novel index (SWAT-Bp) to predict mortality risk of community-acquired pneumonia in Malawi. Malawi medical journal : the journal of Medical Association of Malawi [Internet]. 2018;30(4):230–5. Available from: https://www.ajol.info/index.php/mmj/article/download/181958/171341

16. Cao J, Li M, Li J, Xu J, Shi Z, Ti J, et al. Effect of fiberoptic bronchoscope combined with ambroxol alveolar lavage on the treatment of pulmonary atelectasis in severe pneumonia and its influences on systemic inflammatory response and respiratory mechanics. International Journal of Clinical and Experimental Medicine [Internet]. 2020;13(8):5901–7. Available from: http://www.ijcem.com/files/ijcem0113914.pdf

17. Cardona-Perez JA, Villegas-Mota I, Helguera-Repetto AC, Acevedo-Gallegos S, Rodriguez-Bosch M, Aguinaga-Rios M, et al. Prevalence, clinical features, and outcomes of SARS-CoV-2 infection in pregnant women with or without mild/moderate symptoms: Results from universal screening in a tertiary care center in Mexico City, Mexico. PLoS ONE [Internet]. 2021;16(4 April):e0249584-. Available from: https://journals.plos.org/plosone/article/file?id=10.1371/journal.pone.0249584&type=printable

18. Chang Y, Wan X, Fu X, Yang Z, Lu Z, Wang Z, et al. Severe versus common COVID-19: an early warning nomogram model. Aging. 2022;14(2):544–56.

19. Chhetri UD, Shrestha S, Pradhan R, Shrestha A, Adhikari N, Thorson S, et al. Clinical profile of invasive pneumococcal disease in Patan hospital, Nepal. Kathmandu University Medical Journal [Internet]. 2011;9(33):45–9. Available from: http://www.kumj.com.np/issue/33/45-49.pdf

20. Chisti MJ, Ahmed T, Bardhan PK, Salam MA. Evaluation of simple laboratory investigations to predict fatal outcome in infants with severe malnutrition presenting in an urban diarrhoea treatment centre in Bangladesh. Tropical medicine & international health : TM & IH [Internet]. 2010;15(11):1322–5. Available from: https://onlinelibrary.wiley.com/doi/pdfdirect/10.1111/j.1365-3156.2010.02619.x?download=true

21. Dabalo ML, S AB, G BG, S LF, L AS, W FB, et al. Perinatal Asphyxia and Its Associated Factors among Live Births in the Public Health Facilities of Bahir Dar City, Northwest Ethiopia, 2021. International Journal of Pediatrics (United Kingdom) [Internet]. 2021;2021((Dabalo, Abdisa Sori) School of Nursing and Midwifery, Haramaya University, College of Health and Medical Sciences, Harar, Ethiopia(Animen Bante, Belay Gela, Lake Fanta, Feyisa Balcha, Derebe Tesfahun) Department of Midwifery, Bahir Dar University, Colleg):3180431. Available from: https://www.hindawi.com/journals/ijpedi/

22. Del Carpio-Orantes L, Garcia-Mendez S, Zamudio-Severino GM, Sanchez-Diaz JS, Navarrete-Espinosa B, Rivera-Vinas MA, et al. Baricitinib in Patients with Severe Pneumonia due to COVID-19 in Veracruz, Mexico. Open Forum Infectious Diseases. 2021;8(SUPPL 1):S350–1.

23. Ding S, Xu Y, Yue H, Pan Z, Sun B, Zheng G, et al. Outcome of neonatal hypoxemic respiratory failure: a livebirth population-based retrospective survey. BMC Pediatrics [Internet]. 2022;22(1):552. Available from: http://www.biomedcentral.com/bmcpediatr/

24. Duke T, Pulsan F, Panauwe D, Hwaihwanje I, Sa'avu M, Kaupa M, et al. Solar-powered oxygen, quality improvement and child pneumonia deaths: a large-scale effectiveness study. Archives of disease in childhood. 2021;106(3):224–30.

25. Ekeh B, Ogunniyi A, Isamade E, Ekrikpo U. Stroke mortality and its predictors in a Nigerian teaching hospital. African health sciences [Internet]. 2015;15(1):74–81. Available from: https://www.ncbi.nlm.nih.gov/pmc/articles/PMC4370132/pdf/AFHS1501-0074.pdf

26. Fan X, Zhu B, Nouri-Vaskeh M, Jiang C, Feng X, Poulsen K, et al. Scores based on neutrophil percentage and lactate dehydrogenase with or without oxygen saturation predict hospital mortality risk in severe COVID-19 patients. Virology journal. 2021;18(1):67.

27. Frade MAC, Filho FB, Q de LAL, B de MM, Lugao HB. Asymptomatic low pulse oximetry measurements in leprosy patients in the time of COVID-19: Dapsone side effect. Revista da Sociedade Brasileira de Medicina Tropical [Internet]. 2022;55((Frade, Filho, de Lima, Lugao) Universidade de Sao Paulo, Faculdade de Medicina de Ribeirao Preto, Departamento de Clinica Medica, Divisao de Dermatologia, SP, Ribeirao Preto, Brazil(Frade, Filho, Lima, Lugao) Universidade de Sao Paulo, Faculdade de Me):e0491-2021. Available from: https://www.scielo.br/j/rsbmt/a/pZZMYYqKwbNMWTdLrjtXfgv/?format=pdf&lang=en

28. Gao H, Sun X, Li W, Gao Q, Zhang J, Zhang Y, et al. Development and validation of a risk score to predict 30-day mortality in patients with atrial fibrillation-related stroke: GPS-GF score. Neurological research. 2018;40(7):532–40.

29. Gelaw B, Mulatu G, Tesfa G, Marew C, Chekole B, Alebel A. Magnitude and associated factors of virological failure among children on ART in Bahir Dar Town public health facilities, Northwest Ethiopia: a facility based cross-sectional study. Italian Journal of Pediatrics [Internet]. 2021;47(1):1–9. Available from: https://search.ebscohost.com/login.aspx?direct=true&db=rzh&AN=149671621&site=ehost-live

30. Getaneh Y, Fekadu E, Jemere AT, Mengistu Z, Tarekegn GE, Oumer M. Incidence and determinants of adverse outcomes among women who were managed for eclampsia in the University of Gondar Comprehensive Specialized Hospital, Northwest Ethiopia. BMC Pregnancy & Childbirth [Internet]. 2021;21(1):1–12. Available from: https://search.ebscohost.com/login.aspx?direct=true&db=rzh&AN=153315167&site=ehost-live

31. Ghimire R, Shakya YM, Shrestha TM, Neupane RP. The utility of red cell distribution width to predict mortality of septic patients in a tertiary hospital of Nepal. BMC emergency medicine. 2020;20(1):43.

32. Girma L, Geteneh A, Amenu D, Kassa T. Isolation and characterization of Listeria monocytogenes among women attending Jimma University medical center, Southwest Ethiopia. BMC Infectious Diseases [Internet]. 2021;21(1):1–6. Available from: https://search.ebscohost.com/login.aspx?direct=true&db=rzh&AN=150892197&site=ehost-live

33. Gong X, Kang S, Guo X, Li Y, Gao H, Yuan Y. Associated risk factors with disease severity and antiviral drug therapy in patients with COVID-19. BMC Infectious Diseases [Internet]. 2021;21(1):549. Available from: http://www.biomedcentral.com/bmcinfectdis/

34. Guan X, Zhang B, Fu M, Li M, Yuan X, Zhu Y, et al. Clinical and inflammatory features based machine learning model for fatal risk prediction of hospitalized COVID-19 patients: results from a retrospective cohort study. Annals of medicine. 2021;53(1):257–66.

35. Gwer S, Chengo E, Newton CRJC, Kirkham FJ. Unexpected relationship between tympanometry and mortality in children with nontraumatic coma. Pediatrics [Internet]. 2013;132(3):e713-7. Available from: https://pediatrics.aappublications.org/content/132/3/e713.long

36. Hasan MM, Saha KK, Yunus RM, Alam K. Prevalence of acute respiratory infections among children in India: Regional inequalities and risk factors. Maternal & Child Health Journal [Internet]. 2022;26(7):1594–602. Available from: https://search.ebscohost.com/login.aspx?direct=true&db=rzh&AN=157306335&site=ehost-live

37. Hasan S, Webby RJ, Iqbal M, Rashid H bin, Ahmad M ud D, Nazir J, et al. Sentinel surveillance for influenza A viruses in Lahore District Pakistan in flu season 2015-2016. BMC Infectious Diseases [Internet]. 2022;22(1):1–15. Available from: https://search.ebscohost.com/login.aspx?direct=true&db=rzh&AN=154533332&site=ehost-live

38. Huang Q, Lei Y, Xing W, He C, Wei G, Miao Z, et al. Evaluation of Pulmonary Edema Using Ultrasound Imaging in Patients With COVID-19 Pneumonia Based on a Non-local Channel Attention ResNet. Ultrasound in Medicine and Biology [Internet]. 2022;48(5):945–53. Available from: https://www.elsevier.com/locate/ultrasmedbio

39. Jakka S, Veena S, Rao ARM, Eisenhut M. Cerebrospinal fluid adenosine deaminase levels and adverse neurological outcome in pediatric tuberculous meningitis. Infection [Internet]. 2005;33(4):264–6. Available from: https://link.springer.com/article/10.1007%2Fs15010-005-5005-4

40. James V, Prakash A, Mehta K, Durugappa T. Re-thinking treatment strategies for febrile neutropenia in paediatric oncology population: the perspective from a developing country. Infectious Agents & Cancer [Internet]. 2021;16(1):1–8. Available from: https://search.ebscohost.com/login.aspx?direct=true&db=rzh&AN=150988556&site=ehost-live

41. Jayashree M, KiranBabu HB, Singhi S, Nallasamy K. Use of Nasal Bubble CPAP in Children with Hypoxemic Clinical Pneumonia-Report from a Resource Limited Set-Up. J Trop Pediatr [Internet]. 2016;62(1):69–74. Available from: https://www.ncbi.nlm.nih.gov/pmc/articles/PMC4935781/pdf/fmv063.pdf

42. Joosse P, Soedarmo S, Luitse JS, Ponsen KJ. Trauma outcome analysis of a Jakarta University Hospital using the TRISS method: validation and limitation in comparison with the major trauma outcome study. Trauma and Injury Severity Score. The Journal of trauma. 2001;51(1):134–40.

43. Kaewarpai T, Ekchariyawat P, Phunpang R, Wright SW, Dulsuk A, Moonmueangsan B, et al. Longitudinal profiling of plasma cytokines in melioidosis and their association with mortality: a prospective cohort study. Clinical microbiology and infection : the official publication of the European Society of Clinical Microbiology and Infectious Diseases. 2020;26(6):783.e1-783.e8.

44. Kakaje A, Alhalabi MM, Ghareeb A, Karam B, Mansour B, Zahra B, et al. Interactions of Consanguinity and Number of Siblings with Childhood Acute Lymphoblastic Leukemia. BioMed Research International [Internet]. 2020;1–11. Available from: https://search.ebscohost.com/login.aspx?direct=true&db=rzh&AN=147476805&site=ehost-live

45. Kang L, Cui X, Fu J, Wang W, Li L, Li T, et al. Clinical characteristics of 967 children with pertussis: a single-center analysis over an 8-year period in Beijing, China. European Journal of Clinical Microbiology and Infectious Diseases [Internet]. 2022;41(1):9–20. Available from: https://link.springer.de/link/service/journals/10096/index.htm

46. Kayange N, Kamugisha E, Mwizamholya DL, Jeremiah S, Mshana SE. Predictors of positive blood culture and deaths among neonates with suspected neonatal sepsis in a tertiary hospital, Mwanza-Tanzania. BMC pediatrics [Internet]. 2010;10(100967804):39. Available from: https://bmcpediatr.biomedcentral.com/track/pdf/10.1186/1471-2431-10-39.pdf

47. Kebudi R, Kurucu N, Tugcu D, Hacisalihoglu S, Fisgin T, Ocak S, et al. COVID-19 infection in children with cancer and stem cell transplant recipients in Turkey: A nationwide study. Pediatric Blood and Cancer [Internet]. 2021;68(6):e28915-. Available from: http://onlinelibrary.wiley.com/journal/10.1002/(ISSN)1545-5017

48. Keepanasseril A, Subburaj SP, Nayak D, Bojja V, Chakkalakkoombil SV, Nair PP. Risk factors of intracranial haemorrhage in preeclampsia: a case-control study. Neurological Sciences [Internet]. 2022;43(10):6003–10. Available from: https://search.ebscohost.com/login.aspx?direct=true&db=rzh&AN=159103462&site=ehost-live

49. Khadka P, Maharjan G, Chapagain G, JanukaThapaliya, Paudyal P. Economic and Diagnostic Biomarker Tests of Neonatal Sepsis: A Prospective Study from a Tertiary Care Hospital in a Low-Income Country. BioMed Research International [Internet]. 2022;1–9. Available from: https://search.ebscohost.com/login.aspx?direct=true&db=rzh&AN=160435382&site=ehost-live

50. Khanum I, Samar F, Fatimah Y, Safia A, Adil A, Kiren H, et al. Role of awake prone positioning in patients with moderate-to-severe COVID-19: an experience from a developing country. Monaldi archives for chest disease = Archivio Monaldi per le malattie del torace. 2021;91(2).

51. Kortz TB, Nyirenda J, Tembo D, Elfving K, Baltzell K, Bandawe G, et al. Distinct Biomarker Profiles Distinguish Malawian Children with Malarial and Non-malarial Sepsis. The American journal of tropical medicine and hygiene [Internet]. 2019;101(6):1424–33. Available from: https://www.ajtmh.org/downloadpdf/journals/tpmd/101/6/article-p1424.pdf

52. Koul PA, Khan UH, Asad R, Yousuf R, Broor S, Lal RB, et al. Contribution of influenza to acute exacerbations of chronic obstructive pulmonary disease in Kashmir, India, 2010-2012. Influenza and other Respiratory Viruses [Internet]. 2015;9(1):40–2. Available from: http://onlinelibrary.wiley.com/journal/10.1111/(ISSN)1750-2659

53. Kuster GW, Dutra LA, Brasil IP, Pacheco EP, Arruda MJC, Volcov C, et al. Performance of four ischemic stroke prognostic scores in a Brazilian population. Arquivos de neuro-psiquiatria [Internet]. 2016;74(2):133–7. Available from: http://www.scielo.br/pdf/anp/v74n2/0004-282X-anp-74-02-0133.pdf

54. Li S, Liu J, Chen F, Cai K, Tan J, Xie W, et al. A risk score based on pediatric sequential organ failure assessment predicts 90-day mortality in children with Klebsiella pneumoniae bloodstream infection. BMC infectious diseases [Internet]. 2020;20(1):916. Available from: https://www.ncbi.nlm.nih.gov/pmc/articles/PMC7709332/pdf/12879_2020_Article_5644.pdf

55. Li Y, Li Q, Zhang G, Ma H, Wu Y, Yi Q, et al. Time to positivity of blood culture is a risk factor for clinical outcomes in Staphylococcus aureus bacteremia children: a retrospective study. BMC infectious diseases [Internet]. 2019;19(1):437. Available from: https://www.ncbi.nlm.nih.gov/pmc/articles/PMC6525363/pdf/12879_2019_Article_3993.pdf

56. Liao H, Ou S, Dong X, Liu J, Xiao C. Association of Etoricoxib treatment and incident hypoxia in patients with aortic dissection undergoing endovascular aortic repair. Biomedicine and Pharmacotherapy [Internet]. 2021;139((Liao, Ou, Dong, Liu, Xiao) Department of Cardiology, The Third People's Hospital of Huizhou, The Affiliated Hospital of Guangzhou Medical University, Huizhou, Guangdong, China):111625. Available from: https://www.journals.elsevier.com/biomedicine-and-pharmacotherapy

57. Lisicic-Konakovic M, Kulasevic A, Lokvancic-Bekto A. Clinical characteristics of COVID-19 among children in Sarajevo, Bosnia and Herzegovina. Central European Journal of Paediatrics [Internet]. 2021;17(2):128–34. Available from: https://cejpaediatrics.com/index.php/cejp/article/view/410

58. Liu C, Li L, Song K, Zhan ZY, Yao Y, Gong H, et al. A nomogram for predicting mortality in patients with COVID-19 and solid tumors: a multicenter retrospective cohort study. Journal for immunotherapy of cancer [Internet]. 2020;8(2). Available from: https://jitc.bmj.com/content/jitc/8/2/e001314.full.pdf

59. Liu L, Chen Z, Du Y, Gao J, Li J, Deng T, et al. CD8+ T cells predicted the conversion of common covid-19 to severe. Scientific reports. 2021;11(1):2169.

60. Maranatha D, Mawardi, Hamzah. Severe pneumonia: Etiology and outcome in a tertiary hospital in Indonesia. Indian Journal of Forensic Medicine and Toxicology [Internet]. 2021;15(2):2192–200. Available from: http://medicopublication.com/index.php/ijfmt/article/download/14698/13309

61. Mohammed Z, Saleh Y, AbdelSalam EM, Mohammed NBB, El-Bana E, Hirshon JM. Evaluation of the Revised Trauma Score, MGAP, and GAP scoring systems in predicting mortality of adult trauma patients in a low-resource setting. BMC Emergency Medicine [Internet]. 2022;22(1):1–10. Available from: https://search.ebscohost.com/login.aspx?direct=true&db=rzh&AN=157132760&site=ehost-live

62. Nakiyingi L, Bwanika JM, Ssengooba W, Mubiru F, Nakanjako D, Joloba ML, et al. Chest X-ray interpretation does not complement Xpert MTB/RIF in diagnosis of smear-negative pulmonary tuberculosis among TB-HIV co-infected adults in a resource-limited setting. BMC infectious diseases. 2021;21(1):63.

63. Nascimento IJB do, Pinto LR, Fernandes VA, Romero IM, Oliveira JA de Q, Marcolino MS, et al. Clinical characteristics and outcomes among Brazilian patients with severe acute respiratory syndrome coronavirus 2 infection: an observational retrospective study. Sao Paulo medical journal = Revista paulista de medicina. 2020;138(6):490–7.

64. Nigussie J, Girma B, Molla A, Tamir T, Tilahun R. Magnitude of postpartum hemorrhage and its associated factors in Ethiopia: a systematic review and meta-analysis. Reproductive Health [Internet]. 2022;19(1):1–13. Available from: https://search.ebscohost.com/login.aspx?direct=true&db=rzh&AN=155688085&site=ehost-live

65. Norton EB, Archibald LK, Nwanyanwu OC, Kazembe PN, Dobbie H, Reller LB, et al. Clinical predictors of bloodstream infections and mortality in hospitalized Malawian children. The Pediatric infectious disease journal. 2004;23(2):145.

66. Nwanna-Nzewunwa O, Ngamby MK, Cox J, Feldhaus I, Motwani G, Monono ME, et al. Epidemiology and cost of pediatric injury in Yaoundé, Cameroon: a prospective study. European Journal of Trauma & Emergency Surgery [Internet]. 2020;46(6):1403–12. Available from: https://search.ebscohost.com/login.aspx?direct=true&db=rzh&AN=147225355&site=ehost-live

67. Omran D, al Soda M, Bahbah E, Esmat G, Shousha H, Elgebaly A, et al. Predictors of severity and development of critical illness of Egyptian COVID-19 patients: A multicenter study. PloS one. 2021;16(9):e0256203-.

68. Osman A, Via G, Sallehuddin RM, Ahmad AH, Fei SK, Azil A, et al. Helmet continuous positive airway pressure vs. high flow nasal cannula oxygen in acute cardiogenic pulmonary oedema: a randomized controlled trial. European heart journal Acute cardiovascular care. 2021;10(10):1103–11.

69. Pal S, Sardar S, Sarkar N, Ghosh M, Chatterjee S. Effect of Antenatal Dexamethasone in Late Preterm Period on Neonatal Hypoglycemia: A Prospective Cohort Study from a Developing Country. Journal of Tropical Pediatrics [Internet]. 2022;68(2):1–11. Available from: https://search.ebscohost.com/login.aspx?direct=true&db=rzh&AN=156085904&site=ehost-live

70. Permana H, Huang I, Susandi E, Wisaksana R. The association of admission random blood glucose concentration and body-mass index with mortality in COVID-19 patients. European review for medical and pharmacological sciences. 2021;25(22):7144–50.

71. Peterson E, Lo KB, DeJoy R, Salacup G, Pelayo J, Bhargav R, et al. The relationship between coronary artery disease and clinical outcomes in COVID-19: a single-center retrospective analysis. Coronary artery disease. 2021;32(5):367–71.

72. Petroni G, Quaglino M, Lujan S, Kovalevski L, Rondina C, Videtta W, et al. Early prognosis of severe traumatic brain injury in an urban argentinian trauma center. The Journal of trauma. 2010;68(3):564–70.

73. Pho Y, Nhem S, Sok C, By B, Phann D, Nob H, et al. Melioidosis in patients with suspected tuberculosis in Cambodia: a single-center cross-sectional study. The international journal of tuberculosis and lung disease : the official journal of the International Union against Tuberculosis and Lung Disease [Internet]. 2018;22(12):1481–5. Available from: http://docserver.ingentaconnect.com/deliver/connect/iuatld/10273719/v22n12/s16.pdf?expires=1618470143&id=0000&titleid=3764&checksum=8E0C37C2267B6CACDC648FEF251A048F

74. Pons MJ, Ymana B, Mayanga-Herrera A, Saenz Y, Alvarez-Erviti L, Tapia-Rojas S, et al. Cytokine Profiles Associated With Worse Prognosis in a Hospitalized Peruvian COVID-19 Cohort. Frontiers in Immunology [Internet]. 2021;12((Pons, Ymana, Ugarte-Gil) Grupo Enfermedades Emergentes, Universidad Cientifica del Sur, Lima, Peru(Mayanga-Herrera, Tapia-Rojas) Laboratorio de Cultivo Celular e Inmunologia, Universidad Cientifica del Sur, Lima, Peru(Saenz) Area de Microbiologia Molecul):700921. Available from: https://www.frontiersin.org/journals/immunology#

75. Pulsan F, Duke T. Response to oxygen therapy using oxygen concentrators run off solar power in children with respiratory distress in remote primary health facilities in Papua New Guinea. Trop Doct. 2021;51(1):15–9.

76. Rahman A, Hossain MdM. Prevalence and determinants of fever, ARI and diarrhea among children aged 6-59 months in Bangladesh. BMC Pediatrics [Internet]. 2022;22(1):1–12. Available from: https://search.ebscohost.com/login.aspx?direct=true&db=rzh&AN=155688216&site=ehost-live

77. Rahman AE, Ameen S, Hossain AT, Perkins J, Jabeen S, Majid T, et al. Introducing pulse oximetry for outpatient management of childhood pneumonia: An implementation research adopting a district implementation model in selected rural facilities in Bangladesh. eClinicalMedicine [Internet]. 2022;50((Rahman, Perkins, Cunningham, Dockrell, Nair, Campbell) NIHR Global Health Research Unit on Respiratory Health (RESPIRE), Usher Institute, The University of Edinburgh, UK, Edinburgh, United Kingdom(Rahman, Ameen, Hossain, Jabeen, Majid, Uddin, Shaikh, Ahm):101511. Available from: https://www.thelancet.com/journals/eclinm/issue/current

78. Rao SG, Paramesh RC, Bansal A, Shukla D, Sadashiva N, Saini J. A prospective computed tomography study of maxillofacial injuries in patients with head injury. European Journal of Trauma & Emergency Surgery [Internet]. 2022;48(4):2529–38. Available from: https://search.ebscohost.com/login.aspx?direct=true&db=rzh&AN=158431624&site=ehost-live

79. Roshanaei G, Kiumarsi A, Kasaeian A, Safari M, Abbasi M, Rahimi A. Influential factors on survival in gastric cancer: A single-center study. Journal of Research in Medical Sciences [Internet]. 2022;27(3):19. Available from: https://search.ebscohost.com/login.aspx?direct=true&db=rzh&AN=156245908&site=ehost-live

80. Salah A, Al-Subol I, Hudna A, Alhaj A, Alqubaty AR, Farie W, et al. Neonatal sepsis in Sana'a city, Yemen: a predominance of Burkholderia cepacia. BMC Infectious Diseases [Internet]. 2021;21(1):1–10. Available from: https://search.ebscohost.com/login.aspx?direct=true&db=rzh&AN=153239919&site=ehost-live

81. Salva O, Doreski PA, Giler CS, Quinodoz DC, Guzman LG, Munoz SE, et al. Reversal of SARS-CoV2-Induced Hypoxia by Nebulized Sodium Ibuprofenate in a Compassionate Use Program. Infectious diseases and therapy. 2021;10(4):2511–24.

82. Sami R, Soltaninejad F, Shayganfar A, Mirfendereski S, Mansourian M, Khademi N, et al. Severity of Disease and COVID-19 Complications During Hospital Stay: A Prospective Cohort Study. Archives of Iranian medicine. 2022;25(6):383–93.

83. Şan İ, Yıldırım Ç, Bekgöz B, Gemcioğlu E. Transport of awake hypoxemic probable COVID 19 patients in the prone position. Am J Emerg Med. 2021;46:420–3.

84. Shams S, Tafaroji J, Aghaali M, Ahmadi N, Heydari H, Nasab SDM, et al. Prevalence of enteric adenovirus and co-infection with rotavirus in children under 15 years of age with gastroenteritis in Qom, Iran. Gastroenterology & Hepatology from Bed to Bench [Internet]. 2022;15(3):256–62. Available from: https://search.ebscohost.com/login.aspx?direct=true&db=rzh&AN=159878589&site=ehost-live

85. Sharma K, Mishra A, Singh H, Thinlas T, Pasha MAQ. Differential methylation in EGLN1 associates with blood oxygen saturation and plasma protein levels in high-altitude pulmonary edema. Clinical Epigenetics [Internet]. 2022;14(1):123. Available from: http://www.springer.com/biomed/human+genetics/journal/13148

86. Sominina A, Danilenko D, Komissarov A, Pisareva M, Musaeva T, Bakaev M, et al. Age-Specific Etiology of Severe Acute Respiratory Infections and Influenza Vaccine Effectivity in Prevention of Hospitalization in Russia, 2018-2019 Season. Journal of Epidemiology and Global Health [Internet]. 2021;11(4):413–25. Available from: https://www.atlantis-press.com/journals/jegh

87. Sun S, Pan Y, Bai L, Zhao X, Liu L, Li H, et al. GWTG Risk Model for All Stroke Types Predicts In-Hospital and 3-Month Mortality in Chinese Patients with Acute Stroke. Journal of stroke and cerebrovascular diseases : the official journal of National Stroke Association. 2019;28(3):800–6.

88. Sunil B, Nithya E. Correlation of oxygen saturation index and oxygenation index in hypoxemic respiratory failure among neonates. Journal of Clinical and Diagnostic Research [Internet]. 2021;15(8):SC06–8. Available from: https://www.jcdr.net/article_fulltext.asp?issn=0973-709x&year=2021&volume=15&issue=8&page=SC06&issn=0973-709x&id=15237

89. Surme S, Buyukyazgan A, Bayramlar OF, Cinar AK, Copur B, Zerdali E, et al. Predictors of Intensive Care Unit Admission or Mortality in Patients with Coronavirus Disease 2019 Pneumonia in Istanbul, Turkey. Japanese journal of infectious diseases. 2021;74(5):458–64.

90. Tabernero E, Ruiz LA, Espana PP, Mendez R, Serrano L, Santos B, et al. COVID-19 in young and middle-aged adults: predictors of poor outcome and clinical differences. Infection. 2022;50(1):179–89.

91. Tibaijuka L, Bawakanya SM, Owaraganise A, Kyasimire L, Kumbakumba E, Boatin AA, et al. Incidence and predictors of preterm neonatal mortality at Mbarara Regional Referral Hospital in South Western Uganda. PLoS ONE [Internet]. 2021;16(November):e0259310-. Available from: https://journals.plos.org/plosone/article/file?id=10.1371/journal.pone.0259310&type=printable

92. Uzel Sener M, Yildiz M, Kavurgaci S, Ozturk Ergur F, Sener A, Ozturk A. Evaluation of mortality predictors in hospitalized COVID-19 patients: Retrospective cohort study. Tuberkuloz ve toraks. 2021;69(2):196–206.

93. Vicco MH, Ferini F, Rodeles L, Scholtus P, Long AK, Musacchio HM. In-hospital mortality risk factors in community acquired pneumonia: evaluation of immunocompetent adult patients without comorbidities. Revista da Associacao Medica Brasileira (1992) [Internet]. 2015;61(2):144–9. Available from: http://www.scielo.br/pdf/ramb/v61n2/0104-4230-ramb-61-02-0144.pdf

94. Vidal-Cevallos P, Higuera-De-La-Tijera F, Chavez-Tapia NC, Sanchez-Giron F, Cerda-Reyes E, Rosales-Salyano VH, et al. Lactate-dehydrogenase associated with mortality in hospitalized patients with COVID-19 in Mexico: a multi-centre retrospective cohort study. Annals of hepatology. 2021;24(101155885):100338.

95. Vizheh M, Muhidin S, Aghajani F, Maleki Z, Bagheri F, Hosamirudsari H, et al. Characteristics and outcomes of COVID-19 pneumonia in pregnancy compared with infected nonpregnant women. International journal of gynaecology and obstetrics: the official organ of the International Federation of Gynaecology and Obstetrics. 2021;153(3):462–8.

96. Wang J, Guo S, Zhang Y, Gao K, Zuo J, Tan N, et al. Clinical features and risk factors for severe inpatients with COVID-19: A retrospective study in China. PloS one. 2020;15(12):e0244125-.

97. Wang K, Zuo P, Liu Y, Zhang M, Zhao X, Xie S, et al. Clinical and Laboratory Predictors of In-hospital Mortality in Patients With Coronavirus Disease-2019: A Cohort Study in Wuhan, China. Clinical infectious diseases : an official publication of the Infectious Diseases Society of America. 2020;71(16):2079–88.

98. Winskill P, Hogan AB, Thwing J, Mwandigha L, Walker PGT, Lambert B. Health inequities and clustering of fever, acute respiratory infection, diarrhoea and wasting in children under five in low- and middle-income countries: a Demographic and Health Surveys analysis. BMC Medicine [Internet]. 2021;19(1):1–11. Available from: https://search.ebscohost.com/login.aspx?direct=true&db=rzh&AN=151270620&site=ehost-live

99. Wolter N, Cohen C, Tempia S, Walaza S, Moosa F, Plessis M du, et al. Epidemiology of Pertussis in Individuals of All Ages Hospitalized With Respiratory Illness in South Africa, January 2013—December 2018. Clinical Infectious Diseases [Internet]. 2021;73(3):e745–53. Available from: https://search.ebscohost.com/login.aspx?direct=true&db=rzh&AN=151699097&site=ehost-live

100. Xie X, Li Z, Xu H, Peng D, Yin L, Meng R, et al. Non-Fatal Drowning Risk Prediction Based on Stacking Ensemble Algorithm. Children [Internet]. 2022;9(9):N.PAG-N.PAG. Available from: https://search.ebscohost.com/login.aspx?direct=true&db=rzh&AN=159336194&site=ehost-live

101. Xie Y, Li B, Lin Y, Shi F, Chen W, Wu W, et al. Combining Blood-Based Biomarkers to Predict Mortality of Sepsis at Arrival at the Emergency Department. Medical science monitor : international medical journal of experimental and clinical research. 2021;27(dxw, 9609063):e929527-.

102. Yang Y, Li X, Birkhead GS, Zheng Z, Lu JH. Clinical indices and mortality of hospitalized avian influenza A (H7N9) patients in Guangdong, China. Chinese medical journal [Internet]. 2019;132(3):302–10. Available from: https://www.ncbi.nlm.nih.gov/pmc/articles/PMC6595816/pdf/cm9-132-302.pdf

103. Zeng Z, Wu C, Lin Z, Ye Y, Feng S, Fang Y, et al. Development and validation of a simple-to-use nomogram to predict the deterioration and survival of patients with COVID-19. BMC infectious diseases. 2021;21(1):356.

104. Zhan N, Guo Y, Tian S, Huang B, Tian X, Zou J, et al. Clinical characteristics of COVID-19 complicated with pleural effusion. BMC infectious diseases. 2021;21(1):176.

105. Zhang W, Zheng Y, Feng X, Chen M, Kang Y. Systemic inflammatory response syndrome in Sepsis-3: a retrospective study. BMC infectious diseases [Internet]. 2019;19(1):139. Available from: https://www.ncbi.nlm.nih.gov/pmc/articles/PMC6371503/pdf/12879_2019_Article_3790.pdf

106. Zhao XJ, Li QX, Liu TJ, Wang DL, An YC, Zhang J, et al. Predictive values of CSS and NIHSS in the prognosis of patients with acute cerebral infarction: A comparative analysis. Medicine [Internet]. 2018;97(39):e12419-. Available from: https://www.ncbi.nlm.nih.gov/pmc/articles/PMC6181457/pdf/medi-97-e12419.pdf

107. Zhou J, Huang L, Chen J, Yuan X, Shen Q, Dong S, et al. Clinical features predicting mortality risk in older patients with COVID-19. Current medical research and opinion [Internet]. 2020;36(11):1753–9. Available from: https://www.tandfonline.com/doi/pdf/10.1080/03007995.2020.1825365?needAccess=true

108. Lima HMP, Carvalho FHC, Feitosa FEL, Nunes GC. Factors associated with maternal mortality among patients meeting criteria of severe maternal morbidity and near miss. International journal of gynaecology and obstetrics: the official organ of the International Federation of Gynaecology and Obstetrics. 2017;136(3):337–43.

109. Ueno F, Tamaki R, Saito M, Okamoto M, Saito-Obata M, Kamigaki T, et al. Age-specific incidence rates and risk factors for respiratory syncytial virus-associated lower respiratory tract illness in cohort children under 5 years old in the Philippines. Influenza Other Respir Viruses [Internet]. 2019;13(4):339–53. Available from: https://www.ncbi.nlm.nih.gov/pmc/articles/PMC6586181/pdf/IRV-13-339.pdf

110. Xie J, Covassin N, Fan Z, Singh P, Gao W, Li G, et al. Association Between Hypoxemia and Mortality in Patients With COVID-19. Mayo Clinic Proceedings [Internet]. 2020;95(6):1138–47. Available from: http://www.journals.elsevier.com/mayo-clinic-proceedings

111. Yang LC, Zhang RT, Guo LJ, Xiao H, Zu LY, Zhang YY, et al. [Hypoxia and inflammation are risk factors for acute myocardial injury in patients with coronavirus disease 2019]. Beijing Da Xue Xue Bao Yi Xue Ban. 2020;53(1):159–66.

112. Ghasemzadeh I, Mardani M, Mirtalaee N, Sanadgol G, Abolghasemi S. Risk Factors for In-Hospital Mortality among Cancer Patients with COVID-19: A Cross-Sectional Study. Current Respiratory Medicine Reviews [Internet]. 2022;18(1):57–64. Available from: https://www.eurekaselect.com/624/journal/current-respiratory-medicine-reviews

113. Carriel J, Munoz-Jaramillo R, Bolanos-Ladinez O, Heredia-Villacreses F, Menendez-Sanchon J, Martin-Delgado J. CURB-65 as a predictor of 30-day mortality in patients hospitalized with COVID-19 in Ecuador: COVID-EC study. Revista Clinica Espanola [Internet]. 2022;222(1):37–41. Available from: https://www.fesemi.org/

114. Liu Z, Bai X, Han X, Jiang W, Qiu L, Chen S, et al. The association of diabetes and the prognosis of COVID-19 patients: A retrospective study. Diabetes research and clinical practice. 2020;169(ebi, 8508335):108386.

115. Martin-del-Campo F, Ruvalcaba-Contreras N, Velazquez-Vidaurri AL, Cueto-Manzano AM, Rojas-Campos E, Cortes-Sanabria L, et al. Morbid obesity is associated with mortality and acute kidney injury in hospitalized patients with COVID-19. Clinical Nutrition ESPEN [Internet]. 2021;45((Martin-del-Campo, Ruvalcaba-Contreras, Velazquez-Vidaurri, Cueto-Manzano, Rojas-Campos, Espinel-Bermudez, Hernandez-Gonzalez, Nava-Zavala, Fuentes-Orozco, Balderas-Pena, Gonzalez-Ojeda) Unidad de Investigacion Biomedica 02, Hospital de Especialidades, CM):200–5. Available from: http://www.journals.elsevier.com/clinical-nutrition-espen

116. Hou CK, Qin YF, Wang G, Liu QL, Yang XY, Wang H. Impact of a long-term air pollution exposure on the case fatality rate of COVID-19 patients-A multicity study. Journal of medical virology. 2021;93(5):2938–46.

117. Majumder D, Manjunath M, Jayprakash S, Ishan R, Banerjee S, Kasana R. A Study of Cardiac Troponin T Levels in Acute Exacerbation of Copd and its Correlation with Severity. The Journal of the Association of Physicians of India. 2022;70(4):11–2.

118. Rajan M, Singh J, Singh Dalal J. Exchange blood transfusion in neonates with severe hyperbilirubinemia in a lower-middle-income country: can we minimise the incidence? Tropical Doctor [Internet]. 2021;51(2):146–50. Available from: https://search.ebscohost.com/login.aspx?direct=true&db=rzh&AN=150253650&site=ehost-live

119. Wasim T, Bushra N, Iqbal HI, Mumtaz A, Khan KS. Maternal condition as an underlying cause of perinatal mortality: Prospective cohort study. Journal of Obstetrics & Gynaecology Research [Internet]. 2021;47(2):544–50. Available from: https://search.ebscohost.com/login.aspx?direct=true&db=rzh&AN=148308776&site=ehost-live

120. Eleuterio T de A, Oliveira MC, Velasco MDS, Menezes R de A, Gomes RB, Martins MM, et al. SARS due to COVID-19: Predictors of death and profile of adult patients in the state of Rio de Janeiro, 2020. PloS one. 2022;17(11):e0277338-.

121. Andal VMD, Espiritu AI, Geronimo MAG, Salonga-Quimpo RAM, Gosiengfiao KTP, Fernandez MLL. Clinical features and outcomes of nonconvulsive status epilepticus in a developing country: A 5-year retrospective study. Epilepsy Behav. 2020;113:107547.

122. Tanboga IH, Canpolat U, Cetin EHO, Kundi H, Celik O, Caglayan M, et al. Development and validation of clinical prediction model to estimate the probability of death in hospitalized patients with COVID-19: Insights from a nationwide database. Journal of medical virology. 2021;93(5):3015–22.

123. Etherajan T, Zahir Hussain S, Rajendran K, Ramachandran A, Natrajan M, Venugopalan N, et al. Profile of COVID-19 Patients at Zero Delay COVID-19 Ward, Tertiary Care Hospital in South India. Journal of primary care & community health. 2021;12(101518419):21501327211035096-.

124. Oke OJ, Ajite A, Oluwayemi OI, Olatunya OS, Ogundare EO, Abdulraheem FO. Malaria Infection as a Leading Cause of Febrile Seizures among Children with Seizures in Ekiti State University Teaching Hospital, Ado Ekiti, Nigeria. Journal of Pediatric Neurology [Internet]. 2021;19(1):7–13. Available from: https://search.ebscohost.com/login.aspx?direct=true&db=rzh&AN=148424275&site=ehost-live

125. Thiruvengadam G, Lakshmi M, Ramanujam R. A Study of Factors Affecting the Length of Hospital Stay of COVID-19 Patients by Cox-Proportional Hazard Model in a South Indian Tertiary Care Hospital. Journal of primary care & community health. 2021;12(101518419):21501327211000230-.

126. Ozdemir S, Akca HS, Algin A, Altunok I, Eroglu SE. Effectiveness of the rapid emergency medicine score and the rapid acute physiology score in prognosticating mortality in patients presenting to the emergency department with COVID-19 symptoms. The American journal of emergency medicine. 2021;49(aa2, 8309942):259–64.

127. Rodriguez A, Mendoza D, Ascuntar J, Jaimes F. Supervised classification techniques for prediction of mortality in adult patients with sepsis. The American journal of emergency medicine. 2021;45(aa2, 8309942):392–7.

128. Liu Y, Chen H, Tan W, Kuang Y, Tang K, Luo Y, et al. Clinical characteristics and outcome of SARS-CoV-2 infection during pregnancy. Journal of Infection [Internet]. 2021;82(6):e9–10. Available from: http://www.elsevier.com/inca/publications/store/6/2/3/0/5/4/index.htt

129. Suttapanit K, Boriboon J, Sanguanwit P. Risk factors for non-invasive ventilation failure in influenza infection with acute respiratory failure in emergency department. Am J Emerg Med. 2021;45:368–73.

130. Bahloul M, Chelly H, ben Hmida M, ben Hamida C, Ksibi H, Kallel H, et al. Prognosis of traumatic head injury in South Tunisia: a multivariate analysis of 437 cases. The Journal of trauma. 2004;57(2):255–61.

131. Kirubakaran C, Gnananayagam JEJ, Sundaravalli EK. Comparison of blood gas values in arterial and venous blood. Indian J Pediatr [Internet]. 2003; Available from: http://imsear.searo.who.int/handle/123456789/83344

132. Russell GK, Merle CS, Cooke GS, Casas EC, Silveira da Fonseca M, du Cros P. Towards the WHO target of zero childhood tuberculosis deaths: an analysis of mortality in 13 locations in Africa and Asia. The international journal of tuberculosis and lung disease : the official journal of the International Union against Tuberculosis and Lung Disease [Internet]. 2013;17(12):1518–23. Available from: http://docserver.ingentaconnect.com/deliver/connect/iuatld/10273719/v17n12/s4.pdf?expires=1619064929&id=0000&titleid=3764&checksum=02FBC451CE8A7CDF120A76FFD97BFDE3

133. Samayam P, Ravi Chander B. Study of urinary tract infection and bacteriuria in neonatal sepsis. Indian journal of pediatrics [Internet]. 2012;79(8):1033–6. Available from: https://link.springer.com/content/pdf/10.1007/s12098-012-0727-7.pdf

134. Ogunbosi BO, Orimadegun AE, Carson S. Clinical features associated with oxygen use in children presenting to emergency care in Ibadan, Nigeria. American Journal of Respiratory and Critical Care Medicine [Internet]. 2011;183(1 MeetingAbstracts). Available from: http://ajrccm.atsjournals.org/cgi/reprint/183/1_MeetingAbstracts/A3384?sid=008c46ba-510f-4a68-8072-76be5ba70732

135. Gerardin P, Rogier C, Leteurtre S, Jouvencel P, Ka AS, Imbert P. Evaluation of Pediatric Risk of Mortality (PRISM) scoring in African children with falciparum malaria. Pediatric critical care medicine : a journal of the Society of Critical Care Medicine and the World Federation of Pediatric Intensive and Critical Care Societies. 2006;7(1):45–7.

136. Lupisan SP, Ruutu P, Erma Abucejo-Ladesma P, Quiambao BP, Gozum L, Sombrero LT, et al. Predictors of death from severe pneumonia among children 2-59 months old hospitalized in Bohol, Philippines: implications for referral criteria at a first-level health facility. Tropical medicine & international health : TM & IH. 2007;12(8):962–71.

137. Wang HC, Chang WN, Chang HW, Ho JT, Yang TM, Lin WC, et al. Factors predictive of outcome in posttraumatic seizures. The Journal of trauma. 2008;64(4):883–8.

138. Wang C. Early use of non-invasive positive pressure ventilation for acute exacerbations of chronic obstructive pulmonary disease: A multicentre randomized controlled trial. Chinese Medical Journal [Internet]. 2005;118(24):2034–40. Available from: http://www.cmj.org/Periodical/PaperList.asp?id=LW7641

139. Wang C-Y, Song C-M, Wang S-B, Liu G-H. Prealbumin may predict clinical outcomes in children with severe mycoplasma pneumoniae pneumonia. Iranian Journal of Pediatrics [Internet]. 2020;30(1):e97680-. Available from: http://ijp.tums.pub/

140. Shankar SK, Shankaranarayana Rao BS. Effect of myocardial ischaemia & reperfusion on brain. 2006; Available from: http://imsear.searo.who.int/handle/123456789/19768

141. Yun-Zhi MA, ZHAI HY, Chun-Ya SU. Therapeutic effect of early applying hydrotherapy with Chinese drugs on children hypoxic ischemic encephalopathy. Chinese Journal of Integrated Traditional and Western Medicine [Internet]. 2009;(12):130–2. Available from: http://dx.doi.org/

142. Gopinathan NR, Sen RK, Viswanathan VK, Aggarwal A, Mallikarjun HC, Rajaram Manoharan SR, et al. Early, reliable, utilitarian predictive factors for fat embolism syndrome in polytrauma patients. Indian J Crit Care Med. 2013;17(1):38–42.

143. Rudd KE, Seymour CW, Aluisio AR, Augustin ME, Bagenda DS, Beane A, et al. Association of the Quick Sequential (Sepsis-Related) Organ Failure Assessment (qSOFA) Score With Excess Hospital Mortality in Adults With Suspected Infection in Low- and Middle-Income Countries. JAMA [Internet]. 2018;319(21):2202–11. Available from: https://jamanetwork.com/journals/jama/articlepdf/2681801/jama_rudd_2018_oi_180052.pdf

144. Liu D, Wang Y, Wang J, Liu J, Yue Y, Liu W, et al. Characteristics and Outcomes of a Sample of Patients With COVID-19 Identified Through Social Media in Wuhan, China: Observational Study. Journal of medical Internet research. 2020;22(8):e20108-.

145. Shu L, Niu C, Li R, Huang T, Wang Y, Huang M, et al. Treatment of severe COVID-19 with human umbilical cord mesenchymal stem cells. Stem cell research & therapy [Internet]. 2020;11(1):361. Available from: https://stemcellres.biomedcentral.com/track/pdf/10.1186/s13287-020-01875-5.pdf

146. Leung C. Risk factors for predicting mortality in elderly patients with COVID-19: A review of clinical data in China. Mechanisms of ageing and development. 2020;188(lmj, 0347227):111255.

147. McCollum ED, Mvalo T, Smith AG, Eckerle M, Kondowe D, Lufesi N, et al. Bubble continuous positive airway pressure for children with severe pneumonia and severe malnutrition, human immunodeficiency virus infection or exposure, or severe hypoxemia in Malawi: An open label randomized controlled trial. American Journal of Tropical Medicine and Hygiene [Internet]. 2018;99(4 Supplement):648–9. Available from: http://www.ajtmh.org/docserver/fulltext/14761645/99/4_Suppl/tropmedabstract2018.pdf?expires=1555047060&id=id&accname=12015&checksum=81160026DD083BF102C66BCBC433A8AE

148. Zuberi FF, Khan JA. Prospective comparison of prediction rules of mortality risk for CAP in a developing country. The international journal of tuberculosis and lung disease : the official journal of the International Union against Tuberculosis and Lung Disease. 2008;12(4):447–52.

149. Juan MO, Jiyang LIU, Songbai WU, Ailian LÜ, XIAO L, CHEN D, et al. Predictive role of clinical features in patients with coronavirus disease 2019 for severe disease. Journal of Central South University(Medical Sciences) [Internet]. 2020;(12):536–41. Available from: http://dx.doi.org/10.11817/j.issn.1672-7347.2020.200384

150. Li LIU, ZHANG RF, Hong-Zhou LU, Shui-Hua LU, HUANG Q, XIONG YY, et al. Sixty-two severe and critical patients with 2009 influenza A (H1N1) in Shanghai, China. Chinese Medical Journal [Internet]. 2011;(24):1662–6. Available from: http://dx.doi.org/

151. Liu J, Wang D, Li J, Lin J, Xiong Y, Liu B, et al. Cerebral Microbleeds Do Not Predict Hemorrhagic Transformation in Acute Ischemic Stroke Patients with Atrial Fibrillation and/or Rheumatic Heart Disease. Current neurovascular research. 2017;14(2):104–9.

152. Zheng F, Sun Y, Zhong X, Wang Y, Wu R, Liu M, et al. A multicenter randomized, double-blind, placebo-controlled trial to evaluate the safety and efficacy of rhubarb in treating acute exacerbation of chronic obstructive pulmonary disease of the syndrome type phlegm-heat obstructing the lungs. Journal of Traditional Chinese Medical Sciences [Internet]. 2016;3(2):71–80. Available from: http://www.journals.elsevier.com/journal-of-traditional-chinese-medical-sciences/

153. Mahajan V, Tiwari M, Arya A, Tiwari A, Chawla D, Saini SS. Clinical predictors of hospital admission in acute lower respiratory tract infection in 2 months to 2-year-old children. Respirology [Internet]. 2016;21(2):350–6. Available from: http://www.blackwell-science.com/res

154. Saldias P F, Viviani G P, Pulgar B D, Valenzuela F F, Paredes E S, Diaz P O. [Prognostic factors and mortality in immunocompetent adult patients hospitalized with community-acquired pneumococcal pneumonia]. Revista medica de Chile [Internet]. 2009;137(12):1545–52. Available from: https://scielo.conicyt.cl/pdf/rmc/v137n12/art01.pdf

155. Zhou F, Wang Y, Liu Y, Liu X, Gu L, Zhang X, et al. Disease severity and clinical outcomes of community-acquired pneumonia caused by non-influenza respiratory viruses in adults: A multicentre prospective registry study from the CAP-China Network. European Respiratory Journal [Internet]. 2019;54(2):1802406. Available from: https://erj.ersjournals.com/content/erj/54/2/1802406.full.pdf

156. Teparrukkul P, Hantrakun V, Day NPJ, West TE, Limmathurotsakul D. Management and outcomes of severe dengue patients presenting with sepsis in a tropical country. PLoS ONE [Internet]. 2017;12(4):e0176233-. Available from: http://journals.plos.org/plosone/article/file?id=10.1371/journal.pone.0176233&type=printable

157. Subrahmanyam DKS, Mooney T, Raveendran R, Zachariah B. A clinical and laboratory profile of Cleistanthus collinus poisoning. 2003; Available from: http://imsear.searo.who.int/handle/123456789/88807

158. Deng Y, Liu W, Liu K, Fang YY, Shang J, Zhou L, et al. Clinical characteristics of fatal and recovered cases of coronavirus disease 2019 in Wuhan, China: a retrospective study. Chinese medical journal [Internet]. 2020;133(11):1261–7. Available from: https://www.ncbi.nlm.nih.gov/pmc/articles/PMC7289311/pdf/cm9-133-1261.pdf

159. Li S, Zhao D, Cui J, Wang L, Ma X, Li Y. Prevalence, potential risk factors and mortality rates of acute respiratory distress syndrome in Chinese patients with sepsis. The Journal of international medical research [Internet]. 2020;48(2):300060519895659-. Available from: https://journals.sagepub.com/doi/pdf/10.1177/0300060519895659

160. le Bourdelles G, Estagnasie P, Lenoir F, Brun P, Dreyfuss D. Use of a pulse oximeter in an adult emergency department: impact on the number of arterial blood gas analyses ordered. Chest [Internet]. 1998;113(4):1042–7. Available from: https://journal.chestnet.org/article/S0012-3692(15)47445-8/pdf

161. Pamaran RR, Kamigaki T, Hewe TT, Flores KMC, Mercado ES, Alday PP, et al. Epidemiological characterization of influenza A(H1N1)pdm09 cases from 2009 to 2010 in Baguio City, the Philippines. PLoS ONE [Internet]. 2013;8(11):e79916-. Available from: http://www.plosone.org/article/fetchObject.action?uri=info%3Adoi%2F10.1371%2Fjournal.pone.0079916&representation=PDF

162. El Sony AI, Chiang C-Y, Malik E, Hassanain SA, Hussien H, Khamis AH, et al. Standard case management of asthma in Sudan: A pilot project. Public Health Action [Internet]. 2013;3(3):247–52. Available from: http://docserver.ingentaconnect.com/deliver/connect/iuatld/22208372/v3n3/s14.pdf?

163. Zhou X, Li H, Ni X, Liu M, Hu M, Wu J, et al. Laboratory diagnosis and epidemiology of avian influenza A (H7N9) virus infection in humans in Nanchang City, China. Japanese Journal of Infectious Diseases [Internet]. 2013;66(6):558–60. Available from: https://www.jstage.jst.go.jp/article/yoken/66/6/66_558/_pdf

164. Conroy AL, Glover SJ, Hawkes M, Erdman LK, Seydel KB, Taylor TE, et al. Angiopoietin-2 levels are associated with retinopathy and predict mortality in Malawian children with cerebral malaria: a retrospective case-control study*. Critical care medicine [Internet]. 2012;40(3):952–9. Available from: https://www.ncbi.nlm.nih.gov/pmc/articles/PMC3284252/pdf/ukmss-36933.pdf

165. Lima EJ, Mello MJ, Albuquerque MF, Lopes MI, Serra GH, Abreu-Lima MA, et al. Clinical and epidemiological characteristics of severe community-acquired pneumonia in children after introduction of the 10-valent pneumococcal vaccine. Pediatric Health Med Ther [Internet]. 2015;6:131–8. Available from: https://www.dovepress.com/front_end/cr_data/cache/pdf/download_1618445703_6077858720573/PHMT-88132-clinical-and-epidemiological-characteristics-of-severe-commu_082415.pdf

166. Cao B, Gao H, Zhou B, Deng X, Hu C, Deng C, et al. Adjuvant corticosteroid treatment in adults with influenza a (H7N9) viral pneumonia. Critical Care Medicine [Internet]. 2016;44(6):e318–28. Available from: http://journals.lww.com/ccmjournal/pages/default.aspx

167. Huang C, Wang Y, Li X, Ren L, Zhao J, Hu Y, et al. Clinical features of patients infected with 2019 novel coronavirus in Wuhan, China. The Lancet [Internet]. 2020;395(10223):497–506. Available from: http://www.journals.elsevier.com/the-lancet/

168. Wu W, Wang J, Liu P, Chen W, Yin S, Hang S, et al. A hospital outbreak of severe acute respiratory syndrome in Guangzhou, China. Chinese Medical Journal [Internet]. 2003;116(6):811–8. Available from: https://journals.lww.com/cmj/pages/default.aspx

169. Ayyapan C, Rajeshwari PA, Edwin N. Clinical and computerised tomography evaluation of term neonates with perinatal asphyxia. Indian Pediatr [Internet]. 1999; Available from: http://imsear.searo.who.int/handle/123456789/11165

170. Moschovis PP, Banajeh S, MacLeod WB, Saha S, Hayden D, Christiani DC, et al. Childhood anemia at high altitude: risk factors for poor outcomes in severe pneumonia. Pediatrics [Internet]. 2013;132(5):e1156-62. Available from: https://www.ncbi.nlm.nih.gov/pmc/articles/PMC3812558/pdf/peds.2013-0761.pdf

171. Sittichanbuncha Y, Savatmongkorngul S, Jawroongrit P, Sawanyawisuth K. Low oxygen saturation is associated with pre-hospital mortality among non-traumatic patients using emergency medical services: A national database of Thailand. Turkiye Acil Tip Dergisi [Internet]. 2015;15(3):113–5. Available from: http://www.trjemergmed.com/full-text-pdf/8

172. Fan G, Tu C, Zhou F, Liu Z, Song B, Gu X, et al. Comparison of severity scores for COVID-19 patients with pneumonia: A retrospective study. European Respiratory Journal [Internet]. 2020;56(3):2002113. Available from: http://erj.ersjournals.com/lookup/doi/10.1183/13993003.02113-2020

173. Millman AL, Payne B, Qu Z, Douglas MJ, Hutcheon JA, Lee T, et al. Oxygen saturation as a predictor of adverse maternal outcomes in women with preeclampsia. Journal of Obstetrics and Gynaecology Canada [Internet]. 2011;33(7):705–14. Available from: https://core.ac.uk/download/46166127.pdf

174. Nantanda R, Ostergaard MS, Ndeezi G, Tumwine JK. Clinical outcomes of children with acute asthma and pneumonia in Mulago hospital, Uganda: A prospective study. BMC Pediatrics [Internet]. 2014;14(1):285. Available from: http://www.biomedcentral.com/bmcpediatr/

175. Yang K, Sheng Y, Huang C, Jin Y, Xiong N, Jiang K, et al. Clinical characteristics, outcomes, and risk factors for mortality in patients with cancer and COVID-19 in Hubei, China: a multicentre, retrospective, cohort study. The Lancet Oncology [Internet]. 2020;21(7):904–13. Available from: http://www.journals.elsevier.com/the-lancet-oncology/

176. Husada D, Chanthavanich P, Chotigeat U, Sunttarattiwong P, Sirivichayakul C, Pengsaa K, et al. Predictive model for bacterial late-onset neonatal sepsis in a tertiary care hospital in Thailand. BMC infectious diseases [Internet]. 2020;20(1):151. Available from: https://www.ncbi.nlm.nih.gov/pmc/articles/PMC7029566/pdf/12879_2020_Article_4875.pdf

177. Chisti MJ, Ahmed T, Ahmed AMS, Sarker SA, Faruque ASG, Islam MM, et al. Hypernatremia in Children With Diarrhea: Presenting Features, Management, Outcome, and Risk Factors for Death. Clinical pediatrics [Internet]. 2016;55(7):654–63. Available from: https://journals.sagepub.com/doi/10.1177/0009922815627346?url_ver=Z39.88-2003&rfr_id=ori:rid:crossref.org&rfr_dat=cr_pub%3dpubmed

178. Kress JP, Pohlman AS, Hall JB. Determination of hemoglobin saturation in patients with acute sickle chest syndrome: a comparison of arterial blood gases and pulse oximetry. Chest [Internet]. 1999;115(5):1316–20. Available from: https://journal.chestnet.org/article/S0012-3692(15)35286-7/pdf

179. Huq S, Hossain MI, Malek MA, Faruque ASG, Salam MA. Hypoglycaemia in under-five children with diarrhoea. Journal of tropical pediatrics [Internet]. 2007;53(3):197–201. Available from: https://watermark.silverchair.com/fmm006.pdf?token=AQECAHi208BE49Ooan9kkhW_Ercy7Dm3ZL_9Cf3qfKAc485ysgAAAqIwggKeBgkqhkiG9w0BBwagggKPMIICiwIBADCCAoQGCSqGSIb3DQEHATAeBglghkgBZQMEAS4wEQQMUEvpbf6YZ4CAUH1KAgEQgIICVXVRPAUqox0qipyzuJnuc5JLDfYb08ZWw_U8vOABY-DI20zL

180. Emami A, Fadakar N, Akbari A, Lotfi M, Farazdaghi M, Javanmardi F, et al. Seizure in patients with COVID-19. Neurological sciences : official journal of the Italian Neurological Society and of the Italian Society of Clinical Neurophysiology [Internet]. 2020;41(11):3057–61. Available from: https://link.springer.com/content/pdf/10.1007/s10072-020-04731-9.pdf

181. Satici C, Demirkol MA, Sargin Altunok E, Gursoy B, Alkan M, Kamat S, et al. Performance of pneumonia severity index and CURB-65 in predicting 30-day mortality in patients with COVID-19. International journal of infectious diseases : IJID : official publication of the International Society for Infectious Diseases. 2020;98(c3r, 9610933):84–9.

182. Amalakanti S, Pentakota MR. Pulse oximetry overestimates oxygen saturation in COPD. Respiratory Care [Internet]. 2016;61(4):423–7. Available from: http://rc.rcjournal.com/content/61/4/423.full.pdf

183. King C, Boyd N, Walker I, Zadutsa B, Baqui AH, Ahmed S, et al. Opportunities and barriers in paediatric pulse oximetry for pneumonia in low-resource clinical settings: a qualitative evaluation from Malawi and Bangladesh. BMJ open [Internet]. 2018;8(1):e019177-. Available from: https://www.ncbi.nlm.nih.gov/pmc/articles/PMC5829842/pdf/bmjopen-2017-019177.pdf

184. Yu C, Lei Q, Li W, Wang X, Liu W, Fan X, et al. Clinical Characteristics, Associated Factors, and Predicting COVID-19 Mortality Risk: A Retrospective Study in Wuhan, China. American journal of preventive medicine [Internet]. 2020;59(2):168–75. Available from: https://www.ncbi.nlm.nih.gov/pmc/articles/PMC7250782/pdf/main.pdf

185. Saldias F, Mardonez JM, Marchesse M, Viviani P, Farias G, Diaz A. Community-acquired pneumonia in hospitalized adult patients. Clinical presentation and prognostic factors. Revista medica de Chile. 2002;130(12):1373–82.

186. Ajzenberg D, Lamaury I, Demar M, Vautrin C, Cabie A, Simon S, et al. Performance Testing of PCR Assay in Blood Samples for the Diagnosis of Toxoplasmic Encephalitis in AIDS Patients from the French Departments of America and Genetic Diversity of Toxoplasma gondii: A Prospective and Multicentric Study. PLoS neglected tropical diseases [Internet]. 2016;10(6):e0004790-. Available from: https://www.ncbi.nlm.nih.gov/pmc/articles/PMC4927177/pdf/pntd.0004790.pdf

187. Conroy AL, Hawkes M, Hayford K, Namasopo S, Opoka RO, John CC, et al. Prospective validation of pediatric disease severity scores to predict mortality in Ugandan children presenting with malaria and non-malaria febrile illness. Critical care (London, England) [Internet]. 2015;19(9801902):47. Available from: https://ccforum.biomedcentral.com/track/pdf/10.1186/s13054-015-0773-4.pdf

188. Quiambao BP, Ruutu PJ, Ladesma EA, Gozum LS, Inobaya MT, Lupisan SP, et al. Pneumonia among young infants in rural Southeast Asia (Bohol Island, Philippines). Tropical medicine & international health : TM & IH [Internet]. 2009;14(12):1457–66. Available from: https://onlinelibrary.wiley.com/doi/pdfdirect/10.1111/j.1365-3156.2009.02398.x?download=true

189. Karbalai Saleh S, Oraii A, Soleimani A, Hadadi A, Shajari Z, Montazeri M, et al. The association between cardiac injury and outcomes in hospitalized patients with COVID-19. Internal and emergency medicine [Internet]. 2020;15(8):1415–24. Available from: https://www.ncbi.nlm.nih.gov/pmc/articles/PMC7415198/pdf/11739_2020_Article_2466.pdf

190. Dai RX, Kong QH, Mao B, Xu W, Tao RJ, Wang XR, et al. The mortality risk factor of community acquired pneumonia patients with chronic obstructive pulmonary disease: a retrospective cohort study. BMC pulmonary medicine [Internet]. 2018;18(1):12. Available from: https://www.ncbi.nlm.nih.gov/pmc/articles/PMC5778745/pdf/12890_2018_Article_587.pdf

191. Chhibber AV, Hill PC, Jafali J, Jasseh M, Hossain MI, Ndiaye M, et al. Child Mortality after Discharge from a Health Facility following Suspected Pneumonia, Meningitis or Septicaemia in Rural Gambia: A Cohort Study. PloS one [Internet]. 2015;10(9):e0137095-. Available from: https://www.ncbi.nlm.nih.gov/pmc/articles/PMC4564213/pdf/pone.0137095.pdf

192. Moyes J, Walaza S, Pretorius M, Groome M, A von G, Wolter N, et al. Respiratory syncytial virus in adults with severe acute respiratory illness in a high HIV prevalence setting. Journal of Infection [Internet]. 2017;75(4):346–55. Available from: http://www.elsevier.com/inca/publications/store/6/2/3/0/5/4/index.htt

193. Cerro L, Valencia J, Calle P, Leon A, Jaimes F. [Validation of APACHE II and SOFA scores in 2 cohorts of patients with suspected infection and sepsis, not admitted to critical care units]. Revista espanola de anestesiologia y reanimacion. 2014;61(3):125–32.

194. Han X, Zhou F, Li H, Xing X, Chen L, Wang Y, et al. Effects of age, comorbidity and adherence to current antimicrobial guidelines on mortality in hospitalized elderly patients with community-acquired pneumonia. BMC infectious diseases [Internet]. 2018;18(1):192. Available from: https://www.ncbi.nlm.nih.gov/pmc/articles/PMC5922029/pdf/12879_2018_Article_3098.pdf

195. Hu H, Yao N, Qiu Y. Comparing Rapid Scoring Systems in Mortality Prediction of Critically Ill Patients With Novel Coronavirus Disease. Academic emergency medicine : official journal of the Society for Academic Emergency Medicine [Internet]. 2020;27(6):461–8. Available from: https://onlinelibrary.wiley.com/doi/pdfdirect/10.1111/acem.13992?download=true

196. Vinekar A, Jayadev C, Kumar S, Mangalesh S, Dogra MR, Bauer NJ, et al. Impact of improved neonatal care on the profile of retinopathy of prematurity in rural neonatal centers in India over a 4-year period. Eye and Brain [Internet]. 2016;8((Vinekar, Jayadev, Mangalesh) Department of Pediatric Retina, Narayana Nethralaya Eye Hospital, 121/C, Chord Road, 1st "R" Block, Rajajinagar, Bangalore, Karnataka 560 010, India(Kumar) Raichur Institute of Medical Sciences, Raichur, Karnataka, India(Mang):45–53. Available from: http://www.dovepress.com/eye-and-brain-journal

197. Awasthi S. Three day versus five day treatment with amoxicillin for non-severe pneumonia in young children: A multicentre randomised controlled trial. British Medical Journal. 2004;328(7443):791–4.

198. Rerksuppaphol L, Rerksuppaphol S. Efficacy of Adjunctive Zinc in Improving the Treatment Outcomes in Hospitalized Children with Pneumonia: A Randomized Controlled Trial. Journal of tropical pediatrics [Internet]. 2020;66(4):419–27. Available from: https://watermark.silverchair.com/fmz082.pdf?token=AQECAHi208BE49Ooan9kkhW_Ercy7Dm3ZL_9Cf3qfKAc485ysgAAArowggK2BgkqhkiG9w0BBwagggKnMIICowIBADCCApwGCSqGSIb3DQEHATAeBglghkgBZQMEAS4wEQQM1l2Ke3HJqcfTWCW7AgEQgIICbR137itlk2np6e9pV6geguG5inp5E243Rkwz5pLGnI07KFmU

199. Agarwal G, Awasthi S, Kabra SK, Kaul A, Singhi S, Walter SD, et al. Three day versus five day treatment with amoxicillin for non-severe pneumonia in young children: a multicentre randomised controlled trial. BMJ (Clinical research ed). 2004;328(7443):791.

200. Wang JB, Wang HT, Wang LS, Li LP, Xv J, Xv C, et al. Epidemiological and clinical characteristics of fifty-six cases of COVID-19 in Liaoning Province, China. World journal of clinical cases [Internet]. 2020;8(21):5188–202. Available from: https://www.ncbi.nlm.nih.gov/pmc/articles/PMC7674744/pdf/WJCC-8-5188.pdf

201. Banajeh SM. Nutritional rickets and vitamin D deficiency--association with the outcomes of childhood very severe pneumonia: a prospective cohort study. Pediatric pulmonology [Internet]. 2009;44(12):1207–15. Available from: https://onlinelibrary.wiley.com/doi/abs/10.1002/ppul.21121

202. Yeo TW, Lampah DA, Kenangalem E, Tjitra E, Price RN, Anstey NM. Impaired skeletal muscle microvascular function and increased skeletal muscle oxygen consumption in severe falciparum malaria. Journal of Infectious Diseases [Internet]. 2013;207(3):528–36. Available from: http://jid.oxfordjournals.org/content/current

203. Conroy AL, Hawkes M, Hayford K, Hermann L, McDonald CR, Sharma S, et al. Methemoglobin and nitric oxide therapy in Ugandan children hospitalized for febrile illness: Results from a prospective cohort study and randomized double-blind placebo-controlled trial. BMC Pediatrics [Internet]. 2016;16(1):177. Available from: http://www.biomedcentral.com/bmcpediatr/

204. Wiens MO, Kumbakumba E, Larson CP, Ansermino JM, Singer J, Kissoon N, et al. Postdischarge mortality in children with acute infectious diseases: derivation of postdischarge mortality prediction models. BMJ open [Internet]. 2015;5(11):e009449-. Available from: https://www.ncbi.nlm.nih.gov/pmc/articles/PMC4663423/pdf/bmjopen-2015-009449.pdf

205. Bijani B, Pahlevan AA, Qasemi-Barqi R, Jahanihashemi H. Metabolic syndrome as an independent risk factor of hypoxaemia in influenza a (H1N1) 2009 pandemic. Infezioni in Medicina [Internet]. 2016;24(2):123–30. Available from: http://www.infezmed.it/VisualizzaArticolo.aspx

206. Cao Z, Li T, Liang L, Wang H, Wei F, Meng S, et al. Clinical characteristics of Coronavirus Disease 2019 patients in Beijing, China. PloS one [Internet]. 2020;15(6):e0234764-. Available from: https://www.ncbi.nlm.nih.gov/pmc/articles/PMC7299347/pdf/pone.0234764.pdf

207. Elamari S, Motaib I, Zbiri S, Elaidaoui K, Chadli A, Elkettani C. Characteristics and outcomes of diabetic patients infected by the SARS-CoV-2. The Pan African medical journal. 2020;37(101517926):32.

208. Dhabangi A, Ainomugisha B, Cserti-Gazdewich C, Ddungu H, Kyeyune D, Musisi E, et al. Cerebral oximetry in ugandan children with severe anemia clinical categories and response to transfusion. JAMA Pediatrics [Internet]. 2016;170(10):995–1002. Available from: http://jamanetwork.com/journals/jamapediatrics/data/Journals/PEDS/935728/poi160042.pdf

209. Feikin DR, Njenga MK, Bigogo G, Aura B, Aol G, Audi A, et al. Viral and bacterial causes of severe acute respiratory illness among children aged less than 5 years in a high malaria prevalence area of western Kenya, 2007-2010. The Pediatric infectious disease journal. 2013;32(1):e14-9.

210. Gray AZ, Morpeth M, Duke T, Peel D, Winter C, Satvady M, et al. Improved oxygen systems in district hospitals in Lao PDR: a prospective field trial of the impact on outcomes for childhood pneumonia and equipment sustainability. BMJ Paediatr Open [Internet]. 2017;1(1):e000083-. Available from: https://www.ncbi.nlm.nih.gov/pmc/articles/PMC5862216/pdf/bmjpo-2017-000083.pdf

211. Gupta N, Ish P, Kumar R, Dev N, Yadav SR, Malhotra N, et al. Evaluation of the clinical profile, laboratory parameters and outcome of two hundred COVID-19 patients from a tertiary centre in India. Monaldi archives for chest disease = Archivio Monaldi per le malattie del torace [Internet]. 2020;90(4). Available from: https://www.monaldi-archives.org/index.php/macd/article/download/1507/1123

212. He X, Wang L, Wang H, Xie Y, Yu Y, Sun J, et al. Factors associated with acute cardiac injury and their effects on mortality in patients with COVID-19. Scientific reports [Internet]. 2020;10(1):20452. Available from: https://www.ncbi.nlm.nih.gov/pmc/articles/PMC7686361/pdf/41598_2020_Article_77172.pdf

213. Liu S, Luo H, Wang Y, Cuevas LE, Wang D, Ju S, et al. Clinical characteristics and risk factors of patients with severe COVID-19 in Jiangsu province, China: a retrospective multicentre cohort study. BMC infectious diseases [Internet]. 2020;20(1):584. Available from: https://www.ncbi.nlm.nih.gov/pmc/articles/PMC7407434/pdf/12879_2020_Article_5314.pdf

214. Madhi SA, Ramasamy N, Bessellar TG, Saloojee H, Klugman KP. Lower respiratory tract infections associated with influenza A and B viruses in an area with a high prevalence of pediatric human immunodeficiency type 1 infection. Pediatric Infectious Disease Journal. 2002;21(4):291–7.

215. Assies R, Snik I, Kumwenda M, Chimalizeni Y, Langton J, Woensel JBM van, et al. Etiology, Pathophysiology and Mortality of Shock in Children in Low (Middle) Income Countries: A Systematic Review. Journal of Tropical Pediatrics [Internet]. 2022;68(4):1–17. Available from: https://search.ebscohost.com/login.aspx?direct=true&db=rzh&AN=158394294&site=ehost-live

216. Azarkar G, Osmani F. Clinical characteristics and risk factors for mortality in COVID-19 inpatients in Birjand, Iran: a single-center retrospective study. European journal of medical research. 2021;26(1):79.

217. Buendia JA, Polack FP, Patino DG. Clinical Manifestations and Outcomes of Respiratory Syncytial Virus Infection in Children Less Than Two Years in Colombia. Indian Pediatrics [Internet]. 2021;58(11):1091–2. Available from: https://www.springer.com/journal/13312

218. Olivas-Martinez A, Cardenas-Fragoso JL, Jimenez JV, Lozano-Cruz OA, Ortiz-Brizuela E, Tovar-Mendez VH, et al. In-hospital mortality from severe COVID-19 in a tertiary care center in Mexico City; causes of death, risk factors and the impact of hospital saturation. PloS one. 2021;16(2):e0245772-.

219. Garcia Hernandez D, Calls Samora M, Llimona Gonzalez A, Dinamarca Caceres F, Casanovas Martinez F, Perez Oms A, et al. Acute confusional syndrome: Differences between COVID-19 and others comorbid diseases: A Liason psychiatry service research. Journal of the Neurological Sciences. 2021;429(Supplement):119904.

220. Pya Y, Bekbossynova M, Gaipov A, Lesbekov T, Kapyshev T, Kuanyshbek A, et al. Mortality predictors of hospitalized patients with COVID-19: Retrospective cohort study from Nur-Sultan, Kazakhstan. PloS one. 2021;16(12):e0261272-.

221. Urrets-Zavalia JA, Crim N, Knoll EG, Esposito FA, Collino E, Urrets-Zavalia ME, et al. Impact of changing oxygenation policies on retinopathy of prematurity in a neonatal unit in Argentina. The British journal of ophthalmology [Internet]. 2012;96(12):1456–61. Available from: https://bjo.bmj.com/content/bjophthalmol/96/12/1456.full.pdf

222. Alavi-Moghaddam M, Bakhshi H, Rezaei B, Khashayar P. Pneumonia severity index compared to CURB-65 in predicting the outcome of community acquired pneumonia among patients referred to an Iranian emergency department: a prospective survey. The Brazilian journal of infectious diseases : an official publication of the Brazilian Society of Infectious Diseases. 2013;17(2):179–83.

223. Sessions KL, Mvalo T, Kondowe D, Makonokaya D, Hosseinipour MC, Chalira A, et al. Bubble CPAP and oxygen for child pneumonia care in Malawi: a CPAP IMPACT time motion study. BMC health services research [Internet]. 2019;19(1):533. Available from: https://www.ncbi.nlm.nih.gov/pmc/articles/PMC6668155/pdf/12913_2019_Article_4364.pdf

224. Alan S, Erdeve O, Cakir U, Akduman H, Zenciroglu A, Akcakus M, et al. Outcome of the Respiratory Syncytial Virus related acute lower respiratory tract infection among hospitalized newborns: A prospective multicenter study. Journal of Maternal-Fetal and Neonatal Medicine [Internet]. 2016;29(13):2186–93. Available from: https://www.tandfonline.com/doi/full/10.3109/14767058.2015.1079614

225. Li K, Chen D, Chen S, Feng Y, Chang C, Wang Z, et al. Predictors of fatality including radiographic findings in adults with COVID-19. Respiratory research [Internet]. 2020;21(1):146. Available from: https://www.ncbi.nlm.nih.gov/pmc/articles/PMC7289230/pdf/12931_2020_Article_1411.pdf

226. Fe MMM, Monteiro AJ, Moura FEA. Parainfluenza virus infections in a tropical city: Clinical and epidemiology aspects. Brazilian Journal of Infectious Diseases [Internet]. 2008;12(3):192–7. Available from: http://www.scielo.br/pdf/bjid/v12n3/a06v12n3.pdf

227. Bangirana P, Seggane-Musisi, Allebeck P, Giordani B, John CC, Opoka OR, et al. A preliminary examination of the construct validity of the KABC-II in Ugandan children with a history of cerebral malaria. African health sciences [Internet]. 2009;9(3):186–92. Available from: https://www.ncbi.nlm.nih.gov/pmc/articles/PMC2887024/pdf/AFHS0903-0186.pdf

228. Ji R, Shen H, Pan Y, Wang P, Liu G, Wang Y, et al. Novel risk score to predict pneumonia after acute ischemic stroke. Stroke [Internet]. 2013;44(5):1303–9. Available from: https://www.ahajournals.org/doi/pdf/10.1161/STROKEAHA.111.000598?download=true

229. Adzic-Vukicevic T, Stosic M, Antonijevic G, Jevtic M, Radovanovic-Spurnic A, Velickovic J. Tuberculosis and COVID-19 co-infection in Serbia: Pandemic challenge in a low-burden country. Frontiers in Medicine [Internet]. 2022;9((Adzic-Vukicevic, Radovanovic-Spurnic, Velickovic) Faculty of Medicine, University of Belgrade, Belgrade, Serbia(Adzic-Vukicevic) Clinic for Pulmonology, University Clinical Center of Serbia, Belgrade, Serbia(Adzic-Vukicevic, Radovanovic-Spurnic, Velickov):971008. Available from: https://journal.frontiersin.org/journal/medicine

230. Akdogan D, Guzel M, Tosun D, Akpinar O. Diagnostic and early prognostic value of serum CRP and LDH levels in patients with possible COVID-19 at the first admission. Journal of infection in developing countries. 2021;15(6):766–72.

231. Arias Ramos D, Restrepo Rueda DL, Rios Quintero EV, Olaya Gomez JC, Cortes Bonilla I. Severe and critical COVID-19 in a tertiary center in Colombia, a retrospective cross-sectional study. BMC Infectious Diseases [Internet]. 2022;22(1):247. Available from: http://www.biomedcentral.com/bmcinfectdis/

232. Kilercik M, Demirelce O, Serdar MA, Mikailova P, Serteser M. A new haematocytometric index: Predicting severity and mortality risk value in COVID-19 patients. PloS one. 2021;16(8):e0254073-.

233. Ma X, Wang H, Huang J, Geng Y, Jiang S, Zhou Q, et al. A nomogramic model based on clinical and laboratory parameters at admission for predicting the survival of COVID-19 patients. BMC infectious diseases. 2020;20(1):899.

234. Chen Z, Chen J, Zhou J, Lei F, Zhou F, Qin JJ, et al. A risk score based on baseline risk factors for predicting mortality in COVID-19 patients. Current medical research and opinion. 2021;37(6):917–27.

235. Chen L, Han X, Li YL, Zhang C, Xing X. FluA-p score: a novel prediction rule for mortality in influenza A-related pneumonia patients. Respiratory research. 2020;21(1):109.

236. Shetty A, Aggarwal S, Patel S, Sharma S, Jindal K, Prakash M. Early versus delayed thromboprophylaxis with LMWH in pelvic-acetabular trauma- a prospective study. Injury [Internet]. 2022;53(2):529–33. Available from: https://search.ebscohost.com/login.aspx?direct=true&db=rzh&AN=154972652&site=ehost-live

237. Hailu S, Gebre H, Alemayehu G. Orthopaedic injury patterns at a tertiary referral hospital in Ethiopia: a prospective observational study. Injury [Internet]. 2022;53(10):3195–200. Available from: https://search.ebscohost.com/login.aspx?direct=true&db=rzh&AN=159189055&site=ehost-live

238. Perez-Padilla R, Garcia-Sancho C, Fernandez R, Franco-Marina F, Lopez-Gatell H, Bojorquez I. The impact of altitude on hospitalization and hospital mortality from pandemic 2009 influenza A (H1N1) virus pneumonia in Mexico. Salud Publica de Mexico [Internet]. 2013;55(1):92–5. Available from: http://bvs.insp.mx/rsp/_files/File/2013/vol%2055%20no.1%20Enero%20Febrero/10impact.pdf

239. Ma X, Ng M, Xu S, Xu Z, Qiu H, Liu Y, et al. Development and validation of prognosis model of mortality risk in patients with COVID-19. Epidemiology and infection [Internet]. 2020;148(epi, 8703737):e168-. Available from: https://www.cambridge.org/core/services/aop-cambridge-core/content/view/9A3B09B53AF543DDF0BC8F1348EFF07C/S0950268820001727a.pdf/div-class-title-development-and-validation-of-prognosis-model-of-mortality-risk-in-patients-with-covid-19-div.pdf

240. Zepeda-Romero LC, Lundgren P, Gutierrez-Padilla JA, Gomez-Ruiz LM, Quiles Corona M, Orozco-Monroy JV, et al. Oxygen Monitoring Reduces the Risk for Retinopathy of Prematurity in a Mexican Population. Neonatology. 2016;110(2):135–40.

241. Khwannimit B. Pulse oximetry in adults. Songklanagarind Medical Journal. 2020;24(3):245–52.

242. Song H, Xi J, Li GG, Xu S, Wang C, Cheng T, et al. Upregulation of CD19+CD24(hi)CD38(hi) regulatory B cells is associated with a reduced risk of acute lung injury in elderly pneumonia patients. Internal and emergency medicine [Internet]. 2016;11(3):415–23. Available from: https://link.springer.com/content/pdf/10.1007/s11739-015-1377-3.pdf

243. Tanimowo MO. Mortality predictors in community-acquired pneumonia. Nigerian journal of clinical practice. 2009;12(3):298–301.

244. Wang S, Chen Z, Lin Y, Lin L, Lin Q, Fang S, et al. Clinical characteristics of 199 discharged patients with COVID-19 in Fujian Province: A multicenter retrospective study between January 22nd and February 27th, 2020. PLoS ONE [Internet]. 2020;15(11 November):e0242307-. Available from: https://journals.plos.org/plosone/article/file?id=10.1371/journal.pone.0242307&type=printable

245. Alay I, Yildiz S, Kaya C, Yasar KK, Aydin OA, Karaosmanoglu HK, et al. The clinical findings and outcomes of symptomatic pregnant women diagnosed with or suspected of having coronavirus disease 2019 in a tertiary pandemic hospital in Istanbul, Turkey. Journal of Obstetrics and Gynaecology Research [Internet]. 2020;46(12):2552–60. Available from: http://obgyn.onlinelibrary.wiley.com/hub/journal/10.1111/(ISSN)1447-0756/

246. Pelkonen T, Roine I, Monteiro L, Joao Simoes M, Anjos E, Pelerito A, et al. Acute childhood bacterial meningitis in Luanda, Angola. Scandinavian journal of infectious diseases. 2008;40(11–12):859–66.

247. Cetinkal G, Kocas BB, Ser OS, Kilci H, Keskin K, Ozcan SN, et al. Assessment of the Modified CHA2DS2VASc Risk Score in Predicting Mortality in Patients Hospitalized With COVID-19. The American journal of cardiology [Internet]. 2020;135(3dq, 0207277):143–9. Available from: https://www.ajconline.org/article/S0002-9149(20)30897-3/pdf

248. Avci S, Perincek G. The alveolar-arterial gradient, pneumonia severity scores and inflammatory markers to predict 30-day mortality in pneumonia. The American journal of emergency medicine. 2020;38(9):1796–801.

249. Maitland K, Kiguli S, Opoka RO, Olupot-Olupot P, Engoru C, Njuguna P, et al. Children's Oxygen Administration Strategies Trial (COAST): A randomised controlled trial of high flow versus oxygen versus control in African children with severe pneumonia. Wellcome Open Res [Internet]. 2017;2:100. Available from: https://spiral.imperial.ac.uk:8443/bitstream/10044/1/54643/8/COAST_published_protocol.pdf

250. Iroezindu MO, Isiguzo GC, Chima EI, Mbata GC, Onyedibe KI, Onyedum CC, et al. Predictors of in-hospital mortality and length of stay in community-acquired pneumonia: a 5-year multi-centre case control study of adults in a developing country. Transactions of the Royal Society of Tropical Medicine and Hygiene [Internet]. 2016;110(8):445–55. Available from: https://academic.oup.com/trstmh/article-abstract/110/8/445/2427546?redirectedFrom=fulltext

251. Duke T, Oa O, Mokela D, Oswyn G, Hwaihwanje I, Hawap J. The management of sick young infants at primary health centres in a rural developing country. Archives of disease in childhood [Internet]. 2005;90(2):200–5. Available from: https://www.ncbi.nlm.nih.gov/pmc/articles/PMC1720244/pdf/v090p00200.pdf

252. Subramanian M, Ramadurai S, Arthur P, Gopalan S. Hypoxia as an independent predictor of adverse outcomes in pulmonary embolism. Asian cardiovascular & thoracic annals. 2018;26(1):38–43.

253. Shane AI, Robert W, Arthur K, Patson M, Moses G. Acid-base disorders as predictors of early outcomes in major trauma in a resource limited setting: An observational prospective study. The Pan African medical journal [Internet]. 2014;17:2. Available from: https://www.ncbi.nlm.nih.gov/pmc/articles/PMC4149796/pdf/PAMJ-17-02.pdf

254. Sen HS, Abakay O, Tanrikulu AC, Sezgi C, Taylan M, Abakay A, et al. Is a complete blood cell count useful in determining the prognosis of pulmonary embolism?. Wiener klinische Wochenschrift [Internet]. 2014;126(11–12):347–54. Available from: https://link.springer.com/content/pdf/10.1007/s00508-014-0537-1.pdf

255. Akhter S, Warraich UA, Ghazal S, Rizvi N. Assessment and comparison of APACHE II (Acute Physiology and Chronic Health Evaluation), SOFA (Sequential Organ Failure Assessment) score and CURB 65 (Confusion; Urea; Respiratory Rate; Blood Pressure), for prediction of inpatient mortality in Acute Exace. JPMA The Journal of the Pakistan Medical Association. 2019;69(2):211–5.

256. Barroso-Sousa R, Lobo RR, Mendonca PR, Memoria RR, Spiller F, Cunha FQ, et al. Decreased levels of alpha-1-acid glycoprotein are related to the mortality of septic patients in the emergency department. Clinics (Sao Paulo, Brazil). 2013;68(8):1134–9.

257. Zhang Z, Xu L, Pang X, Zeng Y, Hao Y, Wang Y, et al. A Clinical scoring model to predict mortality in HIV/TB co-infected patients at end stage of AIDS in China: An observational cohort study. Bioscience trends [Internet]. 2019;13(2):136–44. Available from: https://www.jstage.jst.go.jp/article/bst/13/2/13_2018.01309/_pdf

258. Yan Y, Yang Y, Wang F, Ren H, Zhang S, Shi X, et al. Clinical characteristics and outcomes of patients with severe covid-19 with diabetes. BMJ open diabetes research & care [Internet]. 2020;8(1). Available from: https://drc.bmj.com/content/bmjdrc/8/1/e001343.full.pdf

259. Hu GP, Zhou YM, Wu ZL, Li YQ, Liang WQ, Wei LP, et al. Red blood cell distribution width is an independent predictor of mortality for an acute exacerbation of COPD. The international journal of tuberculosis and lung disease : the official journal of the International Union against Tuberculosis and Lung Disease [Internet]. 2019;23(7):817–23. Available from: http://docserver.ingentaconnect.com/deliver/connect/iuatld/10273719/v23n7/s8.pdf?expires=1618445699&id=0000&titleid=3764&checksum=34D043CFC7AEC81C0886BB4EF452E850

260. Godoy DA, Pinero G, di Napoli M. Predicting mortality in spontaneous intracerebral hemorrhage: can modification to original score improve the prediction?. Stroke [Internet]. 2006;37(4):1038–44. Available from: https://www.ahajournals.org/doi/pdf/10.1161/01.STR.0000206441.79646.49?download=true

261. Laman M, Aipit S, Bona C, Aipit J, Davis TME, Manning L. Contribution of Malaria to Inhospital Mortality in Papua New Guinean Children from a Malaria-Endemic Area: A Prospective Observational Study. The American journal of tropical medicine and hygiene [Internet]. 2019;100(4):835–41. Available from: https://www.ajtmh.org/downloadpdf/journals/tpmd/100/4/article-p835.pdf

262. Futrakul S, Praisuwanna P, Thaitumyanon P. Risk factors for hypoxic-ischemic encephalopathy in asphyxiated newborn infants. 2006; Available from: http://imsear.searo.who.int/handle/123456789/41760

263. Shah GS, Singh R, Das BK. Outcome of newborns with birth asphyxia. 2006; Available from: http://imsear.searo.who.int/handle/123456789/45948

264. Jie GAO, Qin-Li SUN, ZHANG YM, Yan-Yan LI, Huan LI, Xin HOU, et al. Semi-quantitative assessment of brain maturation by conventional magnetic resonance imaging in neonates with clinically mild hypoxic-ischemic encephalopathy. Chinese Medical Journal [Internet]. 2015;(24):574–80. Available from: http://dx.doi.org/10.4103/0366-6999.151646

265. Clifton DC, Ramadhani HO, Msuya LJ, Njau BN, Kinabo GD, Buchanan AM, et al. Predicting mortality for paediatric inpatients where malaria is uncommon. Archives of disease in childhood [Internet]. 2012;97(10):889–94. Available from: https://www.ncbi.nlm.nih.gov/pmc/articles/PMC3508729/pdf/nihms422225.pdf

266. Hensleigh PA. Anti-shock garment provides resuscitation and haemostasis for obstetric haemorrhage. BJOG : an international journal of obstetrics and gynaecology. 2002;109(12):1377–84.

267. Green SL, Smith MTD, Cairns C, Clarke DL, Bruce J, Bekker W, et al. The Combined SIRS + qSOFA (qSIRS) Score is More Accurate Than qSOFA Alone in Predicting Mortality in Patients with Surgical Sepsis in an LMIC Emergency Department. World journal of surgery [Internet]. 2020;44(1):21–9. Available from: https://link.springer.com/article/10.1007%2Fs00268-019-05181-x

268. Espino-Núñez JS, Quinto-Sánchez M, Carrada-Varela AC, Román-Morales F. Physician Prehospital Care in Mexico City: Retrospective Analysis of Endotracheal Intubation in Patients with Severe Head Trauma. Prehosp Disaster Med. 2020;35(2):128–32.

269. Woldeamanuel YW, Andemeskel AT, Kyei K, Woldeamanuel MW, Woldeamanuel W. Case fatality of adult tetanus in Africa: Systematic review and meta-analysis. Journal of the Neurological Sciences [Internet]. 2016;368((Woldeamanuel, Woldeamanuel, Woldeamanuel) Advanced Clinical Consultation&Research Center, Addis Ababa, Ethiopia(Woldeamanuel) Department of Neurology and Neurological Sciences, Stanford University School of Medicine, Stanford, United States(Woldeamanue):292–9. Available from: http://www.elsevier.com/locate/jns

270. Li WJ, Gao ZY, He Y, Liu GZ, Gao XG. Application and performance of two stroke outcome prediction models in a chinese population. PM & R : the journal of injury, function, and rehabilitation. 2012;4(2):123–8.

271. van Anh TN, Hao TK, Chi NTD, Son NH. Predictions of Hypoxic-Ischemic Encephalopathy by Umbilical Cord Blood Lactate in Newborns with Birth Asphyxia. Open Access Maced J Med Sci [Internet]. 2019;7(21):3564–7. Available from: https://www.ncbi.nlm.nih.gov/pmc/articles/PMC6986534/pdf/OAMJMS-7-3564.pdf

272. Oduwole OA, Ameh S, Esu EB, Oringanje CM, Meremikwu JT, Meremikwu MM. Assessing agreement of hemoglobin and three- fold conversion of hematocrit as methods for detecting anemia in children living in malaria-endemic areas of Calabar, Nigeria. Nigerian journal of clinical practice. 2019;22(8):1078–82.

273. Thomas N, George KC, Sridhar S, Kumar M, Kuruvilla KA, Jana AK. Whole body cooling in newborn infants with perinatal asphyxial encephalopathy in a low resource setting: A feasibility trial. Indian Pediatrics [Internet]. 2011;48(6):445–51. Available from: https://link.springer.com/content/pdf/10.1007/s13312-011-0076-z.pdf

274. Demirel B. Lactate levels and pneumonia severity index are good predictors of in-hospital mortality in pneumonia. The clinical respiratory journal. 2018;12(3):991–5.

275. Calice-Silva V, Sacomboio E, Raimann JG, Evans R, dos Santos Sebastiao C, Tchivango AT, et al. Diagnostic performance of salivary urea nitrogen dipstick to detect and monitor acute kidney disease in patients with malaria. Malaria journal [Internet]. 2018;17(1):477. Available from: https://www.ncbi.nlm.nih.gov/pmc/articles/PMC6299494/pdf/12936_2018_Article_2627.pdf

276. Bhalla A, Gupta OP, Gupta SB. Predicting mortality in stroke. Neurology India. 2002;50(3):279–81.

277. Shah BA, Ahmed W, Dhobi GN, Shah NN, Khursheed SQ, Haq I. Validity of pneumonia severity index and CURB-65 severity scoring systems in community acquired pneumonia in an Indian setting. The Indian journal of chest diseases & allied sciences. 2010;52(1):9–17.

278. Ge Y, Wang Q, Wang L, Wu H, Peng C, Wang J, et al. Predicting post-stroke pneumonia using deep neural network approaches. International journal of medical informatics. 2019;132(ct4, 9711057):103986.

279. Zhang Q, Dong G, Zhao X, Li CS. High immunoglobulin E values at admission predict mortality in ED patients with sepsis. The American journal of emergency medicine. 2016;34(8):1589–94.

280. Mathur NB, Garg K, Kumar S. Respiratory distress in neonates with special reference to pneumonia. Indian Pediatr [Internet]. 2002; Available from: http://imsear.searo.who.int/handle/123456789/8381

281. Lindblade KA, Katungu I, Wilson ML. Fever and malaria in highland Uganda. Transactions of the Royal Society of Tropical Medicine and Hygiene [Internet]. 2001;95(5):502–3. Available from: https://academic.oup.com/trstmh/article-abstract/95/5/502/1919166?redirectedFrom=fulltext

282. Vincent-Lambert C, Smith CM, Goldstein LN. Hypothermia in trauma patients arriving at an emergency department by ambulance in Johannesburg, South Africa: a prospective study. The Pan African medical journal [Internet]. 2018;31:136. Available from: https://www.ncbi.nlm.nih.gov/pmc/articles/PMC6462367/pdf/PAMJ-31-136.pdf

283. Bhaskaran K, Ebonyi AO, Walther B, Walther M. Predictors of hyperlactataemia among children presenting with malaria in a low transmission area in The Gambia. Malaria journal [Internet]. 2013;12(101139802):423. Available from: https://malariajournal.biomedcentral.com/track/pdf/10.1186/1475-2875-12-423.pdf

284. Huerga H, Ferlazzo G, Wanjala S, Bastard M, Bevilacqua P, Ardizzoni E, et al. Mortality in the first six months among HIV-positive and HIV-negative patients empirically treated for tuberculosis. BMC infectious diseases [Internet]. 2019;19(1):132. Available from: https://www.ncbi.nlm.nih.gov/pmc/articles/PMC6369550/pdf/12879_2019_Article_3775.pdf

285. Zhao Y, Nie HX, Hu K, Wu XJ, Zhang YT, Wang MM, et al. Abnormal immunity of non-survivors with COVID-19: predictors for mortality. Infectious diseases of poverty [Internet]. 2020;9(1):108. Available from: https://www.ncbi.nlm.nih.gov/pmc/articles/PMC7396941/pdf/40249_2020_Article_723.pdf

286. Wang S, Ma P, Zhang S, Song S, Wang Z, Ma Y, et al. Fasting blood glucose at admission is an independent predictor for 28-day mortality in patients with COVID-19 without previous diagnosis of diabetes: a multi-centre retrospective study. Diabetologia [Internet]. 2020;63(10):2102–11. Available from: https://www.ncbi.nlm.nih.gov/pmc/articles/PMC7347402/pdf/125_2020_Article_5209.pdf

287. Xu J, Tao Y, Xie X, Liu G, Wang A, Wang Y, et al. A Comparison of Mortality Prognostic Scores in Ischemic Stroke Patients. Journal of stroke and cerebrovascular diseases : the official journal of National Stroke Association. 2016;25(2):241–7.

288. Oguche S, Omokhodion SI, Adeyemo AA, Olumese PE. Low plasma bicarbonate predicts poor outcome of cerebral malaria in Nigerian children. West African journal of medicine. 2002;21(4):276–9.

289. Huang Y, Cai C, Zang J, Xie J, Xu D, Zheng F, et al. Treatment strategies of hospitalized patients with coronavirus disease-19. Aging [Internet]. 2020;12(12):11224–37. Available from: https://www.ncbi.nlm.nih.gov/pmc/articles/PMC7343487/pdf/aging-12-103370.pdf

290. P M, Awasthi S. Predicting Complicated Parapneumonic Effusion in Community Acquired Pneumonia: Hospital Based Case-Control Study. Indian journal of pediatrics [Internet]. 2019;86(2):140–7. Available from: https://link.springer.com/content/pdf/10.1007/s12098-018-2769-y.pdf

291. Andrijevic L, Milutinov S, Andrijevic I, Jokic D, Vukoja M. Association between the inflammatory biomarkers and left ventricular systolic dysfunction in patients with exacerbations of chronic obstructive pulmonary disease. Balkan Medical Journal [Internet]. 2017;34(3):226–31. Available from: http://cms.galenos.com.tr/FileIssue/28/1216/article/226-231.pdf

292. Wang R, He M, Yin W, Liao X, Wang B, Jin X, et al. The Prognostic Nutritional Index is associated with mortality of COVID-19 patients in Wuhan, China. Journal of clinical laboratory analysis [Internet]. 2020;34(10):e23566-. Available from: https://www.ncbi.nlm.nih.gov/pmc/articles/PMC7595894/pdf/JCLA-34-e23566.pdf

293. Nadjm B, Amos B, Mtove G, Ostermann J, Chonya S, Wangai H, et al. WHO guidelines for antimicrobial treatment in children admitted to hospital in an area of intense Plasmodium falciparum transmission: prospective study. BMJ (Clinical research ed) [Internet]. 2010;340(8900488, bmj, 101090866):c1350-. Available from: https://www.bmj.com/content/bmj/340/bmj.c1350.full.pdf

294. Medetalibeyoglu A, Catma Y, Senkal N, Ormeci A, Cavus B, Kose M, et al. The effect of liver test abnormalities on the prognosis of COVID-19. Annals of hepatology [Internet]. 2020;19(6):614–21. Available from: https://www.ncbi.nlm.nih.gov/pmc/articles/PMC7481800/pdf/main.pdf

295. Hermans SM, Babirye JA, Mbabazi O, Kakooza F, Colebunders R, Castelnuovo B, et al. Treatment decisions and mortality in HIV-positive presumptive smear-negative TB in the Xpert TM MTB/RIF era: a cohort study. BMC infectious diseases [Internet]. 2017;17(1):433. Available from: https://www.ncbi.nlm.nih.gov/pmc/articles/PMC5473987/pdf/12879_2017_Article_2534.pdf

296. Chiabi A, Mbanga C, Mah E, Nguefack Dongmo F, Nguefack S, Fru F, et al. Weight-for-Height Z Score and Mid-Upper Arm Circumference as Predictors of Mortality in Children with Severe Acute Malnutrition. Journal of tropical pediatrics [Internet]. 2017;63(4):260–6. Available from: https://watermark.silverchair.com/fmw083.pdf?token=AQECAHi208BE49Ooan9kkhW_Ercy7Dm3ZL_9Cf3qfKAc485ysgAAAqIwggKeBgkqhkiG9w0BBwagggKPMIICiwIBADCCAoQGCSqGSIb3DQEHATAeBglghkgBZQMEAS4wEQQMOgIN5MN3ASa4_UrlAgEQgIICVY7W8iV4T0la__DEFpM2q4Punc21TfcRAth8jHlSR2PBuPqY

297. Chen R, Xing L, You C. Nutritional risk screening 2002 should be used in hospitalized patients with chronic obstructive pulmonary disease with respiratory failure to determine prognosis: A validation on a large Chinese cohort. European journal of internal medicine. 2016;36(9003220):e16–7.

298. Rathore JA, Kango ZA, Mehraj A. Predictors of mortality after acute stroke a prospective hospital based study. Journal of Ayub Medical College, Abbottabad : JAMC. 2011;23(2):144–6.

299. Sun W, Xian Y, Huang Y, Sun W, Liu R, Li F, et al. Obesity is associated with better survival and functional outcome after acute intracerebral hemorrhage. Journal of the neurological sciences. 2016;370(jbj, 0375403):140–4.

300. Hosoglu S, Geyik MF, Balik I, Aygen B, Erol S, Aygencel TG, et al. Predictors of outcome in patients with tuberculous meningitis. The international journal of tuberculosis and lung disease : the official journal of the International Union against Tuberculosis and Lung Disease. 2002;6(1):64–70.

301. Hancerli S, Somer A, Salman N, Elshana H, Demirkol D, Kanturvardar M, et al. Clinical and epidemiological characteristics of pandemic influenzae a/(H1N1) in hospitalized pediatric patients at a University Hospital, Istanbul. Cocuk Enfeksiyon Dergisi [Internet]. 2010;4(3):104–9. Available from: http://www.cocukenfeksiyon.org/eng/sayilar/21/104-109.pdf

302. El-Sony AI, Mustafa SA, Khamis AH, Sobhi S, Enarson DA, Baraka OZ, et al. Symptoms in patients attending services for diagnosis of pulmonary tuberculosis in Sudan. The international journal of tuberculosis and lung disease : the official journal of the International Union against Tuberculosis and Lung Disease. 2003;7(6):550–5.

303. Liu FY, Sun XL, Zhang Y, Ge L, Wang J, Liang X, et al. Evaluation of the Risk Prediction Tools for Patients With Coronavirus Disease 2019 in Wuhan, China: A Single-Centered, Retrospective, Observational Study. Critical care medicine [Internet]. 2020;48(11):e1004–11. Available from: https://www.ncbi.nlm.nih.gov/pmc/articles/PMC7448719/pdf/ccm-48-10.1097.ccm.0000000000004549.pdf

304. Stephensen CB, Franchi LM, Hernandez H, Campos M, Gilman RH, Alvarez JO. Adverse effects of high-dose vitamin A supplements in children hospitalized with pneumonia. Pediatrics [Internet]. 1998;101(5):E3-. Available from: https://pediatrics.aappublications.org/content/pediatrics/101/5/e3.full.pdf

305. Sha L, Cao L, Chen HZ, Yuan Y, Zhu RN, Deng J, et al. [Analysis of clinical manifestations of 159 hospitalized children infected with 2009 novel influenza A (H1N1) virus]. Zhonghua er ke za zhi Chinese journal of pediatrics. 2010;48(8):575–9.

306. Raffo L. [Influenza A(H1N1) epidemic in Argentina. Experience in a National General Hospital (Hospital Nacional Alejandro Posadas)]. Medicina. 2009;69(4):393–423.

307. Zhao J, He Y, Xu P, Liu J, Ye S, Cao Y. Serum ammonia levels on admission for predicting sepsis patient mortality at D28 in the emergency department: A 2-center retrospective study. Medicine [Internet]. 2020;99(11):e19477-. Available from: https://www.ncbi.nlm.nih.gov/pmc/articles/PMC7220506/pdf/medi-99-e19477.pdf

308. Coarasa A, Giugno H, Cutri A, Loto Y, Torres F, Giubergia V, et al. Validation of a clinical prediction tool to evaluate severity in children with wheezing. Archivos Argentinos de Pediatria [Internet]. 2010;108(2):116–23. Available from: http://www.scielo.org.ar/pdf/aap/v108n2/v108n2a05.pdf

309. Kabootari M, R HT, Hasheminia M, Bozorgmanesh M, Khalili D, Akbari H, et al. Clinical features, risk factors and a prediction model for in-hospital mortality among diabetic patients infected with COVID-19: data from a referral centre in Iran. Public Health [Internet]. 2022;202((Kabootari, Habibi Tirtashi, Akbari) Metabolic Disorders Research Center, Golestan University of Medical Sciences, Gorgan, Iran, Islamic Republic of(Kabootari, Khalili, Hadaegh) Prevention of Metabolic Disorders Research Center, Research Institute for End):84–92. Available from: https://www.elsevier.com/inca/publications/store/6/4/5/7/2/7/645727.pub.htt

310. Perez-Nieto OR, Escarraman-Martinez D, Guerrero-Gutierrez MA, Zamarron-Lopez EI, Mancilla-Galindo J, Kammar-Garcia A, et al. Awake prone positioning and oxygen therapy in patients with COVID-19: The APRONOX study. European Respiratory Journal [Internet]. 2022;59(2). Available from: https://erj.ersjournals.com/content/59/2/2100265

311. Ibrahim OR, Suleiman BM, Abdullahi SB, Oloyede T, Sanda A, Gbadamosi MS, et al. Epidemiology of COVID-19 and Predictors of Outcome in Nigeria: A Single-Center Study. The American journal of tropical medicine and hygiene. 2020;103(6):2376–81.

312. Vazquez RR v, Gallardo-Rincon H, Lomelin-Gascon J, R VB, Juarez LM, Bello HH, et al. Impact of preemptive hospitalization on health outcomes at the temporary COVID-19 hospital in Mexico City: a prospective observational study. Therapeutic Advances in Infectious Disease [Internet]. 2021;8((Vazquez, Lomelin-Gascon, Ville Benavides, Juarez, Bello, Castaneda, Chavarria, Castillo, Gonzalez, Avendano, Berlanga, Loza, Wyssmann, Lezama, Romero, Ortega, Acosta, Schotman, Montoya, Rodriguez, Ramos) Temporary COVID-19 Hospital, Hipodromo de las Amer). Available from: http://tai.sagepub.com/

313. Natoubi S, Amgoun R, Baghdad N, Elkhadir MA, Lafaf A, Mouqhach R, et al. Medico-demographic characteristics and outcomes of COVID-19 patients admitted to a provincial hospital in Center-West of Morocco. The Pan African medical journal. 2022;42(101517926):268.

314. Tekten BO, Temrel TA, Sahin S. Confusion, respiratory rate, shock index (CRSI-65) score in the emergency department triage may be a new severity scoring method for community-acquired pneumonia. Saudi medical journal. 2020;41(5):473–8.

315. Omran A, Abohadid H, Mohammad MHS, Shalaby S. Salivary C-Reactive Protein and Mean Platelet Volume in the Diagnosis and Follow-Up of Community-Acquired Pneumonia in Infants. Pediatric Allergy, Immunology & Pulmonology [Internet]. 2021;34(4):141–6. Available from: https://search.ebscohost.com/login.aspx?direct=true&db=rzh&AN=154335865&site=ehost-live

316. Sneha LM, SakthiKumar L, Kadiyala A, Scott J, Jayaraman D. Implications of undernutrition in children with acute lymphoblastic leukaemia during induction therapy -- experience from a developing country. Malaysian Journal of Nutrition [Internet]. 2022;28(1):79–86. Available from: https://search.ebscohost.com/login.aspx?direct=true&db=rzh&AN=156684951&site=ehost-live

317. Xiong M, Wang L, Su L, Luo W, Li Y, Li L, et al. Acute kidney injury among hospitalized children with cancer. Pediatric Nephrology [Internet]. 2021;36(1):171–9. Available from: https://search.ebscohost.com/login.aspx?direct=true&db=rzh&AN=147268466&site=ehost-live

318. Zahari N, Yeoh SL, Muniandy SR, Bah MNM, Mat Bah MN. Pediatric Rheumatic Heart Disease in a Middle-Income Country: A Population-Based Study. Journal of Tropical Pediatrics [Internet]. 2022;68(1):1–9. Available from: https://search.ebscohost.com/login.aspx?direct=true&db=rzh&AN=156085887&site=ehost-live

319. Karahan I, Cifci A. Are Lipoprotein Levels and Ratios Able to Predict Mortality due to Sepsis?. Journal of the College of Physicians and Surgeons--Pakistan : JCPSP. 2020;30(3):272–5.

320. Rueda Z v, Bermudez M, Restrepo A, Garces C, Morales O, Roya-Pabon C, et al. Induced sputum as an adequate clinical specimen for the etiological diagnosis of community-acquired pneumonia (CAP) in children and adolescents. International Journal of Infectious Diseases [Internet]. 2022;116((Rueda, Lopez, Aguilar, Vera) Facultad de Medicina, Universidad Pontificia Bolivariana, Medellin, Colombia(Rueda, Herrera) Department of Medical Microbiology and Infectious Diseases, University of Manitoba, Winnipeg, Canada(Bermudez, Velez) Grupo Investig):348–54. Available from: https://www.journals.elsevier.com/international-journal-of-infectious-diseases

321. Leela-amornsin S, Triganjananun C, Yuksen C, Jenpanitpong C, Watcharakitpaisan S. Clinical Prediction Score for Successful Weaning from Noninvasive Positive Pressure Ventilation (NIPPV) in Emergency Department; a Retrospective Cohort Study. Archives of Academic Emergency Medicine [Internet]. 2022;10(1):e79-. Available from: http://journals.sbmu.ac.ir/aaem/index.php/AAEM/issue/archive

322. Samsami M, Mehravaran E, Tabarsi P, Javadi A, Arsang-Jang S, Komaki A, et al. Clinical and demographic characteristics of patients with COVID-19 infection: Statistics from a single hospital in Iran. Human antibodies. 2021;29(1):49–54.

323. Ospina-Serrano AV, Bruges R, Ramos P, Bernal L, Aruachan S, Quiroga A, et al. Impact of health insurance type on access to vaccination and mortality due to COVID-19 on patients with cancer in Colombia: a cohort study. Lancet Oncology [Internet]. 23 1077-4114 (Print):S39–S39. Available from: https://search.ebscohost.com/login.aspx?direct=true&db=rzh&AN=157950223&site=ehost-live

324. Estedlal A, Jeddi M, Heydari ST, Jahromi MG, Dabbaghmanesh MH. Impacts of diabetes mellitus on clinical and para-clinical parameters among COVID-19 patients. Journal of diabetes and metabolic disorders. 2021;20(2):1211–9.

325. Sowden M, van Weissenbruch MM, Bulabula ANH, Dramowski A, Lombard C, van Niekerk E. Impact of a Multi-Strain Probiotic on Healthcare-Associated Bloodstream Infection Incidence and Severity in Preterm Neonates. Journal of Pediatric Research [Internet]. 2022;9(4):345–53. Available from: https://search.ebscohost.com/login.aspx?direct=true&db=rzh&AN=160797351&site=ehost-live

326. Hua W, Yang W, Gu J, Wu J, Wang W, Liu Y, et al. Risk factors for right ventricular dysfunction in patients with lymphangioleiomyomatosis. Int J Cardiovasc Imaging. 2021;37(2):439–48.

327. Gainey M, Qu K, Garbern SC, Barry MA, Lee JA, Nasrin S, et al. Assessing the performance of clinical diagnostic models for dehydration among patients with cholera and undernutrition in Bangladesh. Tropical medicine & international health : TM & IH. 2021;26(11):1512–25.

328. Yadla M, Vadakkeveetil AK, Cherian A, Rahul. Clinical features and outcomes of 84 COVID-Positive hemodialysis patients in a resource poor setting from India. Saudi journal of kidney diseases and transplantation : an official publication of the Saudi Center for Organ Transplantation, Saudi Arabia. 2021;32(2):504–9.

329. Ji Y, Li X, Wang Y, Cheng L, Tian H, Li N, et al. Partial pressure of oxygen level at admission as a predictor of postoperative pneumonia after hip fracture surgery in a geriatric population: a retrospective cohort study. BMJ Open [Internet]. 2021;11(10):e048272-. Available from: http://bmjopen.bmj.com/content/early/by/section

330. Zhang Q, Li J, Zhang Y, Gao J, Wang P, Ai M, et al. Differences in clinical characteristics and liver injury between suspected and confirmed COVID-19 patients in Jingzhou, Hubei Province of China. Medicine. 2021;100(19):e25913-.

331. Shayganfar A, Sami R, Sadeghi S, Dehghan M, Khademi N, Rikhtehgaran R, et al. Risk factors associated with intensive care unit (ICU) admission and in-hospital death among adults hospitalized with COVID-19: a two-center retrospective observational study in tertiary care hospitals. Emergency radiology. 2021;28(4):691–7.

332. Costa WN da S, Miguel JP, Prado FDS, Lula LHS de M, Amarante GAJ, Righetti RF, et al. Noninvasive ventilation and high-flow nasal cannula in patients with acute hypoxemic respiratory failure by covid-19: A retrospective study of the feasibility, safety and outcomes. Respiratory physiology & neurobiology. 2022;298(101140022):103842.

Exclusion reason: Wrong patient population (not acute illness at health facility)

1. Baker K, Ward C, Maurel A, A de CM, Smith H, Getachew D, et al. Usability and acceptability of a multimodal respiratory rate and pulse oximeter device in case management of children with symptoms of pneumonia: A cross-sectional study in Ethiopia. Acta Paediatrica, International Journal of Paediatrics [Internet]. 2021;110(5):1620–32. Available from: http://onlinelibrary.wiley.com/journal/10.1111/(ISSN)1651-2227

2. Innovative, enhanced community management of non-hypoxaemic chest-indrawing pneumonia in 2–59-month-old children: a cluster-randomised trial in Africa and Asia. BMJ Global Health. 2022 Jan;7(1):e006405.

3. Majani N, Chillo P, Slieker MG, Sharau G, Mlawi V, Mongella S, et al. Newborn Screening for Critical Congenital Heart Disease in a Low-Resource Setting; Research Protocol and Preliminary Results of the Tanzania Pulse Oximetry Study. Global Heart [Internet]. 2022 [cited 2024 Mar 28];17(1):32. Available from: https://pubmed.ncbi.nlm.nih.gov/35837363/

4. Otiangala D, Agai NO, Olayo B, Adudans S, Ng CH, Calderon R, et al. Oxygen insecurity and mortality in resource-constrained healthcare facilities in rural Kenya. Pediatric pulmonology [Internet]. 2020;55(4):1043–9. Available from: https://onlinelibrary.wiley.com/doi/pdfdirect/10.1002/ppul.24679?download=true

5. Ruangsomboon O, Limsuwat C, Praphruetkit N, Monsomboon A, Chakorn T. Nasal High Flow Oxygen Versus Conventional Oxygen Therapy for Acute Severe Asthma Patients: A Pilot Randomized Controlled Trial. Acad Emerg Med. 2020;

6. Andrews B, Semler MW, Muchemwa L, Kelly P, Lakhi S, Heimburger DC, et al. Effect of an early resuscitation protocol on in-hospital mortality among adults with sepsis and hypotension: A randomized clinical trial. JAMA - Journal of the American Medical Association [Internet]. 2017;318(13):1233–40. Available from: https://jamanetwork.com/journals/jama/articlepdf/2654854/jama_andrews_2017_oi_170091.pdf

7. Cannoodt L, Casteels I, Conard C, S DS, Devlieger H, Fonteyne Y, et al. Retinopathy of prematurity in Rwanda: a prospective multi-centre study following introduction of screening and treatment services. Eye (Basingstoke) [Internet]. 2019;((Mutangana, Mutangana, Nyemazi) Ophthalmology, King Faisal Hospital, Kigali, Rwanda(Muhizi, Muhizi) Ophthalmology, CHUB, Huye, Rwanda(Mudereva, Gisagara, Mudereva) Ophthalmology, CHUK, Kigali, Rwanda(Noe) Ophthalmology, Kabgayi Eye Hospital, Rwanda Charit). Available from: http://www.nature.com/eye/index.html

8. Cui Y, Guan S, Ding J, He Y, Li Q, Wang S, et al. Establishment and evaluation of a model for predicting 3-month mortality in Chinese patients with hepatic encephalopathy. Metabolic brain disease [Internet]. 2019;34(1):213–21. Available from: https://link.springer.com/article/10.1007%2Fs11011-018-0333-0

9. Endalamaw A, Assimamaw NT, Ayele TA, Muche AA, Zeleke EG, Wondim A, et al. Prevalence of childhood Cancer among children attending referral hospitals of outpatient Department in Ethiopia. BMC Cancer [Internet]. 2021;21(1):1–10. Available from: https://search.ebscohost.com/login.aspx?direct=true&db=rzh&AN=149247912&site=ehost-live

10. Hu M, Zhou Q, Zheng R, Li X, Ling J, Chen Y, et al. Application of high-flow nasal cannula in hypoxemic patients with COVID-19: a retrospective cohort study. BMC Pulm Med. 2020;20(1):324.

11. Investigators AC 19 CCOS (ACCCOS). Patient care and clinical outcomes for patients with COVID-19 infection admitted to African high-care or intensive care units (ACCCOS): a multicentre, prospective, observational cohort study. Lancet (London, England). 2021;397(10288):1885–94.

12. Nisar YB. Community-based amoxicillin treatment for fast breathing pneumonia in young infants 7-59 days old: A cluster randomised trial in rural Bangladesh, Ethiopia, India and Malawi. BMJ Global Health [Internet]. 2021;6(8):e006578-. Available from: https://gh.bmj.com/

13. Ramji S, Rasaily R, Mishra PK, Narang A, Jayam S, Kapoor AN, et al. Resuscitation of asphyxiated newborns with room air or 100% oxygen at birth: a multicentric clinical trial. Indian Pediatr [Internet]. 2003; Available from: http://imsear.searo.who.int/handle/123456789/8402

14. Sohrabi MR, Amin R, Maher A, Bahadorimonfared A, Janbazi S, Hannani K, et al. Sociodemographic determinants and clinical risk factors associated with COVID-19 severity: a cross-sectional analysis of over 200,000 patients in Tehran, Iran. BMC infectious diseases. 2021;21(1):474.

15. Wassie M, Fentie B, Asefa T. Human immune deficiency virus among cervical cancer patients at Tikur Anbessa Specialized Hospital, Ethiopia: a cross sectional study. BMC Women's Health [Internet]. 2021;21(1):1–7. Available from: https://search.ebscohost.com/login.aspx?direct=true&db=rzh&AN=151818549&site=ehost-live

16. Zuo L, Dong Y, Zhu R, Jin Z, Li Z, Wang Y, et al. Screening for cognitive impairment with the Montreal Cognitive Assessment in Chinese patients with acute mild stroke and transient ischaemic attack: a validation study. BMJ open [Internet]. 2016;6(7):e011310-. Available from: https://www.ncbi.nlm.nih.gov/pmc/articles/PMC4947786/pdf/bmjopen-2016-011310.pdf

17. Maitland K, Kiguli S, Olupot-Olupot P, Hamaluba M, Thomas K, Alaroker F, et al. Randomised controlled trial of oxygen therapy and high-flow nasal therapy in African children with pneumonia. Intensive care medicine. 2021;47(5):566–76.

18. Samprathi M, Agarwal A, Jayashree M, Bansal A, Baranwal A, Nallasamy K, et al. Better Groundwork Can Avoid Troubled Waters: A Developing Country Perspective on Drowning. J Trop Pediatr [Internet]. 2020;66(4):458–60. Available from: https://watermark.silverchair.com/fmz074.pdf?token=AQECAHi208BE49Ooan9kkhW_Ercy7Dm3ZL_9Cf3qfKAc485ysgAAAsowggLGBgkqhkiG9w0BBwagggK3MIICswIBADCCAqwGCSqGSIb3DQEHATAeBglghkgBZQMEAS4wEQQM_jMn_St_EfPce_h2AgEQgIICfTfuRpEnGIk1CtAvuMoq4gyD-8UcENybZBDEN4BWsOAXmgTN

19. Eckerle M, Mvalo T, Smith AG, Kondowe D, Makonokaya D, Vaidya D, et al. Identifying modifiable risk factors for mortality in children aged 1-59 months admitted with WHO-defined severe pneumonia: a single-centre observational cohort study from rural Malawi. BMJ paediatrics open. 2022;6(1).

20. Sankar J, Pillai MS, Sankar MJ, Lodha R, Kabra SK. Clinical profile of interstitial lung disease in Indian children. Indian pediatrics. 2013;50(1):127–33.

21. Swamy R, Razak A, Mohanty P, Venkatagiri PK, Venkatesh HA, Nagesh NK, et al. Pulse Oximetry as Screening Test for Early-onset Sepsis in Newborns in Tertiary Hospitals in India. Journal of Neonatology. 2017;31(1–2):11–4.

22. Saranchuk P, Boulle A, Hilderbrand K, Coetzee D, Bedelu M, van Cutsem G, et al. Evaluation of a diagnostic algorithm for smear-negative pulmonary tuberculosis in HIV-infected adults. South African medical journal = Suid-Afrikaanse tydskrif vir geneeskunde. 2007;97(7):517–23.

23. Morrow BM, N.-Y. H, Zampoli M, Whitelaw A, Zar HJ. Pneumocystis pneumonia in South African children with and without human immunodeficiency virus infection in the era of highly active antiretroviral therapy. Pediatric Infectious Disease Journal [Internet]. 2010;29(6):535–9. Available from: http://journals.lww.com/pidj

24. Duke T, Poka H, Dale F, Michael A, Mgone J, Wal T. Chloramphenicol versus benzylpenicillin and gentamicin for the treatment of severe pneumonia in children in Papua New Guinea: a randomised trial. Lancet (London, England). 2002;359(9305):474–80.

25. Desalu OO, Oyedepo OO, Ojuawo OB, Ibraheem M, Aladesanmi AO, Suleiman ZA, et al. Acute Oxygen Therapy on Hospital Wards in Low Middle-Income Country: Experience from a Referral Centre in Ilorin, Nigeria. West African journal of medicine. 2019;36(2):122–8.

26. Araujo JG, Araujo-Melo CA, de Menezes-Neto OA, da Silveira DFC, Correia JB, Cipolotti R. Risk factors for acute chest syndrome in patients from low socioeconomic background: a cohort study from Sergipe, Brazil. Journal of pediatric hematology/oncology. 2011;33(7):484–6.

27. Chioukh FZ, K BA, K BH, Mejaouel H, Blibech S, Kebaier H, et al. Transported neonates in Tunisia: Condition at arrival and outcome. Journal of Maternal-Fetal and Neonatal Medicine [Internet]. 2016;29(Supplement 1):206. Available from: https://www.tandfonline.com/doi/full/10.1080/14767058.2016.1191212

28. Rittayamai N, Chuariyakul P, Promlee N, Chailard P, Chierakul N. Efficacy and safety of high-flow nasal oxygen cannula in patients with acute respiratory failure at general internal medicine wards: A prospective cohort study. Intensive Care Medicine Experimental [Internet]. 2019;7(Supplement 3). Available from: https://icm-experimental.springeropen.com/track/pdf/10.1186/s40635-019-0265-y.pdf

29. Gera T, Ramji S. Early predictors of mortality in very low birth weight neonates. Indian Pediatr [Internet]. 2001; Available from: http://imsear.searo.who.int/handle/123456789/11702

30. Panda SK, Nayak MK, Rath S, Mohakud NK, Panda SS. Maternal risk factors and demographic profile of neonates presenting with persistent pulmonary hypertension in a tertiary care hospital, odisha. Indian Journal of Public Health Research and Development [Internet]. 2019;10(9):468–72. Available from: http://indianjournals.com/ijor.aspx?target=ijor:ijphrd&volume=10&issue=9&article=085&type=pdf

31. Bansal A, Singhi SC, Jayashree M. Penicillin and gentamicin therapy vs amoxicillin/clavulanate in severe hypoxemic pneumonia. Indian J Pediatr [Internet]. 2006; Available from: http://imsear.searo.who.int/handle/123456789/79990

32. Lutohin GM, Geraskina LA, Fonyakin A v. [Sleep-disordered breathing syndrome in acute ischemic stroke]. Zhurnal nevrologii i psikhiatrii imeni SS Korsakova. 2016;116(12. Vyp. 2):14–20.

33. TANG X yan, Ji LI, DONG F, SONG H mei. Clinical characteristics of Pneumocystis carinii pneumonia in children with systemic lupus erythematosus. Chinese Journal of Pediatrics [Internet]. 2013;(12):920–4. Available from: http://dx.doi.org/

34. Lira L, Celeste D, Garanito M, Carneiro J. Pulmonary embolism in pediatric patients: A ten-year experience from a tertiary center in brazil. Research and Practice in Thrombosis and Haemostasis [Internet]. 2020;4(SUPPL 1):664. Available from: https://onlinelibrary.wiley.com/doi/pdfdirect/10.1002/rth2.12393?download=true

35. Pulsan F, Duke T. Response to oxygen therapy using oxygen concentrators run off solar power in children with respiratory distress in remote primary health facilities in Papua New Guinea. Tropical Doctor. 2020;49475520947886-.

36. Bjorklund AR, Odongkara Mpora B, Steiner ME, Fischer G, Davey CS, Slusher TM. Use of a modified bubble continuous positive airway pressure (bCPAP) device for children in respiratory distress in low- and middle-income countries: a safety study. Paediatr Int Child Health [Internet]. 2019;39(3):160–7. Available from: https://www.tandfonline.com/doi/pdf/10.1080/20469047.2018.1474698?needAccess=true

37. Rusmawati A, Haksari EL, Naning R. Downes score as a clinical assessment for hypoxemia in neonates with respiratory distress. Paediatrica Indonesiana [Internet]. 2008;48(6):342–5. Available from: https://paediatricaindonesiana.org/index.php/paediatrica-indonesiana/article/download/626/487

38. Zhao X, Huang W, Li J, Liu Y, Wan M, Xue G, et al. Noninvasive Positive-Pressure Ventilation in Acute Respiratory Distress Syndrome in Patients With Acute Pancreatitis: A Retrospective Cohort Study. Pancreas. 2016;45(1):58–63.

39. Lenahan JL, Nkwopara E, Phiri M, Mvalo T, Couasnon MT, Turner K, et al. Repeat assessment of examination signs among children in Malawi with fast-breathing pneumonia. ERJ Open Research [Internet]. 2020;6(2):1–9. Available from: https://openres.ersjournals.com/content/6/2/00275-2019.full.pdf

40. Wells JM, Estepar RSJ, McDonald MLN, Bhatt SP, Diaz AA, Bailey WC, et al. Clinical, physiologic, and radiographic factors contributing to development of hypoxemia in moderate to severe COPD: a cohort study. BMC pulmonary medicine [Internet]. 2016;16(1):169. Available from: https://escholarship.org/content/qt46d0f7cv/qt46d0f7cv.pdf?t=qaeuml

41. Ilik F, Pazarli AC, Kayhan F, Karamanli H, Ozlece HK. Electrophysiological assessment in patients with long term hypoxia. Neurosciences [Internet]. 2016;21(1):26–9. Available from: http://www.neurosciencesjournal.org/PDFFiles/Jan16/01electrophysiological20150333.pdf

42. King C, Colbourn T, Mankhambo L, Beard J, Hay Burgess DC, Costello A, et al. Non-treatment of children with community health worker-diagnosed fast-breathing pneumonia in rural Malawi: exploratory subanalysis of a prospective cohort study. BMJ Open [Internet]. 2016;6(11):e011636-. Available from: https://www.ncbi.nlm.nih.gov/pmc/articles/PMC5128900/pdf/bmjopen-2016-011636.pdf

43. Correa TD, Sanches PR, de Morais LC, Scarin FC, Silva E, Barbas CSV. Performance of noninvasive ventilation in acute respiratory failure in critically ill patients: a prospective, observational, cohort study. BMC pulmonary medicine [Internet]. 2015;15(100968563):144. Available from: https://bmcpulmmed.biomedcentral.com/track/pdf/10.1186/s12890-015-0139-3.pdf

44. Muhe L, Degefu H, Worku B, Oljira B, Mulholland EK. Comparison of nasal prongs with nasal catheters in the delivery of oxygen to children with hypoxia. Journal of tropical pediatrics [Internet]. 1998;44(6):365–8. Available from: https://watermark.silverchair.com/44-6-365.pdf?token=AQECAHi208BE49Ooan9kkhW_Ercy7Dm3ZL_9Cf3qfKAc485ysgAAAs4wggLKBgkqhkiG9w0BBwagggK7MIICtwIBADCCArAGCSqGSIb3DQEHATAeBglghkgBZQMEAS4wEQQMD6XTlWPsvxCo3-kCAgEQgIICgan4zyIhZH6ZvxwW4sXLHiFi3uQJSKoImY2WXtrxuuYUkp

45. Morrow BM, Samuel CM, Zampoli M, Whitelaw A, Zar HJ. Pneumocystis pneumonia in South African children diagnosed by molecular methods. BMC research notes [Internet]. 2014;7(101462768):26. Available from: https://bmcresnotes.biomedcentral.com/track/pdf/10.1186/1756-0500-7-26.pdf

46. Phan PH, Beasley PR, Risser J, Charles F, Liem NT. Acute lung injury in Vietnamese children: Risk factors associated with mortality and prolonged mechanical ventilation. Intensive Care Medicine [Internet]. 2013;39(SUPPL. 1):S163-. Available from: https://link.springer.com/content/pdf/10.1007/s00134-013-2950-8.pdf

47. Vazquez-Perez JA, Ramirez-Gonzalez JE, Moreno-Valencia Y, Hernandez-Hernandez VA, Romero-Espinoza JAI, Castillejos-Lopez M, et al. EV-D68 infection in children with asthma exacerbation and pneumonia in Mexico City during 2014 autumn. Influenza and other Respiratory Viruses [Internet]. 2016;10(3):154–60. Available from: http://onlinelibrary.wiley.com/journal/10.1111/(ISSN)1750-2659

48. Mothabbir G, Rana S, Baqui AH, Ahmed S, Ahmed AN, Taneja S, et al. Management of fast breathing pneumonia in young infants aged 7 to 59 days by community level health workers: protocol for a multi-centre cluster randomized controlled trial. Int J Clin Trials [Internet]. 2020;7(2):83–93. Available from: https://www.ncbi.nlm.nih.gov/pmc/articles/PMC7644113/pdf/nihms-1599331.pdf

49. Zhou S, Yang Y, Zhang X, Li Z, Liu X, Hu C, et al. Clinical Course of 195 Critically Ill COVID-19 Patients: A Retrospective Multicenter Study. Shock (Augusta, Ga). 2020;54(5):644–51.

50. Kamal VK, Agrawal D, Pandey RM. Prognostic models for prediction of outcomes after traumatic brain injury based on patients admission characteristics. Brain injury. 2016;30(4):393–406.

51. Ansari NA, Kombe AH, Kenyon TA, Mazhani L, Binkin N, Tappero JW, et al. Pathology and causes of death in a series of human immunodeficiency virus-positive and -negative pediatric referral hospital admissions in Botswana. The Pediatric infectious disease journal. 2003;22(1):43–7.

52. Rao S, Lucero MG, Nohynek H, Tallo V, Lupisan SP, Garcea RL, et al. WU and KI polyomavirus infections in Filipino children with lower respiratory tract disease. J Clin Virol. 2016;82:112–8.

53. Wu M, J.-J. J, Zhong L, Z.-Y. S, Q.-F. X, Z.-Y. L, et al. Thymosin alpha1 therapy in critically ill patients with COVID-19: A multicenter retrospective cohort study. International Immunopharmacology [Internet]. 2020;88((Wu, Feng) Department of Critical Care Medicine and Hospital Infection Prevention and Control, The Second People's Hospital of Shenzhen&First Affiliated Hospital of Shenzhen University, Health Science Center, Shenzhen 518035, China(Ji, Xie, Liu, Wang, S):106873. Available from: http://www.elsevier.com/locate/intimp

54. Orimadegun AE, Ogunbosi BO, Carson SS. Validity of doctor diagnosed pneumonia in hypoxic children admitted into emergency room in Ibadan, Nigeria. American Journal of Respiratory and Critical Care Medicine [Internet]. 2011;183(1 MeetingAbstracts). Available from: http://ajrccm.atsjournals.org/cgi/reprint/183/1_MeetingAbstracts/A4935?sid=0fa73f84-6146-4836-b0e0-74e4053abff7

55. Walley J, Kunutsor S, Evans M, Thoulass J, Katabira E, Muchuro S, et al. Validation in Uganda of the new WHO diagnostic algorithm for smear-negative pulmonary tuberculosis in HIV prevalent settings. Journal of acquired immune deficiency syndromes (1999). 2011;57(5):e93-100.

56. Chisti MJ, Salam MA, Smith JH, Ahmed T, Pietroni MAC, Shahunja KM, et al. Bubble continuous positive airway pressure for children with severe pneumonia and hypoxaemia in Bangladesh: an open, randomised controlled trial. Lancet (London, England) [Internet]. 2015;386(9998):1057–65. Available from: https://www.thelancet.com/journals/lancet/article/PIIS0140-6736(15)60249-5/fulltext

57. Makani J, Kirkham FJ, Komba A, Ajala-Agbo T, Otieno G, Fegan G, et al. Risk factors for high cerebral blood flow velocity and death in Kenyan children with Sickle Cell Anaemia: role of haemoglobin oxygen saturation and febrile illness. British journal of haematology [Internet]. 2009;145(4):529–32. Available from: https://www.ncbi.nlm.nih.gov/pmc/articles/PMC3001030/pdf/bjh0145-0529.pdf

58. Dong X, Sun L, Li Y. Prognostic value of lactate dehydrogenase for in-hospital mortality in severe and critically ill patients with COVID-19. International journal of medical sciences [Internet]. 2020;17(14):2225–31. Available from: https://www.ncbi.nlm.nih.gov/pmc/articles/PMC7484664/pdf/ijmsv17p2225.pdf

59. Moreira ME, Pereira APE, Gomes Junior SC, Guinsburg R, de Almeida MFB, Gama SG, et al. Factors associated with the use of supplemental oxygen or positive pressure ventilation in the delivery room, in infants born with a gestational age >= 34 weeks. Reproductive health [Internet]. 2016;13(Suppl 3):116. Available from: https://www.ncbi.nlm.nih.gov/pmc/articles/PMC5073976/pdf/12978_2016_Article_235.pdf

60. Pulsan F, Sobi K, Duke T. Continuous positive airway pressure in children with severe pneumonia and hypoxaemia in Papua New Guinea: an evaluation of implementation. Acta Paediatrica, International Journal of Paediatrics [Internet]. 2019;108(10):1887–95. Available from: http://onlinelibrary.wiley.com/journal/10.1111/(ISSN)1651-2227

61. Singh J, Dalal P, Gathwala G. Clinical profile and predictors of mortality among the referred neonates at a tertiary care centre in north India: a prospective observational study. Tropical Doctor [Internet]. 2020;50(3):221–7. Available from: http://www.uk.sagepub.com/journals/Journal202201

62. Rabna P, Andersen A, Wejse C, Oliveira I, Gomes VF, Haaland MB, et al. High mortality risk among individuals assumed to be TB-negative can be predicted using a simple test. Tropical medicine & international health : TM & IH [Internet]. 2009;14(9):986–94. Available from: https://onlinelibrary.wiley.com/doi/pdfdirect/10.1111/j.1365-3156.2009.02328.x?download=true

63. Abrishami M, Maemori GA, Boskabadi H, Yaeghobi Z, Mafi-Nejad S, Abrishami M. Incidence and risk factors of retinopathy of prematurity in mashhad, northeast iran. Iran Red Crescent Med J [Internet]. 2013;15(3):229–33. Available from: https://www.ncbi.nlm.nih.gov/pmc/articles/PMC3745752/pdf/ircmj-15-229.pdf

64. Flanders WD, Tucker G, Krishnadasan A, Martin D, Honig E, McClellan WM. Validation of the pneumonia severity index. Importance of study-specific recalibration. Journal of general internal medicine [Internet]. 1999;14(6):333–40. Available from: https://www.ncbi.nlm.nih.gov/pmc/articles/PMC1496595/pdf/jgi_351.pdf

65. Niknafs P, Norouzi E, Bijari BB, Baneshi MR. Can we replace arterial blood gas analysis by pulse oximetry in neonates with respiratory distress syndrome, who are treated according to INSURE protocol? Iranian Journal of Medical Sciences [Internet]. 2015;40(3):264–7. Available from: http://ijms.sums.ac.ir/index.php/IJMS/article/download/26/414

66. Long L, Zeng X, Zhang X, Xiao W, Guo E, Zhan W, et al. Short-term outcomes of COVID-19 and risk factors for progression. European Respiratory Journal [Internet]. 2020;318(6):2000990. Available from: http://erj.ersjournals.com/lookup/doi/10.1183/13993003.00990-2020

67. Hoffman E, Reichmuth KL, Cooke ML. A review of the use of high-flow nasal cannula oxygen therapy in hospitalised children at a regional hospital in the Cape Town Metro, South Africa. South African Medical Journal [Internet]. 2019;109(4):272–7. Available from: http://www.samj.org.za/index.php/samj/article/download/12570/8782

68. Caballero MT, Hijano DR, Acosta PL, Mateu CG, Marcone DN, Linder JE, et al. Interleukin-13 associates with life-threatening rhinovirus infections in infants and young children. Pediatric Pulmonology [Internet]. 2018;53(6):787–95. Available from: http://onlinelibrary.wiley.com/journal/10.1002/(ISSN)1099-0496

69. Duke T, Mgone J, Frank D. Hypoxaemia in children with severe pneumonia in Papua New Guinea [oxygen therapy in children]. The International Journal of Tuberculosis and Lung Disease [Internet]. 2001;5(6):511–9. Available from: http://docserver.ingentaconnect.com/deliver/connect/iuatld/10273719/v5n6/s4.pdf?expires=1618445475&id=0000&titleid=3764&checksum=09A494ED19C7F638BBAC1363534E07F

70. Hayakawa K, Morioka S, Asai Y, Tsuzuki S, Yamada G, Suzuki S, et al. Predictors of silent hypoxia in hospitalized patients with COVID-19 in Japan. Journal of Infection and Chemotherapy [Internet]. 2022;28(10):1436–8. Available from: http://www.journals.elsevier.com/journal-of-infection-and-chemotherapy/

71. Alonazi B, Mostafa MA, Farghaly AM, Zindani SA, Al-Watban JA, Altaimi F, et al. Primary SARS-CoV-2 Pneumonia Screening in Adults: Analysis of the Correlation between High-Resolution Computed Tomography Pulmonary Patterns and Initial Oxygen Saturation Levels.

Current medical imaging. 2022;((Alonazi) Radiology and Medical Imaging Department, College of Applied Medical Sciences, Prince Sattam bin Abdulaziz University, Saudi Arabia(Mostafa, Farghaly, Zindani, Al-Watban, Altaimi, Almotairy, Fagiry) Medical Imaging Department, Prince Mohammed bi).

72. Ansems K, Grundeis F, Dahms K, Mikolajewska A, Thieme V, Piechotta V, et al. Remdesivir for the treatment of COVID-19. The Cochrane database of systematic reviews. 2021;8(100909747):CD014962-.

73. Avdeev SN, Yaroshetskiy AI, Tsareva NA, Merzhoeva ZM, Trushenko N v, Nekludova G v, et al. Noninvasive ventilation for acute hypoxemic respiratory failure in patients with COVID-19. The American journal of emergency medicine. 2021;39(aa2, 8309942):154–7.

74. Busana M, Gasperetti A, Giosa L, Forleo GB, Schiavone M, Mitacchione G, et al. Prevalence and outcome of silent hypoxemia in COVID-19. Minerva anestesiologica. 2021;87(3):325–33.

75. Buonafine CP, Paiatto BNM, Leal FB, de Matos SF, de Morais CO, Guerra GG, et al. High prevalence of SARS-CoV-2 infection among symptomatic healthcare workers in a large university tertiary hospital in São Paulo, Brazil. BMC Infectious Diseases [Internet]. 2020;20(1):N.PAG-N.PAG. Available from: https://search.ebscohost.com/login.aspx?direct=true&db=rzh&AN=147337856&site=ehost-live

76. Kazemi Aski S, Norooznezhad AH, Mostafaei S, Aleyasin A, Nabavian SM, Alimohammadi S, et al.\ Clinical features and risk factors associated with acute respiratory distress syndrome in pregnant women diagnosed with COVID-19: a multi-center case-control study. Journal of Maternal-Fetal and Neonatal Medicine [Internet]. 2021;((Kazemi Aski, Zarean, Hantoushzadeh) Reproductive Health Research Center, Department of Obstetrics and Gynecology, Guilan University of Medical Scieneces, Rasht, Iran, Islamic Republic of(Norooznezhad) Medical Biology Research Center, Health Technology In). Available from: https://www.tandfonline.com/loi/ijmf20

77. Gashynova K, But N, Rudakova V, Suska K, Dmytrychenko V. A Retrospective Single-Center Analysis of COVID-19 Patients with Fatal Outcome. American Journal of Respiratory and Critical Care Medicine [Internet]. 2022;205(1). Available from: https://www.atsjournals.org/doi/abs/10.1164/ajrccm-conference.2022.205.1_MeetingAbstracts.A4586

78. Akhavizadegan H, Hosamirudsari H, Alizadeh M, Alimohamadi Y, Karbakhsh Davari M, Akbarpour S, et al. Can laboratory tests at the time of admission guide us to the prognosis of patients with COVID-19?. Journal of preventive medicine and hygiene. 2021;62(2):E321–5.

79. Amare A, Melkamu Y, Mekonnen D. Tetanus in adults: Clinical presentation, treatment and predictors of mortality in a tertiary hospital in Ethiopia. Journal of the Neurological Sciences. 2012;317(1–2):62–5.

80. Sobral MFF, Roazzi A, da Penha Sobral AIG, de Oliveira BRB, Duarte GB, da Silva JF, et al. A retrospective cohort study of 238,000 COVID-19 hospitalizations and deaths in Brazil. Scientific reports. 2022;12(1):3629.

81. Louie A, Feiner JR, Bickler PE, Rhodes L, Bernstein M, Lucero J. Four types of pulse oximeters accurately detect hypoxia during low perfusion and motion. Anesthesiology: The Journal of the American Society of Anesthesiologists. 2018;128(3):520–30.

82. Zeng M, Chang M, Zheng H, Li B, Chen Y, He W, et al. Clinical value of soluble urokinase-type plasminogen activator receptor in the diagnosis, prognosis, and therapeutic guidance of sepsis. The American journal of emergency medicine. 2016;34(3):375–80.

83. Chisti MJ. Risk factors for death in severely malnourished under-five children presenting with severe/ very severe pneumonia in an urban critical care ward in a developing country. Indian Journal of Critical Care Medicine. 2013;17(SUPPL. 2):46–7.

84. Cao M, Sheng J, Qiu X, Wang D, Wang D, Wang Y, et al. Acute exacerbations of fibrosing interstitial lung disease associated with connective tissue diseases: a population-based study. BMC pulmonary medicine [Internet]. 2019;19(1):215. Available from: https://www.ncbi.nlm.nih.gov/pmc/articles/PMC6857302/pdf/12890_2019_Article_960.pdf

85. Rao R, Ramji S. Pulse oximetry in asphyxiated newborns in the delivery room. Indian Pediatr [Internet]. 2001; Available from: http://imsear.searo.who.int/handle/123456789/12154

86. Tikmani SS, Muhammad AA, Shafiq Y, Shah S, Kumar N, Ahmed I, et al. Ambulatory Treatment of Fast Breathing in Young Infants Aged <60 Days: A Double-Blind, Randomized, Placebo-Controlled Equivalence Trial in Low-Income Settlements of Karachi. Clinical infectious diseases : an official publication of the Infectious Diseases Society of America [Internet]. 2017;64(2):184–9. Available from: https://watermark.silverchair.com/ciw690.pdf?token=AQECAHi208BE49Ooan9kkhW_Ercy7Dm3ZL_9Cf3qfKAc485ysgAAAsAwggK8BgkqhkiG9w0BBwagggKtMIICqQIBADCCAqIGCSqGSIb3DQEHATAeBglghkgBZQMEAS4wEQQM6g0kUi98Vt-JykdWAgEQgIICc3AiUcch5GJsyhern9MotB_mvXq5ftxnk0H_1y7WJlAF9Acv

87. Yan-Ping ZHU, WANG L, Ming-Xia LI. Correlation between right ventricular function and the indexes of blood gas in neonates with hypoxic pulmonary hypertension. Chinese Journal of Contemporary Pediatrics [Internet]. 2010;(12):436–9. Available from: http://dx.doi.org/

88. Ying REN, Shu LIU, YANG YM, Hong-Jun LIU. Risk factors for capillary leak syndrome in children with hematological malignancies. Journal of Southern Medical University [Internet]. 2015;(12):606–9. Available from: http://dx.doi.org/

89. Pan F, Yang L, Li Y, Liang B, Li L, Ye T, et al. Factors associated with death outcome in patients with severe coronavirus disease-19 (COVID-19): a case-control study. International journal of medical sciences [Internet]. 2020;17(9):1281–92. Available from: https://www.ncbi.nlm.nih.gov/pmc/articles/PMC7294915/pdf/ijmsv17p1281.pdf

90. Morre R, Sobi K, Pameh W, Ripa P, Vince JD, Duke T. Safety, Effectiveness and Feasibility of Outpatient Management of Children with Pneumonia with Chest Indrawing at Port Moresby General Hospital, Papua New Guinea. Journal of Tropical Pediatrics [Internet]. 2019;65(1):71–7. Available from: http://tropej.oxfordjournals.org/

91. Moreira FB, Rosario CS, Santos JS, Avanzi VM, Nogueira MB, Vidal LR, et al. Molecular characterization and clinical epidemiology of human respiratory syncytial virus (HRSV) A and B in hospitalized children, Southern Brazil. Journal of Medical Virology [Internet]. 2017;89(8):1489–93. Available from: http://onlinelibrary.wiley.com/journal/10.1002/(ISSN)1096-9071

92. Attia EF, Weiss NS, E MO, McGrath CJ, Cagle A, West TE, et al. Risk Factors for Hypoxia and Tachypnea Among Adolescents With Vertically-acquired HIV in Nairobi. Pediatric Infectious Disease Journal [Internet]. 2017;36(4):e93–7. Available from: http://journals.lww.com/pidj

93. Pandian JD, Kaur A, Jyotsna R, Sylaja PN, Vijaya P, Padma M v, et al. Complications in acute stroke in India (CAST-I): a multicenter study. Journal of stroke and cerebrovascular diseases : the official journal of National Stroke Association. 2012;21(8):695–703.

94. Yang Y, Guo F, Zhao W, Gu Q, Huang M, Cao Q, et al. Novel avian-origin influenza a (H7N9) in critically ill patients in China. Critical Care Medicine [Internet]. 2015;43(2):339–45. Available from: http://journals.lww.com/ccmjournal/pages/default.aspx

95. Askie LM, Henderson-Smart DJ, Irwig L, Simpson JM. Oxygen-saturation targets and outcomes in extremely preterm infants. New England Journal of Medicine. 2003;349(10):959–67.

96. Escobar G, Matta J, Taype W, Ayala R, Amado J. CLINICOEPIDEMIOLOGICAL CHARACTERISTICS of PATIENTS WHO DIED from COVID-19 at A NATIONAL HOSPITAL of LIMA, Peru. Revista de la Facultad de Medicina Humana [Internet]. 2020;20(2):180–5. Available from: http://revistas.urp.edu.pe/index.php/RFMH/article/view/2940

97. Agrawal A, Agarwal S, Kumar V, Nawal CL, Mital P, Chejara R. A study of an influenza A (H1N1)pdm09 outbreak in pregnant women in Rajasthan, India. International journal of gynaecology and obstetrics: the official organ of the International Federation of Gynaecology and Obstetrics. 2016;132(2):146–50.

Exclusion reason: Wrong setting (not low- or middle-income country; intensive care setting)

1. Brouqui P, Amrane S, Million M, Cortaredona S, Parola P, Lagier JC, et al. Asymptomatic hypoxia in COVID-19 is associated with poor outcome. Int J Infect Dis. 2021;102:233–8.

2. Haidar MK, Vogt F, Takahashi K, Henaff F, Umphrey L, Morton N, et al. Suspected paracetamol overdose in Monrovia, Liberia: A matched case-control study. BMC Pediatrics [Internet]. 2020;20(1):139. Available from: http://www.biomedcentral.com/bmcpediatr/

3. Luis Enrique Vega-Briceño, Marcela Potin S, Marcela Ferrés G, Dahiana Pulgar B, Cristián García B, Linus Holmgren P, et al. Características clínicas de la enfermedad respiratoria causada por Mycoplasma pneumoniae en niños hospitalizados. Revista Chilena De Infectología. 2009 Aug 1;26(4).

4. Scott JW, Nyinawankusi JD, Enumah S, Maine R, Uwitonze E, Hu Y, et al. Improving prehospital trauma care in Rwanda through continuous quality improvement: an interrupted time series analysis. Injury. 2017;48(7):1376–81.

5. Dawood FS, Fry AM, Goswami D, Sharmeen A, Nahar K, Anjali BA, et al. Incidence and characteristics of early childhood wheezing, Dhaka, Bangladesh, 2004-2010. Pediatric pulmonology [Internet]. 2016;51(6):588–95. Available from: https://onlinelibrary.wiley.com/doi/abs/10.1002/ppul.23343

6. Vilar-Compte D, Shah DP, Vanichanan J, Cornejo-Juarez P, Garcia-Horton A, Volkow P, et al. Influenza in patients with hematological malignancies: Experience at two comprehensive cancer centers. Journal of Medical Virology [Internet]. 2018;90(1):50–60. Available from: https://www.ncbi.nlm.nih.gov/pmc/articles/PMC5761331/pdf/nihms906703.pdf

7. Newberry JA, Bills CB, Matheson L, Zhang X, Gimkala A, Ramana Rao G v, et al. A profile of traumatic injury in the prehospital setting in India: A prospective observational study across seven states. Injury. 2020;51(2):286–93.

8. Wang R, Pan M, Zhang X, Han M, Fan X, Zhao F, et al. Epidemiological and clinical features of 125 Hospitalized Patients with COVID-19 in Fuyang, Anhui, China. International Journal of Infectious Diseases [Internet]. 2020;95((Wang, Pan, Zhang, Fan, Miao, Guan, Chen) Department of Geriatric Respiratory and Critical Care, the First Affiliated Hospital of Anhui Medical University, Jixi Road 218, Hefei, Anhui 230022, China(Wang, Han, Zhao, Xu, Deng, Shen) Fuyang Infectious Diseas):421–8. Available from: https://www.journals.elsevier.com/international-journal-of-infectious-diseases

9. Horn AR, Swingler GH, Myer L, Linley LL, Chandrasekaran M, Robertson NJ. Early clinical predictors of a severely abnormal amplitude-integrated electroencephalogram at 48 hours in cooled neonates. Acta paediatrica (Oslo, Norway : 1992) [Internet]. 2013;102(8):e378-84. Available from: https://onlinelibrary.wiley.com/doi/abs/10.1111/apa.12306

10. Karamercan MA, Dündar ZD, Ergin M, Meer VANO, Body R, Harjola VP, et al. Seasonal variations of patients presenting dyspnea to emergency departments in Europe: Results from the EURODEM Study. Turk J Med Sci. 2020;50(8):1879–86.

11. Kelly AM, McAlpine R, Kyle E. How accurate are pulse oximeters in patients with acute exacerbations of chronic obstructive airways disease? Respiratory medicine. 2001;95(5):336–40.

12. Mueller SM, Ackermann BW, Martin S, Seifert K, Mohr A, Alali W, et al. Incidence of Intermittent Hypoxemia Increases during Clinical Care and Parental Touch in Extremely Preterm Infants. Neonatology [Internet]. 2022;((Mueller, Martin, Seifert, Mohr, Alali, Grunwald) Haptic Research Lab, Paul-Flechsig-Institute for Brain Research and Neuropathology, University of Leipzig, Leipzig, Germany(Ackermann, Thome) Department of Neonatology, University Hospital Leipzig, Leipzig). Available from: http://content.karger.com/ProdukteDB/produkte.asp?Aktion=JournalHome&ProduktNr=232056

13. Sener MU, Cicek T, Ozturk A. Highlights of clinical and laboratory parameters among severe COVID-19 patients treated with tocilizumab: a retrospective observational study. Sao Paulo medical journal = Revista paulista de medicina. 2022;140(5):627–35.

14. Shi T, Chen C, Huang L, Fan H, Lu G, Yang D, et al. Risk factors for mortality from severe community-acquired pneumonia in hospitalized children transferred to the pediatric intensive care unit. Pediatrics and neonatology. 2020;61(6):577–83.

15. Zar HJ, Hanslo D, Tannenbaum E, Klein M, Argent A, Eley B, et al. Aetiology and outcome of pneumonia in human immunodeficiency virus-infected children hospitalized in South Africa. Acta paediatrica (Oslo, Norway : 1992). 2001;90(2):119–25.

16. Vedovati MC, Cimini LA, Pierpaoli L, Vanni S, Cotugno M, Pruszczyk P, et al. Prognostic value of respiratory index in haemodynamically stable patients with acute pulmonary embolism: The Respiratory Index model study. European heart journal Acute cardiovascular care. 2020;9(4):286–92.

17. Fernandez AR, Crowe RP, Bourn S, Matt SE, Brown AL, Hawthorn AB, et al. COVID-19 Preliminary Case Series: Characteristics of EMS Encounters with Linked Hospital Diagnoses. Prehosp Emerg Care. 2021;25(1):16–27.

18. Maira D, Duca L, Busti F, Consonni D, Salvatici M, Vianello A, et al. The role of hypoxia and inflammation in the regulation of iron metabolism and erythropoiesis in COVID-19: The IRONCOVID study. American Journal of Hematology [Internet]. 2022;97(11):1404–12. Available from: http://onlinelibrary.wiley.com/journal/10.1002/(ISSN)1096-8652

19. Kudavidanage BP, Jayasundara D, Jayasundara S, Wijekoon ND, DIssanayake SD. Current clinical practices, immediate complications and associated risk factors of emergency tracheal intubation in a tertiary-care hospital in sri lanka. Anaesthesia and Intensive Care. 2020;48(2 SUPPL):61.

20. Chuaychoo B, Rattanasaengloet K, Banlengchit R, Horthongkham N, Athipanyasilp N, Totanarungroj K, et al. Characteristics, complications, and mortality of respiratory syncytial virus compared with influenza infections in hospitalized adult patients in Thailand. International Journal of Infectious Diseases [Internet]. 2021;110((Chuaychoo, Rattanasaengloet) Division of Respiratory Disease and Tuberculosis, Department of Medicine, Faculty of Medicine Siriraj Hospital, Mahidol University, Bangkok, Thailand(Banlengchit) Tufts Medical Center, Boston, MA, United States(Horthongkham,):237–46. Available from: https://www.journals.elsevier.com/international-journal-of-infectious-diseases

21. Rees CA, Colbourn T, Hooli S, King C, Lufesi N, McCollum ED, et al. Derivation and validation of a novel risk assessment tool to identify children aged 2-59 months at risk of hospitalised pneumonia-related mortality in 20 countries. BMJ Global Health [Internet]. 2022;7(4):e008143-. Available from: https://gh.bmj.com/

22. Huguet ET, Iturriaga LAR, Fernandez LS, Yandiola PPE, Mendez R, Zorrozua BS, et al. COVID-19 in young and middle aged adults. Predictors of poor evolution and clinical differences. European Respiratory Journal [Internet]. 2021;58(SUPPL 65). Available from: https://erj.ersjournals.com/content/58/suppl_65/OA88

23. Huang TT, Zhao WX, Lin JH. Risk Factors for Maternal and Perinatal Complications during Pregnancy among Women with Tetralogy of Fallot. Nigerian journal of clinical practice. 2021;24(8):1138–43.

24. Miao H, Chen Y, Wang C, Huang T, Lin J. Pregnancies in women with moderate and severe pulmonary hypertension remain challenging: A single-center experience in East China. International Journal of Gynecology and Obstetrics [Internet]. 2022;157(1):140–8. Available from: http://obgyn.onlinelibrary.wiley.com/hub/journal/10.1002/(ISSN)1879-3479/

25. Hernández-Cárdenas CM, Mendoza-Copa G, Hong-Zhu P, Gómez-García IA, Lugo-Goytia G. A MULTIVARIATE PROGNOSTIC SCORE FOR PREDICTING MORTALITY OF ACQUIRED IMMUNODEFICIENCY SYNDROME PATIENTS WITH HYPOXEMIC RESPIRATORY FAILURE AND PNEUMOCYSTIS JIROVECI PNEUMONIA. Rev Invest Clin. 2019;71(5):311–20.

26. CHUNG WS, Kyung-Man CHA, Hyung-Min KIM, JEONG WJ, Byung-Hak SO. Risk Factors for Aspiration Pneumonia in Acute Benzodiazepine Overdose. Journal of The Korean Society of Clinical Toxicology [Internet]. 2016;26–32. Available from: http://dx.doi.org/

27. Jun-Sung LEE, MOON T, Tae-Hoon KIM, Se-Young KIM, CHOI JY, Kyung-Bok LEE, et al. Deep Vein Thrombosis in Patients with Pulmonary Embolism: Prevalance, Clinical Significance and Outcome. Vascular Specialist International [Internet]. 2016;166–74. Available from: http://dx.doi.org/10.5758/vsi.2016.32.4.166

28. CHOI BH, SONG KB, SHIM JY, HONG SJ. Prospective Randomized Study Comparing L-epinephrine and Budesonide Aerosols in the Treatment of Moderate to Severe Croup. Journal of the Korean Pediatric Society [Internet]. 1999;40–6. Available from: http://dx.doi.org/

29. Jeong-Ryul JO, Young-Ho JIN, JEONG TO, Jae-Bak LEE. The Clinical Impact of the Pneumonia Severity Index and the CURB-65 for Making Admission Decisions. Journal of the Korean Society of Emergency Medicine [Internet]. 2009;122–9. Available from: http://dx.doi.org/

30. PARK SH, Byung-Moon CHO, Sae-Moon OH. Head Injuries from Falls in Preschool Children. Yonsei Medical Journal [Internet]. 2004;229–32. Available from: http://dx.doi.org/10.3349/ymj.2004.45.2.229

31. Hwang SY, Jo IJ, Lee SU, Lee TR, Yoon H, Cha WC, et al. Low Accuracy of Positive qSOFA Criteria for Predicting 28-Day Mortality in Critically Ill Septic Patients During the Early Period After Emergency Department Presentation. Annals of emergency medicine. 2018;71(1):1-9.e2.

32. Su L, Tang B, Liu Y, Zhou G, Guo Q, He W, et al. P(v-a)CO2/C(a-v)O2-directed resuscitation does not improve prognosis compared with SvO2 in severe sepsis and septic shock: A prospective multicenter randomized controlled clinical study. Journal of critical care. 2018;48(buy, 8610642):314–20.

33. Smud A, Nagel CB, Madsen E, Rial M del C, Barcan LA, Gomez AA, et al. Pandemic influenza A/H1N1 virus infection in solid organ transplant recipients: a multicenter study. Transplantation. 2010;90(12):1458–62.

34. Estenssoro E, Dubin A, Laffaire E, Canales H, Saenz G, Moseinco M, et al. Incidence, clinical course, and outcome in 217 patients with acute respiratory distress syndrome. Critical Care Medicine. 2002;30(11):2450–6.

35. Teo J. Early detection of silent hypoxia in Covid-19 pneumonia using smartphone pulse oximetry. Journal of medical systems. 2020;44(8):1–2.

36. Ang BT, Chan SP, Lee KK, Ng I. Prediction of early mortality in primary intracerebral hemorrhage in an Asian population. Acta neurochirurgica Supplement. 2008;102(100962752):299–303.

37. Lagarto L, Cerejeira J. Identification of sub-groups in acutely ill elderly patients with delirium: A cluster analysis. International Psychogeriatrics [Internet]. 2016;28(8):1283–92. Available from: http://journals.cambridge.org/action/displayJournal?jid=IPG

38. Ali Z. Acute respiratory disorders in the newborn at the Mount Hope Women's Hospital, Trinidad. West Indian Medical Journal. 2003;52(1):23–6.

39. Ross PA, Newth CJL, Khemani RG. Accuracy of pulse oximetry in children. Pediatrics [Internet]. 2014;133(1):22–9. Available from: https://pediatrics.aappublications.org/content/pediatrics/133/1/22.full.pdf

40. Mower WR, Myers G, Nicklin EL, Kearin KT, Baraff LJ, Sachs C. Pulse oximetry as a fifth vital sign in emergency geriatric assessment. Academic emergency medicine [Internet]. 1998;5(9):858–65. Available from: https://onlinelibrary.wiley.com/doi/pdfdirect/10.1111/j.1553-2712.1998.tb02813.x?download=true

41. Young BE, Fong SW, Chan YH, Mak TM, Ang LW, Anderson DE, et al. Effects of a major deletion in the SARS-CoV-2 genome on the severity of infection and the inflammatory response: an observational cohort study. Lancet (London, England). 2020;396(10251):603–11.

42. Choi J, Claudius I. Decrease in emergency department length of stay as a result of triage pulse oximetry. Pediatric emergency care. 2006;22(6):412–4.

43. Sulter G, Elting JW, Stewart R, den Arend A, de Keyser J. Continuous pulse oximetry in acute hemiparetic stroke. Journal of the neurological sciences. 2000;179(1–2):65–9.

44. Chiu CY, Wong KS, Yao TC, Huang JL. Asthmatic versus non-asthmatic spontaneous pneumomediastinum in children. Asian Pac J Allergy Immunol [Internet]. 2005; Available from: http://imsear.searo.who.int/handle/123456789/36842

45. YOON Y, Won-Sup KIM, SHIN JS, JEONG EH, YANG H, CHOI KG, et al. Predictive Factors of Neurologic Outcome in Patients With Hypoxic-Ischemic Encephalopathy After Cardiopulmonary Resuscitation. Journal of the Korean Neurological Association [Internet]. 2010;192–202. Available from: http://dx.doi.org/

46. Kuchar E, Miskiewicz K, Szenborn L, Nitsch-Osuch A. Respiratory complications in children hospitalized with varicella. Neurobiology of RespirationAdvances in Experimental Medicine and Biology. 2013;788((Kuchar, Miskiewicz, Szenborn) Department of Pediatric Infectious Diseases, Wroclaw Medical University, 44 Bujwida St., 50-345 Wroclaw, Poland(Nitsch-Osuch) Department of Family Medicine, Warsaw Medical University, Warsaw, Poland):97–102.

47. Kwok AC, Funk LM, Baltaga R, Lipsitz SR, Merry AF, Dziekan G, et al. Implementation of the World Health Organization Surgical Safety Checklist and pulse oximetry in a resource-limited setting. Journal of the American College of Surgeons. 2011;213(3 SUPPL. 1):S113-.

48. Gendrel D. Mycoplasma pneumoniae and acute respiratory tract infections. Revue Francaise d'Allergologie et d'Immunologie Clinique. 2007;47(7):442–5.

49. Chi JH, Knudson MM, Vassar MJ, McCarthy MC, Shapiro MB, Mallet S, et al. Prehospital hypoxia affects outcome in patients with traumatic brain injury: A prospective multicenter study. Journal of Trauma - Injury, Infection and Critical Care. 2006;61(5):1134–41.

50. Jae-Hui KIM, Min-Ji GOO, YEOM JS, PARK ES, Ji-Hyun SEO, Jae-Young LIM, et al. Clinical characteristics of acute renal failure of rhabdomyolysis in children. Korean Journal of Pediatrics [Internet]. 2007;277–83. Available from: http://dx.doi.org/10.3345/kjp.2007.50.3.277

51. YANG SI, Jung-Hee RHO, Yong-Han SUN, Kang-Ho CHO, SHIM SY, Byung-Wook EUN, et al. The Comparison of Clinical Characteristics and Courses of Pediatric Patients Hospitalized with Pandemic Influenza A (H1N1) and Seasonal Influenza from 2009 to 2011. Pediatric Allergy and Respiratory Disease [Internet]. 2012;292–301. Available from: http://dx.doi.org/10.7581/pard.2012.22.3.292

52. Won-Sup OH, Seung-Joon LEE, Chang-Seop LEE, Ji-An HUR, Ae-Chung HUR, PARK YS, et al. A Prediction Rule to Identify Severe Cases among Adult Patients Hospitalized with Pandemic Influenza A (H1N1) 2009. Journal of Korean Medical Science [Internet]. 2011;499–506. Available from: http://dx.doi.org/10.3346/jkms.2011.26.4.499

53. Becattini C, Vedovati MC, Pruszczyk P, Vanni S, Cotugno M, Cimini LA, et al. Oxygen saturation or respiratory rate to improve risk stratification in hemodynamically stable patients with acute pulmonary embolism. Journal of thrombosis and haemostasis : JTH [Internet]. 2018;16(12):2397–402. Available from: https://onlinelibrary.wiley.com/doi/pdfdirect/10.1111/jth.14299?download=true

54. Zou X, Li S, Fang M, Hu M, Bian Y, Ling J, et al. Acute Physiology and Chronic Health Evaluation II Score as a Predictor of Hospital Mortality in Patients of Coronavirus Disease 2019. Critical care medicine [Internet]. 2020;48(8):e657–65. Available from: https://www.ncbi.nlm.nih.gov/pmc/articles/PMC7217128/pdf/ccm-48-e657.pdf

55. Sankar J, Singh A, Sankar MJ, Joghee S, Dewangan S, Dubey N. Pediatric Index of Mortality and PIM2 scores have good calibration in a large cohort of children from a developing country. BioMed research international [Internet]. 2014;2014(101600173):907871. Available from: https://downloads.hindawi.com/journals/bmri/2014/907871.pdf

56. Talmor D, Jones AE, Rubinson L, Howell MD, Shapiro NI. Simple triage scoring system predicting death and the need for critical care resources for use during epidemics. Critical care medicine. 2007;35(5):1251–6.

57. Hukkelhoven CWPM, Steyerberg EW, Habbema JDF, Farace E, Marmarou A, Murray GD, et al. Predicting outcome after traumatic brain injury: development and validation of a prognostic score based on admission characteristics. Journal of neurotrauma [Internet]. 2005;22(10):1025–39. Available from: https://www.liebertpub.com/doi/10.1089/neu.2005.22.1025?url_ver=Z39.88-2003&rfr_id=ori:rid:crossref.org&rfr_dat=cr_pub%3dpubmed

58. Chisti MJ, Shahunja KM, Afroze F, A.S.M.S.B. S, Sharifuzzaman, Ahmed T. Hypoxaemia and septic shock were independent risk factors for mechanical ventilation in Bangladeshi children hospitalised for diarrhoea. Acta Paediatrica, International Journal of Paediatrics [Internet]. 2017;106(7):1159–64. Available from: http://onlinelibrary.wiley.com/journal/10.1111/(ISSN)1651-2227/issues

59. Vespa PM, Bleck TP. Neurogenic pulmonary edema and other mechanisms of impaired oxygenation after aneurysmal subarachnoid hemorrhage. Neurocritical care [Internet]. 2004;1(2):157–70. Available from: https://link.springer.com/content/pdf/10.1385/NCC:1:2:157.pdf

60. Nakatsuka Y, Shiba M, Nishikawa H, Terashima M, Kawakita F, Fujimoto M, et al. Acute-Phase Plasma Osteopontin as an Independent Predictor for Poor Outcome After Aneurysmal Subarachnoid Hemorrhage. Molecular neurobiology [Internet]. 2018;55(8):6841–9. Available from: https://link.springer.com/content/pdf/10.1007/s12035-018-0893-3.pdf

61. Ameenudeen SAKM, Boo NY, Chan LG. Risk factors associated with chronic lung disease in Malaysian very low birthweight infants. The Medical journal of Malaysia. 2007;62(1):40–5.

62. Braun E, Domany E, Kenig Y, Mazor Y, Makhoul BF, Azzam ZS. Elevated red cell distribution width predicts poor outcome in young patients with community acquired pneumonia. Critical care (London, England) [Internet]. 2011;15(4):R194-. Available from: https://ccforum.biomedcentral.com/track/pdf/10.1186/cc10355.pdf

63. Luna CM, Palma I, Niederman MS, Membriani E, Giovini V, Wiemken TL, et al. The impact of age and comorbidities on the mortality of patients of different age groups admitted with community-acquired pneumonia. Annals of the American Thoracic Society [Internet]. 2016;13(9):1519–26. Available from: http://www.atsjournals.org/doi/pdf/10.1513/AnnalsATS.201512-848OC

64. Bertsimas D, Lukin G, Mingardi L, Nohadani O, Orfanoudaki A, Stellato B, et al. COVID-19 mortality risk assessment: An international multi-center study. PloS one. 2020;15(12):e0243262-.

65. Gundogan K, Akbudak IH, Hanci P, Halacli B, Temel S, Gullu Z, et al. Clinical outcomes and independent risk factors for 90-day mortality in critically ill patients with respiratory failure infected with sars-cov-2: A multicenter study in turkish intensive care units. Balkan Medical Journal [Internet]. 2021;38(5):296–303. Available from: https://balkanmedicaljournal.org/Content/files/sayilar/214/296-303.pdf

66. Apiliogullari B, Sunam GS, Ceran S, Koc H. Evaluation of neonatal pneumothorax. The Journal of international medical research [Internet]. 2011;39(6):2436–40. Available from: https://journals.sagepub.com/doi/pdf/10.1177/147323001103900645

67. Pervaiz F, Hossen S, Chavez MA, Miele CH, Moulton LH, McCollum ED, et al. Training and standardization of general practitioners in the use of lung ultrasound for the diagnosis of pediatric pneumonia. Pediatric Pulmonology [Internet]. 2019;54(11):1753–9. Available from: http://onlinelibrary.wiley.com/journal/10.1002/(ISSN)1099-0496

68. Li J, Zhao Y, Liu Z, Zhang T, Liu C, Liu X. Clinical report of serious complications associated with measles pneumonia in children hospitalized at Shengjing hospital, China. Journal of Infection in Developing Countries [Internet]. 2015;9(10):1139–46. Available from: http://www.jidc.org/index.php/journal/article/download/6534/1402

69. Tylicki L, Puchalska-Reglinska E, Tylicki P, Och A, Polewska K, Biedunkiewicz B, et al. Predictors of Mortality in Hemodialyzed Patients after SARS-CoV-2 Infection. Journal of Clinical Medicine [Internet]. 2022;11(2):285. Available from: https://www.mdpi.com/2077-0383/11/2/285/pdf

70. Valik JK, Mellhammar L, Sunden-Cullberg J, Ward L, Unge C, Dalianis H, et al. Peripheral Oxygen Saturation Facilitates Assessment of Respiratory Dysfunction in the Sequential Organ Failure Assessment Score With Implications for the Sepsis-3 Criteria. Critical care medicine. 2022;50(3):e272–83.

71. Potter VAJ. Pulse oximetry in general practice: how would a pulse oximeter influence patient management? The European journal of general practice [Internet]. 2007;13(4):216–20. Available from: https://www.tandfonline.com/doi/pdf/10.1080/13814780701574762?needAccess=true

72. Li L, Zhang W, Hu Y, Tong X, Zheng S, Yang J, et al. Effect of Convalescent Plasma Therapy on Time to Clinical Improvement in Patients With Severe and Life-threatening COVID-19: A Randomized Clinical Trial. JAMA [Internet]. 2020;324(5):460–70. Available from: https://jamanetwork.com/journals/jama/articlepdf/2766943/jama_li_2020_oi_200065.pdf

73. Lozano J. Epidemiology of hypoxaemia in children with acute lower respiratory infection [oxygen therapy in children]. The international journal of tuberculosis and lung disease [Internet]. 2001;5(6):496–504. Available from: http://docserver.ingentaconnect.com/deliver/connect/iuatld/10273719/v5n6/s2.pdf?expires=1618451899&id=0000&titleid=3764&checksum=16F873E5D14F13F65FC970AE587CAF9E

74. Yan-xiang ZHU, Jie YAO, Shang-kun LU, ZHANG G sheng, ZHOU G ren. Study on changes of partial pressure of brain tissue oxygen and brain temperature in acute phase of severe head injury during mild hypothermia therapy. Chinese Journal of Traumatology [Internet]. 2003;(6):152–5. Available from: http://dx.doi.org/

75. Zhang P-J, Li X-L, Cao B, Yang S-G, Liang L-R, Gu L, et al. Clinical features and risk factors for severe and critical pregnant women with 2009 pandemic H1N1 influenza infection in China. BMC Infectious Diseases. 2012;29.

76. CHANG JH, SHIN TR, Ga-Eun WOO, Jong-Seon KIM, HONG ES, Gi-Yeoul SEO, et al. Changes in Plasma and Urine Endothelin Levels During Acute Exacerbation of Asthma. Tuberculosis and Respiratory Diseases [Internet]. 1997;844–52. Available from: http://dx.doi.org/10.4046/trd.1997.44.4.844

77. Heljic S, Maksic H, Kalkan I, Krdalic B. The effects of antenatal corticosteroids and surfactant replacement on neonatal respiratory distress syndrome. Bosnian journal of basic medical sciences / Udruzenje basicnih mediciniskih znanosti = Association of Basic Medical Sciences. 2009;9(3):225–8.

78. Eldaboosy SAM, Halima KM, Shaarawy AT, Kanany HM, Elgamal EM, A.-A. EG, et al. Comparison between CURB-65, PSI, and SIPF scores as predictors of ICU admission and mortality in community-acquired pneumonia. Egyptian Journal of Critical Care Medicine [Internet]. 2015;3(2–3):37–44. Available from: http://www.elsevier.com/journals/egyptian-journal-of-critical-care-medicine/2090-7303

79. Elshamly M, Nour MO, Omar AMM. Clinical presentations and outcome of severe community-acquired pneumonia. Egyptian Journal of Chest Diseases and Tuberculosis. 2016;65(4):831–9.

80. Weber MW, Dackour R, Usen S, Schneider G, Adegbola RA, Cane P, et al. The clinical spectrum of respiratory syncytial virus disease in The Gambia. Pediatric Infectious Disease Journal. 1998;17(3):224–30.

81. Muhe L, Tilahun M, Lulseged S, Kebede S, Enaro D, Ringertz S, et al. Etiology of pneumonia, sepsis and meningitis in infants younger than three months of age in Ethiopia. Pediatric Infectious Disease Journal. 1999;18(10 SUPPL.):S56–61.

82. Smyth A, Carty H, Hart CA. Clinical predictors of hypoxaemia in children with pneumonia. Annals of Tropical Paediatrics. 1998;18(1):31–40.

83. Paul VK, Singh M, Sundaram KR, Deorari AK. Correlates of mortality among hospital-born neonates with birth asphyxia. 1997; Available from: http://imsear.searo.who.int/handle/123456789/118249

84. Sritippayawan S, Deerojanawong J, Prapphal N. Clinical score and arterial oxygen saturation in children with wheezing associated respiratory illness (WARI). 2000; Available from: http://imsear.searo.who.int/handle/123456789/40505

85. Fawzi WW, Mbise RL, Fataki MR, Herrera MG, Kawau F, Hertzmark E, et al. Vitamin A supplementation and severity of pneumonia in children admitted to the hospital in Dar es Salaam, Tanzania. The American journal of clinical nutrition [Internet]. 1998;68(1):187–92. Available from: https://watermark.silverchair.com/187.pdf?token=AQECAHi208BE49Ooan9kkhW_Ercy7Dm3ZL_9Cf3qfKAc485ysgAAApswggKXBgkqhkiG9w0BBwagggKIMIIChAIBADCCAn0GCSqGSIb3DQEHATAeBglghkgBZQMEAS4wEQQM9O2CXVgrw2uDUpVtAgEQgIICTgWtd22u0LWvcGHgfyVBPDOl-rkUiOgd93lCqN-mMGDxoZCk8mo

86. McHugh GS, Engel DC, Butcher I, Steyerberg EW, Lu J, Mushkudiani N, et al. Prognostic value of secondary insults in traumatic brain injury: results from the IMPACT study. Journal of neurotrauma [Internet]. 2007;24(2):287–93. Available from: https://www.liebertpub.com/doi/pdfplus/10.1089/neu.2006.0031

87. Kanburoglu MK, Tayman C, Oncel MY, Akin IM, Can E, Demir N, et al. A Multicentered Study on Epidemiologic and Clinical Characteristics of 37 Neonates with Community-acquired COVID-19. Pediatric Infectious Disease Journal [Internet]. 2020;((Kanburoglu) Recep Tayyip Erdogan University School of Medicine, Department of Pediatrics, Division of Neonatology, Rize, Turkey(Tayman) Ministry of Health, Ankara City Hospital, Division of Neonatology, Ankara, Turkey(Oncel) Izmir Katip Celebi University):E297–302. Available from: http://journals.lww.com/pidj

88. Kaya S, Yilmaz G, Arslan M, Oztuna F, Ozlu T, Koksal I. Predictive factors for fatality in pandemic influenza A (H1N1) virus infected patients. Saudi medical journal. 2012;33(2):146–51.

89. Devoe NC, Kyriazis P, Eltanbedawi A, Contractor A, Esposito AW, Khan MS, et al. An audit of oxygen supplementation in a large tertiary hospital - we should treat oxygen as any other drug. Hosp Pract (1995). 2021;49(2):100–3.

90. Dartois N, Cooper CA, Castaing N, Gandjini H, Sarkozy D. Tigecycline versus levofloxacin in hospitalized patients with community-acquired pneumonia: An analysis of risk factors. Open Respiratory Medicine Journal [Internet]. 2013;7(1):13–20. Available from: https://openrespiratorymedicinejournal.com/contents/volumes/V7/TORMJ-7-13/TORMJ-7-13.pdf

Supplemental material #2

PAPER: The prevalence of hypoxaemia among paediatric and adult patients in healthcare facilities in low- and middle-income countries: systematic review and meta-analysis

AUTHORS: Hamish R Graham, Rami Subhi, Esrat Jahan, Farhia Azrin, Jaclyn Maher, Jasmine Miller, Ahmed Ehsanur Rahman, Felix Lam.

Results Tables and Forest Plots

Primary Results tables and Forest plots (Admitted)

Admitted Neonates - all

Table 1: Studies included in prevalence of hypoxaemia among all admitted neonate

Serial No	Study ID	WB Region	Proportion	LCL	UCL	Weight	Denominator
1	Duke 2002	East Asia & Pacific	43.18	34.59	52.08	8.58	132
2	English 2003	Sub Saharan Africa	19.97	16.88	23.35	10.29	616
3	Junge 2006	Sub Saharan Africa	16.45	12.50	21.06	9.76	310
4	Mwaniki 2009	Sub Saharan Africa	18.64	16.39	21.07	10.54	1105
5	Orimadegun 2013	Sub Saharan Africa	41.41	36.84	46.09	10.09	454
6	McCollum 2013	Sub Saharan Africa	20.59	8.70	37.90	5.37	34
7	Morgan 2018	Sub Saharan Africa	29.24	24.86	33.92	10.01	407
8	Graham 2019	Sub Saharan Africa	18.24	17.37	19.13	10.82	7473
9	King 2022	Sub Saharan Africa	39.29	21.50	59.42	4.85	28
10	Kiputa 2022	Sub Saharan Africa	21.26	17.08	25.94	9.87	348
11	Graham 2022	Sub Saharan Africa	16.26	12.42	20.72	9.81	326
**Overall (I2=94.49%, T2=0.03, p<0.05)**			**24.49**	**19.92**	**29.35**		**11233**

Fig.1: Studies included in prevalence of hypoxaemia among all admitted neonates

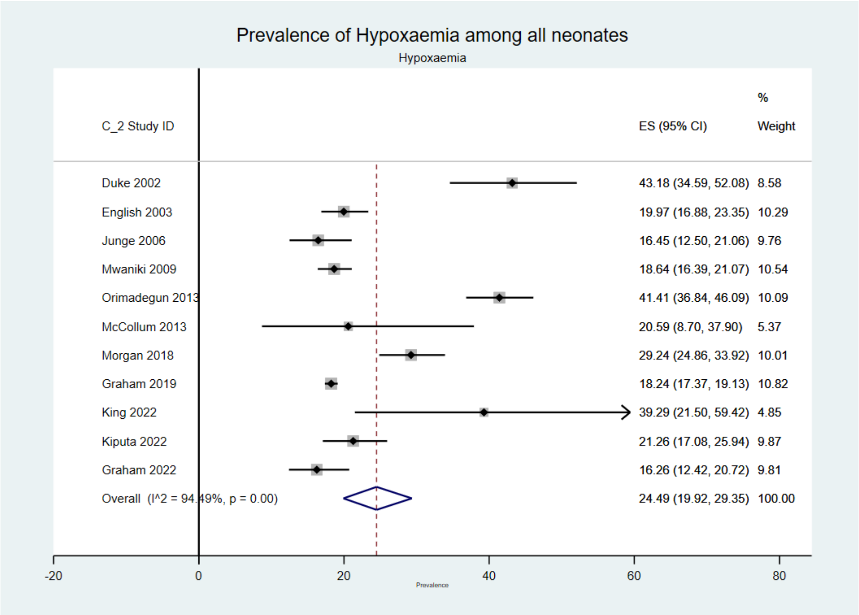

The evidence was assessed as **moderate certainty** because of inconsistency in prevalence estimates between studies.

Admitted Neonates – neonatal encephalopathy / birth asphyxia

Table 2 : Studies included in prevalence of hypoxaemia among admitted neonates with birth asphyxia

Serial No	Study ID	WB Region	Proportion	LCL	UCL	Weight	Denominator
1	Duke 2002	East Asia & Pacific	60.00	38.67	78.87	27.28	25
2	Ondoa-Onama 2003	Sub Saharan Africa	14.68	8.63	22.74	34.84	109
3	Graham 2019	Sub Saharan Africa	33.40	31.54	35.30	37.88	2458
**Overall (I2=92.78%, T2=0.10, p<0.05)**			**32.79**	**16.23**	**51.83**		**2592**

Fig. 2: Studies included in prevalence of hypoxaemia among admitted neonates with birth asphyxia

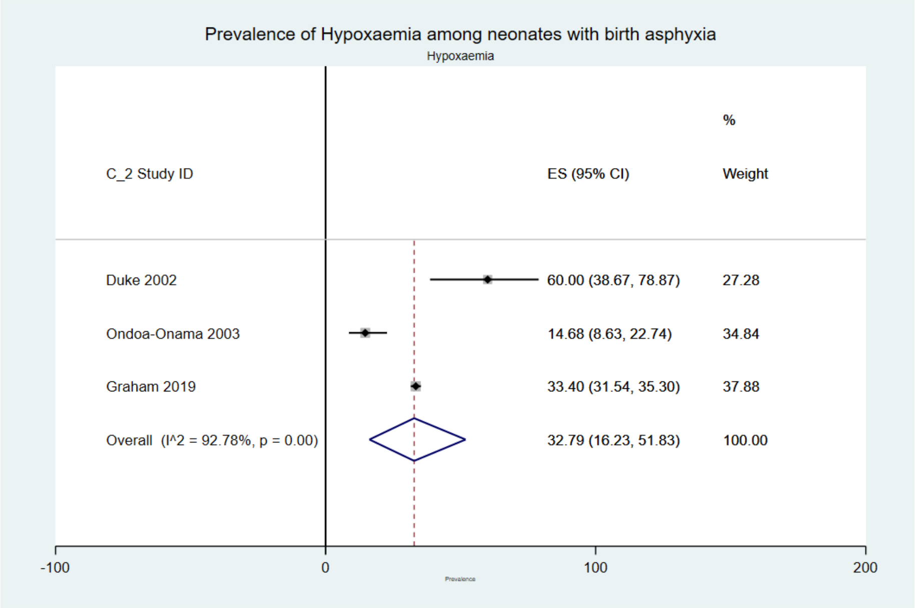

The evidence was assessed as **very low certainty** because of moderate risk of bias (variable validity of diagnostic assessment), imprecision of the estimate, inconsistency in prevalence estimates between studies.

Admitted Neonates - pneumonia

Table 3: Studies included in prevalence of hypoxaemia among admitted neonates with pneumonia

Serial No	Study ID	WB Region	Proportion	LCL	UCL	Weight	Denominator
1	Duke 2002	East Asia & Pacific	77.27	54.63	92.18	31.59	22
2	Dembele 2019	East Asia & Pacific	11.88	9.65	14.42	35.04	749
3	LeRoux 2021	Sub Saharan Africa	31.91	19.09	47.12	33.37	47
**Overall (I2=96.25%, T2=0.39, p<0.05)**			**37.33**	**7.60**	**73.50**		**818**

Fig. 3: Studies included in prevalence of hypoxaemia among admitted neonates with pneumonia

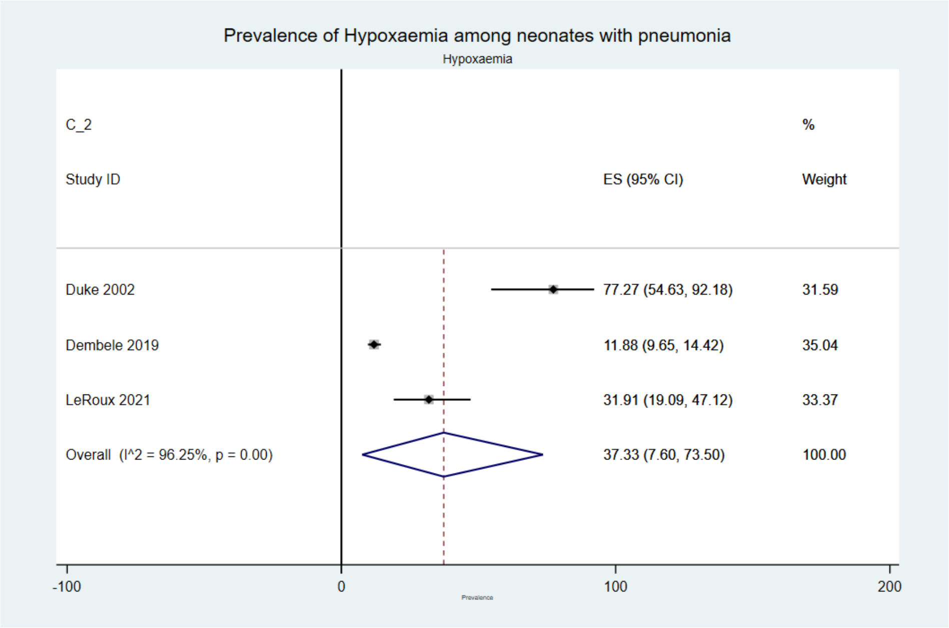

The evidence was assessed as **very low certainty** because of moderate risk of bias (variable representativeness of population / variable validity of diagnostic assessment), imprecision of the estimate, inconsistency in prevalence estimates between studies.

Admitted Neonates - prematurity

Table 4: Studies included in Prevalence of hypoxaemia among admitted neonate with prematurity

Serial no	Study ID	WB Region	Proportion	LCL	UCL	Weight	Denominator
1	Duke 2002	East Asia & Pacific	30.30	15.59	48.71	27.66	33
2	Morgan 2018	Sub Saharan Africa	48.28	37.42	59.25	33.70	87
3	Graham 2019	Sub Saharan Africa	25.80	23.60	28.09	38.64	1500
**Overall (I2=89.23%, T2=0.07, p<0.05)**			**34.29**	**19.50**	**50.77**		**1620**

Fig. 4: Studies included in Prevalence of hypoxaemia among admitted neonate with prematurity

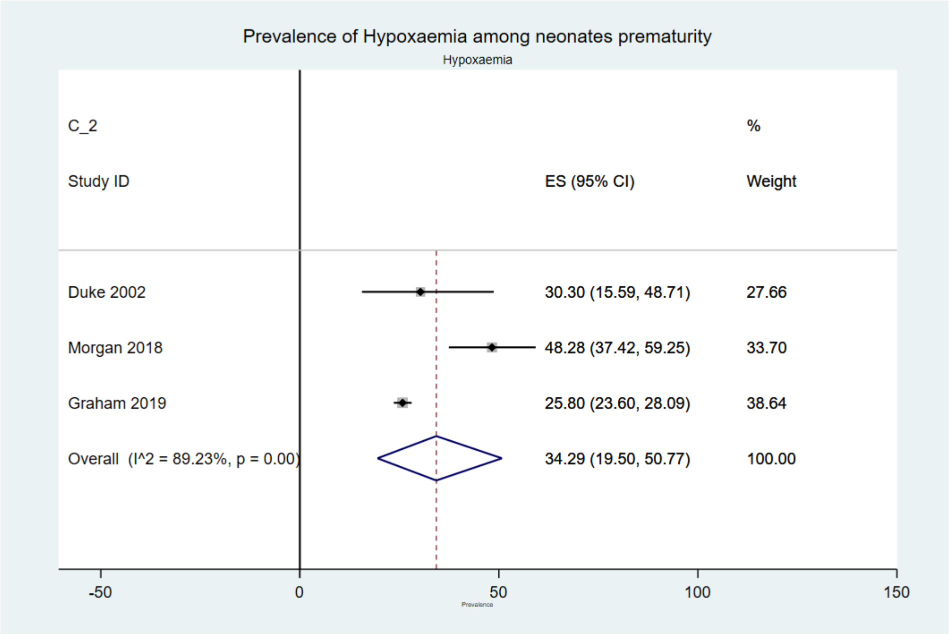

The evidence was assessed as **low certainty** because of imprecision of the estimate, inconsistency in prevalence estimates between studies.

Admitted Neonates - sepsis

Table 5: Studies included in prevalence of hypoxaemia among admitted neonates with sepsis

Serial No	Study ID	WB Region	Proportion	LCL	UCL	Weight	Denominator
1	Duke 2002	East Asia & Pacific	44.12	27.19	62.11	1.07	34
2	Graham 2019	Sub Saharan Africa	21.00	19.60	22.46	98.93	3195

Fig. 5: Studies included in prevalence of hypoxaemia among admitted neonates with sepsis

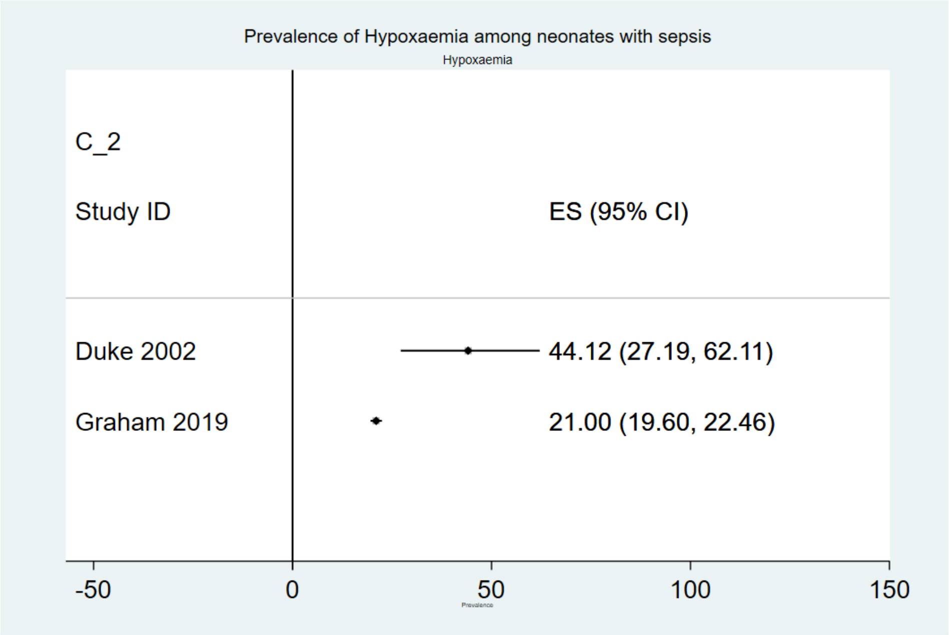

No meta-estimate calculated as only two included studies.

Admitted Children - all

Table 6: Studies included in prevalence of hypoxaemia among all admitted children

Serial No	Study ID	WB Region	Proportion	LCL	UCL	Weight	Denominator
1	Duke 2002	East Asia & Pacific	55.71	50.40	60.92	4.99	359
2	English 2003	Sub Saharan Africa	14.04	10.27	18.56	4.86	292
3	Djelantik 2003	East Asia & Pacific	11.19	10.27	12.17	5.58	4306
4	Wandi 2006	East Asia & Pacific	24.16	22.24	26.15	5.50	1896
5	Junge 2006	Sub Saharan Africa	5.75	4.98	6.60	5.56	3269
6	Mwaniki 2009	Sub Saharan Africa	5.26	4.88	5.65	5.62	13183
7	Foran 2010	Sub Saharan Africa	6.02	1.98	13.50	3.60	83
8	Orimadegun 2013	Sub Saharan Africa	24.06	21.73	26.50	5.44	1272
9	McCollum 2013	Sub Saharan Africa	4.54	3.14	6.32	5.30	727
10	Lowlaavar 2016	Sub Saharan Africa	25.72	23.35	28.19	5.44	1291
11	Barennes 2016	East Asia & Pacific	13.71	10.29	17.77	4.97	350
12	Nielsen 2018	Latin America & Caribbean	8.58	6.91	10.50	5.38	991
13	Hau 2018	Sub Saharan Africa	5.59	3.80	7.88	5.18	537
14	Graham 2019	Sub Saharan Africa	7.93	7.52	8.35	5.62	16453
15	Enoch 2019	Sub Saharan Africa	10.00	9.51	10.50	5.62	14232
16	Tuti 2021	Sub Saharan Africa	6.97	6.77	7.17	5.64	64722
17	Krithika 2022	South Asia	8.17	6.10	10.65	5.23	600
18	King 2022	Sub Saharan Africa	7.69	5.92	9.79	5.32	780
19	Graham 2022	Sub Saharan Africa	11.26	8.64	14.35	5.16	506
**Overall (I2=99.03%, T2=0.02, p<0.05)**			**12.13**	**9.99**	**14.43**		**125849**

Fig. 6: Studies included in prevalence of hypoxaemia among all admitted children

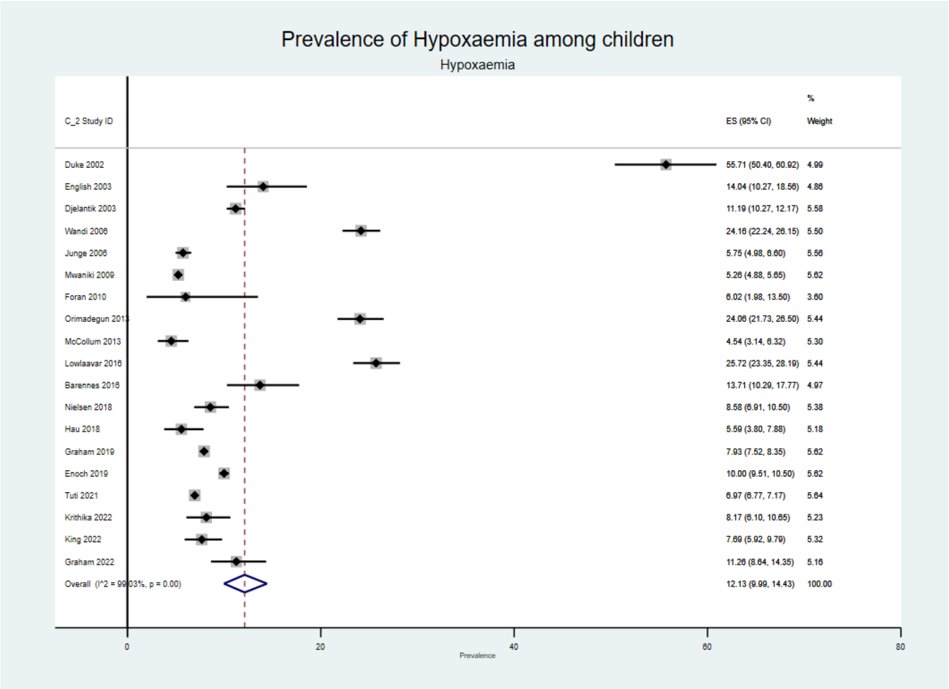

The evidence was assessed as **moderate certainty** because of inconsistency in prevalence estimates between studies.

Admitted Children - fever

Table 7: Studies included in prevalence of hypoxaemia among admitted children with fever

Serial No	Study ID	WB Region	Proportion	LCL	UCL	Weight	Denominator
1	Usen 1999	Sub Saharan Africa	14.86	7.66	25.04	32.13	74
2	Bassat 2016	Sub Saharan Africa	27.75	24.56	31.11	33.86	746
3	Leligdowicz 2021	Sub Saharan Africa	4.13	3.38	4.99	34.00	2469
**Overall (I2=99.30%, T2=0.22, p<0.05)**			**14.06**	**1.12**	**37.26**		**3289**

Fig. 7: Studies included in prevalence of hypoxaemia among admitted children with fever

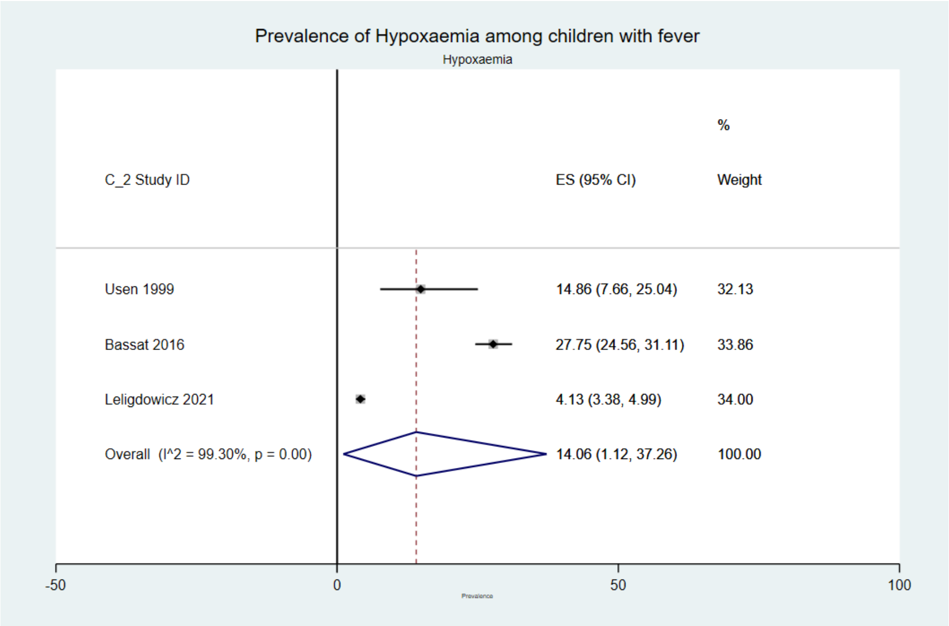

The evidence was assessed as **very low certainty** because of moderate risk of bias (variable representativeness of population), imprecision of the estimate, inconsistency in prevalence estimates between studies.

Admitted Children – pneumonia all WHO-classification

Table 8: Studies included in prevalence of hypoxaemia among all admitted children with pneumonia (WHO-Classification)

Sl no	Study ID	WB Region	Proportion	LCL	UCL	Weight	Denominator
1.	Usen 1999	Sub Saharan Africa	5.88	4.55	7.46	1.54	1072
2.	West 1999	Sub Saharan Africa	43.68	36.52	51.05	1.50	190
3.	Duke 2002	East Asia & Pacific	72.65	66.29	78.38	1.51	223
4.	Djelantik 2003	East Asia & Pacific	11.19	10.27	12.17	1.55	4306
5.	Addo-Yobo 2004	Mixed	19.10	17.25	21.04	1.55	1702
6.	Laman 2005	East Asia & Pacific	25.97	16.64	37.23	1.42	77
7.	Wandi 2006	East Asia & Pacific	54.50	50.34	58.61	1.53	578
8.	Fu 2006	Mixed	19.30	17.45	21.26	1.55	1694
9.	Junge 2006	Sub Saharan Africa	11.70	8.83	15.09	1.53	436
10	Bruce 2007	Latin America & Caribbean	51.71	45.49	57.89	1.51	263
11	Puumalainen 2008	East Asia & Pacific	16.16	14.08	18.42	1.54	1151
12	Ashraf 2008	South Asia	56.97	50.60	63.18	1.51	251
13	Duke 2008	East Asia & Pacific	57.09	52.94	61.17	1.53	578
14	Asghar 2008	Mixed	64.61	61.49	67.65	1.54	958
15	Nantanda 2008	Sub Saharan Africa	39.49	31.79	47.59	1.49	157
16	Mwaniki 2009	Sub Saharan Africa	8.40	7.68	9.16	1.55	5489
17	Sigauque 2009	Sub Saharan Africa	25.84	22.60	29.29	1.54	685
18	Martinez-Medina 2010	Latin America & Caribbean	56.45	43.26	69.01	1.40	62
19	Ashraf 2010	South Asia	52.50	47.20	57.76	1.52	360
20	Chisti 2011	South Asia	54.55	47.33	61.62	1.50	198
21	Webb 2012	Sub Saharan Africa	23.59	20.16	27.30	1.53	568
22	Izadnegahdar 2012	Mixed	15.50	13.66	17.47	1.54	1439
23	Muller 2012	Latin America & Caribbean	22.48	20.66	24.38	1.55	1993
24	Ramakrishna 2012	Sub Saharan Africa	37.34	31.11	43.89	1.51	233
25	Srinivasan 2012	Sub Saharan Africa	28.98	24.29	34.02	1.52	352

26	Jain 2013	South Asia	17.68	12.42	24.03	1.49	181
27	Kuti 2013	Sub Saharan Africa	19.76	16.01	23.94	1.53	410
28	McCollum 2013	Sub Saharan Africa	17.22	11.57	24.20	1.48	151
29	Orimadegun 2013	Sub Saharan Africa	49.20	43.53	54.88	1.52	313
30	Kuti 2013	Sub Saharan Africa	19.29	15.62	23.39	1.53	420
31	Sempertegui 2014	Latin America & Caribbean	85.11	81.48	88.27	1.53	450
32	Ibraheem 2014	Sub Saharan Africa	41.50	34.59	48.66	1.50	200
33	Abdulkadir 2015	Sub Saharan Africa	41.50	34.59	48.66	1.50	200
34	Breiman 2015	Sub Saharan Africa	35.74	33.88	37.63	1.55	2563
35	Kelly 2015	Sub Saharan Africa	38.66	32.43	45.16	1.51	238
36	Basnet 2015	South Asia	61.15	57.15	65.04	1.53	610
37	Salah 2015	Sub Saharan Africa	42.67	34.64	50.99	1.48	150
38	Tran 2016	East Asia & Pacific	8.50	6.91	10.33	1.54	1082
39	Bassat 2016	Sub Saharan Africa	27.88	24.84	31.07	1.54	825
40	Nemani 2016	South Asia	40.00	31.67	48.78	1.48	135
41	Alwadhi 2017	South Asia	50.89	41.27	60.46	1.46	112
42	Graham 2019	Sub Saharan Africa	23.44	21.64	25.33	1.55	2073
43	McCollum 2019	Sub Saharan Africa	64.44	60.61	68.14	1.54	644
44	Laghari 2019	South Asia	34.00	24.82	44.15	1.45	100
45	Ashraf 2019	South Asia	10.64	8.00	13.78	1.53	470
46	Dembele 2019	East Asia & Pacific	13.52	12.50	14.59	1.55	4179
47	Ma 2019	Sub Saharan Africa	85.81	79.30	90.89	1.49	155
48	PERCHStudyGroup 2019	Mixed	35.74	34.25	37.26	1.55	3981
49	Fashanu 2020	Sub Saharan Africa	58.38	55.84	60.90	1.54	1497
50	Hooli 2020	Sub Saharan Africa	14.69	12.93	16.59	1.54	1491
51	Fagbohun 2020	Latin America & Caribbean	13.02	10.85	15.46	1.54	860
52	Shahrin 2020	South Asia	15.18	10.41	21.07	1.50	191
53	Muro 2020	Sub Saharan Africa	32.40	26.64	38.58	1.51	250

54	Oktaria 2021	East Asia & Pacific	13.53	8.22	20.54	1.48	133
55	Bui-Binh-Bao 2021	East Asia & Pacific	11.39	7.92	15.69	1.51	281
56	Chisti 2021	South Asia	28.13	26.74	29.55	1.55	4007
57	Rahman 2021	South Asia	39.98	38.11	41.88	1.55	2646
58	LeRoux 2021	Sub Saharan Africa	32.28	24.26	41.15	1.47	127
59	Ahmed 2022	Sub Saharan Africa	75.32	64.18	84.44	1.42	77
60	Awasthi 2022	South Asia	35.85	34.74	36.97	1.55	7196
61	Saleh 2022	Middle East & North Africa	26.67	20.36	33.76	1.49	180
62	Chisti 2022	South Asia	31.23	29.69	32.80	1.55	3468
63	Jullien 2022	South Asia	75.51	67.74	82.22	1.48	147
64	Kapoor 2022	South Asia	36.11	29.10	43.59	1.49	180
65	Mvalo 2022	Sub Saharan Africa	63.75	59.53	67.82	1.53	538
66	Zar 2022	Sub Saharan Africa	24.38	18.61	30.92	1.50	201
Overall (I2=99.31%, T2=0.14, p<0.05)			34.57	30.22	39.05		68127

Fig. 8: Studies included in prevalence of hypoxaemia among all admitted children with pneumonia (WHO-Classification)

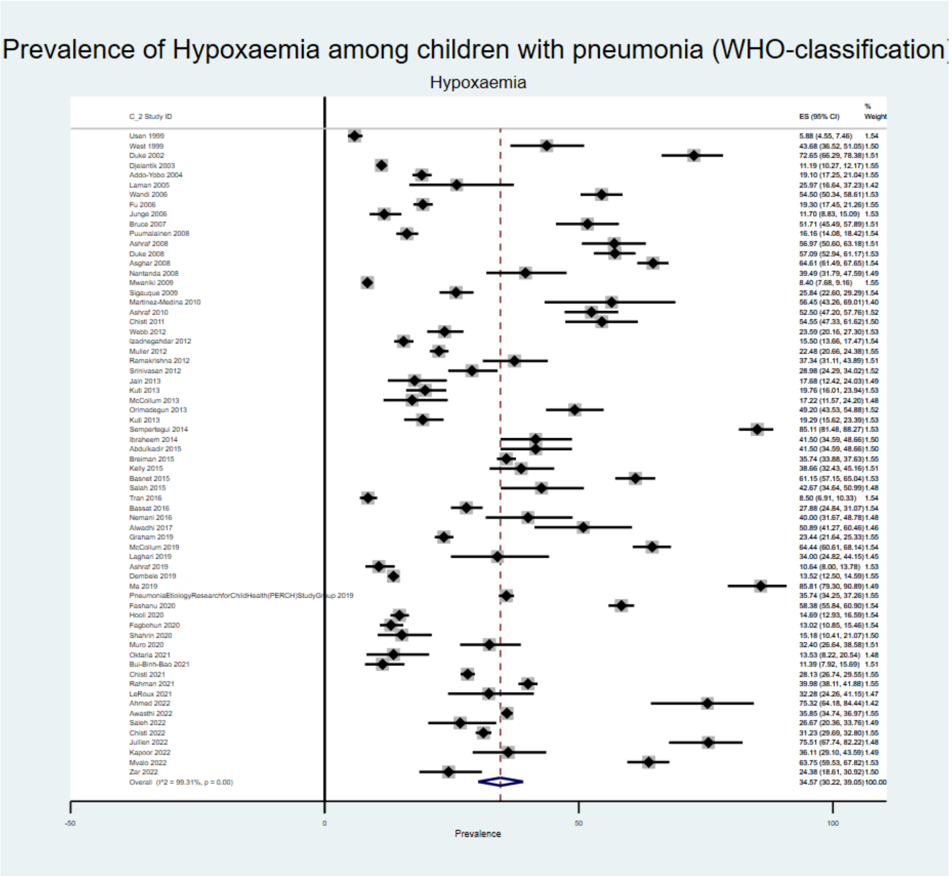

The evidence was assessed as **moderate certainty** because of inconsistency in prevalence estimates between studies.

Admitted Children – pneumonia severe (WHO-classification, severe or very severe)

Table 9: Studies included in prevalence of hypoxaemia among admitted children with severe pneumonia (WHO-Severe)

#	Study ID	WB Region	Prop	LCL	UCL	Wt	Denom
	Djelantik 2003	East Asia & Pacific	11.19	10.27	12.17	2.92	4306
	Addo-Yobo 2004	Mixed	19.10	17.25	21.04	2.91	1702
	Laman 2005	East Asia & Pacific	25.97	16.64	37.23	2.71	77
	Fu 2006	Mixed	19.30	17.45	21.26	2.91	1694
	Puumalainen 2008	East Asia & Pacific	19.54	16.94	22.34	2.90	865
	Ashraf 2008	South Asia	56.97	50.60	63.18	2.85	251
	Asghar 2008	Mixed	64.61	61.49	67.65	2.90	958
	Nantanda 2008	Sub Saharan Africa	39.49	31.79	47.59	2.81	157
	Mwaniki 2009	Sub Saharan Africa	9.33	8.52	10.19	2.92	4792
	Sigauque 2009	Sub Saharan Africa	25.84	22.60	29.29	2.89	685
	Ashraf 2010	South Asia	52.50	47.20	57.76	2.87	360
	Webb 2012	Sub Saharan Africa	23.59	20.16	27.30	2.89	568
	Izadnegahdar 2012	Mixed	15.50	13.66	17.47	2.91	1439
	Ramakrishna 2012	Sub Saharan Africa	37.34	31.11	43.89	2.85	233
	Srinivasan 2012	Sub Saharan Africa	28.98	24.29	34.02	2.87	352
	Kuti 2013	Sub Saharan Africa	19.76	16.01	23.94	2.88	410
	McCollum 2013	Sub Saharan Africa	17.39	11.47	24.76	2.80	138
	Orimadegun 2013	Sub Saharan Africa	49.20	43.53	54.88	2.86	313
	Kuti 2013	Sub Saharan Africa	19.29	15.62	23.39	2.88	420

	Sempertegui 2014	Latin America & Caribbean	85.11	81.48	88.27	2.88	450
	Breiman 2015	Sub Saharan Africa	35.74	33.88	37.63	2.91	2563
	Basnet 2015	South Asia	61.15	57.15	65.04	2.89	610
	Salah 2015	Sub Saharan Africa	48.05	36.52	59.74	2.71	77
	Bassat 2016	Sub Saharan Africa	27.88	24.84	31.07	2.90	825
	Nemani 2016	South Asia	100.00	89.72	100.00	2.48	34
	Alwadhi 2017	South Asia	50.89	41.27	60.46	2.77	112
	Graham 2019	Sub Saharan Africa	36.09	33.13	39.12	2.90	1017
	McCollum 2019	Sub Saharan Africa	64.44	60.61	68.14	2.89	644
	Dembele 2019	East Asia & Pacific	13.52	12.50	14.59	2.92	4179
	PERCHStudyGroup 2019	Mixed	35.74	34.25	37.26	2.92	3981
	Shahrin 2020	South Asia	15.18	10.41	21.07	2.83	191
	Muro 2020	Sub Saharan Africa	32.40	26.64	38.58	2.85	250
	Rahman 2021	South Asia	39.98	38.11	41.88	2.91	2646
	Kapoor 2022	South Asia	36.11	29.10	43.59	2.82	180
	Mvalo 2022	Sub Saharan Africa	63.75	59.53	67.82	2.89	538
Overall (I2=99.41%, T2=0.16, p<0.05)			36.37	30.01	42.99		38017

Fig. 9: Studies included in prevalence of hypoxaemia among admitted children with severe pneumonia (WHO-Severe)

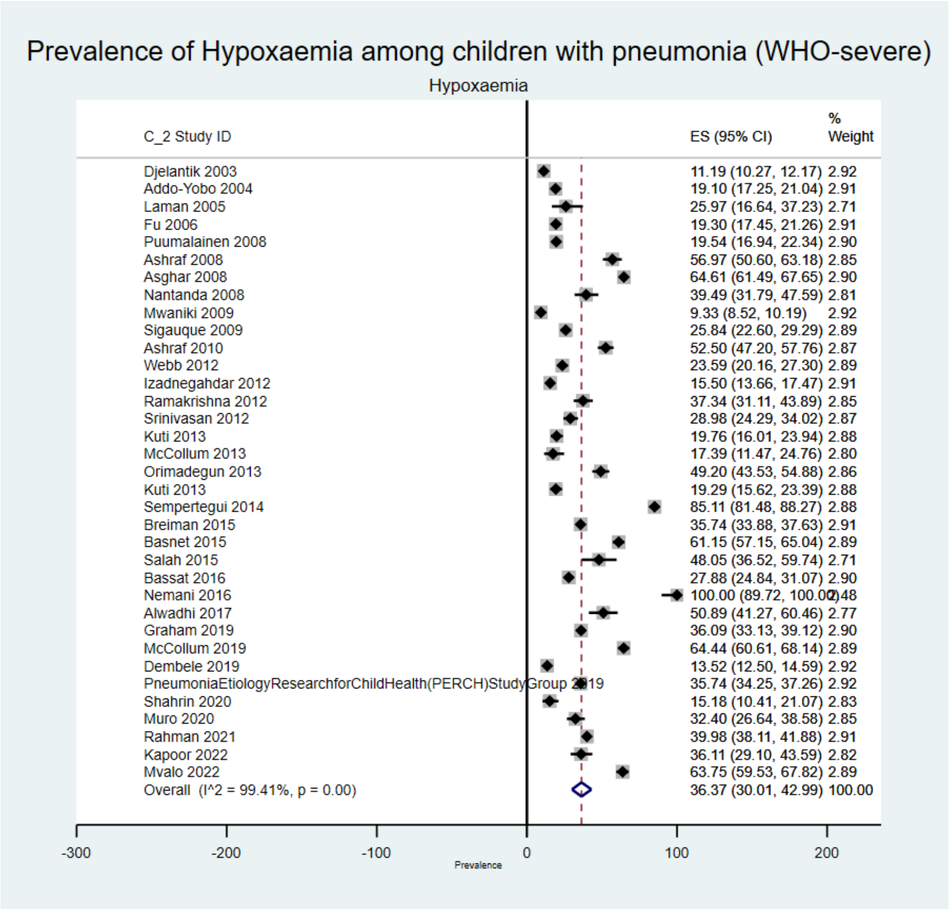

The evidence was assessed as **moderate certainty** because of inconsistency in prevalence estimates between studies.

Admitted Children – pneumonia non-severe (WHO-classification)

Table 10: Studies included in prevalence of hypoxaemia among admitted children with non-severe pneumonia (WHO-non-severe)

Serial No	Study ID	WB Region	Proportion	LCL	UCL	Weight	Denominator
1	Puumalainen 2008	East Asia & Pacific	5.94	3.50	9.35	13.21	286
2	Mwaniki 2009	Sub Saharan Africa	2.01	1.10	3.35	13.43	697
3	McCollum 2013	Sub Saharan Africa	15.38	1.92	45.45	8.37	13
4	Salah 2015	Sub Saharan Africa	36.99	25.97	49.09	12.20	73
5	Nemani 2016	South Asia	19.80	12.54	28.91	12.55	101
6	Bassat 2016	Sub Saharan Africa	27.88	24.84	31.07	13.46	825
7	Graham 2019	Sub Saharan Africa	16.60	13.95	19.53	13.44	717
8	Ashraf 2019	South Asia	10.64	8.00	13.78	13.35	470
**Overall (I2=97.75%, T2=0.12, p<0.05)**			**14.90**	**6.98**	**25.00**		**3182**

Fig. 10: Studies included in prevalence of hypoxaemia among admitted children with non-severe pneumonia (WHO-non-severe)

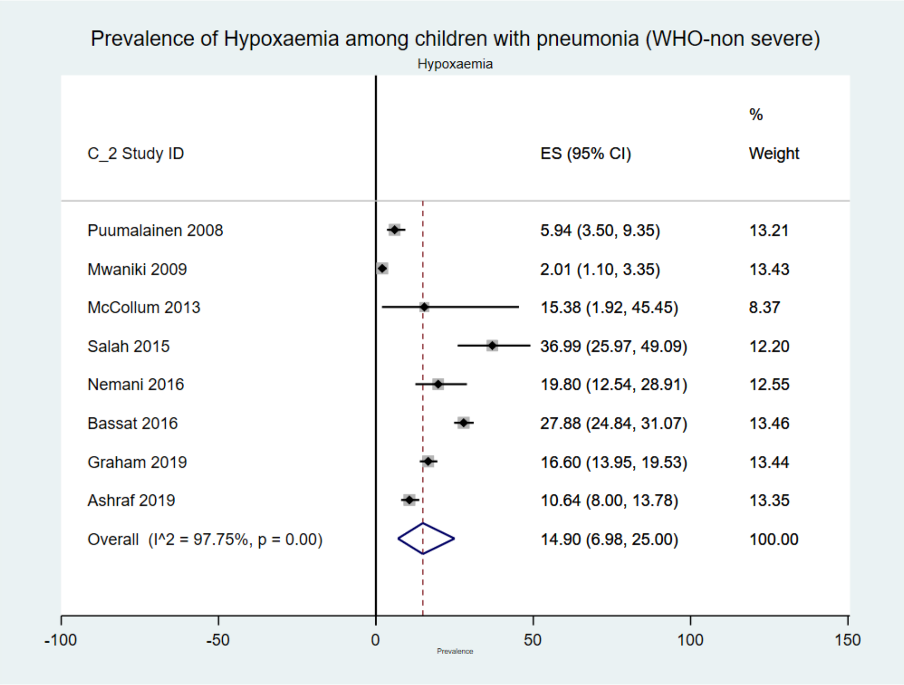

The evidence was assessed as **low certainty** because of imprecision of the estimate, inconsistency in prevalence estimates between studies.

Admitted Children – pneumonia radiological

Table 11: Studies included in prevalence of hypoxaemia among admitted children with pneumonia (radiological)

Serial	Study ID	WB Region	Proportion	LCL	UCL	Weight	Denominator
1.	Usen 1999	Sub Saharan Africa	7.06	3.70	12.01	6.71	170
2.	Magree 2005	East Asia & Pacific	64.41	50.87	76.45	6.45	59
3.	Wandi 2006	East Asia & Pacific	54.50	50.34	58.61	6.82	578
4.	Bruce 2007	Latin America & Caribbean	62.50	51.53	72.60	6.58	88
5.	Tiewsoh 2009	South Asia	68.50	59.67	76.45	6.66	127
6.	Martinez-Medina 2010	Latin America & Caribbean	56.45	43.26	69.01	6.47	62
7.	Chisti 2013	South Asia	11.08	7.92	14.95	6.78	334
8.	Bassat 2016	Sub Saharan Africa	36.68	31.38	42.23	6.78	319
9.	Benet 2017	Mixed	17.28	13.73	21.33	6.80	405
10.	Alwadhi 2017	South Asia	53.76	43.12	64.16	6.59	93
11.	PERCHStudyGroup 2019	Mixed	44.55	42.32	46.80	6.85	1935
12.	Merida-Vieyra 2019	Latin America & Caribbean	96.10	91.71	98.56	6.70	154
13.	Fagbohun 2020	Latin America & Caribbean	13.02	10.85	15.46	6.83	860
14.	Saleh 2022	Middle East & North Africa	26.67	20.36	33.76	6.72	180
15.	Jullien 2022	South Asia	82.05	66.47	92.46	6.26	39
**Overall (I2=98.81%, T2=0.26, p<0.05)**			**45.32**	**32.44**	**58.52**		**5403**

Fig. 11: Studies included in prevalence of hypoxaemia among admitted children with pneumonia (radiological)

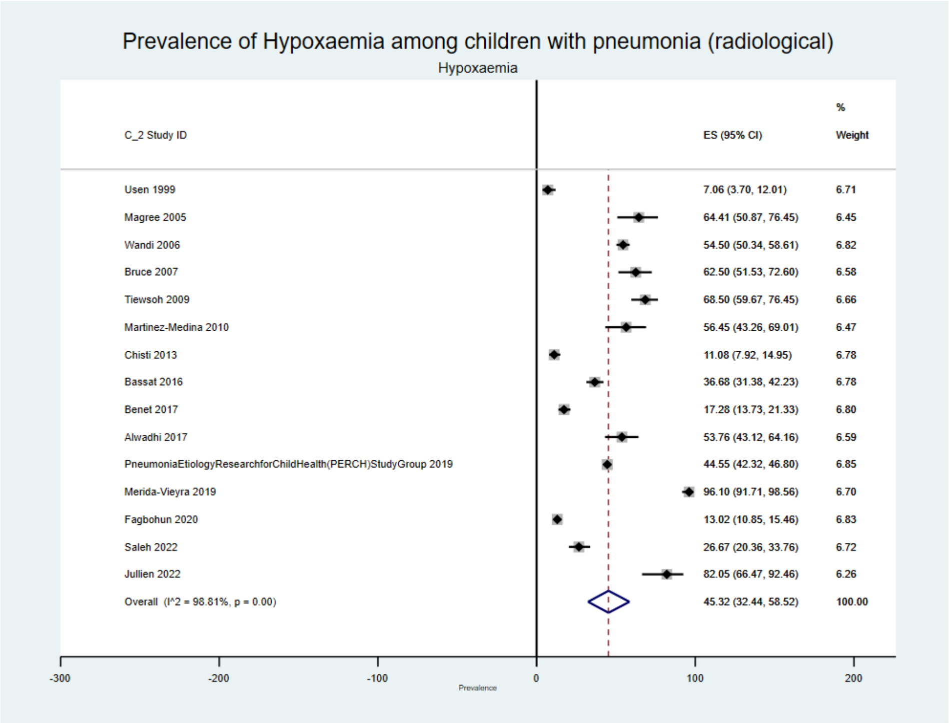

The evidence was assessed as **low certainty** because of imprecision of the estimate, inconsistency in prevalence estimates between studies.

Admitted Children - bronchiolitis

Table 12: Studies included in prevalence of hypoxaemia among admitted children with bronchiolitis

Serial No	Study ID	WB Region	Proportion	LCL	UCL	Weight	Denominator
1	Chan 2002	East Asia & Pacific	14.35	9.96	19.75	25.25	216
2	HongXIAO 2010	East Asia & Pacific	0.00	0.00	11.22	24.10	31
3	Libster 2010	Latin America & Caribbean	82.07	76.76	86.61	25.28	251
4	Nyawanda 2016	Sub Saharan Africa	25.74	21.85	29.95	25.36	470
**Overall (I2=99.16%, T2=0.56, p<0.05)**			**26.31**	**2.38**	**62.75**		**968**

Fig. 12: Studies included in prevalence of hypoxaemia among admitted children with bronchiolitis

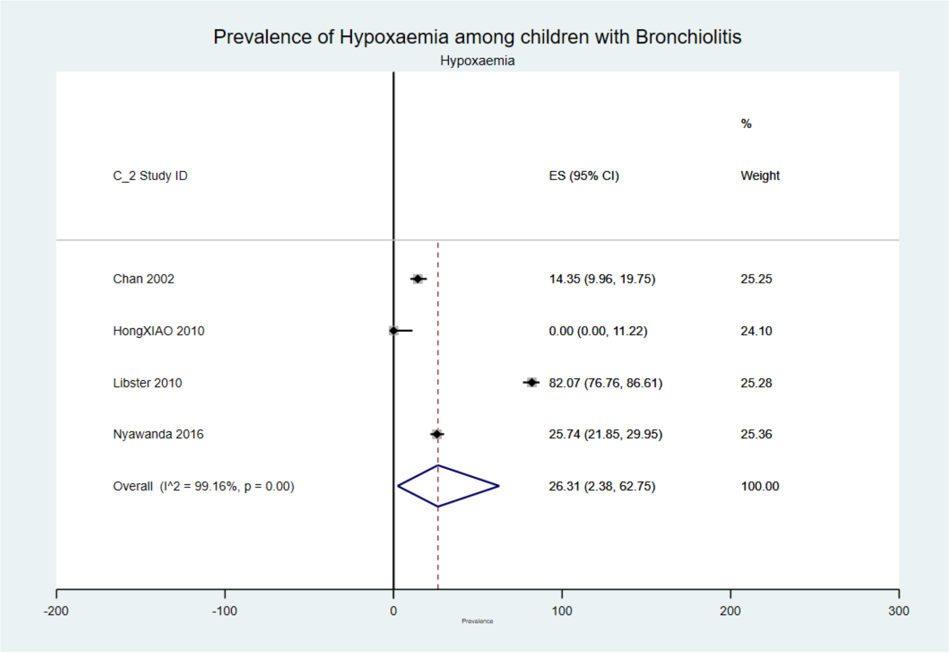

The evidence was assessed as **very low certainty** because of moderate risk of bias (variable representativeness of population), imprecision of the estimate, inconsistency in prevalence estimates between studies.

Admitted Children – pneumonia unspecified

Table 13: Studies included in Prevalence of hypoxaemia among admitted children with pneumonia (unspecified)

Serial No	Study ID	WB Region	Proportion	LCL	UCL	Weight	Denominator
1	Magree 2005	East Asia & Pacific	16.53	12.13	21.75	14.27	248
2	Riccetto 2006	Latin America & Caribbean	22.15	15.76	29.67	13.89	149
3	Nyawanda 2016	Sub Saharan Africa	16.76	15.70	17.86	14.86	4696
4	Zampoli 2017	Sub Saharan Africa	70.87	64.16	76.98	14.15	206
5	Ling 2020	East Asia & Pacific	20.00	15.91	24.62	14.44	345
6	AbdelGhaffar 2022	Middle East & North Africa	33.13	31.50	34.80	14.85	3172
7	King 2022	Sub Saharan Africa	29.63	21.23	39.18	13.54	108
**Overall (I2=98.78%, T2=0.09, p<0.05)**			**28.94**	**19.14**	**39.84**		**8924**

Fig. 13: Studies included in Prevalence of hypoxaemia among admitted children with pneumonia (unspecified)

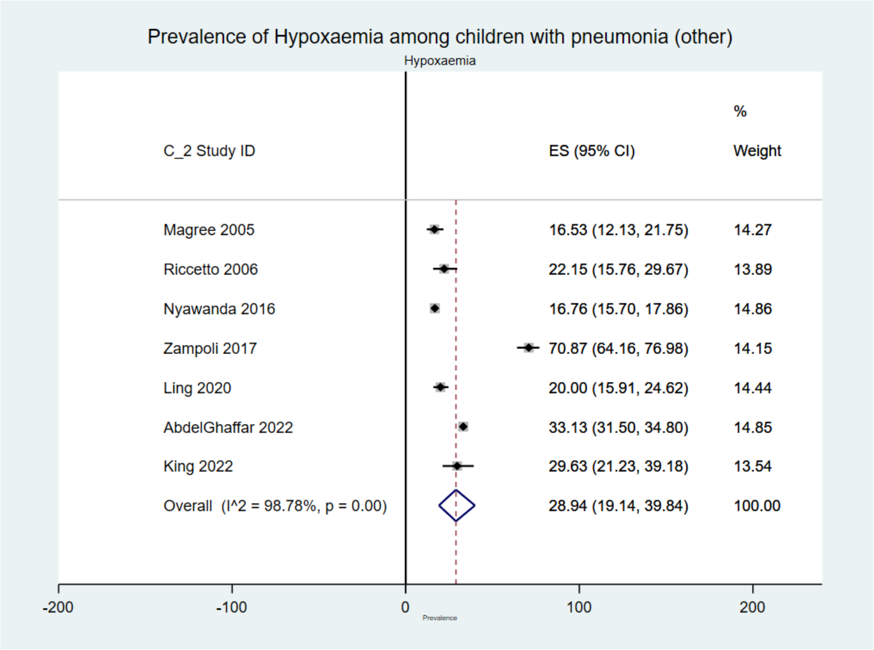

The evidence was assessed as **low certainty** because of moderate risk of bias (variable representativeness of population), imprecision of the estimate.

Admitted Children - anaemia

Table 14: Studies included in prevalence of hypoxaemia among admitted children with anaemia

Serial No	Study ID	WB Region	Proportion	LCL	UCL	Weight	Denominator
1	Wandi 2006	East Asia & Pacific	3.17	0.39	11.00	16.23	63
2	Junge 2006	Sub Saharan Africa	1.78	0.49	4.49	17.53	225
3	Mwaniki 2009	Sub Saharan Africa	4.18	2.84	5.92	17.91	717
4	McCollum 2013	Sub Saharan Africa	3.23	0.08	16.70	14.69	31
5	Orimadegun 2014	Sub Saharan Africa	28.47	23.33	34.06	17.65	288
6	King 2022	Sub Saharan Africa	12.73	5.27	24.48	15.99	55
**Overall (I2=96.14%, T2=0.14, p<0.05)**			**7.39**	**1.14**	**17.71**		**1379**

Fig. 14: Studies included in prevalence of hypoxaemia among admitted children with anaemia (unspecified)

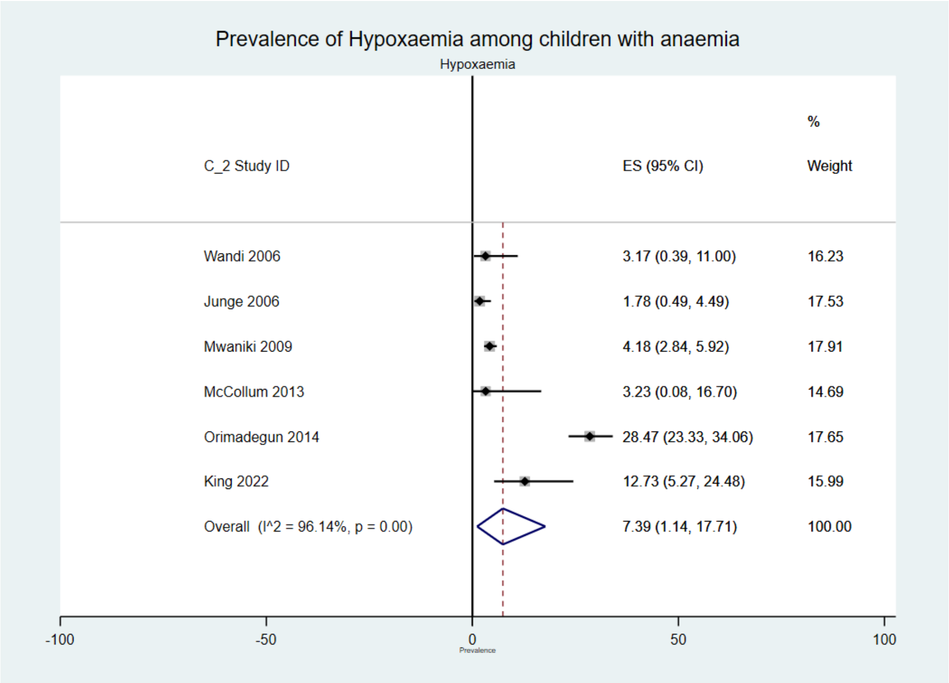

The evidence was assessed as **very low certainty** because of moderate risk of bias (variable representativeness of population), imprecision of the estimate, inconsistency in prevalence estimates between studies.

Admitted Children - asthma

Table 15: Studies included in prevalence of hypoxaemia among admitted children with asthma

Serial No	Study ID	WB Region	Proportion	LCL	UCL	Weight	Denominator
1	Rahnama'i 2006	South Asia	45.10	31.13	59.66	33.06	51
2	Jain 2018	South Asia	83.33	71.48	91.71	33.25	60
3	Graham 2019	Sub Saharan Africa	20.41	12.93	29.74	33.68	98
**Overall (I2=97.03%, T2=0.49, p<0.05)**			**49.76**	**13.35**	**86.31**		**209**

Fig. 15: Studies included in prevalence of hypoxaemia among admitted children with asthma

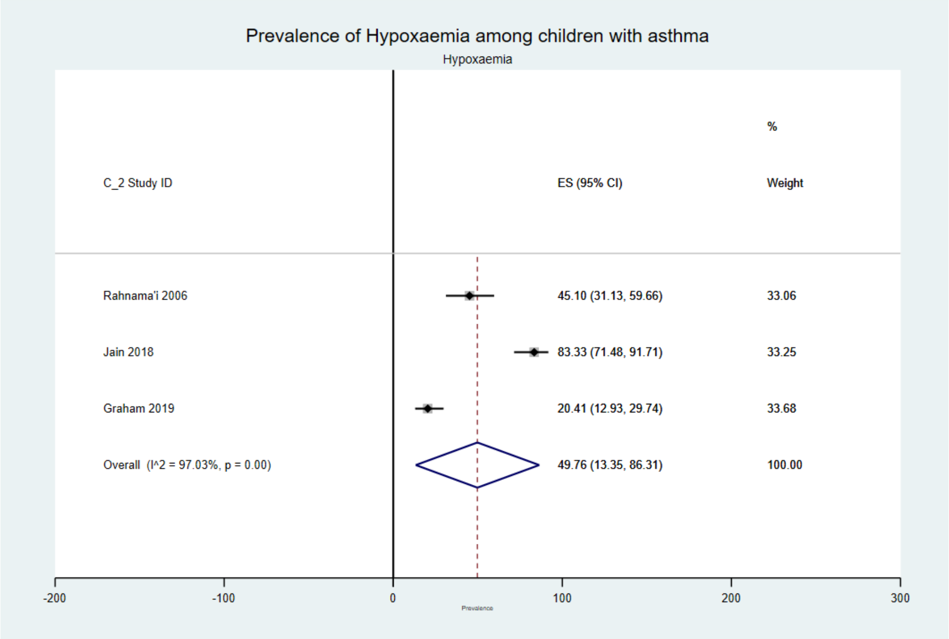

The evidence was assessed as **very low certainty** because of moderate risk of bias (variable representativeness of population), imprecision of the estimate, inconsistency in prevalence estimates between studies.

Admitted Children – HIV complication

Table 16: Studies included in Prevalence of hypoxaemia among admitted children with HIV complication

Serial No	Study ID	WB Region	Proportion	LCL	UCL	Weight	Denominator
1	Kelly 2015	Sub Saharan Africa	55.00	31.53	76.94	29.75	20
2	Njuguna 2018	Sub Saharan Africa	27.85	21.02	35.53	40.97	158
3	Graham 2019	Sub Saharan Africa	10.53	1.30	33.14	29.28	19
**Overall (I2=78.30%, T2=0.11, p<0.05)**			**29.47**	**11.86**	**50.69**		**197**

Fig. 16: Studies included in Prevalence of hypoxaemia among admitted children with HIV complication

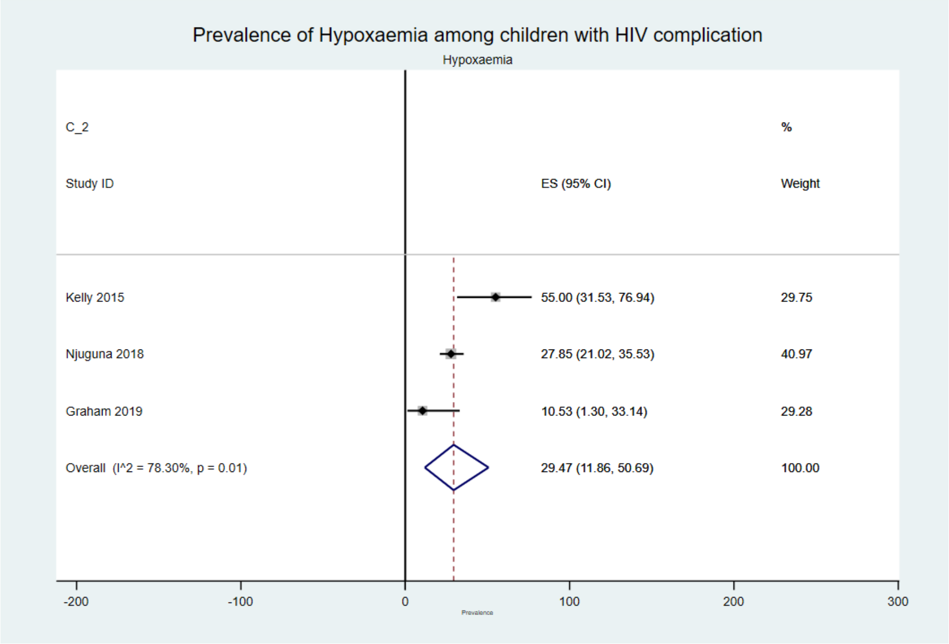

The evidence was assessed as **very low certainty** because of moderate risk of bias (variable representativeness of population), imprecision of the estimate, inconsistency in prevalence estimates between studies.

Admitted Children - malaria

Table 17: Studies Included in prevalence of hypoxaemia among admitted children with malaria

Serial No	Study ID	WB Region	Proportion	LCL	UCL	Weight	Denominator
1	Usen 1999	Sub Saharan Africa	3.88	1.07	9.65	6.29	103
2	Duke 2002	East Asia & Pacific	0.00	0.00	52.18	1.27	5
3	Maitland 2003	Sub Saharan Africa	17.17	13.97	20.76	7.63	501
4	Wandi 2006	East Asia & Pacific	3.31	1.52	6.19	7.29	272
5	Junge 2006	Sub Saharan Africa	2.87	1.95	4.08	7.85	1044
6	Mwaniki 2009	Sub Saharan Africa	4.90	4.31	5.53	8.03	4982
7	Cserti-Gazdewich 2013	Sub Saharan Africa	2.26	1.64	3.03	7.95	1901
8	Orimadegun 2013	Sub Saharan Africa	14.42	11.53	17.71	7.65	527
9	McCollum 2013	Sub Saharan Africa	1.87	0.81	3.66	7.55	427
10	Orimadegun 2014	Sub Saharan Africa	29.81	25.19	34.76	7.48	369
11	Graham 2019	Sub Saharan Africa	8.50	7.74	9.31	8.03	5035
12	Olupot-Olupot 2020	Sub Saharan Africa	7.40	5.53	9.67	7.73	662
13	Leligdowicz 2021	Sub Saharan Africa	2.51	1.73	3.50	7.90	1317
14	King 2022	Sub Saharan Africa	9.03	6.09	12.79	7.37	310
**Overall (I2=97.37%, T2=0.03, p<0.05)**			**6.42**	**3.99**	**9.32**		**17455**

Fig. 17: Studies Included in prevalence of hypoxaemia among admitted children with malaria

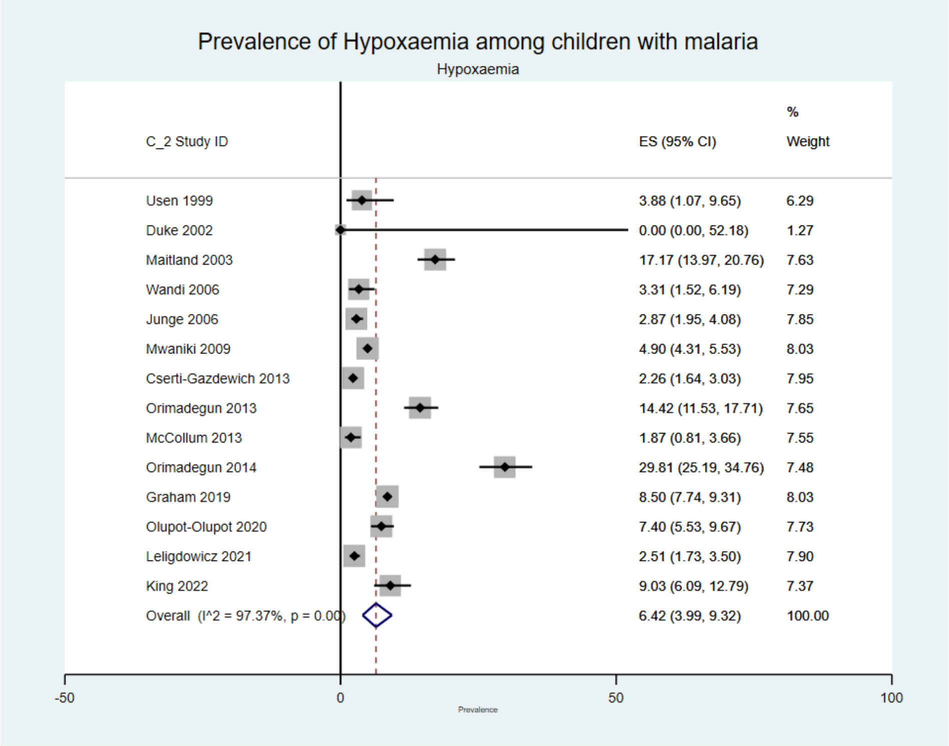

The evidence was assessed as **high certainty**.

Admitted Children – meningitis / encephalitis

Table 18: Studies included in prevalence of hypoxaemia among admitted children with meningitis

Serial No	Study ID	WB Region	Proportion	LCL	UCL	Weight	Denominator
1	Weber 2002	Sub Saharan Africa	4.49	1.24	11.11	17.23	89
2	Duke 2002	East Asia & Pacific	52.50	36.13	68.49	15.55	40
3	Wandi 2006	East Asia & Pacific	14.63	5.57	29.17	15.62	41
4	Junge 2006	Sub Saharan Africa	2.70	0.33	9.42	16.93	74
5	Orimadegun 2013	Sub Saharan Africa	8.00	2.22	19.23	16.12	50
6	Graham 2019	Sub Saharan Africa	17.40	13.94	21.32	18.55	431
**Overall (I2=91.02%, T2=0.11, p<0.05)**			**13.70**	**5.13**	**25.26**		**725**

Fig. 18: Studies included in prevalence of hypoxaemia among admitted children with meningitis

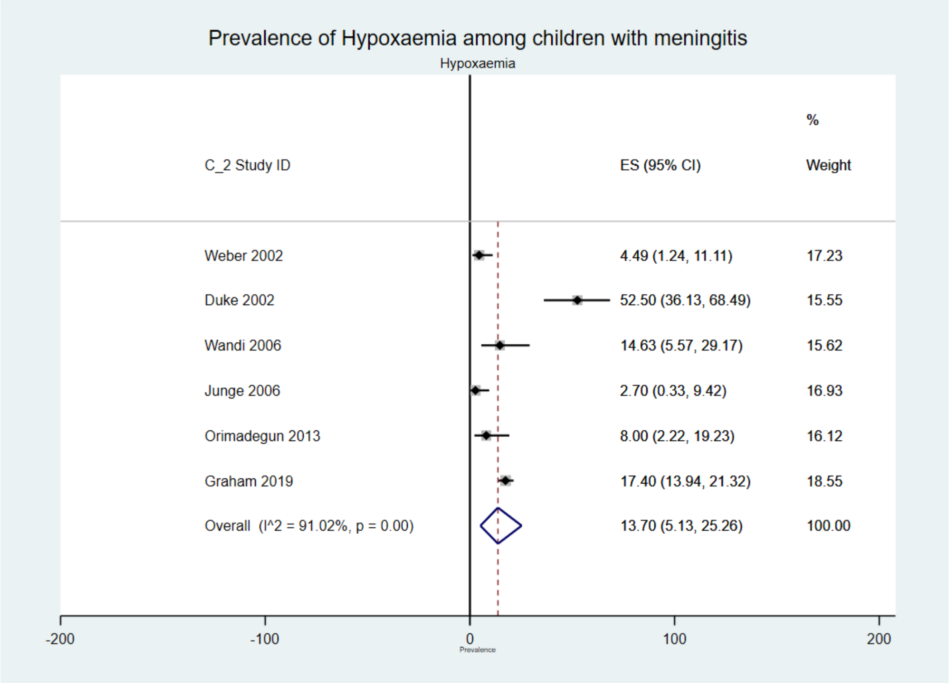

The evidence was assessed as **low certainty** because of moderate risk of bias (variable representativeness of population), imprecision of the estimate.

Admitted Children - seizures

Table 19: Studies included in prevalence of hypoxaemia among admitted children with seizure.

Serial	Study ID	WB Region	Proportion	LCL	UCL	Denominator
1.	Weber 2002	Sub Saharan Africa	4.05	3.25	4.99	2097
2.	Graham 2019	Sub Saharan Africa	18.29	16.23	20.50	1301

Fig. 19: Studies included in prevalence of hypoxaemia among admitted children with seizure.

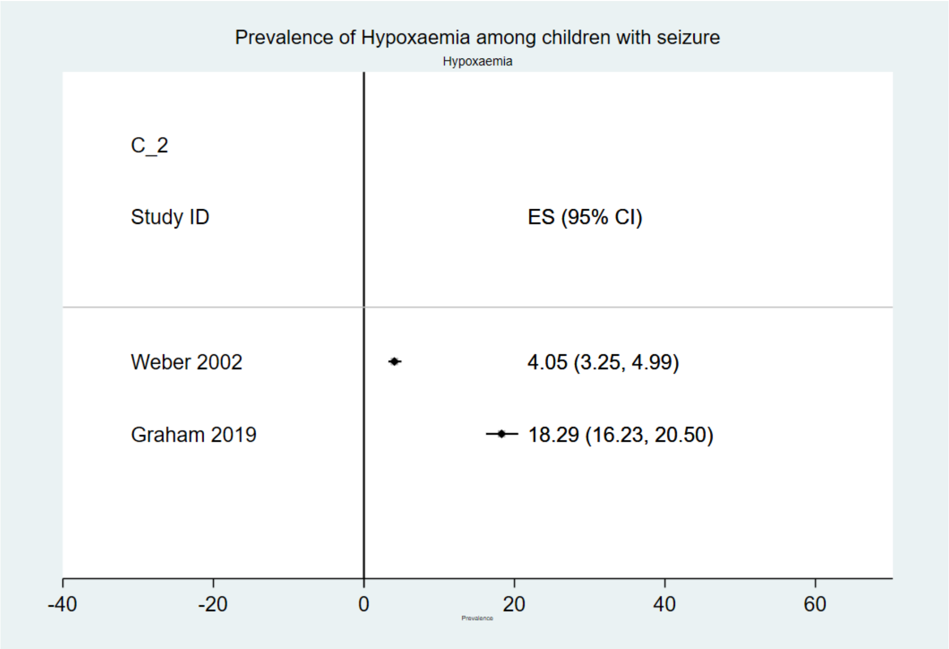

No meta-estimate calculated as only two included studies.

Admitted Children – sepsis

Table 20: Studies included in prevalence of hypoxaemia among admitted children with sepsis.

Serial	Study ID	WB Region	Proportion	LCL	UCL	Denominator
1.	Duke 2002	East Asia & Pacific	60.00	26.24	87.84	10
2.	Santhanam 2008	South Asia	27.89	20.82	35.88	147
3.	Orimadegun 2013	Sub Saharan Africa	22.62	16.53	29.70	168
4.	McCollum 2013	Sub Saharan Africa	3.45	0.09	17.76	29
5.	Rudd 2014	Sub Saharan Africa	29.03	14.22	48.04	31
6.	Shahid 2016	South Asia	23.08	8.97	43.65	26
7.	Graham 2019	Sub Saharan Africa	8.70	7.85	9.61	4092
8.	Shahunja 2020	South Asia	16.96	13.42	21.00	401
9.	King 2022	Sub Saharan Africa	13.04	6.93	21.68	92
**Overall (I2=92.40%, T2=0.06, p<0.05)**			**18.66**	**11.80**	**26.58**	**4996**

Fig. 20: Studies included in prevalence of hypoxaemia among admitted children with sepsis.

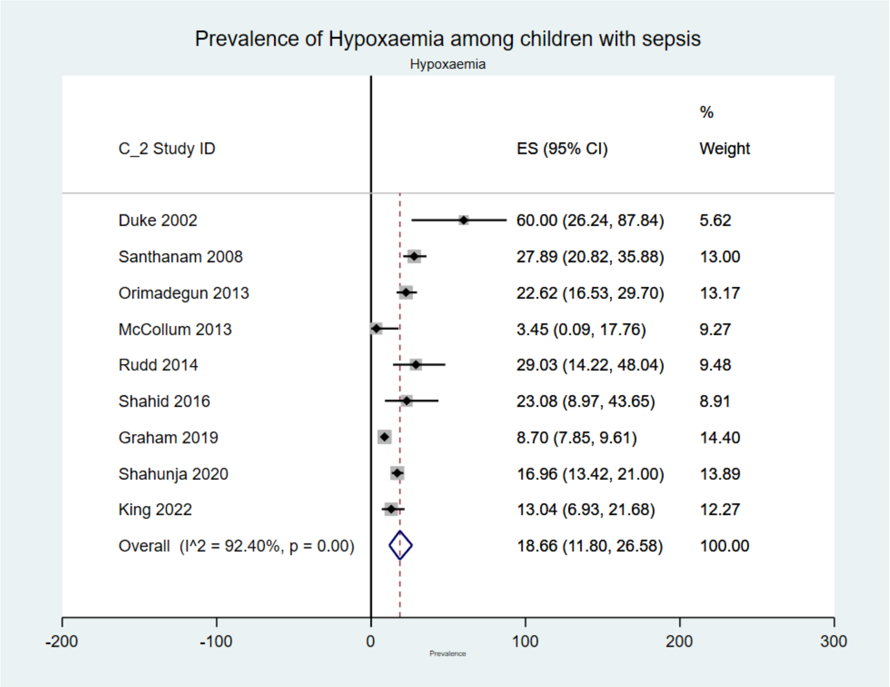

The evidence was assessed as **moderate certainty** because of imprecision of the estimate.

Admitted Children – trauma / injury

Table 21: Studies included in prevalence of hypoxaemia among admitted children with trauma.

Serial	Study ID	WB Region	Proportion	LCL	UCL	Denominator
1.	Graham 2019	Sub Saharan Africa	7.09	4.39	10.74	282
2.	King 2022	Sub Saharan Africa	2.00	0.55	5.04	200

Fig. 21: Studies included in prevalence of hypoxaemia among admitted children with trauma.

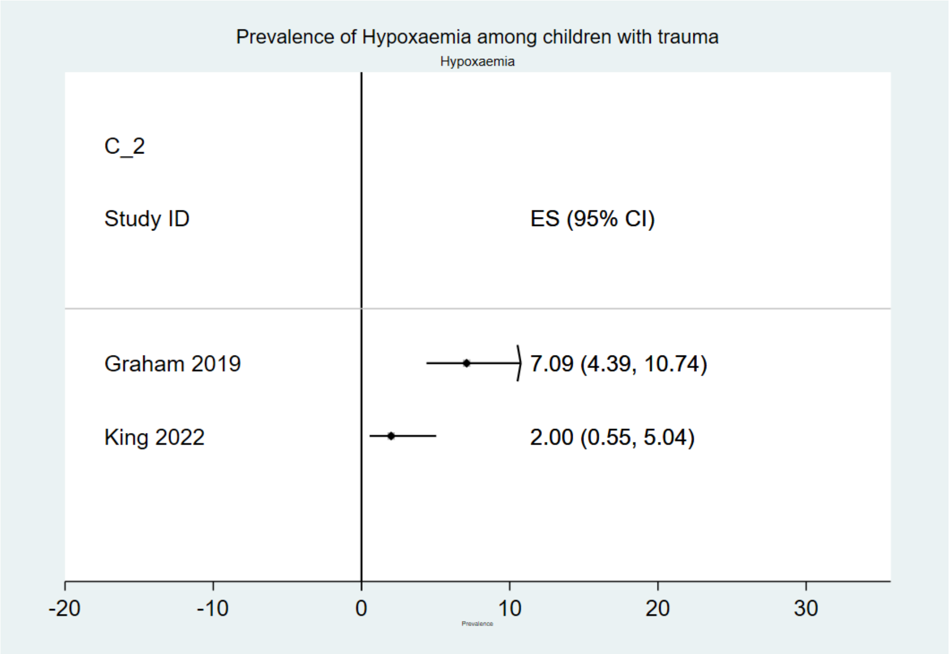

No meta-estimate calculated as only two included studies.

Admitted Children - Tuberculosis

Table 22: Studies included in prevalence of hypoxaemia among admitted children with tuberculosis.

Serial	Study ID	WB Region	Proportion	LCL	UCL	Denominator
1.	Wandi 2006	East Asia & Pacific	20.00	5.73	43.66	20

Fig. 22: Studies included in prevalence of hypoxaemia among admitted children with tuberculosis.

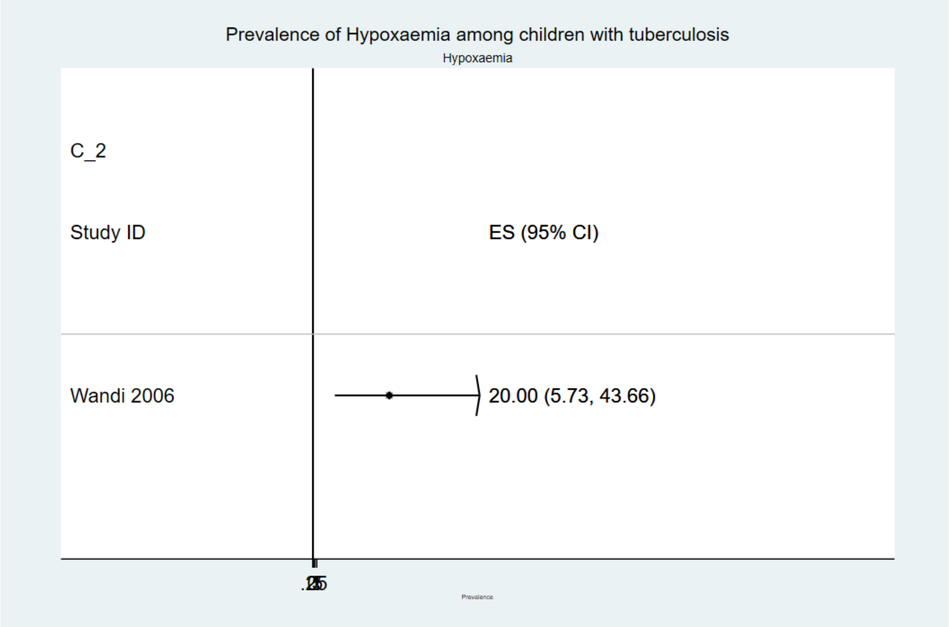

No meta-estimate calculated as only one included studies.

Admitted Children – COVID-19

Table 23: Studies included in prevalence of hypoxaemia among admitted children with Covid-19.

Serial	Study ID	WB Region	Proportion	LCL	UCL	Denominator
1.	Rabha 2020	Latin America & Caribbean	4.347826	1.426583	9.854973	115
2.	Rao 2021	South Asia	19.32773	12.66439	27.57604	119
3.	AbdelGhaffar 2022	Middle East & North Africa	33.13367	31.49606	34.80244	3172
4.	Snouber 2022	Middle East & North Africa	49.10714	39.53714	58.72552	112
5.	Oliveira 2022	Latin America & Caribbean	34.84322	34.20743	35.48309	21591
**Overall (I2=96.17%, T2=0.02, p<0.05)**			**27.50**	**22.02**	**33.34**	**25109**

Fig. 23: Studies included in prevalence of hypoxaemia among admitted children with Covid-19.

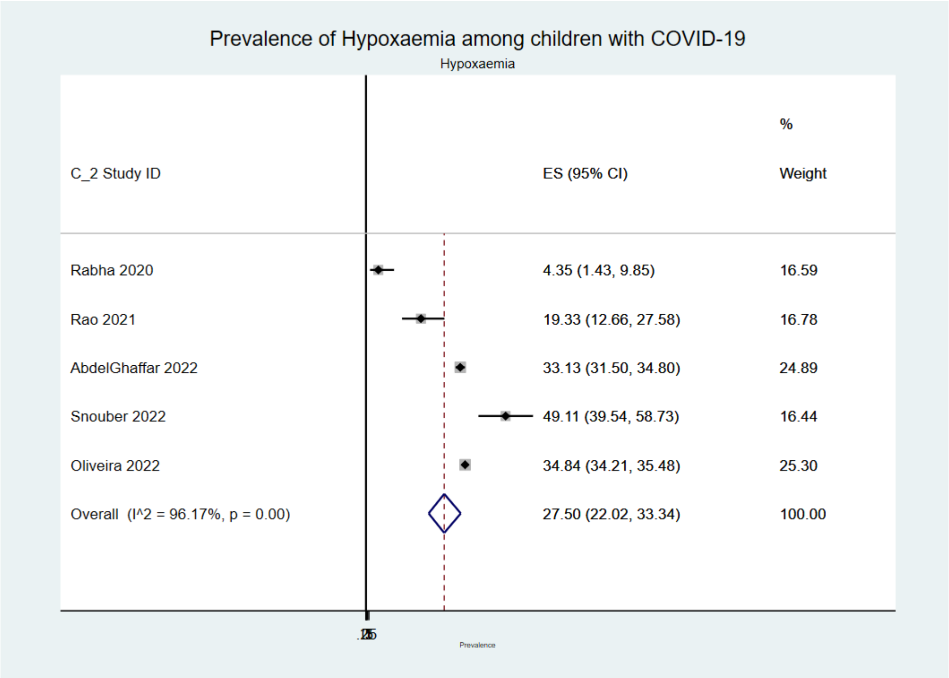

The evidence was assessed as **moderate certainty** because of inconsistency in prevalence estimates between studies.

Admitted Children - diarrhoea

Table 24: Studies included in prevalence of hypoxaemia among admitted children with diarrhoea.

Serial	Study ID	WB Region	Proportion	LCL	UCL	Denominator
1.	Duke 2002	East Asia & Pacific	8.57	1.80	23.06	35
2.	Wandi 2006	East Asia & Pacific	4.72	1.75	10.00	127
3.	Junge 2006	Sub Saharan Africa	0.00	0.00	3.18	114
4.	Mor 2010	Sub Saharan Africa	0.54	0.18	1.26	926
5.	Chisti 2011	South Asia	18.33	9.52	30.44	60
6.	Orimadegun 2013	Sub Saharan Africa	6.38	2.38	13.38	94
7.	McCollum 2013	Sub Saharan Africa	0.00	0.00	11.94	29
8.	Shahid 2016	South Asia	6.73	2.75	13.38	104
9.	Graham 2019	Sub Saharan Africa	6.10	4.91	7.48	1410
10.	King 2022	Sub Saharan Africa	5.56	0.14	27.29	18
**Overall (I2=91.25%, T2=0.05, p<0.05)**			**4.09**	**1.32**	**7.99**	**2917**

Fig. 24: Studies included in prevalence of hypoxaemia among admitted children with diarrhoea.

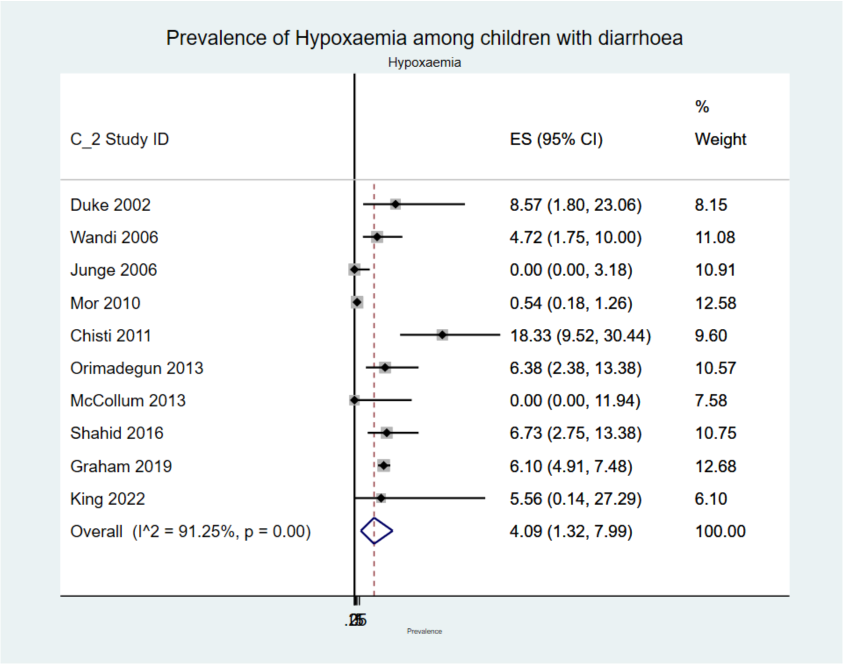

The evidence was assessed as **low certainty** because of imprecision of the estimate, inconsistency in prevalence estimates between studies.

Admitted Children - malnutrition

Table 25: Studies included in prevalence of hypoxaemia among admitted children with malnutrition.

Serial	Study ID	WB Region	Proportion	LCL	UCL	Denominator
1.	Duke 2002	East Asia & Pacific	23.81	8.22	47.17	21
2.	Wandi 2006	East Asia & Pacific	8.33	0.21	38.48	12
3.	Maitland 2006	Sub Saharan Africa	26.31	22.86	29.99	612
4.	Junge 2006	Sub Saharan Africa	1.85	0.60	4.25	271
5.	Chisti 2013	South Asia	11.08	7.92	14.95	334
6.	McCollum 2013	Sub Saharan Africa	5.26	0.13	26.03	19
7.	Nemani 2016	South Asia	75.00	61.05	85.97	52
8.	Nabukeera-Barungi 2018	Sub Saharan Africa	13.37	8.84	19.10	187
9.	Graham 2019	Sub Saharan Africa	3.50	2.56	4.65	1286
10.	Ashraf 2019	South Asia	10.64	8.00	13.78	470
11.	Kintwa 2021	East Asia & Pacific	20.71	14.33	28.38	140
12.	Faruk 2022	South Asia	9.80	6.71	13.70	306
13.	King 2022	Sub Saharan Africa	5.56	0.68	18.66	36

**Overall (I2=96.76%, T2=0.12, p<0.05)**	**14.06**	**7.66**	**21.88**	**3746**

Fig. 25: Studies included in prevalence of hypoxaemia among admitted children with malnutrition.

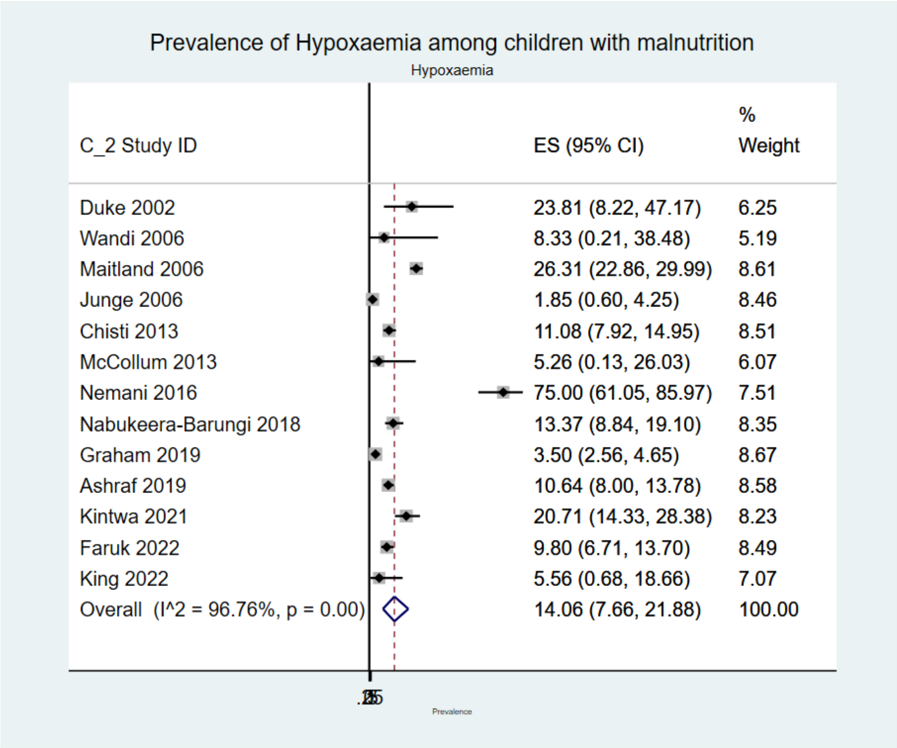

The evidence was assessed as **low certainty** because of imprecision of the estimate, inconsistency in prevalence estimates between studies.

Admitted Adults – all

Table 26: Studies included in prevalence of hypoxaemia among all admitted adults.

Serial	Study ID	WB Region	Proportion	LCL	UCL	Weight	Denominator
1.	Foran 2010	Sub Saharan Africa	9.17	4.49	16.23	13.38	109
2.	Evans 2012	Sub Saharan Africa	9.72	5.42	15.77	13.70	144
3.	Riviello 2016	Sub Saharan Africa	12.05	10.13	14.17	14.62	1046
4.	Wasingya-Kasereka 2020	Sub Saharan Africa	6.31	5.41	7.31	14.71	2599
5.	Aslam 2021	South Asia	15.17	11.25	19.83	14.22	290
6.	Kayambankadzanja 2021	Sub Saharan Africa	3.96	2.91	5.27	14.63	1135
7.	Navuluri 2023	Sub Saharan Africa	23.76	22.46	25.09	14.74	4104
**Overall (I2=98.98%, T2=0.09, p<0.05)**			**10.81**	**4.86**	**18.71**		**9427**

Fig. 26: Studies included in prevalence of hypoxaemia among all admitted adults.

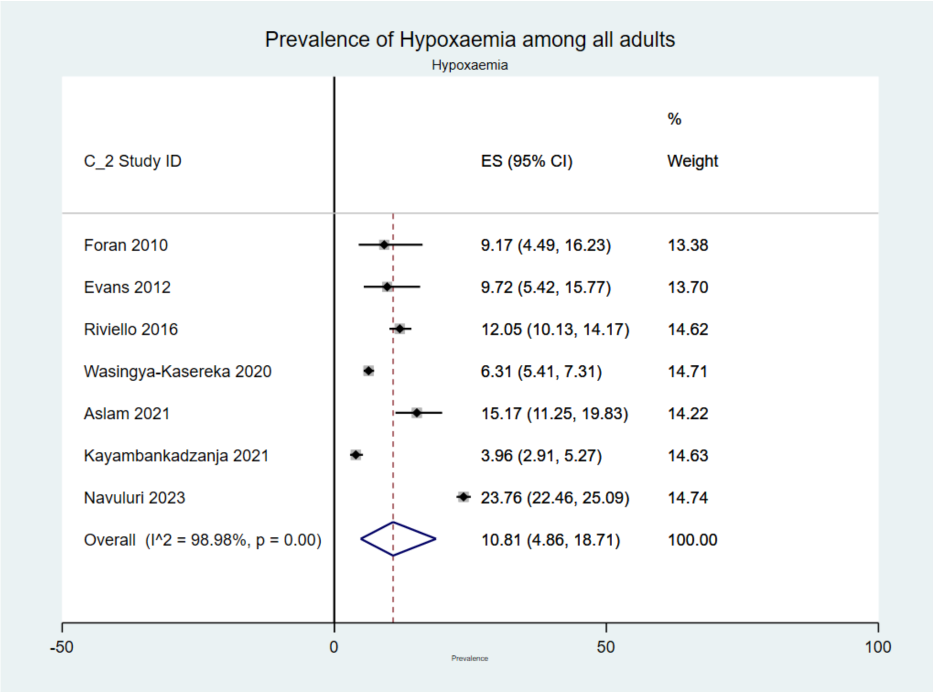

The evidence was assessed as **low certainty** because of imprecision of the estimate, inconsistency in prevalence estimates between studies.

Admitted Adults - fever

Table 27: Studies included in prevalence of hypoxaemia among admitted adults with fever.

Serial	Study ID	WB Region	Proportion	LCL	UCL	Denominator
1.	Carugati 2018	Sub Saharan Africa	9.79	7.11	13.04	419
2.	YeLynn 2019	East Asia & Pacific	21.01	14.08	29.43	119

Fig. 27: Studies included in prevalence of hypoxaemia among admitted adults with fever.

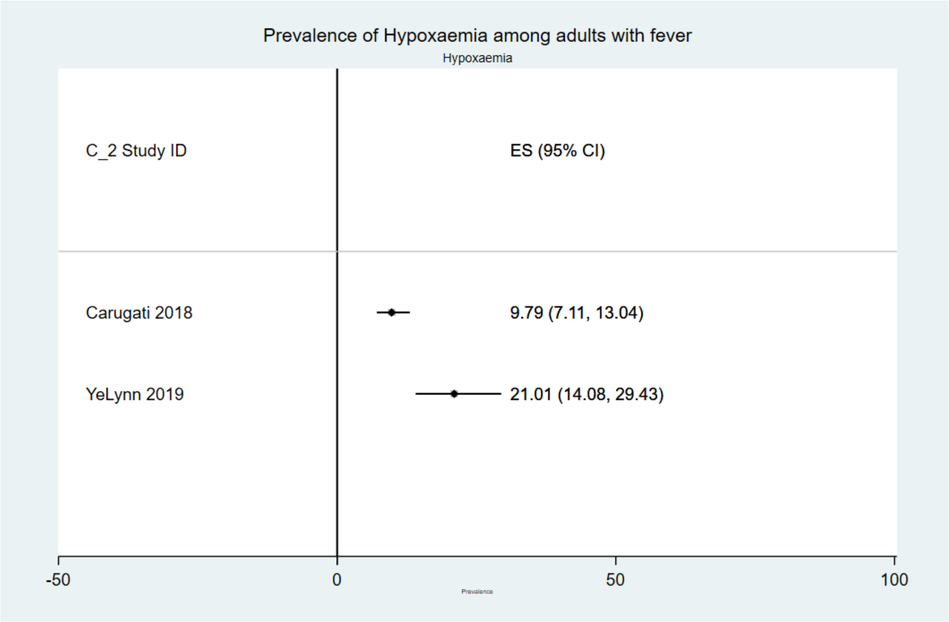

No meta-estimate calculated as only two included studies.

Admitted Adults - pneumonia

Table 28: Studies included in prevalence of hypoxaemia among admitted adults with any pneumonia.

Serial	Study ID	WB Region	Proportion	LCL	UCL	Denominator
1.	HongXIAO 2010	East Asia & Pacific	0.00	0.00	2.91	125
2.	Davis 2010	Sub Saharan Africa	26.24	20.31	32.87	202
3.	Pati 2013	South Asia	5.15	1.69	11.62	97
4.	Tokman 2014	Sub Saharan Africa	15.77	11.41	20.99	241
5.	Worodria 2018	Sub Saharan Africa	10.60	9.25	12.08	1887
6.	Aston 2019	Sub Saharan Africa	16.22	12.94	19.96	450
7.	Ojuawo 2020	Sub Saharan Africa	25.00	17.98	33.14	136
8.	Arana-Calderon 2022	Latin America & Caribbean	90.51	84.83	94.59	158
**Overall (I2=98.82%, T2=0.28, p<0.05)**			**20.36**	**7.70**	**37.03**	**3296**

Fig. 28: Studies included in prevalence of hypoxaemia among admitted adults with any pneumonia.

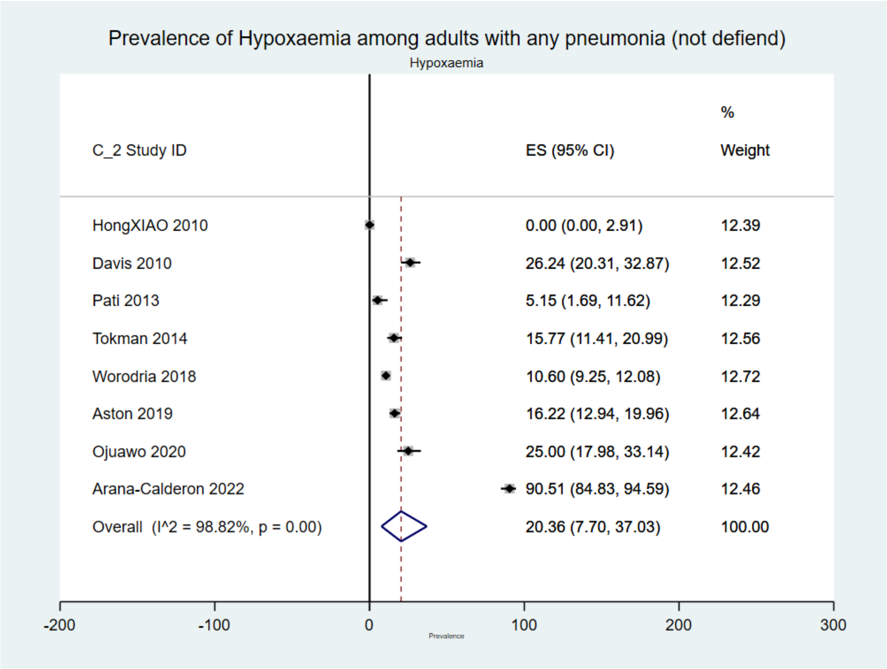

The evidence was assessed as **low certainty** because of imprecision of the estimate, inconsistency in prevalence estimates between studies.

Admitted Adults - asthma

Table 29: Studies included in prevalence of hypoxaemia among admitted adults with asthma.

Serial	Study ID	WB Region	Proportion	LCL	UCL	Denominator
1.	Hussain 2005	South Asia	16.84	9.94	25.90	95

Fig. 29: Studies included in prevalence of hypoxaemia among admitted adults with asthma.

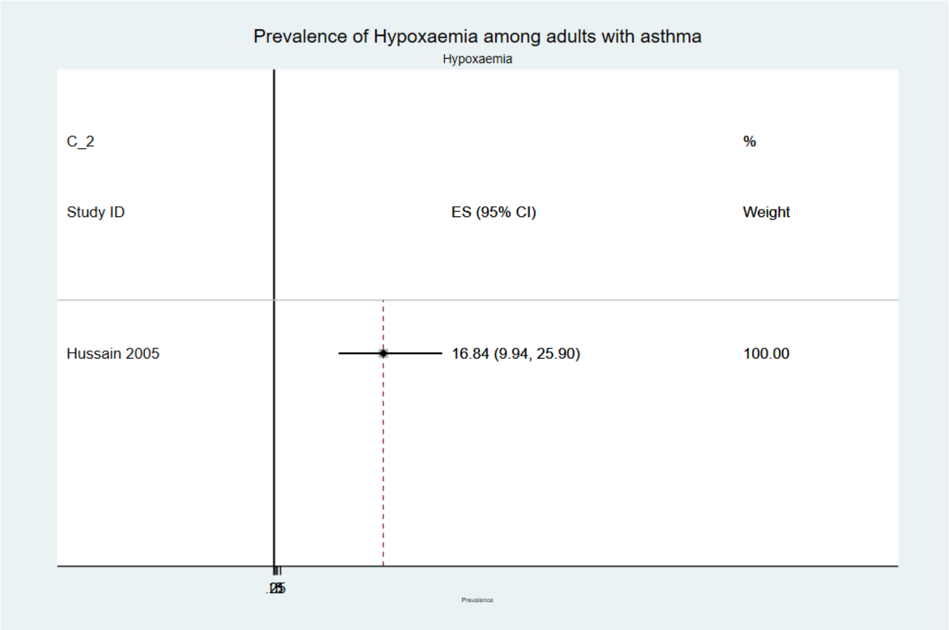

No meta-estimate calculated as only two included studies.

Admitted Adults – HIV complications

Table 30: Studies included in prevalence of hypoxaemia among admitted adults with HIV complications.

Serial	Study ID	WB Region	Proportion	LCL	UCL	Denominator
1.	Davis 2010	Sub Saharan Africa	26.24	20.31	32.87	202
2.	Koss 2015	Sub Saharan Africa	16.41	13.96	19.10	835
3.	Worodria 2018	Sub Saharan Africa	11.41	9.66	13.35	1192
4.	Carugati 2018	Sub Saharan Africa	15.00	9.85	21.49	160
**Overall (I2=90.08%, T2=0.02, p<0.05)**			**16.64**	**11.62**	**22.35**	**2389**

Fig. 30: Studies included in prevalence of hypoxaemia among admitted adults with HIV complications.

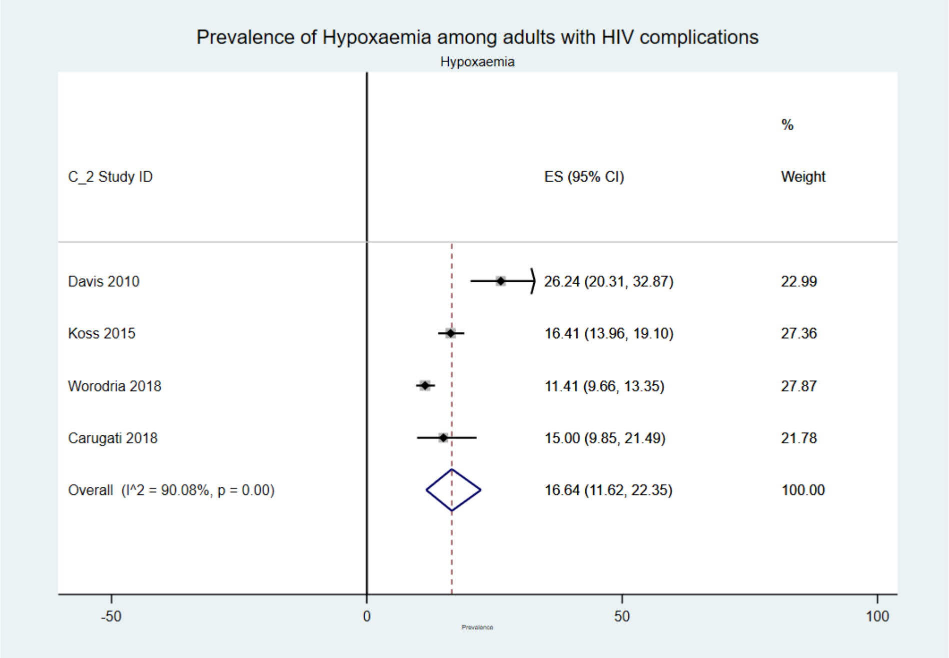

The evidence was assessed as **low certainty** because of imprecision of the estimate, inconsistency in prevalence estimates between studies.

Admitted Adults - seizures

Table 31: Studies included in prevalence of hypoxaemia among admitted adults with seizure.

Serial	Study ID	WB Region	Proportion	LCL	UCL	Denominator
1.	Amare 2008	Sub Saharan Africa	25.21008	17.70029	33.99314	119

Fig. 31: Studies included in prevalence of hypoxaemia among admitted adults with seizure.

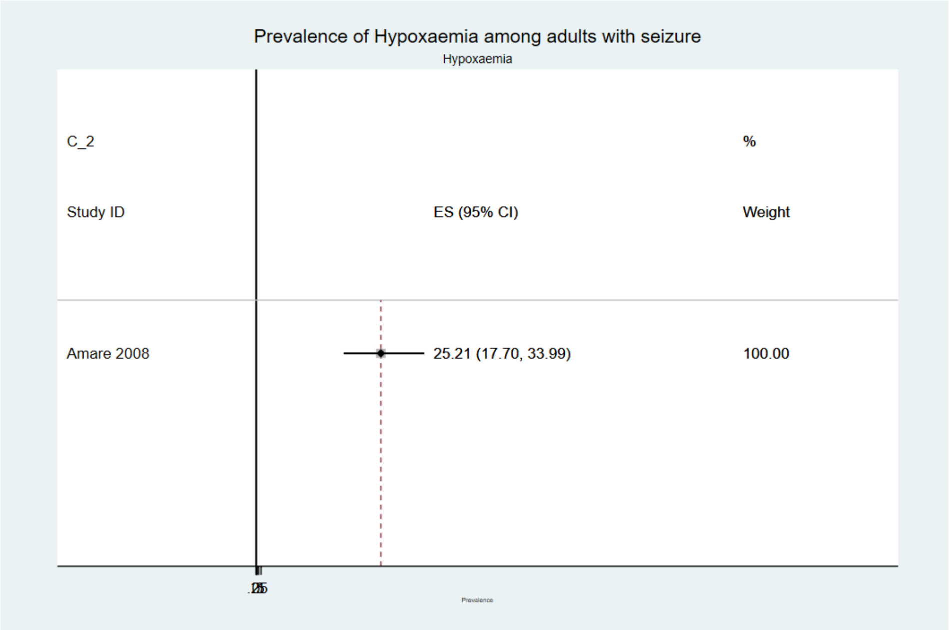

No meta-estimate calculated as only one included studies.

Admitted Adults - sepsis

Table 32: Studies included in prevalence of hypoxaemia among admitted adults with sepsis.

Serial	Study ID	WB Region	Proportion	LCL	UCL	Denominator
1.	Rudd 2014	Sub Saharan Africa	55.00	31.53	76.94	20
2.	Papali 2017	Latin America & Caribbean	24.10	17.81	31.33	166
3.	Boonmee 2020	East Asia & Pacific	27.17	25.01	29.41	1616
4.	MarMinn 2021	East Asia & Pacific	12.18	9.47	15.34	509
**Overall (I2=95.41%, T2=0.06, p<0.05)**			**25.29**	**14.83**	**37.39**	**2311**

Fig. 32: Studies included in prevalence of hypoxaemia among admitted adults with sepsis.

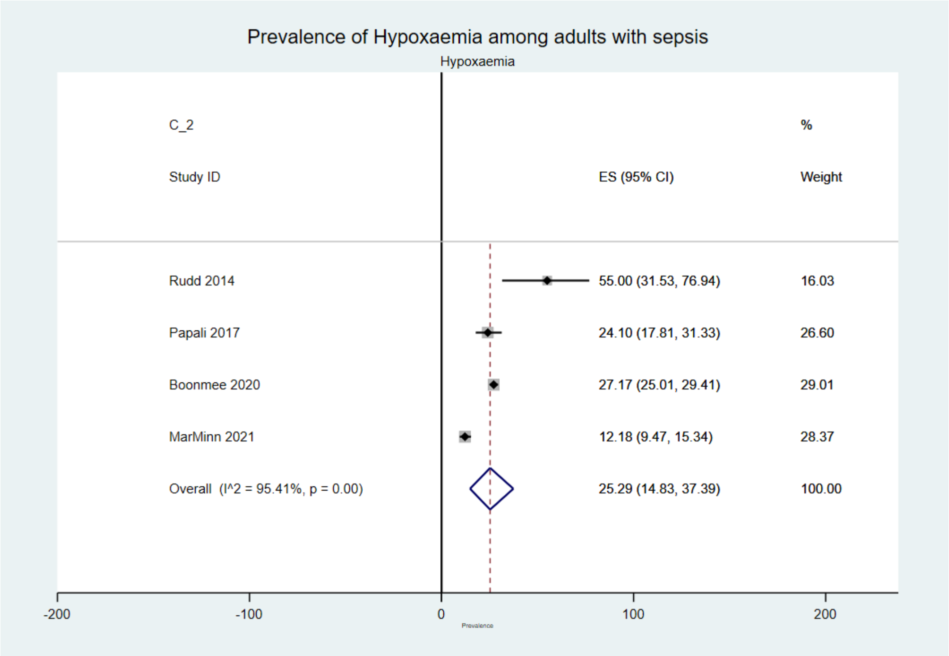

The evidence was assessed as **low certainty** because of imprecision of the estimate, inconsistency in prevalence estimates between studies.

Admitted Adults – trauma / injury

Table 33: Studies included in prevalence of hypoxaemia among admitted adults with trauma.

Serial	Study ID	WB Region	Proportion	LCL	UCL	Denominator
1.	Stassen 2014	Sub Saharan Africa	37.88	26.22	50.66	66

Fig. 33: Studies included in prevalence of hypoxaemia among admitted adults with trauma.

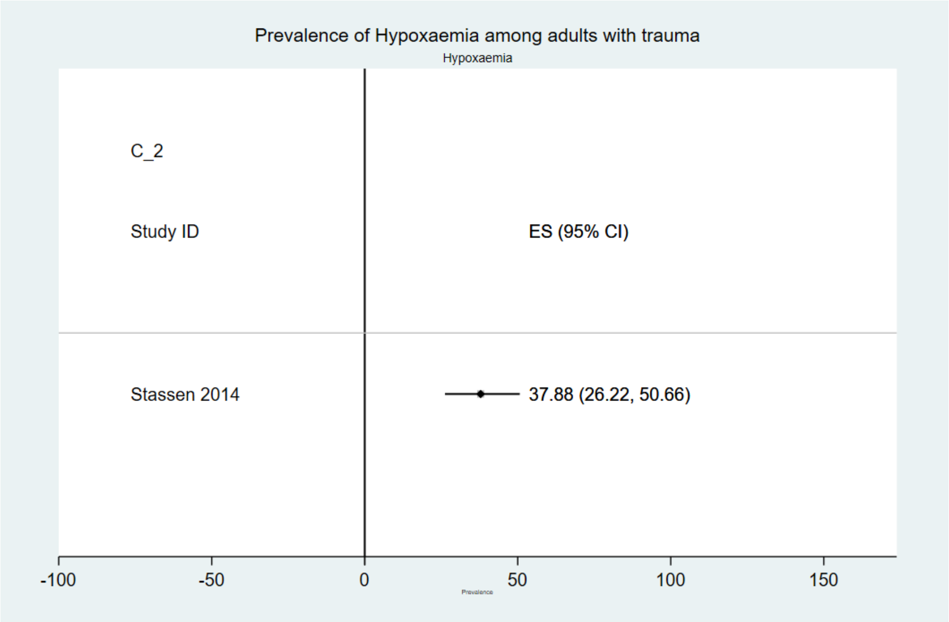

No meta-estimate calculated as only one included studies.

Admitted Adults - tuberculosis

Table 34: Studies included in prevalence of hypoxaemia among admitted adults with tuberculosis.

Serial	Study ID	WB Region	Proportion	LCL	UCL	Denominator
1.	Davis 2010	Sub Saharan Africa	26.39	16.70	38.10	72
2.	Katagira 2016	Sub Saharan Africa	34.23	30.53	38.08	631

Fig. 34: Studies included in prevalence of hypoxaemia among admitted adults with tuberculosis.

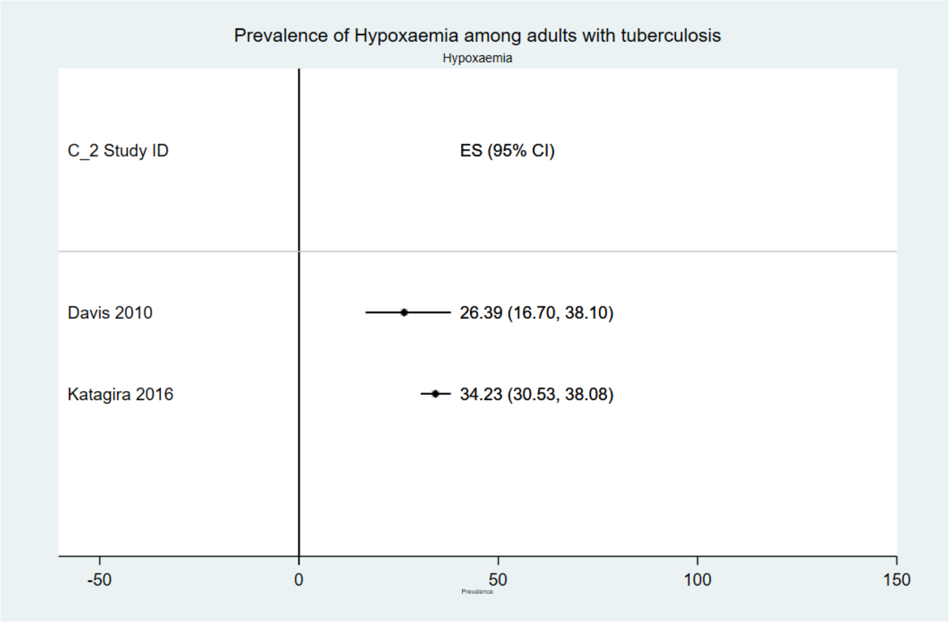

No meta-estimate calculated as only two included studies.

Admitted Adults – COVID-19

Table 35: Studies included in prevalence of hypoxaemia among admitted adults with COVID-19.

Serial	Study ID	WB Region	Proportion	LCL	UCL	Denominator
1.	Li 2020	East Asia & Pacific	3.02	1.11	6.45	199
2.	Mejia 2020	Latin America & Caribbean	64.50	59.38	69.38	369
3.	Wollenstein-Betech 2020	Latin America & Caribbean	55.57	55.28	55.86	113214
4.	Homayounieh 2020	Middle East & North Africa	29.33	19.38	40.98	75
5.	Zhou 2020	East Asia & Pacific	3.25	0.89	8.12	123
6.	Liu 2020	East Asia & Pacific	28.44	20.21	37.88	109
7.	Bepouka 2020	Sub Saharan Africa	67.38	58.98	75.03	141
8.	Nemati 2021	Middle East & North Africa	61.32	58.22	64.36	998
9.	Honarvar 2021	Middle East & North Africa	49.82	46.34	53.30	819
10.	Sarfaraz 2021	South Asia	65.29	57.63	72.42	170
11.	Sultan 2021	Sub Saharan Africa	59.78	49.04	69.88	92
12.	Acar 2021	Europe & Central Asia	41.73	37.97	45.56	671
13.	Diaz-Velez 2021	Latin America & Caribbean	56.39	51.88	60.82	493
14.	Diendere 2021	Sub Saharan Africa	14.48	11.33	18.11	442
15.	Sharawat 2021	South Asia	55.20	52.06	58.31	1000
16.	Marcolino 2021	Latin America & Caribbean	13.39	11.91	14.98	1964
17.	Sampathkumar 2021	South Asia	51.92	37.63	65.99	52
18.	VafadarMoradi 2021	Middle East & North Africa	64.84	58.12	71.15	219
19.	Anyaypoma-Ocon 2021	Latin America & Caribbean	72.89	66.58	78.58	225
20.	Patel 2021	South Asia	29.97	24.72	35.63	287
21.	Padmaprakash 2021	South Asia	5.14	4.09	6.37	1536
22.	Xiong 2021	East Asia & Pacific	88.90	86.82	90.76	1027
23.	Ramatillah 2022	East Asia & Pacific	25.27	16.75	35.47	91

24.	AbdelGhaffar 2022	Middle East & North Africa	28.31	26.87	29.79	3712
25.	deJesus 2022	Latin America & Caribbean	83.63	83.17	84.07	26292
26.	Alizadehsani 2022	Middle East & North Africa	66.67	62.74	70.43	600
27.	KazemiAski 2022	Middle East & North Africa	29.55	16.76	45.20	44
28.	Soto 2022	Latin America & Caribbean	56.84	54.22	59.44	1418
29.	Alva 2022	Latin America & Caribbean	29.89	25.12	35.00	348
30.	Siqueira 2022	Latin America & Caribbean	37.84	37.06	38.61	15105
31.	Tolossa 2022	Sub Saharan Africa	73.58	68.38	78.35	318
32.	Araban 2022	Middle East & North Africa	38.60	36.91	40.32	3181
33.	Sirohiya 2022	South Asia	38.99	36.89	41.12	2080
**Overall (I2=99.84%, T2=0.20, p<0.05)**			**44.15**	**36.56**	**51.88**	**177414**

Fig. 35: Studies included in prevalence of hypoxaemia among admitted adults with COVID-19.

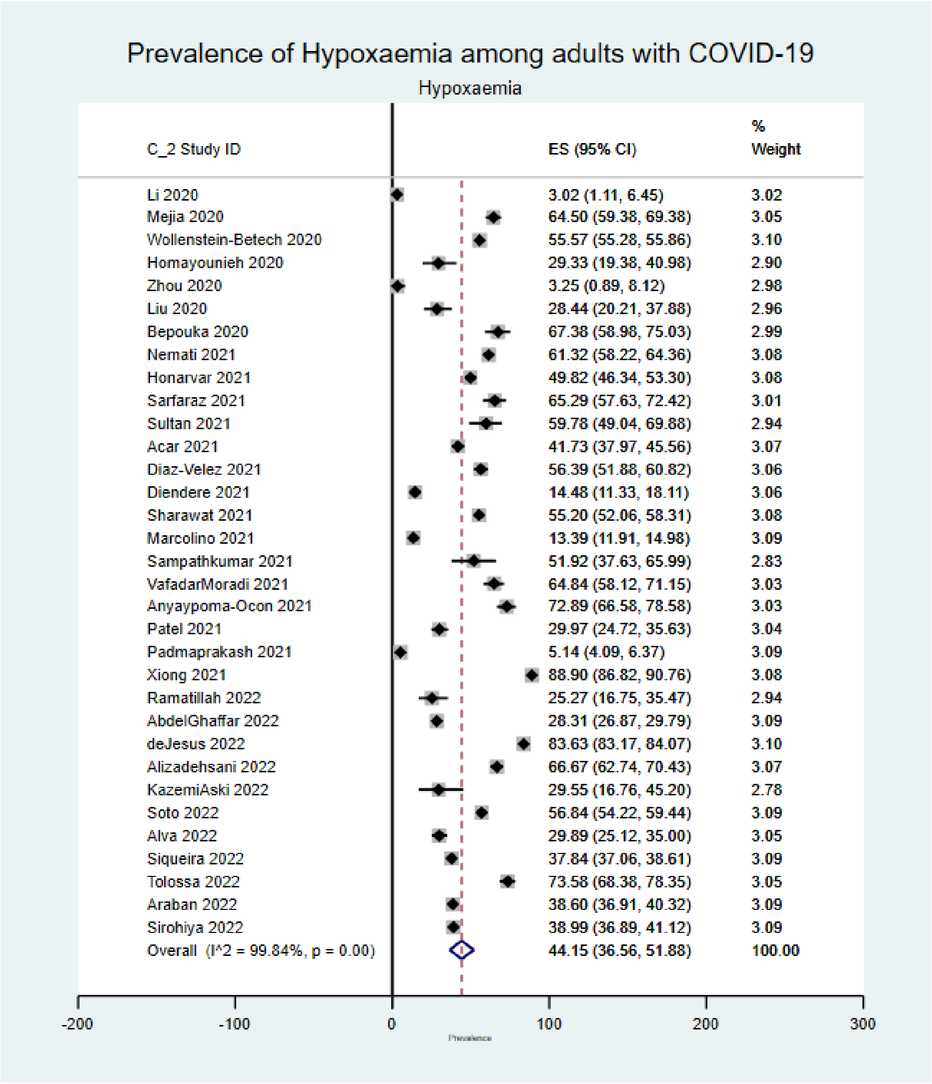

.

The evidence was assessed as **low certainty** because of imprecision of the estimate, inconsistency in prevalence estimates between studies.

Primary Results tables and Forest plots (OPD/ED)

OPD/ED Neonates - all

Table 36: Studies included in prevalence of hypoxaemia among all presenting neonates

Serial	Study ID	WB Region	Proportion	LCL	UCL	Weight	Denominator
1	Rajesh 2000	South Asia	38.50	31.72	45.62	20.95	200
2	Weber 2003	Mixed	7.84	6.95	8.81	21.50	3303
3	Agrawal 2011	South Asia	32.93	22.94	44.19	20.17	82
4	Emdin 2015	South Asia	0.38	0.05	1.36	21.31	528
5	Graham 2021	Sub Saharan Africa	0.00	0.00	20.59	16.07	16
**Overall (I2=98.43%, T2=0.18, p<0.05)**			**11.77**	**2.07**	**27.07**		**4129**

Fig. 36: Studies included in prevalence of hypoxaemia among all presenting neonates

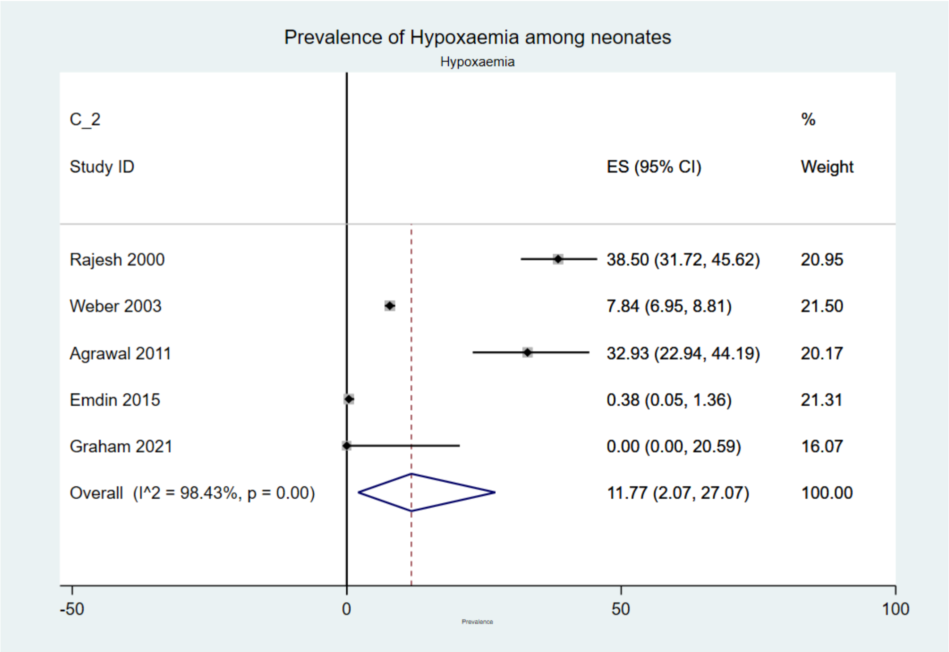

The evidence was assessed as **low certainty** because of imprecision of the estimate, inconsistency in prevalence estimates between studies.

OPD/ED Neonates - pneumonia

Table 37: Studies included in prevalence of hypoxaemia among presenting neonates with pneumonia

No studies

Fig. 37: Studies included in prevalence of hypoxaemia among presenting neonates with pneumonia

No studies

OPD/ED Children - all

Table 38: Studies included in prevalence of hypoxaemia among all presenting children

Serial	Study ID	WB Region	Proportion	LCL	UCL	Weight	Denominator
1	Agrawal 2011	South Asia	43.59	32.39	55.30	20.83	78
2	Garde 2015	South Asia	5.63	4.88	6.46	26.47	3374
3	Blanc 2019	East Asia & Pacific	1.38	0.88	2.07	26.29	1663
4	Graham 2021	Sub Saharan Africa	0.97	0.62	1.44	26.41	2480
**Overall (I2=98.68%, T2=0.05, p<0.05)**			**7.04**	**2.48**	**13.60**		**7595**

Table 38: Studies included in prevalence of hypoxaemia among all presenting children

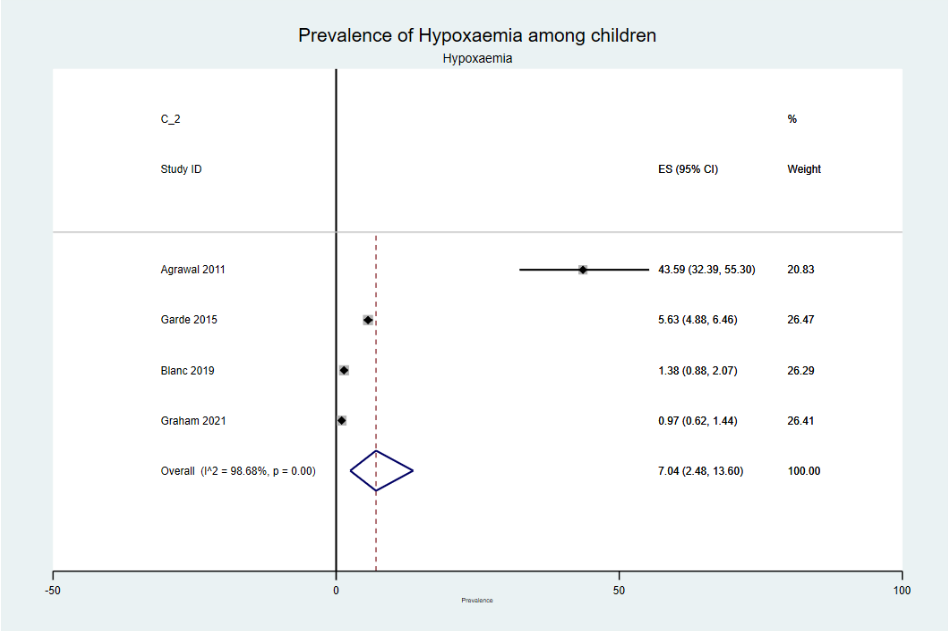

The evidence was assessed as **low certainty** because of imprecision of the estimate, inconsistency in prevalence estimates between studies.

OPD/ED Children - fever

Table 39: Studies included in prevalence of hypoxaemia among presenting children with fever

Serial	Study ID	WB Region	Proportion	LCL	UCL	Weight	Denominator
1	Salah 2014	Sub Saharan Africa	0.67	0.02	3.66	7.58	150
2	Graham 2021	Sub Saharan Africa	0.98	0.58	1.55	92.42	1835

Fig. 39: Studies included in prevalence of hypoxaemia among presenting children with fever

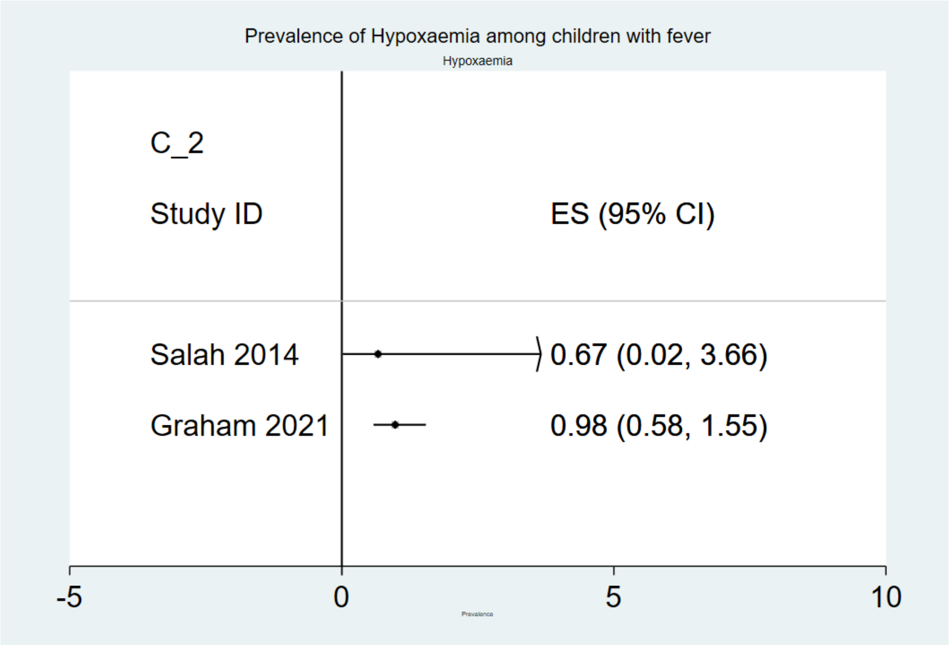

No meta-estimate calculated as only two included studies.

OPD/ED Children – pneumonia all WHO-classified

Table 40: Studies included in prevalence of hypoxaemia among presenting all children with pneumonia (WHO-classification)

Sl no	Study ID	WB Region	Prop	LCL	UCL	Weight	Denom
1.	Lodha 2004	South Asia	25.69	17.80	34.94	8.09	109
2.	Basnet 2006	South Asia	38.67	30.84	46.95	8.20	150
3.	Al Janabi 2009	Middle East & North Africa	6.73	2.75	13.38	8.07	104
4.	Mathisen 2010	South Asia	1.89	1.37	2.55	8.50	2219
5.	Smith 2011	Latin America & Caribbean	26.17	22.86	29.70	8.45	661
6.	Rao 2012	South Asia	36.99	29.15	45.36	8.20	146
7.	Thomas 2015	East Asia & Pacific	7.42	6.50	8.43	8.51	2951
8.	McCollum 2016	Sub Saharan Africa	4.91	4.55	5.30	8.52	13266
9.	Tesfaye 2020	Sub Saharan Africa	14.55	12.34	16.98	8.47	928
10	Pukai 2020	East Asia & Pacific	91.46	86.67	94.94	8.28	199
11	Shrestha 2020	South Asia	58.13	50.08	65.87	8.22	160
12	Bills 2020	South Asia	32.40	29.72	35.16	8.48	1173
Overall (I2=99.52%, T2=0.17, p<0.05)			25.86	16.26	36.80		22066

Fig. 40: Studies included in prevalence of hypoxaemia among all presenting children with pneumonia (WHO-classification)

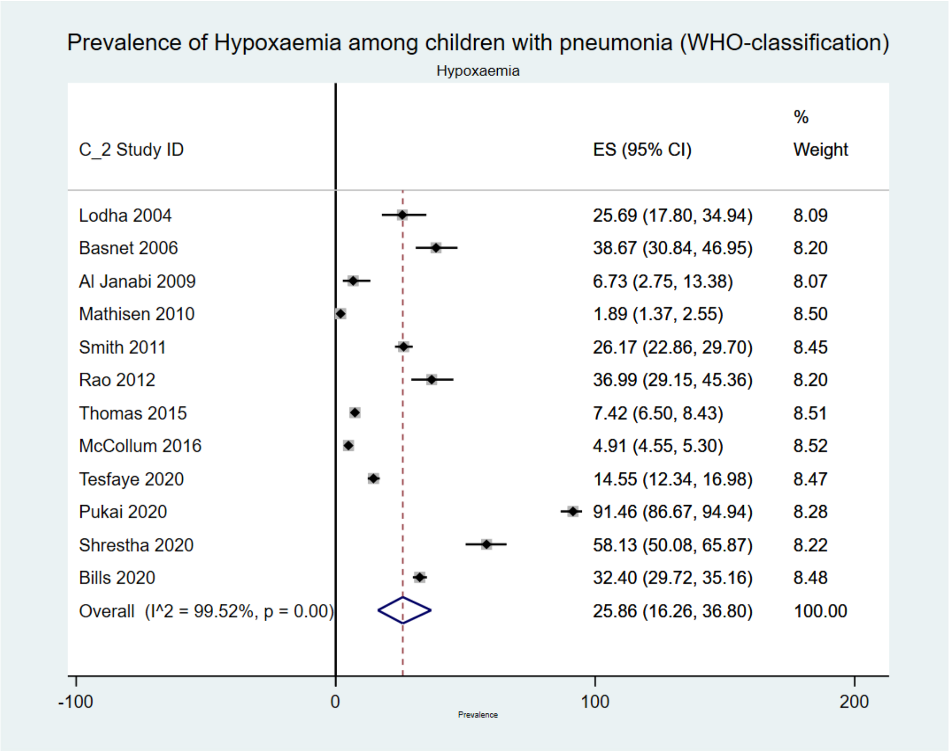

The evidence was assessed as **low certainty** because of imprecision of the estimate, inconsistency in prevalence estimates between studies.

OPD/ED Children – pneumonia severe (WHO-classified severe or very severe)

Table 41: Studies included in prevalence of hypoxaemia among presenting children with severe pneumonia (WHO-severe)

Sl no	Study ID	WB Region	Proportion	LCL	UCL	Weight	Denominator
1.	Basnet 2006	South Asia	88.89	75.95	96.29	32.51	45
2.	Mathisen 2010	South Asia	26.72	19.37	35.15	33.84	131
3.	Rao 2012	South Asia	48.08	38.17	58.09	33.65	104
Overall (I2=96.77%, T2=0.34, p<0.05)			55.36	22.66	85.69		280

Fig. 41: Studies included in prevalence of hypoxaemia among presenting children with severe pneumonia (WHO-severe)

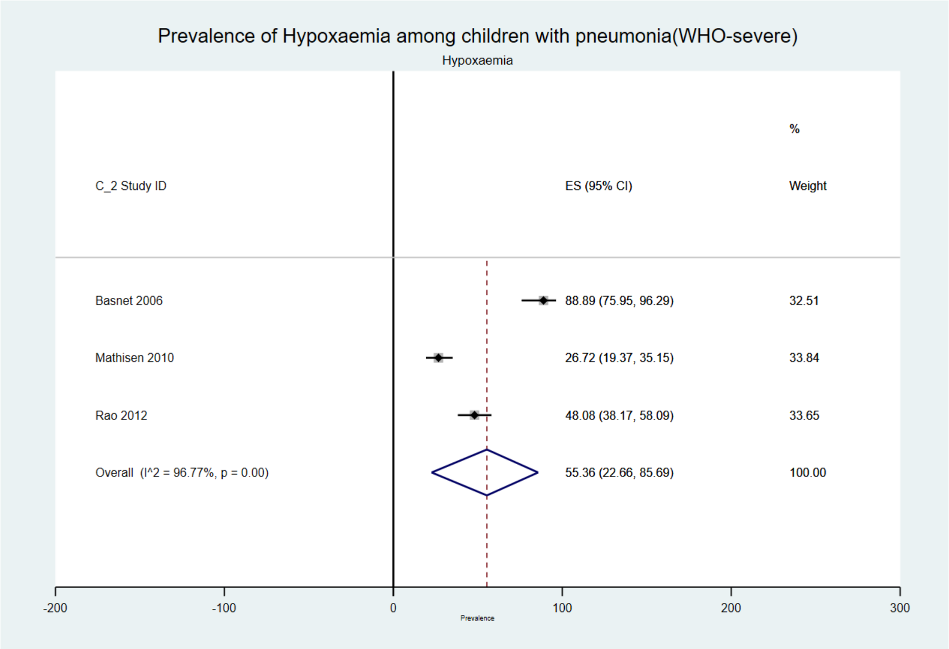

The evidence was assessed as **low certainty** because of imprecision of the estimate, inconsistency in prevalence estimates between studies.

OPD/ED Children – pneumonia non-severe (WHO-classified)

Table 42: Studies included in prevalence of hypoxaemia among presenting children with non-severe pneumonia (WHO-non severe)

Serial	Study ID	WB Region	Proportion	LCL	UCL	Weight	Denominator
1	Basnet 2006	South Asia	17.14	10.49	25.73	33.52	105
2	Mathisen 2010	South Asia	0.34	0.13	0.69	34.84	2088
3	Rao 2012	South Asia	9.52	2.66	22.62	31.64	42
**Overall (I2=96.96%, T2=0.23, p<0.05)**			**6.61**	**0.00**	**26.68**		**2235**

Fig. 42: Studies included in prevalence of hypoxaemia among presenting children with non-severe pneumonia (WHO-non severe)

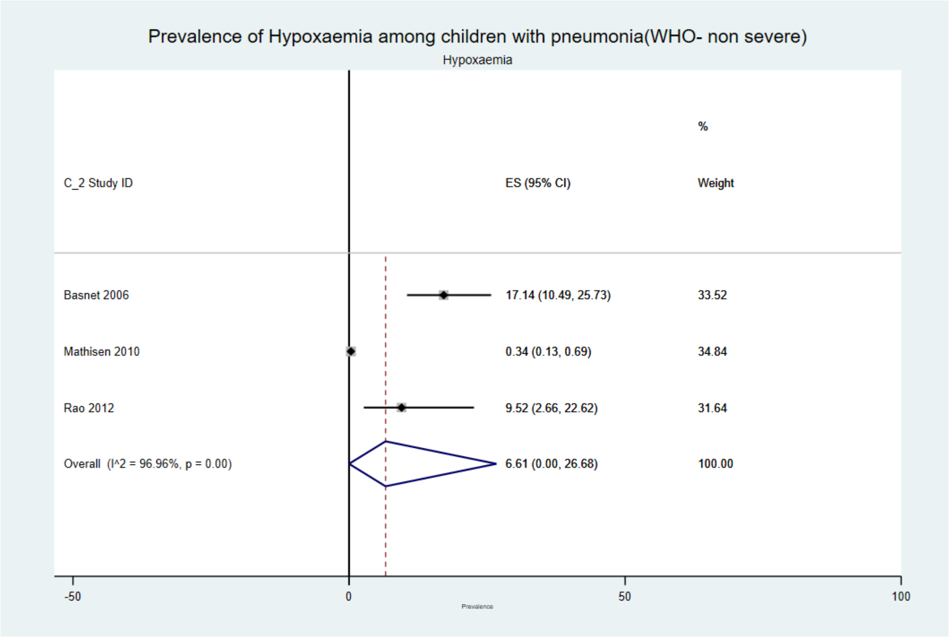

The evidence was assessed as **low certainty** because of imprecision of the estimate, inconsistency in prevalence estimates between studies.

OPD/ED Children – pneumonia radiological

Table 43: Studies included in prevalence of hypoxaemia among presenting children with pneumonia (radiological)

Serial	Study ID	WB Region	Proportion	LCL	UCL	Weight	Denominator
1	Shrestha 2020	South Asia	67.01	56.73	76.22	100	97

Fig. 43: Studies included in prevalence of hypoxaemia among presenting children with pneumonia (radiological)

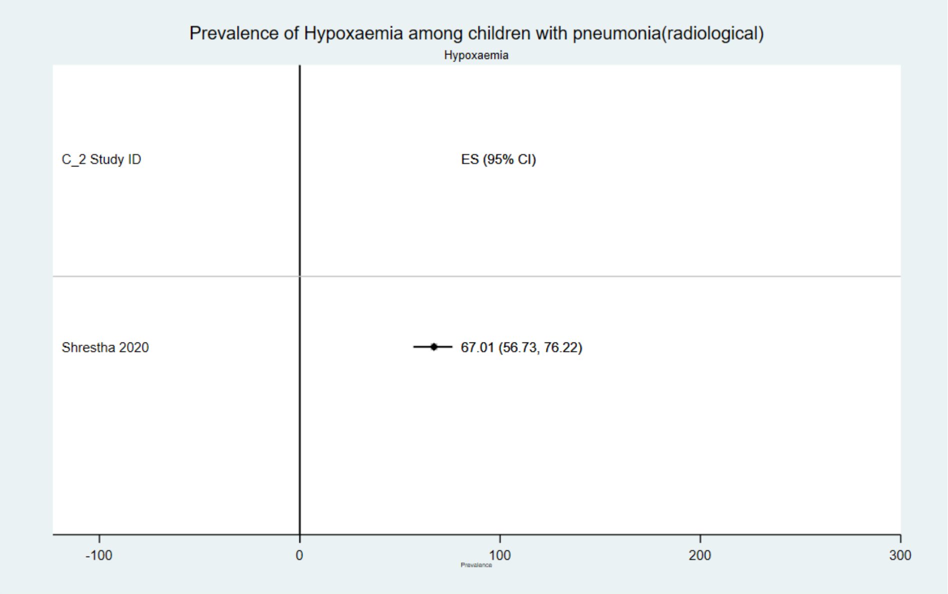

No meta-estimate calculated as only one included study.

OPD/ED Children - bronchiolitis

Table 44: Studies included in prevalence of hypoxaemia among presenting children with bronchiolitis

Serial	Study ID	WB Region	Proportion	LCL	UCL	Weight	Denominator
1	Jeena 2003	Sub Saharan Africa	11.40	6.21	18.71	34.61	114
2	Rao 2012	South Asia	8.89	3.92	16.77	33.94	90
3	Yahia 2012	Middle East & North Africa	39.58	25.77	54.73	31.45	48
**Overall (I2=89.77%, T2=0.11, p<0.05)**			**17.80**	**5.20**	**35.41**		**252**

Fig. 44: Studies included in prevalence of hypoxaemia among presenting children with bronchiolitis

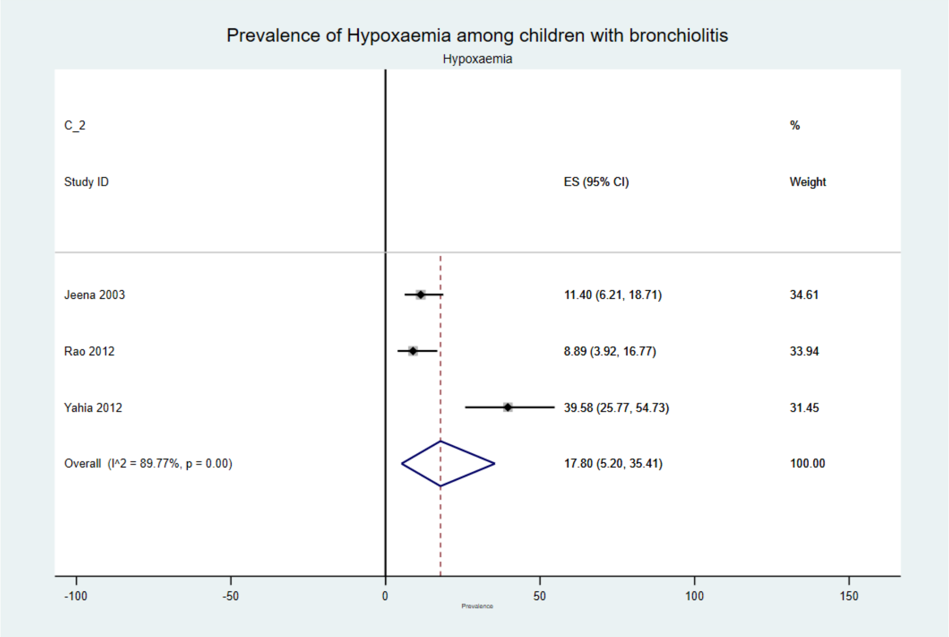

The evidence was assessed as **very low certainty** because of moderate risk of bias (variable representativeness of population / variable validity of diagnostic assessment), imprecision of the estimate, inconsistency in prevalence estimates between studies.

OPD/ED Children – pneumonia unspecified

Table 45: Studies included in prevalence of hypoxaemia among presenting children with pneumonia (unspecified)

Serial	Study ID	WB Region	Proportion	LCL	UCL	Weight	Denominator
1	Graham 2021	Sub Saharan Africa	5.63	2.46	10.80	17.46	142
2	vonderWeid 2018	Sub Saharan Africa	15.91	6.64	30.07	14.70	44
3	Rao 2012	South Asia	23.75	18.72	29.39	18.17	261
4	McCracken 2013	Latin America & Caribbean	29.22	26.71	31.83	18.89	1249
5	Yahia 2012	Middle East & North Africa	39.58	25.77	54.73	14.99	48
6	Sylvies 2020	Sub Saharan Africa	46.03	33.39	59.06	15.79	63
**Overall (I2=93.08%, T2=0.08, p<0.05)**			**24.88**	**15.35**	**35.79**		**1807**

Fig. 45: Table 45: Studies included in prevalence of hypoxaemia among presenting children with pneumonia (unspecified)

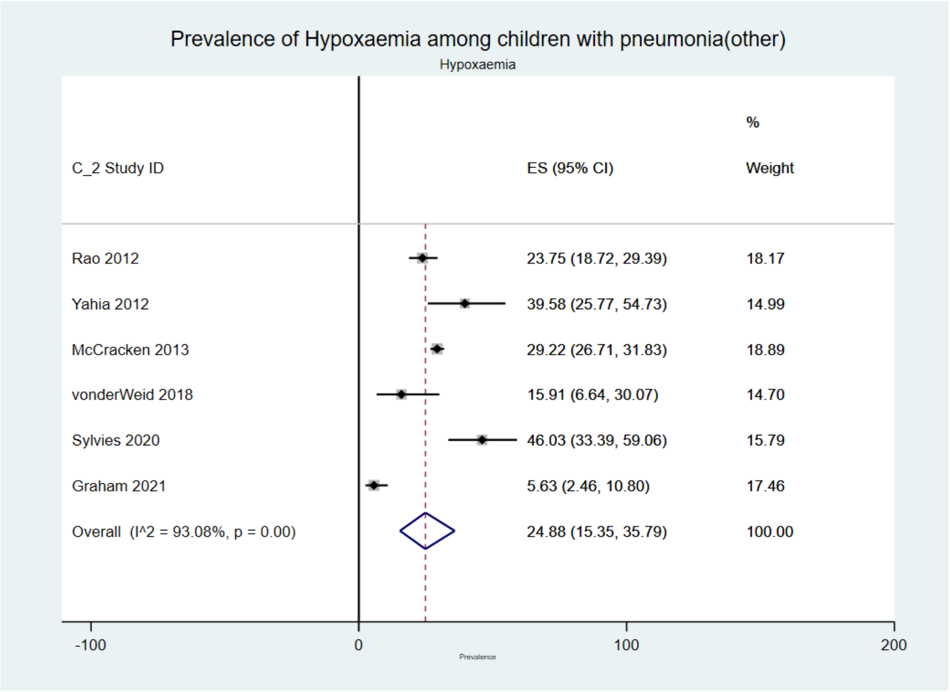

The evidence was assessed as **very low certainty** because of moderate risk of bias (variable representativeness of population), imprecision of the estimate, inconsistency in prevalence estimates between studies.

OPD/ED Children - anaemia

Table 46: Table 45: Studies included in prevalence of hypoxaemia among presenting children with anaemia (unspecified)

Serial	Study ID	WB Region	Proportion	LCL	UCL	Weight	Denominator
1	Chinawa 2013	Sub Saharan Africa	18.18	10.76	27.84	100.00	88

Fig. 46: Studies included in prevalence of hypoxaemia among presenting children with anaemia (unspecified)

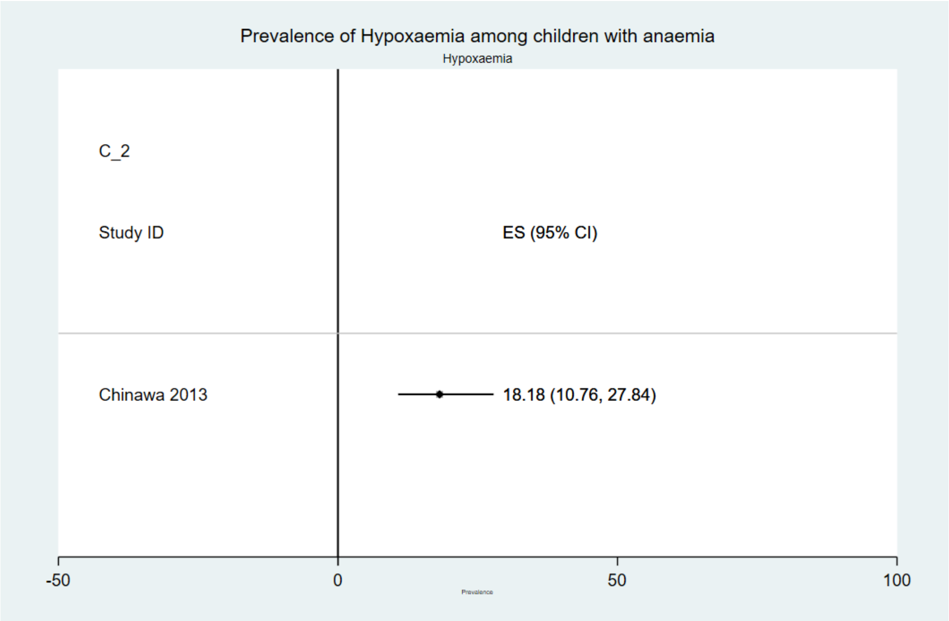

No meta-estimate calculated as only one included study.

OPD/ED Children - asthma

Table 47: Studies included in prevalence of hypoxaemia among presenting children with asthma

Serial	Study ID	WB Region	Proportion	LCL	UCL	Weight	Denominator
1	vonderWeid 2018	Sub Saharan Africa	9.76	4.31	18.32	30.33	82
2	Onubogu 2022	Sub Saharan Africa	3.17	1.17	6.78	69.67	189

Fig. 47: Studies included in prevalence of hypoxaemia among presenting children with asthma

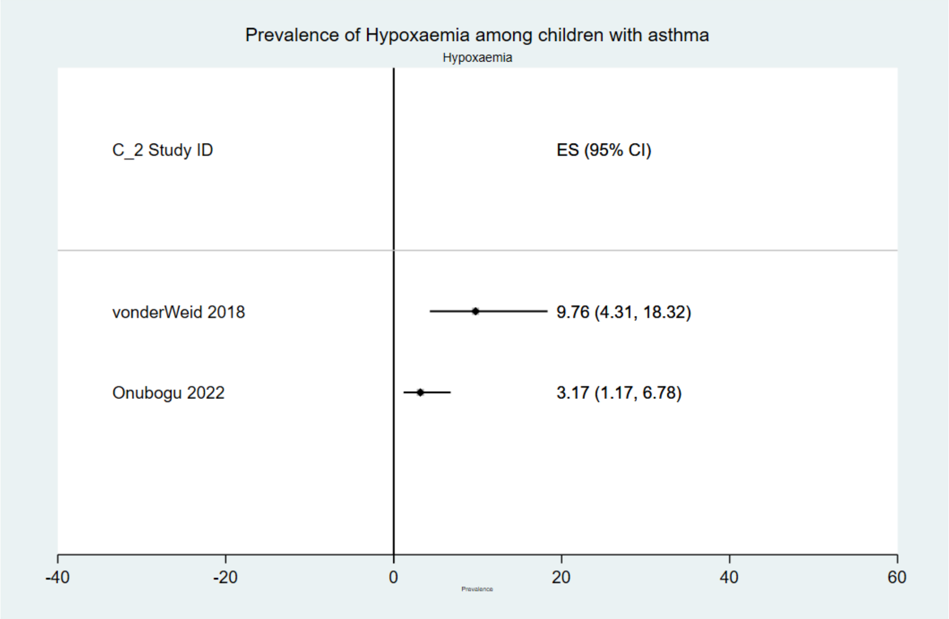

No meta-estimate calculated as only two included studies.

OPD/ED Children - malaria

Table 48: Studies included in prevalence of hypoxaemia among presenting children with malaria

Serial	Study ID	WB Region	Proportion	LCL	UCL	Weight	Denominator
1	Boyce 2017	Sub Saharan Africa	9.70	8.11	11.47	47.80	1248
2	Graham 2021	Sub Saharan Africa	0.81	0.40	1.44	52.20	1363

Fig. 48: Studies included in prevalence of hypoxaemia among presenting children with malaria

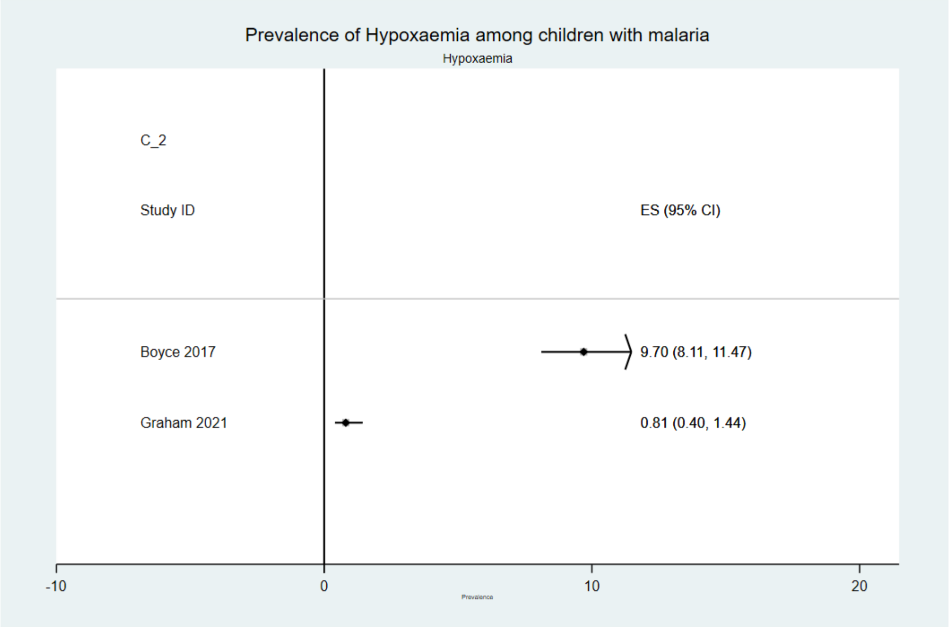

No meta-estimate calculated as only two included studies.

OPD/ED Children - sepsis

Table 49: Studies included in prevalence of hypoxaemia among presenting children with sepsis

Serial	Study ID	WB Region	Proportion	LCL	UCL	Weight	Denominator
1	Graham 2021	Sub Saharan Africa	0.00	0.00	6.49	100.00	55

Fig. 49: Studies included in prevalence of hypoxaemia among presenting children with sepsis

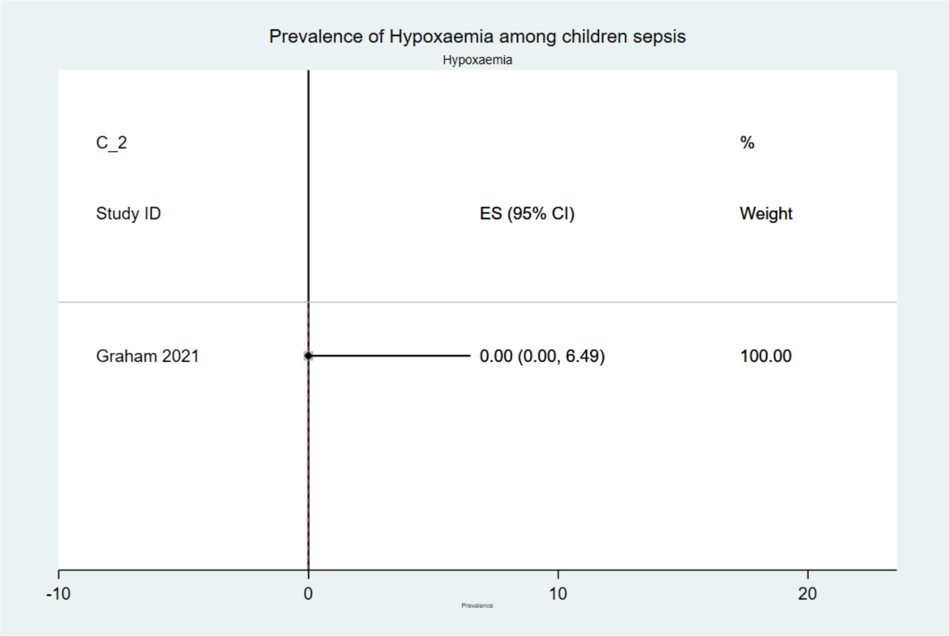

No meta-estimate calculated as only one included study.

OPD/ED Children - diarrhoea

Table 50: Studies included in prevalence of hypoxaemia among presenting children with diarrhoea

Serial	Study ID	WB Region	Proportion	LCL	UCL	Weight	Denominator
1	Graham 2021	Sub Saharan Africa	1	0.21	2.89	100	300

Fig. 50: Studies included in prevalence of hypoxaemia among presenting children with diarrhoea

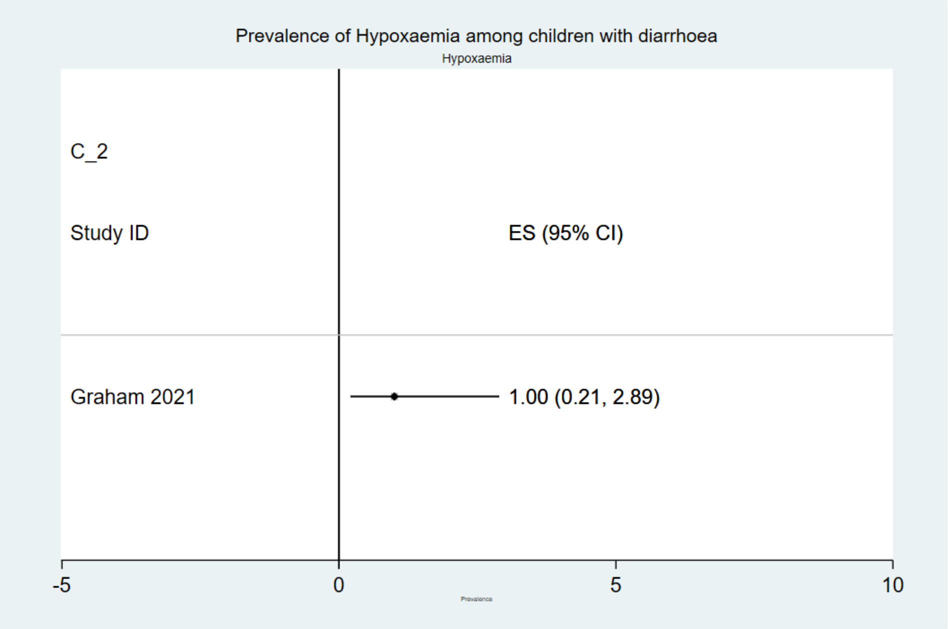

No meta-estimate calculated as only one included study.

OPD/ED Adults - all

Table 51: Studies included in prevalence of hypoxaemia among all presenting adults

Serial	Study ID	WB Region	Proportion	LCL	UCL	Weight	Denominator
1	Sutherland 2019	Sub Saharan Africa	12.09723	10.61318	13.70823	33.23456	1769
2	Kwizera 2020	Sub Saharan Africa	4.479452	4.016355	4.979323	33.4189	7300
3	Graham 2021	Sub Saharan Africa	0.091352	0.018843	0.266735	33.34655	3284
**Overall (I2=99.63%, T2=0.08, p<0.05)**			**3.95**	**0.17**	**12.25**		**12353**

Fig. 51: Studies included in prevalence of hypoxaemia among all presenting adults

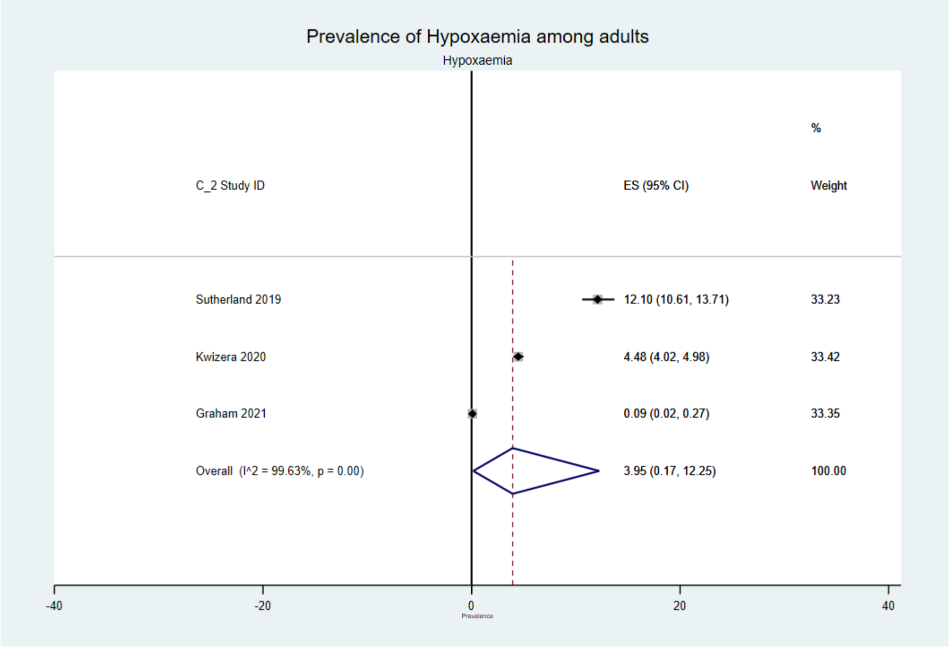

The evidence was assessed as **low certainty** because of imprecision of the estimate, inconsistency in prevalence estimates between studies.

OPD/ED Adults - pneumonia

Table 52: Studies included in prevalence of hypoxaemia among all presenting adults with pneumonia

Serial	Study ID	WB Region	Proportion	LCL	UCL	Weight	Denominator
1	McCracken 2013	Latin America & Caribbean	35.71	25.55	46.92	25.61	84
2	Hu 2020	East Asia & Pacific	4.70	2.85	7.25	26.48	404
3	Friedman 2020	Latin America & Caribbean	34.03	30.57	37.62	26.58	720
4	Graham 2021	Sub Saharan Africa	0.00	0.00	23.16	21.33	14
**Overall (I2=98.36%, T2=0.27, p<0.05)**			**15.65**	**1.37**	**39.43**		**1222**

Fig. 52: Studies included in prevalence of among all presenting adults with pneumonia

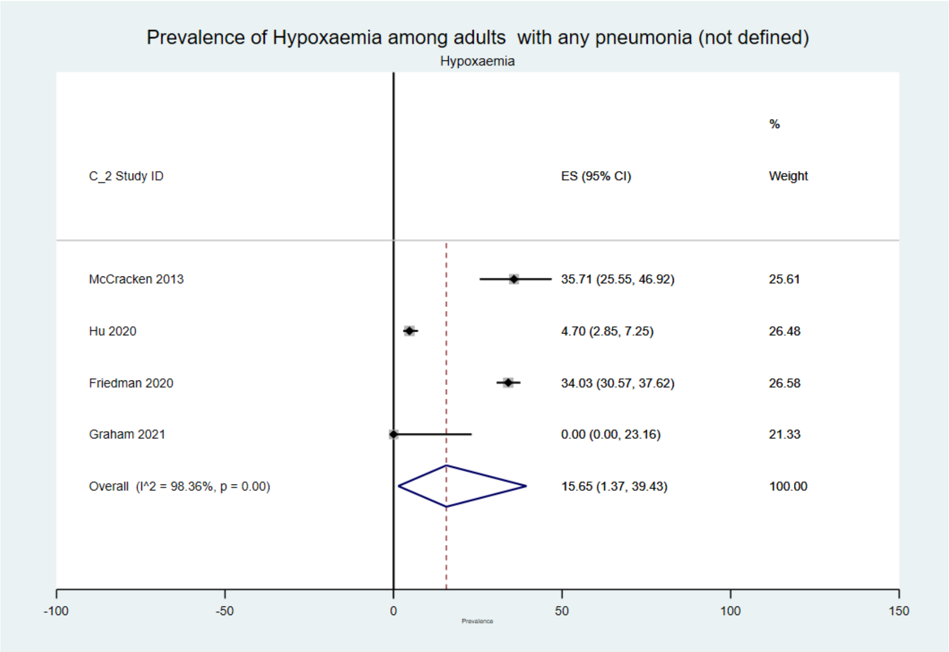

The evidence was assessed as **low certainty** because of imprecision of the estimate, inconsistency in prevalence estimates between studies.

OPD/ED Adults – pneumonia radiological

Table 53: Studies included in prevalence of hypoxaemia among presenting adults with pneumonia (radiological)

Serial no	Study ID	WB Region	Proportion	LCL	UCL	Weight	Denominator
1	Hu 2020	East Asia & Pacific	4.70	2.85	7.25	100.00	404

Fig. 53: Studies included in prevalence of hypoxaemia among presenting adults with pneumonia (radiological)

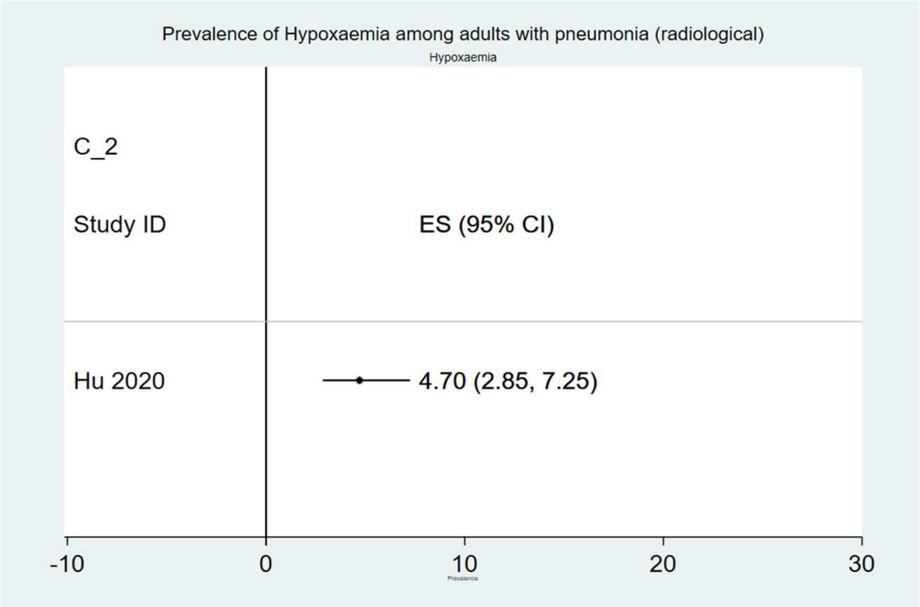

No meta-estimate calculated as only one included study.

OPD/ED Adults – HIV complications

Table 54: Studies included in prevalence of hypoxaemia among presenting adults with HIV complications

Serial no	Study ID	WB Region	Proportion	LCL	UCL	Weight	Denominator
1	Laher 2022	Sub Saharan Africa	16.01	14.00	18.19	100.00	1224

Fig. 54: Studies included in prevalence of hypoxaemia among presenting adults with HIV complications

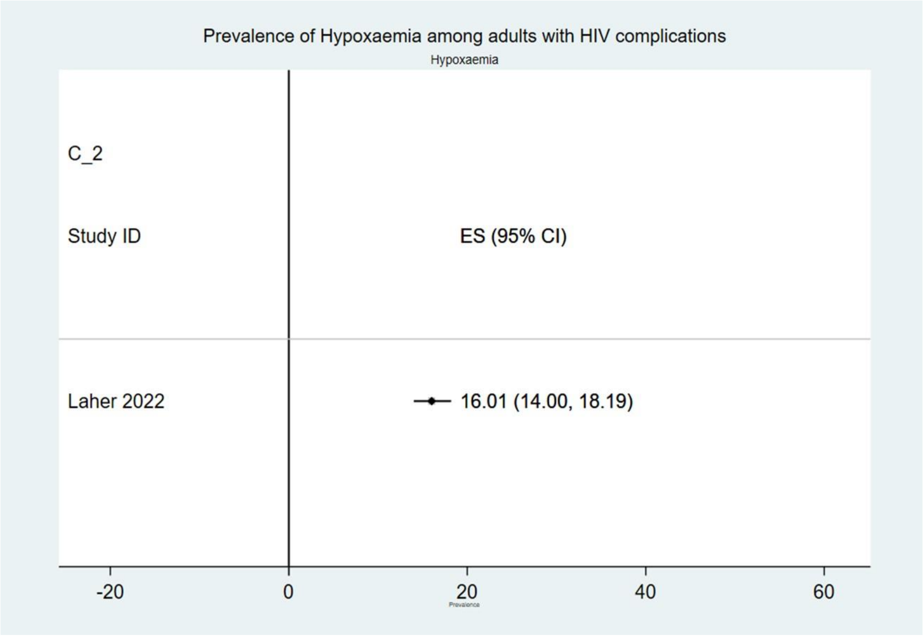

No meta-estimate calculated as only one included study.

OPD/ED Adults – malaria

Table 55: Studies included in prevalence of hypoxaemia among presenting adults with malaria

Serial no	Study ID	WB Region	Proportion	LCL	UCL	Weight	Denominator
1	Graham 2021	Sub Saharan Africa	0.10	0.00	0.58	100	965

Fig. 55: Studies included in prevalence of hypoxaemia among presenting adults with malaria

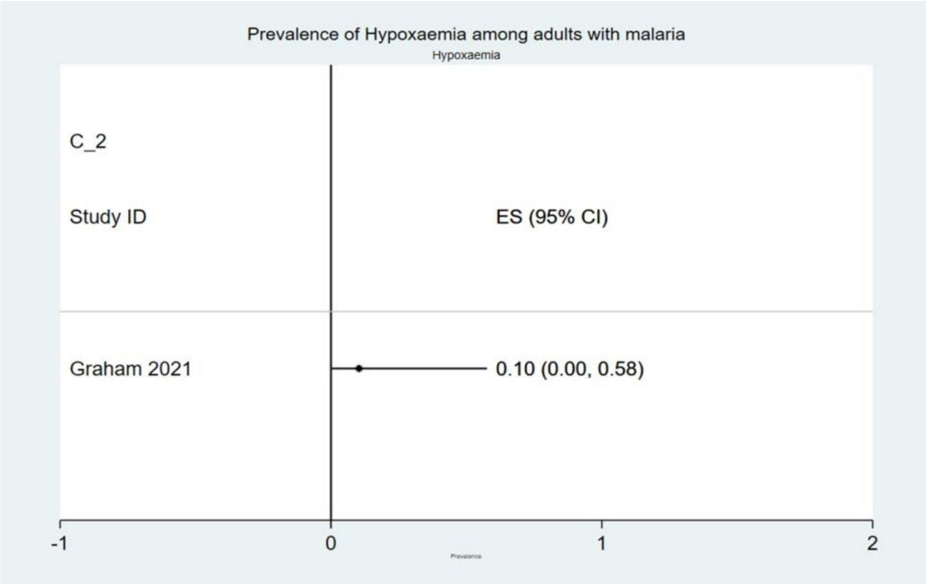

No meta-estimate calculated as only one included study.

OPD/ED Adults – sepsis

Table 56: Studies included in prevalence of hypoxaemia among presenting adults with sepsis

Serial no	Study ID	WB Region	Proportion	LCL	UCL	Weight	Denominator
1	Graham 2021	Sub Saharan Africa	0.00	0.00	6.38	100.00	56

Fig. 56: Studies included in prevalence of hypoxaemia among presenting adults with sepsis

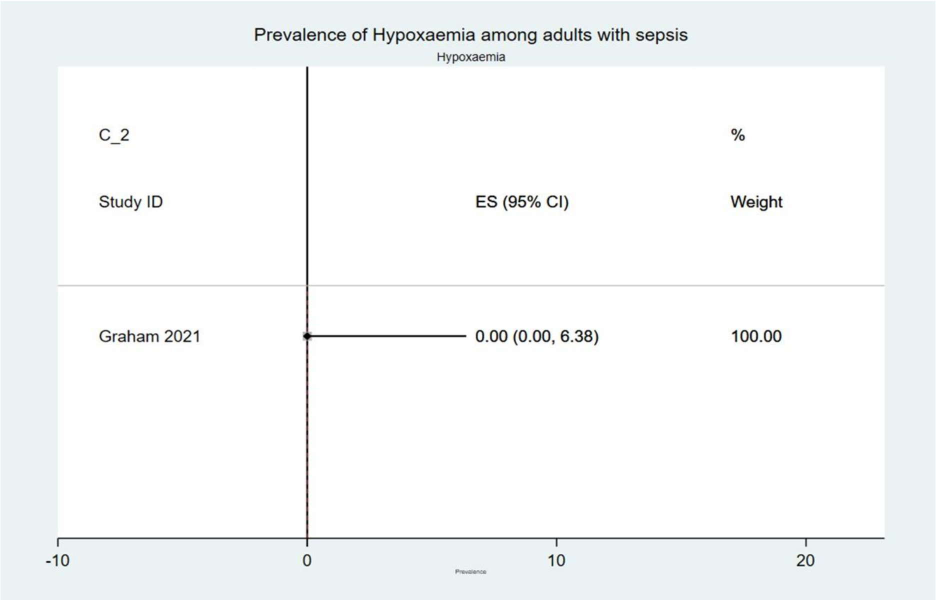

No meta-estimate calculated as only one included study.

OPD/ED Adults – trauma / injury

Table 57: Studies included in prevalence of hypoxaemia among presenting adults with trauma

Serial no	Study ID	WB Region	Proportion	LCL	UCL	Weight	Denominator
1	Krebs 2017	Sub Saharan Africa	3.43	2.19	5.11	34.03	670
2	Landes 2017	Sub Saharan Africa	20.59	15.26	26.79	32.16	204
3	Karim 2021	Sub Saharan Africa	5.62	3.82	7.92	33.81	534
**Overall (I2=96.08%, T2=0.06, p<0.05)**			**8.48**	**2.39**	**17.74**		**1408**

Fig. 57: Studies included in prevalence of hypoxaemia among presenting adults with trauma

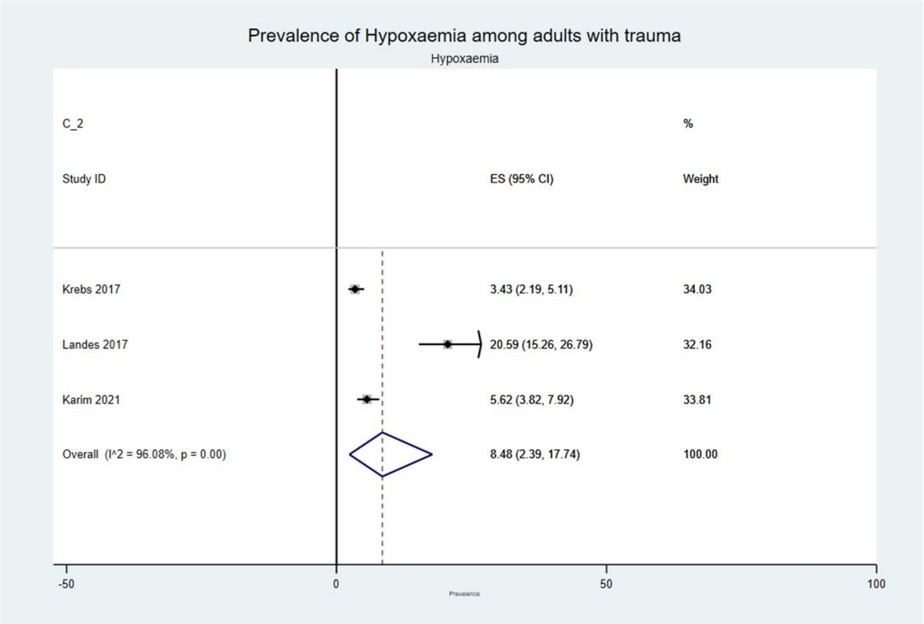

The evidence was assessed as **low certainty** because of imprecision of the estimate, inconsistency in prevalence estimates between studies.

Subgroup Results tables and Forest plots (Tertiary vs Secondary level facilities)

Tertiary Admitted Neonates

Table 58: Studies included in prevalence of hypoxaemia among all neonates in tertiary settings

Serial no	Study ID	WB Region	Proportion	LCL	UCL	Weight	Denominator
1	Junge 2006	Sub Saharan Africa	16.45	12.50	21.06	24.78	310
2	Orimadegun 2013	Sub Saharan Africa	41.41	36.84	46.09	25.20	454
3	Morgan 2018	Sub Saharan Africa	29.24	24.86	33.92	25.09	407
4	Kiputa 2022	Sub Saharan Africa	21.26	17.08	25.94	24.92	348
**Overall (I2=95.65%, T2=0.06, p<0.05)**			**26.66**	**16.71**	**37.97**		**1519**

Fig. 58: Studies included in prevalence of hypoxaemia among all neonates in tertiary settings

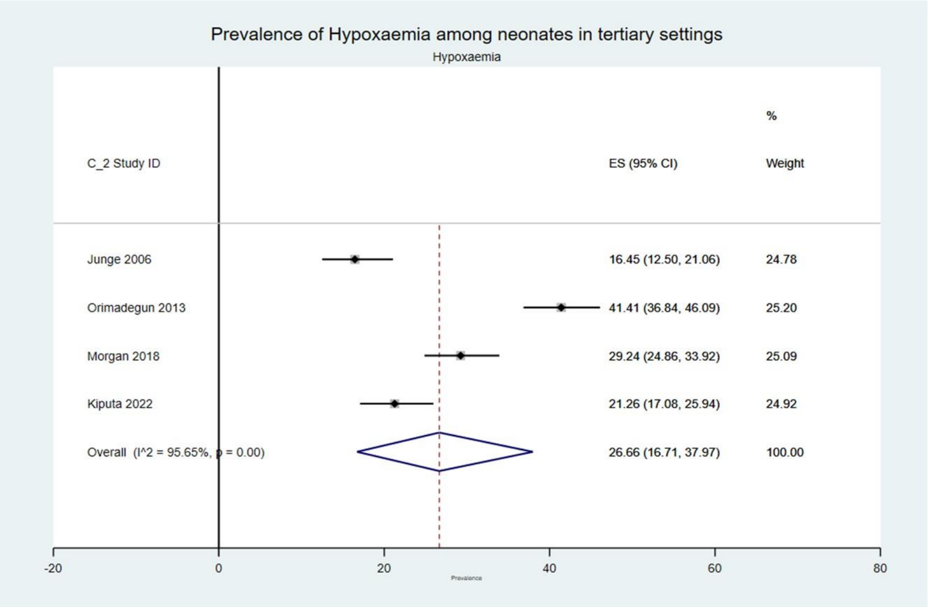

The evidence was assessed as **low certainty** because of imprecision of the estimate, inconsistency in prevalence estimates between studies.

Tertiary Admitted Children

Table 59: Studies included in prevalence of hypoxaemia all children in tertiary setting.

Serial	Study ID	WB Region	Proportion	LCL	UCL	Weight	Denominator
1	Junge 2006	Sub Saharan Africa	5.75	4.98	6.60	14.47	3269
2	Orimadegun 2013	Sub Saharan Africa	24.06	21.73	26.50	14.38	1272
3	Barennes 2016	East Asia & Pacific	13.71	10.29	17.77	14.01	350
4	Lowlaavar 2016	Sub Saharan Africa	25.72	23.35	28.19	14.38	1291
5	Hau 2018	Sub Saharan Africa	5.59	3.80	7.88	14.19	537
6	Nielsen 2018	Latin America & Caribbean	8.58	6.91	10.50	14.34	991
7	Krithika 2022	South Asia	8.17	6.10	10.65	14.22	600
**Overall (I2=98.81%, T2=0.08, p<0.05)**			**12.16**	**6.21**	**19.74**		**8310**

Fig. 59: Studies included in prevalence of hypoxaemia all children in tertiary setting.

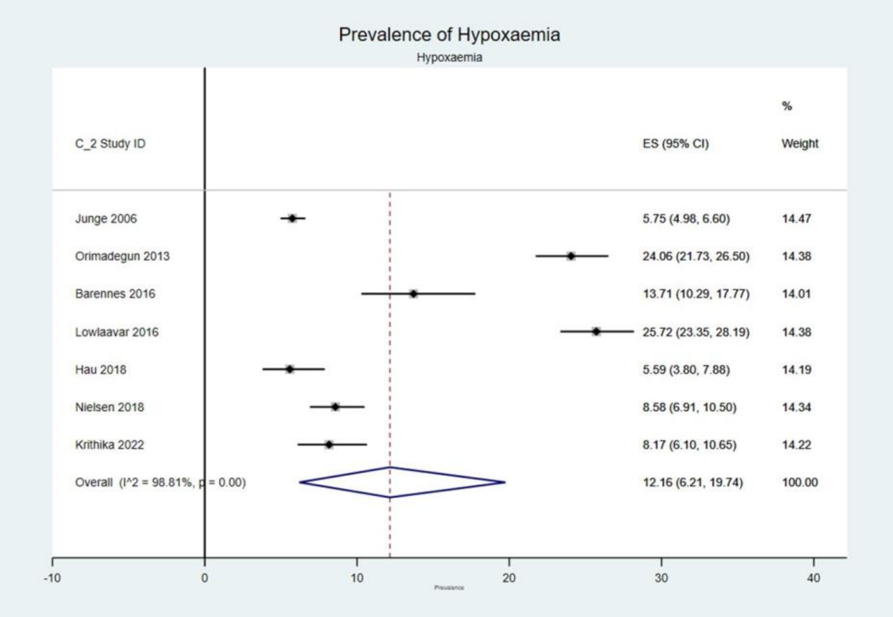

The evidence was assessed as **low certainty** because of imprecision of the estimate, inconsistency in prevalence estimates between studies.

Tertiary Admitted Adults

Table 60: Studies included in prevalence of hypoxaemia among all adults in tertiary setting.

Serial	Study ID	WB Region	Proportion	LCL	UCL	Weight	Denominator
1.	Evans 2012	Sub Saharan Africa	9.72	5.42	15.77	22.80	144
2.	Riviello 2016	Sub Saharan Africa	12.05	10.13	14.17	26.07	1046
3.	Aslam 2021	South Asia	15.17	11.25	19.83	24.60	290
4.	Navuluri 2023	Sub Saharan Africa	23.76	22.46	25.09	26.53	4104
**Overall (I2=96.96%, T2=0.04, p<0.05)**			**15.05**	**8.49**	**23.06**		**5584**

Fig. 60: Studies included in prevalence of hypoxaemia among all adults in tertiary setting.

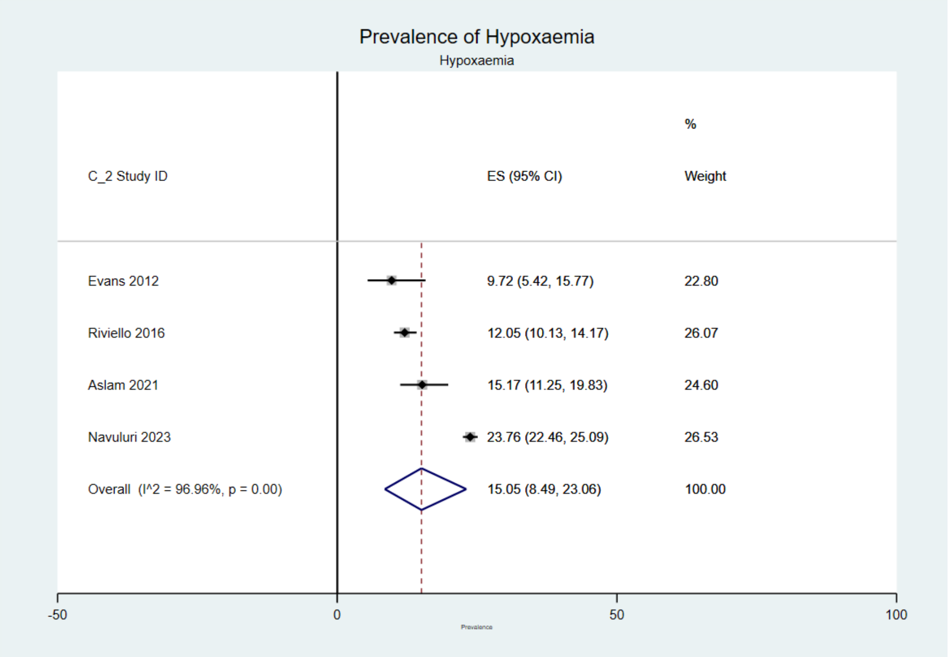

The evidence was assessed as **low certainty** because of imprecision of the estimate, inconsistency in prevalence estimates between studies.

Tertiary Admitted Children – pneumonia all WHO-classified

Table 61: Studies included in prevalence of hypoxaemia all children with WHO pneumonia in tertiary

Sl no	Study ID	WB Region	Proportion	LCL	UCL	Weight	Denominator
1.	West 1999	Sub Saharan Africa	43.68	36.52	51.05	2.76	190
2.	Addo-Yobo 2004	Mixed	19.10	17.25	21.04	2.85	1702
3.	Laman 2005	East Asia & Pacific	25.97	16.64	37.23	2.61	77
4.	Fu 2006	Mixed	19.30	17.45	21.26	2.85	1694
5.	Junge 2006	Sub Saharan Africa	11.70	8.83	15.09	2.82	436
6.	Puumalainen 2008	East Asia & Pacific	16.16	14.08	18.42	2.85	1151
7.	Asghar 2008	Mixed	64.61	61.49	67.65	2.84	958
8.	Nantanda 2008	Sub Saharan Africa	39.49	31.79	47.59	2.73	157
9.	Sigauque 2009	Sub Saharan Africa	25.84	22.60	29.29	2.84	685
10	Martinez-Medina 2010	Latin America & Caribbean	56.45	43.26	69.01	2.55	62
11	Chisti 2011	South Asia	54.55	47.33	61.62	2.76	198
12	Izadnegahdar 2012	Mixed	15.50	13.66	17.47	2.85	1439
13	Ramakrishna 2012	Sub Saharan Africa	37.34	31.11	43.89	2.78	233
14	Srinivasan 2012	Sub Saharan Africa	28.98	24.29	34.02	2.81	352
15	Jain 2013	South Asia	17.68	12.42	24.03	2.75	181
16	Orimadegun 2013	Sub Saharan Africa	49.20	43.53	54.88	2.80	313
17	Sempertegui 2014	Latin America & Caribbean	85.11	81.48	88.27	2.82	450
18	Ibraheem 2014	Sub Saharan Africa	41.50	34.59	48.66	2.76	200
19	Abdulkadir 2015	Sub Saharan Africa	41.50	34.59	48.66	2.76	200
20	Kelly 2015	Sub Saharan Africa	38.66	32.43	45.16	2.78	238
21	Basnet 2015	South Asia	61.15	57.15	65.04	2.83	610
22	Salah 2015	Sub Saharan Africa	42.67	34.64	50.99	2.73	150
23	Tran 2016	East Asia & Pacific	8.50	6.91	10.33	2.85	1082
24	Nemani 2016	South Asia	40.00	31.67	48.78	2.71	135
25	Alwadhi 2017	South Asia	50.89	41.27	60.46	2.68	112
26	Laghari 2019	South Asia	34.00	24.82	44.15	2.66	100
27	Fagbohun 2020	Latin America & Caribbean	13.02	10.85	15.46	2.84	860
28	Shahrin 2020	South Asia	15.18	10.41	21.07	2.76	191
29	Muro 2020	Sub Saharan Africa	32.40	26.64	38.58	2.78	250
30	Bui-Binh-Bao 2021	East Asia & Pacific	11.39	7.92	15.69	2.79	281
31	Chisti 2021	South Asia	28.13	26.74	29.55	2.86	4007
32	Rahman 2021	South Asia	39.98	38.11	41.88	2.86	2646
33	Saleh 2022	Middle East & North Africa	26.67	20.36	33.76	2.75	180
34	Chisti 2022	South Asia	31.23	29.69	32.80	2.86	3468
35	Jullien 2022	South Asia	75.51	67.74	82.22	2.72	147
36	Kapoor 2022	South Asia	36.11	29.10	43.59	2.75	180
Overall (I2=98.85%, T2=0.13, p<0.05)			34.47	28.90	40.25		25315

Fig. 61: Studies included in prevalence of hypoxaemia all children with WHO pneumonia in tertiary

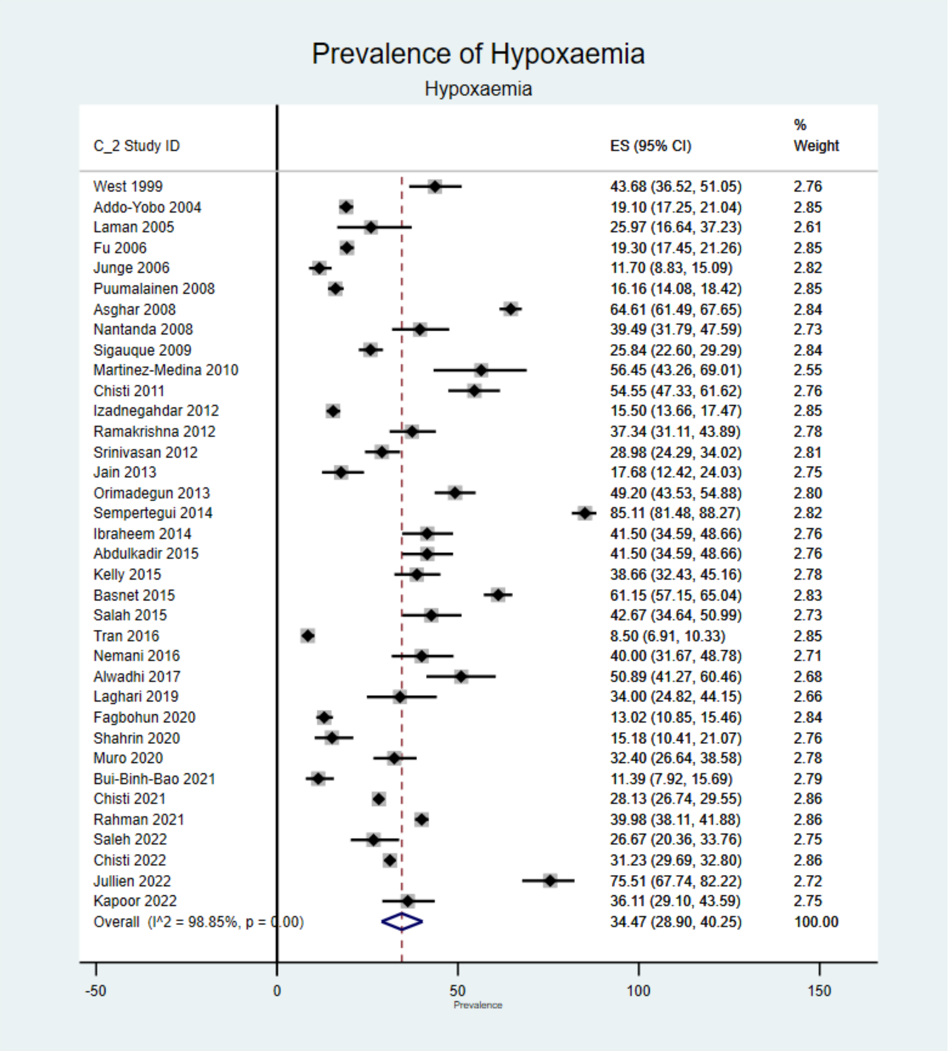

The evidence was assessed as **moderate certainty** because of inconsistency in prevalence estimates between studies.

Tertiary Admitted Children – malaria

Table 62: Studies included in prevalence of hypoxaemia among children with malaria in tertiary setting

Serial	Study ID	WB Region	Proportion	LCL	UCL	Weight	Denominator
1	Junge 2006	Sub Saharan Africa	2.87	1.95	4.08	25.12	1044
2	Cserti-Gazdewich 2013	Sub Saharan Africa	2.26	1.64	3.03	25.21	1901
3	Orimadegun 2013	Sub Saharan Africa	14.42	11.53	17.71	24.92	527
4	Orimadegun 2014	Sub Saharan Africa	29.81	25.19	34.76	24.75	369
**Overall (I2=98.98%, T2=0.12, p<0.05)**			**9.94**	**2.28**	**22.09**		**3841**

Fig. 62: Studies included in prevalence of hypoxaemia among children with malaria in tertiary setting

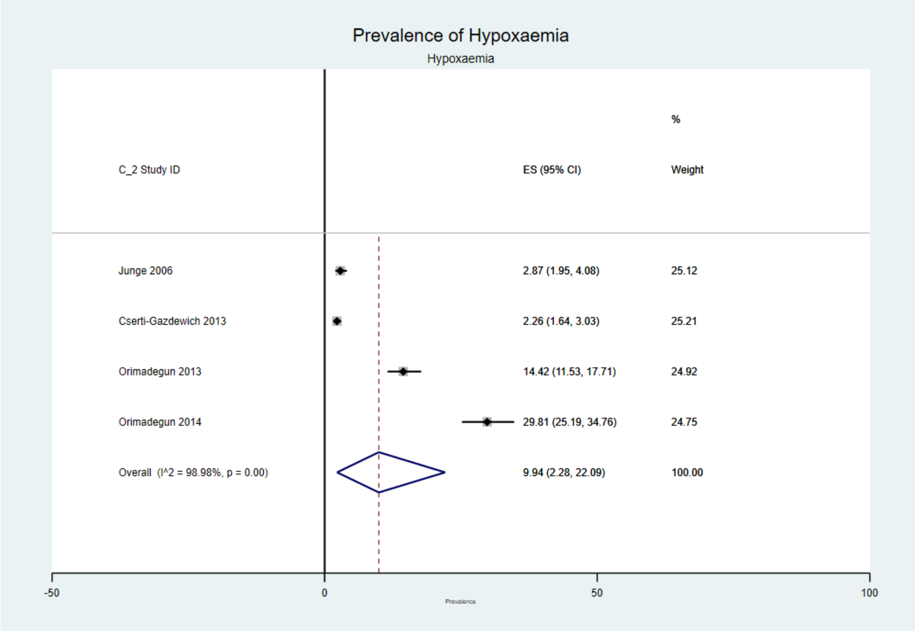

The evidence was assessed as **low certainty** because of imprecision of the estimate, inconsistency in prevalence estimates between studies.

Tertiary Admitted Children – sepsis

Table 63: Studies included in prevalence of hypoxaemia among children with sepsis in tertiary setting

Serial	Study ID	WB Region	Proportion	LCL	UCL	Weight	Denominator
1	Santhanam 2008	South Asia	27.89	20.82	35.88	26.87	147
2	Orimadegun 2013	Sub Saharan Africa	22.62	16.53	29.70	28.14	168
3	Shahid 2016	South Asia	23.08	8.97	43.65	10.10	26
4	Shahunja 2020	South Asia	16.96	13.42	21.00	34.89	401
**Overall (I2=64.50%, T2=0.01, p<0.05)**			**21.79**	**16.25**	**27.89**		**742**

Fig. 63: Studies included in prevalence of hypoxaemia among children with sepsis in tertiary setting

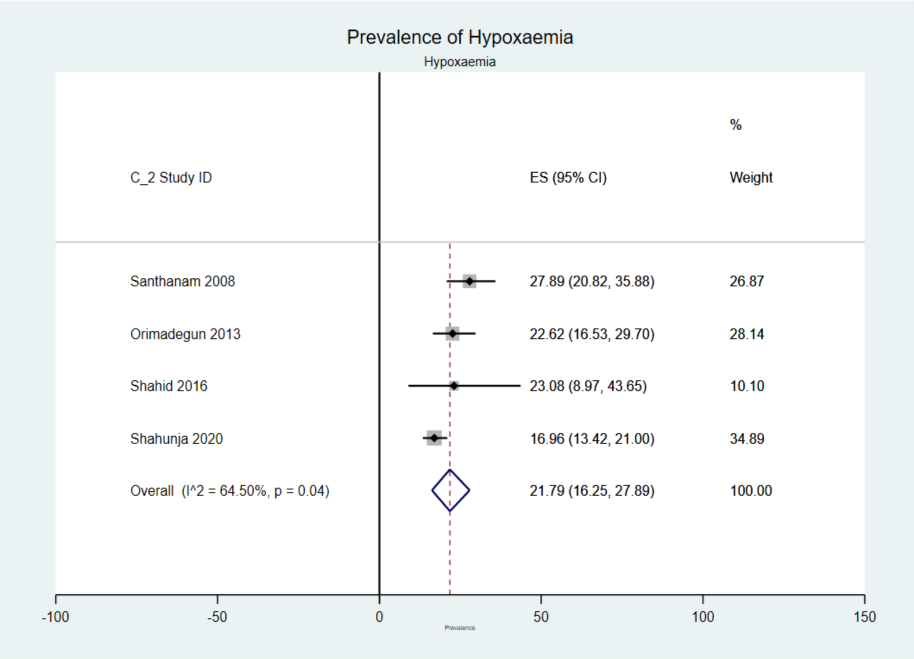

The evidence was assessed as **moderate certainty** because of imprecision of the estimate.

Tertiary Admitted Children – malnutrition

Table 64: Studies included in prevalence of hypoxaemia children with malnutrition in tertiary setting

Serial	Study ID	WB Region	Proportion	LCL	UCL	Weight	Denominator
1	Junge 2006	Sub Saharan Africa	1.85	0.60	4.25	20.36	271
2	Chisti 2013	South Asia	11.08	7.92	14.95	20.44	334
3	Nemani 2016	South Asia	75.00	61.05	85.97	18.63	52
4	Nabukeera-Barungi 2018	Sub Saharan Africa	13.37	8.84	19.10	20.16	187
5	Faruk 2022	South Asia	9.80	6.71	13.70	20.41	306
**Overall (I2=97.27%, T2=0.16, p<0.05)**			**17.49**	**6.02**	**33.13**		**1150**

Fig. 64: Studies included in prevalence of hypoxaemia children with malnutrition in tertiary setting

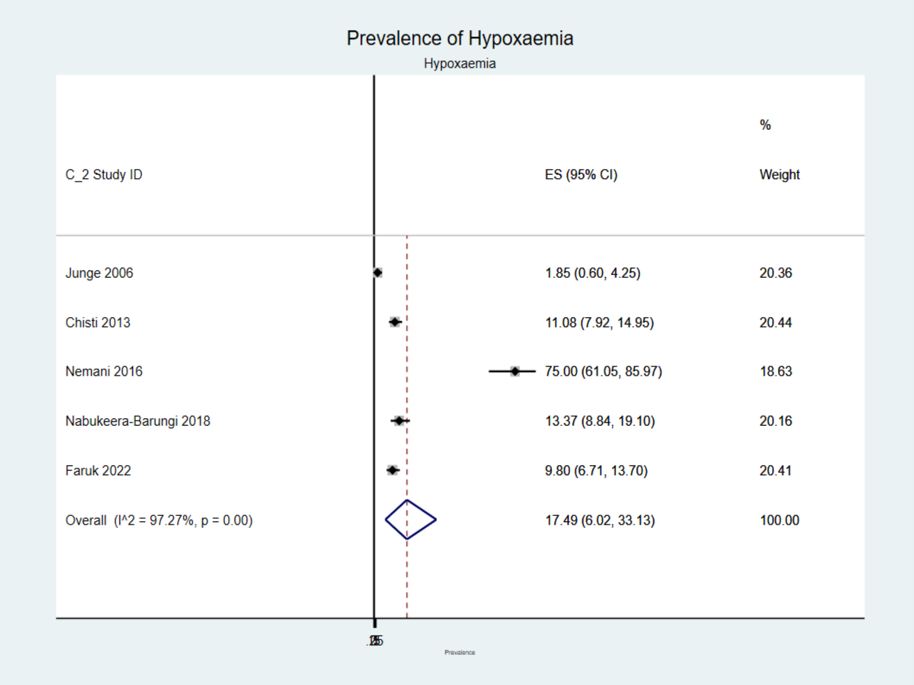

The evidence was assessed as **low certainty** because of imprecision of the estimate, inconsistency in prevalence estimates between studies.

Secondary Admitted Neonates

Table 65: Studies included in prevalence of hypoxaemia among all neonates in secondary setting

Serial	Study ID	WB Region	Proportion	LCL	UCL	Weight	Denominator
1	Duke 2002	East Asia & Pacific	43.18	34.59	52.08	13.03	132
2	English 2003	Sub Saharan Africa	19.97	16.88	23.35	19.77	616
3	Mwaniki 2009	Sub Saharan Africa	18.64	16.39	21.07	21.09	1105
4	McCollum 2013	Sub Saharan Africa	20.59	8.70	37.90	5.83	34
5	Graham 2019	Sub Saharan Africa	18.24	17.37	19.13	22.72	7473
6	Graham 2022	Sub Saharan Africa	16.26	12.42	20.72	17.56	326
**Overall (I2=88.05%, T2=0.01, p<0.05)**			**21.19**	**17.47**	**25.16**		**9686**

Fig. 65: Studies included in prevalence of hypoxaemia among all neonates in secondary setting

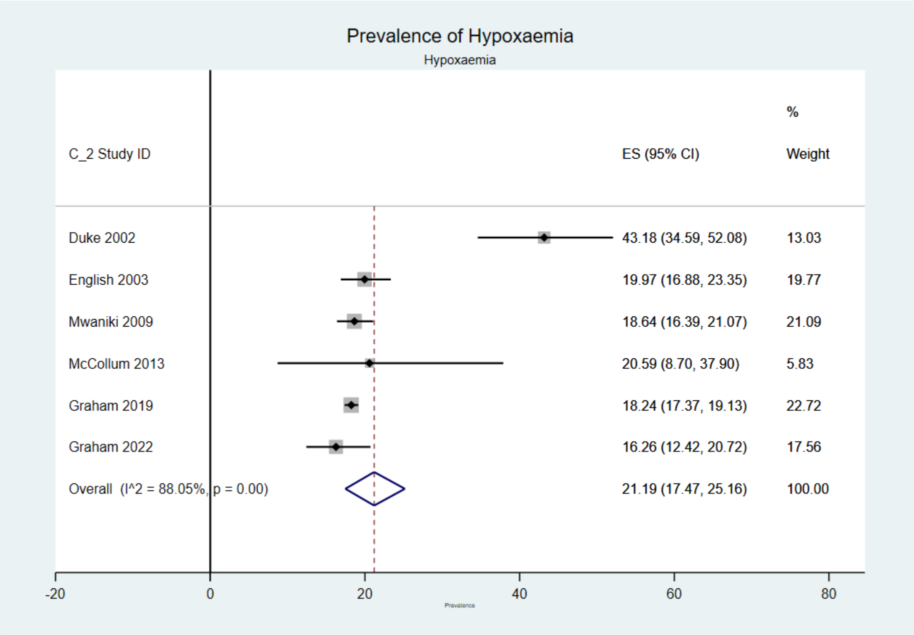

The evidence was assessed as **high certainty**.

Secondary Admitted Children

Table 66: Studies included in prevalence of hypoxaemia among all children in secondary setting

Serial	Study ID	WB Region	Proportion	LCL	UCL	Weight	Denominator
1	Duke 2002	East Asia & Pacific	55.71	50.40	60.92	8.53	359
2	Djelantik 2003	East Asia & Pacific	11.19	10.27	12.17	9.85	4306
3	English 2003	Sub Saharan Africa	14.04	10.27	18.56	8.26	292
4	Wandi 2006	East Asia & Pacific	24.16	22.24	26.15	9.67	1896
5	Mwaniki 2009	Sub Saharan Africa	5.26	4.88	5.65	9.94	13183
6	Foran 2010	Sub Saharan Africa	6.02	1.98	13.50	5.76	83
7	McCollum 2013	Sub Saharan Africa	4.54	3.14	6.32	9.21	727
8	Graham 2019	Sub Saharan Africa	7.93	7.52	8.35	9.95	16453
9	Enoch 2019	Sub Saharan Africa	10.00	9.51	10.50	9.94	14232
10	Tuti 2021	Sub Saharan Africa	6.97	6.77	7.17	9.98	64722
11	Graham 2022	Sub Saharan Africa	11.26	8.64	14.35	8.91	506
**Overall (I2=99.19%, T2=0.02, p<0.05)**			**12.50**	**9.99**	**15.24**		**116759**

Fig. 66: Studies included in prevalence of hypoxaemia among all children in secondary setting

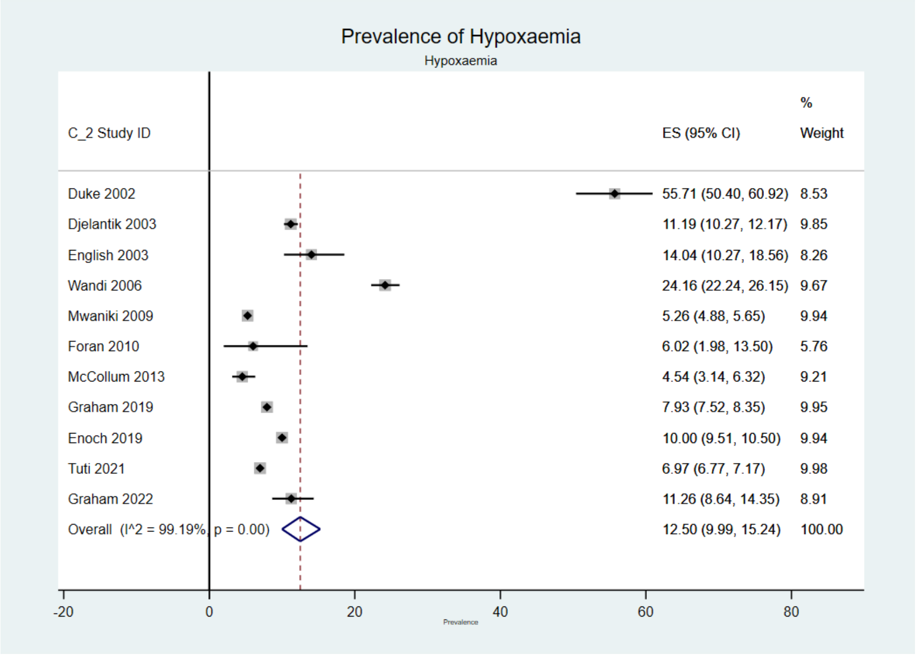

The evidence was assessed as **moderate certainty** because of inconsistency in prevalence estimates between studies.

Secondary Admitted Adults

Table 67: Studies included in prevalence of hypoxaemia among all adults in secondary setting

Serial	Study ID	WB Region	Proportion	LCL	UCL	Weight	Denominator
1	Foran 2010	Sub Saharan Africa	9.17	4.49	16.23	4.04	109
2	Wasingya-Kasereka 2020	Sub Saharan Africa	6.31	5.41	7.31	95.96	2599

Fig. 67: Studies included in prevalence of hypoxaemia among all adults in secondary setting

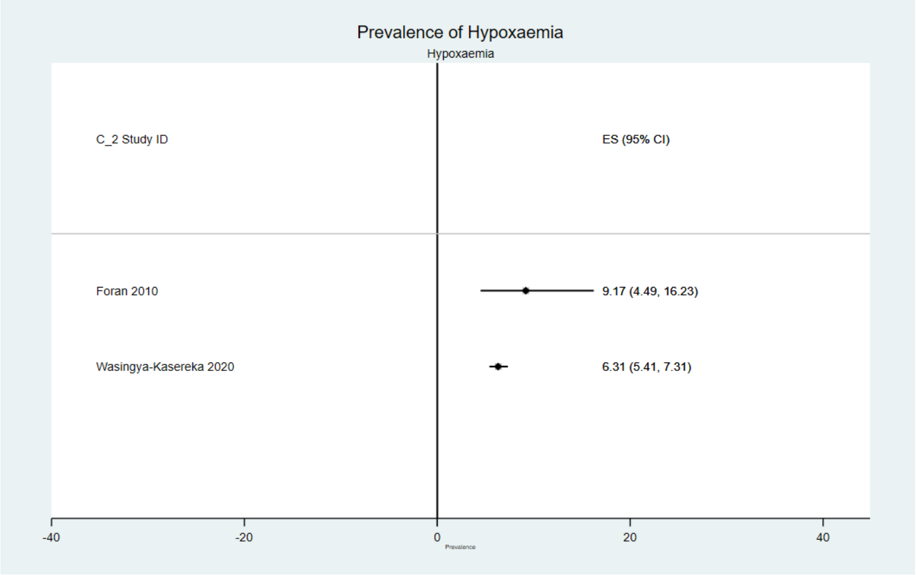

No meta-estimate calculated as only two included studies.

Secondary Admitted Children – pneumonia all WHO-classification

Table 68: Studies included in prevalence of hypoxaemia among children with pneumonia in secondary setting

Serial	Study ID	WB Region	Proportion	LCL	UCL	Weight	Denominator
1	Duke 2002	East Asia & Pacific	72.65	66.29	78.38	6.20	223
2	Djelantik 2003	East Asia & Pacific	11.19	10.27	12.17	6.34	4306
3	Wandi 2006	East Asia & Pacific	54.50	50.34	58.61	6.29	578
4	Duke 2008	East Asia & Pacific	57.09	52.94	61.17	6.29	578
5	Mwaniki 2009	Sub Saharan Africa	8.40	7.68	9.16	6.34	5489
6	Webb 2012	Sub Saharan Africa	23.59	20.16	27.30	6.29	568
7	Kuti 2013	Sub Saharan Africa	19.29	15.62	23.39	6.27	420
8	McCollum 2013	Sub Saharan Africa	17.22	11.57	24.20	6.13	151
9	Kuti 2013	Sub Saharan Africa	19.76	16.01	23.94	6.27	410
10	Bassat 2016	Sub Saharan Africa	27.88	24.84	31.07	6.31	825
11	Graham 2019	Sub Saharan Africa	23.44	21.64	25.33	6.33	2073
12	McCollum 2019	Sub Saharan Africa	64.44	60.61	68.14	6.29	644
13	Oktaria 2021	East Asia & Pacific	13.53	8.22	20.54	6.10	133
14	Ahmed 2022	Sub Saharan Africa	75.32	64.18	84.44	5.94	77
15	Awasthi 2022	South Asia	35.85	34.74	36.97	6.34	7196
16	Mvalo 2022	Sub Saharan Africa	63.75	59.53	67.82	6.28	538
**Overall (I2=99.60%, T2=0.19, p<0.05)**			**35.17**	**25.36**	**45.65**		**24209**

Fig. 68: Studies included in prevalence of hypoxaemia among children with pneumonia in secondary setting

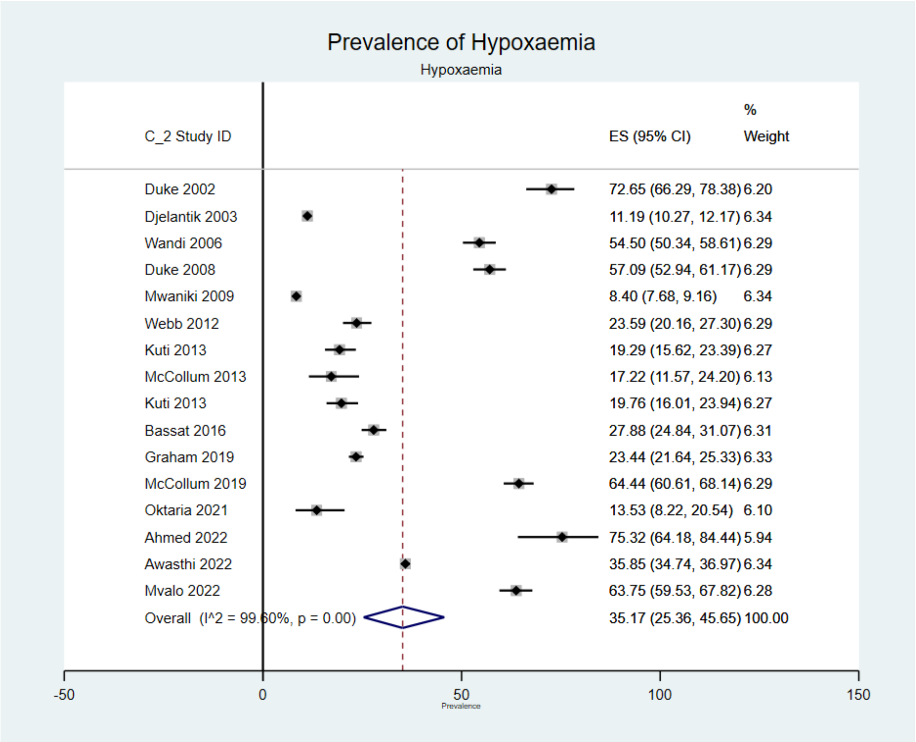

The evidence was assessed as **low certainty** because of imprecision of the estimate, inconsistency in prevalence estimates between studies.

Secondary Admitted Children – malaria

Table 69: Studies included in prevalence of hypoxaemia among children with malaria in secondary setting

Serial	Study ID	WB Region	Proportion	LCL	UCL	Weight	Denominator
1	Duke 2002	East Asia & Pacific	0.00	0.00	52.18	1.48	5
2	Maitland 2003	Sub Saharan Africa	17.17	13.97	20.76	13.73	501
3	Wandi 2006	East Asia & Pacific	3.31	1.52	6.19	12.75	272
4	Mwaniki 2009	Sub Saharan Africa	4.90	4.31	5.53	14.96	4982
5	McCollum 2013	Sub Saharan Africa	1.87	0.81	3.66	13.52	427
6	Graham 2019	Sub Saharan Africa	8.50	7.74	9.31	14.96	5035
7	Olupot-Olupot 2020	Sub Saharan Africa	7.40	5.53	9.67	14.04	662
8	Leligdowicz 2021	Sub Saharan Africa	2.51	1.73	3.50	14.55	1317
**Overall (I2=96.30%, T2=0.02, p<0.05)**			**4.78**	**2.50**	**7.60**		**13201**

Fig. 69: Studies included in prevalence of hypoxaemia among children with malaria in secondary setting

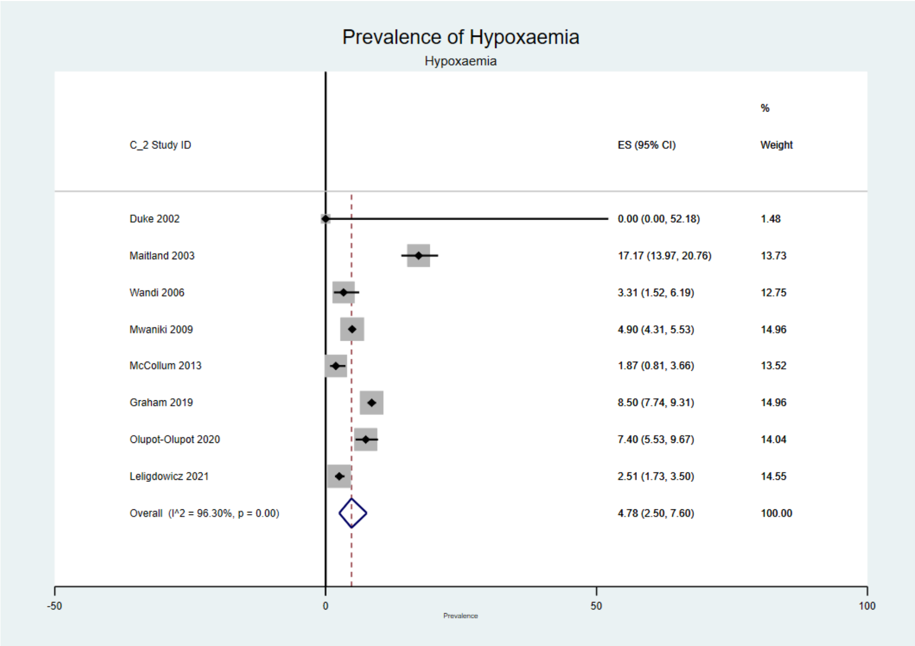

The evidence was assessed as **high certainty**.

Secondary Admitted Children – sepsis

Table 70: Studies included in prevalence of hypoxaemia among children with sepsis in secondary setting

Serial	Study ID	WB Region	Proportion	LCL	UCL	Weight	Denominator
1	Duke 2002	East Asia & Pacific	60.00	26.24	87.84	25.58	10
2	McCollum 2013	Sub Saharan Africa	3.45	0.09	17.76	33.67	29
3	Graham 2019	Sub Saharan Africa	8.70	7.85	9.61	40.74	4092
**Overall (I2=86.40%, T2=0.16, p<0.05)**			**15.29**	**1.18**	**38.24**		**4131**

Fig. 70: Studies included in prevalence of hypoxaemia among children with sepsis in secondary setting

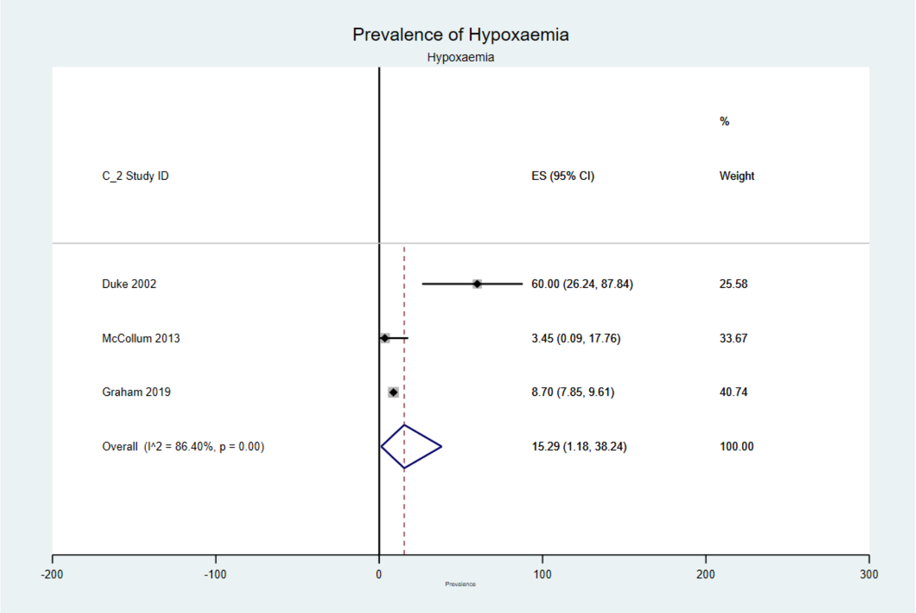

The evidence was assessed as **low certainty** because of imprecision of the estimate, inconsistency in prevalence estimates between studies.

Secondary Admitted Children – malnutrition

Table 71: Studies included in prevalence of hypoxaemia among children with malnutrition in secondary setting

Serial	Study ID	WB Region	Proportion	LCL	UCL	Weight	Denominator
1	Duke 2002	East Asia & Pacific	23.81	8.22	47.17	15.35	21
2	Wandi 2006	East Asia & Pacific	8.33	0.21	38.48	13.49	12
3	Maitland 2006	Sub Saharan Africa	26.31	22.86	29.99	18.84	612
4	McCollum 2013	Sub Saharan Africa	5.26	0.13	26.03	15.06	19
5	Graham 2019	Sub Saharan Africa	3.50	2.56	4.65	18.93	1286
6	Kintwa 2021	East Asia & Pacific	20.71	14.33	28.38	18.33	140
**Overall (I2=97.76%, T2=0.20, p<0.05)**			**13.50**	**2.74**	**29.50**		**2090**

Fig. 71: Studies included in prevalence of hypoxaemia among children with malnutrition in secondary setting

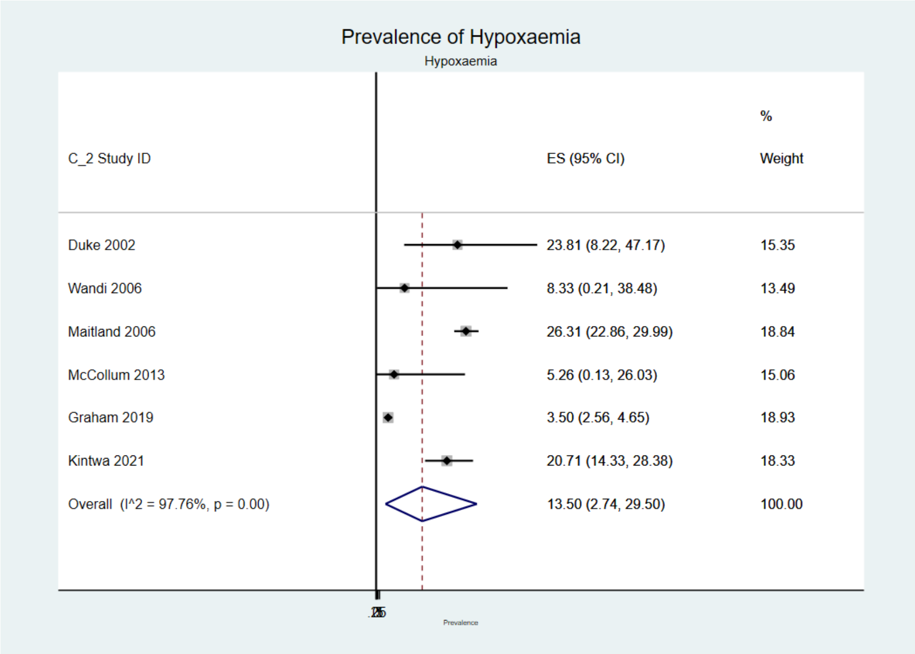

The evidence was assessed as **low certainty** because of imprecision of the estimate, inconsistency in prevalence estimates between studies.

Subgroup Results tables and Forest plots (Altitude and Region)

High versus Low-altitude Admitted Children with pneumonia (WHO-classified pneumonia, severe, very severe)

Table 72: Studies included in prevalence of hypoxaemia among admitted children with pneumonia by altitude

#	Study ID	WB Region	Proportion	LCL	UCL	Weight	Denom
High							
1	Duke 2002	East Asia & Pacific	72.65	66.29	78.38	1.51	223
2	Bruce 2007	Latin America & Caribbean	51.71	45.49	57.89	1.51	263
3	Jullien 2022	South Asia	75.51	67.74	82.22	1.48	147
Overall (I2=93.91, T2=0.07, p<0.05)			66.84	51.11	80.86		633
Low							
1	Usen 1999	Sub Saharan Africa	5.88	4.55	7.46	1.54	1072
2	West 1999	Sub Saharan Africa	43.68	36.52	51.05	1.50	190
3	Djelantik 2003	East Asia & Pacific	11.19	10.27	12.17	1.55	4306
4	Laman 2005	East Asia & Pacific	25.97	16.64	37.23	1.42	77
5	Wandi 2006	East Asia & Pacific	54.50	50.34	58.61	1.53	578
6	Fu 2006	Mixed	19.30	17.45	21.26	1.55	1694
7	Junge 2006	Sub Saharan Africa	11.70	8.83	15.09	1.53	436
8	Puumalainen 2008	East Asia & Pacific	16.16	14.08	18.42	1.54	1151
9	Ashraf 2008	South Asia	56.97	50.60	63.18	1.51	251
10	Nantanda 2008	Sub Saharan Africa	39.49	31.79	47.59	1.49	157
11	Mwaniki 2009	Sub Saharan Africa	8.40	7.68	9.16	1.55	5489
12	Sigauque 2009	Sub Saharan Africa	25.84	22.60	29.29	1.54	685
13	Martinez-Medina 2010	Latin America & Caribbean	56.45	43.26	69.01	1.40	62
14	Ashraf 2010	South Asia	52.50	47.20	57.76	1.52	360
15	Chisti 2011	South Asia	54.55	47.33	61.62	1.50	198
16	Webb 2012	Sub Saharan Africa	23.59	20.16	27.30	1.53	568
17	Izadnegahdar 2012	Mixed	15.50	13.66	17.47	1.54	1439
18	Ramakrishna 2012	Sub Saharan Africa	37.34	31.11	43.89	1.51	233

19	Srinivasan 2012	Sub Saharan Africa	28.98	24.29	34.02	1.52	352
20	Jain 2013	South Asia	17.68	12.42	24.03	1.49	181
21	Kuti 2013	Sub Saharan Africa	19.76	16.01	23.94	1.53	410
22	McCollum 2013	Sub Saharan Africa	17.22	11.57	24.20	1.48	151
23	Orimadegun 2013	Sub Saharan Africa	49.20	43.53	54.88	1.52	313
24	Kuti 2013	Sub Saharan Africa	19.29	15.62	23.39	1.53	420
25	Sempertegu i 2014	Latin America & Caribbean	85.11	81.48	88.27	1.53	450
26	Ibraheem 2014	Sub Saharan Africa	41.50	34.59	48.66	1.50	200
27	Abdulkadir 2015	Sub Saharan Africa	41.50	34.59	48.66	1.50	200
28	Breiman 2015	Sub Saharan Africa	35.74	33.88	37.63	1.55	2563
29	Kelly 2015	Sub Saharan Africa	38.66	32.43	45.16	1.51	238
30	Basnet 2015	South Asia	61.15	57.15	65.04	1.53	610
31	Salah 2015	Sub Saharan Africa	42.67	34.64	50.99	1.48	150
32	Tran 2016	East Asia & Pacific	8.50	6.91	10.33	1.54	1082
33	Bassat 2016	Sub Saharan Africa	27.88	24.84	31.07	1.54	825
34	Nemani 2016	South Asia	40.00	31.67	48.78	1.48	135
35	Alwadhi 2017	South Asia	50.89	41.27	60.46	1.46	112
36	Graham 2019	Sub Saharan Africa	23.44	21.64	25.33	1.55	2073
37	McCollum 2019	Sub Saharan Africa	64.44	60.61	68.14	1.54	644
38	Laghari 2019	South Asia	34.00	24.82	44.15	1.45	100
39	Ashraf 2019	South Asia	10.64	8.00	13.78	1.53	470
40	Dembele 2019	East Asia & Pacific	13.52	12.50	14.59	1.55	4179
41	Ma 2019	Sub Saharan Africa	85.81	79.30	90.89	1.49	155
42	PERCHStud yGroup 2019	Mixed	35.74	34.25	37.26	1.55	3981
43	Fashanu 2020	Sub Saharan Africa	58.38	55.84	60.90	1.54	1497
44	Hooli 2020	Sub Saharan Africa	14.69	12.93	16.59	1.54	1491
45	Fagbohun 2020	Latin America & Caribbean	13.02	10.85	15.46	1.54	860
46	Shahrin 2020	South Asia	15.18	10.41	21.07	1.50	191
47	Muro 2020	Sub Saharan Africa	32.40	26.64	38.58	1.51	250
48	Oktaria 2021	East Asia & Pacific	13.53	8.22	20.54	1.48	133

49	Bui-Binh-Bao 2021	East Asia & Pacific	11.39	7.92	15.69	1.51	281
50	Chisti 2021	South Asia	28.13	26.74	29.55	1.55	4007
51	Rahman 2021	South Asia	39.98	38.11	41.88	1.55	2646
52	LeRoux 2021	Sub Saharan Africa	32.28	24.26	41.15	1.47	127
53	Ahmed 2022	Sub Saharan Africa	75.32	64.18	84.44	1.42	77
54	Awasthi 2022	South Asia	35.85	34.74	36.97	1.55	7196
55	Saleh 2022	Middle East & North Africa	26.67	20.36	33.76	1.49	180
56	Chisti 2022	South Asia	31.23	29.69	32.80	1.55	3468
57	Kapoor 2022	South Asia	36.11	29.10	43.59	1.49	180
58	Mvalo 2022	Sub Saharan Africa	63.75	59.53	67.82	1.53	538
59	Zar 2022	Sub Saharan Africa	24.38	18.61	30.92	1.50	201
Overall (I2=99.28, T2=0.14, p<0.05)			32.63	28.23	37.20		62263
Mixed							
1	Addo-Yobo 2004	Mixed	19.10	17.25	21.04	1.55	1702
2	Duke 2008	East Asia & Pacific	57.09	52.94	61.17	1.53	578
3	Asghar 2008	Mixed	64.61	61.49	67.65	1.54	958
4	Muller 2012	Latin America & Caribbean	22.48	20.66	24.38	1.55	1993
Overall (I2=99.63, T2=0.22, p<0.05)			39.90	19.12	62.79		5231

Figure 72: Studies included in prevalence of hypoxaemia among children with pneumonia (Sub, altitude).

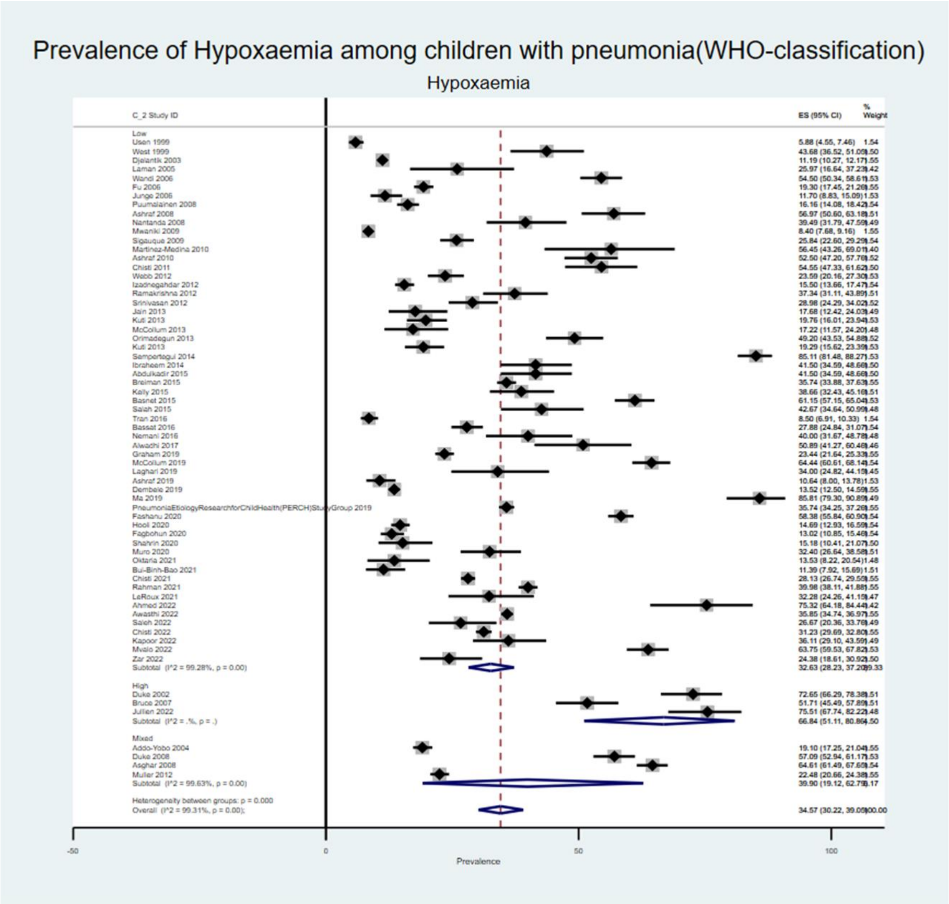

The evidence was assessed as **moderate certainty** because of inconsistency in prevalence estimates between studies.

Regional breakdown Admitted Children with pneumonia (WHO-classified pneumonia, severe, very severe)

Table 73: Studies included in sub-region-wise prevalence of hypoxaemia among children with pneumonia

#	Study ID	Proport	LCL	UCL	Weight	Denom
East Asia & Pacific						
1.	Duke 2002	72.65	66.29	78.38	1.51	223
2.	Djelantik 2003	11.19	10.27	12.17	1.55	4306
3.	Laman 2005	25.97	16.64	37.23	1.42	77
4.	Wandi 2006	54.50	50.34	58.61	1.53	578
5.	Puumalainen 2008	16.16	14.08	18.42	1.54	1151
6.	Duke 2008	57.09	52.94	61.17	1.53	578
7.	Tran 2016	8.50	6.91	10.33	1.54	1082
8.	Dembele 2019	13.52	12.50	14.59	1.55	4179
9.	Oktaria 2021	13.53	8.22	20.54	1.48	133
10.	Bui-Binh-Bao 2021	11.39	7.92	15.69	1.51	281
Overall (I2=99.34, T2=0.14, p<0.05)		26.30	16.57	37.36		12588
Latin America & Caribbean						
1.	Bruce 2007	51.71	45.49	57.89	1.51	263
2.	Martinez-Medina 2010	56.45	43.26	69.01	1.40	62
3.	Muller 2012	22.48	20.66	24.38	1.55	1993
4.	Sempertegui 2014	85.11	81.48	88.27	1.53	450
5.	Fagbohun 2020	13.02	10.85	15.46	1.54	860
Overall (I2=99.56, T2=0.40, p<0.05)		45.11	19.28	72.43		3628
Middle East & North Africa						
1.	Saleh 2022	26.67	20.36	33.76	1.49	180
Mixed						
1.	Addo-Yobo 2004	19.10	17.25	21.04	1.55	1702
2.	Fu 2006	19.30	17.45	21.26	1.55	1694
3.	Asghar 2008	64.61	61.49	67.65	1.54	958
4.	Izadnegahdar 2012	15.50	13.66	17.47	1.54	1439
5.	PneumoniaEtiologyResearchforChildHealth(PERCH) StudyGroup 2019	35.74	34.25	37.26	1.55	3981
Overall (I2=99.57, T2=0.13, p<0.05)		29.76	16.64	44.85		9774
South Asia						
1.	Ashraf 2008	56.97	50.60	63.18	1.51	251
2.	Ashraf 2010	52.50	47.20	57.76	1.52	360

3.	Chisti 2011	54.55	47.33	61.62	1.50	198
4.	Jain 2013	17.68	12.42	24.03	1.49	181
5.	Basnet 2015	61.15	57.15	65.04	1.53	610
6.	Nemani 2016	40.00	31.67	48.78	1.48	135
7.	Alwadhi 2017	50.89	41.27	60.46	1.46	112
8.	Laghari 2019	34.00	24.82	44.15	1.45	100
9.	Ashraf 2019	10.64	8.00	13.78	1.53	470
10.	Shahrin 2020	15.18	10.41	21.07	1.50	191
11.	Chisti 2021	28.13	26.74	29.55	1.55	4007
12.	Rahman 2021	39.98	38.11	41.88	1.55	2646
13.	Awasthi 2022	35.85	34.74	36.97	1.55	7196
14.	Chisti 2022	31.23	29.69	32.80	1.55	3468
15.	Jullien 2022	75.51	67.74	82.22	1.48	147
16.	Kapoor 2022	36.11	29.10	43.59	1.49	180
Overall (I2=98.10, T2=0.05, p<0.05)		39.10	33.67	44.67		20252
Sub Saharan Africa						
1.	Usen 1999	5.88	4.55	7.46	1.54	1072
2.	West 1999	43.68	36.52	51.05	1.50	190
3.	Junge 2006	11.70	8.83	15.09	1.53	436
4.	Nantanda 2008	39.49	31.79	47.59	1.49	157
5.	Mwaniki 2009	8.40	7.68	9.16	1.55	5489
6.	Sigauque 2009	25.84	22.60	29.29	1.54	685
7.	Webb 2012	23.59	20.16	27.30	1.53	568
8.	Ramakrishna 2012	37.34	31.11	43.89	1.51	233
9.	Srinivasan 2012	28.98	24.29	34.02	1.52	352
10.	Kuti 2013	19.76	16.01	23.94	1.53	410
11.	McCollum 2013	17.22	11.57	24.20	1.48	151
12.	Orimadegun 2013	49.20	43.53	54.88	1.52	313
13.	Kuti 2013	19.29	15.62	23.39	1.53	420
14.	Ibraheem 2014	41.50	34.59	48.66	1.50	200
15.	Abdulkadir 2015	41.50	34.59	48.66	1.50	200
16.	Breiman 2015	35.74	33.88	37.63	1.55	2563
17.	Kelly 2015	38.66	32.43	45.16	1.51	238
18.	Salah 2015	42.67	34.64	50.99	1.48	150
19.	Bassat 2016	27.88	24.84	31.07	1.54	825
20.	Graham 2019	23.44	21.64	25.33	1.55	2073
21.	McCollum 2019	64.44	60.61	68.14	1.54	644
22.	Ma 2019	85.81	79.30	90.89	1.49	155
23.	Fashanu 2020	58.38	55.84	60.90	1.54	1497
24.	Hooli 2020	14.69	12.93	16.59	1.54	1491
25.	Muro 2020	32.40	26.64	38.58	1.51	250
26.	LeRoux 2021	32.28	24.26	41.15	1.47	127

27.	Ahmed 2022	75.32	64.18	84.44	1.42	77
28.	Mvalo 2022	63.75	59.53	67.82	1.53	538
29.	Zar 2022	24.38	18.61	30.92	1.50	201
Overall (I2=99.28, T2=0.20, p<0.05)		34.47	26.89	42.46		21705

Fig. 73: Studies included in sub-region-wise prevalence of hypoxaemia among children with pneumonia by World Bank region

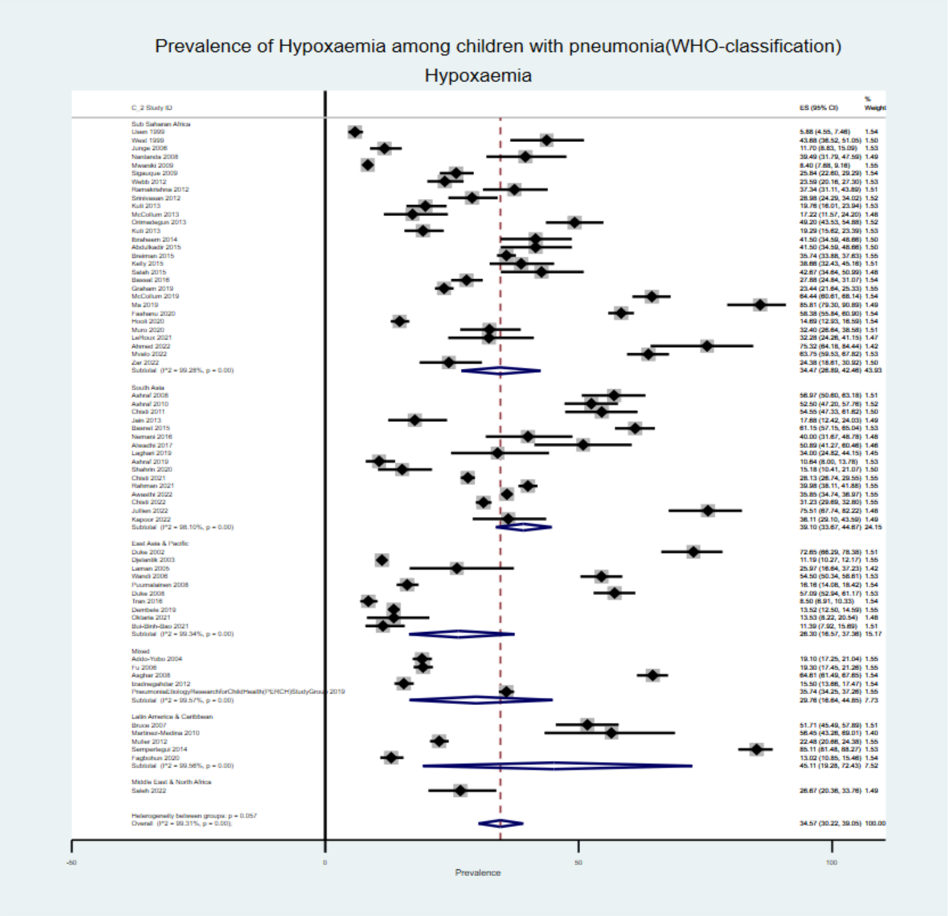

Secondary Results tables and Forest plots (Relative Odds of Death)

Relative Odds of Death – neonates

Table 74: Studies included in odds ratio of mortality (hypoxaemic vs. normoxaemic) among all admitted neonates.

Serial	StudyID	Region	OR	LCL	UCL	Weight	Total
1.	Ondoa-Onama 2003	Sub Saharan Africa	4.94	1.47	16.54	10.63	124
2.	Morgan 2018	Sub Saharan Africa	6.51	3.51	12.06	40.84	407
3.	Kiputa 2022	Sub Saharan Africa	6.18	3.51	10.87	48.54	348
**Overall (I2=0, p=0.924)**			**6.16**	**4.153**	**9.137**		**879**

Fig. 74: Studies included in odds ratio of mortality (hypoxaemic vs. normoxaemic) among all admitted neonates.

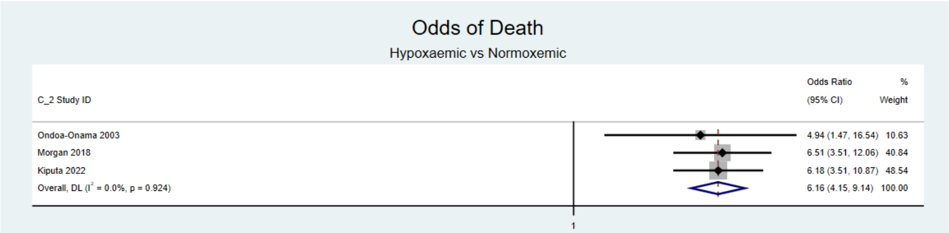

Relative Odds of Death – neonates with non-primary respiratory disease

Table 75: Studies included in odds ratio of mortality (hypoxaemic vs. normoxaemic) among admitted neonates with non-respiratory disease

Serial	StudyID	Region	OR	LCL	UCL	Weight	Total
1.	Ondoa-Onama 2003	Sub Saharan Africa	4.94	1.47	16.54	100	124

Fig. 75: Studies included in odds ratio of mortality (hypoxaemic vs. normoxaemic) among all admitted neonates with non-respiratory disease

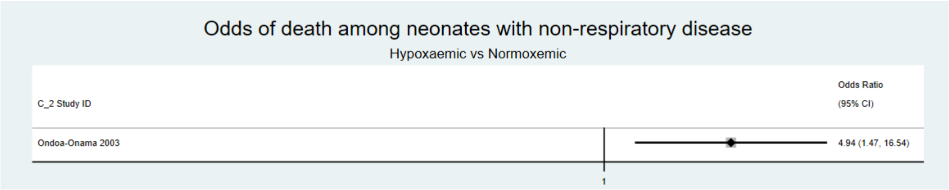

Relative Odds of Death - children

Table 76: Studies included in odds ratio of mortality (hypoxaemic vs. normoxaemic) among all admitted children

Serial	StudyID	Region	OR	LCL	UCL	Weight	Total
1.	Usen 1999	Sub Saharan Africa	5.10	2.22	11.70	2.74	1072
2.	West 1999	Sub Saharan Africa	2.71	0.77	9.56	2.13	118
3.	Djelantik 2003	East Asia & Pacific	5.56	4.39	7.05	3.44	4306
4.	Maitland 2006	Sub Saharan Africa	1.52	1.02	2.28	3.30	718
5.	Asghar 2008	Mixed	2.55	1.34	4.85	3.01	958
6.	Nantanda 2008	Sub Saharan Africa	6.07	2.25	16.36	2.51	157
7.	Sigauque 2009	Sub Saharan Africa	2.19	1.13	4.25	2.98	584
8.	Libster 2010	Latin America & Caribbean	0.32	0.10	1.04	2.25	251
9.	Ashraf 2010	South Asia	0.18	0.01	3.75	0.73	360
10.	Chisti 2011	South Asia	8.98	3.02	26.64	2.37	258
11.	Ramakrishna 2012	Sub Saharan Africa	6.52	2.49	17.07	2.55	233
12.	Chisti 2013	South Asia	22.67	5.98	85.92	2.03	140
13.	Cserti-Gazdewich 2013	Sub Saharan Africa	4.79	2.23	10.29	2.84	849
14.	Kuti 2013	Sub Saharan Africa	3.05	1.05	8.87	2.40	389
15.	Orimadegun 2014	Sub Saharan Africa	4.71	2.16	10.27	2.81	369
16.	Abdulkadir 2015	Sub Saharan Africa	61.84	3.66	1044.98	0.82	200
17.	Lowlaavar 2016	Sub Saharan Africa	2.88	1.72	4.81	3.17	1291
18.	Bassat 2016	Sub Saharan Africa	3.00	1.87	4.81	3.23	825
19.	Barennes 2016	East Asia & Pacific	4.45	1.74	11.39	2.58	350
20.	Benet 2017	Mixed	3.83	1.29	11.42	2.36	405
21.	Alwadhi 2017	South Asia	9.34	0.49	177.63	0.77	112
22.	Zampoli 2017	Sub Saharan Africa	7.78	1.01	59.81	1.30	206
23.	Hau 2018	Sub Saharan Africa	4.55	2.12	9.76	2.84	537
24.	Nabukeera-Barungi 2018	Sub Saharan Africa	3.27	1.22	8.76	2.52	400
25.	McCollum 2019	Sub Saharan Africa	1.02	0.64	1.63	3.23	644
26.	Ma 2019	Sub Saharan Africa	3.94	0.50	30.88	1.28	155
27.	Hooli 2020	Sub Saharan Africa	4.60	2.32	9.14	2.95	1491
28.	Shahunja 2020	South Asia	14.73	7.35	29.52	2.94	401
29.	Fagbohun 2020	Latin America & Caribbean	11.07	6.23	19.66	3.10	860
30.	Olupot-Olupot 2020	Sub Saharan Africa	4.60	2.32	9.13	2.95	662
31.	Shahrin 2020	South Asia	4.53	1.44	14.27	2.28	176
32.	Rao 2021	South Asia	8.00	2.43	26.33	2.22	119
33.	Bui-Binh-Bao 2021	East Asia & Pacific	69.44	8.21	587.42	1.22	281
34.	Chisti 2021	South Asia	13.55	9.24	19.88	3.32	4007

35.	Kintwa 2021	East Asia & Pacific	10.49	3.62	30.39	2.40	140
36.	Oliveira 2022	Latin America & Caribbean	2.78	2.42	3.20	3.49	10911
37.	Krithika 2022	South Asia	191.45	76.61	478.48	2.62	600
38.	Awasthi 2022	South Asia	3.40	2.06	5.59	3.19	7196
39.	Chisti 2022	South Asia	14.60	9.77	21.83	3.30	3468
40.	Kapoor 2022	South Asia	16.95	3.74	76.92	1.81	180
**Overall (I2=87.9%, p<0.05)**			5.232	3.905	7.009		46379

Fig. 76: Studies included in odds ratio of mortality (hypoxaemic vs. normoxaemic) among all admitted children

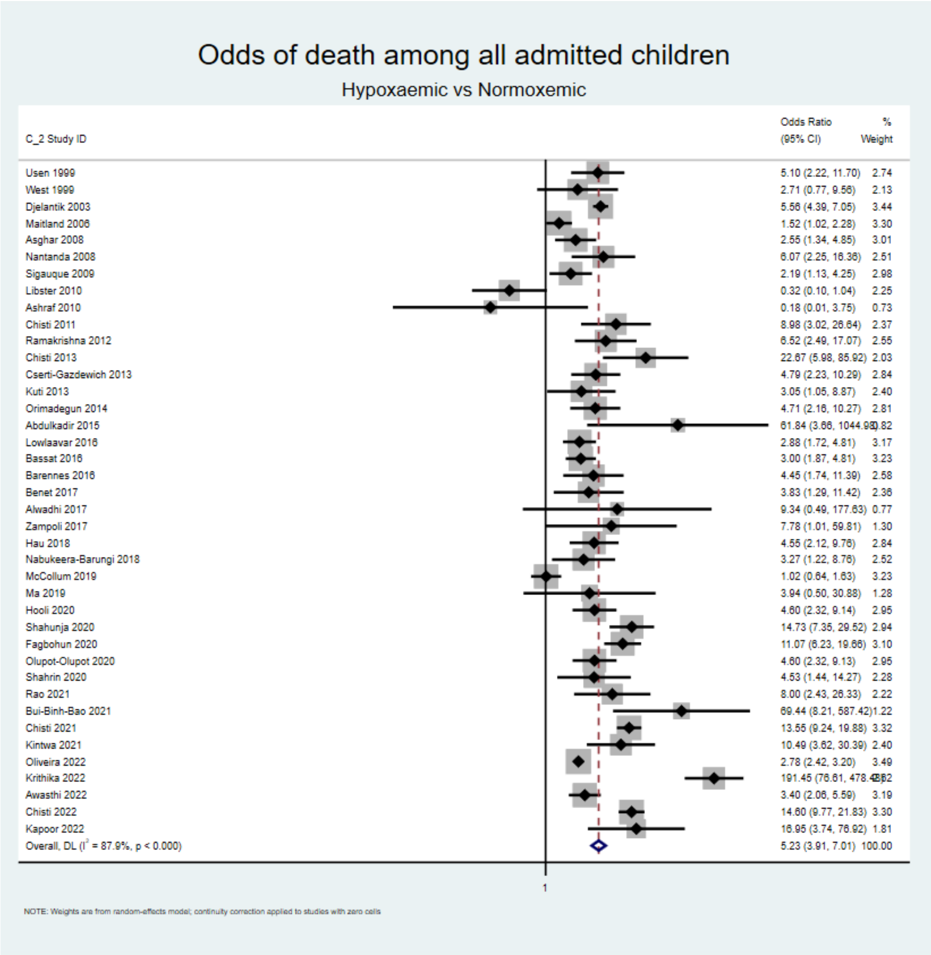
Odds of death among all admitted children
Hypoxaemic vs Normoxemic
C_2 Study ID
Odds Ratio (95% CI)
% Weight
Usen 1999 5.10 (2.22, 11.70) 2.74
West 1999 2.71 (0.77, 9.56) 2.13
Djelantik 2003 5.56 (4.39, 7.05) 3.44
Maitland 2006 1.52 (1.02, 2.28) 3.30
Asghar 2008 2.55 (1.34, 4.85) 3.01
Nantanda 2008 6.07 (2.25, 16.36) 2.51
Sigauque 2009 2.19 (1.13, 4.25) 2.98
Libster 2010 0.32 (0.10, 1.04) 2.25
Ashraf 2010 0.18 (0.01, 3.75) 0.73
Chisti 2011 8.98 (3.02, 26.64) 2.37
Ramakrishna 2012 6.52 (2.49, 17.07) 2.55
Chisti 2013 22.67 (5.98, 85.92) 2.03
Cserti-Gazdewich 2013 4.79 (2.23, 10.29) 2.84
Kuti 2013 3.05 (1.05, 8.87) 2.40
Orimadegun 2014 4.71 (2.16, 10.27) 2.81
Abdulkadir 2015 61.84 (3.66, 1044.9
Lowlaavar 2016 2.88 (1.72, 4.81) 3.17
Bassat 2016 3.00 (1.87, 4.81) 3.23
Barennes 2016 4.45 (1.74, 11.39) 2.58
Benet 2017 3.83 (1.29, 11.42) 2.36
Alwadhi 2017 9.34 (0.49, 177.63) 0.77
Zampoli 2017 7.78 (1.01, 59.81) 1.30
Hau 2018 4.55 (2.12, 9.76) 2.84
Nabukeera-Barungi 2018 3.27 (1.22, 8.76) 2.52
McCollum 2019 1.02 (0.64, 1.63) 3.23
Ma 2019 3.94 (0.50, 30.88) 1.28
Hooli 2020 4.60 (2.32, 9.14) 2.95
Shahunja 2020 14.73 (7.35, 29.52) 2.94
Fagbohun 2020 11.07 (6.23, 19.66) 3.10
Olupot-Olupot 2020 4.60 (2.32, 9.13) 2.95
Shahrin 2020 4.53 (1.44, 14.27) 2.28
Rao 2021 8.00 (2.43, 26.33) 2.22
Bui-Binh-Bao 2021 69.44 (8.21, 587.42)1.22
Chisti 2021 13.55 (9.24, 19.88) 3.32
Kintwa 2021 10.49 (3.62, 30.39) 2.40
Oliveira 2022 2.78 (2.42, 3.20) 3.49
Krithika 2022 191.45 (76.61, 478.4
Awasthi 2022 3.40 (2.06, 5.59) 3.19
Chisti 2022 14.60 (9.77, 21.83) 3.30
Kapoor 2022 16.95 (3.74, 76.92) 1.81
Overall, DL (I² = 87.9%, p < 0.000) 5.23 (3.91, 7.01) 100.00
1
NOTE: Weights are from random-effects model; continuity correction applied to studies with zero cells

Relative Odds of Death – children with primary respiratory disease

Table 77: Studies included in odds ratio of mortality (hypoxaemic vs. normoxaemic) among admitted children with respiratory disease

Serial	StudyID	Region	OR	LCL	UCL	Weight	Total
1.	West 1999	Sub Saharan Africa	2.71	0.77	9.56	4.11	118
2.	Asghar 2008	Mixed	2.55	1.34	4.85	5.59	958
3.	Nantanda 2008	Sub Saharan Africa	6.07	2.25	16.36	4.76	157
4.	Sigauque 2009	Sub Saharan Africa	2.19	1.13	4.25	5.54	584
5.	Libster 2010	Latin America & Caribbean	0.32	0.10	1.04	4.33	251
6.	Ashraf 2010	South Asia	0.18	0.01	3.75	1.51	360
7.	Ramakrishna 2012	Sub Saharan Africa	6.52	2.49	17.07	4.83	233
8.	Kuti 2013	Sub Saharan Africa	3.05	1.05	8.87	4.58	389
9.	Abdulkadir 2015	Sub Saharan Africa	61.84	3.66	1044.98	1.69	200
10.	Benet 2017	Mixed	3.83	1.29	11.42	4.51	405
11.	Alwadhi 2017	South Asia	9.34	0.49	177.63	1.59	112
12.	Zampoli 2017	Sub Saharan Africa	7.78	1.01	59.81	2.61	206
13.	McCollum 2019	Sub Saharan Africa	1.02	0.64	1.63	5.93	644
14.	Ma 2019	Sub Saharan Africa	3.94	0.50	30.88	2.58	155
15.	Hooli 2020	Sub Saharan Africa	4.60	2.32	9.14	5.49	1491
16.	Fagbohun 2020	Latin America & Caribbean	11.07	6.23	19.66	5.73	860
17.	Rao 2021	South Asia	8.00	2.43	26.33	4.28	119
18.	Bui-Binh-Bao 2021	East Asia & Pacific	69.44	8.21	587.42	2.47	281
19.	Chisti 2021	South Asia	13.55	9.24	19.88	6.07	4007
20.	Oliveira 2022	Latin America & Caribbean	2.78	2.42	3.20	6.34	10911
21.	Awasthi 2022	South Asia	3.40	2.06	5.59	5.88	7196
22.	Chisti 2022	South Asia	14.60	9.77	21.83	6.04	3468
23.	Kapoor 2022	South Asia	16.95	3.74	76.92	3.56	180
**Overall (I2=88.6%, p<0.05)**			4.562	2.972	7.004		33285

Fig. 77: Studies included in odds ratio of mortality (hypoxaemic vs. normoxaemic) among admitted children with respiratory disease

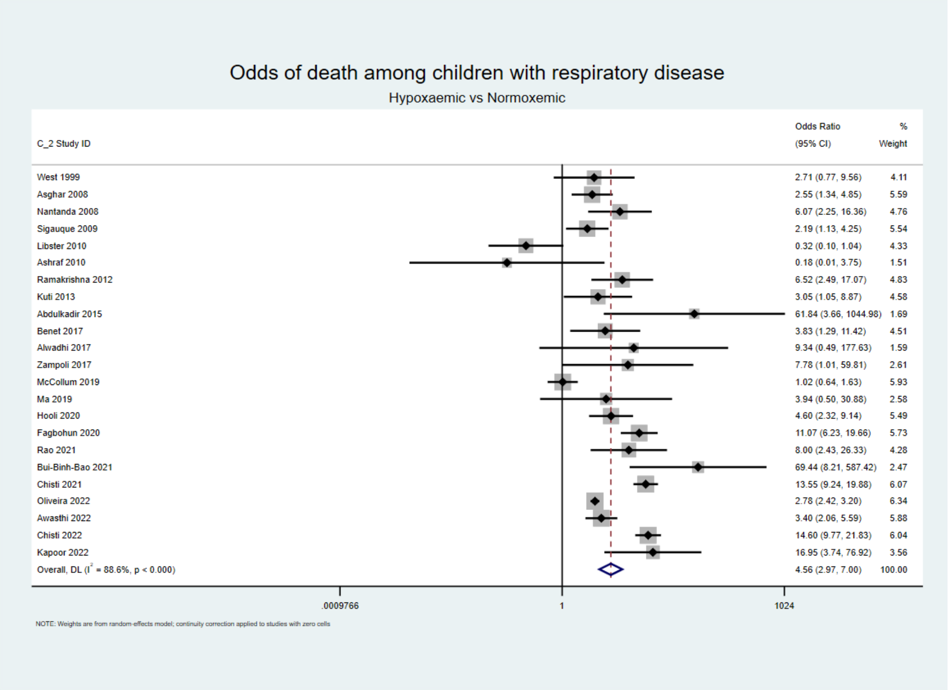

Relative Odds of Death – children with non-primary respiratory disease

Table 78: **Studies included in odds ratio of mortality (hypoxaemic vs. normoxaemic) among admitted children with non-respiratory disease**

Serial	StudyID	Region	OR	LCL	UCL	Weight	Total
1.	Maitland 2006	Sub Saharan Africa	1.52	1.02	2.28	16.5	718
2.	Cserti-Gazdewich 2013	Sub Saharan Africa	4.79	2.23	10.29	14.36	849
3.	Orimadegun 2014	Sub Saharan Africa	4.71	2.16	10.27	14.26	369
4.	Nabukeera-Barungi 2018	Sub Saharan Africa	3.27	1.22	8.76	12.85	400
5.	Shahunja 2020	South Asia	14.73	7.35	29.52	14.83	401
6.	Olupot-Olupot 2020	Sub Saharan Africa	4.60	2.32	9.13	14.89	662
7.	Kintwa 2021	East Asia & Pacific	10.49	3.62	30.39	12.31	140
**Overall (I2=84.5%, p<0.05)**			**4.87**	**2.452**	**9.674**		**3539**

Fig. 78: **Studies included in odds ratio of mortality (hypoxaemic vs. normoxaemic) among admitted children with non-respiratory disease**

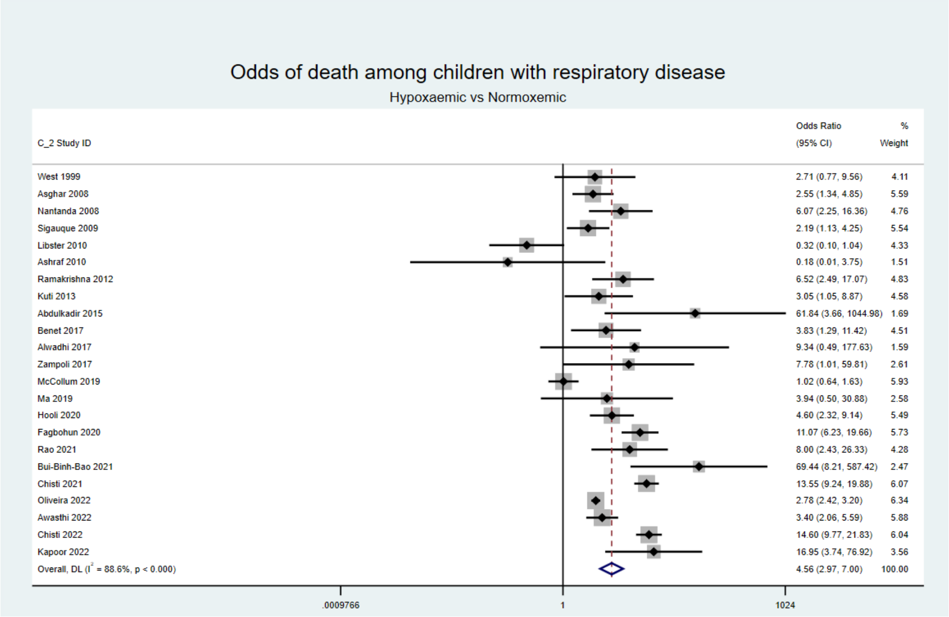

Relative Odds of Death – adults

Table 79: **Studies included in odds ratio of mortality (hypoxaemic vs. normoxaemic) among all admitted adults**

Seria l	StudyID	Region	OR	LCL	UCL	Weigh t	Total
1.	Amare 2008	Sub Saharan Africa	1.29	0.48	3.50	2.66	119
2.	Husain 2009	South Asia	5.73	0.28	117.6 5	0.63	30
3.	Tokman 2014	Sub Saharan Africa	3.67	1.54	8.75	2.94	241
4.	Koss 2015	Sub Saharan Africa	2.47	1.63	3.74	3.95	835
5.	Carugati 2018	Sub Saharan Africa	3.83	1.76	8.34	3.15	419
6.	Worodria 2018	Sub Saharan Africa	3.70	2.72	5.03	4.14	1887
7.	Aston 2019	Sub Saharan Africa	5.38	2.99	9.70	3.59	431
8.	YeLynn 2019	East Asia & Pacific	0.33	0.13	0.83	2.79	118
9.	Boonmee 2020	East Asia & Pacific	2.17	1.72	2.75	4.25	1616
10.	Ojuawo 2020	Sub Saharan Africa	9.90	2.91	33.69	2.22	102
11.	Homayounieh 2020	Middle East & North Africa	2.63	0.81	8.46	2.32	75
12.	Bepouka 2020	Sub Saharan Africa	15.3 2	3.51	66.96	1.81	141
13.	Wasingya-Kasereka 2020	Sub Saharan Africa	10.1 9	6.87	15.11	3.99	2599
14.	Mejia 2020	Latin America & Caribbean	5.80	3.42	9.83	3.72	500
15.	Acar 2021	Europe & Central Asia	5.99	3.36	10.69	3.61	671
16.	Diaz-Velez 2021	Latin America & Caribbean	4.54	3.09	6.68	4.01	493
17.	Padmaprakash 2021	South Asia	86.5 1	47.8 2	156.4 9	3.58	1536
18.	Anyaypoma-Ocon 2021	Latin America & Caribbean	1.11	0.72	1.71	3.91	324
19.	MarMinn 2021	East Asia & Pacific	3.86	1.96	7.60	3.38	507
20.	Sarfaraz 2021	South Asia	3.35	1.63	6.88	3.28	170
21.	Honarvar 2021	Middle East & North Africa	3.63	2.73	4.83	4.18	1975
22.	Kayambankadzanja 2021	Sub Saharan Africa	3.92	1.87	8.23	3.23	1135
23.	Xiong 2021	East Asia & Pacific	40.4 4	20.7 5	78.83	3.4	799
24.	Marcolino 2021	Latin America & Caribbean	3.13	2.37	4.14	4.19	1907
25.	Nemati 2021	Middle East & North Africa	1.31	0.83	2.06	3.88	946
26.	Arana-Calderon 2022	Latin America & Caribbean	26.8 6	3.43	210.2 9	1.16	158
27.	Siqueira 2022	Latin America & Caribbean	4.46	4.02	4.96	4.37	1510 5
28.	Araban 2022	Middle East & North Africa	5.20	4.00	6.77	4.21	3181

29.	Soto 2022	Latin America & Caribbean	5.12	3.99	6.58	4.23	1323
30.	Alizadehsani 2022	Middle East & North Africa	6.48	3.07	13.67	3.22	600
**Overall (I2=91.0%, p<0.05)**			**4.436**	**3.424**	**5.746**		**39943**

Fig. 79: **Studies included in odds ratio of mortality (hypoxaemic vs. normoxaemic) among all admitted adults**

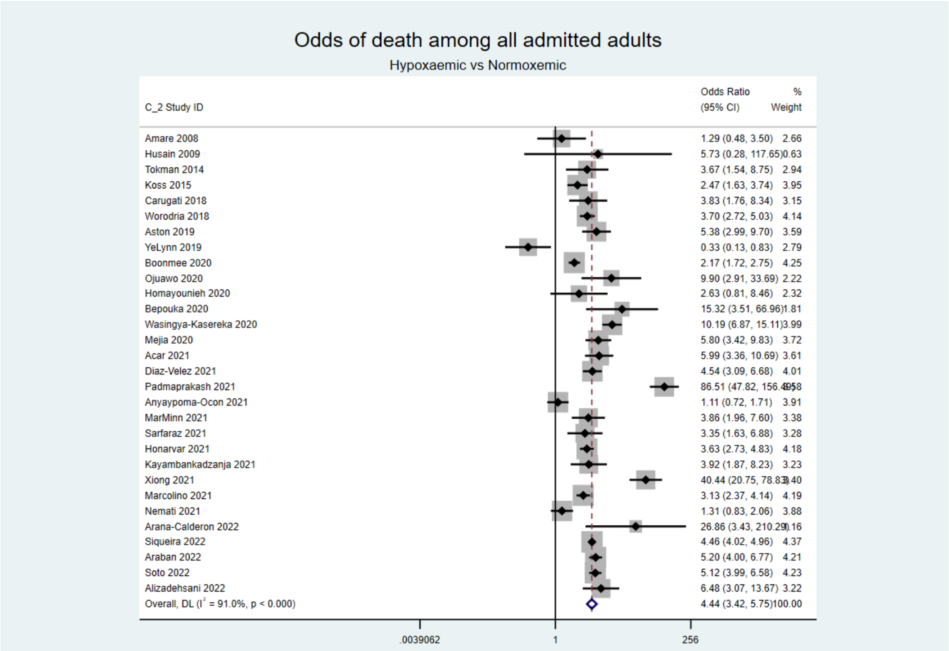

Relative Odds of Death – adults with primary respiratory disease

Table 80: **Studies included in odds ratio of mortality (hypoxaemic vs. normoxaemic) among admitted adults with respiratory disease**

Serial	StudyID	Region	OR	LCL	UCL	Weight	Total
1.	Husain 2009	South Asia	5.73	0.28	117.65	0.98	30
2.	Tokman 2014	Sub Saharan Africa	3.67	1.54	8.75	4.53	241
3.	Aston 2019	Sub Saharan Africa	5.38	2.99	9.70	5.5	431
4.	Ojuawo 2020	Sub Saharan Africa	9.90	2.91	33.69	3.43	102
5.	Homayounieh 2020	Middle East & North Africa	2.63	0.81	8.46	3.58	75
6.	Bepouka 2020	Sub Saharan Africa	15.32	3.51	66.96	2.81	141
7.	Mejia 2020	Latin America & Caribbean	5.80	3.42	9.83	5.7	500
8.	Acar 2021	Europe & Central Asia	5.99	3.36	10.69	5.53	671
9.	Diaz-Velez 2021	Latin America & Caribbean	4.54	3.09	6.68	6.13	493
10.	Padmaprakash 2021	South Asia	86.51	47.82	156.49	5.48	1536
11.	Anyaypoma-Ocon 2021	Latin America & Caribbean	1.11	0.72	1.71	5.99	324
12.	Sarfaraz 2021	South Asia	3.35	1.63	6.88	5.04	170
13.	Honarvar 2021	Middle East & North Africa	3.63	2.73	4.83	6.39	1975
14.	Xiong 2021	East Asia & Pacific	40.44	20.75	78.83	5.23	799
15.	Marcolino 2021	Latin America & Caribbean	3.13	2.37	4.14	6.4	1907
16.	Nemati 2021	Middle East & North Africa	1.31	0.83	2.06	5.93	946
17.	Arana-Calderon 2022	Latin America & Caribbean	26.86	3.43	210.29	1.81	158
18.	Siqueira 2022	Latin America & Caribbean	4.46	4.02	4.96	6.67	15105
19.	Araban 2022	Middle East & North Africa	5.20	4.00	6.77	6.43	3181
20.	Soto 2022	Latin America & Caribbean	5.12	3.99	6.58	6.46	1323
**Overall (I2=91.6%, p<0.05)**			**5.46**	**3.949**	**7.549**		**30108**

Fig. 80: **Studies included in odds ratio of mortality (hypoxaemic vs. normoxaemic) among admitted adults with primary respiratory disease**

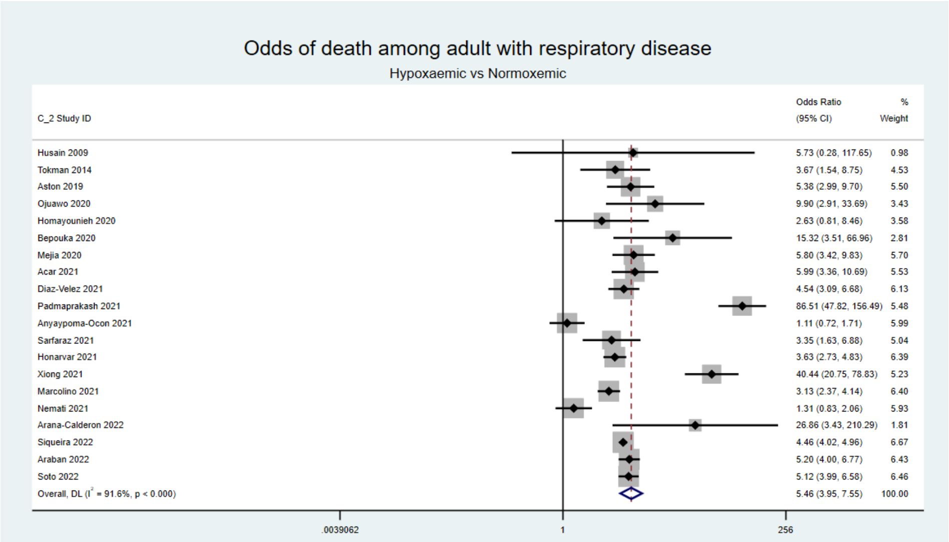

Relative Odds of Death – adults with non-primary respiratory disease

Table 81: **Studies included in odds ratio of mortality (hypoxaemic vs. normoxaemic) among admitted adults with non-respiratory disease**

Serial	StudyID	Region	OR	LCL	UCL	Weight	Total
1.	Amare 2008	Sub Saharan Africa	1.29	0.48	3.50	12	119
2.	Koss 2015	Sub Saharan Africa	2.47	1.63	3.74	20.68	835
3.	Carugati 2018	Sub Saharan Africa	3.83	1.76	8.34	14.98	419
4.	YeLynn 2019	East Asia & Pacific	0.33	0.13	0.83	12.76	118
5.	Boonmee 2020	East Asia & Pacific	2.17	1.72	2.75	23.07	1616
6.	MarMinn 2021	East Asia & Pacific	3.86	1.96	7.60	16.5	507
**Overall (I2=77.2%, p<0.05)**			**1.97**	**1.213**	**3.2**		**3614**

Fig. 81: **Studies included in odds ratio of mortality (hypoxaemic vs. normoxaemic) among admitted adults with non-respiratory disease**

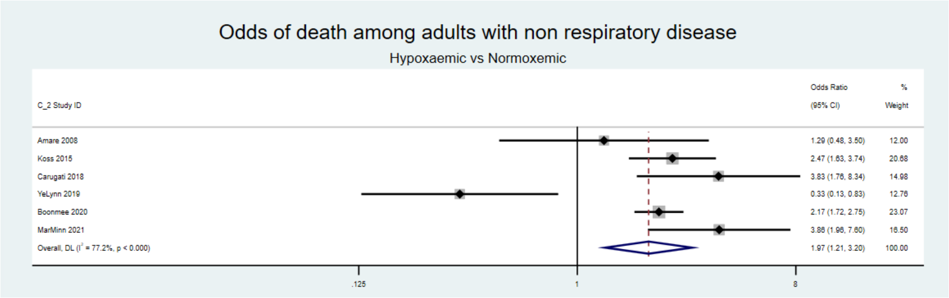

Relative Odds of Death – overall (neonates, children, and adults)

Table 82: **Studies included in odds ratio of overall mortality (hypoxaemic vs. normoxaemic)**

Serial	StudyID	Region	OR	LCL	UCL	Weight	Total
1.	Usen 1999	Sub Saharan Africa	5.10	2.22	11.70	1.21	1072
2.	West 1999	Sub Saharan Africa	2.71	0.77	9.56	0.85	118
3.	Duke 2002	East Asia & Pacific	3.01	1.26	7.18	1.17	491
4.	Ondoa-Onama 2003	Sub Saharan Africa	4.94	1.47	16.54	0.89	124
5.	Djelantik 2003	East Asia & Pacific	5.56	4.39	7.05	1.7	4306
6.	Maitland 2006	Sub Saharan Africa	1.52	1.02	2.28	1.6	718
7.	Junge 2006	Sub Saharan Africa	7.45	5.48	10.14	1.66	3269
8.	Asghar 2008	Mixed	2.55	1.34	4.85	1.38	958
9.	Nantanda 2008	Sub Saharan Africa	6.07	2.25	16.36	1.06	157
10.	Amare 2008	Sub Saharan Africa	1.29	0.48	3.50	1.06	119
11.	Mwaniki 2009	Sub Saharan Africa	6.37	5.47	7.43	1.74	15156
12.	Husain 2009	South Asia	5.73	0.28	117.65	0.25	30
13.	Sigauque 2009	Sub Saharan Africa	2.19	1.13	4.25	1.36	584
14.	Libster 2010	Latin America & Caribbean	0.32	0.10	1.04	0.92	251
15.	Ashraf 2010	South Asia	0.18	0.01	3.75	0.24	360
16.	Chisti 2011	South Asia	8.98	3.02	26.64	0.98	258
17.	Ramakrishna 2012	Sub Saharan Africa	6.52	2.49	17.07	1.09	233
18.	Chisti 2013	South Asia	22.67	5.98	85.92	0.8	140
19.	Cserti-Gazdewich 2013	Sub Saharan Africa	4.79	2.23	10.29	1.27	849
20.	McCollum 2013	Sub Saharan Africa	5.21	2.23	12.19	1.19	761
21.	Orimadegun 2013	Sub Saharan Africa	1.73	1.21	2.46	1.63	1726
22.	Kuti 2013	Sub Saharan Africa	3.05	1.05	8.87	1	389
23.	Tokman 2014	Sub Saharan Africa	3.67	1.54	8.75	1.17	241
24.	Orimadegun 2014	Sub Saharan Africa	4.71	2.16	10.27	1.25	369
25.	Koss 2015	Sub Saharan Africa	2.47	1.63	3.74	1.58	835
26.	Abdulkadir 2015	Sub Saharan Africa	61.84	3.66	1044.98	0.28	200
27.	Lowlaavar 2016	Sub Saharan Africa	2.88	1.72	4.81	1.5	1291
28.	Bassat 2016	Sub Saharan Africa	3.00	1.87	4.81	1.54	825
29.	Barennes 2016	East Asia & Pacific	4.45	1.74	11.39	1.11	350
30.	Benet 2017	Mixed	3.83	1.29	11.42	0.98	405
31.	Alwadhi 2017	South Asia	9.34	0.49	177.63	0.26	112
32.	Zampoli 2017	Sub Saharan Africa	7.78	1.01	59.81	0.46	206
33.	Hau 2018	Sub Saharan Africa	4.55	2.12	9.76	1.27	537
34.	Carugati 2018	Sub Saharan Africa	3.83	1.76	8.34	1.26	419
35.	Morgan 2018	Sub Saharan Africa	6.51	3.51	12.06	1.41	407
36.	Nabukeera-Barungi 2018	Sub Saharan Africa	3.27	1.22	8.76	1.07	400

37.	Worodria 2018	Sub Saharan Africa	3.70	2.72	5.03	1.66	1887
38.	Aston 2019	Sub Saharan Africa	5.38	2.99	9.70	1.43	431
39.	McCollum 2019	Sub Saharan Africa	1.02	0.64	1.63	1.54	644
40.	Dembele 2019	East Asia & Pacific	4.14	3.13	5.48	1.68	5023
41.	YeLynn 2019	East Asia & Pacific	0.33	0.13	0.83	1.11	118
42.	Ma 2019	Sub Saharan Africa	3.94	0.50	30.88	0.46	155
43.	Boonmee 2020	East Asia & Pacific	2.17	1.72	2.75	1.71	1616
44.	Hooli 2020	Sub Saharan Africa	4.60	2.32	9.14	1.34	1491
45.	Ojuawo 2020	Sub Saharan Africa	9.90	2.91	33.69	0.88	102
46.	Homayounieh 2020	Middle East & North Africa	2.63	0.81	8.46	0.92	75
47.	Shahunja 2020	South Asia	14.73	7.35	29.52	1.33	401
48.	Bepouka 2020	Sub Saharan Africa	15.32	3.51	66.96	0.72	141
49.	Wasingya-Kasereka 2020	Sub Saharan Africa	10.19	6.87	15.11	1.6	2599
50.	Fagbohun 2020	Latin America & Caribbean	11.07	6.23	19.66	1.45	860
51.	Mejia 2020	Latin America & Caribbean	5.80	3.42	9.83	1.49	500
52.	Olupot-Olupot 2020	Sub Saharan Africa	4.60	2.32	9.13	1.34	662
53.	Shahrin 2020	South Asia	4.53	1.44	14.27	0.94	176
54.	Rao 2021	South Asia	8.00	2.43	26.33	0.9	119
55.	Acar 2021	Europe & Central Asia	5.99	3.36	10.69	1.44	671
56.	Diaz-Velez 2021	Latin America & Caribbean	4.54	3.09	6.68	1.61	493
57.	Padmaprakash 2021	South Asia	86.51	47.82	156.49	1.43	1536
58.	Bui-Binh-Bao 2021	East Asia & Pacific	69.44	8.21	587.42	0.43	281
59.	Chisti 2021	South Asia	13.55	9.24	19.88	1.61	4007
60.	Anyaypoma-Ocon 2021	Latin America & Caribbean	1.11	0.72	1.71	1.57	324
61.	MarMinn 2021	East Asia & Pacific	3.86	1.96	7.60	1.35	507
62.	Sarfaraz 2021	South Asia	3.35	1.63	6.88	1.31	170
63.	Honarvar 2021	Middle East & North Africa	3.63	2.73	4.83	1.68	1975
64.	Kintwa 2021	East Asia & Pacific	10.49	3.62	30.39	1	140
65.	Kayambankadzanja 2021	Sub Saharan Africa	3.92	1.87	8.23	1.29	1135
66.	Xiong 2021	East Asia & Pacific	40.44	20.75	78.83	1.36	799
67.	Marcolino 2021	Latin America & Caribbean	3.13	2.37	4.14	1.68	1907
68.	Nemati 2021	Middle East & North Africa	1.31	0.83	2.06	1.55	946
69.	Oliveira 2022	Latin America & Caribbean	2.78	2.42	3.20	1.75	10911

70.	Krithika 2022	South Asia	191.45	76.61	478.48	1.13	600
71.	Awasthi 2022	South Asia	3.40	2.06	5.59	1.52	7196
72.	Arana-Calderon 2022	Latin America & Caribbean	26.86	3.43	210.29	0.46	158
73.	Siqueira 2022	Latin America & Caribbean	4.46	4.02	4.96	1.76	15105
74.	AbdelGhaffar 2022	Middle East & North Africa	7.84	6.64	9.26	1.74	3712
75.	Araban 2022	Middle East & North Africa	5.20	4.00	6.77	1.69	3181
76.	Kiputa 2022	Sub Saharan Africa	6.18	3.51	10.87	1.45	348
77.	Chisti 2022	South Asia	14.60	9.77	21.83	1.59	3468
78.	King 2022	Sub Saharan Africa	6.38	2.83	14.37	1.22	802
79.	Soto 2022	Latin America & Caribbean	5.12	3.99	6.58	1.7	1323
80.	Kapoor 2022	South Asia	16.95	3.74	76.92	0.7	180
81.	Alizadehsani 2022	Middle East & North Africa	6.48	3.07	13.67	1.29	600
**Overall (I2=89.5%, p<0.05)**			**4.836**	**4.113**	**5.685**		**118141**

Fig. 82: **Studies included in odds ratio of overall mortality (hypoxaemic vs. normoxaemic)**

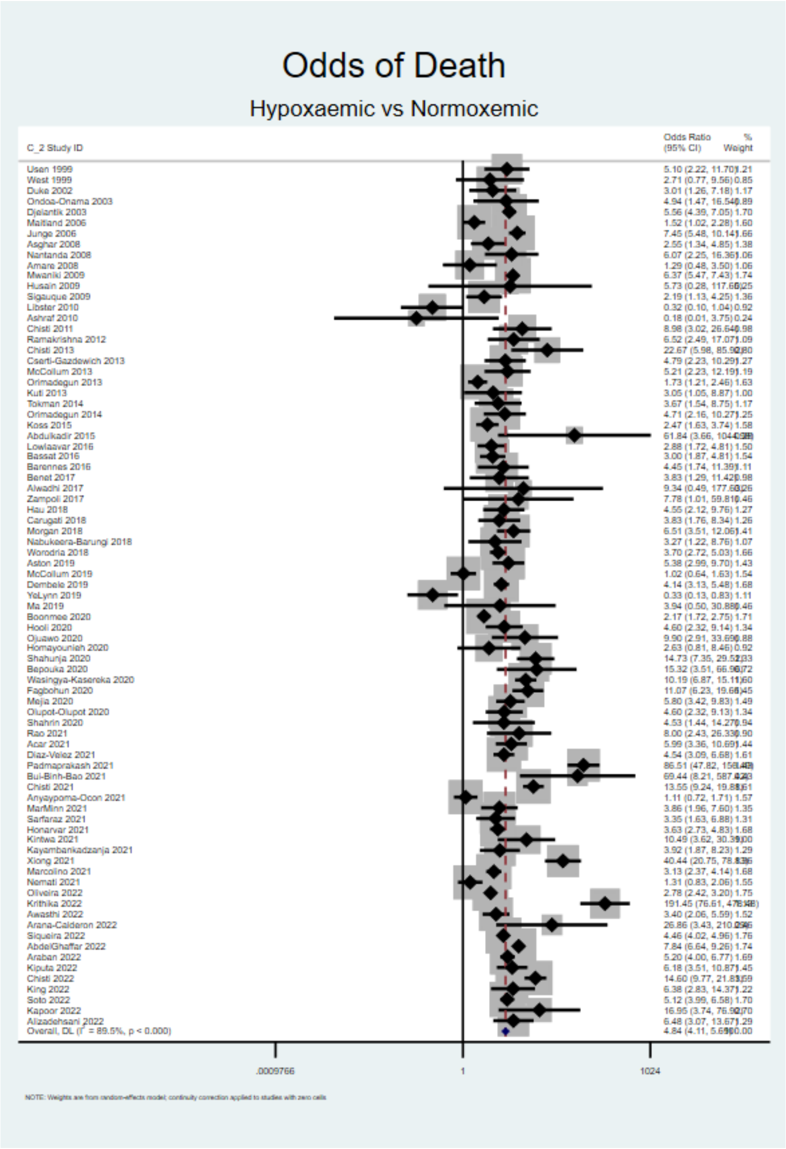
Odds of Death
Hypoxaemic vs Normoxemic
C_2 Study ID
Odds Ratio (95% CI)
% Weight
Usen 1999 5.10 (2.22, 11.70) 1.21
West 1999 2.71 (0.77, 9.56) 0.85
Duke 2002 3.01 (1.26, 7.18) 1.17
Ondoa-Onama 2003 4.94 (1.47, 16.54) 0.89
Djelantik 2003 5.56 (4.39, 7.05) 1.70
Maitland 2006 1.52 (1.02, 2.28) 1.60
Junge 2006 7.45 (5.48, 10.14) 1.66
Asghar 2008 2.55 (1.34, 4.85) 1.38
Nantanda 2008 6.07 (2.25, 16.36) 1.06
Amare 2008 1.29 (0.48, 3.50) 1.06
Mwaniki 2009 6.37 (5.47, 7.43) 1.74
Husain 2009 5.73 (0.28, 117.6
Sigauque 2009 2.19 (1.13, 4.25) 1.36
Libster 2010 0.32 (0.10, 1.04) 0.92
Ashraf 2010 0.18 (0.01, 3.75) 0.24
Chisti 2011 8.98 (3.02, 26.64) 0.98
Ramakrishna 2012 6.52 (2.49, 17.07) 1.09
Chisti 2013 22.67 (5.98, 85.9
Cserti-Gazdewich 2013 4.79 (2.23, 10.29) 1.27
McCollum 2013 5.21 (2.23, 12.19) 1.19
Orimadegun 2013 1.73 (1.21, 2.46) 1.63
Kuti 2013 3.05 (1.05, 8.87) 1.00
Tokman 2014 3.67 (1.54, 8.75) 1.17
Orimadegun 2014 4.71 (2.16, 10.27) 1.25
Koss 2015 2.47 (1.63, 3.74) 1.58
Abdulkadir 2015 61.84 (3.66, 104
Lowlaavar 2016 2.88 (1.72, 4.81) 1.50
Bassat 2016 3.00 (1.87, 4.81) 1.54
Barennes 2016 4.45 (1.74, 11.39) 1.11
Benet 2017 3.83 (1.29, 11.42) 0.98
Alwadhi 2017 9.34 (0.49, 177.6
Zampoli 2017 7.78 (1.01, 59.81) 0.46
Hau 2018 4.55 (2.12, 9.76) 1.27
Carugati 2018 3.83 (1.76, 8.34) 1.26
Morgan 2018 6.51 (3.51, 12.06) 1.41
Nabukeera-Barungi 2018 3.27 (1.22, 8.76) 1.07
Worodria 2018 3.70 (2.72, 5.03) 1.66
Aston 2019 5.38 (2.99, 9.70) 1.43
McCollum 2019 1.02 (0.64, 1.63) 1.54
Dembele 2019 4.14 (3.13, 5.48) 1.68
YeLynn 2019 0.33 (0.13, 0.83) 1.11
Ma 2019 3.94 (0.50, 30.88) 0.46
Boonmee 2020 2.17 (1.72, 2.75) 1.71
Hooli 2020 4.60 (2.32, 9.14) 1.34
Ojuawo 2020 9.90 (2.91, 33.69) 0.88
Homayounieh 2020 2.63 (0.81, 8.46) 0.92
Shahunja 2020 14.73 (7.35, 29.5
Bepouka 2020 15.32 (3.51, 66.9
Wasingya-Kasereka 2020 10.19 (6.87, 15.11
Fagbohun 2020 11.07 (6.23, 19.6
Mejia 2020 5.80 (3.42, 9.83) 1.49
Olupot-Olupot 2020 4.60 (2.32, 9.13) 1.34
Shahrin 2020 4.53 (1.44, 14.27) 0.94
Rao 2021 8.00 (2.43, 26.33) 0.90
Acar 2021 5.99 (3.36, 10.69) 1.44
Diaz-Velez 2021 4.54 (3.09, 6.68) 1.61
Padmaprakash 2021 86.51 (47.82, 15
Bui-Binh-Bao 2021 69.44 (8.21, 587
Chisti 2021 13.55 (9.24, 19.8
Anyaypoma-Ocon 2021 1.11 (0.72, 1.71) 1.57
MarMinn 2021 3.86 (1.96, 7.60) 1.35
Sarfaraz 2021 3.35 (1.63, 6.88) 1.31
Honarvar 2021 3.63 (2.73, 4.83) 1.68
Kintwa 2021 10.49 (3.62, 30.3
Kayambankadzanja 2021 3.92 (1.87, 8.23) 1.29
Xiong 2021 40.44 (20.75, 78.8
Marcolino 2021 3.13 (2.37, 4.14) 1.68
Nemati 2021 1.31 (0.83, 2.06) 1.55
Oliveira 2022 2.78 (2.42, 3.20) 1.75
Krithika 2022 191.45 (76.61, 47
Awasthi 2022 3.40 (2.06, 5.59) 1.52
Arana-Calderon 2022 26.86 (3.43, 210.
Siqueira 2022 4.46 (4.02, 4.96) 1.76
AbdelGhaffar 2022 7.84 (6.64, 9.26) 1.74
Araban 2022 5.20 (4.00, 6.77) 1.69
Kiputa 2022 6.18 (3.51, 10.87) 1.45
Chisti 2022 14.60 (9.77, 21.8
King 2022 6.38 (2.83, 14.37) 1.22
Soto 2022 5.12 (3.99, 6.58) 1.70
Kapoor 2022 16.95 (3.74, 76.9
Alizadehsani 2022 6.48 (3.07, 13.67) 1.29
Overall, DL (I^2 = 89.5%, p < 0.000) 4.84 (4.11, 5.6
.0009766
1
1024
NOTE: Weights are from random-effects model; continuity correction applied to studies with zero cells

Sensitivity Analysis (SpO2<90%) Results tables and Forest plots

Admitted Neonates - all

Table 83: Studies included in prevalence of hypoxaemia among all admitted neonates (SpO2<90)

Serial No	Study ID	WB Region	Proportion	LCL	UCL	Weight	Denominator
1	Duke 2002	East Asia & Pacific	43.18	34.59	52.08	8.58	132
2	English 2003	Sub Saharan Africa	19.97	16.88	23.35	10.29	616
3	Junge 2006	Sub Saharan Africa	16.45	12.50	21.06	9.76	310
4	Mwaniki 2009	Sub Saharan Africa	18.64	16.39	21.07	10.54	1105
5	Orimadegun 2013	Sub Saharan Africa	41.41	36.84	46.09	10.09	454
6	McCollum 2013	Sub Saharan Africa	20.59	8.70	37.90	5.37	34
7	Morgan 2018	Sub Saharan Africa	29.24	24.86	33.92	10.01	407
8	Graham 2019	Sub Saharan Africa	18.24	17.37	19.13	10.82	7473
9	King 2022	Sub Saharan Africa	39.29	21.50	59.42	4.85	28
10	Kiputa 2022	Sub Saharan Africa	21.26	17.08	25.94	9.87	348
11	Graham 2022	Sub Saharan Africa	16.26	12.42	20.72	9.81	326
**Overall (I2=94.49%, T2=0.03, p<0.05)**			**24.49**	**19.92**	**29.35**		**11233**

Fig. **83: Studies included in prevalence of hypoxaemia among all admitted neonates (SpO2<90)**

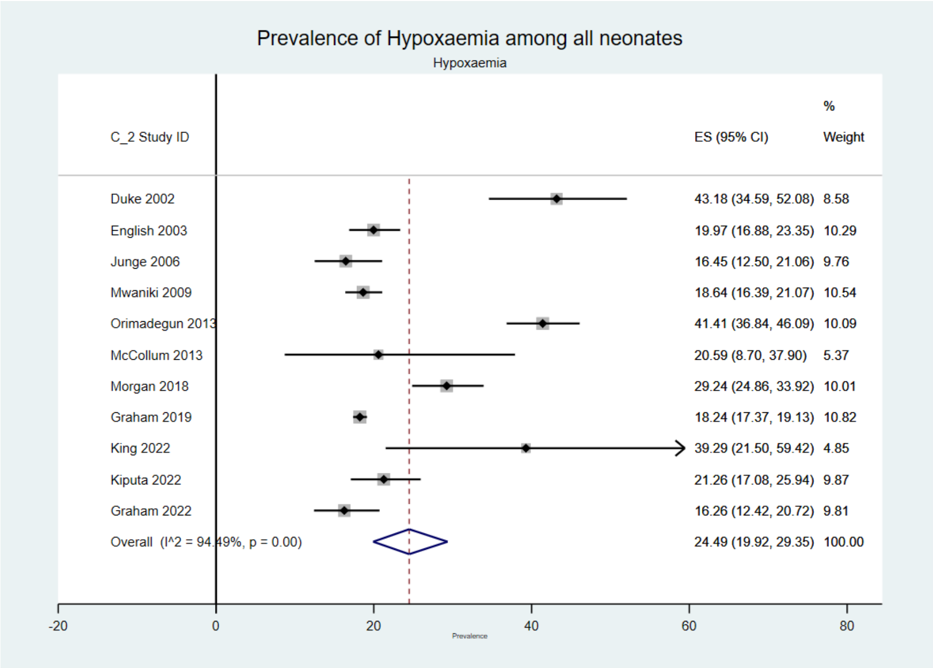

Admitted Neonates – neonatal encephalopathy / birth asphyxia

Table 84: Studies included in prevalence of hypoxaemia among admitted neonates with birth asphyxia (SpO2<90)

Serial	Study ID	WB Region	Proportion	LCL	UCL	Weight	Denominator
1	Duke 2002	East Asia & Pacific	60.00	38.67	78.87	27.28	25
2	Ondoa-Onama 2003	Sub Saharan Africa	14.68	8.63	22.74	34.84	109
3	Graham 2019	Sub Saharan Africa	33.40	31.54	35.30	37.88	2458
**Overall (I2=92.78%, T2=0.10, p<0.05)**			**32.79**	**16.23**	**51.83**		**2592**

Fig. 84: Studies included in prevalence of hypoxaemia among admitted neonates with birth asphyxia (SpO2<90)

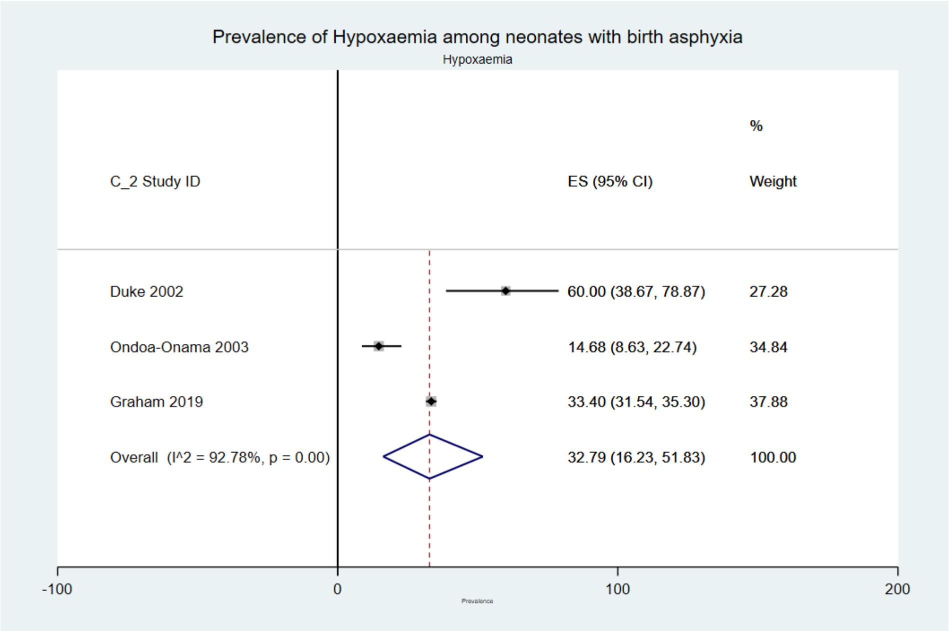
Prevalence of Hypoxaemia among neonates with birth asphyxia
Hypoxaemia
%
C_2 Study ID
ES (95% CI)
Weight
Duke 2002
60.00 (38.67, 78.87)
27.28
Ondoa-Onama 2003
14.68 (8.63, 22.74)
34.84
Graham 2019
33.40 (31.54, 35.30)
37.88
Overall (I^2 = 92.78%, p = 0.00)
32.79 (16.23, 51.83)
100.00
-100
0
100
200

Admitted Neonates – pneumonia

Table 85: Studies included in prevalence of hypoxaemia among admitted neonates with pneumonia (SpO2<90)

Serial No	Study ID	WB Region	Proportion	LCL	UCL	Weight	Denominator
1	Duke 2002	East Asia & Pacific	77.27	54.63	92.18	31.59	22
2	Dembele 2019	East Asia & Pacific	11.88	9.65	14.42	35.04	749
3	LeRoux 2021	Sub Saharan Africa	31.91	19.09	47.12	33.37	47
**Overall (I2=96.25%, T2=0.39, p<0.05)**			**37.33**	**7.60**	**73.50**		**818**

Fig. 85: Studies included in prevalence of hypoxaemia among admitted neonates with pneumonia (SpO2<90)

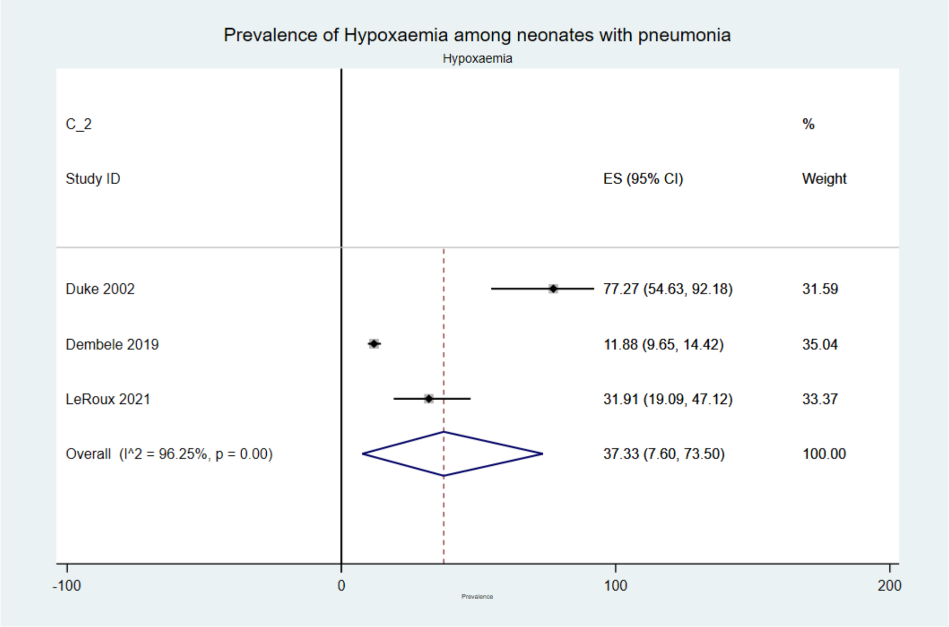

Admitted Neonates - prematurity

Table 86: Studies included in prevalence of hypoxaemia among admitted neonates with prematurity (SpO2<90)

Serial	Study ID	WB Region	Proportion	LCL	UCL	Weight	Denominator
1	Duke 2002	East Asia & Pacific	30.30	15.59	48.71	27.66	33
2	Morgan 2018	Sub Saharan Africa	48.28	37.42	59.25	33.70	87
3	Graham 2019	Sub Saharan Africa	25.80	23.60	28.09	38.64	1500
**Overall (I2=89.23%, T2=0.07, p<0.05)**			**34.29**	**19.50**	**50.77**		**1620**

Fig. 86: Studies included in prevalence of hypoxaemia among admitted neonates with prematurity (SpO2<90)

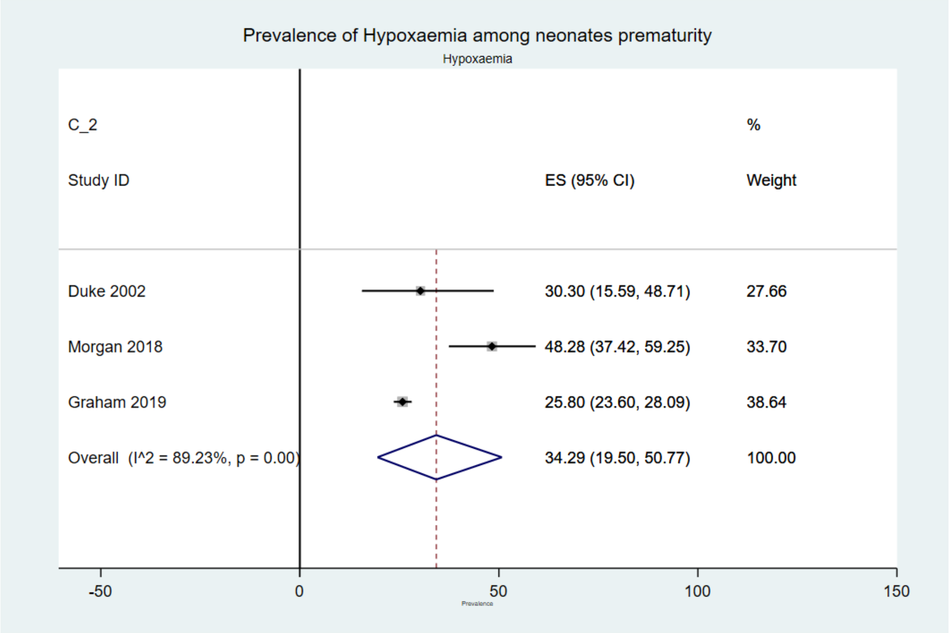

Admitted Neonates - sepsis

Table 87: Studies included prevalence of hypoxaemia among admitted neonates with sepsis (SpO2<90)

Serial No	Study ID	WB Region	Proportion	LCL	UCL	Weight	Denominator
1	Duke 2002	East Asia & Pacific	44.12	27.19	62.11	1.07	34
2	Graham 2019	Sub Saharan Africa	21.00	19.60	22.46	98.93	3195

Fig. 87: Studies included prevalence of hypoxaemia among admitted neonates with sepsis (SpO2<90)

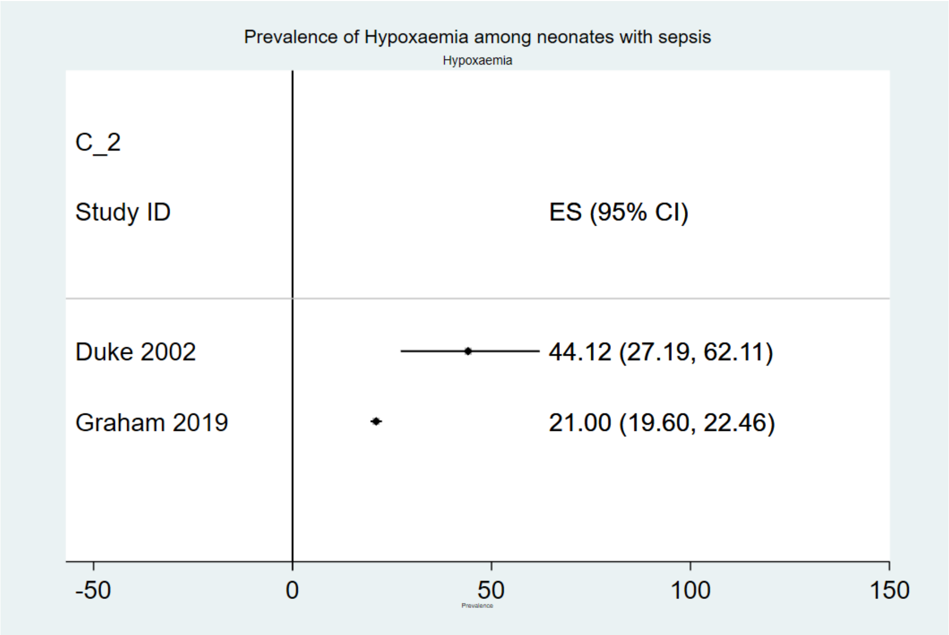

Admitted Children – all

Table 88: Studies included in prevalence of hypoxaemia among all admitted children (SpO2<90)

Serial	Study ID	WB Region	Proportion	LCL	UCL	Weight	Denominator
1	Duke 2002	East Asia & Pacific	55.71	50.40	60.92	4.99	359
2	English 2003	Sub Saharan Africa	14.04	10.27	18.56	4.86	292
3	Djelantik 2003	East Asia & Pacific	11.19	10.27	12.17	5.58	4306
4	Wandi 2006	East Asia & Pacific	24.16	22.24	26.15	5.50	1896
5	Junge 2006	Sub Saharan Africa	5.75	4.98	6.60	5.56	3269
6	Mwaniki 2009	Sub Saharan Africa	5.26	4.88	5.65	5.62	13183
7	Foran 2010	Sub Saharan Africa	6.02	1.98	13.50	3.60	83
8	Orimadegun 2013	Sub Saharan Africa	24.06	21.73	26.50	5.44	1272
9	McCollum 2013	Sub Saharan Africa	4.54	3.14	6.32	5.30	727
10	Lowlaavar 2016	Sub Saharan Africa	25.72	23.35	28.19	5.44	1291
11	Barennes 2016	East Asia & Pacific	13.71	10.29	17.77	4.97	350
12	Nielsen 2018	Latin America & Caribbean	8.58	6.91	10.50	5.38	991
13	Hau 2018	Sub Saharan Africa	5.59	3.80	7.88	5.18	537
14	Graham 2019	Sub Saharan Africa	7.93	7.52	8.35	5.62	16453
15	Enoch 2019	Sub Saharan Africa	10.00	9.51	10.50	5.62	14232
16	Tuti 2021	Sub Saharan Africa	6.97	6.77	7.17	5.64	64722
17	Krithika 2022	South Asia	8.17	6.10	10.65	5.23	600
18	King 2022	Sub Saharan Africa	7.69	5.92	9.79	5.32	780
19	Graham 2022	Sub Saharan Africa	11.26	8.64	14.35	5.16	506
**Overall (I2=99.03%, T2=0.02, p<0.05)**			**12.13**	**9.99**	**14.43**		**125849**

Fig. 88: Studies included in prevalence of hypoxaemia among all admitted children (SpO2<90)

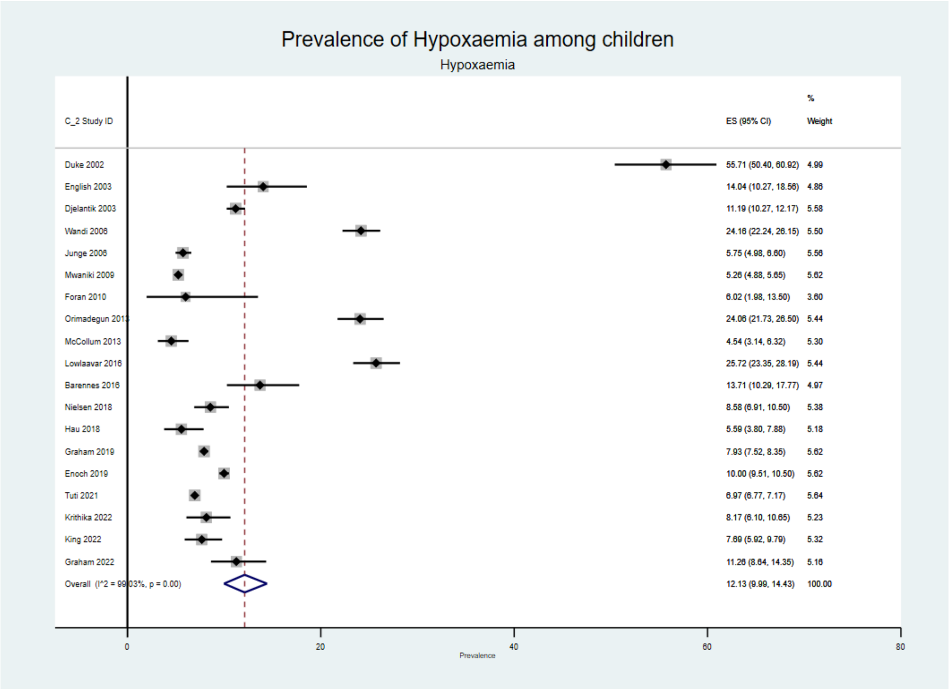

Admitted Children - fever

Table 89: Studies included in prevalence of hypoxaemia among admitted children with fever (SpO2<90)

Serial	Study ID	WB Region	Proportion	LCL	UCL	Weight	Denominator
1	Usen 1999	Sub Saharan Africa	14.86	7.66	25.04	32.13	74
2	Bassat 2016	Sub Saharan Africa	27.75	24.56	31.11	33.86	746
3	Leligdowicz 2021	Sub Saharan Africa	4.13	3.38	4.99	34.00	2469
**Overall (I2=99.30%, T2=0.22, p<0.05)**			**14.06**	**1.12**	**37.26**		**3289**

Fig. 89: Studies included in prevalence of hypoxaemia among admitted children with fever (SpO2<90)

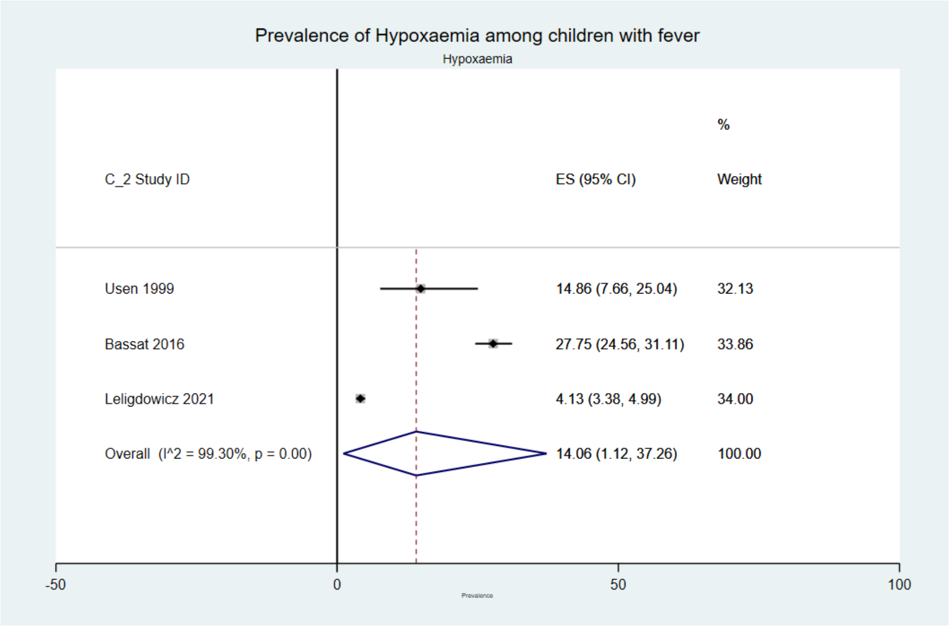

Admitted Children – pneumonia all WHO-classified

Table 90: Studies included in prevalence of hypoxaemia among admitted children with pneumonia (WHO-classification) (SpO2<90)

Sl no	Study ID	WB Region	Proportion	LCL	UCL	Weight	Denominator
1.	Usen 1999	Sub Saharan Africa	5.88	4.55	7.46	1.99	1072
2.	West 1999	Sub Saharan Africa	43.68	36.52	51.05	1.93	190
3.	Addo-Yobo 2004	Mixed	19.10	17.25	21.04	1.99	1702
4.	Laman 2005	East Asia & Pacific	25.97	16.64	37.23	1.84	77
5.	Wandi 2006	East Asia & Pacific	54.50	50.34	58.61	1.98	578
6.	Fu 2006	Mixed	19.30	17.45	21.26	1.99	1694
7.	Junge 2006	Sub Saharan Africa	11.70	8.83	15.09	1.97	436
8.	Puumalainen 2008	East Asia & Pacific	16.16	14.08	18.42	1.99	1151
9.	Duke 2008	East Asia & Pacific	57.09	52.94	61.17	1.98	578
10	Asghar 2008	Mixed	64.61	61.49	67.65	1.99	958
11	Mwaniki 2009	Sub Saharan Africa	8.40	7.68	9.16	2.00	5489
12	Sigauque 2009	Sub Saharan Africa	25.84	22.60	29.29	1.98	685
13	Chisti 2011	South Asia	54.55	47.33	61.62	1.94	198
14	Webb 2012	Sub Saharan Africa	23.59	20.16	27.30	1.98	568
15	Muller 2012	Latin America & Caribbean	22.48	20.66	24.38	2.00	1993
16	Ramakrishna 2012	Sub Saharan Africa	37.34	31.11	43.89	1.95	233
17	Jain 2013	South Asia	17.68	12.42	24.03	1.93	181
18	Kuti 2013	Sub Saharan Africa	19.76	16.01	23.94	1.97	410
19	McCollum 2013	Sub Saharan Africa	17.22	11.57	24.20	1.92	151
20	Orimadegun 2013	Sub Saharan Africa	49.20	43.53	54.88	1.96	313
21	Kuti 2013	Sub Saharan Africa	19.29	15.62	23.39	1.97	420
22	Sempertegui 2014	Latin America & Caribbean	85.11	81.48	88.27	1.97	450
23	Ibraheem 2014	Sub Saharan Africa	41.50	34.59	48.66	1.94	200
24	Abdulkadir 2015	Sub Saharan Africa	41.50	34.59	48.66	1.94	200
25	Breiman 2015	Sub Saharan Africa	35.74	33.88	37.63	2.00	2563
26	Kelly 2015	Sub Saharan Africa	38.66	32.43	45.16	1.95	238
27	Basnet 2015	South Asia	61.15	57.15	65.04	1.98	610
28	Salah 2015	Sub Saharan Africa	42.67	34.64	50.99	1.92	150
29	Bassat 2016	Sub Saharan Africa	27.88	24.84	31.07	1.99	825
30	Alwadhi 2017	South Asia	50.89	41.27	60.46	1.89	112
31	Graham 2019	Sub Saharan Africa	23.44	21.64	25.33	2.00	2073
32	McCollum 2019	Sub Saharan Africa	64.44	60.61	68.14	1.98	644
33	Ashraf 2019	South Asia	10.64	8.00	13.78	1.97	470
34	Dembele 2019	East Asia & Pacific	13.52	12.50	14.59	2.00	4179
35	Ma 2019	Sub Saharan Africa	85.81	79.30	90.89	1.92	155
36	Fashanu 2020	Sub Saharan Africa	58.38	55.84	60.90	1.99	1497
37	Hooli 2020	Sub Saharan Africa	14.69	12.93	16.59	1.99	1491
38	Fagbohun 2020	Latin America & Caribbean	13.02	10.85	15.46	1.99	860

39	Shahrin 2020	South Asia	15.18	10.41	21.07	1.93	191
40	Muro 2020	Sub Saharan Africa	32.40	26.64	38.58	1.95	250
41	Oktaria 2021	East Asia & Pacific	13.53	8.22	20.54	1.91	133
42	Bui-Binh-Bao 2021	East Asia & Pacific	11.39	7.92	15.69	1.96	281
43	Chisti 2021	South Asia	28.13	26.74	29.55	2.00	4007
44	Rahman 2021	South Asia	39.98	38.11	41.88	2.00	2646
45	Ahmed 2022	Sub Saharan Africa	75.32	64.18	84.44	1.84	77
46	Awasthi 2022	South Asia	35.85	34.74	36.97	2.00	7196
47	Chisti 2022	South Asia	31.23	29.69	32.80	2.00	3468
48	Jullien 2022	South Asia	75.51	67.74	82.22	1.91	147
49	Kapoor 2022	South Asia	36.11	29.10	43.59	1.93	180
50	Mvalo 2022	Sub Saharan Africa	63.75	59.53	67.82	1.98	538
51	Zar 2022	Sub Saharan Africa	24.38	18.61	30.92	1.94	201
Overall (I2=99.35%, T2=0.15, p<0.05)			34.10	29.15	39.22		55109

Fig. 90: Studies included in prevalence of hypoxaemia among admitted children with pneumonia (WHO-classification) (SpO2<90)

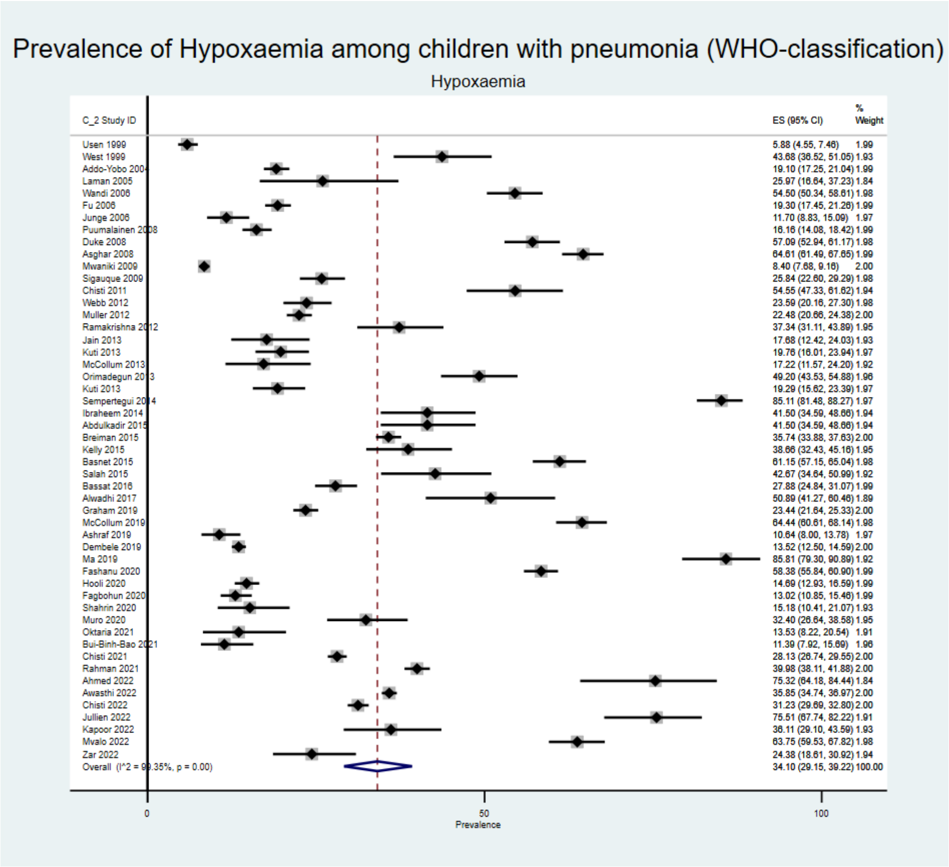

Admitted Children – pneumonia severe (WHO classification severe or very severe)

Table 91: Studies included in Prevalence of hypoxaemia among admitted children with pneumonia (WHO-severe) (SpO2<90)

Sl no	Study ID	WB Region	Proportion	LCL	UCL	Weight	Denominator
1.	Addo-Yobo 2004	Mixed	19.10	17.25	21.04	3.76	1702
2.	Laman 2005	East Asia & Pacific	25.97	16.64	37.23	3.52	77
3.	Fu 2006	Mixed	19.30	17.45	21.26	3.76	1694
4.	Puumalainen 2008	East Asia & Pacific	19.54	16.94	22.34	3.74	865
5.	Asghar 2008	Mixed	64.61	61.49	67.65	3.75	958
6.	Mwaniki 2009	Sub Saharan Africa	9.33	8.52	10.19	3.76	4792
7.	Sigauque 2009	Sub Saharan Africa	25.84	22.60	29.29	3.74	685
8.	Webb 2012	Sub Saharan Africa	23.59	20.16	27.30	3.73	568
9.	Ramakrishna 2012	Sub Saharan Africa	37.34	31.11	43.89	3.68	233
10	Kuti 2013	Sub Saharan Africa	19.76	16.01	23.94	3.72	410
11	McCollum 2013	Sub Saharan Africa	17.39	11.47	24.76	3.62	138
12	Orimadegun 2013	Sub Saharan Africa	49.20	43.53	54.88	3.70	313
13	Kuti 2013	Sub Saharan Africa	19.29	15.62	23.39	3.72	420
14	Sempertegui 2014	Latin America & Caribbean	85.11	81.48	88.27	3.72	450
15	Breiman 2015	Sub Saharan Africa	35.74	33.88	37.63	3.76	2563
16	Basnet 2015	South Asia	61.15	57.15	65.04	3.73	610
17	Salah 2015	Sub Saharan Africa	48.05	36.52	59.74	3.52	77
18	Bassat 2016	Sub Saharan Africa	27.88	24.84	31.07	3.74	825
19	Alwadhi 2017	South Asia	50.89	41.27	60.46	3.59	112
20	Graham 2019	Sub Saharan Africa	36.09	33.13	39.12	3.75	1017
21	McCollum 2019	Sub Saharan Africa	64.44	60.61	68.14	3.74	644
22	Dembele 2019	East Asia & Pacific	13.52	12.50	14.59	3.76	4179
23	Shahrin 2020	South Asia	15.18	10.41	21.07	3.66	191
24	Muro 2020	Sub Saharan Africa	32.40	26.64	38.58	3.69	250
25	Rahman 2021	South Asia	39.98	38.11	41.88	3.76	2646
26	Kapoor 2022	South Asia	36.11	29.10	43.59	3.66	180
27	Mvalo 2022	Sub Saharan Africa	63.75	59.53	67.82	3.73	538
Overall (I2=99.42%, T2=0.18, p<0.05)			34.68	27.20	42.55		27137

Fig. 91: Studies included in Prevalence of hypoxaemia among admitted children with pneumonia (WHO-severe) (SpO2<90)

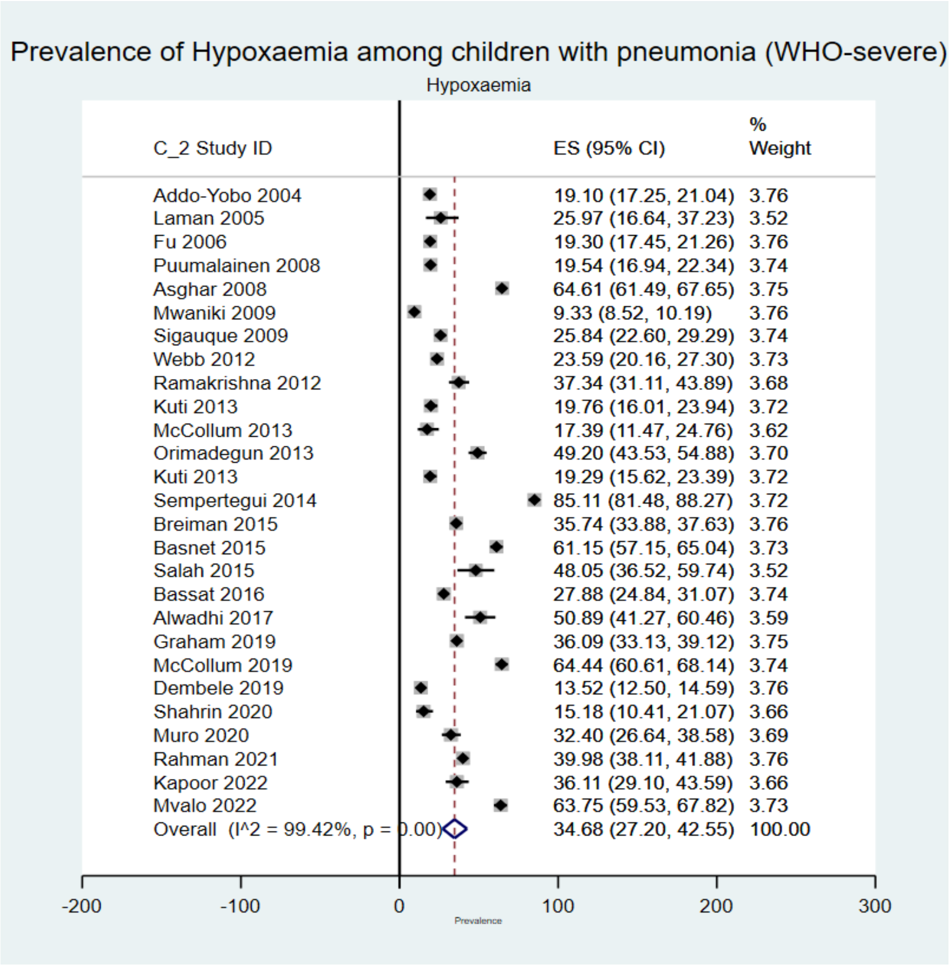

Admitted Children – pneumonia non-severe (WHO classification)

Table 92: Studies included in Prevalence of hypoxaemia among admitted children with pneumonia (WHO-non-severe) (SpO2<90)

Serial	Study ID	WB Region	Proportion	LCL	UCL	Weight	Denominator
1	Puumalainen 2008	East Asia & Pacific	5.94	3.50	9.35	13.21	286
2	Mwaniki 2009	Sub Saharan Africa	2.01	1.10	3.35	13.43	697
3	McCollum 2013	Sub Saharan Africa	15.38	1.92	45.45	8.37	13
4	Salah 2015	Sub Saharan Africa	36.99	25.97	49.09	12.20	73
5	Nemani 2016	South Asia	19.80	12.54	28.91	12.55	101
6	Bassat 2016	Sub Saharan Africa	27.88	24.84	31.07	13.46	825
7	Graham 2019	Sub Saharan Africa	16.60	13.95	19.53	13.44	717
8	Ashraf 2019	South Asia	10.64	8.00	13.78	13.35	470
**Overall (I2=97.75%, T2=0.12, p<0.05)**			**14.90**	**6.98**	**25.00**		**3182**

Fig. 92: Studies included in Prevalence of hypoxaemia among admitted children with pneumonia (WHO-non-severe) (SpO2<90)

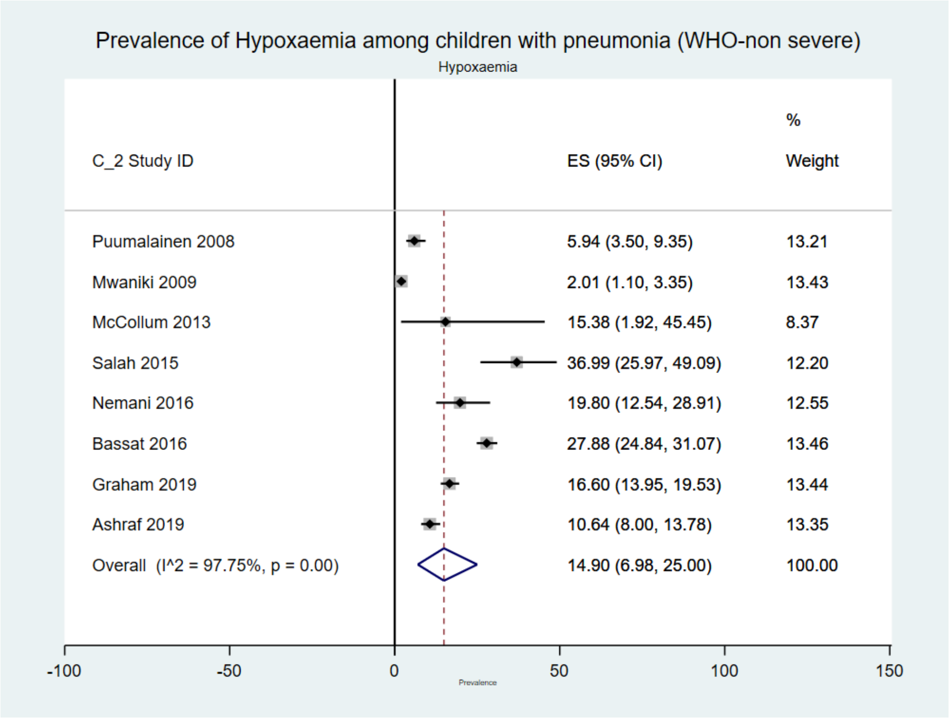

Admitted Children – pneumonia radiological

Table 93: Studies included in prevalence of hypoxaemia among admitted children with pneumonia (radiological) (SpO2<90)

Serial	Study ID	WB Region	Proportion	LCL	UCL	Weight	Denominator
1	Usen 1999	Sub Saharan Africa	7.06	3.70	12.01	6.71	170
2	Magree 2005	East Asia & Pacific	64.41	50.87	76.45	6.45	59
3	Wandi 2006	East Asia & Pacific	54.50	50.34	58.61	6.82	578
4	Bruce 2007	Latin America & Caribbean	62.50	51.53	72.60	6.58	88
5	Tiewsoh 2009	South Asia	68.50	59.67	76.45	6.66	127
6	Martinez-Medina 2010	Latin America & Caribbean	56.45	43.26	69.01	6.47	62
7	Chisti 2013	South Asia	11.08	7.92	14.95	6.78	334
8	Bassat 2016	Sub Saharan Africa	36.68	31.38	42.23	6.78	319
9	Benet 2017	Mixed	17.28	13.73	21.33	6.80	405
10	Alwadhi 2017	South Asia	53.76	43.12	64.16	6.59	93
11	PERCHStudyGroup 2019	Mixed	44.55	42.32	46.80	6.85	1935
12	Merida-Vieyra 2019	Latin America & Caribbean	96.10	91.71	98.56	6.70	154
13	Fagbohun 2020	Latin America & Caribbean	13.02	10.85	15.46	6.83	860
14	Saleh 2022	Middle East & North Africa	26.67	20.36	33.76	6.72	180
15	Jullien 2022	South Asia	82.05	66.47	92.46	6.26	39
**Overall (I2=98.81%, T2=0.26, p<0.05)**			**45.32**	**32.44**	**58.52**		**5403**

Fig. 93: Studies included in prevalence of hypoxaemia among admitted children with pneumonia (radiological) (SpO2<90)

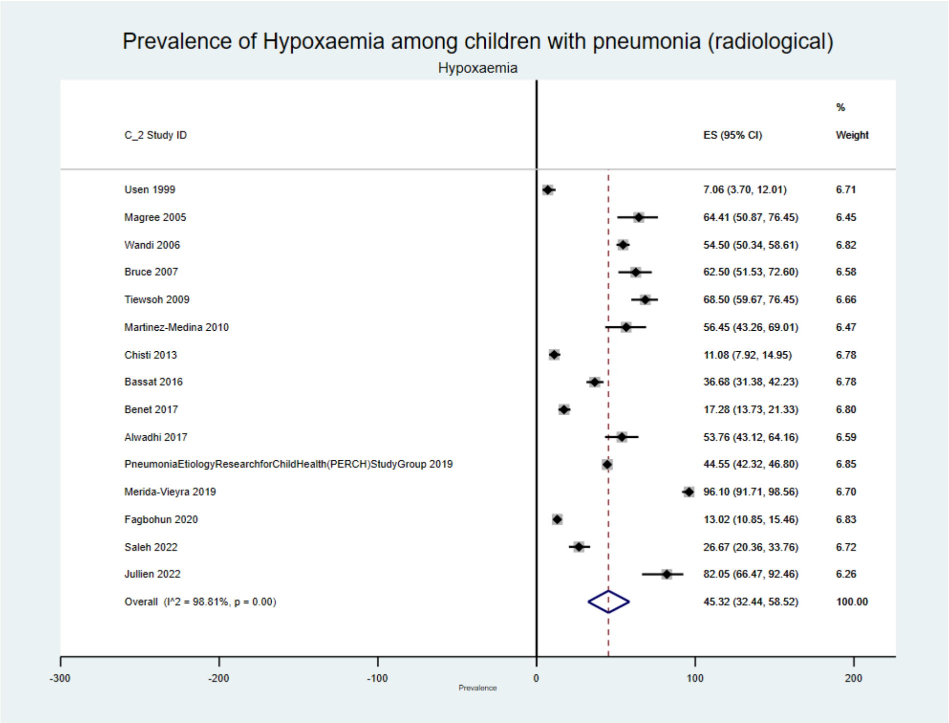

Admitted Children - bronchiolitis

Table 94: Studies included in Prevalence of hypoxaemia among admitted children with bronchiolitis (SpO2<90)

Serial No	Study ID	WB Region	Proportion	LCL	UCL	Weight	Denominator
1	Chan 2002	East Asia & Pacific	14.35	9.96	19.75	25.25	216
2	HongXIAO 2010	East Asia & Pacific	0.00	0.00	11.22	24.10	31
3	Libster 2010	Latin America & Caribbean	82.07	76.76	86.61	25.28	251
4	Nyawanda 2016	Sub Saharan Africa	25.74	21.85	29.95	25.36	470
**Overall (I2=99.16%, T2=0.56, p<0.05)**			**26.31**	**2.38**	**62.75**		**968**

Fig. 94: Studies included in Prevalence of hypoxaemia among admitted children with bronchiolitis (SpO2<90)

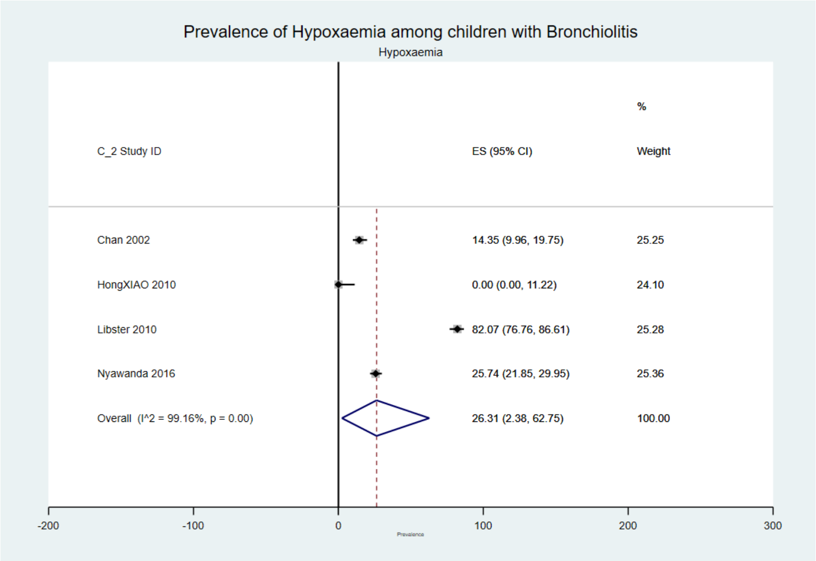

Admitted Children – pneumonia unspecified

Table 95: Studies included in prevalence of hypoxaemia among admitted children with pneumonia (unspecified) (SpO2<90)

Serial No	Study ID	WB Region	Proportion	LCL	UCL	Weight	Denominator
1	Magree 2005	East Asia & Pacific	16.53	12.13	21.75	14.27	248
2	Riccetto 2006	Latin America & Caribbean	22.15	15.76	29.67	13.89	149
3	Nyawanda 2016	Sub Saharan Africa	16.76	15.70	17.86	14.86	4696
4	Zampoli 2017	Sub Saharan Africa	70.87	64.16	76.98	14.15	206
5	Ling 2020	East Asia & Pacific	20.00	15.91	24.62	14.44	345
6	AbdelGhaffar 2022	Middle East & North Africa	33.13	31.50	34.80	14.85	3172
7	King 2022	Sub Saharan Africa	29.63	21.23	39.18	13.54	108
**Overall (I2=98.78%, T2=0.09, p<0.05)**			**28.94**	**19.14**	**39.84**		**8924**

Fig. 95: Studies included in prevalence of hypoxaemia among admitted children with pneumonia (unspecified) (SpO2<90)

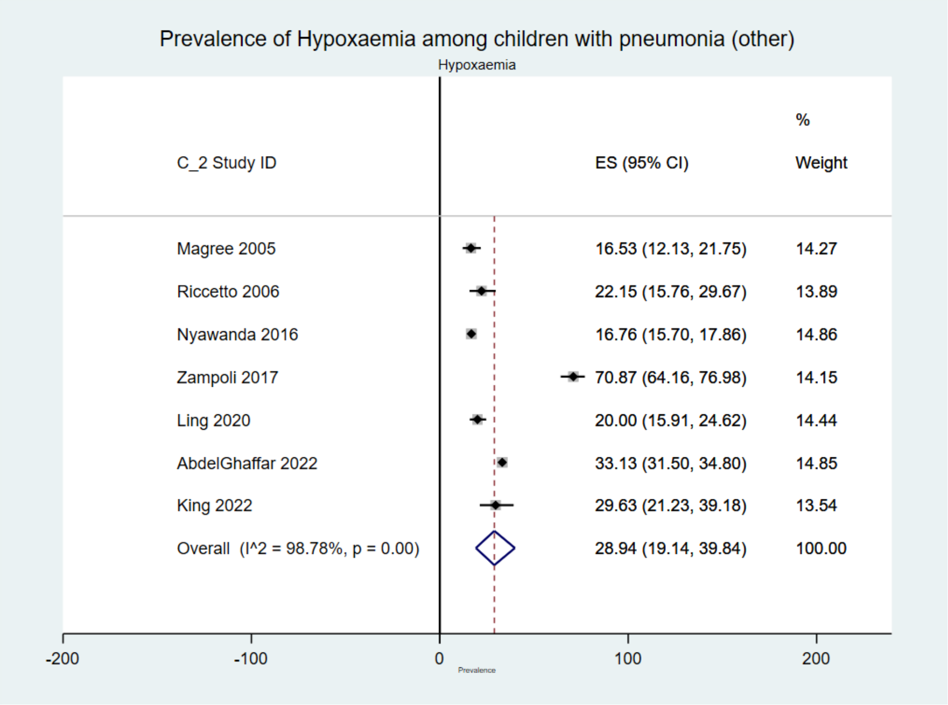

Admitted Children - anaemia

Table 96 : Studies included in prevalence of hypoxaemia among admitted children with anaemia (SpO2<90)

Serial No	Study ID	WB Region	Proportion	LCL	UCL	Weight	Denominator
1	Wandi 2006	East Asia & Pacific	3.17	0.39	11.00	16.23	63
2	Junge 2006	Sub Saharan Africa	1.78	0.49	4.49	17.53	225
3	Mwaniki 2009	Sub Saharan Africa	4.18	2.84	5.92	17.91	717
4	McCollum 2013	Sub Saharan Africa	3.23	0.08	16.70	14.69	31
5	Orimadegun 2014	Sub Saharan Africa	28.47	23.33	34.06	17.65	288
6	King 2022	Sub Saharan Africa	12.73	5.27	24.48	15.99	55
**Overall (I2=96.14%, T2=0.14, p<0.05)**			**7.39**	**1.14**	**17.71**		**1379**

Fig. 96 : Studies included in prevalence of hypoxaemia among admitted children with anaemia (SpO2<90)

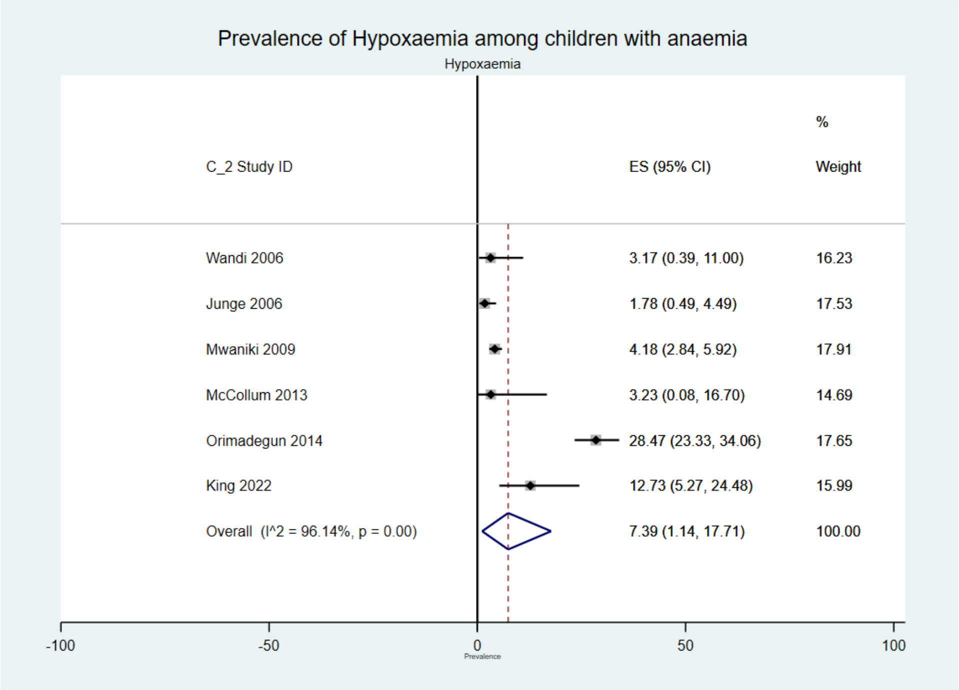

Admitted Children – asthma

Table 97: Studies included in prevalence of hypoxaemia among admitted children with asthma (SpO2<90)

Serial no	Study ID	WB Region	Proportion	LCL	UCL	Weight	Denominator
1	Jain 2018	South Asia	83.33	71.48	91.71	38.05	60

Fig. 97: Studies included in prevalence of hypoxaemia among admitted children with asthma (SpO2<90)

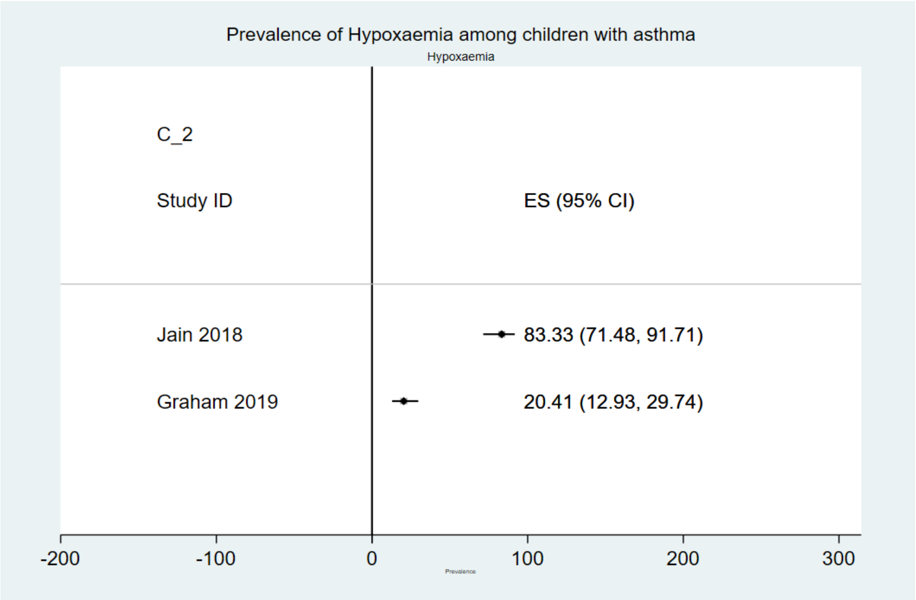

Admitted Children – HIV complications

Table 98: Studies included in prevalence of hypoxaemia among admitted children with HIV complication (SpO2<90)

Serial no	Study ID	WB Region	Proportion	LCL	UCL	Weight	Denominator
1	Kelly 2015	Sub Saharan Africa	55.00	31.53	76.94	51.25	20
2	Graham 2019	Sub Saharan Africa	10.53	1.30	33.14	48.75	19

Fig. 98: Studies included in prevalence of hypoxaemia among admitted children with HIV complication (SpO2<90)

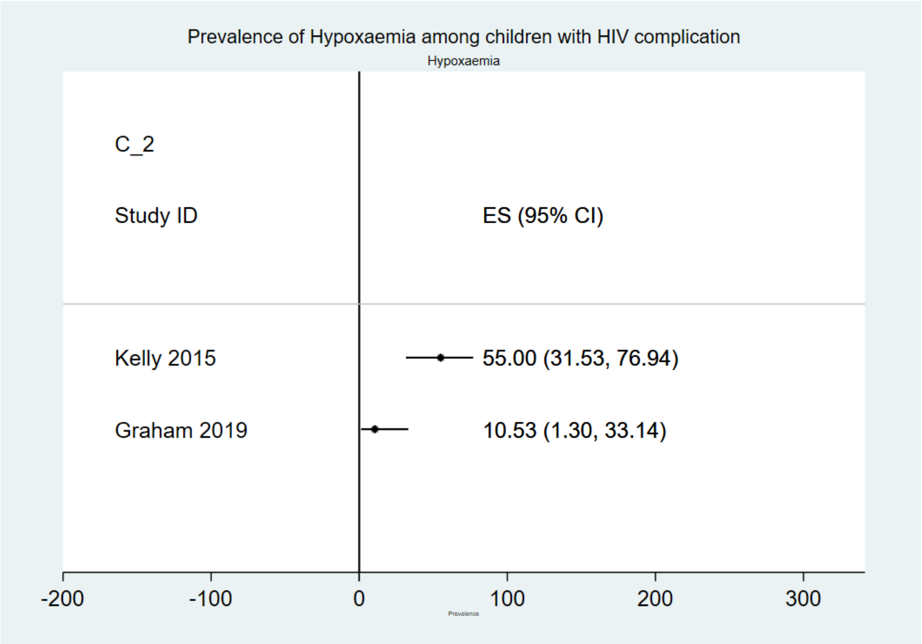

Admitted Children – malaria

Table 99: Studies included in prevalence of hypoxaemia among admitted children with malaria (SpO2<90)

Serial	Study ID	WB Region	Proportion	LCL	UCL	Weight	Denominator
1	Usen 1999	Sub Saharan Africa	3.88	1.07	9.65	6.97	103
2	Maitland 2003	Sub Saharan Africa	17.17	13.97	20.76	8.38	501
3	Wandi 2006	East Asia & Pacific	3.31	1.52	6.19	8.02	272
4	Junge 2006	Sub Saharan Africa	2.87	1.95	4.08	8.61	1044
5	Mwaniki 2009	Sub Saharan Africa	4.90	4.31	5.53	8.79	4982
6	Orimadegun 2013	Sub Saharan Africa	14.42	11.53	17.71	8.40	527
7	McCollum 2013	Sub Saharan Africa	1.87	0.81	3.66	8.30	427
8	Cserti-Gazdewich 2013	Sub Saharan Africa	2.26	1.64	3.03	8.72	1901
9	Orimadegun 2014	Sub Saharan Africa	29.81	25.19	34.76	8.22	369
10	Graham 2019	Sub Saharan Africa	8.50	7.74	9.31	8.80	5035
11	Leligdowicz 2021	Sub Saharan Africa	2.51	1.73	3.50	8.66	1317
12	King 2022	Sub Saharan Africa	9.03	6.09	12.79	8.12	310
**Overall (I2=97.76%, T2=0.04, p<0.05)**			**7.02**	**4.44**	**10.13**		**16788**

Fig. 99: Studies included in prevalence of hypoxaemia among admitted children with malaria (SpO2<90)

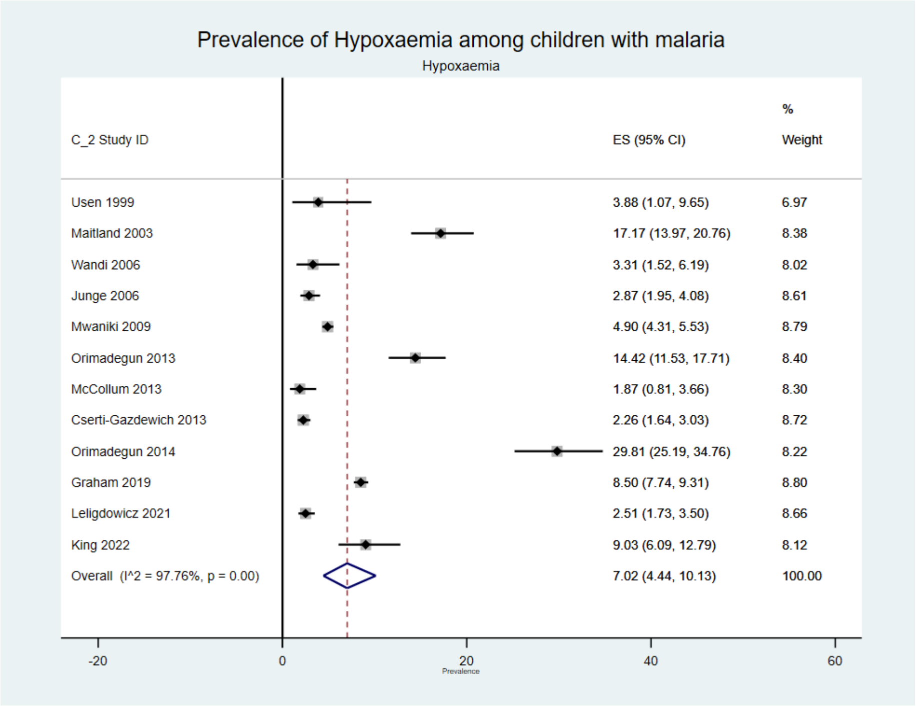

Admitted Children – meningitis

Table 100: Studies included in prevalence of hypoxaemia among admitted children with meningitis (SpO2<90)

Serial no	Study ID	WB Region	Proportion	LCL	UCL	Weight	Denominator
1	Weber 2002	Sub Saharan Africa	4.49	1.24	11.11	20.65	89
2	Wandi 2006	East Asia & Pacific	14.63	5.57	29.17	17.28	41
3	Junge 2006	Sub Saharan Africa	2.70	0.33	9.42	19.97	74
4	Orimadegun 2013	Sub Saharan Africa	8.00	2.22	19.23	18.27	50
5	Graham 2019	Sub Saharan Africa	17.40	13.94	21.32	23.83	431
**Overall (I2=84.35%, T2=0.06, p<0.05)**			**8.77**	**3.16**	**16.56**		**685**

Fig. 100: Studies included in prevalence of hypoxaemia among admitted children with meningitis (SpO2<90)

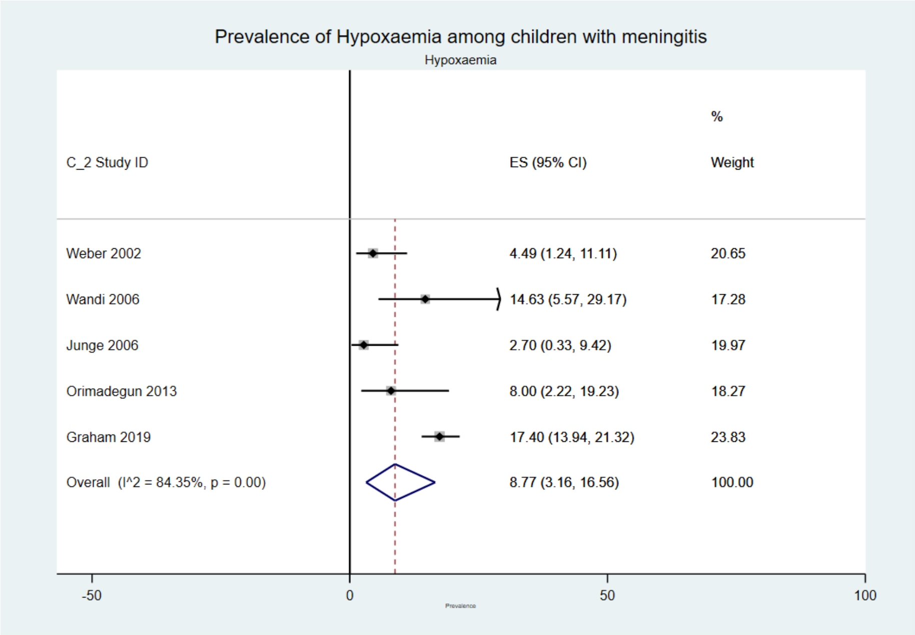

Admitted Children – seizures

Table 101: Studies included in prevalence of hypoxaemia among admitted children with seizure (SpO2<90)

Serial no	Study ID	WB Region	Proportion	LCL	UCL	Weight	Denominator
1	Weber 2002	Sub Saharan Africa	4.05	3.25	4.99	61.71	2097
2	Graham 2019	Sub Saharan Africa	18.29	16.23	20.50	38.29	1301

Fig. 101: Studies included in prevalence of hypoxaemia among admitted children with seizure (SpO2<90)

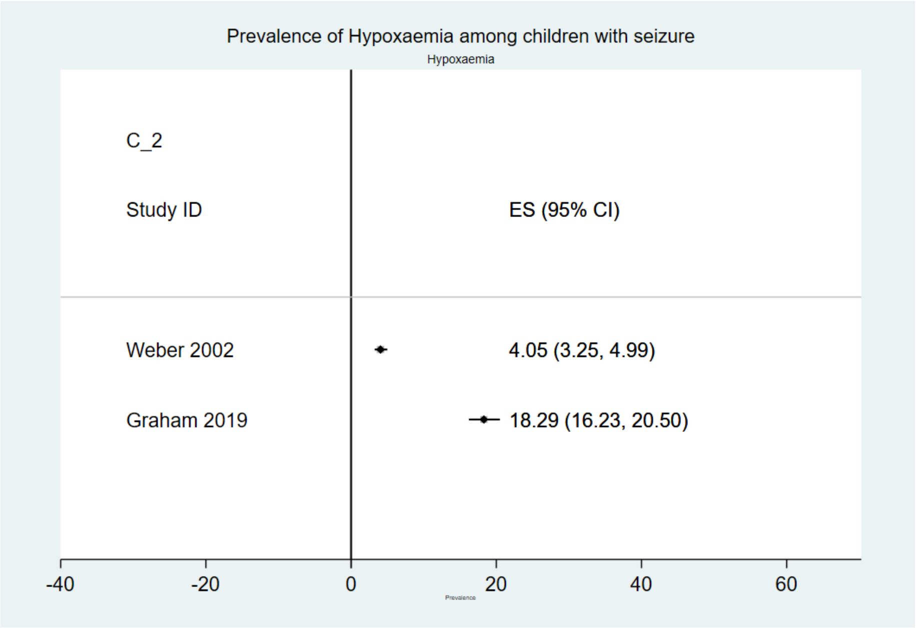

Admitted Children – sepsis

Table 102: Studies included in Prevalence of Hypoxaemia among admitted children with sepsis (SpO2<90)

Serial no	Study ID	WB Region	Proportion	LCL	UCL	Weight	Denominator
1	Orimadegun 2013	Sub Saharan Africa	22.62	16.53	29.70	18.81	168
2	McCollum 2013	Sub Saharan Africa	3.45	0.09	17.76	11.35	29
3	Shahid 2016	South Asia	23.08	8.97	43.65	10.76	26
4	Graham 2019	Sub Saharan Africa	8.70	7.85	9.61	21.72	4092
5	Shahunja 2020	South Asia	16.96	13.42	21.00	20.47	401
6	King 2022	Sub Saharan Africa	13.04	6.93	21.68	16.88	92
**Overall (I2=90.41%, T2=0.04, p<0.05)**			**13.88**	**8.22**	**20.65**		**4808**

Fig. 102: Studies included in Prevalence of Hypoxaemia among admitted children with sepsis (SpO2<90)

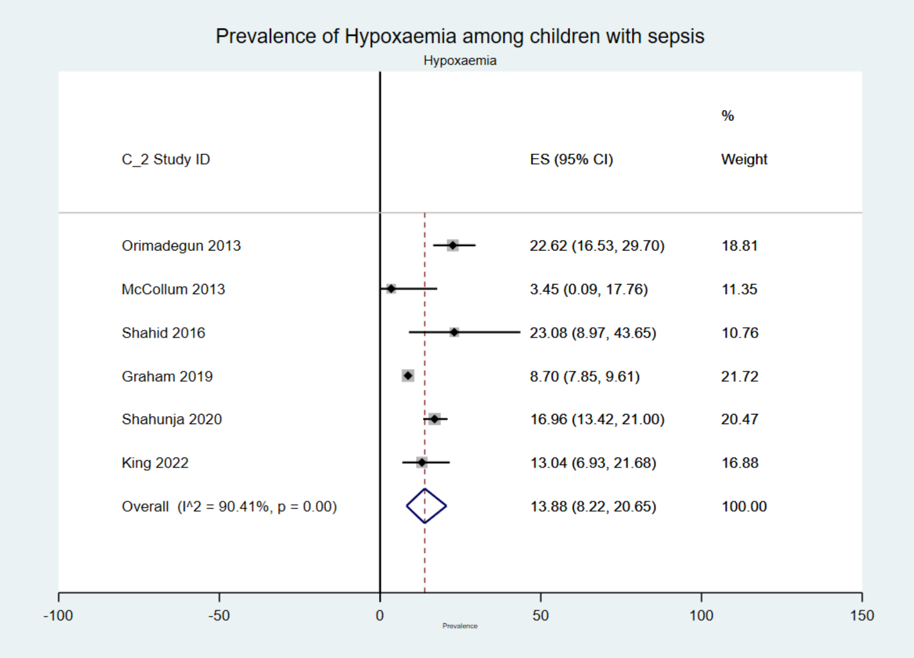

Admitted Children – trauma / injury

Table 103: Studies included in Prevalence of Hypoxaemia among admitted children with trauma (SpO2<90)

Serial no	Study ID	WB Region	Proportion	LCL	UCL	Weight	Denominator
1	Graham 2019	Sub Saharan Africa	7.09	4.39	10.74	58.49	282
2	King 2022	Sub Saharan Africa	2.00	0.55	5.04	41.51	200

Fig.103: Studies included in Prevalence of Hypoxaemia among admitted children with trauma (SpO2<90)

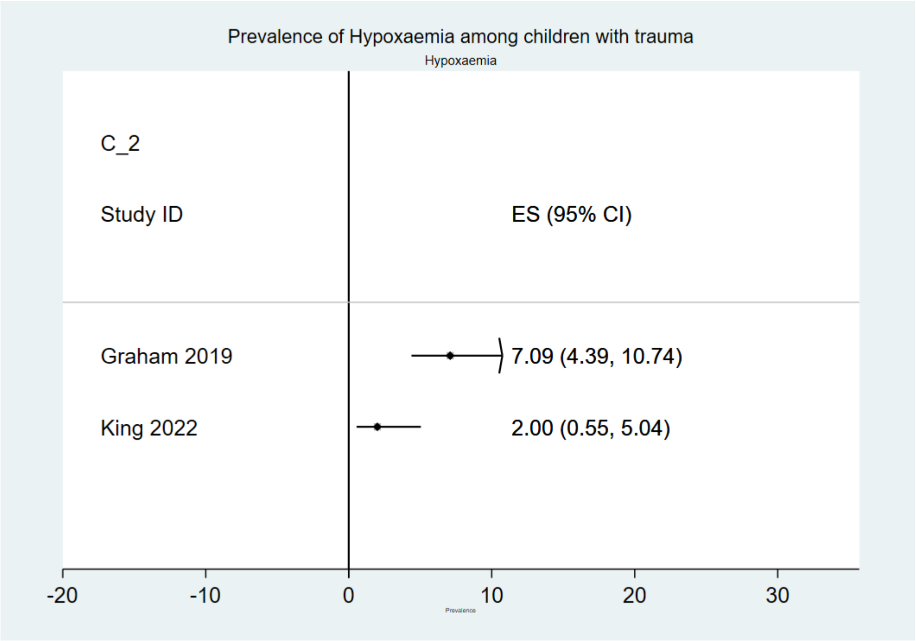

Admitted Children – tuberculosis

Table 104: Studies included in Prevalence of Hypoxaemia among admitted children with tuberculosis (SpO2<90)

Serial no	Study ID	WB Region	Proportion	LCL	UCL	Weight	Denominator
1	Wandi 2006	East Asia & Pacific	20	5.7334	43.6614	100	20

Fig. 104: Studies included in Prevalence of Hypoxaemia among admitted children with tuberculosis (SpO2<90)

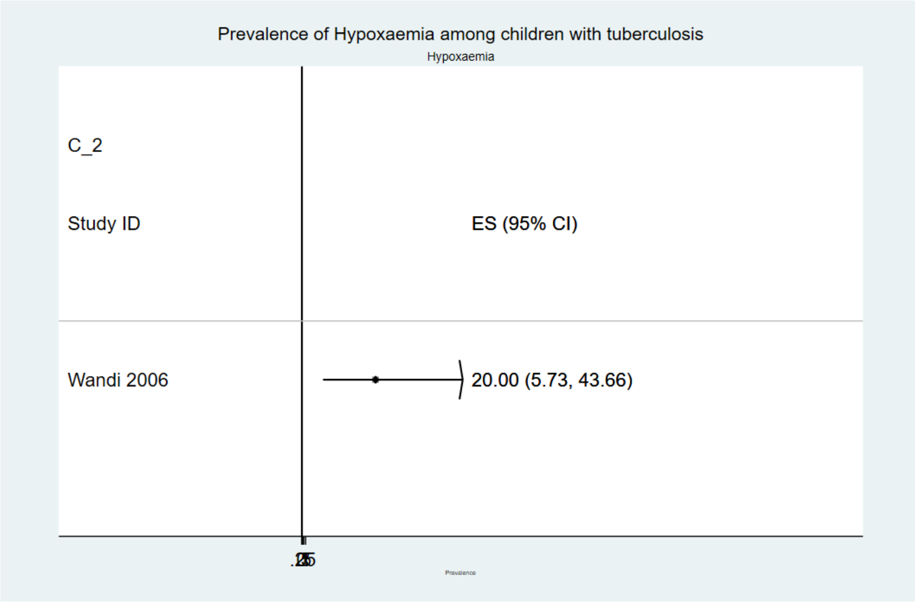

Admitted Children – COVID-19

Table 105: Studies included in prevalence of hypoxaemia among admitted children with COVID-19 (SpO2<90)

Serial no	Study ID	WB Region	Proportion	LCL	UCL	Weight	Denominator
1	Snouber 2022	Middle East & North Africa	49.11	39.54	58.73	100.00	112

Fig. 105: Studies included in prevalence of hypoxaemia among admitted children with COVID-19 (SpO2<90)

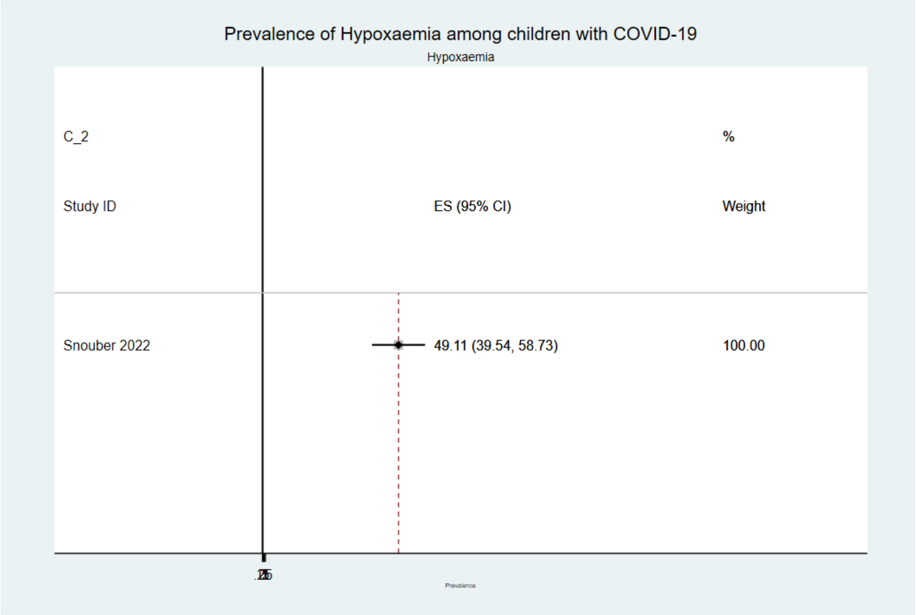

Admitted Children – diarrhoea

Table 106: Studies included in prevalence of hypoxaemia among admitted children with diarrheoa (SpO2<90)

Serial no	Study ID	WB Region	Proportion	LCL	UCL	Weight	Denominator
1	Wandi 2006	East Asia & Pacific	4.72	1.75	10.00	14.47	127
2	Junge 2006	Sub Saharan Africa	0.00	0.00	3.18	14.10	114
3	Chisti 2011	South Asia	18.33	9.52	30.44	11.57	60
4	Orimadegun 2013	Sub Saharan Africa	6.38	2.38	13.38	13.41	94
5	McCollum 2013	Sub Saharan Africa	0.00	0.00	11.94	8.26	29
6	Shahid 2016	South Asia	6.73	2.75	13.38	13.78	104
7	Graham 2019	Sub Saharan Africa	6.10	4.91	7.48	18.21	1410
8	King 2022	Sub Saharan Africa	5.56	0.14	27.29	6.20	18
**Overall (I2=77.87%, T2=0.03, p<0.05)**			**4.64**	**1.92**	**8.25**		**1956**

Fig. 106: Studies included in prevalence of hypoxaemia among admitted children with diarrheoa (SpO2<90)

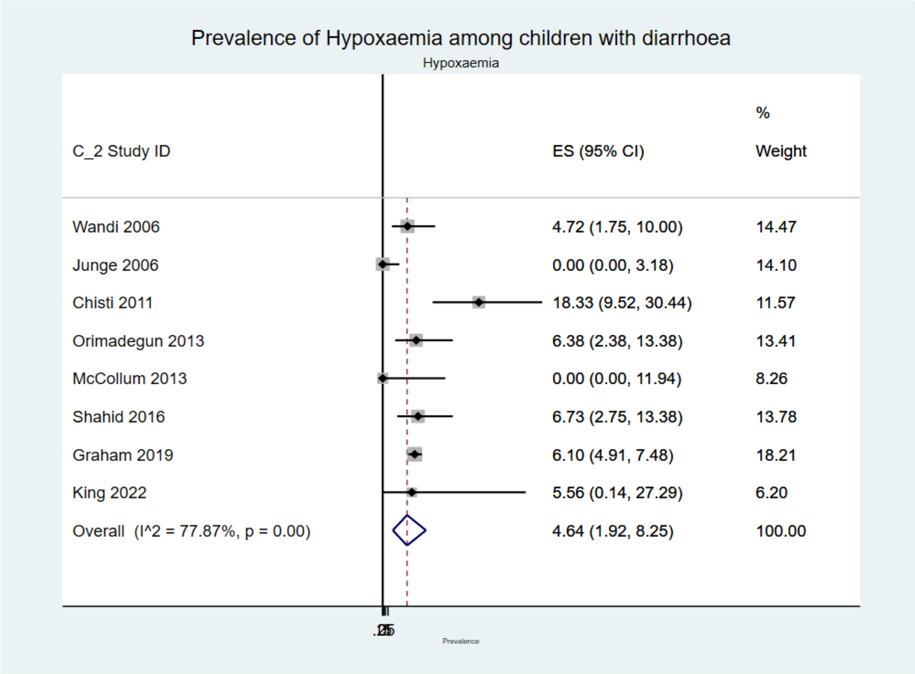

Admitted Children – malnutrition

Table 107: Studies included in prevalence of hypoxaemia among admitted children with malnutrition (SpO2<90)

Serial no	Study ID	WB Region	Proportion	LCL	UCL	Weight	Denominator
1	Wandi 2006	East Asia & Pacific	8.33	0.21	38.48	4.75	12
2	Junge 2006	Sub Saharan Africa	1.85	0.60	4.25	13.27	271
3	McCollum 2013	Sub Saharan Africa	5.26	0.13	26.03	6.26	19
4	Chisti 2013	South Asia	11.08	7.92	14.95	13.49	334
5	Graham 2019	Sub Saharan Africa	3.50	2.56	4.65	14.24	1286
6	Ashraf 2019	South Asia	10.64	8.00	13.78	13.77	470
7	Kintwa 2021	East Asia & Pacific	20.71	14.33	28.38	12.28	140
8	King 2022	Sub Saharan Africa	5.56	0.68	18.66	8.52	36
9	Faruk 2022	South Asia	9.80	6.71	13.70	13.40	306
**Overall (I2=91.16%, T2=0.04, p<0.05)**			**7.62**	**3.94**	**12.24**		**2874**

Fig. 107: Studies included in prevalence of hypoxaemia among admitted children with malnutrition (SpO2<90)

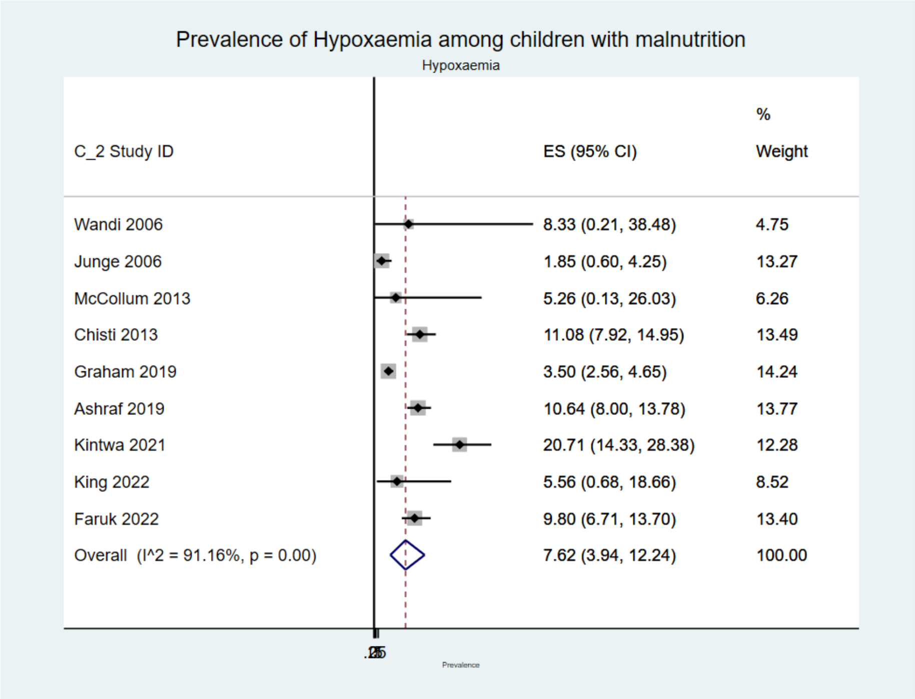

Admitted Adults – all

Table 108: Studies included in prevalence of hypoxaemia among all admitted adults (SpO2<90)

Serial	C_2StudyID	N_8WB_Region	ES_100	LCL_100	UCL_100	_WT	Adult_denom
1.	Foran 2010	Sub Saharan Africa	9.17	4.49	16.23	13.25	109
2.	Evans 2012	Sub Saharan Africa	9.72	5.42	15.77	14.34	144
3.	Riviello 2016	Sub Saharan Africa	12.05	10.13	14.17	18.45	1046
4.	Wasingya-Kasereka 2020	Sub Saharan Africa	6.31	5.41	7.31	18.97	2599
5.	Aslam 2021	South Asia	15.17	11.25	19.83	16.48	290
6.	Kayambankadzanja 2021	Sub Saharan Africa	3.96	2.91	5.27	18.51	1135
**Overall (I2=93.45%, T2=0.02, p<0.05)**			8.87	5.69	12.66		5323

Fig. 108: Studies included in prevalence of hypoxaemia among all admitted adults (SpO2<90)

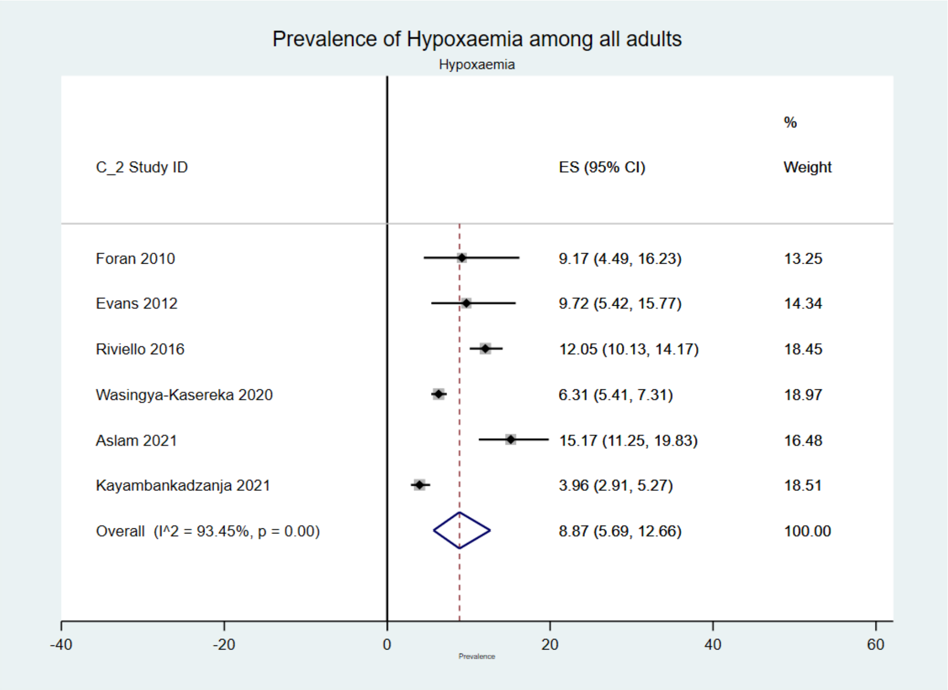

Admitted Adults - fever

Table 109: Studies included in prevalence of hypoxaemia among admitted adults with fever (SpO2<90)

Serial no	Study ID	WB Region	Proportion	LCL	UCL	Weight	Denominator
1	Carugati 2018	Sub Saharan Africa	9.79	7.11	13.04	77.83	419
2	YeLynn 2019	East Asia Pacific	21.01	14.08	29.43	22.17	120

Fig. 109: Studies included in prevalence of hypoxaemia among admitted adults with fever (SpO2<90)

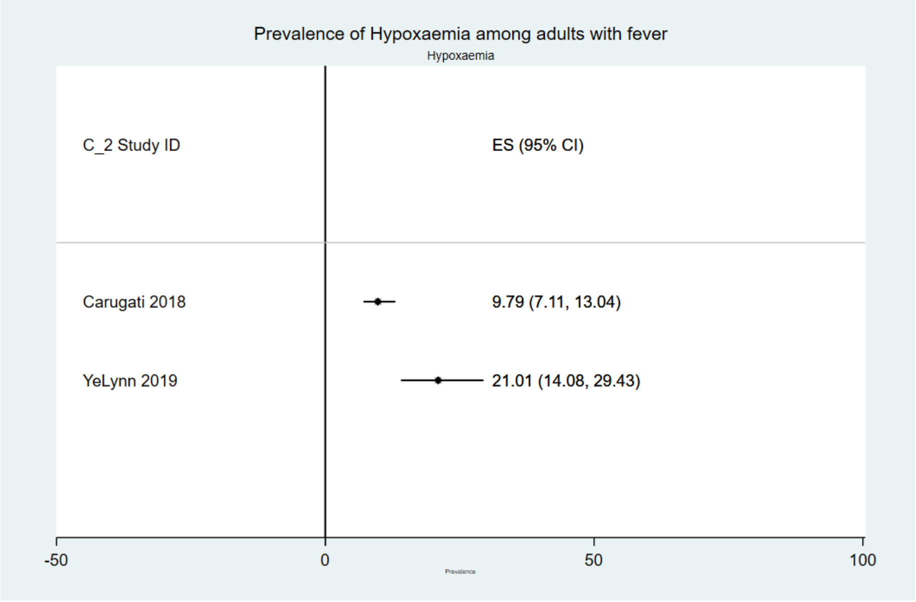

Admitted Adults – pneumonia

Table 110: Studies included in prevalence of hypoxaemia among admitted adults with any pneumonia (not defined) (SpO2<90)

Serial	Study ID	WB Region	Proportion	LCL	UCL	Weight	Denominator
1	Tokman 2014	Sub Saharan Africa	15.77	11.41	20.99	24.94	241
2	Worodria 2018	Sub Saharan Africa	10.60	9.25	12.08	25.19	1887
3	Aston 2019	Sub Saharan Africa	16.22	12.94	19.96	25.07	450
4	Arana-Calderon 2022	Latin America & Caribbean	90.51	84.83	94.59	24.80	158
**Overall (I2=99.40%, T2=0.37, p<0.05)**			**31.91**	**8.67**	**61.45**		**2736**

Fig. 110: Studies included in prevalence of hypoxaemia among admitted adults with any pneumonia (not defined) (SpO2<90)

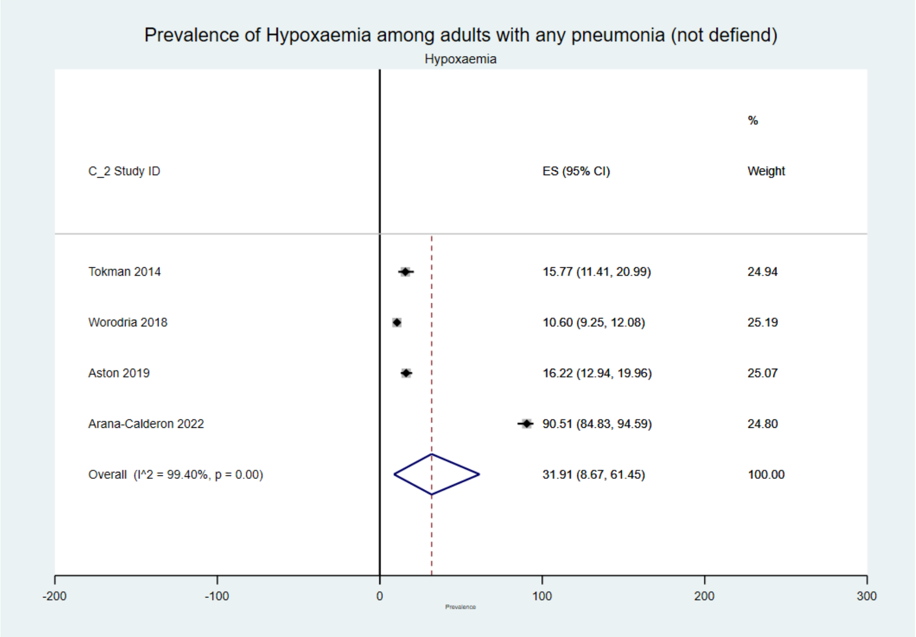

Admitted Adults – asthma

Table 111: Studies included in prevalence of hypoxaemia among admitted adults with asthma (SpO2<90)

Serial	Study ID	WB Region	Proportion	LCL	UCL	Weight	Denominator
1	Hussain 2005	South Asia	16.84	9.94	25.90	100.00	95

Fig. 111: Studies included in prevalence of hypoxaemia among admitted adults with asthma (SpO2<90)

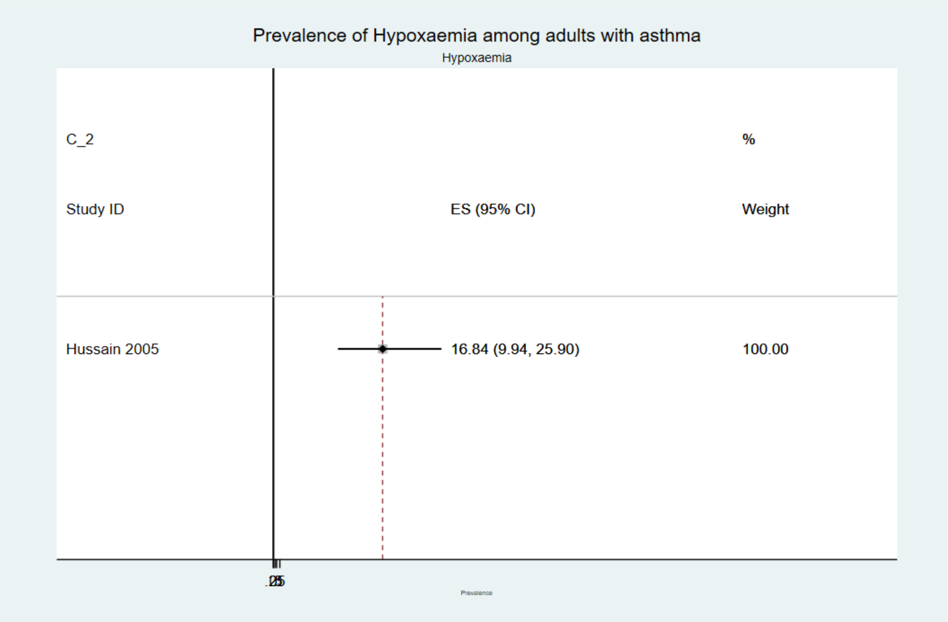

Admitted Adults – HIV complications

Table 112: Studies included in prevalence of hypoxaemia among admitted adults with HIV complications (SpO2<90)

Serial	Study ID	WB Region	Proportion	LCL	UCL	Weight	Denominator
1	Koss 2015	Sub Saharan Africa	16.41	13.96	19.10	37.45	835
2	Worodria 2018	Sub Saharan Africa	11.41	9.66	13.35	39.11	1192
3	Carugati 2018	Sub Saharan Africa	15.00	9.85	21.49	23.44	160
**Overall (I2=81.36%, T2=0.01, p<0.05)**			**14.02**	**10.41**	**18.08**		**2187**

Fig. 112: Studies included in prevalence of hypoxaemia among admitted adults with HIV complications (SpO2<90)

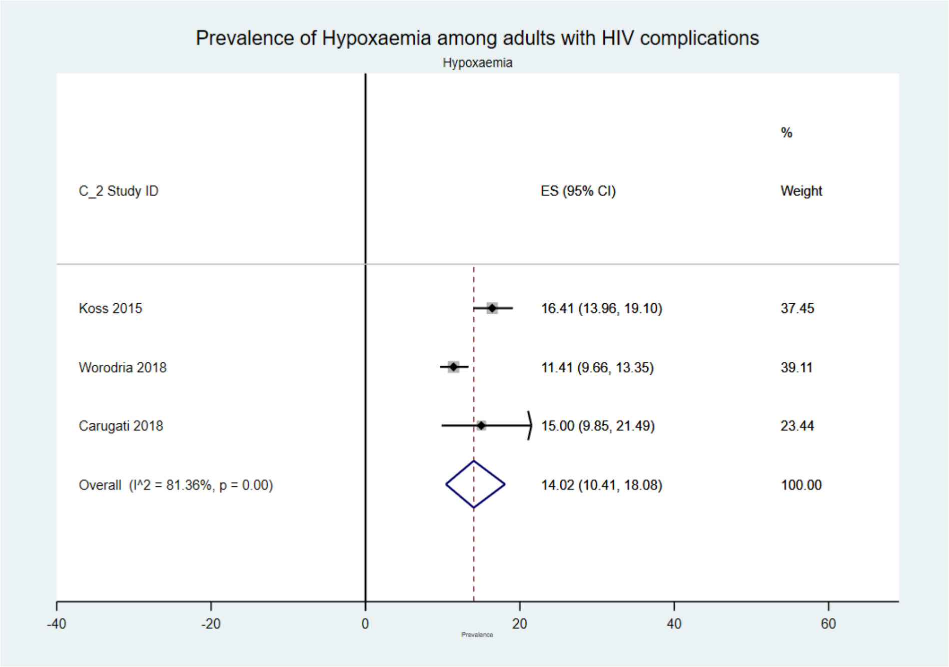

Admitted Adults – seizures

Table 113: Studies included in prevalence of hypoxaemia among admitted adults with seizure (SpO2<90)

Serial	Study ID	WB Region	Proportion	LCL	UCL	Weight	Denominator
1	Amare 2008	Sub Saharan Africa	25.21	17.70	33.99	100.00	119

Fig. 113: Studies included in prevalence of hypoxaemia among admitted adults with seizure (SpO2<90)

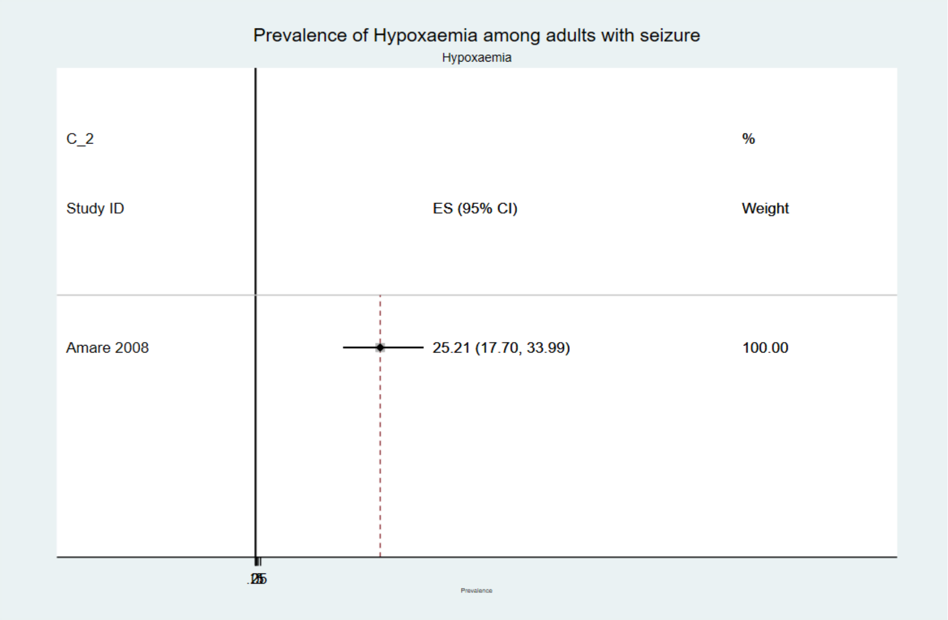

Admitted Adults – sepsis

Table 114: Studies included in prevalence of hypoxaemia among admitted adults with sepsis (SpO2<90)

Serial	Study ID	WB Region	Proportion	LCL	UCL	Weight	Denominator
1	Papali 2017	Latin America & Caribbean	24.10	17.81	31.33	31.51	166
2	Boonmee 2020	East Asia & Pacific	27.17	25.01	29.41	34.67	1616
3	MarMinn 2021	East Asia & Pacific	12.18	9.47	15.34	33.82	509
**Overall (I2=96.46%, T2=0.05, p<0.05)**			**20.70**	**10.99**	**32.49**		**2291**

Fig. 114: Studies included in prevalence of hypoxaemia among admitted adults with sepsis (SpO2<90)

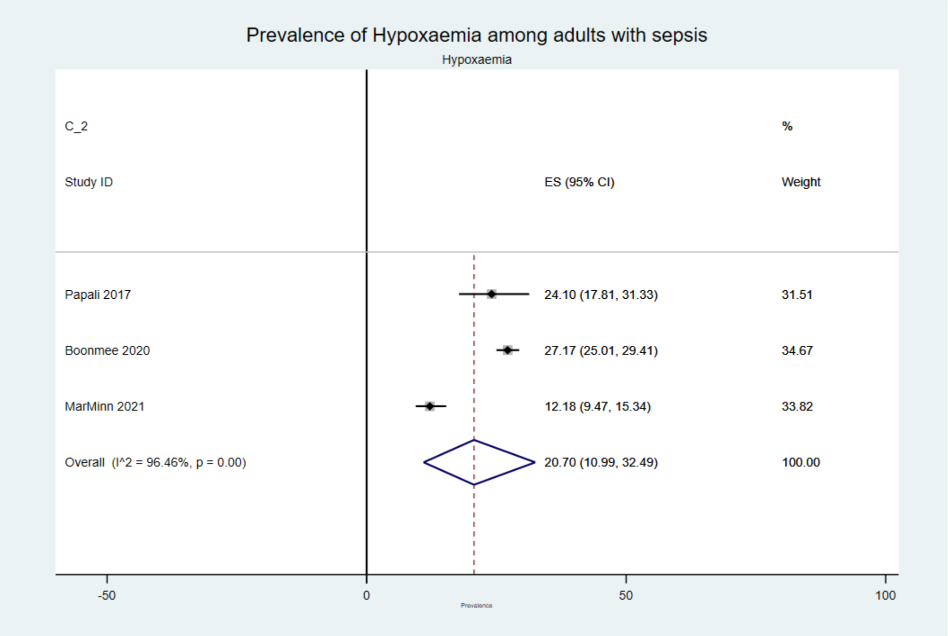

Admitted Adults – trauma / injury

Table 115: Studies included in prevalence of hypoxaemia among admitted adults with trauma (SpO2<90)

Serial	Study ID	WB Region	Proportion	LCL	UCL	Weight	Denominator
1	Stassen 2014	Sub Saharan Africa	37.88	26.22	50.66	100.00	66

Fig. 115: Studies included in prevalence of hypoxaemia among admitted adults with trauma (SpO2<90)

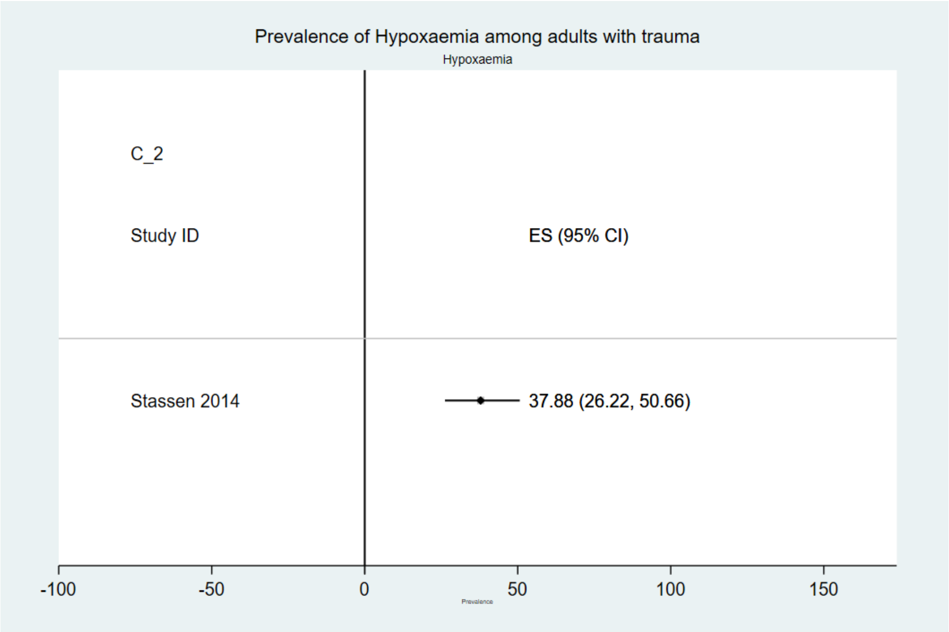

Admitted Adults – COVID-19

Table 116: Studies included in prevalence of hypoxaemia among admitted adults with COVID-19 (SpO2<90)

Serial	Study ID	WB Region	Proportion	LCL	UCL	Weight	Denominator
1	Homayounieh 2020	Middle East & North Africa	29.33	19.38	40.98	10.94	75
2	Mejia 2020	Latin America & Caribbean	64.50	59.38	69.38	11.12	369
3	VafadarMoradi 2021	Middle East & North Africa	64.84	58.12	71.15	11.09	219
4	Xiong 2021	East Asia & Pacific	88.90	86.82	90.76	11.15	1027
5	Diaz-Velez 2021	Latin America & Caribbean	56.39	51.88	60.82	11.13	493
6	Anyaypoma-Ocon 2021	Latin America & Caribbean	72.89	66.58	78.58	11.09	225
7	Marcolino 2021	Latin America & Caribbean	13.39	11.91	14.98	11.16	1964
8	Padmaprakash 2021	South Asia	5.14	4.09	6.37	11.16	1536
9	Soto 2022	Latin America & Caribbean	56.84	54.22	59.44	11.16	1418
**Overall (I2=99.78%, T2=0.61, p<0.05)**			**49.27**	**24.72**	**74.02**		**7326**

Fig. 116: Studies included in prevalence of hypoxaemia among admitted adults with COVID-19 (SpO2<90)

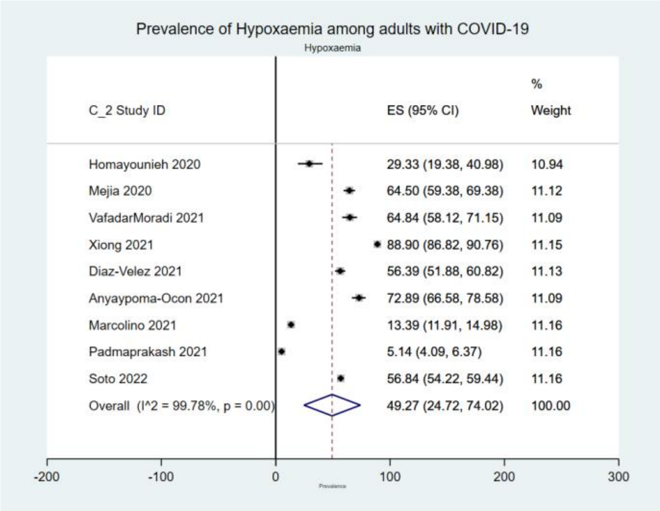

OPD/ED Neonates – all

Table 117: Studies included in prevalence of hypoxaemia among all presenting neonates (SpO2<90)

Serial	Study ID	WB Region	Proportion	LCL	UCL	Weight	Denominator
1	Weber 2003	Mixed	7.84	6.95	8.81	28.16	3303
2	Agrawal 2011	South Asia	32.93	22.94	44.19	25.55	82
3	Emdin 2015	South Asia	0.38	0.05	1.36	27.78	528
4	Graham 2021	Sub Saharan Africa	0.00	0.00	20.59	18.50	16
**Overall (I2=97.65%, T2=0.12, p<0.05)**			**6.76**	**0.31**	**18.74**		**3929**

Fig. 117: Studies included in prevalence of hypoxaemia among all presenting neonates (SpO2<90)

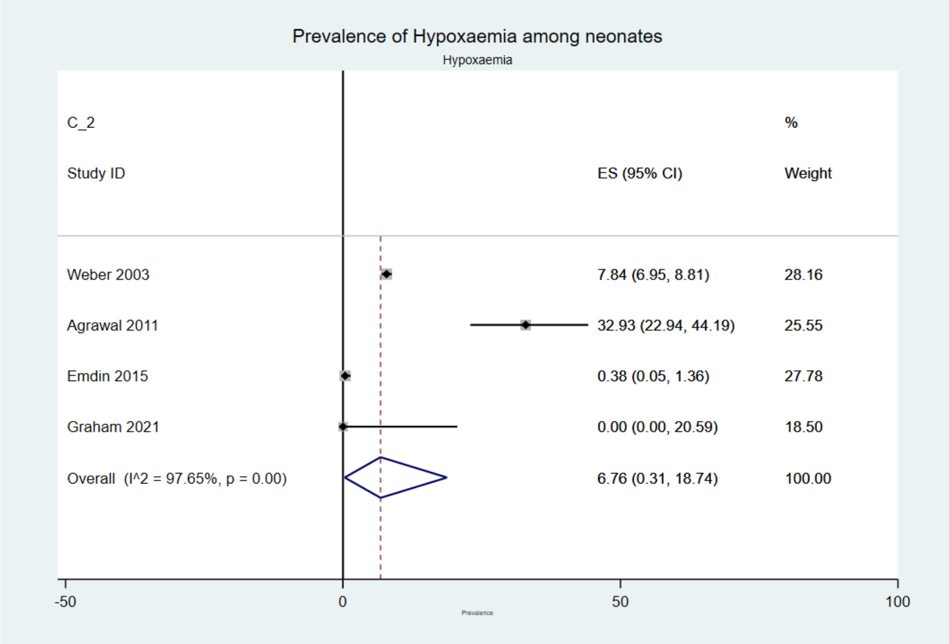

OPD/ED Neonates – pneumonia

Table 118: Studies included in prevalence of hypoxaemia among presenting neonates with pneumonia (SpO2<90)

No studies

Fig. 118: Studies included in prevalence of hypoxaemia among presenting neonates with pneumonia (SpO2<90)

No studies

OPD/ED Children – all

Table 119: Studies included in prevalence of hypoxaemia among all presenting children (SpO2<90)

Serial	Study ID	WB Region	Proportion	LCL	UCL	Weight	Denominator
1	Agrawal 2011	South Asia	43.59	32.39	55.30	20.83	78
2	Garde 2015	South Asia	5.63	4.88	6.46	26.47	3374
3	Blanc 2019	East Asia & Pacific	1.38	0.88	2.07	26.29	1663
4	Graham 2021	Sub Saharan Africa	0.97	0.62	1.44	26.41	2480
**Overall (I2=98.68%, T2=0.05, p<0.05)**			**7.04**	**2.48**	**13.60**		**7595**

Fig. 119: Studies included in prevalence of hypoxaemia among all presenting children (SpO2<90)

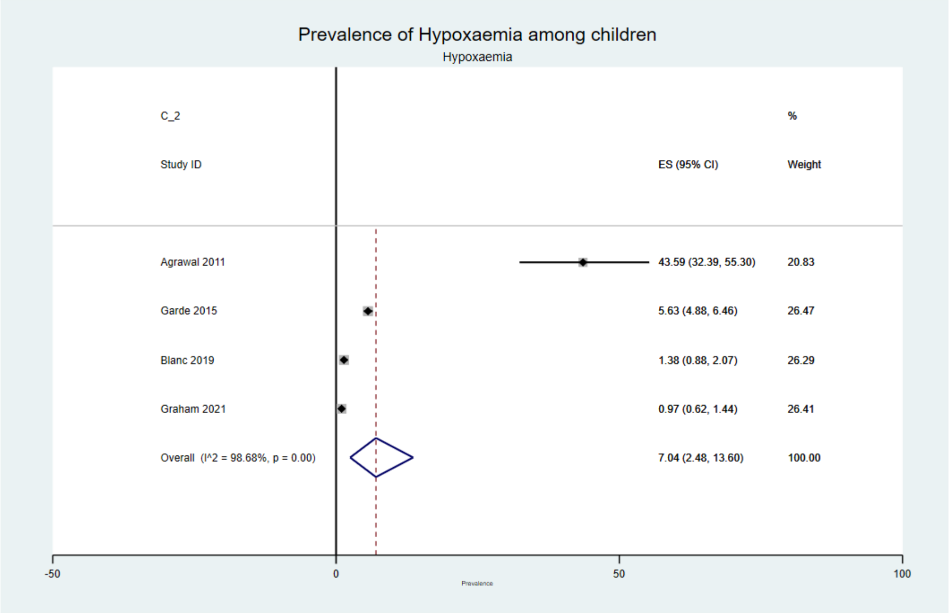

OPD/ED Children - fever

Table 120: Studies included in prevalence of hypoxaemia among presenting children with fever (SpO2<90)

Serial	Study ID	WB Region	Proportion	LCL	UCL	Weight	Denominator
1	Salah 2014	Sub Saharan Africa	0.667	0.017	3.658	7.578	150
2	Graham 2021	Sub Saharan Africa	0.981	0.582	1.546	92.422	1835

Fig. 120: Studies included in prevalence of hypoxaemia among presenting children with fever (SpO2<90)

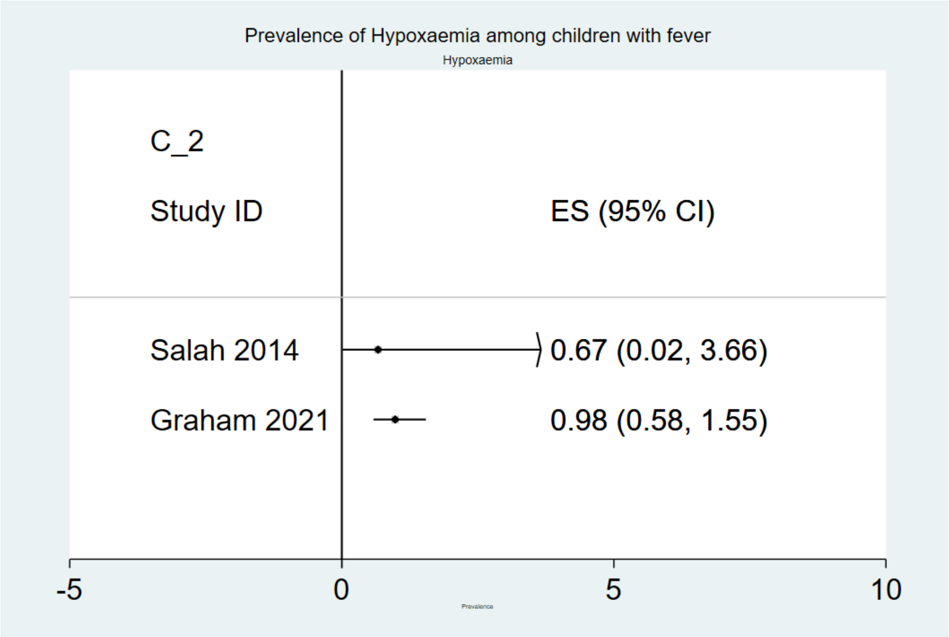

OPD/ED Children – pneumonia all WHO-classified

Table 121: Studies included in prevalence of hypoxaemia among presenting children with pneumonia (WHO-classification) (SpO2<90)

Sl no	Study ID	WB Region	Proportion	LCL	UCL	Weight	Denominator
1.	Lodha 2004	South Asia	25.69	17.80	34.94	9.69	109
2.	Basnet 2006	South Asia	38.67	30.84	46.95	9.85	150
3.	Al Janabi 2009	Middle East & North Africa	6.73	2.75	13.38	9.66	104
4.	Mathisen 2010	South Asia	1.89	1.37	2.55	10.28	2219
5.	Rao 2012	South Asia	36.99	29.15	45.36	9.84	146
6.	Thomas 2015	East Asia & Pacific	7.42	6.50	8.43	10.29	2951
7.	McCollum 2016	Sub Saharan Africa	4.91	4.55	5.30	10.31	13266
8.	Tesfaye 2020	Sub Saharan Africa	14.55	12.34	16.98	10.24	928
9.	Pukai 2020	East Asia & Pacific	91.46	86.67	94.94	9.96	199
10.	Shrestha 2020	South Asia	58.13	50.08	65.87	9.88	160
Overall (I2=99.42%, T2=0.14, p<0.05)			25.14	15.59	36.08		20232

Fig. 121: Studies included in prevalence of hypoxaemia among presenting children with pneumonia (WHO-classification) (SpO2<90)

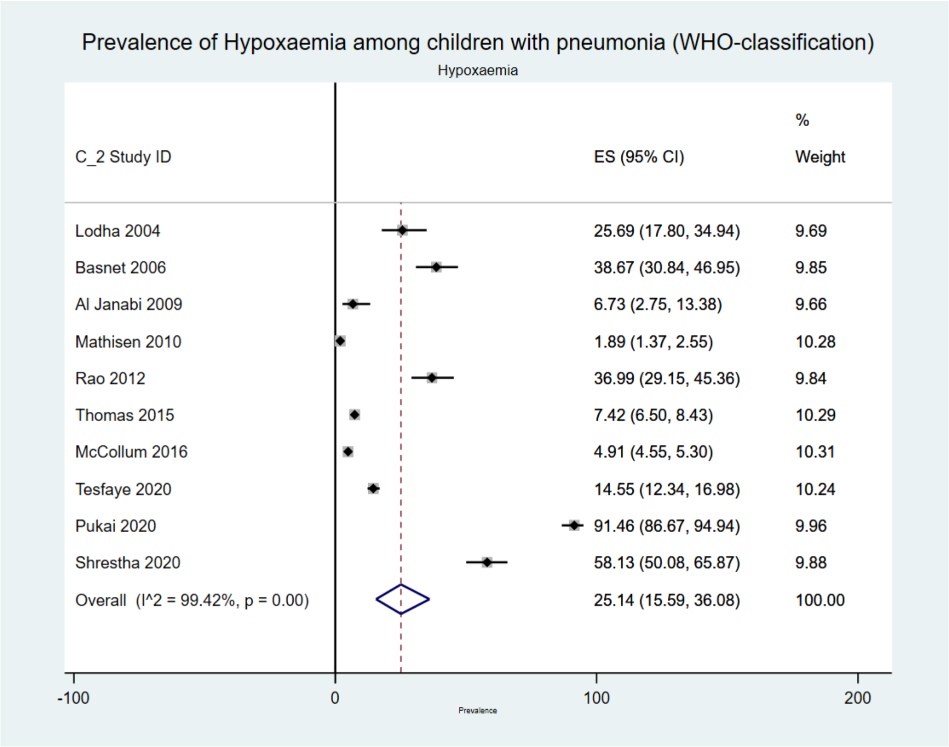

OPD/ED Children – pneumonia severe (WHO classification)

Table 122: Studies included in prevalence of hypoxaemia among presenting children with pneumonia (WHO-severe) (SpO2<90)

Sl no	Study ID	WB Region	Proportion	LCL	UCL	Weight	Denominator
1.	Basnet 2006	South Asia	88.89	75.95	96.29	32.51	45
2.	Mathisen 2010	South Asia	26.72	19.37	35.15	33.84	131
3.	Rao 2012	South Asia	48.08	38.17	58.09	33.65	104
Overall (I2=96.77%, T2=0.34, p<0.05)			55.36	22.66	85.69		280

Fig. 122: Studies included in prevalence of hypoxaemia among presenting children with pneumonia (WHO-severe) (SpO2<90)

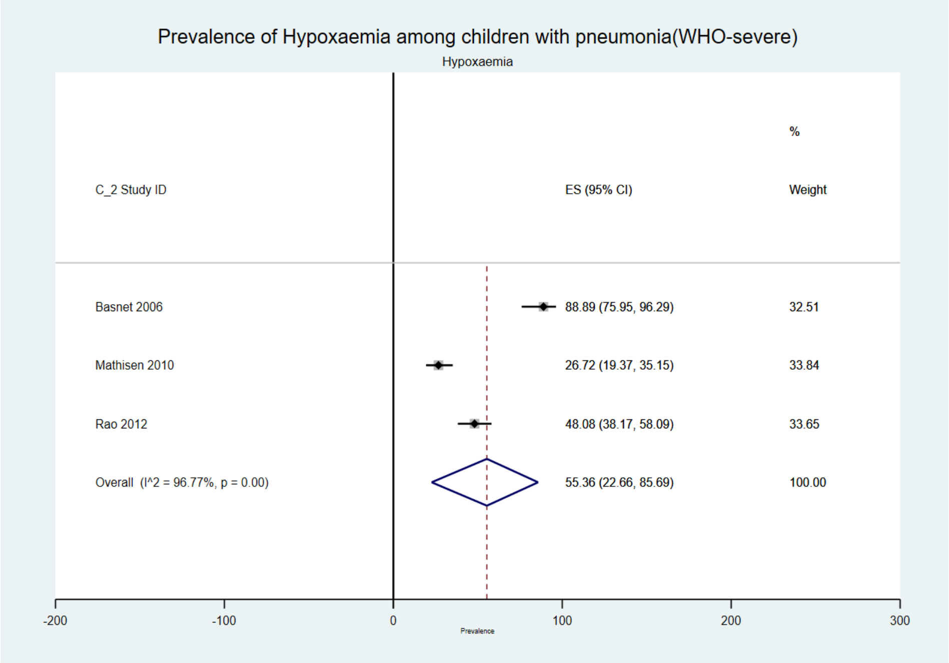

OPD/ED Children – pneumonia non-severe (WHO classification)

Table 123: Studies included in prevalence of hypoxaemia among presenting children with pneumonia (WHO-non severe) (SpO2<90)

Serial	Study ID	WB Region	Proportion	LCL	UCL	Weight	Denominator
1	Basnet 2006	South Asia	17.14	10.49	25.73	33.52	105
2	Mathisen 2010	South Asia	0.34	0.13	0.69	34.84	2088
3	Rao 2012	South Asia	9.52	2.66	22.62	31.64	42
**Overall (I2=96.96%, T2=0.23, p<0.05)**			**6.61**	**0.00**	**26.68**		**2235**

Fig. 123: Studies included in prevalence of hypoxaemia among presenting children with pneumonia (WHO-non severe) (SpO2<90)

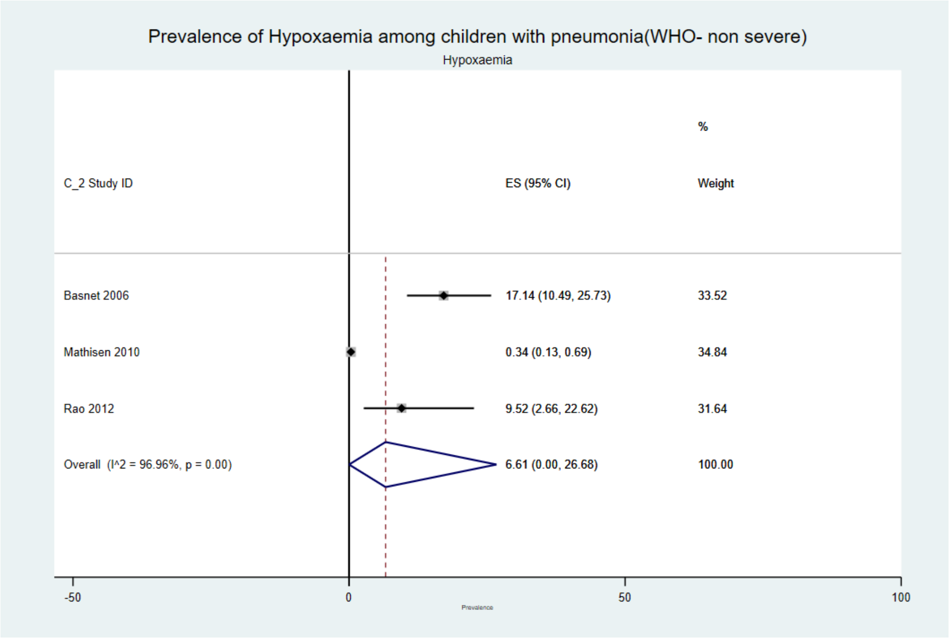

OPD/ED Children – pneumonia radiological

Table 124: Studies included in prevalence of hypoxaemia among presenting children with pneumonia (radiological) (SpO2<90)

Serial	Study ID	WB Region	Proportion	LCL	UCL	Weight	Denominator
1	Shrestha 2020	South Asia	67.01	56.73	76.22	100.00	97

Fig. 124: Studies included in prevalence of hypoxaemia among presenting children with pneumonia (radiological) (SpO2<90)

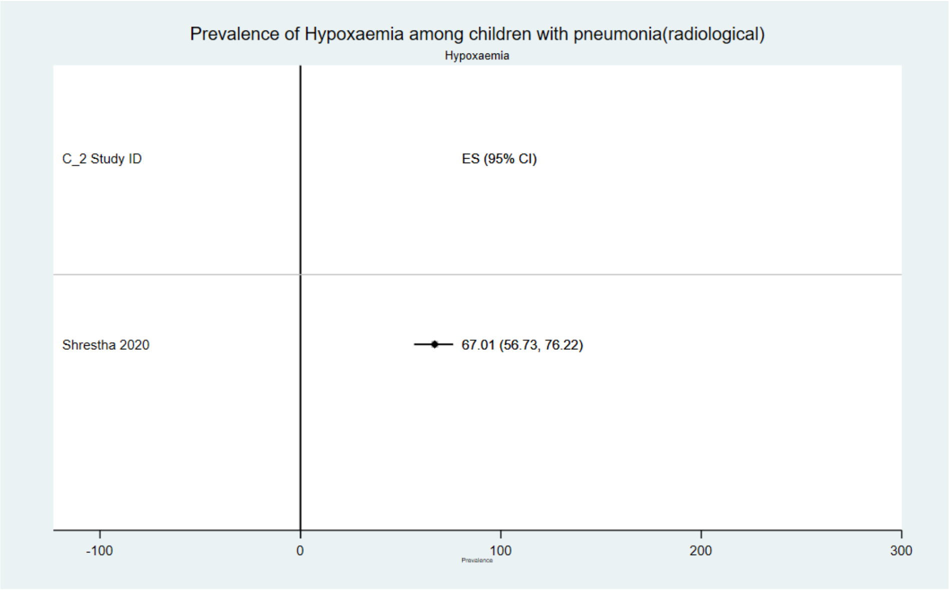

OPD/ED Children – bronchiolitis

Table 125: Studies included in prevalence of hypoxaemia among presenting children with bronchiolitis (SpO2<90)

Serial	Study ID	WB Region	Proportion	LCL	UCL	Weight	Denominator
1.	Rao 2012	South Asia	8.89	3.92	16.77	100	90

Fig. 125: Studies included in prevalence of hypoxaemia among presenting children with bronchiolitis (SpO2<90)

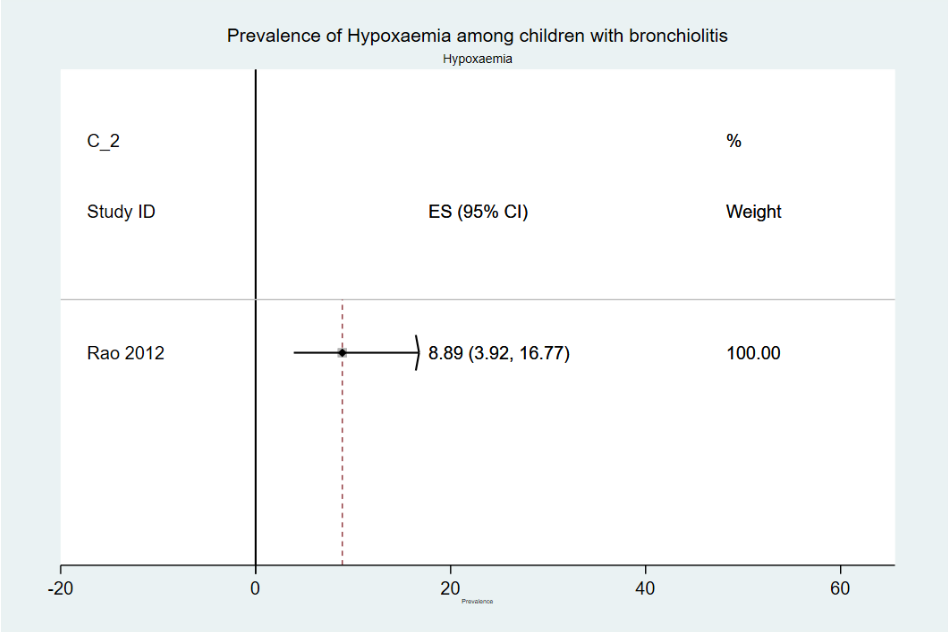

OPD/ED Children – pneumonia unspecified

Table 126: Studies included in prevalence of hypoxaemia among presenting children with pneumonia (unspecified) (SpO2<90)

Seria l	Study ID	WB Region	Proportio n	LCL	UCL	Weigh t	Denominat or
1.	Rao 2012	South Asia	23.75	18.7 2	29.3 9	21.33	261
2.	McCracken 2013	Latin America & Caribbean	29.22	26.7 1	31.8 3	22.14	1249
3.	vonderWeid 2018	Sub Saharan Africa	15.91	6.64	30.0 7	17.38	44
4.	Sylvies 2020	Sub Saharan Africa	46.03	33.3 9	59.0 6	18.62	63
5.	Graham 2021	Sub Saharan Africa	5.63	2.46	10.8 0	20.53	142
**Overall (I2=94.15%, T2=0.08, p<0.05)**			**22.54**	**12.5 8**	**34.3 3**		**1759**

Fig. 126: Studies included in prevalence of hypoxaemia among presenting children with pneumonia (unspecified) (SpO2<90)

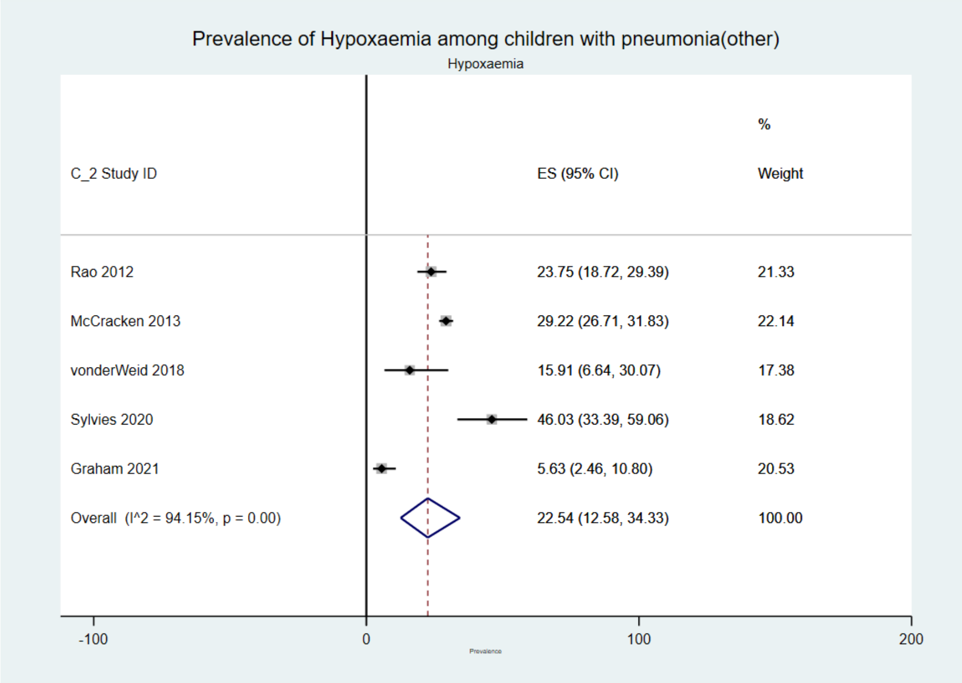

OPD/ED Children – anaemia

Table 127: Studies included in prevalence of hypoxaemia among presenting children with anaemia (SpO2<90)

Serial	Study ID	WB Region	Proportion	LCL	UCL	Weight	Denominator
1.	Chinawa 2013	Sub Saharan Africa	18.18	10.76	27.84	100.00	88

Fig. 127: Studies included in prevalence of hypoxaemia among presenting children with anaemia (SpO2<90)

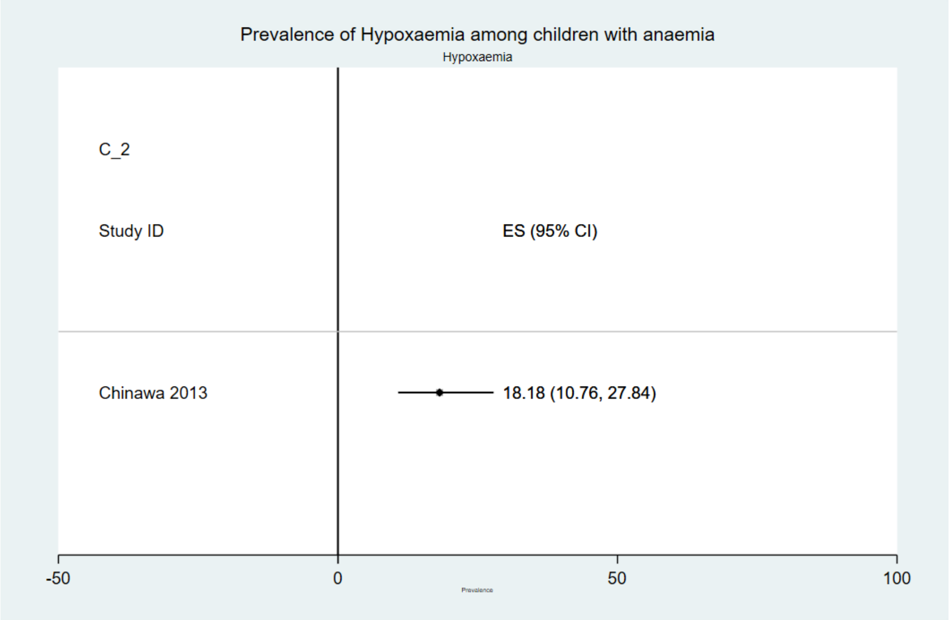

OPD/ED Children – asthma

Table 128: Studies included in prevalence of hypoxaemia among presenting children with asthma (SpO2<90)

Serial	Study ID	WB Region	Proportion	LCL	UCL	Weight	Denominator
1.	vonderWeid 2018	Sub Saharan Africa	9.76	4.31	18.32	100.00	82

Fig. 128: Studies included in prevalence of hypoxaemia among presenting children with asthma (SpO2<90)

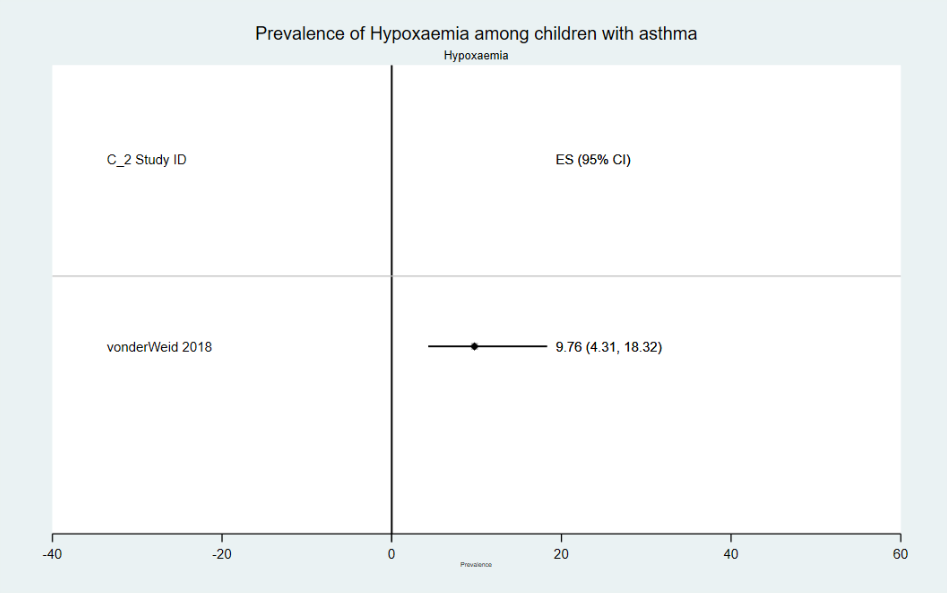

OPD/ED Children – malaria

Table 129: Studies included in prevalence of hypoxaemia among presenting children with malaria (SpO2<90)

Serial	Study ID	WB Region	Proportion	LCL	UCL	Weight	Denominator
1.	Graham 2021	Sub Saharan Africa	0.81	0.40	1.44	100.00	1363

Fig. 129: Studies included in prevalence of hypoxaemia among presenting children with malaria (SpO2<90)

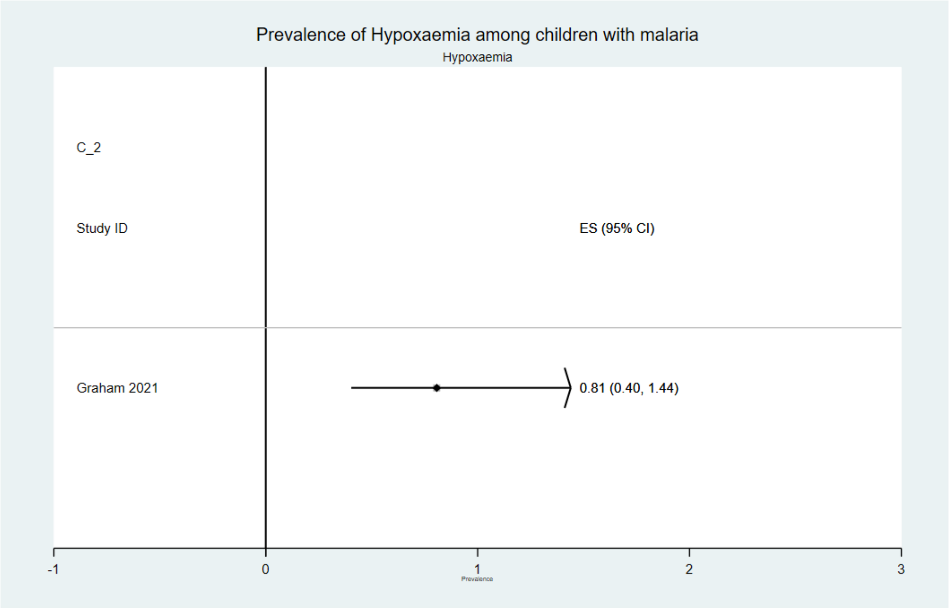

OPD/ED Children – sepsis

Table 130: Studies included in prevalence of hypoxaemia among presenting children with sepsis (SpO2<90)

Serial	Study ID	WB Region	Proportion	LCL	UCL	Weight	Denominator
1.	Graham 2021	Sub Saharan Africa	0.00	0.00	6.49	100.00	55

Fig. 130: Studies included in prevalence of hypoxaemia among presenting children with sepsis (SpO2<90)

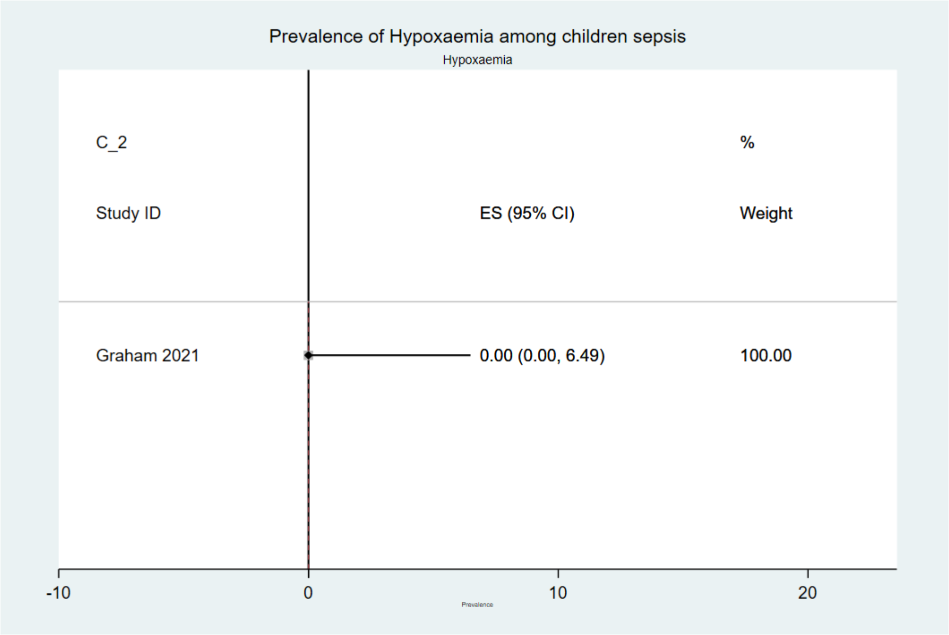

OPD/ED Children – diarrhoea

Table 131: Studies included in prevalence of hypoxaemia among presenting children with diarrhea (SpO2<90)

Serial	Study ID	WB Region	Proportion	LCL	UCL	Weight	Denominator
1.	Graham 2021	Sub Saharan Africa	1.00	0.21	2.89	100.00	300

Fig. 131: Studies included in prevalence of hypoxaemia among presenting children with diarrhea (SpO2<90)

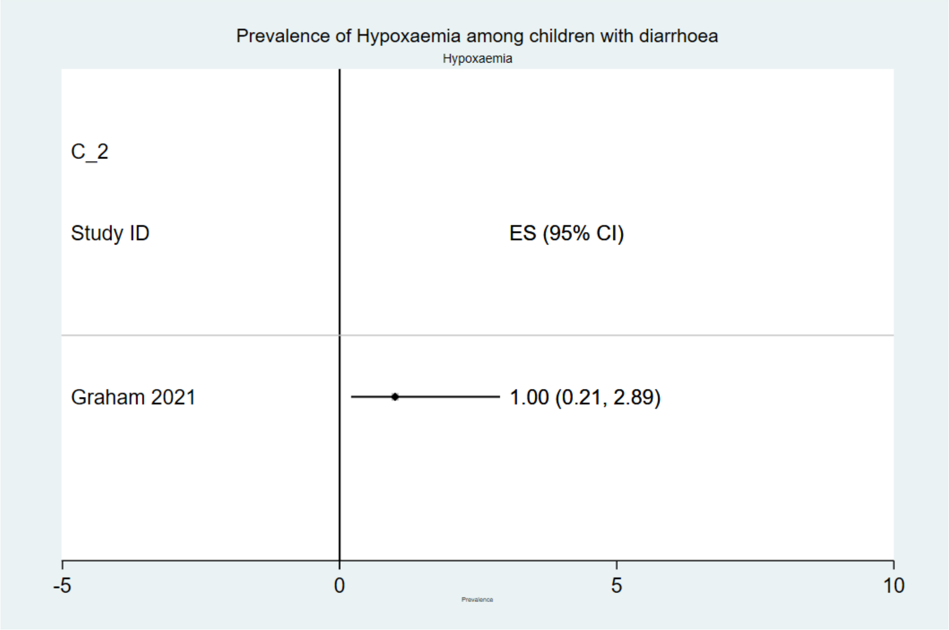

OPD/ED Adults – all

Table 132: Studies included in prevalence of hypoxaemia among all presenting adults

Serial	Study ID	WB Region	Proportion	LCL	UCL	Weight	Denominator
1.	Sutherland 2019	Sub Saharan Africa	12.10	10.61	13.71	35.01	1769
2.	Graham 2021	Sub Saharan Africa	0.09	0.02	0.27	64.99	3284

Fig. 132: Studies included in prevalence of hypoxaemia among all presenting adults

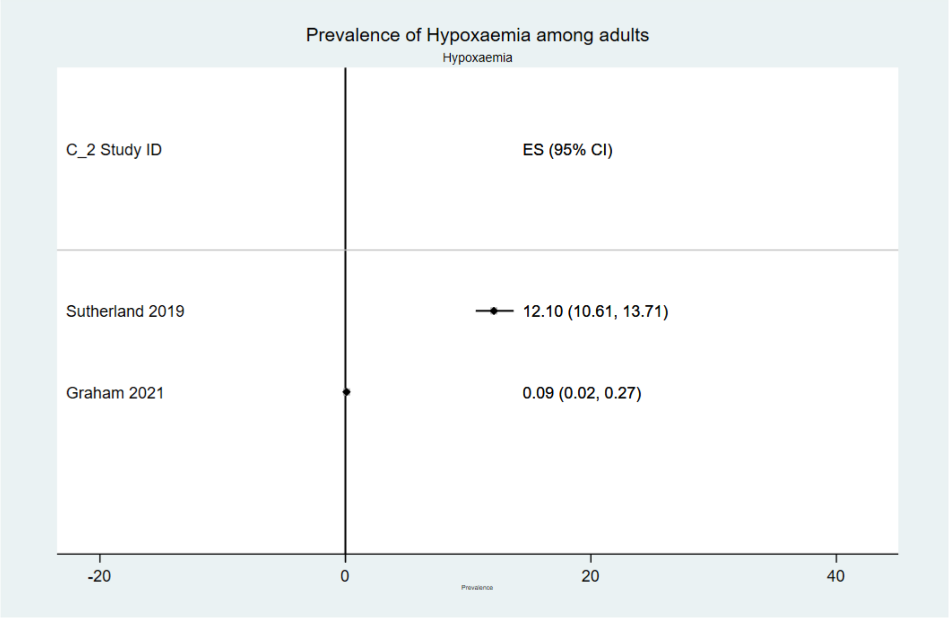

OPD/ED Adults – pneumonia

Table 133: Studies included in prevalence of hypoxaemia among presenting adults with any pneumonia (undefined) (SpO2<90)

Serial	Study ID	WB Region	Proportion	LCL	UCL	Weight	Denominator
1.	McCracken 2013	Latin America & Caribbean	35.71	25.55	46.92	25.61	84
2.	Friedman 2020	Latin America & Caribbean	34.03	30.57	37.62	26.58	720
3.	Hu 2020	East Asia & Pacific	4.70	2.85	7.25	26.48	404
4.	Graham 2021	Sub Saharan Africa	0.00	0.00	23.16	21.33	14
**Overall (I2=98.36%, T2=0.27, p<0.05)**			**15.65**	**1.37**	**39.43**		**1222**

Fig. 133: Studies included in prevalence of hypoxaemia among presenting adults with any pneumonia (undefined) (SpO2<90)

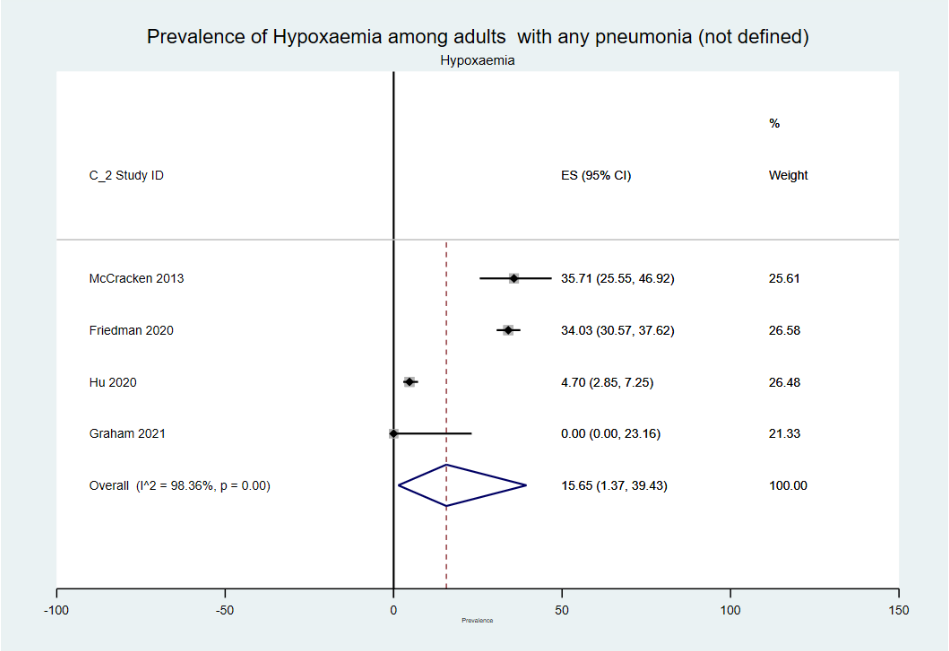

OPD/ED Adults – pneumonia radiological

Table 134: Studies included in prevalence of hypoxaemia among presenting adults with pneumonia (radiological) (SpO2<90)

Serial	Study ID	WB Region	Proportion	LCL	UCL	Weight	Denominator
1.	Hu 2020	East Asia & Pacific	4.70	2.85	7.25	100.00	404

Fig. 134: Studies included in prevalence of hypoxaemia among presenting adults with any pneumonia (radiological) (SpO2<90)

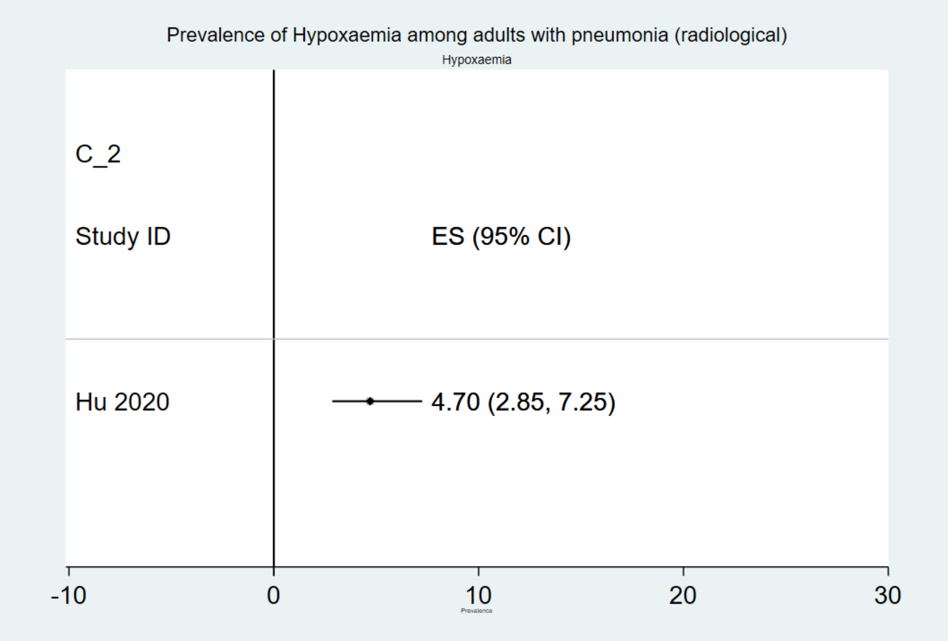

OPD/ED Adults – HIV complications

Table 135: Studies included in prevalence of hypoxaemia among presenting adults with HIV complications (SpO2<90)

Serial	Study ID	WB Region	Proportion	LCL	UCL	Weight	Denominator
1.	Laher 2022	Sub Saharan Africa	16.01	14.00	18.19	100.00	1224

Fig. 135: Studies included in prevalence of hypoxaemia among presenting adults with HIV complications (SpO2<90)

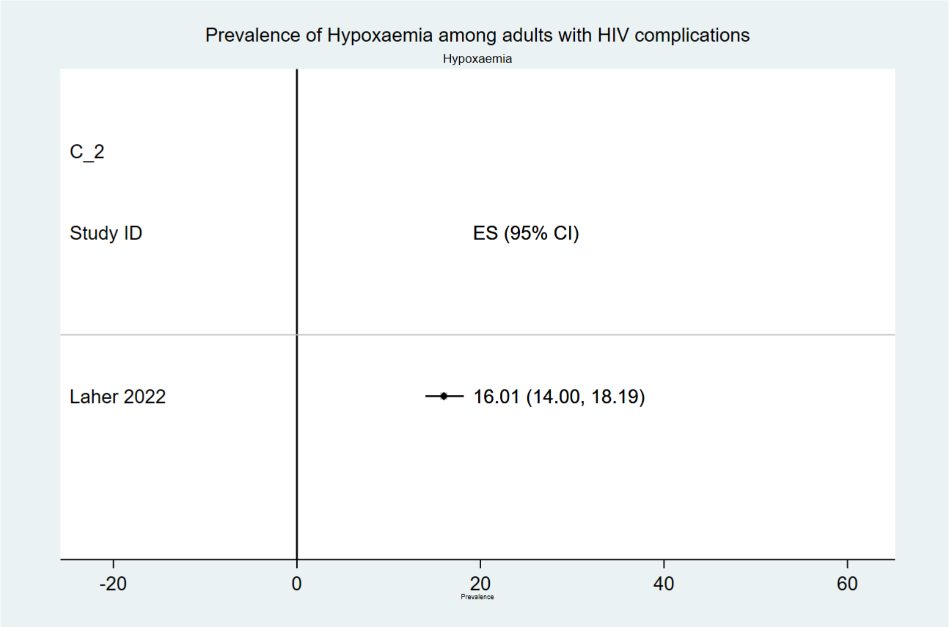

OPD/ED Adults – malaria

Table 136: Studies included in prevalence of hypoxaemia among presenting adults with malaria (SpO2<90)

Serial	Study ID	WB Region	Proportion	LCL	UCL	Weight	Denominator
1.	Graham 2021	Sub Saharan Africa	0.10	0.00	0.58	100.00	965

Fig. 136: Studies included in prevalence of hypoxaemia among presenting adults with malaria (SpO2<90)

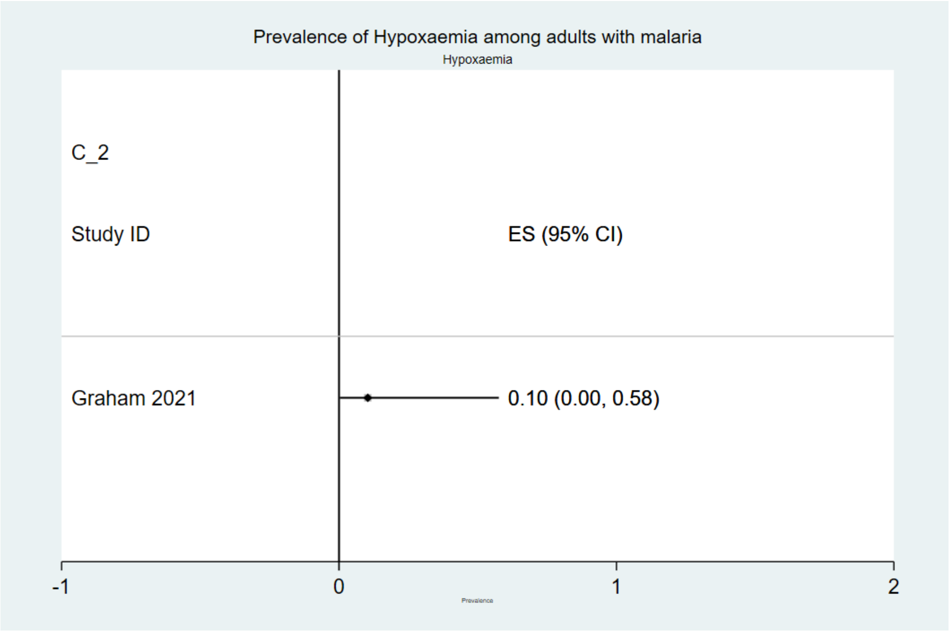

OPD/ED Adults – sepsis

Table 137: Studies included in prevalence of hypoxaemia among presenting adults with sepsis (SpO2<90)

Serial	Study ID	WB Region	Proportion	LCL	UCL	Weight	Denominator
1.	Graham 2021	0.00	0.00	6.38	100.00	56	

Fig. 137: Studies included in prevalence of hypoxaemia among presenting adults with sepsis (SpO2<90)

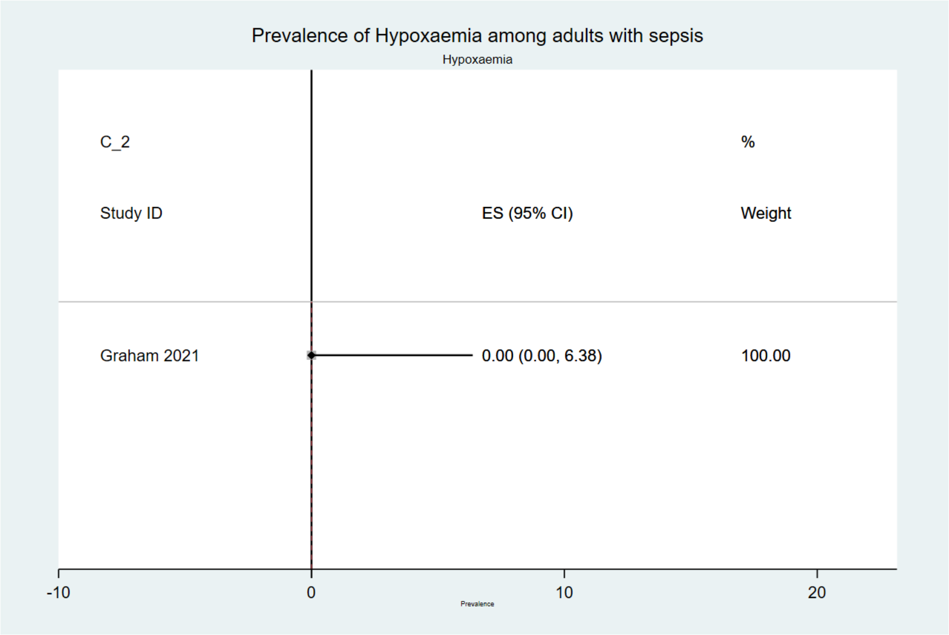

OPD/ED Adults – trauma / injury

Table 138: Studies included in prevalence of hypoxaemia among presenting adults with trauma (SpO2<90)

Serial	Study ID	WB Region	Proportion	LCL	UCL	Weight	Denominator
1.	Landes 2017	Sub Saharan Africa	20.59	15.26	26.79	23.37	204
2.	Krebs 2017	Sub Saharan Africa	3.43	2.19	5.11	76.63	670

Fig. 138: Studies included in prevalence of hypoxaemia among presenting adults with trauma (SpO2<90)

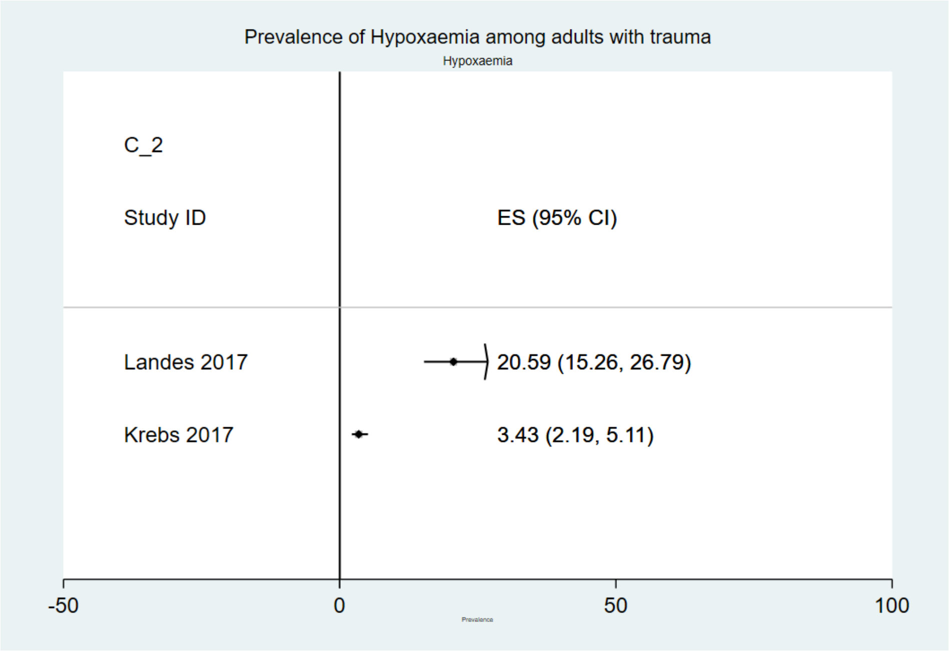

Tertiary Admitted Neonates

Table 139: Studies included in prevalence of hypoxaemia among all neonates in tertiary setting (SpO2<90)

Serial	Study ID	WB Region	Proportion	LCL	UCL	Weight	Denominator
1.	Junge 2006	Sub Saharan Africa	16.45	12.50	21.06	33.17	310
2.	Orimadegun 2013	Sub Saharan Africa	41.41	36.84	46.09	33.53	454
3.	Kiputa 2022	Sub Saharan Africa	21.26	17.08	25.94	33.29	348
**Overall (I2=97.08%, T2=0.09, p<0.05)**			**25.79**	**12.33**	**42.12**		**1112**

Fig. 139: Studies included in prevalence of hypoxaemia among all neonates in tertiary setting (SpO2<90)

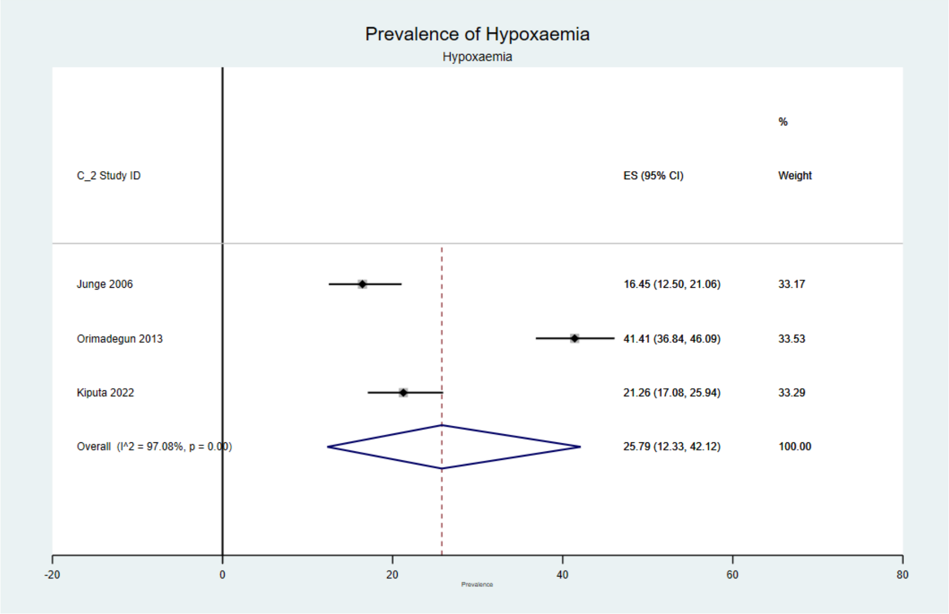

Tertiary Admitted Children

Table 140: Studies included in prevalence of hypoxaemia among all children in tertiary setting (SpO2<90)

Serial	Study ID	WB Region	Proportion	LCL	UCL	Weight	Denominator
1.	Junge 2006	Sub Saharan Africa	5.75	4.98	6.60	14.47	3269
2.	Orimadegun 2013	Sub Saharan Africa	24.06	21.73	26.50	14.38	1272
3.	Barennes 2016	East Asia & Pacific	13.71	10.29	17.77	14.01	350
4.	Lowlaavar 2016	Sub Saharan Africa	25.72	23.35	28.19	14.38	1291
5.	Hau 2018	Sub Saharan Africa	5.59	3.80	7.88	14.19	537
6.	Nielsen 2018	Latin America & Caribbean	8.58	6.91	10.50	14.34	991
7.	Krithika 2022	South Asia	8.17	6.10	10.65	14.22	600
**Overall (I2=98.81%, T2=0.08, p<0.05)**			**12.16**	**6.21**	**19.74**		**8310**

Fig. 140: Studies included in prevalence of hypoxaemia among all children in tertiary setting (SpO2<90)

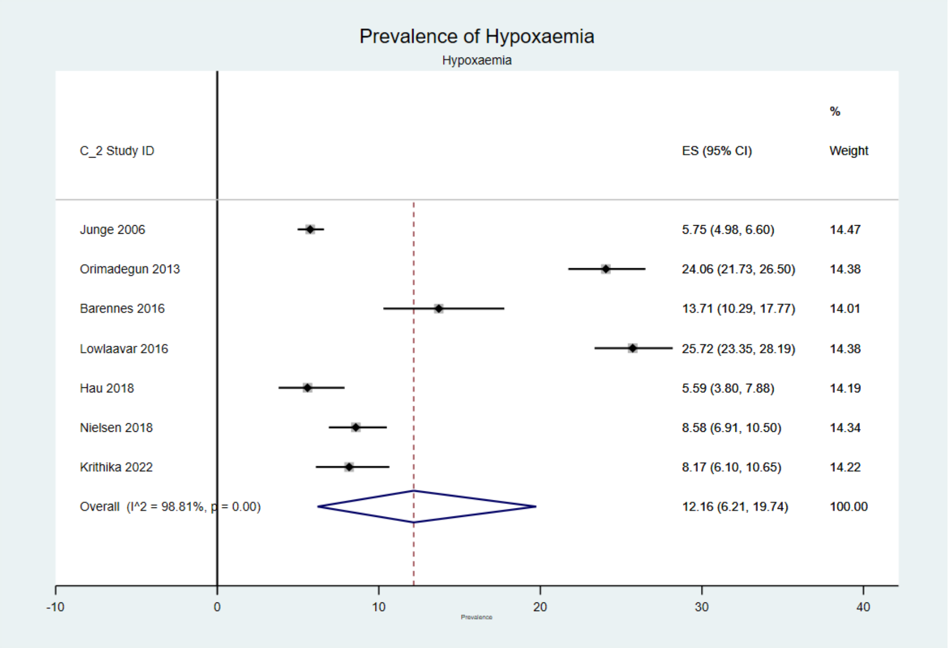

Tertiary Admitted Adults

Table 141: Studies included in prevalence of hypoxaemia among all adults in tertiary setting (SpO2<90)

Serial	Study ID	WB Region	Proportion	LCL	UCL	Weight	Denominator
1.	Evans 2012	Sub Saharan Africa	9.72	5.42	15.77	16.16	144
2.	Riviello 2016	Sub Saharan Africa	12.05	10.13	14.17	56.20	1046
3.	Aslam 2021	South Asia	15.17	11.25	19.83	27.64	290
**Overall (I2=1.97%, T2=0.00, p=0.38)**			**12.44**	**10.14**	**14.93**		**1480**

Fig. 141: Studies included in prevalence of hypoxaemia among all adults in tertiary setting (SpO2<90)

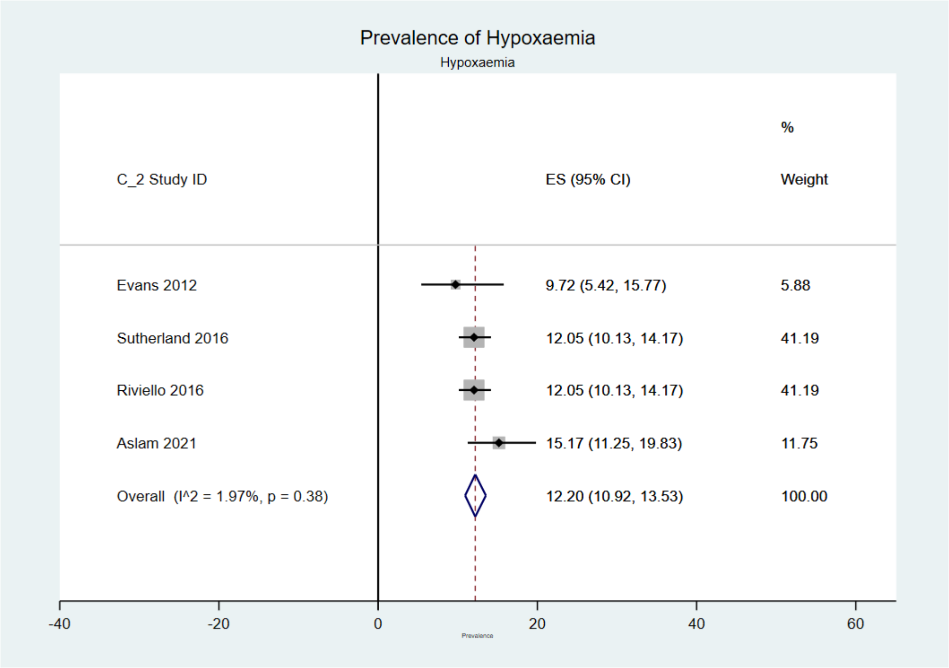

Tertiary Admitted Children – pneumonia all WHO-classified

Table 142: Studies included in prevalence of hypoxaemia among children with pneumonia in tertiary setting (SpO2<90)

Sl no	Study ID	WB Region	Proportion	LCL	UCL	Weight	Denominator
1.	West 1999	Sub Saharan Africa	43.68	36.52	51.05	3.53	190
2.	Addo-Yobo 2004	Mixed	19.10	17.25	21.04	3.66	1702
3.	Laman 2005	East Asia & Pacific	25.97	16.64	37.23	3.33	77
4.	Fu 2006	Mixed	19.30	17.45	21.26	3.66	1694
5.	Junge 2006	Sub Saharan Africa	11.70	8.83	15.09	3.61	436
6.	Puumalainen 2008	East Asia & Pacific	16.16	14.08	18.42	3.65	1151
7.	Asghar 2008	Mixed	64.61	61.49	67.65	3.65	958
8.	Sigauque 2009	Sub Saharan Africa	25.84	22.60	29.29	3.63	685
9.	Chisti 2011	South Asia	54.55	47.33	61.62	3.53	198
10	Ramakrishna 2012	Sub Saharan Africa	37.34	31.11	43.89	3.55	233
11	Jain 2013	South Asia	17.68	12.42	24.03	3.52	181
12	Orimadegun 2013	Sub Saharan Africa	49.20	43.53	54.88	3.58	313
13	Sempertegui 2014	Latin America & Caribbean	85.11	81.48	88.27	3.61	450
14	Ibraheem 2014	Sub Saharan Africa	41.50	34.59	48.66	3.53	200
15	Abdulkadir 2015	Sub Saharan Africa	41.50	34.59	48.66	3.53	200
16	Kelly 2015	Sub Saharan Africa	38.66	32.43	45.16	3.56	238
17	Basnet 2015	South Asia	61.15	57.15	65.04	3.63	610
18	Salah 2015	Sub Saharan Africa	42.67	34.64	50.99	3.49	150
19	Alwadhi 2017	South Asia	50.89	41.27	60.46	3.43	112
20	Fagbohun 2020	Latin America & Caribbean	13.02	10.85	15.46	3.64	860
21	Shahrin 2020	South Asia	15.18	10.41	21.07	3.53	191
22	Muro 2020	Sub Saharan Africa	32.40	26.64	38.58	3.56	250
23	Bui-Binh-Bao 2021	East Asia & Pacific	11.39	7.92	15.69	3.57	281
24	Chisti 2021	South Asia	28.13	26.74	29.55	3.67	4007
25	Rahman 2021	South Asia	39.98	38.11	41.88	3.66	2646
26	Chisti 2022	South Asia	31.23	29.69	32.80	3.67	3468
27	Jullien 2022	South Asia	75.51	67.74	82.22	3.49	147
28	Kapoor 2022	South Asia	36.11	29.10	43.59	3.52	180
Overall (I2=98.91%, T2=0.12, p<0.05)			35.86	29.62	42.34		21808

Fig. 142: Studies included in prevalence of hypoxaemia among children with pneumonia in tertiary setting (SpO2<90)

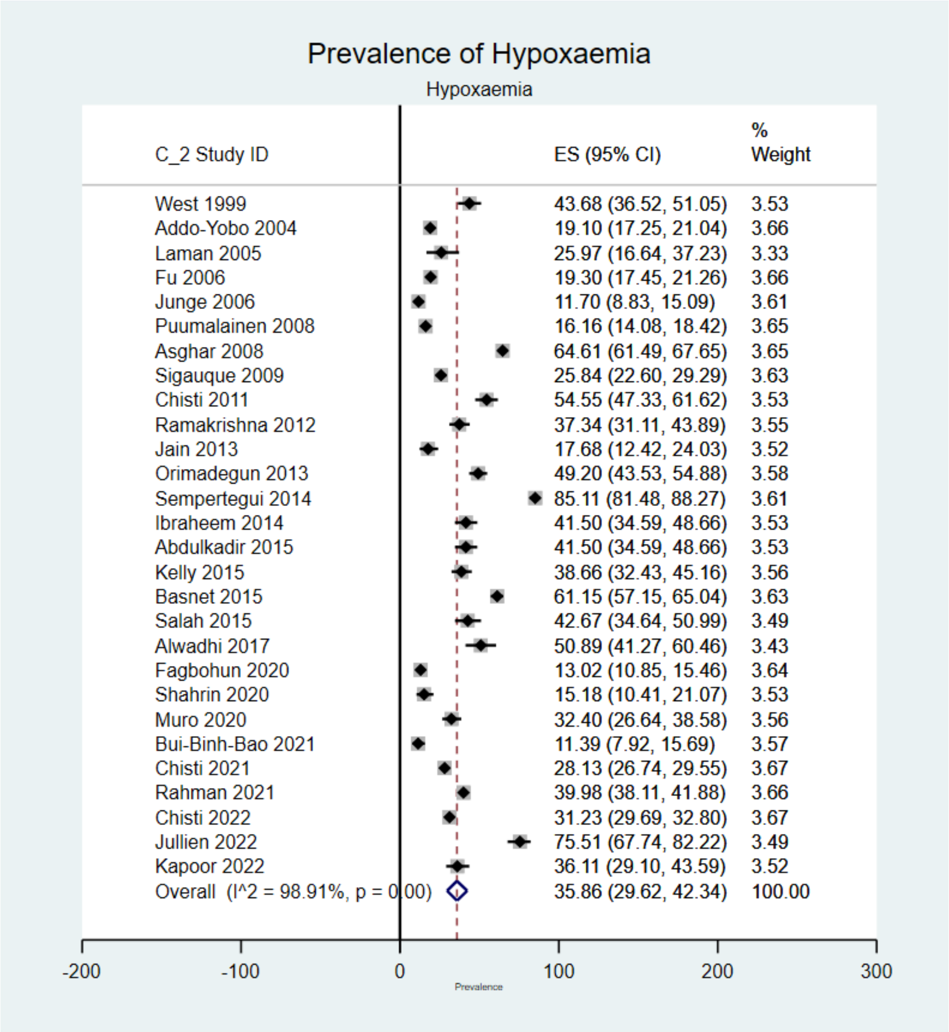

Tertiary Admitted Children – malaria

Table 143: Studies included in prevalence of hypoxaemia among children with malaria in tertiary setting (SpO2<90)

Serial	Study ID	WB Region	Proportion	LCL	UCL	Weight	Denominator
1.	Junge 2006	Sub Saharan Africa	2.87	1.95	4.08	25.12	1044
2.	Cserti-Gazdewich 2013	Sub Saharan Africa	2.26	1.64	3.03	25.21	1901
3.	Orimadegun 2013	Sub Saharan Africa	14.42	11.53	17.71	24.92	527
4.	Orimadegun 2014	Sub Saharan Africa	29.81	25.19	34.76	24.75	369
**Overall (I2=98.98%, T2=0.12, p<0.05)**			**9.94**	**2.28**	**22.09**		**3841**

Fig. 143: Studies included in prevalence of hypoxaemia among children with malaria in tertiary setting (SpO2<90)

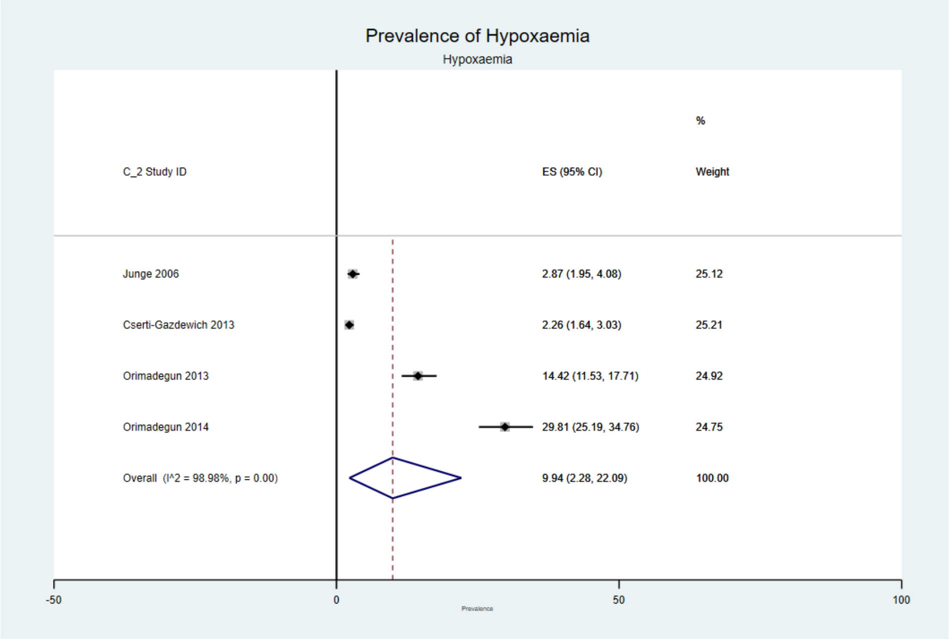

Tertiary Admitted Children – sepsis

Table 144: Studies included in prevalence of hypoxaemia among children with sepsis in tertiary setting (SpO2<90)

Serial	Study ID	WB Region	Proportion	LCL	UCL	Weight	Denominator
1.	Orimadegun 2013	Sub Saharan Africa	22.62	16.53	29.70	35.41	168
2.	Shahid 2016	South Asia	23.08	8.97	43.65	7.94	26
3.	Shahunja 2020	South Asia	16.96	13.42	21.00	56.65	401
**Overall (I2=31.09%, T2=0.00, p=0.23)**			**19.07**	**14.78**	**23.76**		**595**

Fig. 144: Studies included in prevalence of hypoxaemia among children with sepsis in tertiary setting (SpO2<90)

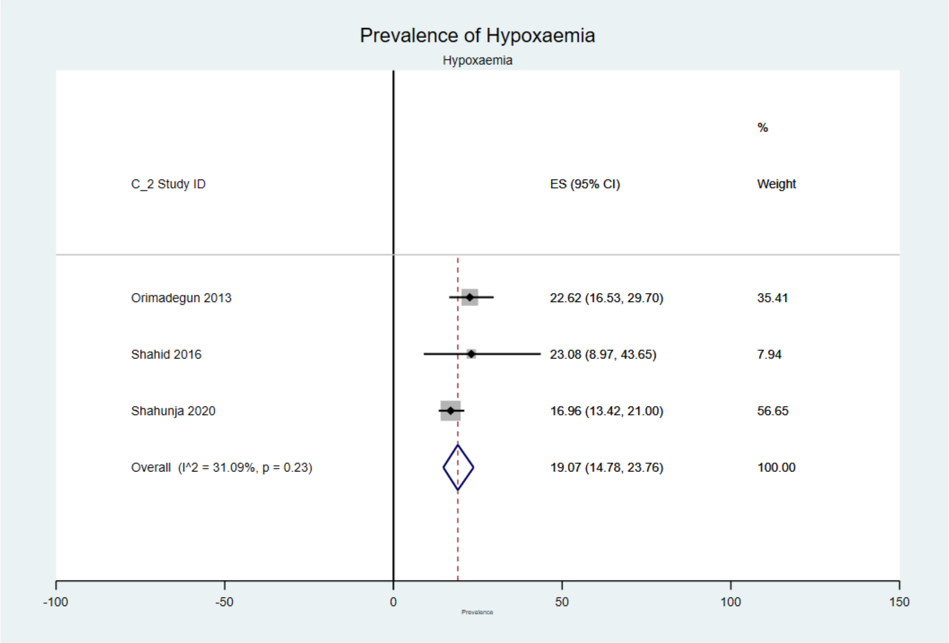

Tertiary Admitted Children – malnutrition

Table 145: Studies included in prevalence of hypoxaemia among children with malnutrition in tertiary setting (SpO2<90)

Serial	Study ID	WB Region	Proportion	LCL	UCL	Weight	Denominator
1.	Junge 2006	Sub Saharan Africa	1.85	0.60	4.25	33.06	271
2.	Chisti 2013	South Asia	11.08	7.92	14.95	33.57	334
3.	Faruk 2022	South Asia	9.80	6.71	13.70	33.37	306
**Overall (I2=92.72%, T2=0.04, p<0.05)**			**6.90**	**2.02**	**14.27**		**911**

Fig. 145: Studies included in prevalence of hypoxaemia among children with malnutrition in tertiary setting (SpO2<90)

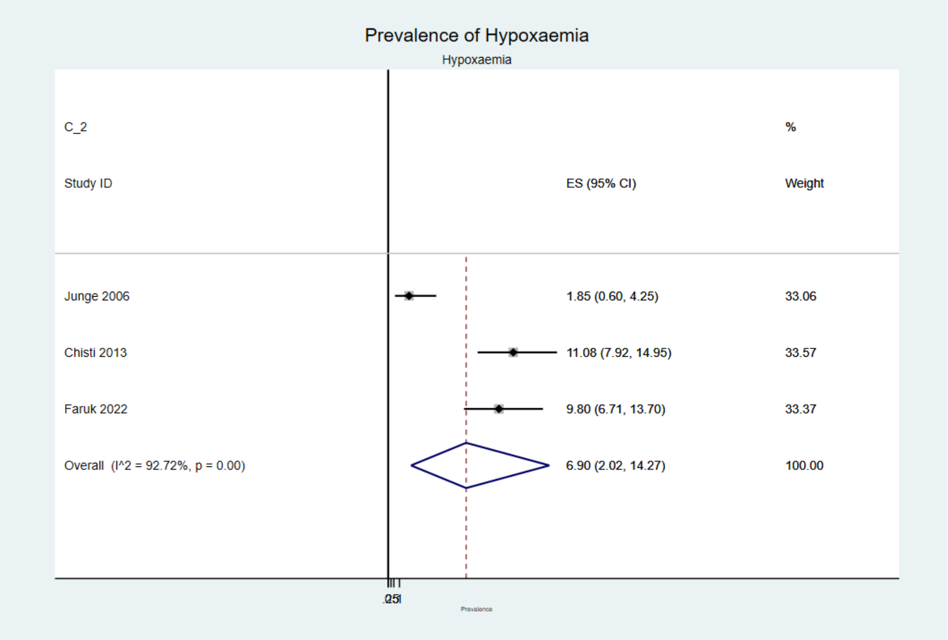

Secondary Admitted Neonates

Table 146: Studies included in prevalence of hypoxaemia among all neonates in secondary setting (SpO2<90)

Serial	Study ID	WB Region	Proportion	LCL	UCL	Weight	Denominator
1.	English 2003	Sub Saharan Africa	19.97	16.88	23.35	6.45	616
2.	Mwaniki 2009	Sub Saharan Africa	18.64	16.39	21.07	11.57	1105
3.	McCollum 2013	Sub Saharan Africa	20.59	8.70	37.90	0.36	34
4.	Graham 2019	Sub Saharan Africa	18.24	17.37	19.13	78.20	7473
5.	Graham 2022	Sub Saharan Africa	16.26	12.42	20.72	3.42	326
**Overall (I2=0.00%, T2=0.00, p=0.67)**			**18.13**	**17.35**	**18.92**		**9554**

Fig. 146: Studies included in prevalence of hypoxaemia among all neonates in secondary setting (SpO2<90)

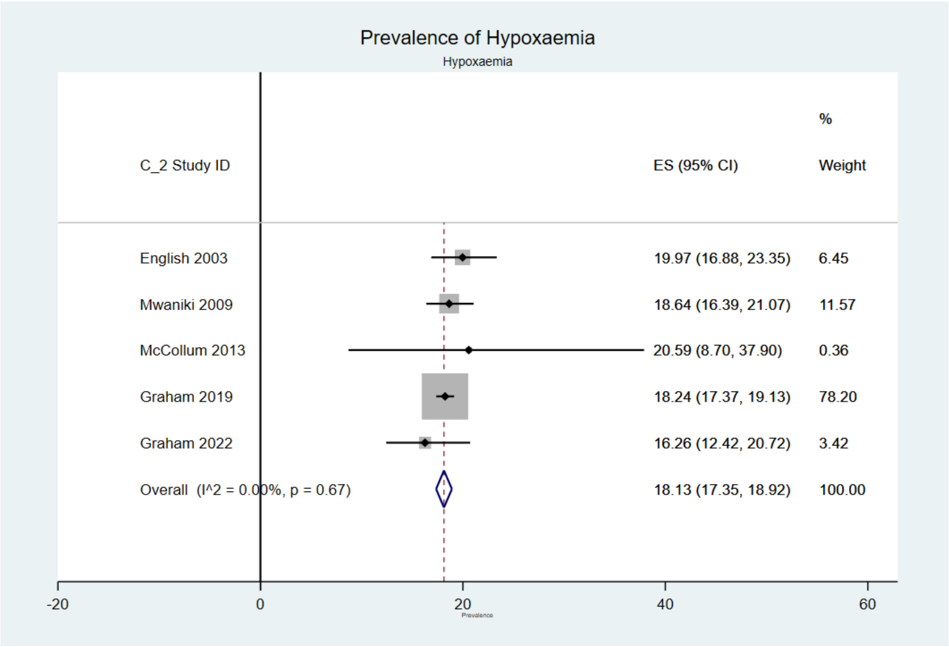

Secondary Admitted Children

Table 147: Studies included in prevalence of hypoxaemia among all children in secondary setting (SpO2<90)

Serial	Study ID	WB Region	Proportion	LCL	UCL	Weight	Denominator
1.	English 2003	Sub Saharan Africa	14.04	10.27	18.56	9.54	292
2.	Wandi 2006	East Asia & Pacific	24.16	22.24	26.15	12.10	1896
3.	Mwaniki 2009	Sub Saharan Africa	5.26	4.88	5.65	12.63	13183
4.	Foran 2010	Sub Saharan Africa	6.02	1.98	13.50	5.86	83
5.	McCollum 2013	Sub Saharan Africa	4.54	3.14	6.32	11.22	727
6.	Graham 2019	Sub Saharan Africa	7.93	7.52	8.35	12.65	16453
7.	Enoch 2019	Sub Saharan Africa	10.00	9.51	10.50	12.64	14232
8.	Tuti 2021	Sub Saharan Africa	6.97	6.77	7.17	12.70	64722
9.	Graham 2022	Sub Saharan Africa	11.26	8.64	14.35	10.67	506
**Overall (I2=98.88%, T2=0.01, p<0.05)**			**9.51**	**7.52**	**11.70**		**112094**

Fig. 147: Studies included in prevalence of hypoxaemia among all children in secondary setting (SpO2<90)

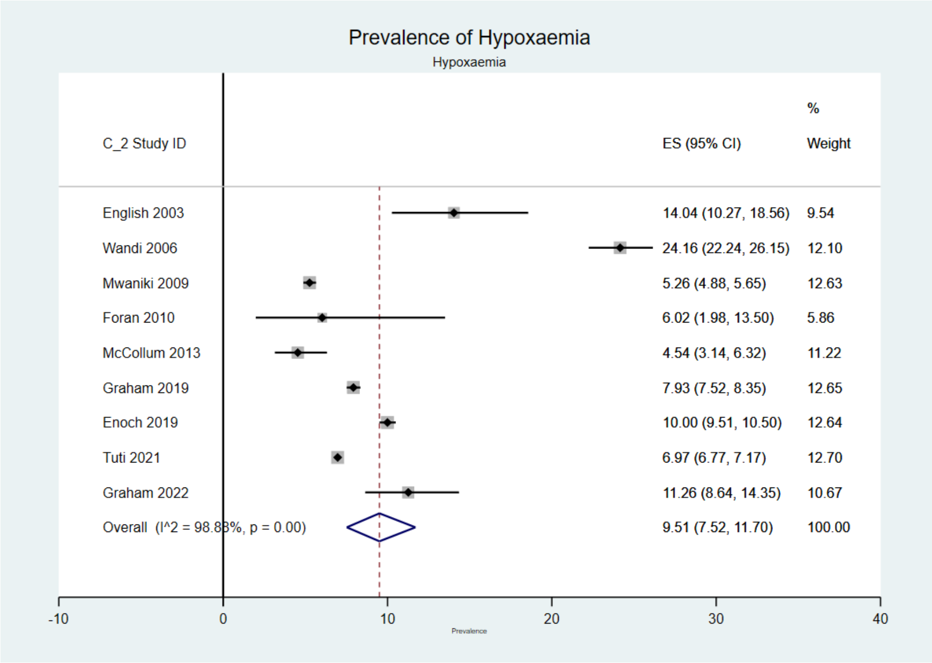

Secondary Admitted Adults

Table 148: Studies included in prevalence of hypoxaemia among all adults in secondary setting (SpO2<90)

Serial	Study ID	WB Region	Proportion	LCL	UCL	Weight	Denominator
1.	Foran 2010	Sub Saharan Africa	9.17	4.49	16.23	4.04	109
2.	Wasingya-Kasereka 2020	Sub Saharan Africa	6.31	5.41	7.31	95.96	2599

Fig. 148: Studies included in prevalence of hypoxaemia among all adults in secondary setting (SpO2<90)

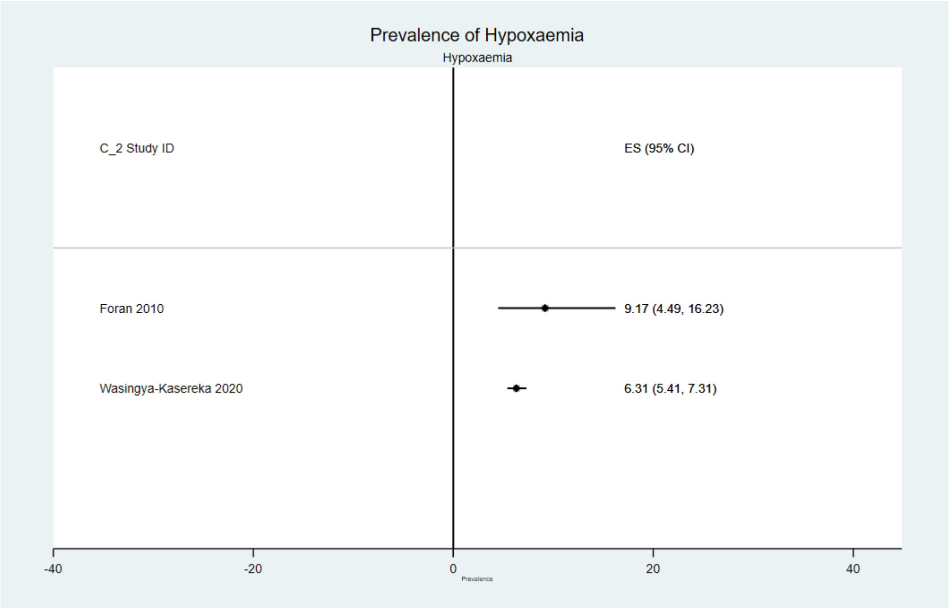

Secondary Admitted Children – pneumonia all WHO-classified

Table 149: Studies included in prevalence of hypoxaemia among children with pneumonia in secondary setting (SpO2<90)

Serial	Study ID	WB Region	Proportion	LCL	UCL	Weight	Denominator
1.	Wandi 2006	East Asia & Pacific	54.50	50.34	58.61	7.19	578
2.	Duke 2008	East Asia & Pacific	57.09	52.94	61.17	7.19	578
3.	Mwaniki 2009	Sub Saharan Africa	8.40	7.68	9.16	7.25	5489
4.	Webb 2012	Sub Saharan Africa	23.59	20.16	27.30	7.19	568
5.	Kuti 2013	Sub Saharan Africa	19.29	15.62	23.39	7.16	420
6.	McCollum 2013	Sub Saharan Africa	17.22	11.57	24.20	7.01	151
7.	Kuti 2013	Sub Saharan Africa	19.76	16.01	23.94	7.16	410
8.	Bassat 2016	Sub Saharan Africa	27.88	24.84	31.07	7.21	825
9.	Graham 2019	Sub Saharan Africa	23.44	21.64	25.33	7.24	2073
10.	McCollum 2019	Sub Saharan Africa	64.44	60.61	68.14	7.20	644
11.	Oktaria 2021	East Asia & Pacific	13.53	8.22	20.54	6.98	133
12.	Ahmed 2022	Sub Saharan Africa	75.32	64.18	84.44	6.79	77
13.	Awasthi 2022	South Asia	35.85	34.74	36.97	7.25	7196
14.	Mvalo 2022	Sub Saharan Africa	63.75	59.53	67.82	7.18	538
**Overall (I2=99.55%, T2=0.19, p<0.05)**			**34.61**	**24.11**	**45.91**		**19680**

Fig. 149: Studies included in prevalence of hypoxaemia among children with pneumonia in secondary setting (SpO2<90)

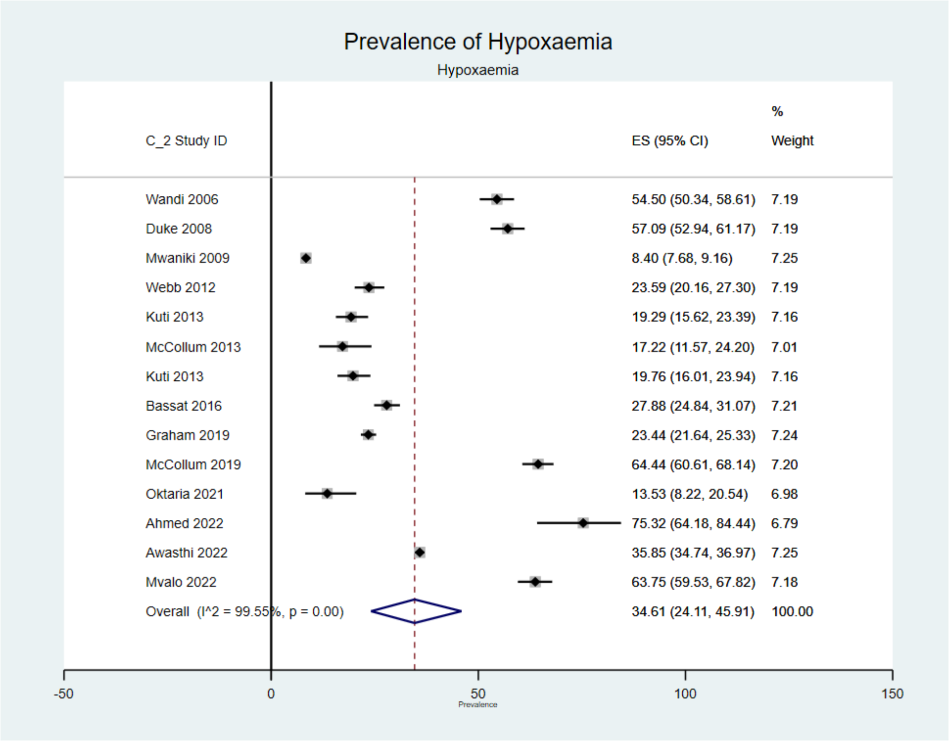

Secondary Admitted Children – malaria

Table 150: Studies included in prevalence of hypoxaemia among children with malaria in secondary setting (SpO2<90)

Serial	Study ID	WB Region	Proportion	LCL	UCL	Weight	Denominator
1.	Maitland 2003	Sub Saharan Africa	17.17	13.97	20.76	16.29	501
2.	Wandi 2006	East Asia & Pacific	3.31	1.52	6.19	15.22	272
3.	Mwaniki 2009	Sub Saharan Africa	4.90	4.31	5.53	17.62	4982
4.	McCollum 2013	Sub Saharan Africa	1.87	0.81	3.66	16.06	427
5.	Graham 2019	Sub Saharan Africa	8.50	7.74	9.31	17.62	5035
6.	Leligdowicz 2021	Sub Saharan Africa	2.51	1.73	3.50	17.18	1317
**Overall (I2=97.33%, T2=0.19, p<0.05)**			**5.58**	**3.10**	**8.73**		**12534**

Fig. 150: Studies included in prevalence of hypoxaemia among children with malaria in secondary setting (SpO2<90)

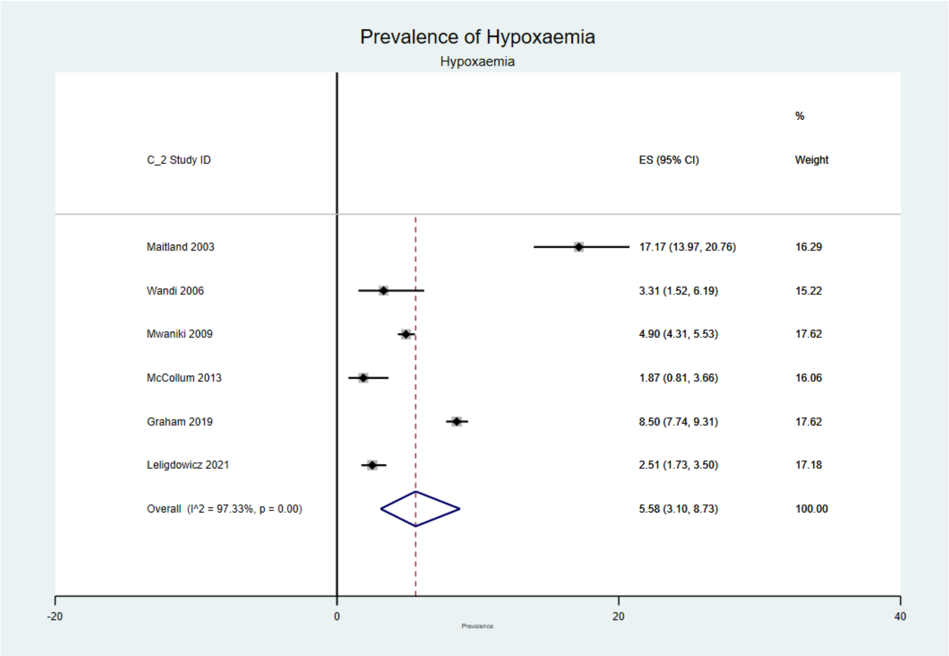

Secondary Admitted Children – sepsis

Table 151: Studies included in prevalence of hypoxaemia among children with sepsis in secondary setting (SpO2<90)

Serial	Study ID	WB Region	Proportion	LCL	UCL	Weight	Denominator
1.	McCollum 2013	Sub Saharan Africa	3.45	0.09	17.76	0.72	29
2.	Graham 2019	Sub Saharan Africa	8.70	7.85	9.61	99.28	4092

Fig. 151: Studies included in prevalence of hypoxaemia among children with sepsis in secondary setting (SpO2<90)

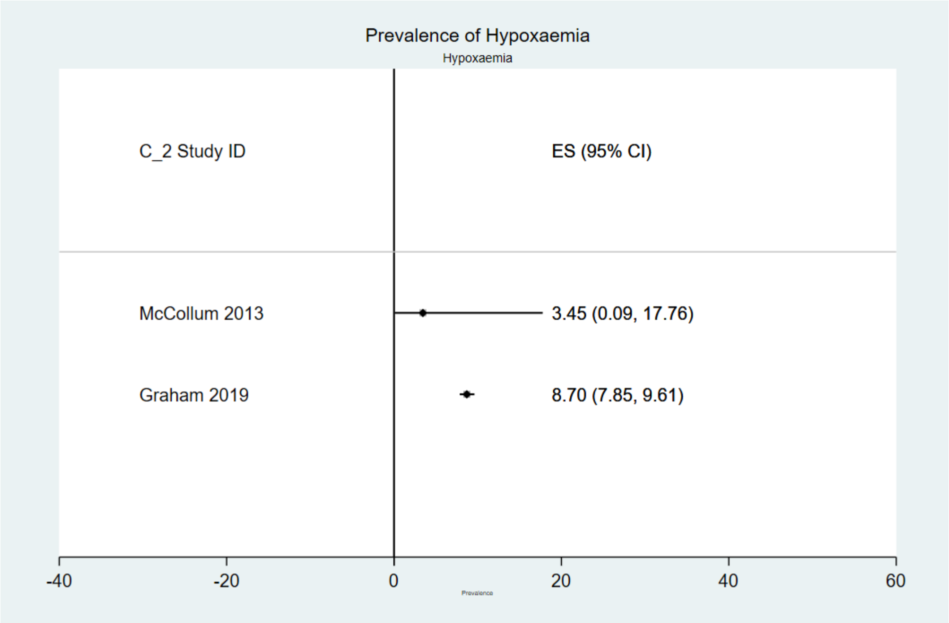

Secondary Admitted Children – malnutrition

Table 152: Studies included in prevalence of hypoxaemia among children with malnutrition in secondary setting (SpO2<90)

Serial	Study ID	WB Region	Proportion	LCL	UCL	Weight	Denominator
1.	Wandi 2006	East Asia & Pacific	8.33	0.21	38.48	18.78	12
2.	McCollum 2013	Sub Saharan Africa	5.26	0.13	26.03	21.80	19
3.	Graham 2019	Sub Saharan Africa	3.50	2.56	4.65	30.43	1286
4.	Kintwa 2021	East Asia & Pacific	20.71	14.33	28.38	28.99	140
**Overall (I2=92.93%, T2=0.13, p<0.05)**			**8.47**	**0.33**	**23.12**		**1457**

Fig. 152: Studies included in prevalence of hypoxaemia among children with malnutrition in secondary setting (SpO2<90)

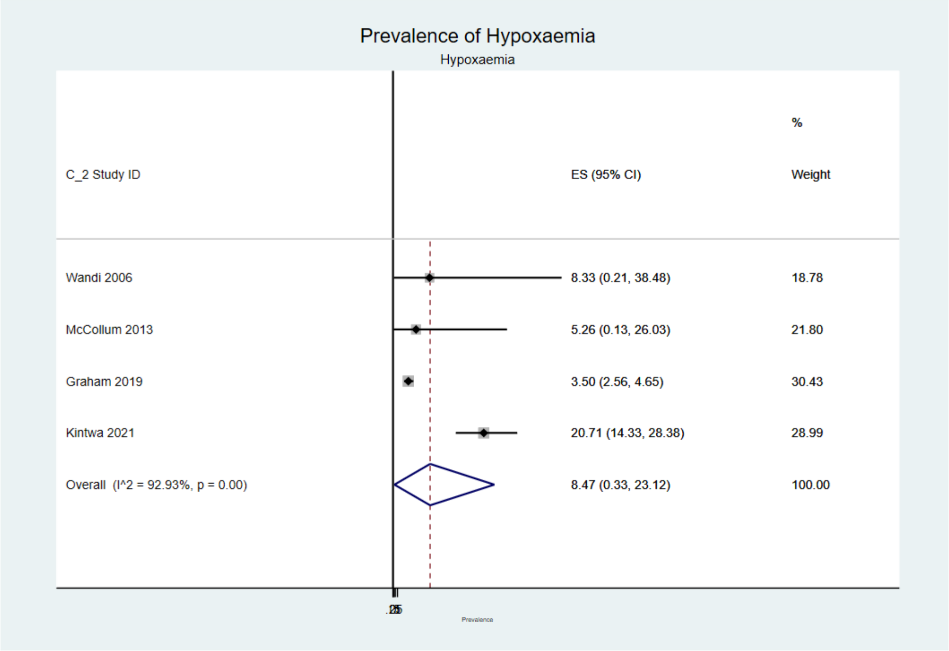

Relative Odds of Death - neonates

Table 153: Studies included in odds ratio of mortality (hypoxaemic vs. normoxaemic) among all neonates (SpO2<90)

Serial	StudyID	Region	OR	LCL	UCL	Weight	Total
1.	Kiputa 2022	Sub Saharan Africa	6.18	3.51	10.87	100	348

Fig. 153: Studies included in odds ratio of mortality (hypoxaemic vs. normoxaemic) among all neonates (SpO2<90)

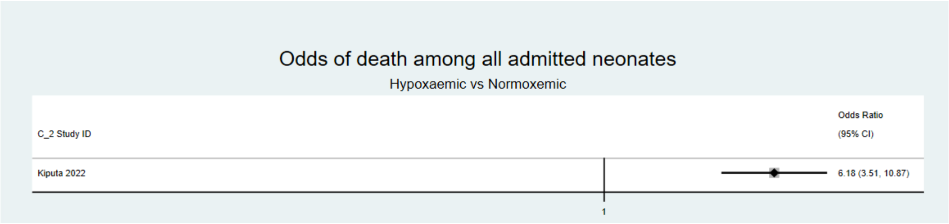

Relative Odds of Death – children

Table 154: Studies included in odds ratio of mortality (hypoxaemic vs. normoxaemic) among all children (SpO2<90)

Serial	StudyID	Region	OR	LCL	UCL	Weight	Total
1.	Usen 1999	Sub Saharan Africa	5.10	2.22	11.70	3.55	1072
2.	West 1999	Sub Saharan Africa	2.71	0.77	9.56	2.91	118
3.	Asghar 2008	Mixed	2.55	1.34	4.85	3.82	958
4.	Sigauque 2009	Sub Saharan Africa	2.19	1.13	4.25	3.79	584
5.	Chisti 2011	South Asia	8.98	3.02	26.64	3.17	258
6.	Ramakrishna 2012	Sub Saharan Africa	6.52	2.49	17.07	3.36	233
7.	Chisti 2013	South Asia	22.67	5.98	85.92	2.8	140
8.	Cserti-Gazdewich 2013	Sub Saharan Africa	4.79	2.23	10.29	3.65	849
9.	Kuti 2013	Sub Saharan Africa	3.05	1.05	8.87	3.2	389
10.	Orimadegun 2014	Sub Saharan Africa	4.71	2.16	10.27	3.63	369
11.	Abdulkadir 2015	Sub Saharan Africa	61.84	3.66	1044.98	1.27	200
12.	Lowlaavar 2016	Sub Saharan Africa	2.88	1.72	4.81	3.97	1291
13.	Bassat 2016	Sub Saharan Africa	3.00	1.87	4.81	4.02	825
14.	Barennes 2016	East Asia & Pacific	4.45	1.74	11.39	3.39	350
15.	Benet 2017	Mixed	3.83	1.29	11.42	3.16	405
16.	Alwadhi 2017	South Asia	9.34	0.49	177.63	1.19	112
17.	Zampoli 2017	Sub Saharan Africa	7.78	1.01	59.81	1.91	206
18.	Hau 2018	Sub Saharan Africa	4.55	2.12	9.76	3.65	537
19.	McCollum 2019	Sub Saharan Africa	1.02	0.64	1.63	4.02	644
20.	Ma 2019	Sub Saharan Africa	3.94	0.50	30.88	1.89	155
21.	Hooli 2020	Sub Saharan Africa	4.60	2.32	9.14	3.76	1491
22.	Shahunja 2020	South Asia	14.73	7.35	29.52	3.75	401
23.	Fagbohun 2020	Latin America & Caribbean	11.07	6.23	19.66	3.9	860
24.	Shahrin 2020	South Asia	4.53	1.44	14.27	3.08	176
25.	Bui-Binh-Bao 2021	East Asia & Pacific	69.44	8.21	587.42	1.82	281
26.	Chisti 2021	South Asia	13.55	9.24	19.88	4.1	4007
27.	Kintwa 2021	East Asia & Pacific	10.49	3.62	30.39	3.2	140
28.	Krithika 2022	South Asia	191.45	76.61	478.48	3.43	600
29.	Awasthi 2022	South Asia	3.40	2.06	5.59	3.99	7196
30.	Chisti 2022	South Asia	14.60	9.77	21.83	4.09	3468
31.	Kapoor 2022	South Asia	16.95	3.74	76.92	2.55	180
**Overall(I2=86.0%, p<0.05)**			6.48	4.435	9.467		28495

Fig. 154: Studies included in odds ratio of mortality (hypoxaemic vs. normoxaemic) among all children (SpO2<90)

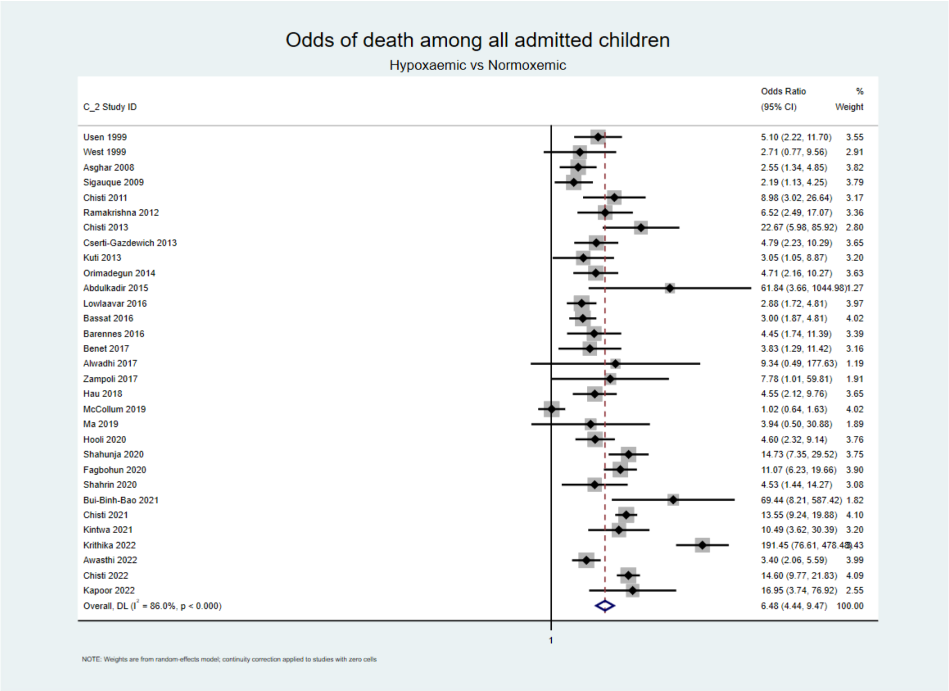

Relative Odds of Death – children with primary respiratory disease

Table 155: Studies included in odds ratio of mortality (hypoxaemic vs. normoxaemic) among children with respiratory disease (SpO2<90)

Serial	StudyID	Region	OR	LCL	UCL	Weight	Total
1.	West 1999	Sub Saharan Africa	2.71	0.77	9.56	5.34	118
2.	Asghar 2008	Mixed	2.55	1.34	4.85	6.94	958
3.	Sigauque 2009	Sub Saharan Africa	2.19	1.13	4.25	6.89	584
4.	Ramakrishna 2012	Sub Saharan Africa	6.52	2.49	17.07	6.14	233
5.	Kuti 2013	Sub Saharan Africa	3.05	1.05	8.87	5.86	389
6.	Abdulkadir 2015	Sub Saharan Africa	61.84	3.66	1044.98	2.36	200
7.	Benet 2017	Mixed	3.83	1.29	11.42	5.79	405
8.	Alwadhi 2017	South Asia	9.34	0.49	177.63	2.23	112
9.	Zampoli 2017	Sub Saharan Africa	7.78	1.01	59.81	3.55	206
10.	McCollum 2019	Sub Saharan Africa	1.02	0.64	1.63	7.3	644
11.	Ma 2019	Sub Saharan Africa	3.94	0.50	30.88	3.51	155
12.	Hooli 2020	Sub Saharan Africa	4.60	2.32	9.14	6.84	1491
13.	Fagbohun 2020	Latin America & Caribbean	11.07	6.23	19.66	7.09	860
14.	Bui-Binh-Bao 2021	East Asia & Pacific	69.44	8.21	587.42	3.37	281
15.	Chisti 2021	South Asia	13.55	9.24	19.88	7.45	4007
16.	Awasthi 2022	South Asia	3.40	2.06	5.59	7.25	7196
17.	Chisti 2022	South Asia	14.60	9.77	21.83	7.42	3468
18.	Kapoor 2022	South Asia	16.95	3.74	76.92	4.69	180
**Overall(I2=86.9%, p<0.05)**			**5.652**	**3.356**	**9.519**		**21487**

Fig. 155: Studies included in odds ratio of mortality (hypoxaemic vs. normoxaemic) among children with respiratory disease (SpO2<90)

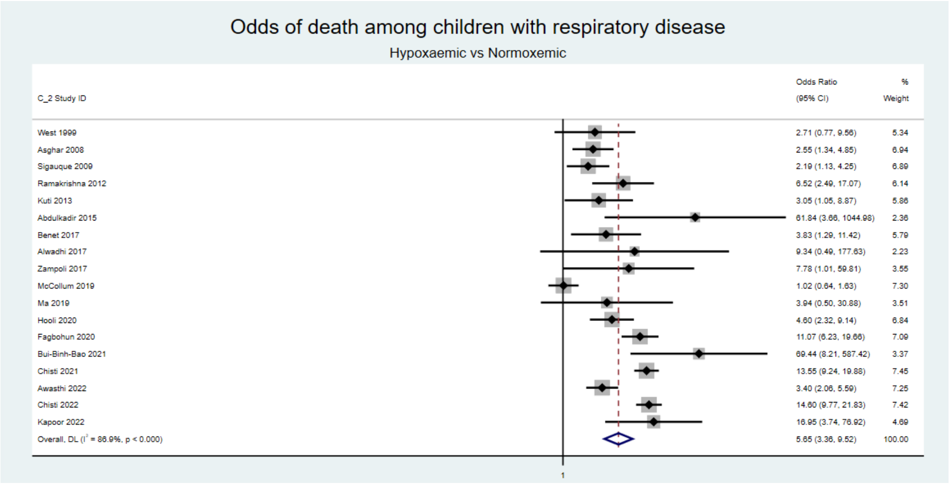

Relative Odds of Death – children with non-primary respiratory disease

Table 156: Studies included in odds ratio of mortality (hypoxaemic vs. normoxaemic) among children with non-respiratory disease (SpO2<90)

Serial	StudyID	Region	OR	LCL	UCL	Weight	Total
1.	Cserti-Gazdewich 2013	Sub Saharan Africa	4.79	2.23	10.29	26.45	849
2.	Orimadegun 2014	Sub Saharan Africa	4.71	2.16	10.27	26.01	369
3.	Shahunja 2020	South Asia	14.73	7.35	29.52	28.57	401
4.	Kintwa 2021	East Asia & Pacific	10.49	3.62	30.39	18.97	140
**Overall(I2=55.1%, p=0.083)**			7.624	4.163	13.965		1759

Fig. 156: Studies included in odds ratio of mortality (hypoxaemic vs. normoxaemic) among children with non-respiratory disease (SpO2<90)

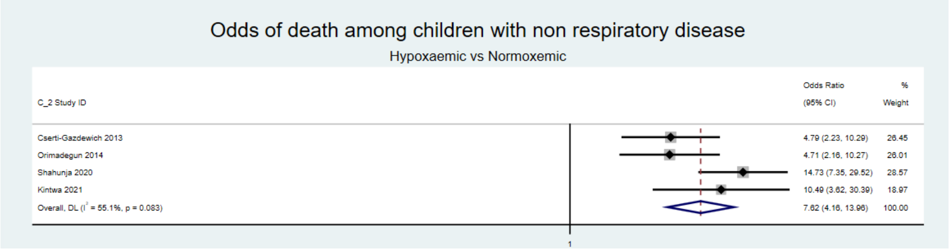

Relative Odds of Death - adults

Table 157: Studies included in odds ratio of mortality (hypoxaemic vs. normoxaemic) among all adults (SpO2<90)

Serial	StudyID	Region	OR	LCL	UCL	Weight	Total
1.	Amare 2008	Sub Saharan Africa	1.29	0.48	3.50	4.32	119
2.	Tokman 2014	Sub Saharan Africa	3.67	1.54	8.75	4.61	241
3.	Koss 2015	Sub Saharan Africa	2.47	1.63	3.74	5.51	835
4.	Carugati 2018	Sub Saharan Africa	3.83	1.76	8.34	4.81	419
5.	Worodria 2018	Sub Saharan Africa	3.70	2.72	5.03	5.65	1887
6.	Aston 2019	Sub Saharan Africa	5.38	2.99	9.70	5.2	431
7.	YeLynn 2019	East Asia & Pacific	0.33	0.13	0.83	4.46	118
8.	Boonmee 2020	East Asia & Pacific	2.17	1.72	2.75	5.73	1616
9.	Homayounieh 2020	Middle East & North Africa	2.63	0.81	8.46	3.93	75
10.	Wasingya-Kasereka 2020	Sub Saharan Africa	10.19	6.87	15.11	5.54	2599
11.	Mejia 2020	Latin America & Caribbean	5.80	3.42	9.83	5.32	500
12.	Diaz-Velez 2021	Latin America & Caribbean	4.54	3.09	6.68	5.55	493
13.	Padmaprakash 2021	South Asia	86.51	47.82	156.49	5.2	1536
14.	Anyaypoma-Ocon 2021	Latin America & Caribbean	1.11	0.72	1.71	5.47	324
15.	MarMinn 2021	East Asia & Pacific	3.86	1.96	7.60	5.02	507
16.	Kayambankadzanja 2021	Sub Saharan Africa	3.92	1.87	8.23	4.89	1135
17.	Xiong 2021	East Asia & Pacific	40.44	20.75	78.83	5.05	799
18.	Marcolino 2021	Latin America & Caribbean	3.13	2.37	4.14	5.69	1907
19.	Arana-Calderon 2022	Latin America & Caribbean	26.86	3.43	210.29	2.34	158
20.	Soto 2022	Latin America & Caribbean	5.12	3.99	6.58	5.72	1323
**Overall(I2=93.3%, p<0.05)**			4.391	2.926	6.588		17022

Fig. 157: Studies included in odds ratio of mortality (hypoxaemic vs. normoxaemic) among all adults (SpO2<90)

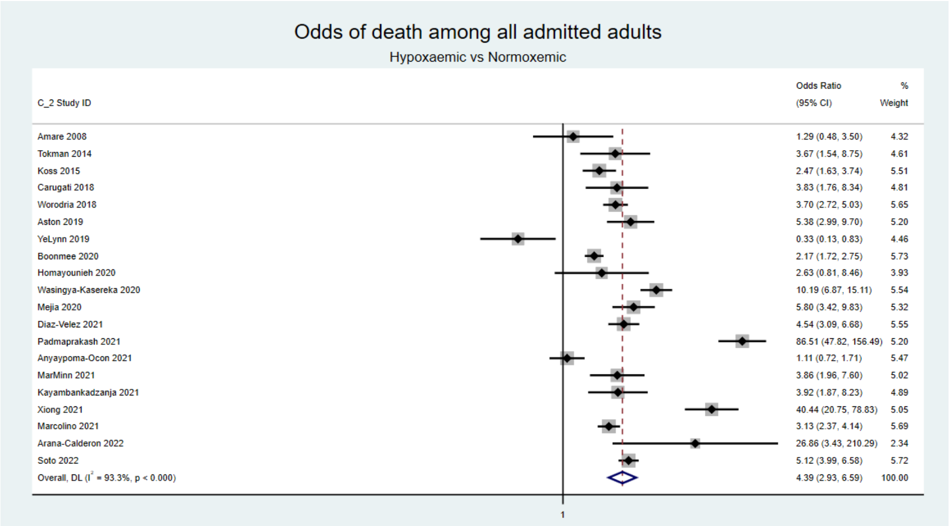

Relative Odds of Death – adults with primary respiratory disease

Table 158: Studies included in odds ratio of mortality (hypoxaemic vs. normoxaemic) among adults with respiratory disease (SpO2<90)

Serial	StudyID	Region	OR	LCL	UCL	Weight	Total
1.	Tokman 2014	Sub Saharan Africa	3.67	1.54	8.75	8.73	241
2.	Aston 2019	Sub Saharan Africa	5.38	2.99	9.70	9.56	431
3.	Homayounieh 2020	Middle East & North Africa	2.63	0.81	8.46	7.72	75
4.	Mejia 2020	Latin America & Caribbean	5.80	3.42	9.83	9.71	500
5.	Diaz-Velez 2021	Latin America & Caribbean	4.54	3.09	6.68	10.02	493
6.	Padmaprakash 2021	South Asia	86.51	47.82	156.49	9.55	1536
7.	Anyaypoma-Ocon 2021	Latin America & Caribbean	1.11	0.72	1.71	9.92	324
8.	Xiong 2021	East Asia & Pacific	40.44	20.75	78.83	9.34	799
9.	Marcolino 2021	Latin America & Caribbean	3.13	2.37	4.14	10.2	1907
10.	Arana-Calderon 2022	Latin America & Caribbean	26.86	3.43	210.29	5.02	158
11.	Soto 2022	Latin America & Caribbean	5.12	3.99	6.58	10.23	1323
**Overall(I2=94.7%, p<0.05)**			**6.698**	**3.527**	**12.72**		**7787**

Fig. 158: Studies included in odds ratio of mortality (hypoxaemic vs. normoxaemic) among adults with respiratory disease (SpO2<90)

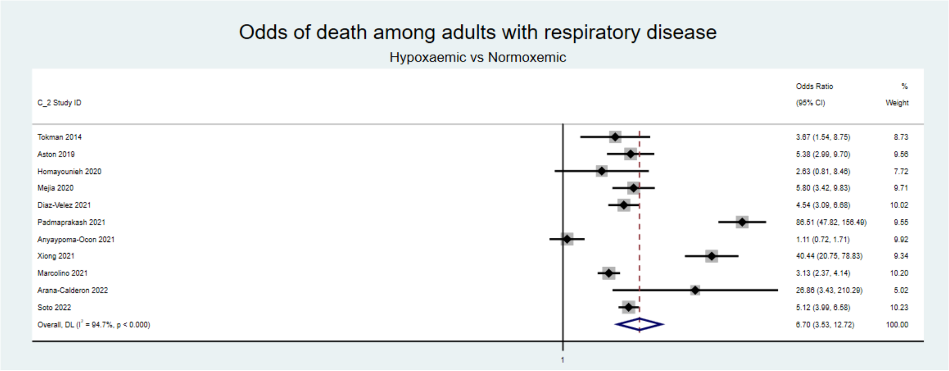

Relative Odds of Death – adults with non-primary respiratory disease

Table 159: Studies included in odds ratio of mortality (hypoxaemic vs. normoxaemic) among adults with non-respiratory disease (SpO2<90)

Serial	StudyID	Region	OR	LCL	UCL	Weight	Total
1.	Amare 2008	Sub Saharan Africa	1.29	0.48	3.50	12	119
2.	Koss 2015	Sub Saharan Africa	2.47	1.63	3.74	20.68	835
3.	Carugati 2018	Sub Saharan Africa	3.83	1.76	8.34	14.98	419
4.	YeLynn 2019	East Asia & Pacific	0.33	0.13	0.83	12.76	118
5.	Boonmee 2020	East Asia & Pacific	2.17	1.72	2.75	23.07	1616
6.	MarMinn 2021	East Asia & Pacific	3.86	1.96	7.60	16.5	507
**Overall(I2=77.2%, p<0.05)**			1.97	1.213	3.2		3614

Fig. 159: Studies included in odds ratio of mortality (hypoxaemic vs. normoxaemic) among adults with non-respiratory disease (SpO2<90)

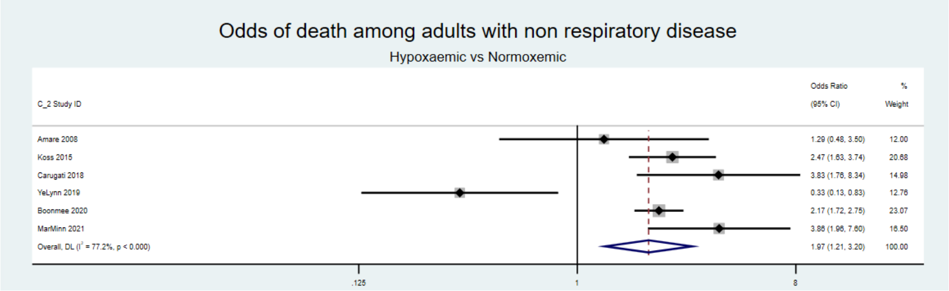

Relative Odds of Death – overall (neonates, children, and adults)

Table 160: Studies included in odds ratio of overall mortality (hypoxaemic vs. normoxaemic) (SpO2<90)

Serial	StudyID	Region	OR	LCL	UCL	Weight	Total
1.	Usen 1999	Sub Saharan Africa	5.10	2.22	11.70	1.73	1072
2.	West 1999	Sub Saharan Africa	2.71	0.77	9.56	1.33	118
3.	Junge 2006	Sub Saharan Africa	7.45	5.48	10.14	2.17	3269
4.	Asghar 2008	Mixed	2.55	1.34	4.85	1.91	958
5.	Amare 2008	Sub Saharan Africa	1.29	0.48	3.50	1.57	119
6.	Mwaniki 2009	Sub Saharan Africa	6.37	5.47	7.43	2.23	15156
7.	Sigauque 2009	Sub Saharan Africa	2.19	1.13	4.25	1.89	584
8.	Chisti 2011	South Asia	8.98	3.02	26.64	1.48	258
9.	Ramakrishna 2012	Sub Saharan Africa	6.52	2.49	17.07	1.6	233
10.	Chisti 2013	South Asia	22.67	5.98	85.92	1.27	140
11.	Cserti-Gazdewich 2013	Sub Saharan Africa	4.79	2.23	10.29	1.79	849
12.	McCollum 2013	Sub Saharan Africa	5.21	2.23	12.19	1.71	761
13.	Orimadegun 2013	Sub Saharan Africa	1.73	1.21	2.46	2.14	1726
14.	Kuti 2013	Sub Saharan Africa	3.05	1.05	8.87	1.5	389
15.	Tokman 2014	Sub Saharan Africa	3.67	1.54	8.75	1.69	241
16.	Orimadegun 2014	Sub Saharan Africa	4.71	2.16	10.27	1.78	369
17.	Koss 2015	Sub Saharan Africa	2.47	1.63	3.74	2.1	835
18.	Abdulkadir 2015	Sub Saharan Africa	61.84	3.66	1044.98	0.5	200
19.	Lowlaavar 2016	Sub Saharan Africa	2.88	1.72	4.81	2.02	1291
20.	Bassat 2016	Sub Saharan Africa	3.00	1.87	4.81	2.06	825
21.	Barennes 2016	East Asia & Pacific	4.45	1.74	11.39	1.62	350
22.	Benet 2017	Mixed	3.83	1.29	11.42	1.48	405
23.	Alwadhi 2017	South Asia	9.34	0.49	177.63	0.47	112
24.	Zampoli 2017	Sub Saharan Africa	7.78	1.01	59.81	0.8	206
25.	Hau 2018	Sub Saharan Africa	4.55	2.12	9.76	1.8	537
26.	Carugati 2018	Sub Saharan Africa	3.83	1.76	8.34	1.78	419
27.	Worodria 2018	Sub Saharan Africa	3.70	2.72	5.03	2.17	1887
28.	Aston 2019	Sub Saharan Africa	5.38	2.99	9.70	1.96	431
29.	McCollum 2019	Sub Saharan Africa	1.02	0.64	1.63	2.06	644
30.	Dembele 2019	East Asia & Pacific	4.14	3.13	5.48	2.18	5023
31.	YeLynn 2019	East Asia & Pacific	0.33	0.13	0.83	1.63	118
32.	Ma 2019	Sub Saharan Africa	3.94	0.50	30.88	0.79	155
33.	Boonmee 2020	East Asia & Pacific	2.17	1.72	2.75	2.2	1616
34.	Hooli 2020	Sub Saharan Africa	4.60	2.32	9.14	1.87	1491
35.	Homayounieh 2020	Middle East & North Africa	2.63	0.81	8.46	1.41	75

36.	Shahunja 2020	South Asia	14.73	7.35	29.52	1.86	401
37.	Wasingya-Kasereka 2020	Sub Saharan Africa	10.19	6.87	15.11	2.11	2599
38.	Fagbohun 2020	Latin America & Caribbean	11.07	6.23	19.66	1.97	860
39.	Mejia 2020	Latin America & Caribbean	5.80	3.42	9.83	2.01	500
40.	Shahrin 2020	South Asia	4.53	1.44	14.27	1.43	176
41.	Diaz-Velez 2021	Latin America & Caribbean	4.54	3.09	6.68	2.12	493
42.	Padmaprakash 2021	South Asia	86.51	47.8 2	156.49	1.95	1536
43.	Bui-Binh-Bao 2021	East Asia & Pacific	69.44	8.21	587.42	0.75	281
44.	Chisti 2021	South Asia	13.55	9.24	19.88	2.12	4007
45.	Anyaypoma-Ocon 2021	Latin America & Caribbean	1.11	0.72	1.71	2.08	324
46.	MarMinn 2021	East Asia & Pacific	3.86	1.96	7.60	1.88	507
47.	Kintwa 2021	East Asia & Pacific	10.49	3.62	30.39	1.51	140
48.	Kayambankadzanja 2021	Sub Saharan Africa	3.92	1.87	8.23	1.82	1135
49.	Xiong 2021	East Asia & Pacific	40.44	20.7 5	78.83	1.89	799
50.	Marcolino 2021	Latin America & Caribbean	3.13	2.37	4.14	2.18	1907
51.	Krithika 2022	South Asia	191.4 5	76.6 1	478.48	1.65	600
52.	Awasthi 2022	South Asia	3.40	2.06	5.59	2.04	7196
53.	Arana-Calderon 2022	Latin America & Caribbean	26.86	3.43	210.29	0.79	158
54.	Kiputa 2022	Sub Saharan Africa	6.18	3.51	10.87	1.98	348
55.	Chisti 2022	South Asia	14.60	9.77	21.83	2.11	3468
56.	King 2022	Sub Saharan Africa	6.38	2.83	14.37	1.75	802
57.	Soto 2022	Latin America & Caribbean	5.12	3.99	6.58	2.2	1323
58.	Kapoor 2022	South Asia	16.95	3.74	76.92	1.12	180
**Overall(I2=90.1%, p<0.05)**			5.323	4.24 4	6.676		7260 2

Fig. 160: Studies included in odds ratio of overall mortality (hypoxaemic vs. normoxaemic) (SpO2<90)

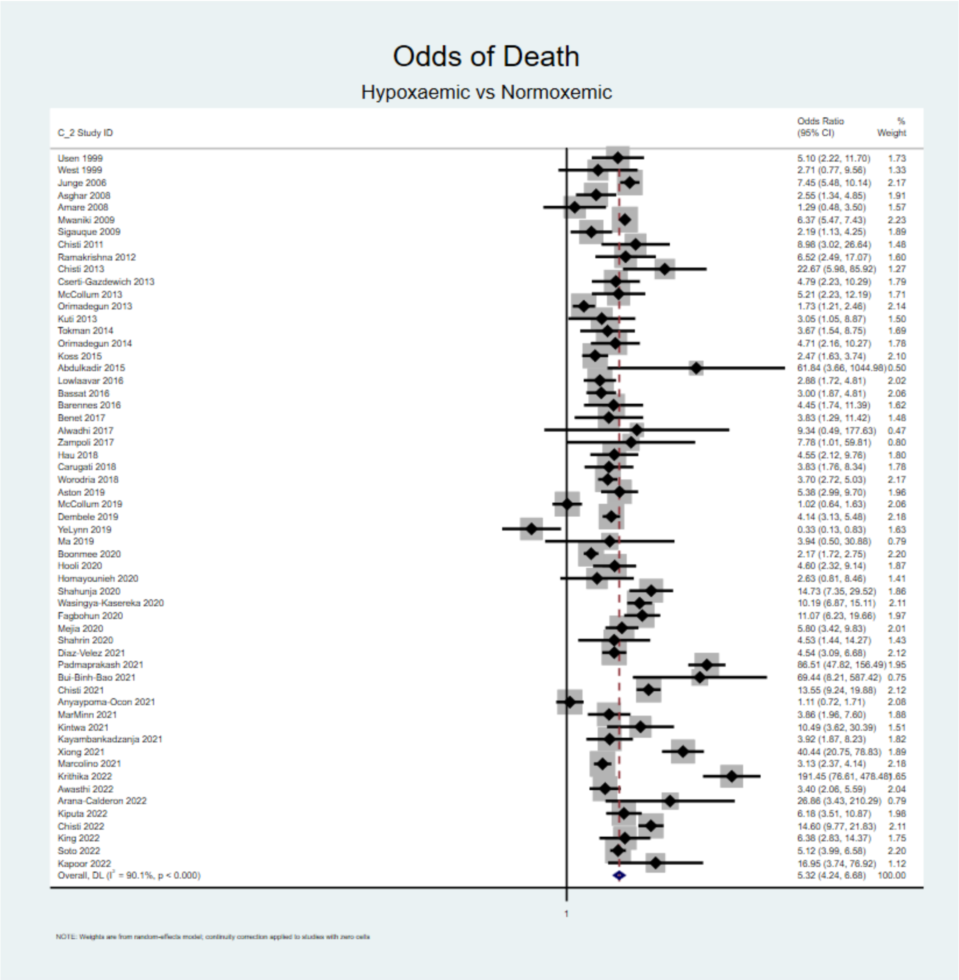

High versus Low-altitude Admitted Children with pneumonia (WHO-classified pneumonia, severe, very severe)

Table 161: Studies included in prevalence of hypoxaemia among children with pneumonia by altitude (sensitivity analysis restricting to SpO2<90)

#	Study ID	WB Region	Proportion	LCL	UCL	Weight	Denom
High							
1.	Jullien 2022	South Asia	75.51	67.74	82.22	1.91	147
Low							
1.	Usen 1999	Sub Saharan Africa	5.88	4.55	7.46	1.99	1072
2.	West 1999	Sub Saharan Africa	43.68	36.52	51.05	1.93	190
3.	Laman 2005	East Asia & Pacific	25.97	16.64	37.23	1.84	77
4.	Wandi 2006	East Asia & Pacific	54.50	50.34	58.61	1.98	578
5.	Fu 2006	Mixed	19.30	17.45	21.26	1.99	1694
6.	Junge 2006	Sub Saharan Africa	11.70	8.83	15.09	1.97	436
7.	Puumalainen 2008	East Asia & Pacific	16.16	14.08	18.42	1.99	1151
8.	Mwaniki 2009	Sub Saharan Africa	8.40	7.68	9.16	2.00	5489
9.	Sigauque 2009	Sub Saharan Africa	25.84	22.60	29.29	1.98	685
10.	Chisti 2011	South Asia	54.55	47.33	61.62	1.94	198
11.	Webb 2012	Sub Saharan Africa	23.59	20.16	27.30	1.98	568
12.	Ramakrishna 2012	Sub Saharan Africa	37.34	31.11	43.89	1.95	233
13.	Jain 2013	South Asia	17.68	12.42	24.03	1.93	181
14.	Kuti 2013	Sub Saharan Africa	19.76	16.01	23.94	1.97	410
15.	McCollum 2013	Sub Saharan Africa	17.22	11.57	24.20	1.92	151
16.	Orimadegun 2013	Sub Saharan Africa	49.20	43.53	54.88	1.96	313
17.	Kuti 2013	Sub Saharan Africa	19.29	15.62	23.39	1.97	420
18.	Sempertegui 2014	Latin America & Caribbean	85.11	81.48	88.27	1.97	450
19.	Ibraheem 2014	Sub Saharan Africa	41.50	34.59	48.66	1.94	200
20.	Abdulkadir 2015	Sub Saharan Africa	41.50	34.59	48.66	1.94	200
21.	Breiman 2015	Sub Saharan Africa	35.74	33.88	37.63	2.00	2563

22.	Kelly 2015	Sub Saharan Africa	38.66	32.43	45.16	1.95	238
23.	Basnet 2015	South Asia	61.15	57.15	65.04	1.98	610
24.	Salah 2015	Sub Saharan Africa	42.67	34.64	50.99	1.92	150
25.	Bassat 2016	Sub Saharan Africa	27.88	24.84	31.07	1.99	825
26.	Alwadhi 2017	South Asia	50.89	41.27	60.46	1.89	112
27.	Graham 2019	Sub Saharan Africa	23.44	21.64	25.33	2.00	2073
28.	McCollum 2019	Sub Saharan Africa	64.44	60.61	68.14	1.98	644
29.	Ashraf 2019	South Asia	10.64	8.00	13.78	1.97	470
30.	Dembele 2019	East Asia & Pacific	13.52	12.50	14.59	2.00	4179
31.	Ma 2019	Sub Saharan Africa	85.81	79.30	90.89	1.92	155
32.	Fashanu 2020	Sub Saharan Africa	58.38	55.84	60.90	1.99	1497
33.	Hooli 2020	Sub Saharan Africa	14.69	12.93	16.59	1.99	1491
34.	Fagbohun 2020	Latin America & Caribbean	13.02	10.85	15.46	1.99	860
35.	Shahrin 2020	South Asia	15.18	10.41	21.07	1.93	191
36.	Muro 2020	Sub Saharan Africa	32.40	26.64	38.58	1.95	250
37.	Oktaria 2021	East Asia & Pacific	13.53	8.22	20.54	1.91	133
38.	Bui-Binh-Bao 2021	East Asia & Pacific	11.39	7.92	15.69	1.96	281
39.	Chisti 2021	South Asia	28.13	26.74	29.55	2.00	4007
40.	Rahman 2021	South Asia	39.98	38.11	41.88	2.00	2646
41.	Ahmed 2022	Sub Saharan Africa	75.32	64.18	84.44	1.84	77
42.	Awasthi 2022	South Asia	35.85	34.74	36.97	2.00	7196
43.	Chisti 2022	South Asia	31.23	29.69	32.80	2.00	3468
44.	Kapoor 2022	South Asia	36.11	29.10	43.59	1.93	180
45.	Mvalo 2022	Sub Saharan Africa	63.75	59.53	67.82	1.98	538
46.	Zar 2022	Sub Saharan Africa	24.38	18.61	30.92	1.94	201
Overall (I2=99.32, T2=0.14, p<0.05)			32.74	27.67	38.01		49731
Mixed							
8376	Addo-Yobo 2004	Mixed	19.10	17.25	21.04	1.99	1702
	Duke 2008	East Asia & Pacific	57.09	52.94	61.17	1.98	578
1213	Asghar 2008	Mixed	64.61	61.49	67.65	1.99	958

119	Muller 2012	Latin America & Caribbean	22.48	20.66	24.38	2.00	1993
Overall (I2=99.63, T2=0.22, p<0.05)			39.90	19.12	62.79		5231

Figure 161: Studies included in prevalence of hypoxaemia among children with pneumonia by altitude (sensitivity analysis restricted to SpO2<90)

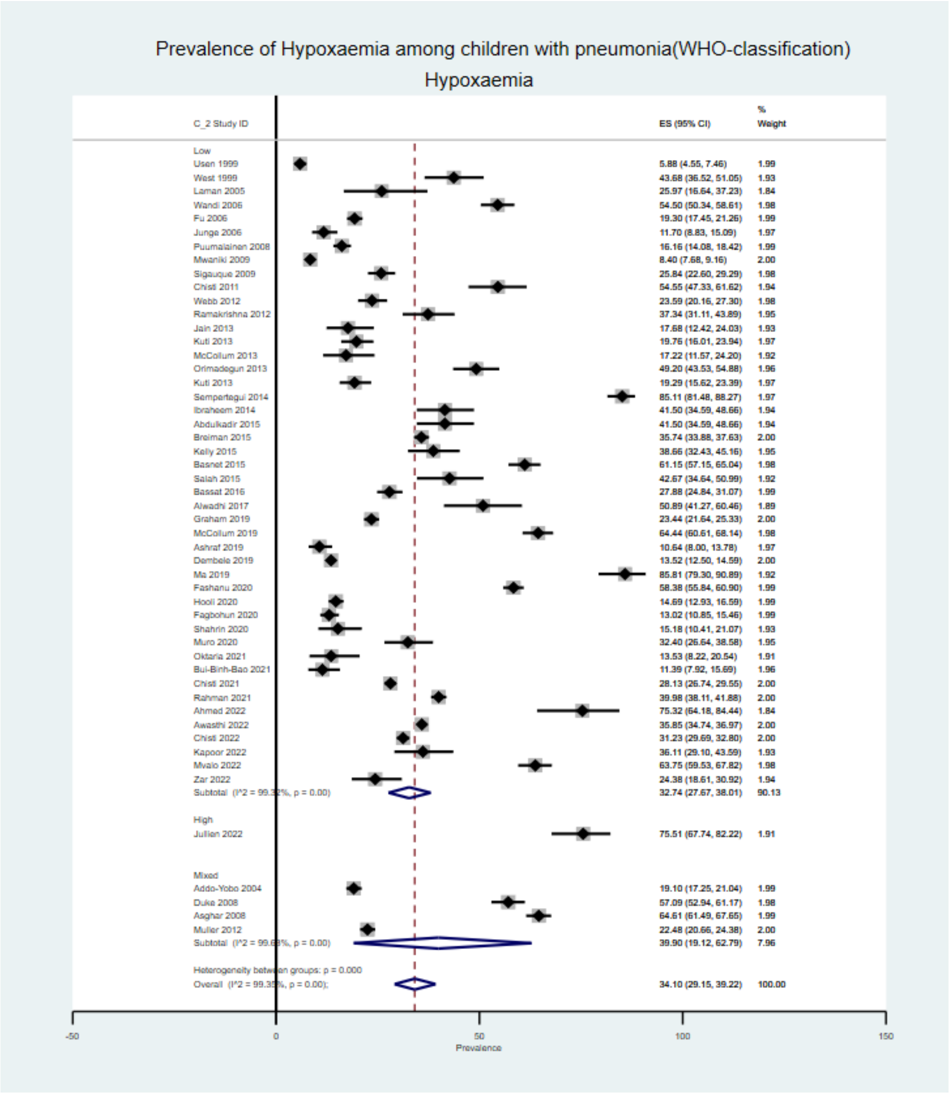

Regional breakdown Admitted Children with pneumonia (WHO-classified pneumonia, severe, very severe)

Table 162: Studies included in sub-region-wise prevalence of hypoxaemia among children with pneumonia (SpO2<90)

Sl no	Study ID	Proportion	LCL	UCL	Weight	Denom
East Asia & Pacific						
1.	Laman 2005	25.97	16.64	37.23	1.84	77
2.	Wandi 2006	54.50	50.34	58.61	1.98	578
3.	Puumalainen 2008	16.16	14.08	18.42	1.99	1151
4.	Duke 2008	57.09	52.94	61.17	1.98	578
5.	Dembele 2019	13.52	12.50	14.59	2.00	4179
6.	Oktaria 2021	13.53	8.22	20.54	1.91	133
7.	Bui-Binh-Bao 2021	11.39	7.92	15.69	1.96	281
Overall (I2=99.27, T2=0.20, p<0.05)		25.92	12.89	41.58		6977
Latin America & Caribbean						
1.	Muller 2012	22.48	20.66	24.38	2.00	1993
2.	Sempertegui 2014	85.11	81.48	88.27	1.97	450
3.	Fagbohun 2020	13.02	10.85	15.46	1.99	860
Overall (I2=99.76, T2=0.46, p<0.05)		39.40	8.37	76.38		3303
Mixed						
1.	Addo-Yobo 2004	19.10	17.25	21.04	1.99	1702
2.	Fu 2006	19.30	17.45	21.26	1.99	1694
3.	Asghar 2008	64.61	61.49	67.65	1.99	958
Overall (I2=99.71, T2=0.24, p<0.05)		33.14	10.72	60.69		4354
South Asia						
1.	Chisti 2011	54.55	47.33	61.62	1.94	198
2.	Jain 2013	17.68	12.42	24.03	1.93	181
3.	Basnet 2015	61.15	57.15	65.04	1.98	610
4.	Alwadhi 2017	50.89	41.27	60.46	1.89	112
5.	Ashraf 2019	10.64	8.00	13.78	1.97	470
6.	Shahrin 2020	15.18	10.41	21.07	1.93	191
7.	Chisti 2021	28.13	26.74	29.55	2.00	4007
8.	Rahman 2021	39.98	38.11	41.88	2.00	2646
9.	Awasthi 2022	35.85	34.74	36.97	2.00	7196
10.	Chisti 2022	31.23	29.69	32.80	2.00	3468
11.	Jullien 2022	75.51	67.74	82.22	1.91	147
12.	Kapoor 2022	36.11	29.10	43.59	1.93	180
Overall (I2=98.40, T2=0.05, p<0.05)		36.86	30.92	42.99		19406

Sub Saharan Africa						
1.	Usen 1999	5.88	4.55	7.46	1.99	1072
2.	West 1999	43.68	36.52	51.05	1.93	190
3.	Junge 2006	11.70	8.83	15.09	1.97	436
4.	Mwaniki 2009	8.40	7.68	9.16	2.00	5489
5.	Sigauque 2009	25.84	22.60	29.29	1.98	685
6.	Webb 2012	23.59	20.16	27.30	1.98	568
7.	Ramakrishna 2012	37.34	31.11	43.89	1.95	233
8.	Kuti 2013	19.76	16.01	23.94	1.97	410
9.	McCollum 2013	17.22	11.57	24.20	1.92	151
10.	Orimadegun 2013	49.20	43.53	54.88	1.96	313
11.	Kuti 2013	19.29	15.62	23.39	1.97	420
12.	Ibraheem 2014	41.50	34.59	48.66	1.94	200
13.	Abdulkadir 2015	41.50	34.59	48.66	1.94	200
14.	Breiman 2015	35.74	33.88	37.63	2.00	2563
15.	Kelly 2015	38.66	32.43	45.16	1.95	238
16.	Salah 2015	42.67	34.64	50.99	1.92	150
17.	Bassat 2016	27.88	24.84	31.07	1.99	825
18.	Graham 2019	23.44	21.64	25.33	2.00	2073
19.	McCollum 2019	64.44	60.61	68.14	1.98	644
20.	Ma 2019	85.81	79.30	90.89	1.92	155
21.	Fashanu 2020	58.38	55.84	60.90	1.99	1497
22.	Hooli 2020	14.69	12.93	16.59	1.99	1491
23.	Muro 2020	32.40	26.64	38.58	1.95	250
24.	Ahmed 2022	75.32	64.18	84.44	1.84	77
25.	Mvalo 2022	63.75	59.53	67.82	1.98	538
26.	Zar 2022	24.38	18.61	30.92	1.94	201
Overall (I2=99.36, T2=0.21, p<0.05)		34.58	26.47	43.17		21069

Figure 162: Studies included in sub-region-wise prevalence of hypoxaemia among children with pneumonia (SpO2<90)

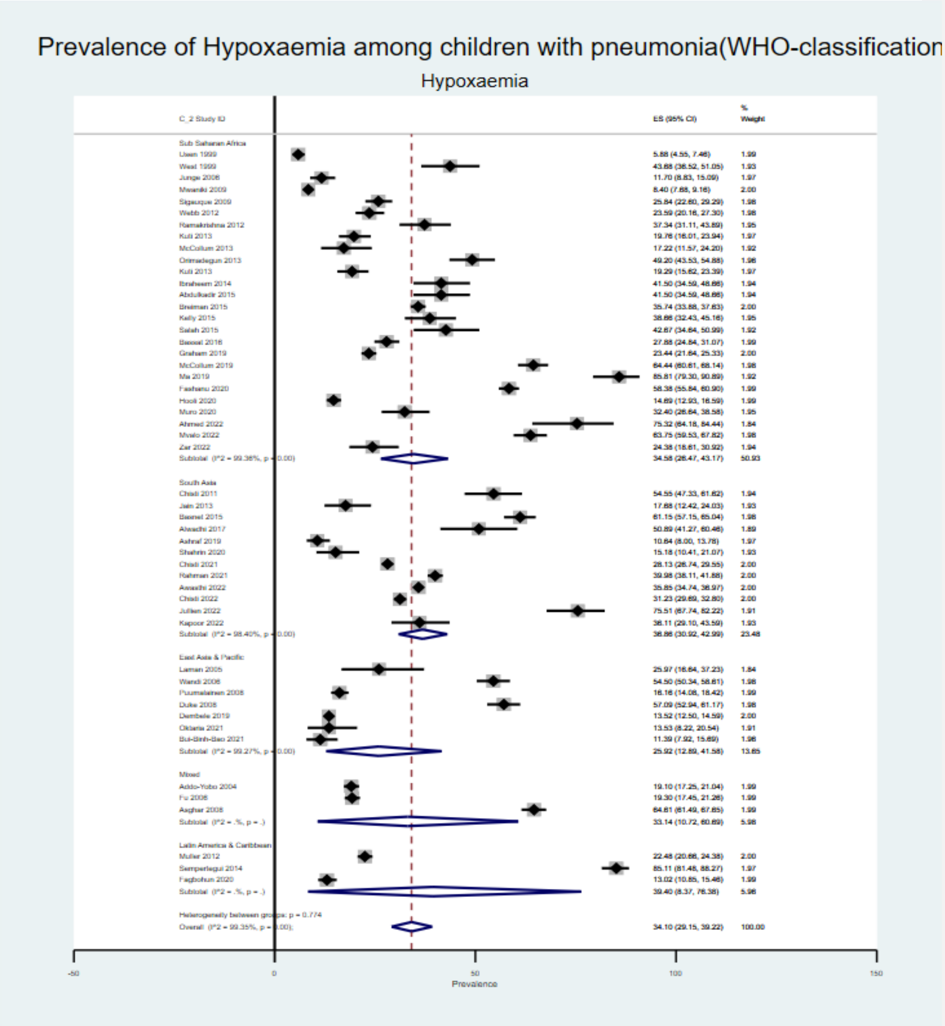

sss

References

1. Duke T, Blaschke aJ, Sialis S, Bonkowsky JL. Hypoxaemia in acute respiratory and non-respiratory illnesses in neonates and children in a developing country. *Arch Dis Child* 2002; **86**: 108-12.
2. Graham HR, Kamuntu Y, Miller J, et al. Hypoxaemia prevalence and management among children and adults presenting to primary care facilities in Uganda: A prospective cohort study. *PLOS Glob Public Health* 2022; **2**(4): e0000352.
3. Graham H, Bakare AA, Ayede AI, et al. Hypoxaemia in hospitalised children and neonates: A prospective cohort study in Nigerian secondary-level hospitals. *EClinicalMedicine* 2019; **16**: 51-63.
4. Junge S, Palmer A, Greenwood BM, Kim Mulholland E, Weber MW. The spectrum of hypoxaemia in children admitted to hospital in The Gambia, West Africa. *Tropical Medicine and International Health* 2006; **11**: 367-72.
5. King C, Zadutsa B, Banda L, et al. Prospective cohort study of referred Malawian children and their survival by hypoxaemia and hypoglycaemia status. *Bull World Health Organ* 2022; **100**(5): 302-14B.
6. McCollum ED, Bjornstad E, Preidis GA, Hosseinipour MC, Lufesi N. Multicenter study of hypoxemia prevalence and quality of oxygen treatment for hospitalized Malawian children. *Trans R Soc Trop Med Hyg* 2013; **107**(5): 285-92.
7. Mwaniki MK, Nokes DJ, Ignas J, et al. Emergency triage assessment for hypoxaemia in neonates and young children in a Kenyan hospital: an observational study. *Bull World Health Organ* 2009; **87**(4): 263-70.
8. Orimadegun AE, Ogunbosi BO, Carson SS. Prevalence and predictors of hypoxaemia in respiratory and non-respiratory primary diagnoses among emergently ill children at a tertiary hospital in south western Nigeria. *Trans R Soc Trop Med Hyg* 2013; **107**(11): 699-705.
9. Wandi F, Peel D, Duke T. Hypoxaemia among children in rural hospitals in Papua New Guinea: epidemiology and resource availability--a study to support a national oxygen programme. *Ann Trop Paediatr* 2006; **26**: 277-84.
10. Borges Migliavaca C, Stein C, Colpani V, et al. How are systematic reviews of prevalence conducted? A methodological study. *BMC Med Res Methodol* 2020; **20**(1): 96.
11. Munn Z, Moola S, Riitano D, Lisy K. The development of a critical appraisal tool for use in systematic reviews addressing questions of prevalence. *Int J Health Policy Manag* 2014; **3**(3): 123-8.
12. Iorio A, Spencer FA, Falavigna M, et al. Use of GRADE for assessment of evidence about prognosis: rating confidence in estimates of event rates in broad categories of patients. *Bmj* 2015; **350**: h870.
